# Repurposing and computational design of PARP inhibitors as SARS-CoV-2 inhibitors

**DOI:** 10.1038/s41598-023-36342-7

**Published:** 2023-06-29

**Authors:** Shailima Rampogu, Tae Sung Jung, Min Woo Ha, Keun Woo Lee

**Affiliations:** 1 Department of Bio and Medical Big Data (BK4 Program), Division of Life Sciences, Research Institute of Natural Science (RINS), Gyeongsang National University (GNU), Jinju, Republic of Korea; 2 Laboratory of Aquatic Animal Diseases, College of Veterinary Medicine, Research Institute of Natural Science, Gyeongsang National University, Jinju, 52828 Republic of Korea; 3 Interdisciplinary Graduate Program in Advanced Convergence Technology and Science, Jeju National University, 102 Jejudaehak-ro, Jeju, 63243 Republic of Korea

**Keywords:** Virtual drug screening, Drug discovery and development

## Abstract

Coronavirus disease 2019 (COVID-19) is a recent pandemic that caused serious global emergency. To identify new and effective therapeutics, we employed a drug repurposing approach. The poly (ADP ribose) polymerase inhibitors were used for this purpose and were repurposed against the main protease (Mpro) target of severe acute respiratory syndrome Coronavirus 2 (SARS-CoV-2). The results from these studies were used to design compounds using the ‘*Grow Scaffold*’ modules available on Discovery Studio v2018. The three designed compounds, olaparib 1826 and olaparib 1885, and rucaparib 184 demonstrated better CDOCKER docking scores for Mpro than their parent compounds. Moreover, the compounds adhered to Lipinski’s rule of five and demonstrated a synthetic accessibility score of 3.55, 3.63, and 4.30 for olaparib 1826, olaparib 1885, and rucaparib 184, respectively. The short-range Coulombic and Lennard-Jones potentials also support the potential binding of the modified compounds to Mpro. Therefore, we propose these three compounds as novel SARS-CoV-2 inhibitors.

## Introduction

Recent Coronavirus disease 2019 (COVID-19) events have led the world into unprecedented circumstances^1,2^. COVID-19 is caused by the severe acute respiratory syndrome Coronavirus 2 (SARS-CoV-2). It belongs to the family Coronaviridae and subfamily Orthocoronavirinae and consists of four genera^3^. The genome of SARS-CoV-2 is 30 kb in size, and codes for a large non-structural polyprotein that further generates 15/16 proteins, five accessory proteins, and four structural proteins after proteolytic cleavage^3^.

The identification of new inhibitors with effective and quick therapeutics is still in progress for this disease. An approach to discovering novel inhibitors is to repurpose approved drugs to identify new indications for older drugs^4^. This approach is one of the most effective methods for drug discovery^5^. Drug repurposing (DR) also called drug reprofiling, drug recycling, drug rescue, therapeutic switching, drug retasking, and drug redirection^5^. Many studies have adopted this approach to discover COVID-19 therapeutics, which has resulted in the discovery of plausible inhibitors^6–9^. Several studies have also reported the intervention of computational approaches in detecting SARS-CoV-2 inhibitors using predominantly molecular docking, molecular dynamics simulation, and pharmacophore modelling studies^10–24^.

In this study, we applied the DR approach to a SARS-CoV-2 main protease (Mpro) target using PARP inhibitors. PARP enzymes contribute greatly to genome stability, and their inhibitors occupy the catalytic domain of PARP, hindering poly ADP-ribosylation (PARylation) of target proteins^25^.

The present study is focused on Mpro, as it has the largest active site volume (Supplementary Fig. 1). The SARS-CoV-2 Mpro processes the proteolytic step during the replication of the virus. This 33.8-kDa protein, also known as 3-chymotrypsin-like protease (3C-like protease), is a promising target for developing and identifying new inhibitors^26–29^. The homodimer Mpro consists of two protomers^30^. Mpro consists of 306 amino acid residues divided into three domains^31^. The residues 8–101 form domain I; residues 102–184 form domain II; and residues 201–303 form domain III^26,30,32^. Domain III is connected to domain II by residues 185–200.

A Cys-His catalytic dyad is present between domains I and domain II^26,30–32^. The S1 subsite residues were Phe140, Leu141, Asn142, His163, Glu166, and His172. The residues in S1 were Thr25, Thr26, and Leu27^33^. The hydrophobic S2 subsite consists of His41, Met49, Tyr54, Met165, and Asp187 residues. The S4 binding subsite contains Met165, Leu167, Phe185, Gln189, and Gln192^33,34^. The residues Cys145-His41 form the catalytic dyad^33,34^. All these residues bind to the ligand and are termed key residues.

In the present study, we used approved PARP inhibitors to target COVID-19 Mpro. PARP inhibitors exhibit anticancer ability^35–37^. It is reported that the PARP inhibitors can reduce the levels of interleukin 1 (IL‐1), interleukin 6 (IL6), and tumour necrosis factor alpha (TNF‐α); and decrease lung fibrosis^38^. These inhibitors act against cell death promoted by inflammation, thereby favouring cell survival^38^. In another study, computational and experimental analyses revealed that the PARP1 inhibitor CVL218 is a potential COVID-19 inhibitor^39^. Another study reported that the in vitro PARP inhibitor tenoparib prevented the replication of SARS-CoV-2 and human coronavirus NL63 (HCoV-NL63)^40^. The PARP inhibitor mefuparib has been reported to bind to the nucleocapsid (N) protein^39^ and inhibit SARS-CoV-2 replication with a half maximal inhibitory concentration (IC_50_) of 5.12 µM^41^. These findings highlight the potential of novel PARP inhibitors in treating COVID-19.

**Figure 1:**
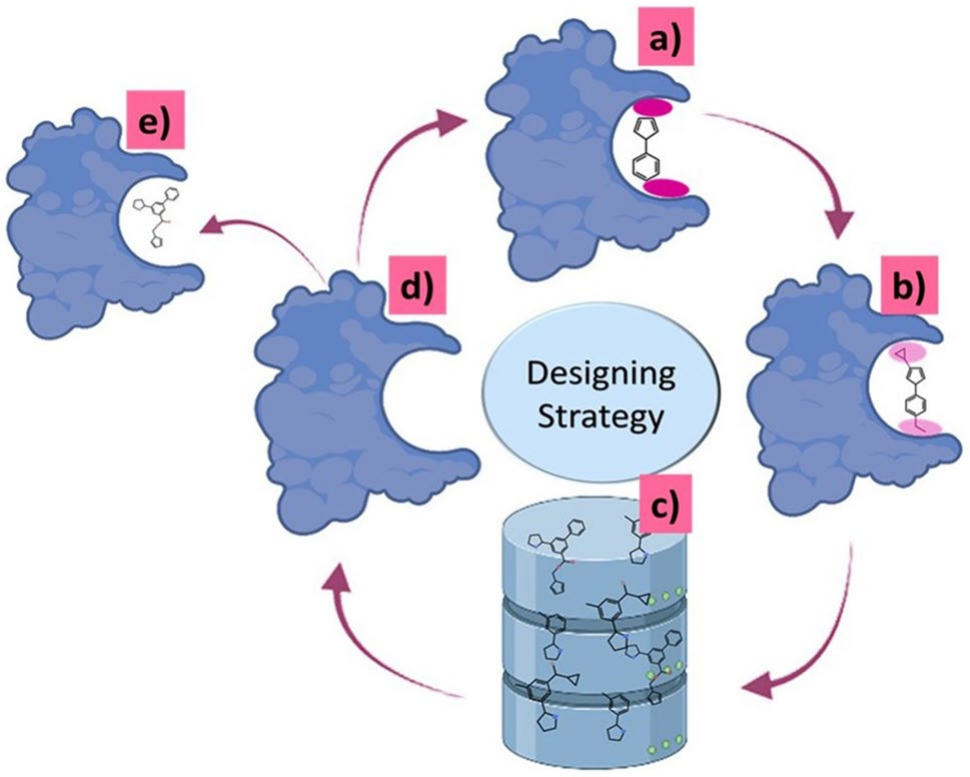
Illustration presenting the study methodology. (**a**) Identification of the unoccupied space in the active site of Mpro. (**b**) Molecular docking of the known PARP inhibitors at the active site of Mpro and applying *Grow Scaffold* to design the compounds (pink oval). (**c**) Database creation with compounds obtained from *Grow Scaffold.* (**d**) Target ready for molecular docking. (**e**) Obtaining a protein- ligand complex with modified compounds. The icons are taken from BioRender.com and are modified accordingly.

Based on these reports, we attempted to computationally design new PARP compounds with high affinity for SARS-CoV-2 targets by using *Grow Scaffold*, a drug design approach. This approach locates spaces within the active site after molecular docking to grow the scaffold. Since we selected PARP inhibitors for this study, we diligently sought compounds that exhibited greater affinity towards the COVID-19 Mpro target. The infographic workflow is illustrated in Fig. 1.

## Results

### Step 1: Molecular docking based binding affinity studies to identify the empty spaces in the binding pocket

Molecular docking was performed using the validated SARS-CoV-2 target, Mpro. All the chosen compounds, except Veliparib and Pamiparib, demonstrated a high docking score compared to the reference compound (co-crystallised ligand). Based on the docking scores, the high-scoring compounds, rucaparib and olaparib, were considered for further studies (Supplementary Table 1). Here, molecular docking was performed to discover compounds with better docking scores towards the COVID-19 target and to select empty space for designing new compounds.

### Strategy to design new compounds

To design a novel compound, the target-ligand active site of Mpro was scrupulously examined, as it had a large volume. After careful visual analysis using DS, we identified two empty sites with olaparib (site 1 and site 2) and one empty site with rucaparib (site 1) and called them *R*_*1*_ (Supplementary Fig. 2)_*.*_

**Figure 2:**
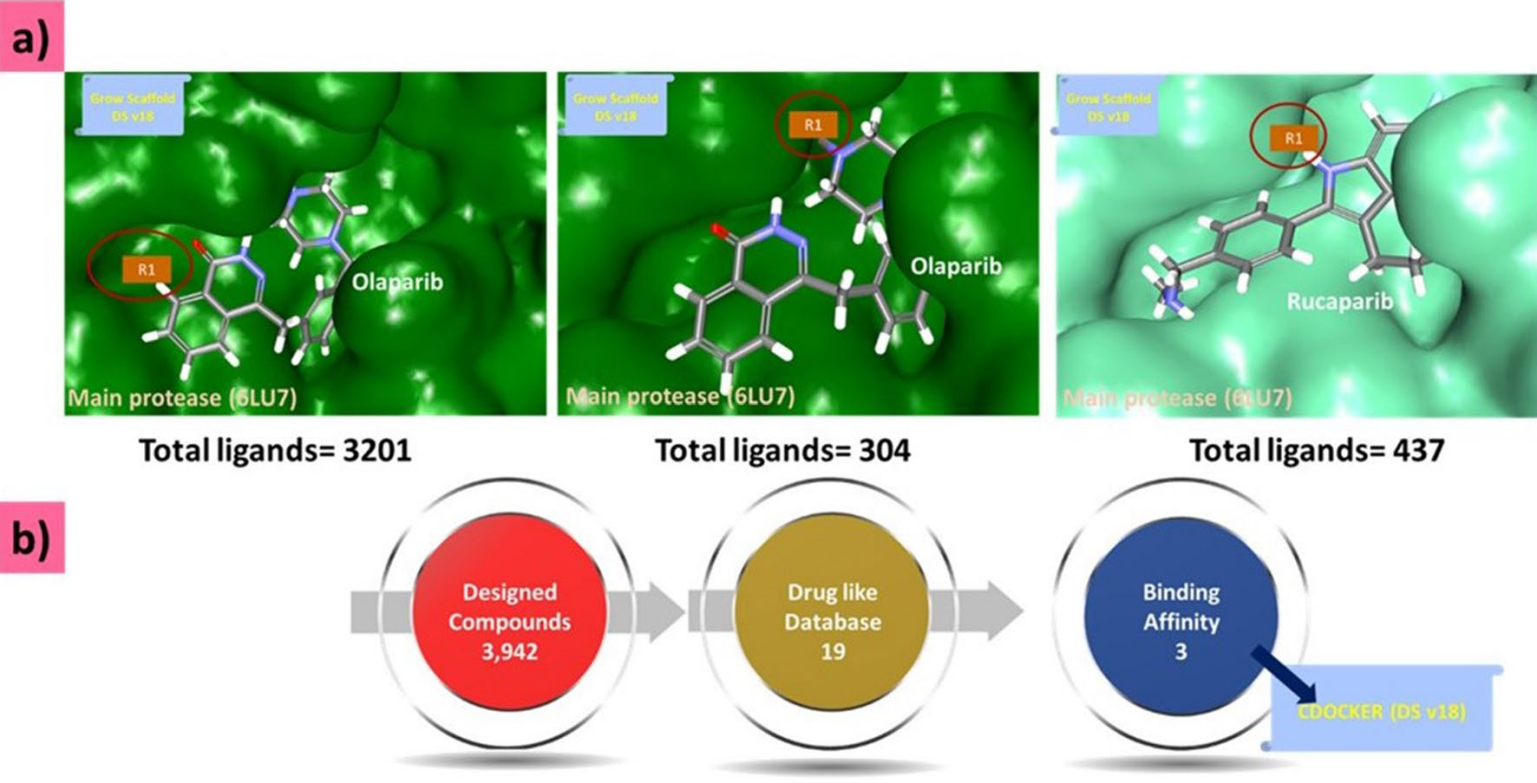
Generation of novel compounds and drug-like database creation. (**a**) Marking the site for growing the scaffold in the protein–ligand complex. (**b**) Screening the compound for molecular docking.

At the identified *R*_*1*_ position, we applied the “Grow Scaffold” approach, which has inbuilt libraries that facilitate the addition of groups to the small molecule (Supplementary Fig. 3). The olaparib site 1 generated a total of 3,201 compounds, site 2 generated 304 compounds; and site 1 of rucaparib generated 437 compounds, accounting for 3942 compounds after these identified sites were subjected to *Grow Scaffold* modules (Fig. 2a). The simplified molecular-input line-entry system (SMILES) representations of these compounds are provided in (Supplementary Table 2).

The 3942 compounds were subjected to Lipinski’s rule of five, enabling the ‘*Filter by Lipinski and Veber Rules*’ protocol. According to the “*Rule of Five*”, a drug-like molecule should have maximum five hydrogen bond donors, 10 hydrogen bond acceptors, a molecular weight less than 500 Dalton, and a LogP value less than five. This process resulted in the production of 19 compounds. These compounds were subsequently docked to Mpro to delineate their binding affinities (Fig. 2b).

### Step 2: Binding affinity studies and clustering of small molecules

**Figure 3:**
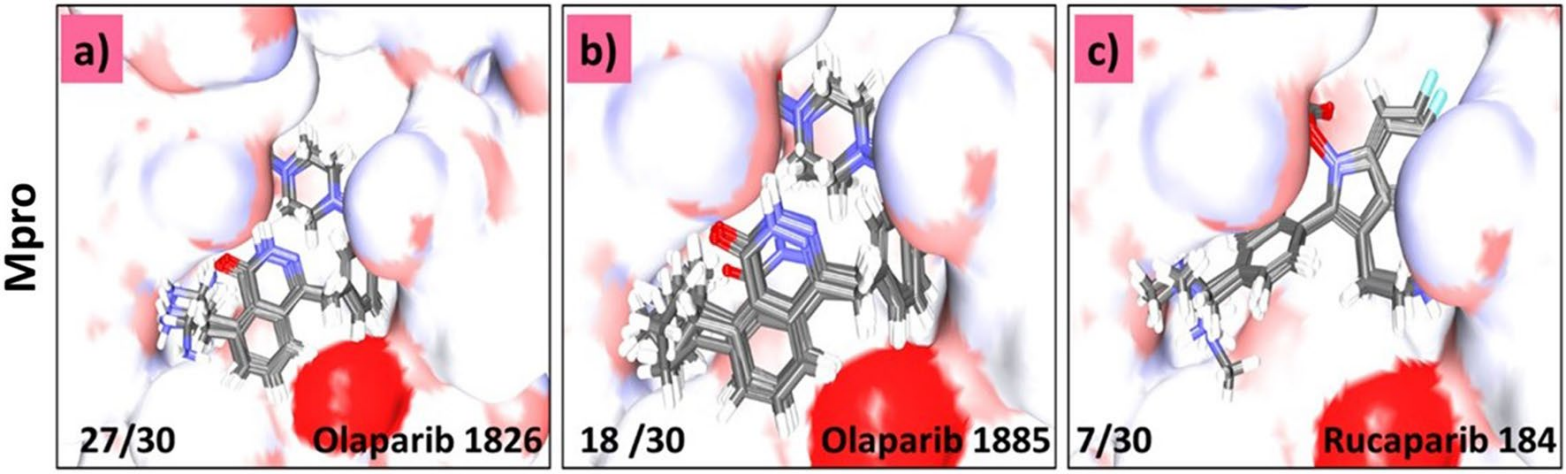
Clustering of small molecules at the active site of Mpro. (**a**) Olaparib1826; (**b**) Olaparib1885; (**c**) Rucaparib 184.

In this step, all 19 modified compounds were docked into the Mpro-binding pocket. The results revealed that three compounds displayed higher binding affinities than their parent structures (Table 1 and Supplementary Table 1). Manual clustering of the compounds was performed to understand the prospective binding modes of the small molecules (Fig. 3). The pose with the best docking score from the largest cluster was further examined using molecular dynamics simulation (MDS).

**Table 1:** Various intermolecular interactions of the modified compounds and the target Mpro.

-CDOCKER interaction energy (kcal/mol)	Various intermolecular interactions			
Compounds	Mpro (6LU7)	Hydrogen bond interactions	Alkyl/π-alkyl interactions	Van der Waals interactions
Olaparib 1826	58.89	Gly143 and Gln189	His41, Cys145, His164, and Met165	Met49, Phe140, Leu141, Asn142, Ser144, His163, Glu166, His172, Asp187, and Arg188
Olaparib 1885	57.51	Gly143, Ser144, and Cys145	His41, Cys145, and Met164	Met49, Phe140, Leu141, Glu166, His172, Asp187, Arg188, and Glu189
Rucaparib 184	58.80	Arg40, His41, and Gln192	Met49, His 41, and Met165	Thr45, Ser46, Tyr54, Cys85, Glu166, Leu167, Pro168, Phe181, Thr190, and Ala191

The two Olaparib-modified compounds were well aligned within the binding pocket of the target. Olaparib 1826 formed 27 poses in a cluster (Fig. 3a), whereas olaparib 1885 formed 18 poses in a cluster (Fig. 3b). Whereas, rucaparib184 formed a cluster with 7 poses (Fig. 3c). These results indicate that olaparib 1826 may have a higher affinity for Mpro, followed by olaparib 1885 and rucaparib184.

The three compounds demonstrated satisfactory results, with higher docking scores than the parent compounds (Table 1 and Supplementary Table 1). The parent compounds, olaparib and rucaparib, generated dock scores of 50.38 kcal/mol and 58.80 kcal/mol, respectively. The modified olaparib compounds, olaparib 1826 and olaparib 1885, demonstrated a dock score of 58.89 kcal/mol and 57.51 kcal/mol, respectively. Similarly, the modified rucaparib compound, rucaparib 184, had a dock score of 58.80 kcal/mol. These findings suggest that the modified compounds demonstrate a stronger affinity for Mpro than the parent structure (Table 1 and Supplementary Table 1).

From the largest cluster, the compounds with the best docking scores were upgraded to molecular dynamics simulation (MDS) to understand the binding potential of the modified compounds to the binding pocket of the target.

### MDS analysis

MDS was performed for 100 ns to analyse the binding potential of the ligands at the binding pocket of the targets. The MDS analysis was based on the root mean square deviation (RMSD), radius of gyration (Rg), root mean square fluctuation (RMSF), and hydrogen bond number.

#### Stability analysis by RMSD

**Figure 4:**
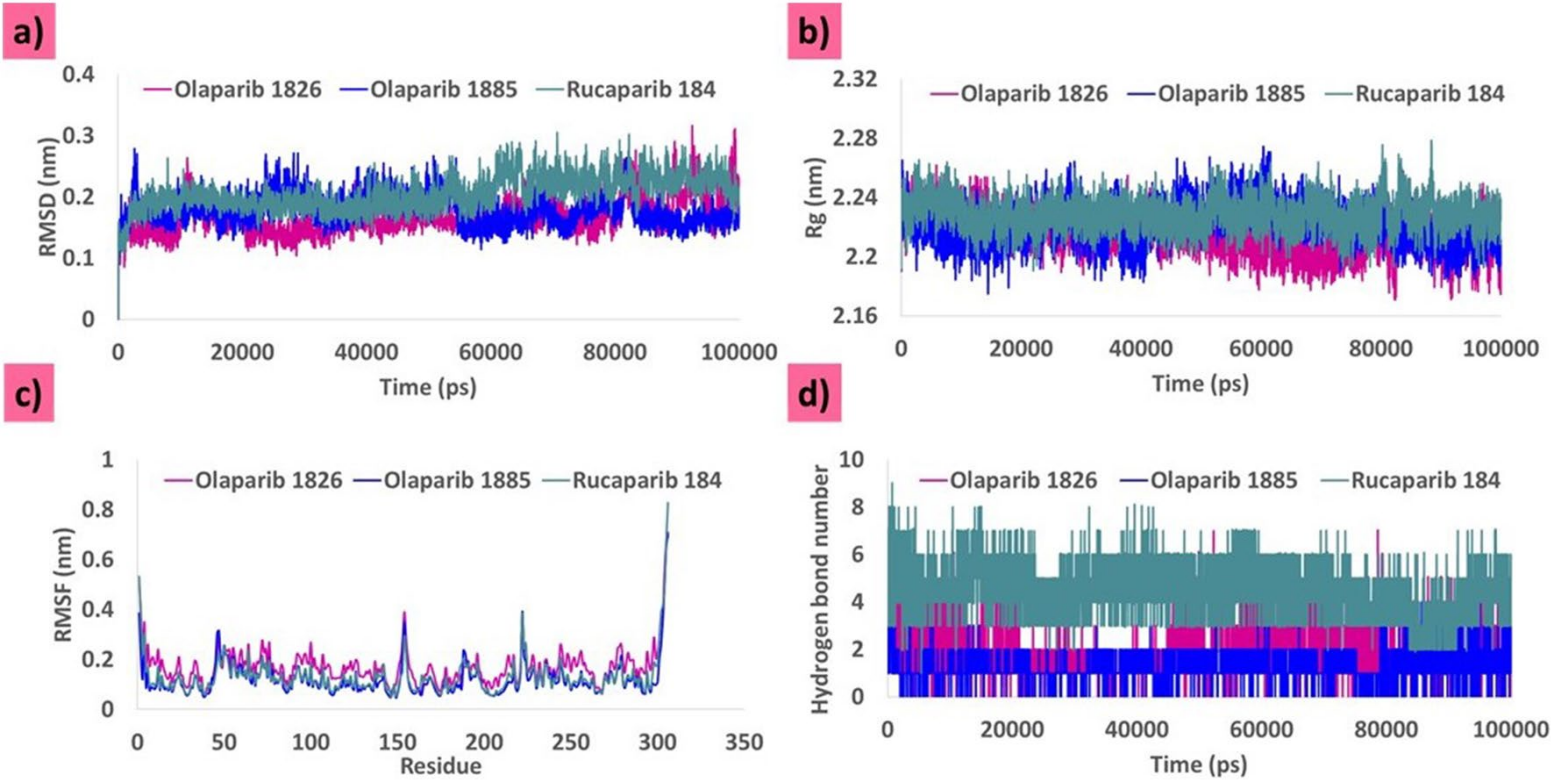
MDS results for the three systems. (**a**) Deviation analysis of the three systems according to RMSD. (**b**) Compactness analysis according to Rg. (**c**) Fluctuation analysis of the three systems according to RMSF. (**d**) Analysis to determine the number of hydrogen bonds for the three systems.

The protein backbone demonstrated stability^42^ throughout the simulation. All the systems converged well and exhibited RMSD below 0.3 nm. The system with olaparib 1826 was largely stable during MDS evolution. At ~ 11,000 ps, a minute surge in the RMSD profiles was noted, and thereafter the system remained steady with an average of 0.16 nm (Fig. 4a). The RMSD profiles of olaparib 1885 and rucaparib 184 were stable throughout the MDS evolution without any noticeable variations, with an average of 0.17 nm and 0.2 nm, respectively (Fig. 4a).

#### Compactness analysis by Rg

Rg is “defined as the ratio of the protein accessible surface area to that of an ideal sphere of the same volume”^43^. This principle governs the protein-folding mechanism^43^. Our analysis revealed that Rg defines the distance between each atom of a protein and its centroid, thereby defining its compactness^44^. These three systems showed that the protein backbone was highly stable and compact. The readings exhibit a range of 2.20 nm to 2.28 nm with an average of 2.21 nm, 2.22 nm, and 2.22 nm for olaparib 1826, olaparib 1885, and rucaparib 184, respectively (Fig. 4b). This finding demonstrates that the systems were remarkably compact.

### Fluctuation analysis by RMSF

The RMSF plots reveal fluctuations during the MDS run. All the systems were stable, with no major fluctuations. The systems were stable with RMSF below 0.3 nm (Fig. 4c). The average RMSF was 0.17 nm, 0.12 nm, and 0.13 nm for olaparib 1826, olaparib 1885, and rucaparib 184, respectively (Fig. 4c). This result indicates that each residue in the system was stable.

### Number of hydrogen bonds

The number of hydrogen bonds between the protein and ligand was assessed during the entire simulation run. The presence of stable interactions suggested that ligands were held within the binding pocket throughout the simulation run. The three systems demonstrated hydrogen bond interactions during the entire simulation run, with an average of 2.0 for olaparib 1826, 1.3 for olaparib 1885, and 4.4 for rucaparib 184 (Fig. 4d). Rucaparib184 demonstrated a higher number of hydrogen bonds than the olaparib derivatives (Fig. 4d). This result implied that the compounds were firmly presented in the binding pocket of the protein.

### Binding mode analysis

**Figure 5:**
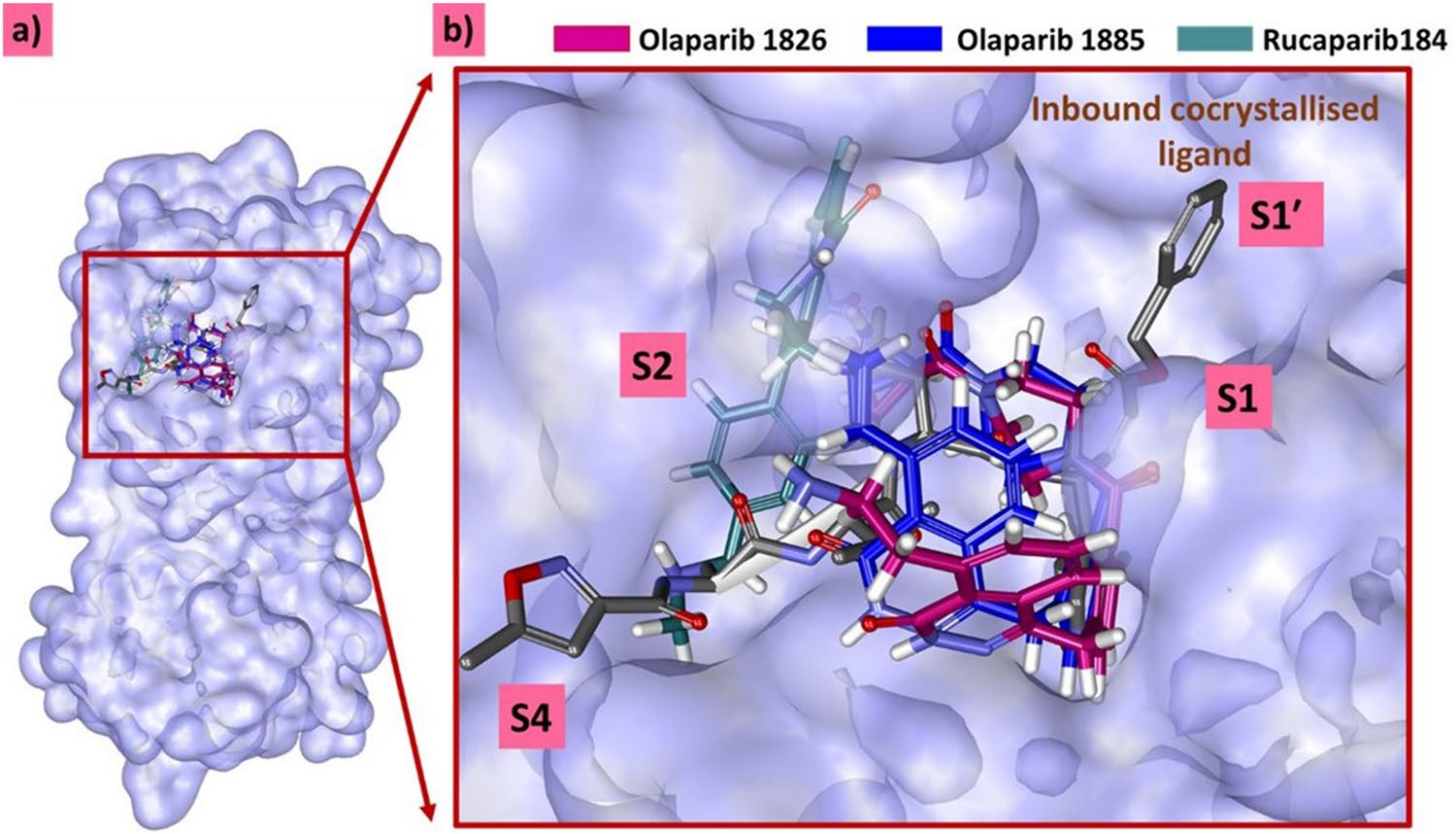
Superimposition of MDS derived structures against the X-ray structure. (**a**) Compounds present at the Mpro binding pocket. (**b**) The zoomed version of the accommodation of the compounds at the binding pocket.

From the stable RMSD of the last 10 ns (~ 90,000–100,000 ps), representative complex structures were extracted and superimposed onto the X-ray crystal structure of Mpro. The results demonstrated that the ligands occupied a binding pocket similar to that of the co-crystallised ligand (Fig. 5), held by various intermolecular interactions. The modified compounds settled at different subsites of the target-binding pocket held by various interactions.

### Key residue interaction of the modified compounds with the target residues

#### Olaparib1826

**Figure 6:**
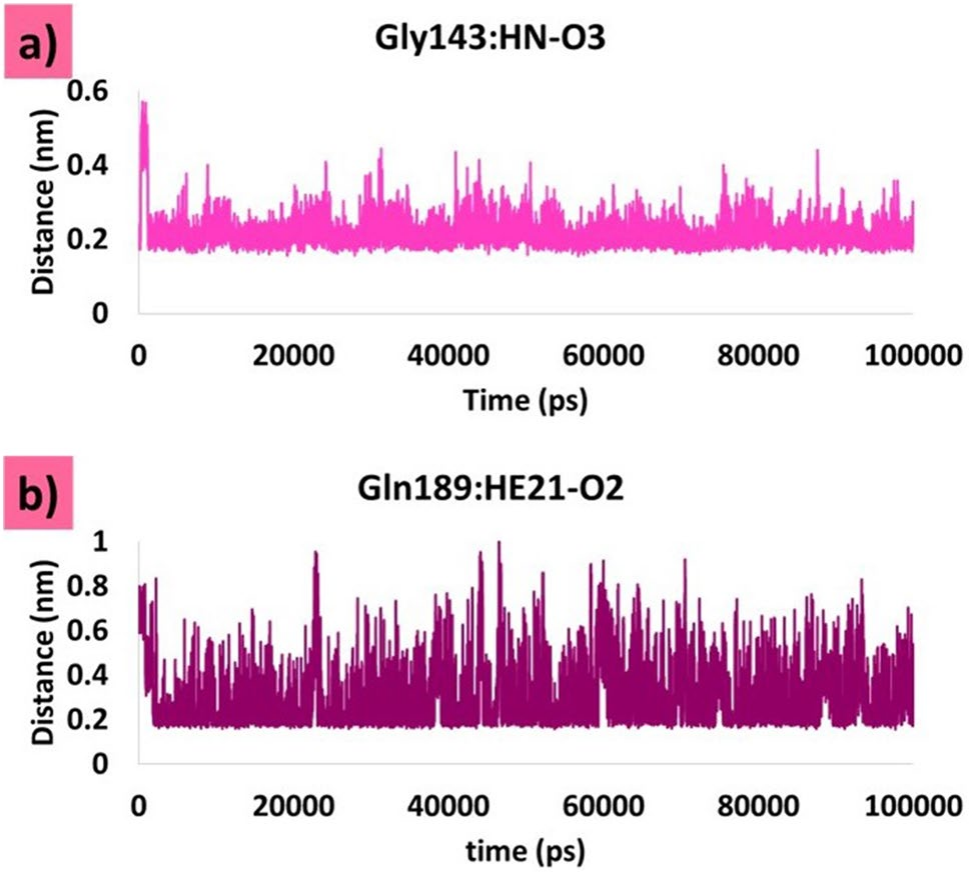
Hydrogen bond distances between the interacting protein atoms and the ligand atoms. (**a**) Distance between Gly143: HN-O3. (**b**) Distance between Gln189:HE21-O2.

The modified compound olaparib 1826, formed hydrogen bonds with Gly143 and Gln189 (Fig. 9a). Interestingly, oxygen atoms were involved in these interactions. The distances between the atoms involved in hydrogen bonding were calculated. The Gly143: HN-O3 interaction was highly stable, with an average of 0.21 nm throughout the simulation run (Fig. 6a). Similarly, the interaction between Gln189:HE21-O2 was also stable with an average of 0.28 nm, which indicates that the ligand tightly adheres to the binding pocket of the target (Fig. 6b). The key residue, His164, formed carbon hydrogen bonds to hold the ligand in the binding pocket. The residue Cys145 formed the π-alkyl interaction with the modified compound. The residues His41, His164, and Met165 adhered to the compound via alkyl interactions. His164 interacted with the fluorine atom of the ligand, thereby settling the ligand in the binding pocket of the target. Other residues, such as Met49, Phe140, Leu141, Asn142, Ser144, His163, Glu166, His172, Asp187, and Arg188 (Table 1) firmly held the compound in the binding pocket via van der Waals interactions (Fig. 9d).

#### Olaparib 1885

**Figure 7:**
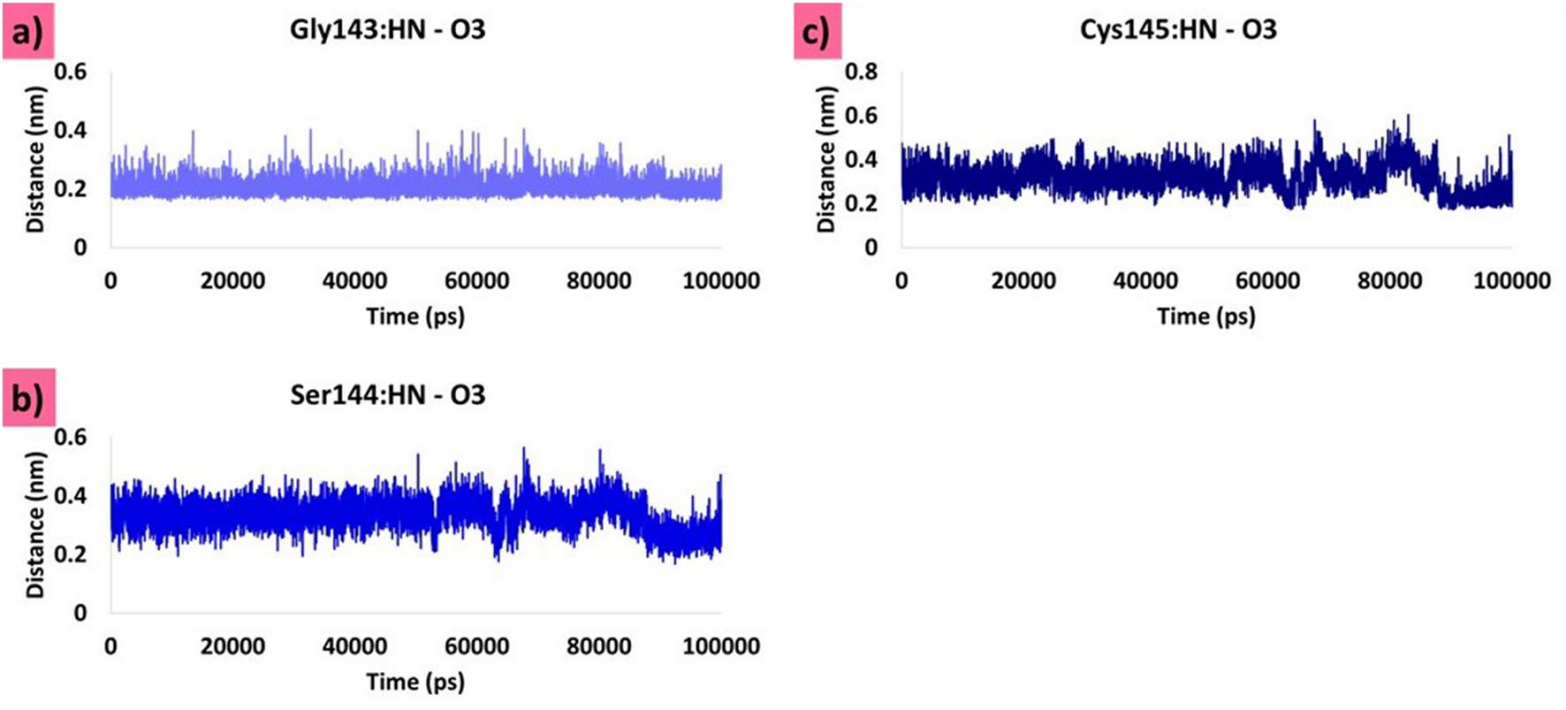
Hydrogen bond distance between the interacting protein atoms and the ligand atoms. (**a**) Distance between Gly143: HN-O3. (**b**) Distance between Ser144: HN-O3. (**c**) Distance between Cys145: HN-O3.

Olaparib 1885 formed hydrogen bonds with the Gly143, Ser144, and Cys145 residues (Fig. 9b). The hydrogen bond distance between Gly143: HN-O3 was stable, with no major aberrations. The average distance measured was found to be 0.2 nm (Fig. 7a). The hydrogen bond distance between Ser144: HN-O3 was analysed during the evolution of MDS (Fig. 7b). These findings reveal that the distance was largely stable, with a dip in the profile at 89,900 ps, which remained stable thereafter. Interestingly, the overall average distance was measured at 0.33 nm, while the last 10 ns were measured at 0.26 nm, suggesting that Ser144 strongly interacted with the ligand (Fig. 7b). Hydrogen bond interactions between Cys145: HN-O3 were stable throughout the simulation. There was a minor depression in the distance plot at 85,000 ps, which remained stable thereafter. While the overall distance was measured to be 0.31 nm, the average measurement for the last 10 ns was 0.23 nm (Fig. 7c). Generally, these three interactions are firm and hold the ligand in the binding pocket. Residues Asn142, His163, and His164 formed carbon–hydrogen bonds that firmly held the ligand at the active site. The key residues His41 and Cys145 have generated alkyl and π-alkyl interactions, thereby positioning the ligand at the binding site. The residue Met164 has prompted an amide π-stacked interaction. Residues Met49, Phe140, Leu141, Glu166, His172, Asp187, Arg188, and Glu189 (Table 1) promoted van der Waals interactions, thereby aiding the ligand to be seated at the binding pocket. The Ser144 residue interacts with the fluorine atom of the ligand (Fig. 9e).

### Rucaparib184

**Figure 8:**
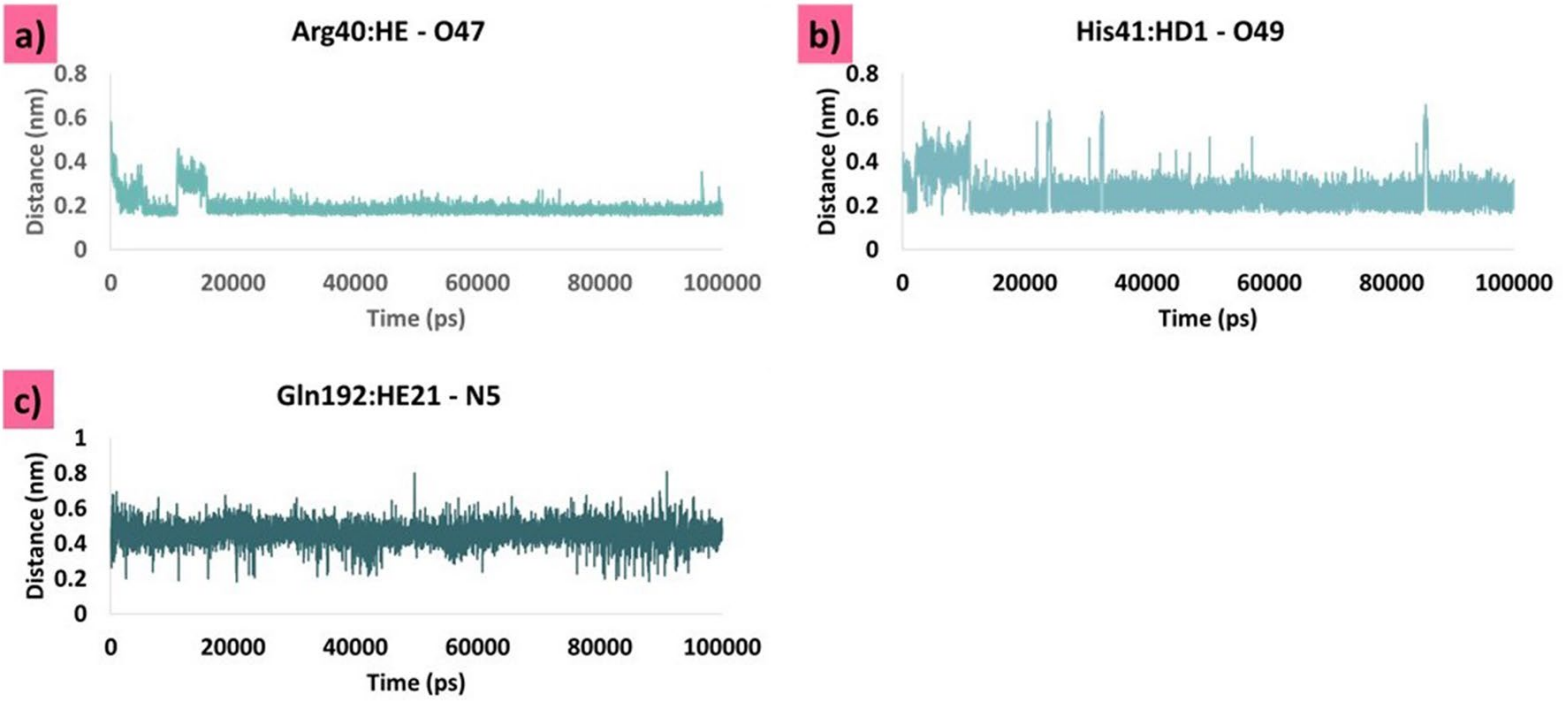
Hydrogen bond distances between the interacting protein atoms and the ligand atoms. (**a**) Distance between Arg40:HE-O47; (**b**) Distance between His41:HD1-O49; (**c**) Distance between Gln192:HE21-N5.

**Figure 9:**
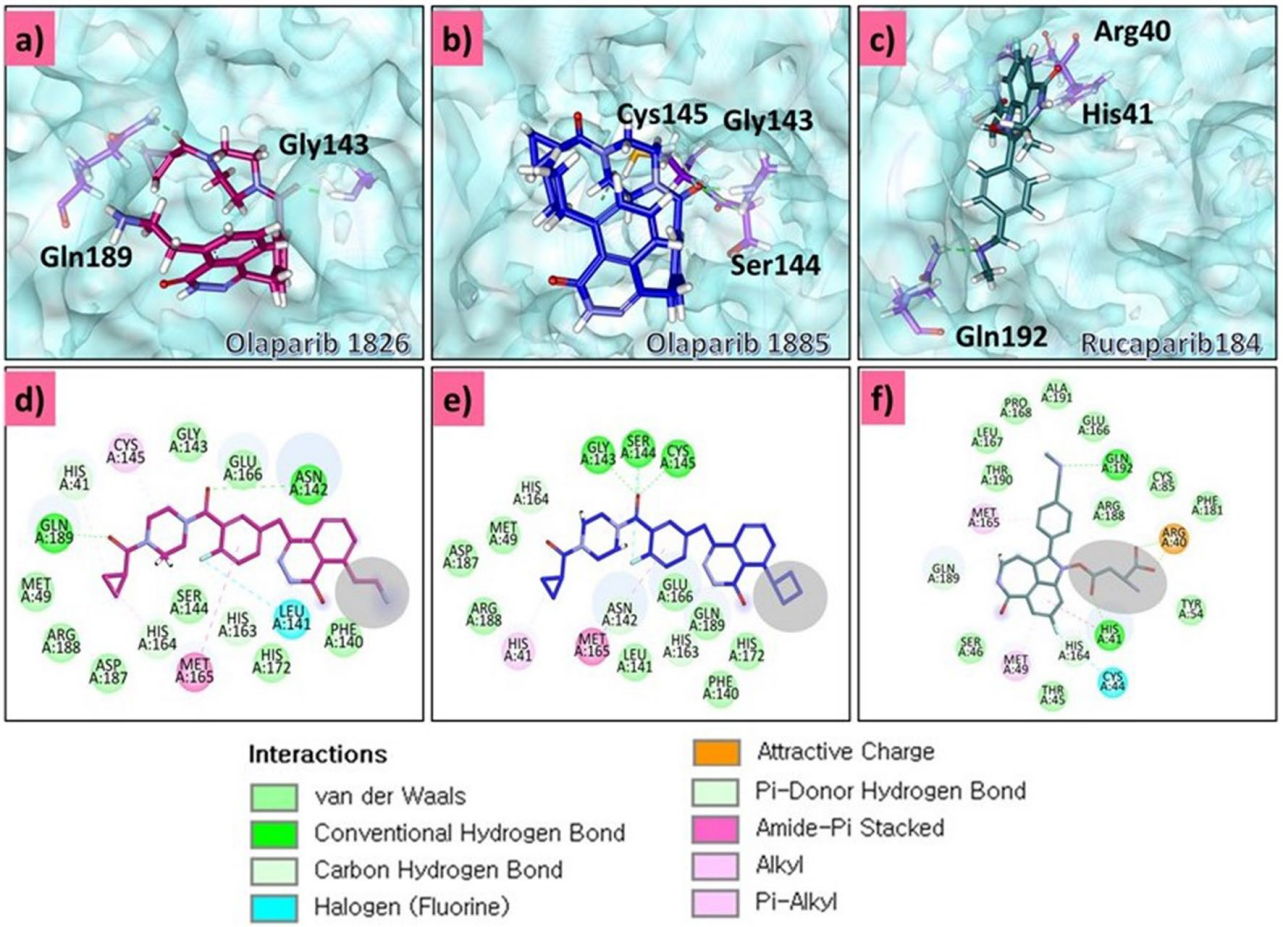
Comprehensive intermolecular interactions. (**a**–**c**) represent the hydrogen bond interactions between protein and ligand atoms of olaparib 1826, olaparib 1885, and rucaparib184. (**d**–**f**) represent the overall 2D interactions of olaparib1826, olaparib1885, and rucaparib 184 with the residues of the target.

Residues Arg40, His41, and Gln192 formed hydrogen bonds with rucaparib 184 (Fig. 9c). The hydrogen bond distances between the protein residue atoms and ligand atoms indicated that the interactions were stable during the simulation run. The Arg40: HE-O47 interaction has seen an elevation during the initial steps of the simulation run to 15,710 ps; however, it was soon stable thereafter with an overall average of 0.19 nm (Fig. 8a). Another interaction was observed between His41: HD1-O49. The distance profile displayed a stable interaction, although a few fluctuations were observed from 10 to 11 ns. The average distance was measured to be 0.25 nm during the evolution of the simulation (Fig. 8b). The hydrogen bond interaction distance between Gln192: HE21–N5 was also stable throughout the simulation. However, the average distance was projected to be 0.46 nm. This distance measured seems to be marginally weaker than the previous interactions that allowed the ligand to settle at the binding pocket (Fig. 8c). His164 and Glu189 residues adhered to the compound via carbon-hydrogen bonds. The key residues Met49, His41 and Met165 have prompted π-alkyl and π–π T shaped interactions with the ligand. The residue Cys44 formed an interaction with the fluorine atom, and Arg40 generated an attractive charge interaction, holding the ligand in the binding pocket of the compound. The residues Thr45, Ser46, Tyr54, Cys85, Glu166, Leu167, Pro168, Phe181, Thr190 and Ala191 (Table 1) have interacted with the ligand via the van der Waals interactions, thus positioning the ligand at the binding pocket (Fig. 9f). Analysis of the modified groups of the three compounds revealed that rucaparib 184 generated two hydrogen bonds with key residues.

### Interaction energies between protein and ligands

**Figure 10:**
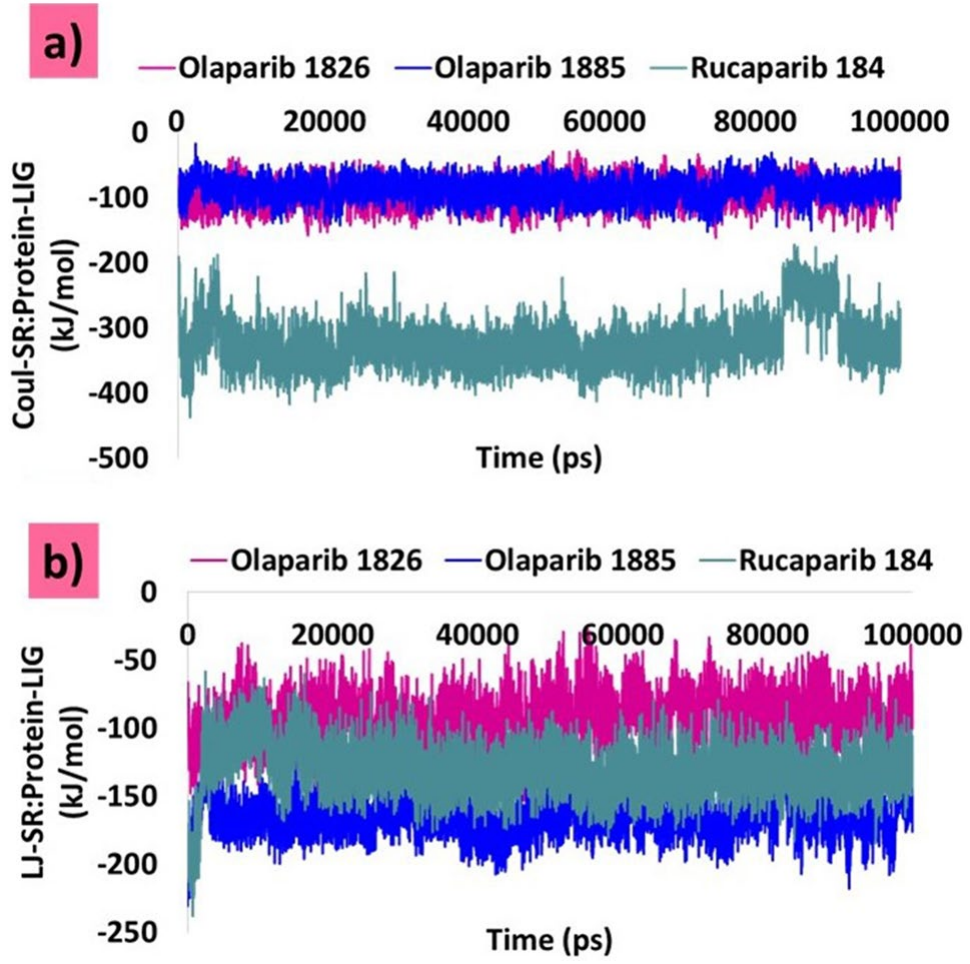
Interaction energy calculation. (**a**) Coul-SR: Protein-LIG. (**b**) LJ-SR: Protein-LIG.

Additionally, protein-ligand (Protein-LIG) interactions were confirmed by retrieving the short-range Coulomb (Coul-SR) and short-range Lennard-Jones (LJ-SR) energy terms. During the simulation, the complexes projected negative values. The average Coul-SR: Protein-LIG values for olaparib 1826, olaparib 1885, and rucaparib 184 were − 93.14 kJ/mol, 86.77 kJ/mol, and − 322.26 kJ/mol, respectively (Fig. 10a). The LJ-SR: Protein-LIG values for olaparib 1826, olaparib 1885 and rucaparib 184 are − 78.40 kJ/mol, − 165.11 kJ/mol, and − 131.55 kJ/mol, respectively (Fig. 10b). The average results of LJ-SR: Protein-LIG were similar for all ligands. However, the results of Coul-SR: Protein-LIG revealed that olaparib 1826 and olaparib 1885 demonstrated similar results, but rucaparib 184 projected a better result than both olaparib 1826 and olaparib 1885. These findings suggest that the modified compounds formed thermodynamically firm bonds with Mpro, as reported earlier^45^.

## Discussion

PARP proteins demonstrate structural similarity and function, with two riboses and two phosphates in a unit polymer^36^. PARP1 was discovered in 1963^36^. They perform critical functions including apoptosis, DNA damage response, and transcription modulation^46,47^. Their inhibitors exert anticancer activities^36,46,48^. In the current study, PARP inhibitors were used against COVID-19 target to specifically design putative inhibitors with affinity for SARS-CoV-2 Mpro.

To identify effective therapeutics for COVID-19, PARP inhibitors were employed as starting structures. Here, we computationally designed compounds that demonstrate higher affinity for Mpro than their parent structures.

To design new molecules computationally, we first identified a target with a large active site volume. We then marked the unoccupied spaces within the binding pocket after molecular docking. Correspondingly, Mpro was identified as the target and PARP inhibitors were docked to it. Olaparib and rucaparib had higher docking scores for Mpro. Therefore, these compounds were used in subsequent experiments.

**Figure 11:**
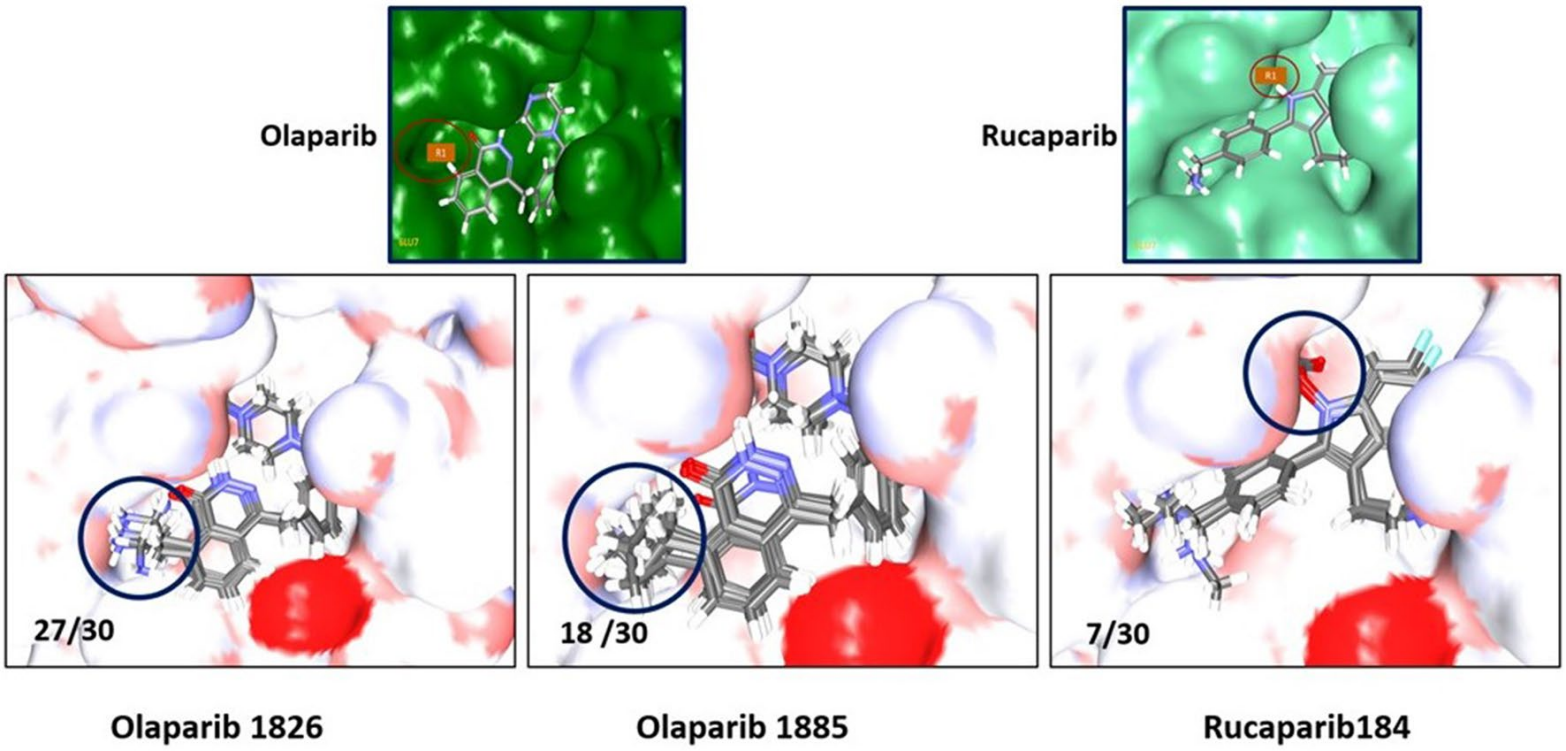
Clustering of the modified compounds post docking. The modified group occupies the same designated position that is marked.

The Mpro-ligand complex was selected to design new compounds by identifying the empty space located in the binding pocket (unoccupied by the ligand). Accordingly, with olaparib, two sites were identified (site 1 and site 2) and one site was identified with rucaparib (site 1) (Fig. 11, Supplementary Fig. 2). These sites were marked *R*_1_ and the *Grow Scaffold* module was initiated.

Molecular docking was initiated, followed by clustering analysis, to evaluate whether the new compounds induced conformational changes. The results revealed that the modified groups occupied positions at the desired sites, which aligned with the hypothesis. Furthermore, the binding affinity results showed that two compounds (olaparib 1826 and olaparib 1885) from olaparib site1 showed stronger affinity than site2 and occupied a defined position (Fig. 11), as did one compound from site3 (rucaparib 184). The olaparib-modified compounds showed better results against Mpro than the rucaparib-modified compounds. However, the modified compounds generated higher docking scores than the parent compounds.

Olaparib 1826 was obtained from the fragment library of organosilanes, reaction name yama coupling, and fragment name 2-(triethoxysilyl)ethylamine. Olaparib 1885 was obtained from the fragment library GrignardReagents, the reaction name Kumada Coupling, and the fragment name cyclobutylmagnesium chloride. Rucaparib 184 was synthesized using the fragment library acids with the fragment name monosodium L-aspartate dehydrate #2 and a reaction called esterification.

**Figure 12:**
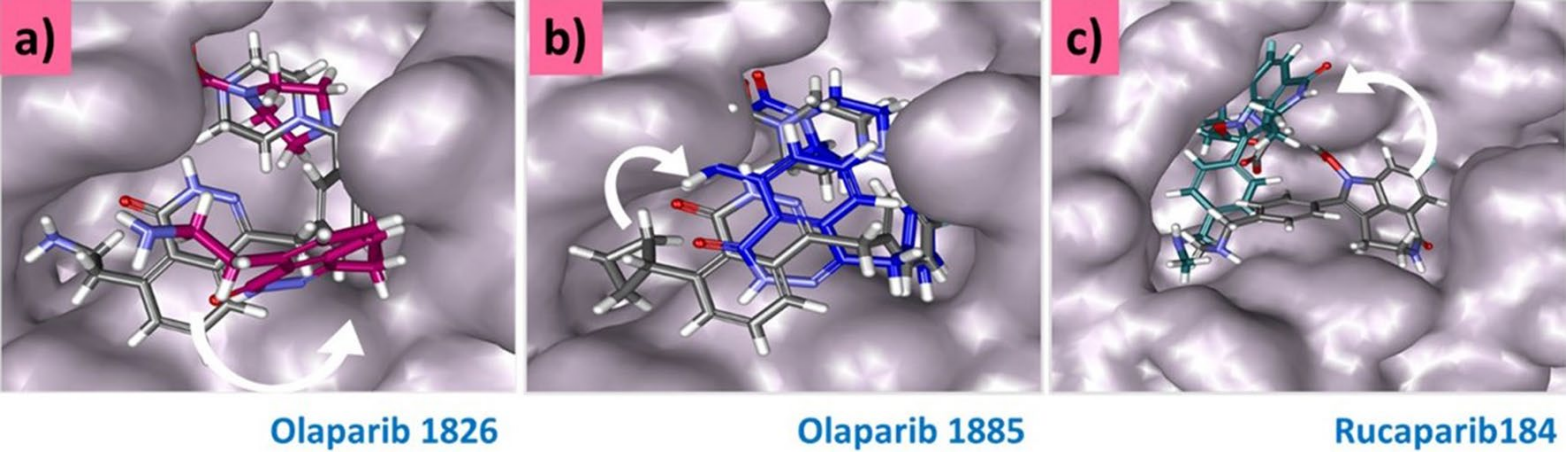
Binding mode examination for the initial pose and MDS pose. The molecular docked pose is represented in grey and the final MDS pose is represented in colour code.

In the MDS studies, olaparib 1826 and olaparib 1885 maintained a similar binding mode to that seen in molecular docking, while rucaparib 184 changed dramatically from its initial position. However, all the compounds were accommodated at the active site of the target while adhering to key residue interactions (Fig. 12).

Molecular docking interactions revealed that with Mpro, olaparib 1826 had hydrogen bond interactions with the key residues Gly143, Ser144, Gln189, and Thr190. MDS retained hydrogen bonds with Gly143 and Gln189. Interactions with Gly143 have been reported previously^28,30,49^. Similarly, a hydrogen bond was also observed with Gln189 in the molecular docking and MDS pose, as reported previously^50–52^. Hydrogen bonds between these residues were also observed in the X-ray structures. In addition, several other interactions originating from the binding pocket of the target firmly hold the ligand at its active site. The distance measured for these residues was also within the acceptable length below 0.3 nm during the progression of MDS (Fig. 6**)**. Moreover, targeting the catalytic dyad residues His41 and Cys145 is important for the developing of Mpro inhibitors^53^. Our results show that olaparib 1826 formed van der Waals interactions with His41, and alkyl interactions with Cys145. No change in the binding mode was observed in the molecular docking or MDS poses (Fig. 12a).

The compound olaparib 1885 formed hydrogen bonds with Gly143, Ser144, and Cys145. Similar bonds have been observed in a previous study^54–56^. The residue Glu189 that formed a hydrogen bond with the docked pose, also formed van der Waals interactions after MDS. However, the remaining two hydrogen bonds with Gly143 and Ser144 were retained. Hydrogen bond interactions with Gly143 and Ser144 were also observed in the X-ray structure; and Cys145 formed van der Waals interactions. The binding modes of olaparib 1826 and olaparib 1885 did not significantly change (Fig. 12b).

Rucaparib 184 formed hydrogen bonds with Arg40, His41, and Gln192 after MDS which was different from the molecular docked pose. Among the three compounds, rucaparib underwent drastic changes from its initial structure and was buried deep in the active site (Figs. 11 and 12c). The interactions between Arg40 and His41 were stronger, with an acceptable distance measured below 0.3 nm. Various interactions with Gln192 have been reported earlier^57,58^. The key residue Met165 had formed π-alkyl interactions with olaparib 1885 and olaparib 1826 while with rucaparib it has formed an alkyl interaction as was seen in the X-ray structure. However, in the molecular docking pose, this residue generated a carbon-hydrogen bond (Supplementary Fig. 4). Several other key residues originating from different subsites^59,60^ also prompted van der Waals interactions. In particular, Glu166 promoted van der Waals interactions with all the ligands (Table 1).

We also calculated the binding energies for the three complexes along with the co-crystallised compound. This was done by initiating the ‘*Calculate Binding Energies’* tool available on the DS. This calculation allows for the assessment of the binding energy between the receptor and the ligand. The binding energy for the co-crystallised compound was estimated to be − 136.724 kcal/mol. The binding energy for olaparib 1826, olaparib 1885, and rucaparib 184 were calculated to be − 44.2554 kcal/mol, − 63.7637 kcal/mol, and − 86.8231 kcal/mol, respectively. The higher results for the co-crystallised ligand with respect to the molecular docking score and binding energy may be due to its larger size than the modified compounds.

The identified compounds also demonstrated an acceptable Lipinski’s rule of five which was calculated using the *Filter by Lipinski’s and Veber.* These compounds showed acceptable results (Table 2). Since the compounds were novel, we estimated their ability to be synthesised by adapting the SWISS ADME^61^ web tool and read it according to the synthetic accessibility score. The results ranged from 1(very easy) to 10 (very difficult)^61^. The synthetic accessibility score for olaparib 1826, olaparib 1885, and rucaparib 184 were 3.55, 3.63, and 4.30, respectively. Scores close to 5, indicated that these compounds may be easily synthesised (Table 2). These elegant findings show the compounds olaparib 1826, olaparib 1885, and rucaparib 184 are possible Mpro inhibitors.

**Table 2:** Synthetic accessibility score and drug-like properties of the modified compounds.

Compound Name	SMILES	Synthetic accessibility	Hydrogen bond Acceptor	Hydrogen bond Donor	MW	ALogP	Rotatable bonds	Polar surface area (Å2)
Olaparib 1826	NCCc1cccc2C(=NNC(=O)c12)Cc3ccc(F)c(c3)C(=O)N4CCN(CC4)C(=O)C5CC5	3.55	8	3	477.531	1.554	6	108.1
Olaparib 1885	Fc1ccc(CC2=NNC(=O)c3c(cccc23)C4CCC4)cc1C(=O)N5CCN(CC5)C(=O)C6CC6	3.63	7	1	488.553	3.406	5	82.08
Rucaparib184	CNCc1ccc(cc1)c2c3CCNC(=O)c4cc(F)cc(c34)n2OC(=O)C[C@H](N)C(=O)[O-]	4.30	9	4	453.443	− 2.051	8	138.51

**Figure 13:**
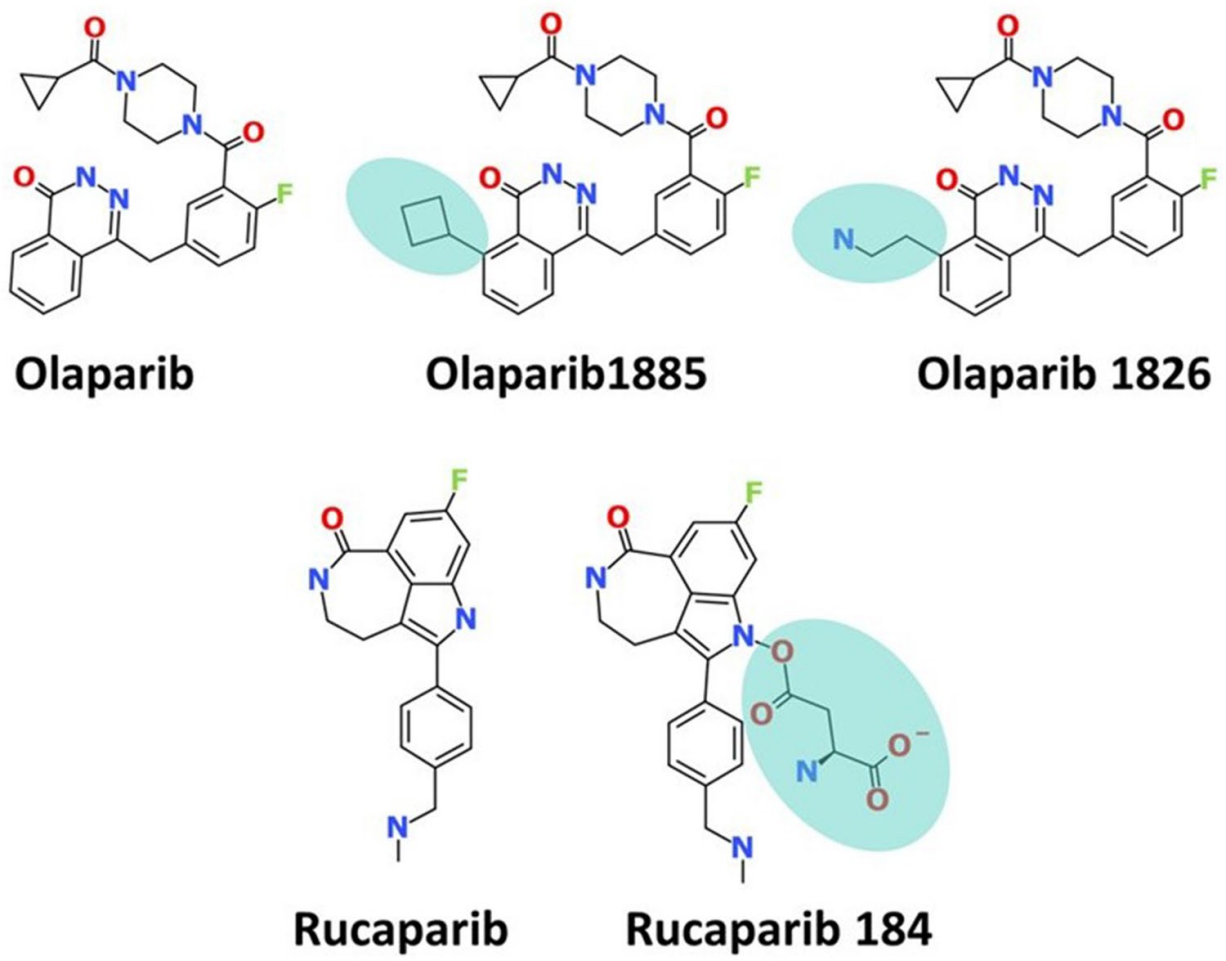
The 2D structures of the modified compounds in comparison with the parent structures.

The novelty of the compounds was verified using SMILES as an input to PubChem^62^. These results indicate that these compounds were not yet used. We speculated that these designed compounds (Fig. 13) have not yet been synthesised and could be new compounds for the treatment of COVID-19.

## Materials and methods

### Selection of the ligands

**Figure 14:**
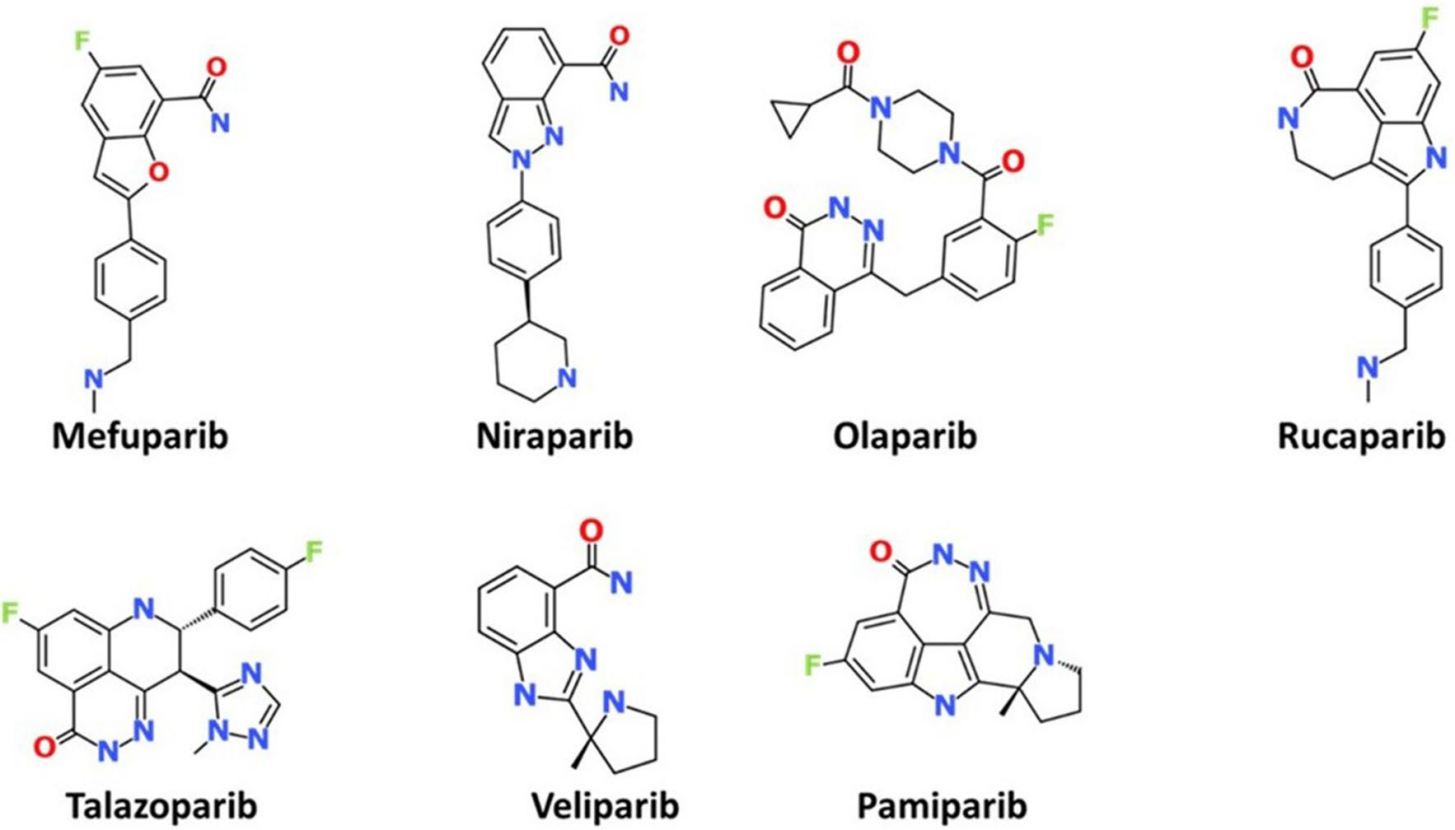
2D structures of the PARP inhibitors that are used to design new compounds.

PARP inhibitors were sketched using Biovia Draw v2017 and saved as a molfile (. Mol) format **(**Fig. 14**)** and were exported to the DS. The ligands were minimised using the “minimize ligands” protocol available with the DS. The CHARMM force field, was adapted using a *Smart Minimizer* algorithm. This was executed using 1000 steps of steepest descent with a RMS gradient tolerance of 3; subsequently, conjugate gradient minimisation was applied.

### Selection of the targets

The target selected for this study was Mpro (PDB ID:6LU7) from SARS-CoV-2^26^. The protein was prepared by enabling the ‘*Prepare Protein’* protocol available with the DS. This protocol prepares and checks the given protein and performs actions that include modelling the missing loop regions, standardising the atom names, dislodging the water molecules, inserting missing atoms in the incomplete residues^63,64^, deleting the alternate conformations, and protonating titratable residues using the predicted pKs. The active site for molecular docking was selected around the co-crystallised ligand for all atoms and residues at 13.82 Ǻ. This was done by enabling the tool ‘*Define and Edit Binding Site’* that correspondingly creates a binding sphere. It has been reported that the active site contains subsites of origin of different residues^65^.

Prior to the initiation of molecular docking, the co-crystallised ligand N3 was dislodged and redocked into the selected binding pocket to ensure that the binding mode was reproduced. The results have shown that the ligand generated a similar binding mode as that of the co-crystallised ligand with an acceptable RMSD of 0.9 Ǻ (Supplementary Fig. 5).

### Binding affinity studies

Binding affinity studies were performed between Mpro and small molecules using the CDOCKER program available in the DS. Prior to the initiation of docking studies, the binding pockets of the targets were examined to determine the active sites. We aimed to identify a target with a large active site to facilitate the designing the SARS-CoV-2 specific inhibitors (Supplementary Fig. 1).

Accordingly, Mpro was selected, and PARP inhibitors were docked into the active site. Correspondingly, the active site was chosen to be around the co-crystallised inhibitor N3. The selected ligands were used to generate 30 conformations. The best pose was selected after clustering the conformers to determine the best binding mode. From the largest cluster, the compound with the best docking score (binding affinity) revealing interactions with the key residues, was chosen.

### Strategy to design the new compounds

To design new compounds computationally, we examined the target ligand complex, after molecular docking, to locate the empty spaces. Subsequently, certain *points* are identified and marked as *R*_1._ The “*Grow Scaffold*” module available with the DS was enabled which resulted in a total of 3942 compounds. These compounds were docked into Mpro to estimate their binding affinities after a drug-like assessment.

The ‘*Grow Scaffold’* module permits the user to accomplish reaction-based ligand listing inside the protein’s active site by performing lead optimisation. It begins with the positioning of the scaffold at the receptor-binding site. The user can select a position that can act as a reaction vector, followed by the reactions and reagents to be used. Furthermore, the ligand “novelty” is computed by sorting and ranking the ligands by number of chain assemblies, number of double and aromatic bonds; and N, S, O atom count.

### Molecular dynamics simulation (MDS) analysis

The protein-LIG complex structures obtained from molecular docking were escalated to MDS to understand their binding potency at the binding pocket of the targets. GROningen MAchine for Chemical Simulations (GROMACS) v2016.6 was used to study the MDS^66,67^. A CHARMM27 all-atom force field was utilized^68^. The topologies of the ligands were acquired from SwissParam^69^ and the *topol.top* file was updated accordingly. Subsequently, the systems were solvated in a dodecahedral water box using the TIP3P water model. The system was neutralised using counterions. Energy minimisation was performed in 50,000 steps to remove bad contacts and clashes. After successful energy minimisation, the protein and ligand were coupled using the *gmx make_ndx* command to progress through the two-step equilibration process. The first step of equilibration was (constant number of particles, volume, and temperature) NVT equilibration, which was performed for 100 ps using a V-rescale thermostat at 300 K. The second equilibration was conducted with a constant number of particles, pressure, and temperature (NPT) for 100 ps using a Parrinello-Rahman barostat to monitor the pressure at 1 bar. During equilibration, the protein backbone was restrained and the non-protein was permitted to wobble. The long-range electrostatic interactions were evaluated by the Particle Mesh Ewald (PME) method and the short-range interactions and interactions by van der Waals were calculated after applying upper limit of 9 Å and 14 Å, correspondingly. MDS progressed under periodic boundary conditions for 100 ns. The corresponding analysis was conducted using various GROMACS tools and visual molecular dynamics (VMD)^42,70^.

### Analysis of the trajectory

The different tools available to analyse the MDS results, and GROMACS were utilized^71^. RMSD was assessed using the *gmx rms*. These calculations provide knowledge of the deviation present, if any, in the protein from the initial to final conformation during the MDS run. Logically, the lower the RMSD, the greater the stability of the protein^72^. Rg, which determines the compactness of the protein, was studied using *gmx gyrate*^73^. The RMSF of the protein residues was determined using the *gmx rmsf* tool. Here, the fluctuations in each residue of the protein were examined. The RMSD, Rg and RMSF were computed for the protein backbone. Using the *gmx hbond*, the number of hydrogen bond interactions between protein and ligand atoms were evaluated during MDS progression. Furthermore, the interaction energy was calculated using the *gmx energy*, which computes both the short-ranged Coulombic and Lennard–Jones energy interactions^45^.

## Conclusion

PARP inhibitors are well-known for their anticancer properties. Recently, in vitro studies also demonstrated their anti-COVID-19 properties. In the present study, we aimed to design new PARP inhibitors with high affinity for the COVID-19 Mpro. Correspondingly, two compounds originating from olaparib and one compound originating from rucaparib displayed high binding affinities for Mpro. This indicates their potential use as SARS-CoV-2 inhibitors. These compounds also demonstrated favourable synthetic accessibility scores. Additionally, our method could aid the scientific community to design new compounds and discover new horizons for drug discovery against SARS-CoV-2.

## Supplementary Information

Supplementary Figures.

Supplementary Table 1.

Supplementary Table 2.

## Data Availability

The generated dataset resulted from the ‘Grow Scaffold’ are provided as the Supplementary Table 2. A SMILES representation of the compounds is provided.

## References

[1] Major C (2020). Unprecedented times and innovation. Innov. High. Educ..

[2] Hitchings E, Maclean M (2020). Unprecedented times: some thoughts on the consequences of the COVID-19 pandemic from a family and social welfare law perspective. J. Soc. Welf. Fam. Law.

[3] Li H, Liu SM, Yu XH, Tang SL, Tang CK (2020). Coronavirus disease 2019 (COVID-19): Current status and future perspectives. Int. J. Antimicrob. Agents.

[4] Pushpakom S (2018). Drug repurposing: Progress, challenges and recommendations. Nat. Rev. Drug Discov..

[5] Rudrapal M, Khairnar SJ (2020). Drug repurposing (DR): An emerging approach in drug discovery. Drug Repurposing - Hypothesis, Molecular Aspects and Therapeutic Applications.

[6] Dae JW, Sangeun J, Seungtaek K, Yup LS (2021). Drugs repurposed for COVID-19 by virtual screening of 6218 drugs and cell-based assay. Proc. Natl. Acad. Sci..

[7] Guy RK, DiPaola RS, Romanelli F, Dutch RE (2020). Rapid repurposing of drugs for COVID-19. Science.

[8] Gupta RK, Nwachuku EL, Zusman BE, Jha RM, Puccio AM (2021). Drug repurposing for COVID-19 based on an integrative meta-analysis of SARS-CoV-2 induced gene signature in human airway epithelium. PLoS ONE.

[9] Bakowski MA (2021). Drug repurposing screens identify chemical entities for the development of COVID-19 interventions. Nat. Commun..

[10] Omer SE (2022). Drug repurposing for SARS-CoV-2 main protease: Molecular docking and molecular dynamics investigations. Biochem. Biophys. Rep..

[11] Rampogu S, Lee KW (2021). Pharmacophore modelling-based drug repurposing approaches for SARS-CoV-2 therapeutics. Front. Chem..

[12] Rampogu S, Gajula RG, Lee G, Kim MO, Lee KW (2021). Unravelling the therapeutic potential of marine drugs as SARS-CoV-2 inhibitors: An insight from essential dynamics and free energy landscape. Comput. Biol. Med..

[13] Singh R, Bhardwaj VK, Sharma J, Purohit R, Kumar S (2022). In-silico evaluation of bioactive compounds from tea as potential SARS-CoV-2 nonstructural protein 16 inhibitors. J. Tradit. Complement. Med..

[14] Aljindan RY (2021). Investigation of nonsynonymous mutations in the spike protein of SARS-CoV-2 and its interaction with the ACE2 receptor by molecular docking and MM/GBSA approach. Comput. Biol. Med..

[15] Borgio JF (2020). State-of-the-art tools unveil potent drug targets amongst clinically approved drugs to inhibit helicase in SARS-CoV-2. Arch. Med. Sci..

[16] Singh R, Bhardwaj VK, Purohit R (2022). Inhibition of nonstructural protein 15 of SARS-CoV-2 by golden spice: A computational insight. Cell Biochem. Funct..

[17] Fadlalla M, Ahmed M, Ali M, Elshiekh AA, Yousef BA (2022). Molecular docking as a potential approach in repurposing drugs against COVID-19: A systematic review and novel pharmacophore models. Curr. Pharmacol. Reports.

[18] Lazniewski M (2022). Drug repurposing for identification of potential spike inhibitors for SARS-CoV-2 using molecular docking and molecular dynamics simulations. Methods.

[19] Al-Karmalawy AA (2021). Molecular docking and dynamics simulation revealed the potential inhibitory activity of aceis against SARS-CoV-2 targeting the hACE2 receptor. Front. Chem..

[20] Eweas AF, Alhossary AA, Abdel-Moneim AS (2021). Molecular docking reveals ivermectin and remdesivir as potential repurposed drugs against SARS-CoV-2. Front. Microbiol..

[21] Rampogu S, Lee KW (2021). Old drugs for new purpose—fast pace therapeutic identification for SARS-CoV-2 infections by pharmacophore guided drug repositioning approach. Bull. Korean Chem. Soc..

[22] Hosseini M, Chen W, Xiao D, Wang C (2021). Computational molecular docking and virtual screening revealed promising SARS-CoV-2 drugs. Precis. Clin. Med..

[23] Mohammed AO, Abo-Idrees MI, Makki AA, Ibraheem W, Alzain AA (2022). Drug repurposing against main protease and RNA-dependent RNA polymerase of SARS-CoV-2 using molecular docking, MM-GBSA calculations and molecular dynamics. Struct. Chem..

[24] Matondo A (2022). In silico drug repurposing of anticancer drug 5-FU and analogues against SARS-CoV-2 main protease: Molecular docking, molecular dynamics simulation, pharmacokinetics and chemical reactivity studies. Adv. Appl. Bioinform. Chem. AABC.

[25] Capoluongo E (2020). PARP-inhibitors in a non-oncological indication as COVID-19: Are we aware about its potential role as anti-thrombotic drugs?. Discuss. Open. Biomed. Pharmacother..

[26] Jin Z (2020). Structure of Mpro from SARS-CoV-2 and discovery of its inhibitors. Nature.

[27] Ullrich S, Nitsche C (2020). The SARS-CoV-2 main protease as drug target. Bioorg. Med. Chem. Lett..

[28] Zhang L (2020). Crystal structure of SARS-CoV-2 main protease provides a basis for design of improved a-ketoamide inhibitors. Science.

[29] Hu Q (2022). The SARS-CoV-2 main protease (Mpro): Structure, function, and emerging therapies for COVID-19. MedComm.

[30] Mengist HM, Dilnessa T, Jin T (2021). Structural basis of potential inhibitors targeting SARS-CoV-2 main protease. Front. Chem..

[31] Katre SG (2022). Review on development of potential inhibitors of SARS-CoV-2 main protease (MPro). Future J. Pharm. Sci..

[32] Citarella A, Scala A, Piperno A, Micale N (2021). SARS-CoV-2 M(pro): A potential target for peptidomimetics and small-molecule inhibitors. Biomolecules.

[33] Kanhed AM (2020). Identification of potential Mpro inhibitors for the treatment of COVID-19 by using systematic virtual screening approach. Mol. Divers..

[34] Cui W, Yang K, Yang H (2020). recent progress in the drug development targeting SARS-CoV-2 main protease as treatment for COVID-19. Front. Mol. Biosci..

[35] Wang Y (2021). PARP inhibitors in gastric cancer: Beacon of hope. J. Exp. Clin. Cancer Res..

[36] Chen A (2011). PARP inhibitors: its role in treatment of cancer. Chin. J. Cancer.

[37] Du Y, Yamaguchi H, Hsu JL, Hung M-C (2017). PARP inhibitors as precision medicine for cancer treatment. Natl. Sci. Rev..

[38] Curtin N (2020). Repositioning PARP inhibitors for SARS-CoV-2 infection(COVID-19); A new multi-pronged therapy for acute respiratory distress syndrome?. Br. J. Pharmacol..

[39] Ge Y (2021). An integrative drug repositioning framework discovered a potential therapeutic agent targeting COVID-19. Signal Transduct. Target. Ther..

[40] Stone NE (2021). Stenoparib, an inhibitor of cellular Poly(ADP-Ribose) polymerase, blocks replication of the SARS-CoV-2 and HCoV-NL63 human coronaviruses in vitro. MBio.

[41] Badawy AA-B (2020). Immunotherapy of COVID-19 with poly (ADP-ribose) polymerase inhibitors: Starting with nicotinamide. Biosci. Rep..

[42] Rampogu S (2023). Explicit molecular dynamics simulation studies to discover novel natural compound analogues as *Mycobacterium tuberculosis* inhibitors. Heliyon.

[43] Galzitskaya OV, Bogatyreva NS, Ivankov DN (2008). Compactness determines protein folding type. J. Bioinform. Comput. Biol..

[44] Muhammad S (2022). Exploring the inhibitory potential of novel bioactive compounds from mangrove actinomycetes against nsp10 the major activator of SARS-CoV-2 replication. Chem. Pap..

[45] Bepari A, Reza H (2021). Identification of a novel inhibitor of SARS-CoV-2 3CL-PRO through virtual screening and molecular dynamics simulation. PeerJ.

[46] Rose M, Burgess JT, O’Byrne K, Richard DJ, Bolderson E (2020). PARP inhibitors: clinical relevance, mechanisms of action and tumor resistance. Front. Cell Dev. Biol..

[47] Morales J (2014). Review of poly (ADP-ribose) polymerase (PARP) mechanisms of action and rationale for targeting in cancer and other diseases. Crit. Rev. Eukaryot. Gene Expr..

[48] Chan CY, Tan KV, Cornelissen B (2021). PARP inhibitors in cancer diagnosis and therapy PARP imaging and therapy. Clin. Cancer Res..

[49] Huff S (2022). Discovery and mechanism of SARS-CoV-2 main protease inhibitors. J. Med. Chem..

[50] Hattori S (2021). A small molecule compound with an indole moiety inhibits the main protease of SARS-CoV-2 and blocks virus replication. Nat. Commun..

[51] Mahmud S (2022). Plant-derived compounds effectively inhibit the main protease of SARS-CoV-2: An in silico approach. PLoS ONE.

[52] Bahun M (2022). Inhibition of the SARS-CoV-2 3CLpro main protease by plant polyphenols. Food Chem..

[53] Ferreira JC, Fadl S, Villanueva AJ, Rabeh WM (2021). Catalytic dyad residues His41 and Cys145 impact the catalytic activity and overall conformational fold of the main SARS-CoV-2 protease 3-chymotrypsin-like protease. Front. Chem..

[54] Sacco MD (2020). Structure and inhibition of the SARS-CoV-2 main protease reveal strategy for developing dual inhibitors against Mpro and cathepsin L. Sci. Adv..

[55] Su H (2021). Identification of pyrogallol as a warhead in design of covalent inhibitors for the SARS-CoV-2 3CL protease. Nat. Commun..

[56] Singh R (2022). Benchmarking the ability of novel compounds to inhibit SARS-CoV-2 main protease using steered molecular dynamics simulations. Comput. Biol. Med..

[57] Suárez D, Díaz N (2020). SARS-CoV-2 main protease: A molecular dynamics study. J. Chem. Inf. Model..

[58] Verma S, Patel CN, Chandra M (2021). Identification of novel inhibitors of SARS-CoV-2 main protease (M(pro) ) from *Withania* sp. by molecular docking and molecular dynamics simulation. J. Comput. Chem..

[59] Rampogu S, Lee KW (2021). Old drugs for new purpose—Fast pace therapeutic identification for SARS-CoV-2 infections by pharmacophore guided drug repositioning approach. Bull. Korean Chem. Soc..

[60] Rampogu S, Gajula RG, Lee G, Kim MO, Lee KW (2021). Unravelling the therapeutic potential of marine drugs as SARS-CoV-2 inhibitors: An insight from essential dynamics and free energy landscape. Comput. Biol. Med..

[61] Daina A, Michielin O, Zoete V (2017). SwissADME: A free web tool to evaluate pharmacokinetics, drug-likeness and medicinal chemistry friendliness of small molecules. Sci. Rep..

[62] Kim S (2016). PubChem substance and compound databases. Nucleic Acids Res..

[63] Spassov VZ, Flook PK, Yan L (2008). LOOPER: A molecular mechanics-based algorithm for protein loop prediction. Protein Eng. Des. Sel..

[64] Spassov VZ, Yan L (2008). A fast and accurate computational approach to protein ionization. Protein Sci..

[65] Kneller DW (2020). Structural plasticity of SARS-CoV-2 3CL Mpro active site cavity revealed by room temperature X-ray crystallography. Nat. Commun..

[66] Van Der Spoel D (2005). GROMACS: Fast, flexible, and free. J. Comput. Chem..

[67] Lemkul J (2018). From proteins to perturbed hamiltonians: A suite of tutorials for the GROMACS-2018 molecular simulation package [Article v1.0]. Living J. Comput. Mol. Sci..

[68] Bjelkmar P, Larsson P, Cuendet MA, Hess B, Lindahl E (2010). Implementation of the CHARMM force field in GROMACS: Analysis of protein stability effects from correction maps, virtual interaction sites, and water models. J. Chem. Theory Comput..

[69] Zoete V, Cuendet MA, Grosdidier A, Michielin O (2011). SwissParam: A fast force field generation tool for smallorganic molecules. J. Comput. Chem..

[70] Humphrey W, Dalke A, Schulten KVMD (1996). Visual molecular dynamics. J. Mol. Graph..

[71] Kushwaha PP (2021). Identification of natural inhibitors against SARS-CoV-2 drugable targets using molecular docking, molecular dynamics simulation, and MM-PBSA approach. Front. Cell. Infect. Microbiol..

[72] Sharma J (2021). An in-silico evaluation of different bioactive molecules of tea for their inhibition potency against non structural protein-15 of SARS-CoV-2. Food Chem..

[73] Lobanov MY, Bogatyreva NS, Galzitskaya OV (2008). Radius of gyration as an indicator of protein structure compactness. Mol. Biol..

