# Supplementary material for: Repurposing and computational design of PARP inhibitors as SARS-CoV-2 inhibitors
Source: Sci Rep. 2023 Jun 29;13:10583. doi: 10.1038/s41598-023-36342-7 (PMC10310815; doi:10.1038/s41598-023-36342-7)
Supplement: Supplementary file 1 — Supplementary Figures. [file 41598_2023_36342_MOESM1_ESM.docx]

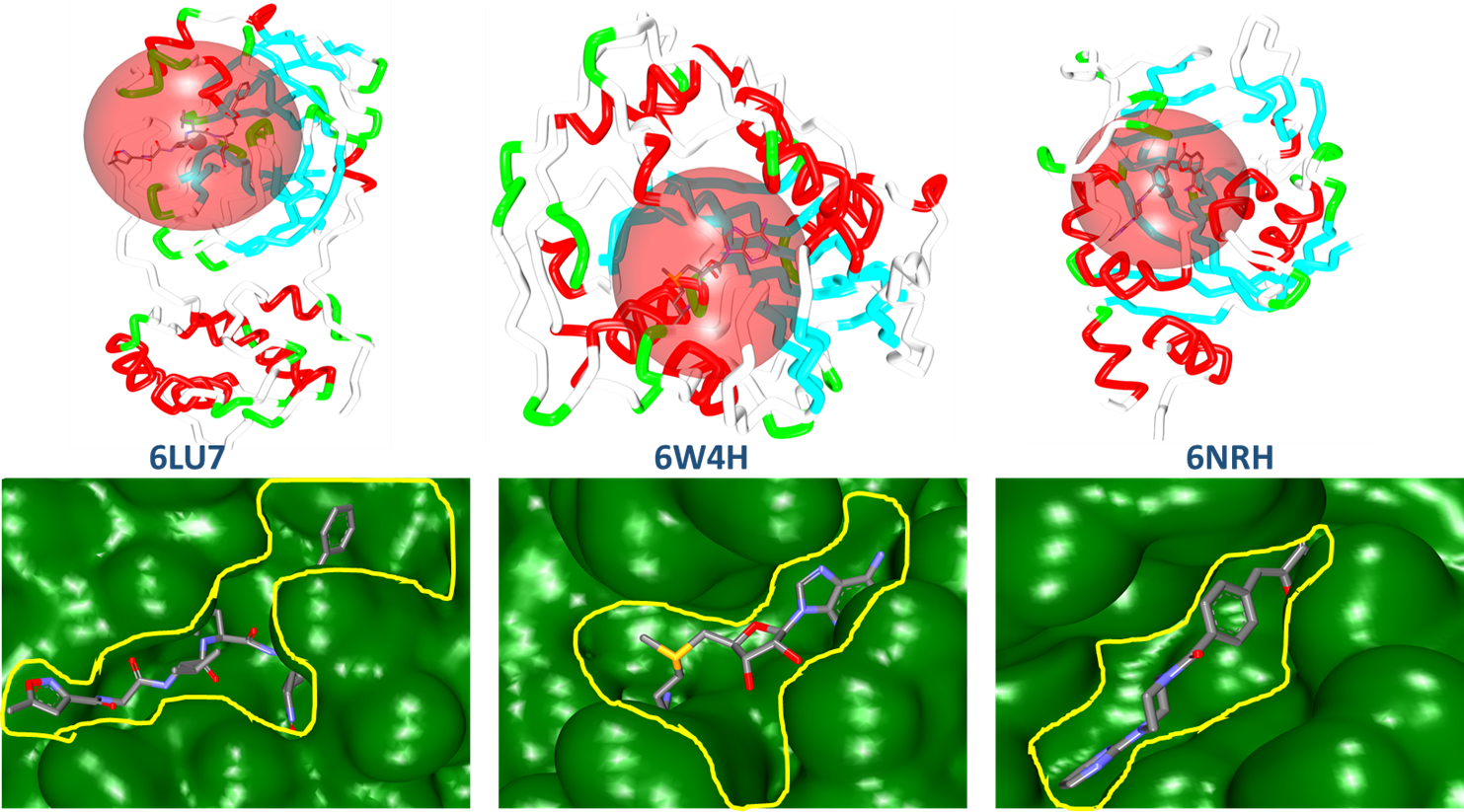


Supplementary Figure 1. Binding pocket of Mpro indicated in red sphere and in yellow


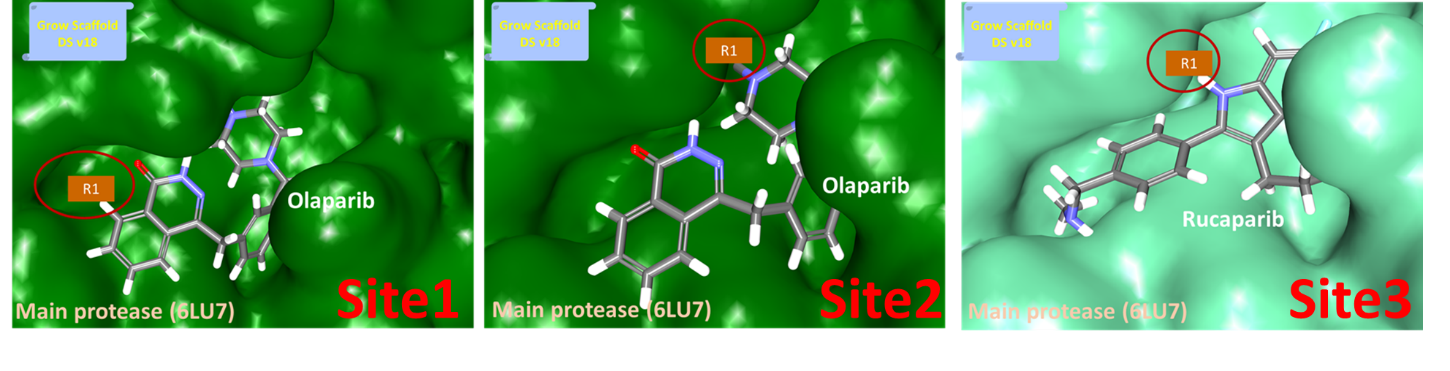


Supplementary Figure 2. Identify the three sites for growing the scaffold.


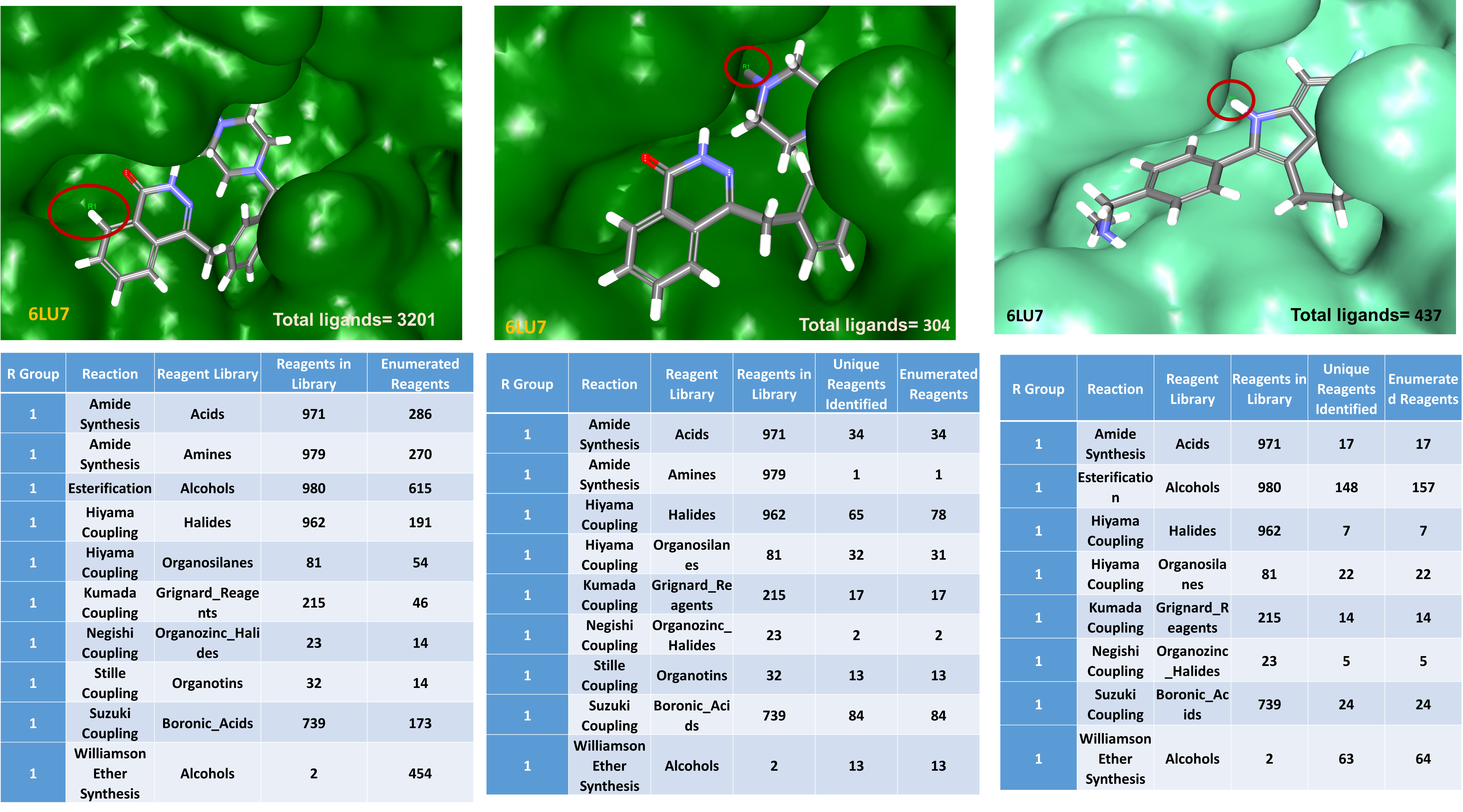


Supplementary Figure 3. Various reagent libraries used during the grow scaffold approach. This resulted in a total of 3,942 compounds.


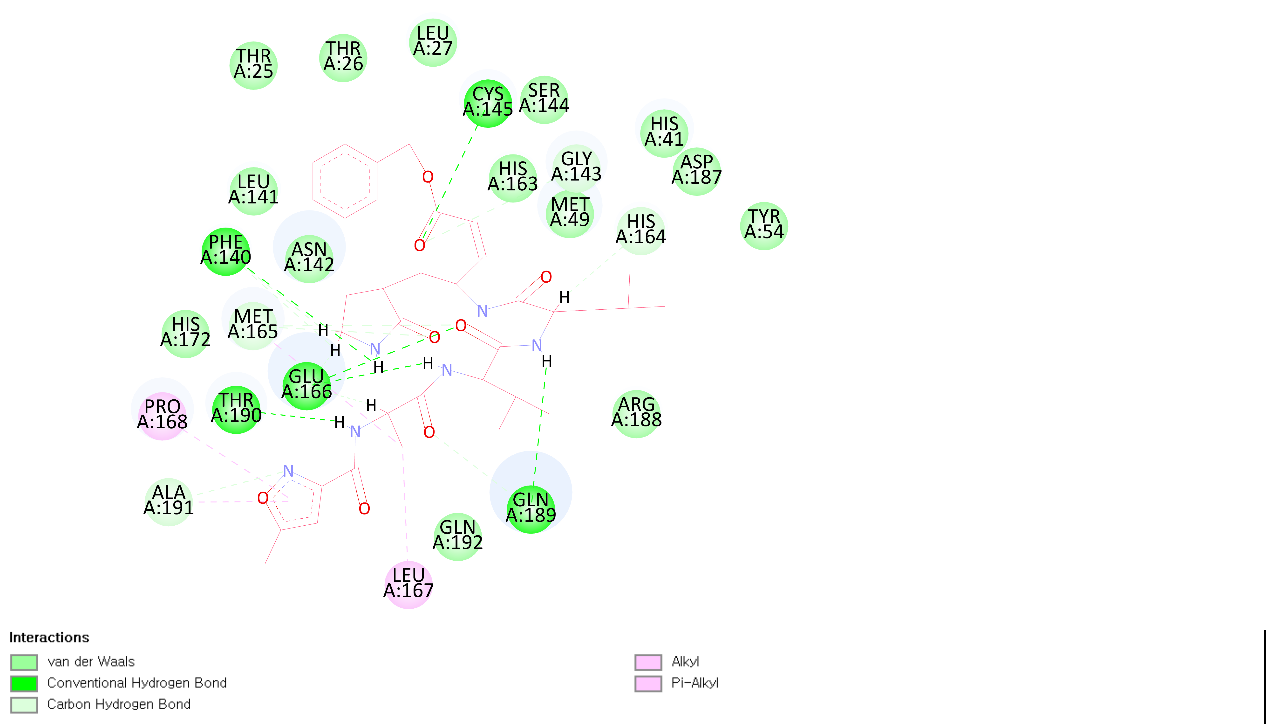


Supplementary Figure 4. 2D interactions of the cocrystallised ligand after molecular docking.


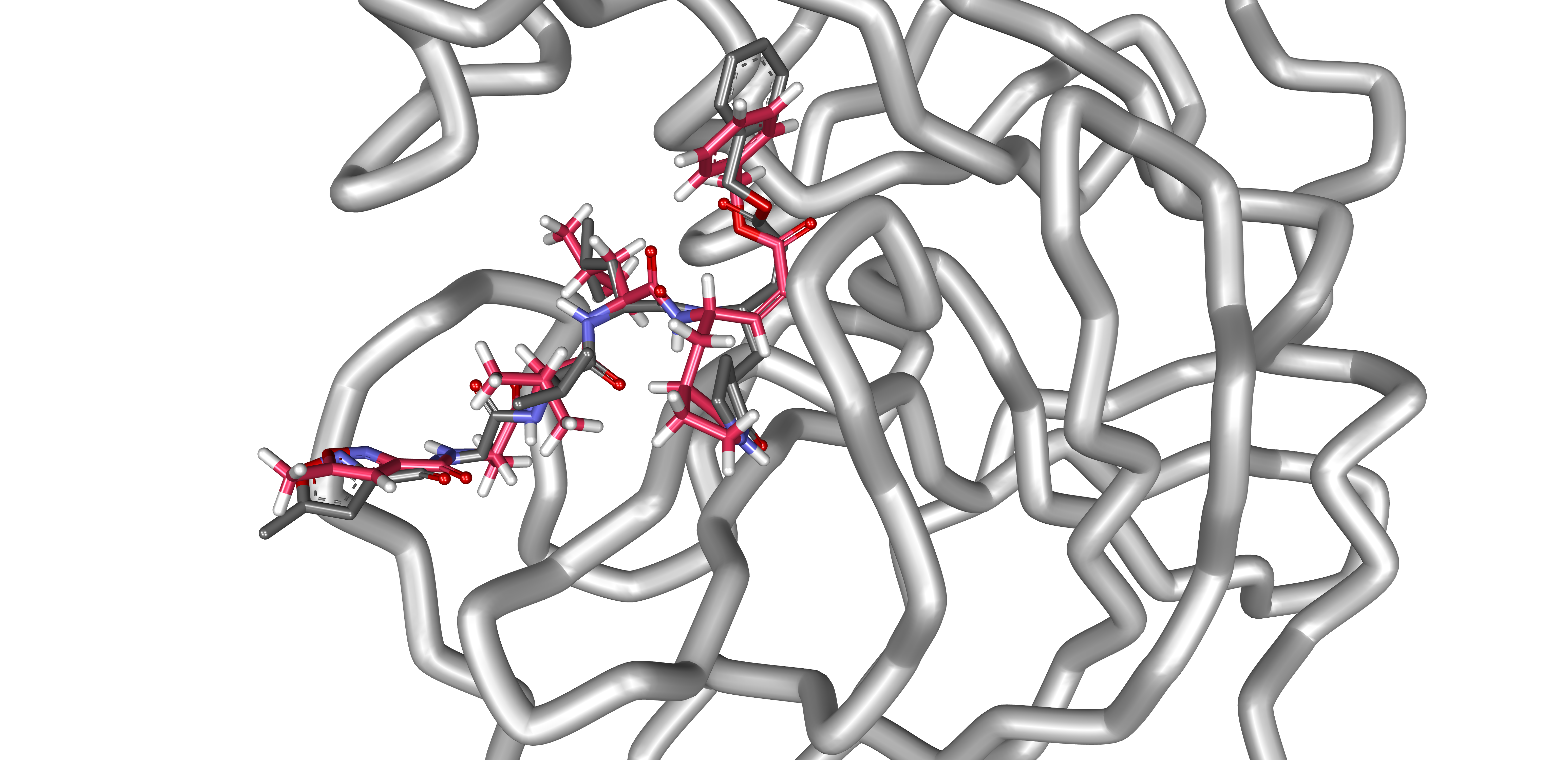


Supplementary Figure 5. Redocking of the cocrystallised ligand at the binding pocket of the target. The docked pose is represented in pink and the inbound cocrystallised ligand is represented in grey.
