# Supplementary material for: Repurposing and computational design of PARP inhibitors as SARS-CoV-2 inhibitors
Source: Sci Rep. 2023 Jun 29;13:10583. doi: 10.1038/s41598-023-36342-7 (PMC10310815; doi:10.1038/s41598-023-36342-7)
Supplement: Supplementary file 2 — Supplementary Table 1. [file 41598_2023_36342_MOESM2_ESM.docx]

**Supplementary Table 1.** Binding affinity calculations of the modified compound in comparison with the parent structures.

| -CDOCKER interaction energy (kcal/mol) | |
| --- | --- |
| Compounds | Main protease (6LU7) |
| Olaparib 1826 | 58.89 |
| Olaparib 1885 | 57.51 |
| Rucaparib 184 | 58.80 |
| Olaparib | 50.38 |
| Rucaparib | 54.15 |
