# Supplementary material for: Repurposing and computational design of PARP inhibitors as SARS-CoV-2 inhibitors
Source: Sci Rep. 2023 Jun 29;13:10583. doi: 10.1038/s41598-023-36342-7 (PMC10310815; doi:10.1038/s41598-023-36342-7)
Supplement: Supplementary file 3 — Supplementary Table 2. [file 41598_2023_36342_MOESM3_ESM.docx]

Supplementary Table 2. SMILES representation of the compounds generated through ‘Grow Scaffold’ module.

| SMILES | Name |
| --- | --- |
| Fc1ccc(CC2=NNC(=O)c3c(cccc23)C4CCC4)cc1C(=O)N5CCN(CC5)C(=O)C6CC6 | Olaparib 1885 |
| NCCc1cccc2C(=NNC(=O)c12)Cc3ccc(F)c(c3)C(=O)N4CCN(CC4)C(=O)C5CC5 | Olaparib 1826 |
| C[C@@H]1[C@@H]2C[C@@H]3C[C@H]1O[C@@](COC(=O)c4cccc5C(=NNC(=O)c45)Cc6ccc(F)c(c6)C(=O)N7CCN(CC7)C(=O)C8CC8)(C3)C2 | Olaparib 1170 |
| FC(F)C(F)(F)C(=O)Nc1cccc2C(=NNC(=O)c12)Cc3ccc(F)c(c3)C(=O)N4CCN(CC4)C(=O)C5CC5 | Olaparib 303 |
| CC(=O)N1[C@@H]2C[C@H]3C[C@@H](C2)C[C@]1(COC(=O)c4cccc5C(=NNC(=O)c45)Cc6ccc(F)c(c6)C(=O)N7CCN(CC7)C(=O)C8CC8)C3 | Olaparib 1165 |
| OS(=O)(=O)C(F)(F)[C@H](F)Oc1cccc2C(=NNC(=O)c12)Cc3ccc(F)c(c3)C(=O)N4CCN(CC4)C(=O)C5CC5 | Olaparib 2124 |
| Oc1cnc(cc1c2cccc3C(=NNC(=O)c23)Cc4ccc(F)c(c4)C(=O)N5CCN(CC5)C(=O)C6CC6)C(F)(F)F | Olaparib 1959 |
| Fc1ccc(CC2=NNC(=O)c3c2cccc3c4cc(ncc4Cl)C(F)(F)F)cc1C(=O)N5CCN(CC5)C(=O)C6CC6 | Olaparib 1957 |
| Fc1ccc(CC2=NNC(=O)c3c2cccc3c4cc(NCc5cnn6ccccc56)ccc4F)cc1C(=O)N7CCN(CC7)C(=O)C8CC8 | Olaparib 1787 |
| Fc1ccc(CC2=NNC(=O)c3c(OCC[C@@]45C[C@@H]6C[C@@H](C[C@@H](C6)C4)C5)cccc23)cc1C(=O)N7CCN(CC7)C(=O)C8CC8 | Olaparib 3201 |
| CCN(Cc1nc(Oc2cccc3C(=NNC(=O)c23)Cc4ccc(F)c(c4)C(=O)N5CCN(CC5)C(=O)C6CC6)c7cnn(C)c7n1)C8CC8 | Olaparib 2662 |
| Fc1cc(F)c2c(Cc3c2onc3C(=O)Oc4cccc5C(=NNC(=O)c45)Cc6ccc(F)c(c6)C(=O)N7CCN(CC7)C(=O)C8CC8)c1 | Olaparib 1586 |
| Fc1ccc(CC2=NNC(=O)c3c(cccc23)C(=O)NCc4noc(n4)c5coc6ccccc56)cc1C(=O)N7CCN(CC7)C(=O)C8CC8 | Olaparib 269 |
| NNc1c(F)cc(cc1c2cccc3C(=NNC(=O)c23)Cc4ccc(F)c(c4)C(=O)N5CCN(CC5)C(=O)C6CC6)C(=O)N7C[C@@H]8CC[C@H]7C8 | Olaparib 1785 |
| Fc1ccc(CC2=NNC(=O)c3c(NC(=O)C[C@@]45C[C@@H]6C[C@@H](C[C@@H](C6)C4)C5)cccc23)cc1C(=O)N7CCN(CC7)C(=O)C8CC8 | Olaparib 556 |
| Cc1cc(C)n(Cc2nc(Oc3cccc4C(=NNC(=O)c34)Cc5ccc(F)c(c5)C(=O)N6CCN(CC6)C(=O)C7CC7)c8cnn(C)c8n2)n1 | Olaparib 2635 |
| Fc1ccc(CC2=NNC(=O)c3c(cccc23)C(=O)OCC[C@@]45C[C@@H]6C[C@@H](C[C@@H](C6)C4)C5)cc1C(=O)N7CCN(CC7)C(=O)C8CC8 | Olaparib 1171 |
| FC(F)C(F)(F)C(=O)Oc1cccc2C(=NNC(=O)c12)Cc3ccc(F)c(c3)C(=O)N4CCN(CC4)C(=O)C5CC5 | Olaparib 1223 |
| CC(C)Cc1cccc2C(=NNC(=O)c12)Cc3ccc(F)c(c3)C(=O)N4CCN(CC4)C(=O)C5CC5 | Olaparib 1820 |
| [O-]S(=O)(=O)C(F)(F)C(F)(F)Oc1cccc2C(=NNC(=O)c12)Cc3ccc(F)c(c3)C(=O)N4CCN(CC4)C(=O)C5CC5 | Olaparib 2109 |
| Fc1ccc(CC2=NNC(=O)c3c(O[C@]4(F)C(F)(F)CC4(Cl)Cl)cccc23)cc1C(=O)N5CCN(CC5)C(=O)C6CC6 | Olaparib 2271 |
| O[C@H](c1ccccc1)c2c(F)c(F)c(Oc3cccc4C(=NNC(=O)c34)Cc5ccc(F)c(c5)C(=O)N6CCN(CC6)C(=O)C7CC7)c(F)c2F | Olaparib 2447 |
| Fc1ccc(CC2=NNC(=O)c3c(cccc23)C4CC4)cc1C(=O)N5CCN(CC5)C(=O)C6CC6 | Olaparib 1916 |
| Fc1ccc(CC2=NNC(=O)c3c(OC(=O)C(F)(F)C(F)(F)F)cccc23)cc1C(=O)N4CCN(CC4)C(=O)C5CC5 | Olaparib 1195 |
| OC(=O)[C@H]1CSCN1C(=O)NCCC(F)(F)c2cccc3C(=NNC(=O)c23)Cc4ccc(F)c(c4)C(=O)N5CCN(CC5)C(=O)C6CC6 | Olaparib 1646 |
| Fc1ccc(CC2=NNC(=O)c3c(O[C@@]4(F)CC[C@]4(F)Cl)cccc23)cc1C(=O)N5CCN(CC5)C(=O)C6CC6 | Olaparib 2420 |
| COc1ccc(cc1)\N=C(/c2cccc3C(=NNC(=O)c23)Cc4ccc(F)c(c4)C(=O)N5CCN(CC5)C(=O)C6CC6)\C(F)(F)F | Olaparib 1657 |
| Fc1ccc(CC2=NNC(=O)c3c(cccc23)C(=O)NC[C@@H]4CCC[C@H]4N5CCCc6ccccc6C5)cc1C(=O)N7CCN(CC7)C(=O)C8CC8 | Olaparib 254 |
| CNc1c(F)cc(cc1c2cccc3C(=NNC(=O)c23)Cc4ccc(F)c(c4)C(=O)N5CCN(CC5)C(=O)C6CC6)C(=O)N(C)C7CC7 | Olaparib 1750 |
| Fc1ccc(CC2=NNC(=O)c3c(cccc23)C(=O)NC[C@H]4Nc5ccccc5Cc6ccccc46)cc1C(=O)N7CCN(CC7)C(=O)C8CC8 | Olaparib 265 |
| Fc1ccc(CC2=NNC(=O)c3c(Oc4ncnc5c4cnn5c6ccccc6)cccc23)cc1C(=O)N7CCN(CC7)C(=O)C8CC8 | Olaparib 3196 |
| Cc1ccc(cc1)c2cn3cc(Oc4cccc5C(=NNC(=O)c45)Cc6ccc(F)c(c6)C(=O)N7CCN(CC7)C(=O)C8CC8)ccc3n2 | Olaparib 2721 |
| Fc1ccc(CC2=NNC(=O)c3c(OC(=O)C[C@@]45C[C@@H]6C[C@@H](C[C@@H](C6)C4)C5)cccc23)cc1C(=O)N7CCN(CC7)C(=O)C8CC8 | Olaparib 1600 |
| Fc1ccc(CC2=NNC(=O)c3c(cccc23)[C]4[CH][CH][CH][CH]4)cc1C(=O)N5CCN(CC5)C(=O)C6CC6 | Olaparib 2047 |
| Fc1ccc(CC2=NNC(=O)c3c(O[C@@]45C[C@@H]6C[C@@H](C[C@@H](C6)C4)C5)cccc23)cc1C(=O)N7CCN(CC7)C(=O)C8CC8 | Olaparib 2741 |
| FC(F)O[C@](F)(Oc1cccc2C(=NNC(=O)c12)Cc3ccc(F)c(c3)C(=O)N4CCN(CC4)C(=O)C5CC5)C(F)F | Olaparib 2135 |
| CC(C)(C#N)c1cc(Oc2cccc3C(=NNC(=O)c23)Cc4ccc(F)c(c4)C(=O)N5CCN(CC5)C(=O)C6CC6)c(cc1O)C(F)(F)F | Olaparib 2186 |
| Fc1ccc(CC2=NNC(=O)c3c(NC(=O)C(F)(F)F)cccc23)cc1C(=O)N4CCN(CC4)C(=O)C5CC5 | Olaparib 422 |
| Fc1ccc(CC2=NNC(=O)c3c(cccc23)C(=O)NCc4cnc5c(cnn5Cc6cccnc6)c4)cc1C(=O)N7CCN(CC7)C(=O)C8CC8 | Olaparib 260 |
| F[C@H](C(=O)Oc1cccc2C(=NNC(=O)c12)Cc3ccc(F)c(c3)C(=O)N4CCN(CC4)C(=O)C5CC5)C(F)(F)F | Olaparib 1224 |
| Fc1ccc2CN(C(=N)c2c1)c3ccc(Oc4cccc5C(=NNC(=O)c45)Cc6ccc(F)c(c6)C(=O)N7CCN(CC7)C(=O)C8CC8)c(F)c3 | Olaparib 2674 |
| Fc1ccc(CC2=NNC(=O)c3c(cccc23)C(=O)NCc4nc5ccccc5s4)cc1C(=O)N6CCN(CC6)C(=O)C7CC7 | Olaparib 248 |
| Cc1csc(NC(=O)OCC(F)(F)Oc2cccc3C(=NNC(=O)c23)Cc4ccc(F)c(c4)C(=O)N5CCN(CC5)C(=O)C6CC6)n1 | Olaparib 2219 |
| Fc1ccc(CC2=NNC(=O)c3c2cccc3[C@@]4(Cl)CC(F)(F)[C@@]4(F)Cl)cc1C(=O)N5CCN(CC5)C(=O)C6CC6 | Olaparib 1674 |
| Cc1ccc(C)c(c1)C2=NO[C@@H](COc3cccc4C(=NNC(=O)c34)Cc5ccc(F)c(c5)C(=O)N6CCN(CC6)C(=O)C7CC7)C2 | Olaparib 3098 |
| CC(C)n1nc(C)cc1c2cccc3C(=NNC(=O)c23)Cc4ccc(F)c(c4)C(=O)N5CCN(CC5)C(=O)C6CC6 | Olaparib 1982 |
| Fc1ccc(CC2=NNC(=O)c3c(cccc23)C(=O)NC[C@@H]4CCCN4C[C@@H]5CCCc6ccccc56)cc1C(=O)N7CCN(CC7)C(=O)C8CC8 | Olaparib 255 |
| Fc1ccc(CC2=NNC(=O)c3c(Oc4ncnc5c4cnn5C6CCCC6)cccc23)cc1C(=O)N7CCN(CC7)C(=O)C8CC8 | Olaparib 2737 |
| Nc1nccnc1c2onc(n2)c3cccc(Oc4cccc5C(=NNC(=O)c45)Cc6ccc(F)c(c6)C(=O)N7CCN(CC7)C(=O)C8CC8)c3 | Olaparib 2701 |
| F[C@@H](CC(F)(F)F)Oc1cccc2C(=NNC(=O)c12)Cc3ccc(F)c(c3)C(=O)N4CCN(CC4)C(=O)C5CC5 | Olaparib 2162 |
| NCCCc1cccc2C(=NNC(=O)c12)Cc3ccc(F)c(c3)C(=O)N4CCN(CC4)C(=O)C5CC5 | Olaparib 1819 |
| C[C@H](NC(=O)c1cccc2C(=NNC(=O)c12)Cc3ccc(F)c(c3)C(=O)N4CCN(CC4)C(=O)C5CC5)C(F)(F)F | Olaparib 83 |
| OC(=O)CC1(OCCO1)C(F)(F)Oc2cccc3C(=NNC(=O)c23)Cc4ccc(F)c(c4)C(=O)N5CCN(CC5)C(=O)C6CC6 | Olaparib 2316 |
| Fc1ccc(CC2=NNC(=O)c3c(NC(=O)Cc4nc5c(CCc6ccccc56)s4)cccc23)cc1C(=O)N7CCN(CC7)C(=O)C8CC8 | Olaparib 550 |
| Fc1ccc(CC2=NNC(=O)c3c2cccc3c4ccnc5cc(Cl)ccc45)cc1C(=O)N6CCN(CC6)C(=O)C7CC7 | Olaparib 1768 |
| Fc1ccc(CC2=NNC(=O)c3c(cccc23)C(=O)C=C)cc1C(=O)N4CCN(CC4)C(=O)C5CC5 | Olaparib 1909 |
| OS(=O)(=O)C(F)(F)[C@@H](F)c1cccc2C(=NNC(=O)c12)Cc3ccc(F)c(c3)C(=O)N4CCN(CC4)C(=O)C5CC5 | Olaparib 1607 |
| C[C@H]1CCN(C[C@@H](Oc2cccc3C(=NNC(=O)c23)Cc4ccc(F)c(c4)C(=O)N5CCN(CC5)C(=O)C6CC6)C(F)(F)F)[C@@H](C)C1 | Olaparib 2862 |
| CNC[C@@H]1CCN(C2CC2)[C@H]1c3ccc(Oc4cccc5C(=NNC(=O)c45)Cc6ccc(F)c(c6)C(=O)N7CCN(CC7)C(=O)C8CC8)c(F)c3 | Olaparib 2661 |
| Oc1cccc2cc3cccc(O)c3c(OC(=O)c4cccc5C(=NNC(=O)c45)Cc6ccc(F)c(c6)C(=O)N7CCN(CC7)C(=O)C8CC8)c12 | Olaparib 1158 |
| NS(=O)(=O)C(F)(F)Oc1cccc2C(=NNC(=O)c12)Cc3ccc(F)c(c3)C(=O)N4CCN(CC4)C(=O)C5CC5 | Olaparib 2149 |
| Fc1ccc(CC2=NNC(=O)c3c(NC(=O)Cc4onc(n4)[C@H]5C[C@H]6CC[C@@H]5O6)cccc23)cc1C(=O)N7CCN(CC7)C(=O)C8CC8 | Olaparib 555 |
| Fc1ccc2CN(C(=N)c2c1)c3ccc(F)c(Oc4cccc5C(=NNC(=O)c45)Cc6ccc(F)c(c6)C(=O)N7CCN(CC7)C(=O)C8CC8)c3 | Olaparib 2629 |
| C[C@@H](O)c1cc(Oc2cccc3C(=NNC(=O)c23)Cc4ccc(F)c(c4)C(=O)N5CCN(CC5)C(=O)C6CC6)ccc1N7CCc8sccc8C7 | Olaparib 2634 |
| Fc1ccc(CC2=NNC(=O)c3c2cccc3c4cc(nc5ccccc45)C(=O)NC6CCCC6)cc1C(=O)N7CCN(CC7)C(=O)C8CC8 | Olaparib 1782 |
| Fc1ccc(CC2=NNC(=O)c3c(cccc23)C(=O)OCc4onc(n4)c5ccc6ccccc6n5)cc1C(=O)N7CCN(CC7)C(=O)C8CC8 | Olaparib 1154 |
| NC(=N)c1cc(nc2ccccc12)n3cc(Oc4cccc5C(=NNC(=O)c45)Cc6ccc(F)c(c6)C(=O)N7CCN(CC7)C(=O)C8CC8)cn3 | Olaparib 2651 |
| CC(C)(C)OC(=O)\N=C(/C(F)(F)F)\C(F)(F)Oc1cccc2C(=NNC(=O)c12)Cc3ccc(F)c(c3)C(=O)N4CCN(CC4)C(=O)C5CC5 | Olaparib 2094 |
| Fc1ccc(CC2=NNC(=O)c3c(Oc4ccc(cc4)C(n5cncn5)n6cncn6)cccc23)cc1C(=O)N7CCN(CC7)C(=O)C8CC8 | Olaparib 2680 |
| Fc1ccc(CC2=NNC(=O)c3c(cccc23)C(=C)C=C)cc1C(=O)N4CCN(CC4)C(=O)C5CC5 | Olaparib 1845 |
| FC(F)CC(F)(F)Oc1cccc2C(=NNC(=O)c12)Cc3ccc(F)c(c3)C(=O)N4CCN(CC4)C(=O)C5CC5 | Olaparib 2161 |
| Cc1ccc(cc1)[C@H](N)C(F)(F)C(=O)Oc2cccc3C(=NNC(=O)c23)Cc4ccc(F)c(c4)C(=O)N5CCN(CC5)C(=O)C6CC6 | Olaparib 1432 |
| Fc1ccc(CC2=NNC(=O)c3c(NC(=O)C(F)(F)C(F)(F)F)cccc23)cc1C(=O)N4CCN(CC4)C(=O)C5CC5 | Olaparib 285 |
| OC(=O)C(F)(F)C(=O)Nc1cccc2C(=NNC(=O)c12)Cc3ccc(F)c(c3)C(=O)N4CCN(CC4)C(=O)C5CC5 | Olaparib 325 |
| NCC(=C)c1cccc2C(=NNC(=O)c12)Cc3ccc(F)c(c3)C(=O)N4CCN(CC4)C(=O)C5CC5 | Olaparib 1917 |
| Fc1ccc(CC2=NNC(=O)c3c2cccc3c4cccc5ccnc(Cl)c45)cc1C(=O)N6CCN(CC6)C(=O)C7CC7 | Olaparib 2061 |
| FC(=C(Cl)C(=O)Nc1cccc2C(=NNC(=O)c12)Cc3ccc(F)c(c3)C(=O)N4CCN(CC4)C(=O)C5CC5)F | Olaparib 318 |
| Fc1cncc(c1)c2cccc3C(=NNC(=O)c23)Cc4ccc(F)c(c4)C(=O)N5CCN(CC5)C(=O)C6CC6 | Olaparib 2016 |
| Fc1ccc(CC2=NNC(=O)c3c2cccc3c4c[nH]c5ncc(cc45)c6ccccc6)cc1C(=O)N7CCN(CC7)C(=O)C8CC8 | Olaparib 2088 |
| N[C@@]([C@@H]1CCCN1)(C(=O)Nc2cccc3C(=NNC(=O)c23)Cc4ccc(F)c(c4)C(=O)N5CCN(CC5)C(=O)C6CC6)C(F)(F)F | Olaparib 472 |
| NC1(CC1)c2cccc(OCc3cccc(Oc4cccc5C(=NNC(=O)c45)Cc6ccc(F)c(c6)C(=O)N7CCN(CC7)C(=O)C8CC8)c3)c2 | Olaparib 2697 |
| Fc1ccc(CC2=NNC(=O)c3c(Oc4ccc5NC(=O)c6ccsc6Nc5c4)cccc23)cc1C(=O)N7CCN(CC7)C(=O)C8CC8 | Olaparib 2728 |
| N[C@@]([C@@H]1CCCN1)(C(=O)Oc2cccc3C(=NNC(=O)c23)Cc4ccc(F)c(c4)C(=O)N5CCN(CC5)C(=O)C6CC6)C(F)(F)F | Olaparib 1437 |
| Fc1ccc(CC2=NNC(=O)c3c(OC(=O)Cc4nc5c(CCc6ccccc56)s4)cccc23)cc1C(=O)N7CCN(CC7)C(=O)C8CC8 | Olaparib 1579 |
| Cn1cc(Cn2nc(CNC(=O)c3cccc4C(=NNC(=O)c34)Cc5ccc(F)c(c5)C(=O)N6CCN(CC6)C(=O)C7CC7)c8ccccc28)cn1 | Olaparib 258 |
| Fc1ccc(CC2=NNC(=O)c3c2cccc3c4c[nH]c5cc6OCOc6cc45)cc1C(=O)N7CCN(CC7)C(=O)C8CC8 | Olaparib 2091 |
| Cc1ccc2Cc3c(onc3C(=O)Oc4cccc5C(=NNC(=O)c45)Cc6ccc(F)c(c6)C(=O)N7CCN(CC7)C(=O)C8CC8)c2c1 | Olaparib 1599 |
| Fc1ccc(CC2=NNC(=O)c3c(cccc23)C(=O)Oc4ccccc4c5onc(n5)c6ccsc6)cc1C(=O)N7CCN(CC7)C(=O)C8CC8 | Olaparib 1130 |
| Fc1ccc(CC2=NNC(=O)c3c(O[C@H]4CSc5nc6ccccc6n5C4)cccc23)cc1C(=O)N7CCN(CC7)C(=O)C8CC8 | Olaparib 3199 |
| COCCc1cccc2C(=NNC(=O)c12)Cc3ccc(F)c(c3)C(=O)N4CCN(CC4)C(=O)C5CC5 | Olaparib 1950 |
| Fc1ccc(CC2=NNC(=O)c3c(CCC=C)cccc23)cc1C(=O)N4CCN(CC4)C(=O)C5CC5 | Olaparib 1822 |
| Fc1ccc(CC2=NNC(=O)c3c(OC(F)(F)C(F)(F)I)cccc23)cc1C(=O)N4CCN(CC4)C(=O)C5CC5 | Olaparib 2098 |
| Fc1ccc(CC2=NNC(=O)c3c(Oc4ccc5C(=O)C=CN(Cc6nn[nH]n6)c5c4)cccc23)cc1C(=O)N7CCN(CC7)C(=O)C8CC8 | Olaparib 2719 |
| C[C@H](OC(=O)N)C(F)(F)[C@@H](F)Oc1cccc2C(=NNC(=O)c12)Cc3ccc(F)c(c3)C(=O)N4CCN(CC4)C(=O)C5CC5 | Olaparib 2113 |
| Fc1ccc(CC2=NNC(=O)c3c(OC4C[C@H]5CC[C@@H](C4)N5C(=O)[C@H]6CCSC6)cccc23)cc1C(=O)N7CCN(CC7)C(=O)C8CC8 | Olaparib 3166 |
| CON(C)C(=O)c1ccc(Cl)c(c1)c2cccc3C(=NNC(=O)c23)Cc4ccc(F)c(c4)C(=O)N5CCN(CC5)C(=O)C6CC6 | Olaparib 1664 |
| Fc1ccc(CC2=NNC(=O)c3c(O[C@@]4(F)CCC4(F)F)cccc23)cc1C(=O)N5CCN(CC5)C(=O)C6CC6 | Olaparib 2421 |
| Fc1ccc(CC2=NNC(=O)c3c(cccc23)C(=O)NC(=S)\C(=C\c4ccccc4Cl)\C#N)cc1C(=O)N5CCN(CC5)C(=O)C6CC6 | Olaparib 132 |
| FC(F)C(F)(F)O[C@@H](F)c1cccc2C(=NNC(=O)c12)Cc3ccc(F)c(c3)C(=O)N4CCN(CC4)C(=O)C5CC5 | Olaparib 1615 |
| Fc1ccc(CC2=NNC(=O)c3c2cccc3c4c(cnc5[nH]ccc45)c6ccccc6)cc1C(=O)N7CCN(CC7)C(=O)C8CC8 | Olaparib 2085 |
| Cc1ncsc1Cn2c(N)nc3cc(Oc4cccc5C(=NNC(=O)c45)Cc6ccc(F)c(c6)C(=O)N7CCN(CC7)C(=O)C8CC8)ccc23 | Olaparib 2626 |
| C[C@@H](NC(=O)c1cccc2C(=NNC(=O)c12)Cc3ccc(F)c(c3)C(=O)N4CCN(CC4)C(=O)C5CC5)c6cnc(s6)[C@H]7C[C@H]8CC[C@@H]7O8 | Olaparib 264 |
| Fc1ccc(CC2=NNC(=O)c3c(cccc23)C(=O)NCc4cnn(Cc5ccc6ncccc6c5)c4)cc1C(=O)N7CCN(CC7)C(=O)C8CC8 | Olaparib 261 |
| Nc1ccc(cc1c2cccc3C(=NNC(=O)c23)Cc4ccc(F)c(c4)C(=O)N5CCN(CC5)C(=O)C6CC6)C(=O)NC[C@@H]7C[C@@H]8CC[C@H]7C8 | Olaparib 1778 |
| Fc1ccc(CC2=NNC(=O)c3c(OC(=O)Cc4cccc5c4oc6ccccc56)cccc23)cc1C(=O)N7CCN(CC7)C(=O)C8CC8 | Olaparib 1594 |
| Nc1ccnc2c1c(COC(=O)c3cccc4C(=NNC(=O)c34)Cc5ccc(F)c(c5)C(=O)N6CCN(CC6)C(=O)C7CC7)nn2[C@@H]8CCOC8 | Olaparib 1148 |
| Fc1ccc(CC2=NNC(=O)c3c(CCC(F)(F)F)cccc23)cc1C(=O)N4CCN(CC4)C(=O)C5CC5 | Olaparib 1803 |
| CC[C@@H](C)c1cccc2C(=NNC(=O)c12)Cc3ccc(F)c(c3)C(=O)N4CCN(CC4)C(=O)C5CC5 | Olaparib 1821 |
| Fc1ccc(CC2=NNC(=O)c3c(OCCN4c5ccccc5Oc6ccccc46)cccc23)cc1C(=O)N7CCN(CC7)C(=O)C8CC8 | Olaparib 3187 |
| CC(=Cc1cccc2C(=NNC(=O)c12)Cc3ccc(F)c(c3)C(=O)N4CCN(CC4)C(=O)C5CC5)C | Olaparib 1851 |
| CC(C)(C(=O)Nc1cccc2C(=NNC(=O)c12)Cc3ccc(F)c(c3)C(=O)N4CCN(CC4)C(=O)C5CC5)c6cc(Cl)ccc6O | Olaparib 469 |
| Fc1ccc2CN(C(=N)c2c1)c3ccc(F)c(c3)c4cccc5C(=NNC(=O)c45)Cc6ccc(F)c(c6)C(=O)N7CCN(CC7)C(=O)C8CC8 | Olaparib 1779 |
| Fc1ccc(CC2=NNC(=O)c3c(OC4C[C@H]5CC[C@@H](C4)N5C(=O)[C@H]6CCCS6)cccc23)cc1C(=O)N7CCN(CC7)C(=O)C8CC8 | Olaparib 3165 |
| COc1c(F)cc(cc1c2cccc3C(=NNC(=O)c23)Cc4ccc(F)c(c4)C(=O)N5CCN(CC5)C(=O)C6CC6)C(C)(C)N | Olaparib 1670 |
| C[C@@](O)(C(=O)O)C(F)(F)Oc1cccc2C(=NNC(=O)c12)Cc3ccc(F)c(c3)C(=O)N4CCN(CC4)C(=O)C5CC5 | Olaparib 2143 |
| Fc1ccc(CC2=NNC(=O)c3c(OC(F)(F)C(F)(F)F)cccc23)cc1C(=O)N4CCN(CC4)C(=O)C5CC5 | Olaparib 2100 |
| OS(=O)(=O)CC(F)(F)Oc1cccc2C(=NNC(=O)c12)Cc3ccc(F)c(c3)C(=O)N4CCN(CC4)C(=O)C5CC5 | Olaparib 2138 |
| Fc1ccc(CC2=NNC(=O)c3c(\C=C\C(F)(F)F)cccc23)cc1C(=O)N4CCN(CC4)C(=O)C5CC5 | Olaparib 1928 |
| O[C@H](C(=O)Oc1cccc2C(=NNC(=O)c12)Cc3ccc(F)c(c3)C(=O)N4CCN(CC4)C(=O)C5CC5)C(F)(F)F | Olaparib 1235 |
| Fc1ccc(CC2=NNC(=O)c3c(OC(=O)Cc4csc(n4)[C@H]5C[C@H]6CC[C@@H]5O6)cccc23)cc1C(=O)N7CCN(CC7)C(=O)C8CC8 | Olaparib 1585 |
| Fc1ccc(CC2=NNC(=O)c3c(cccc23)C(=O)NCc4cnc(s4)c5ccc6ccccc6n5)cc1C(=O)N7CCN(CC7)C(=O)C8CC8 | Olaparib 257 |
| C[C@@H](NC(=O)c1cccc2C(=NNC(=O)c12)Cc3ccc(F)c(c3)C(=O)N4CCN(CC4)C(=O)C5CC5)c6oc(nn6)[C@@H]7CCOc8ccccc78 | Olaparib 253 |
| CNC[C@@H]1CCN(C2CC2)[C@H]1c3ccc(F)c(Oc4cccc5C(=NNC(=O)c45)Cc6ccc(F)c(c6)C(=O)N7CCN(CC7)C(=O)C8CC8)c3 | Olaparib 2660 |
| Fc1ccc(CC2=NNC(=O)c3c(Oc4ccc(NC(=O)[C@H]5C[C@H]6CC[C@@H]5C6)cc4F)cccc23)cc1C(=O)N7CCN(CC7)C(=O)C8CC8 | Olaparib 3148 |
| Fc1ccc(CC2=NNC(=O)c3c(cccc23)C(=O)OCc4nc5NC(=C)C=C(c6ccccc6)n5n4)cc1C(=O)N7CCN(CC7)C(=O)C8CC8 | Olaparib 1139 |
| O[C@H](c1ccc(Oc2cccc3C(=NNC(=O)c23)Cc4ccc(F)c(c4)C(=O)N5CCN(CC5)C(=O)C6CC6)cc1)c7ccnc8ccccc78 | Olaparib 2706 |
| Fc1ccc(CC2=NNC(=O)c3c(cccc23)C(=O)OCc4cc(c5ccccc5n4)n6cncn6)cc1C(=O)N7CCN(CC7)C(=O)C8CC8 | Olaparib 1157 |
| FC(F)CC(F)(F)c1cccc2C(=NNC(=O)c12)Cc3ccc(F)c(c3)C(=O)N4CCN(CC4)C(=O)C5CC5 | Olaparib 1638 |
| CCCCc1cccc2C(=NNC(=O)c12)Cc3ccc(F)c(c3)C(=O)N4CCN(CC4)C(=O)C5CC5 | Olaparib 1830 |
| OC[C@@H](C(=O)O)C(F)(F)Oc1cccc2C(=NNC(=O)c12)Cc3ccc(F)c(c3)C(=O)N4CCN(CC4)C(=O)C5CC5 | Olaparib 2142 |
| Cc1c(Cl)cncc1c2cccc3C(=NNC(=O)c23)Cc4ccc(F)c(c4)C(=O)N5CCN(CC5)C(=O)C6CC6 | Olaparib 1978 |
| Fc1ccc(CC2=NNC(=O)c3c(CC=C)cccc23)cc1C(=O)N4CCN(CC4)C(=O)C5CC5 | Olaparib 1831 |
| Fc1ccc(CC2=NNC(=O)c3c(cccc23)C(=O)NCCOc4ccc(Cl)cc4Cl)cc1C(=O)N5CCN(CC5)C(=O)C6CC6 | Olaparib 144 |
| Fc1ccc(CC2=NNC(=O)c3c(cccc23)C(=O)NCc4ccc(cc4F)c5cnccn5)cc1C(=O)N6CCN(CC6)C(=O)C7CC7 | Olaparib 239 |
| FC(=C(Cl)C(=O)Oc1cccc2C(=NNC(=O)c12)Cc3ccc(F)c(c3)C(=O)N4CCN(CC4)C(=O)C5CC5)F | Olaparib 1241 |
| OC(=O)C(=O)C(F)(F)Oc1cccc2C(=NNC(=O)c12)Cc3ccc(F)c(c3)C(=O)N4CCN(CC4)C(=O)C5CC5 | Olaparib 2154 |
| Fc1ccc(CC2=NNC(=O)c3c2cccc3c4ccccc4CN5C[C@H]6CC[C@@H]5C6)cc1C(=O)N7CCN(CC7)C(=O)C8CC8 | Olaparib 2090 |
| Fc1ccc(CC2=NNC(=O)c3c(NC(=O)CO\N=C/4\C[N@@]5CC[C@H]4CC5)cccc23)cc1C(=O)N6CCN(CC6)C(=O)C7CC7 | Olaparib 542 |
| Fc1ccc(CC2=NNC(=O)c3c(Oc4ccc5nnc(c6ccc(Cl)cc6)n5n4)cccc23)cc1C(=O)N7CCN(CC7)C(=O)C8CC8 | Olaparib 2665 |
| Fc1ccc(CC2=NNC(=O)c3c2cccc3c4cccc(N5CCC[N@@]6CCC[C@H]6C5)c4C#N)cc1C(=O)N7CCN(CC7)C(=O)C8CC8 | Olaparib 1781 |
| Fc1ccc(CC2=NNC(=O)c3c(Oc4cncc(c4)c5onc(C[C@H]6CCCO6)n5)cccc23)cc1C(=O)N7CCN(CC7)C(=O)C8CC8 | Olaparib 3153 |
| Fc1ccc(CC2=NNC(=O)c3c2cccc3c4ccnc5[nH]c(cc45)c6ccccc6)cc1C(=O)N7CCN(CC7)C(=O)C8CC8 | Olaparib 2087 |
| Nc1ncc2[C@H](OC(=O)c3cccc4C(=NNC(=O)c34)Cc5ccc(F)c(c5)C(=O)N6CCN(CC6)C(=O)C7CC7)Oc8ccccc8c2n1 | Olaparib 1160 |
| Fc1ccc(CC2=NNC(=O)c3c(O[C@H](c4oc5ccccc5c4)c6cocc6)cccc23)cc1C(=O)N7CCN(CC7)C(=O)C8CC8 | Olaparib 3195 |
| Fc1ccc(CC2=NNC(=O)c3c(cccc23)C(=O)Oc4ccc(cc4)c5n[nH]c(n5)c6cccs6)cc1C(=O)N7CCN(CC7)C(=O)C8CC8 | Olaparib 1132 |
| F[C@H](CC(F)(F)F)c1cccc2C(=NNC(=O)c12)Cc3ccc(F)c(c3)C(=O)N4CCN(CC4)C(=O)C5CC5 | Olaparib 1639 |
| CCc1cccc2C(=NNC(=O)c12)Cc3ccc(F)c(c3)C(=O)N4CCN(CC4)C(=O)C5CC5 | Olaparib 1817 |
| COc1ccc2nc(Oc3cccc4C(=NNC(=O)c34)Cc5ccc(F)c(c5)C(=O)N6CCN(CC6)C(=O)C7CC7)c(C=O)c(Cl)c2c1 | Olaparib 2489 |
| Fc1ccc(CC2=NNC(=O)c3c(C=C)cccc23)cc1C(=O)N4CCN(CC4)C(=O)C5CC5 | Olaparib 1796 |
| COC(=O)c1c(F)c(F)c(Oc2cccc3C(=NNC(=O)c23)Cc4ccc(F)c(c4)C(=O)N5CCN(CC5)C(=O)C6CC6)c(F)c1F | Olaparib 2242 |
| Fc1ccc(CC2=NNC(=O)c3c2cccc3c4nnc5oc6ccccc6cc45)cc1C(=O)N7CCN(CC7)C(=O)C8CC8 | Olaparib 1790 |
| Fc1ccc(CC2=NNC(=O)c3c2cccc3c4cnc5ccc(Cl)nn45)cc1C(=O)N6CCN(CC6)C(=O)C7CC7 | Olaparib 2068 |
| OP(=O)(O)CNC(=O)c1cccc2C(=NNC(=O)c12)Cc3ccc(F)c(c3)C(=O)N4CCN(CC4)C(=O)C5CC5 | Olaparib 86 |
| Fc1ccc(CC2=NNC(=O)c3c(OC(=O)CCn4cnc5cc6OCCOc6cc45)cccc23)cc1C(=O)N7CCN(CC7)C(=O)C8CC8 | Olaparib 1576 |
| Fc1ccc(CC2=NNC(=O)c3c(NC(=O)C(C(F)(F)F)C(F)(F)F)cccc23)cc1C(=O)N4CCN(CC4)C(=O)C5CC5 | Olaparib 274 |
| CCC(=O)[C@@](F)(C(=O)OC)c1cccc2C(=NNC(=O)c12)Cc3ccc(F)c(c3)C(=O)N4CCN(CC4)C(=O)C5CC5 | Olaparib 1616 |
| OP(=O)(O)COc1cccc2C(=NNC(=O)c12)Cc3ccc(F)c(c3)C(=O)N4CCN(CC4)C(=O)C5CC5 | Olaparib 2816 |
| Cc1cc(NC(=O)c2ccc(N)c(c2)c3cccc4C(=NNC(=O)c34)Cc5ccc(F)c(c5)C(=O)N6CCN(CC6)C(=O)C7CC7)[nH]n1 | Olaparib 1747 |
| Cc1ccc2sc3nc(CC(=O)Nc4cccc5C(=NNC(=O)c45)Cc6ccc(F)c(c6)C(=O)N7CCN(CC7)C(=O)C8CC8)cn3c2c1 | Olaparib 548 |
| Fc1ccc(CC2=NNC(=O)c3c2cccc3c4c[nH]c5nc(ccc45)c6ccccc6)cc1C(=O)N7CCN(CC7)C(=O)C8CC8 | Olaparib 2086 |
| C[C@@H](NC(=O)c1cccc2C(=NNC(=O)c12)Cc3ccc(F)c(c3)C(=O)N4CCN(CC4)C(=O)C5CC5)c6noc(n6)[C@@H]7COc8ccccc8C7 | Olaparib 252 |
| CCCc1cccc2C(=NNC(=O)c12)Cc3ccc(F)c(c3)C(=O)N4CCN(CC4)C(=O)C5CC5 | Olaparib 1807 |
| Fc1ccc(CC2=NNC(=O)c3c(OC(=O)C(F)(F)F)cccc23)cc1C(=O)N4CCN(CC4)C(=O)C5CC5 | Olaparib 1368 |
| Fc1ccc(CC2=NNC(=O)c3c(cccc23)C(=O)Oc4ncnc5c4cnn5c6ccccc6)cc1C(=O)N7CCN(CC7)C(=O)C8CC8 | Olaparib 1162 |
| OC(=O)C[C@@](O)(C(F)(F)F)C(F)(F)Oc1cccc2C(=NNC(=O)c12)Cc3ccc(F)c(c3)C(=O)N4CCN(CC4)C(=O)C5CC5 | Olaparib 2101 |
| Nc1c(F)c(N)c(Oc2cccc3C(=NNC(=O)c23)Cc4ccc(F)c(c4)C(=O)N5CCN(CC5)C(=O)C6CC6)c(F)c1F | Olaparib 2358 |
| Fc1ccc(CC2=NNC(=O)c3c(Oc4ccc(cc4)C5C(=O)c6ccccc6C5=O)cccc23)cc1C(=O)N7CCN(CC7)C(=O)C8CC8 | Olaparib 2723 |
| CCN1CC[C@@H](CNC(=O)c2cccc3C(=NNC(=O)c23)Cc4ccc(F)c(c4)C(=O)N5CCN(CC5)C(=O)C6CC6)[C@@H]1c7ccccc7 | Olaparib 235 |
| CC(C)[C@@H](O)C(F)(F)C(=O)Nc1cccc2C(=NNC(=O)c12)Cc3ccc(F)c(c3)C(=O)N4CCN(CC4)C(=O)C5CC5 | Olaparib 282 |
| CC1(C)CCCN(C1)[C@@]2(CNC(=O)c3cccc4C(=NNC(=O)c34)Cc5ccc(F)c(c5)C(=O)N6CCN(CC6)C(=O)C7CC7)CCOC2 | Olaparib 230 |
| Fc1ccc(CC2=NNC(=O)c3c(NC(=O)CN4C(=O)c5ccccc5C4=O)cccc23)cc1C(=O)N6CCN(CC6)C(=O)C7CC7 | Olaparib 539 |
| Fc1ccc(CC2=NNC(=O)c3c(OC(=O)Cc4ncc5c6ccoc6ccn45)cccc23)cc1C(=O)N7CCN(CC7)C(=O)C8CC8 | Olaparib 1598 |
| C[C@@H](NC(=O)c1cccc2C(=NNC(=O)c12)Cc3ccc(F)c(c3)C(=O)N4CCN(CC4)C(=O)C5CC5)c6ccc7c(Cc8ccccc78)c6 | Olaparib 270 |
| Cc1ccc(CN2Cc3ccc(Oc4cccc5C(=NNC(=O)c45)Cc6ccc(F)c(c6)C(=O)N7CCN(CC7)C(=O)C8CC8)cc3C2=N)cc1 | Olaparib 2705 |
| Fc1ccc(CC2=NNC(=O)c3c2cccc3c4ccnc5c4ccc6c(Cl)ccnc56)cc1C(=O)N7CCN(CC7)C(=O)C8CC8 | Olaparib 1788 |
| CC[C@@H](NC(=O)c1cccc2C(=NNC(=O)c12)Cc3ccc(F)c(c3)C(=O)N4CCN(CC4)C(=O)C5CC5)c6onc(n6)[C@H]7C[C@H]8CC[C@@H]7O8 | Olaparib 267 |
| Cc1noc(COc2cccc3[C@@H](CCc23)OC(=O)c4cccc5C(=NNC(=O)c45)Cc6ccc(F)c(c6)C(=O)N7CCN(CC7)C(=O)C8CC8)n1 | Olaparib 1127 |
| Cc1cccc2C(=NNC(=O)c12)Cc3ccc(F)c(c3)C(=O)N4CCN(CC4)C(=O)C5CC5 | Olaparib 1799 |
| Fc1ccc(CC2=NNC(=O)c3c(OC(=O)C(F)(F)Cl)cccc23)cc1C(=O)N4CCN(CC4)C(=O)C5CC5 | Olaparib 1287 |
| COc1cc(N)c(Oc2cccc3C(=NNC(=O)c23)Cc4ccc(F)c(c4)C(=O)N5CCN(CC5)C(=O)C6CC6)cc1c7nc8ccncc8[nH]7 | Olaparib 2640 |
| C[C@@](N)(C(=O)O)C(F)(F)Oc1cccc2C(=NNC(=O)c12)Cc3ccc(F)c(c3)C(=O)N4CCN(CC4)C(=O)C5CC5 | Olaparib 2146 |
| CN1C(=O)C(=C(C1=O)c2cccc3C(=NNC(=O)c23)Cc4ccc(F)c(c4)C(=O)N5CCN(CC5)C(=O)C6CC6)Br | Olaparib 1648 |
| Fc1ccc(CC2=NNC(=O)c3c(Oc4ccc5C(=O)C(=COc5c4)c6ccccc6)cccc23)cc1C(=O)N7CCN(CC7)C(=O)C8CC8 | Olaparib 3172 |
| O[C@H](c1ccccc1)c2c(F)c(F)c(F)c(F)c2Oc3cccc4C(=NNC(=O)c34)Cc5ccc(F)c(c5)C(=O)N6CCN(CC6)C(=O)C7CC7 | Olaparib 2445 |
| Fc1ccc(CC2=NNC(=O)c3c(cccc23)C(=O)OCc4onc(n4)[C@H]5C[C@H]6CC[C@@H]5O6)cc1C(=O)N7CCN(CC7)C(=O)C8CC8 | Olaparib 1169 |
| Cc1ccc(s1)C2=NO[C@](C)(C2)C(=O)Nc3cccc4C(=NNC(=O)c34)Cc5ccc(F)c(c5)C(=O)N6CCN(CC6)C(=O)C7CC7 | Olaparib 531 |
| CC(C)[C@@H](O)C(F)(F)C(=O)Oc1cccc2C(=NNC(=O)c12)Cc3ccc(F)c(c3)C(=O)N4CCN(CC4)C(=O)C5CC5 | Olaparib 1190 |
| Fc1ccc([C@@H]2CCC(=O)N2)c(Oc3cccc4C(=NNC(=O)c34)Cc5ccc(F)c(c5)C(=O)N6CCN(CC6)C(=O)C7CC7)c1F | Olaparib 3087 |
| Fc1ccc(CC2=NNC(=O)c3c(NC(=O)CC4c5ccccc5c6ccccc46)cccc23)cc1C(=O)N7CCN(CC7)C(=O)C8CC8 | Olaparib 554 |
| O[C@H](c1ccc2OCOc2c1)c3cncc(Oc4cccc5C(=NNC(=O)c45)Cc6ccc(F)c(c6)C(=O)N7CCN(CC7)C(=O)C8CC8)c3 | Olaparib 2713 |
| CC(=O)Nc1ccc2c(Cc3cc(Oc4cccc5C(=NNC(=O)c45)Cc6ccc(F)c(c6)C(=O)N7CCN(CC7)C(=O)C8CC8)ccc23)c1 | Olaparib 2693 |
| Cc1oc(COc2cccc3[C@@H](CCc23)OC(=O)c4cccc5C(=NNC(=O)c45)Cc6ccc(F)c(c6)C(=O)N7CCN(CC7)C(=O)C8CC8)nn1 | Olaparib 1126 |
| Fc1ccc(CC2=NNC(=O)c3c(Oc4ccc(C[C@H]5C(=O)Nc6ccccc56)cc4)cccc23)cc1C(=O)N7CCN(CC7)C(=O)C8CC8 | Olaparib 2694 |
| Fc1ccc(CC2=NNC(=O)c3c2cccc3C(F)(F)C(F)(F)F)cc1C(=O)N4CCN(CC4)C(=O)C5CC5 | Olaparib 1604 |
| Oc1ccc(Oc2cccc3C(=NNC(=O)c23)Cc4ccc(F)c(c4)C(=O)N5CCN(CC5)C(=O)C6CC6)cc1c7onc(n7)c8ccsc8 | Olaparib 2628 |
| N[C@H](COc1cccc2C(=NNC(=O)c12)Cc3ccc(F)c(c3)C(=O)N4CCN(CC4)C(=O)C5CC5)C(=O)O | Olaparib 2165 |
| Fc1ccc(CC2=NNC(=O)c3c(OC4=CC(=O)NC(=C4)C(F)(F)F)cccc23)cc1C(=O)N5CCN(CC5)C(=O)C6CC6 | Olaparib 2900 |
| Fc1ccc(CC2=NNC(=O)c3c(Oc4nc(NC=O)nc(Cl)c4NC=O)cccc23)cc1C(=O)N5CCN(CC5)C(=O)C6CC6 | Olaparib 2226 |
| Fc1ccc(CC2=NNC(=O)c3c(OC(=O)\C=C\C(F)(F)F)cccc23)cc1C(=O)N4CCN(CC4)C(=O)C5CC5 | Olaparib 1248 |
| Fc1ccc(CC2=NNC(=O)c3c(OCCN4C(=O)Sc5ccccc45)cccc23)cc1C(=O)N6CCN(CC6)C(=O)C7CC7 | Olaparib 3104 |
| CC1=CC(=O)N(C1=O)C2(CC(=O)Nc3cccc4C(=NNC(=O)c34)Cc5ccc(F)c(c5)C(=O)N6CCN(CC6)C(=O)C7CC7)CCCC2 | Olaparib 525 |
| O[C@@H](C[C@H]1CCCO1)c2cccc(Oc3cccc4C(=NNC(=O)c34)Cc5ccc(F)c(c5)C(=O)N6CCN(CC6)C(=O)C7CC7)c2F | Olaparib 2550 |
| C[C@]1(C[C@@H](CCO1)Oc2cccc3C(=NNC(=O)c23)Cc4ccc(F)c(c4)C(=O)N5CCN(CC5)C(=O)C6CC6)c7ccc8ccccc8c7 | Olaparib 3164 |
| Cc1c(Oc2cccc3C(=NNC(=O)c23)Cc4ccc(F)c(c4)C(=O)N5CCN(CC5)C(=O)C6CC6)ccc7c8NOC(=O)c8cnc17 | Olaparib 2727 |
| Fc1ccc(CC2=NNC(=O)c3c(cccc23)C(=O)NCc4ccnc(n4)c5nccc6ccccc56)cc1C(=O)N7CCN(CC7)C(=O)C8CC8 | Olaparib 262 |
| Cc1noc(CN2Cc3ccc(Oc4cccc5C(=NNC(=O)c45)Cc6ccc(F)c(c6)C(=O)N7CCN(CC7)C(=O)C8CC8)cc3C2=N)n1 | Olaparib 2716 |
| Fc1ccc(CC2=NNC(=O)c3c(Oc4ccc5O[C@@H](CC(=O)c5c4)c6ccccc6)cccc23)cc1C(=O)N7CCN(CC7)C(=O)C8CC8 | Olaparib 2690 |
| FC(=C(F)C(=O)Oc1cccc2C(=NNC(=O)c12)Cc3ccc(F)c(c3)C(=O)N4CCN(CC4)C(=O)C5CC5)F | Olaparib 1314 |
| Oc1cc(Oc2cccc3C(=NNC(=O)c23)Cc4ccc(F)c(c4)C(=O)N5CCN(CC5)C(=O)C6CC6)ccc1c7onc(n7)c8ccsc8 | Olaparib 2627 |
| N[C@H](C(=O)O)C(F)(F)Oc1cccc2C(=NNC(=O)c12)Cc3ccc(F)c(c3)C(=O)N4CCN(CC4)C(=O)C5CC5 | Olaparib 2153 |
| COc1c(C=O)cccc1c2cccc3C(=NNC(=O)c23)Cc4ccc(F)c(c4)C(=O)N5CCN(CC5)C(=O)C6CC6 | Olaparib 1971 |
| CC(C)S(=O)(=O)N1CCC[C@@H](C)[C@@H]1CNC(=O)c2cccc3C(=NNC(=O)c23)Cc4ccc(F)c(c4)C(=O)N5CCN(CC5)C(=O)C6CC6 | Olaparib 131 |
| C[C@H]1COCCN1[C@]2(CNC(=O)c3cccc4C(=NNC(=O)c34)Cc5ccc(F)c(c5)C(=O)N6CCN(CC6)C(=O)C7CC7)CCCOC2 | Olaparib 229 |
| Fc1ccc(CC2=NNC(=O)c3c(cccc23)C(=O)NCC\C=C\c4ccnc5ccccc45)cc1C(=O)N6CCN(CC6)C(=O)C7CC7 | Olaparib 241 |
| Nc1cc(Oc2cccc3C(=NNC(=O)c23)Cc4ccc(F)c(c4)C(=O)N5CCN(CC5)C(=O)C6CC6)c(F)c(c1)C7(CCC7)C#N | Olaparib 2590 |
| Fc1ccc(CC2=NNC(=O)c3c2cccc3C(F)(F)c4cccc(c4)[C@@H]5CNC[C@@H](O5)C6CC6)cc1C(=O)N7CCN(CC7)C(=O)C8CC8 | Olaparib 1783 |
| Fc1ccc(CC2=NNC(=O)c3c(OC(=O)CN4CCCn5c4nc6ccccc56)cccc23)cc1C(=O)N7CCN(CC7)C(=O)C8CC8 | Olaparib 1591 |
| C[C@H](OC(=O)c1cccc2C(=NNC(=O)c12)Cc3ccc(F)c(c3)C(=O)N4CCN(CC4)C(=O)C5CC5)c6onc(n6)c7ccc8ccccc8c7 | Olaparib 1140 |
| Fc1ccc(CC2=NNC(=O)c3c(cccc23)C(=O)NC(=N)c4cccc(n4)N5CCO[C@@H]6CCC[C@@H]56)cc1C(=O)N7CCN(CC7)C(=O)C8CC8 | Olaparib 251 |
| Fc1ccc(cc1F)N2Cc3ccc(Oc4cccc5C(=NNC(=O)c45)Cc6ccc(F)c(c6)C(=O)N7CCN(CC7)C(=O)C8CC8)cc3C2=N | Olaparib 2675 |
| CC(C)(CC(F)(F)F)C(=O)Nc1cccc2C(=NNC(=O)c12)Cc3ccc(F)c(c3)C(=O)N4CCN(CC4)C(=O)C5CC5 | Olaparib 280 |
| Nc1ccc2[nH]cc(C(=O)N3CC[C@H](C3)OC(=O)c4cccc5C(=NNC(=O)c45)Cc6ccc(F)c(c6)C(=O)N7CCN(CC7)C(=O)C8CC8)c2c1 | Olaparib 1129 |
| C[C@H](NC(=O)Nc1cc(C)ccc1F)C(=O)Oc2cccc3C(=NNC(=O)c23)Cc4ccc(F)c(c4)C(=O)N5CCN(CC5)C(=O)C6CC6 | Olaparib 1411 |
| OC(=O)c1cc(Br)c(F)c(c1F)c2cccc3C(=NNC(=O)c23)Cc4ccc(F)c(c4)C(=O)N5CCN(CC5)C(=O)C6CC6 | Olaparib 1650 |
| Fc1ccc(CC2=NNC(=O)c3c2cccc3c4c(Cl)nncc4OC5CCCC5)cc1C(=O)N6CCN(CC6)C(=O)C7CC7 | Olaparib 1751 |
| Cc1ccc(cc1)c2nc(N)nc(n2)c3cccc4C(=NNC(=O)c34)Cc5ccc(F)c(c5)C(=O)N6CCN(CC6)C(=O)C7CC7 | Olaparib 1755 |
| O[C@@H](C(=O)Oc1cccc2C(=NNC(=O)c12)Cc3ccc(F)c(c3)C(=O)N4CCN(CC4)C(=O)C5CC5)c6ccc(cc6)c7ccccc7 | Olaparib 1541 |
| Fc1ccc(CC2=NNC(=O)c3c(Oc4cc5[C@@H]6CC[C@@H](N6)c5cc4Cl)cccc23)cc1C(=O)N7CCN(CC7)C(=O)C8CC8 | Olaparib 2743 |
| Nc1ccc2nc(sc2c1)N3CCC[C@H](C3)Oc4cccc5C(=NNC(=O)c45)Cc6ccc(F)c(c6)C(=O)N7CCN(CC7)C(=O)C8CC8 | Olaparib 3147 |
| Fc1ccc(CC2=NNC(=O)c3c(OC4=CC(=O)NC(=N4)c5nccc6ccccc56)cccc23)cc1C(=O)N7CCN(CC7)C(=O)C8CC8 | Olaparib 3171 |
| Fc1ccc(CC2=NNC(=O)c3c(cccc23)C(=O)OC[C@H]4CN(C(=O)C4)c5nc(cs5)C6CC6)cc1C(=O)N7CCN(CC7)C(=O)C8CC8 | Olaparib 1143 |
| Fc1ccc(CC2=NNC(=O)c3c(Oc4ccc5C(=O)c6ncccc6CCc5c4)cccc23)cc1C(=O)N7CCN(CC7)C(=O)C8CC8 | Olaparib 2720 |
| CCC[C@H](NC(=O)c1cccc2C(=NNC(=O)c12)Cc3ccc(F)c(c3)C(=O)N4CCN(CC4)C(=O)C5CC5)C(F)(F)F | Olaparib 10 |
| Cc1cc(ccc1Oc2cccc3C(=NNC(=O)c23)Cc4ccc(F)c(c4)C(=O)N5CCN(CC5)C(=O)C6CC6)C(=O)Nc7oc(nn7)C8CC8 | Olaparib 2678 |
| COc1nc(NC(C)(C)C#C)nc(n1)c2cccc3C(=NNC(=O)c23)Cc4ccc(F)c(c4)C(=O)N5CCN(CC5)C(=O)C6CC6 | Olaparib 1666 |
| OC(=O)c1cccc(c1F)c2cccc3C(=NNC(=O)c23)Cc4ccc(F)c(c4)C(=O)N5CCN(CC5)C(=O)C6CC6 | Olaparib 1969 |
| CC1(C)OCc2c(nc(Cl)nc12)c3cccc4C(=NNC(=O)c34)Cc5ccc(F)c(c5)C(=O)N6CCN(CC6)C(=O)C7CC7 | Olaparib 1757 |
| Fc1ccc(cc1F)c2cc(CNC(=O)c3cccc4C(=NNC(=O)c34)Cc5ccc(F)c(c5)C(=O)N6CCN(CC6)C(=O)C7CC7)on2 | Olaparib 232 |
| COc1ccc2nc(Cl)c(C=O)c(c3cccc4C(=NNC(=O)c34)Cc5ccc(F)c(c5)C(=O)N6CCN(CC6)C(=O)C7CC7)c2c1 | Olaparib 1744 |
| Fc1ccc(CC2=NNC(=O)c3c2cccc3c4ccnc5[C@H]6C[C@H](C=C6)c45)cc1C(=O)N7CCN(CC7)C(=O)C8CC8 | Olaparib 1791 |
| Fc1ccc(CC2=NNC(=O)c3c(OC(=O)Cc4csc5nc(cn45)C6CC6)cccc23)cc1C(=O)N7CCN(CC7)C(=O)C8CC8 | Olaparib 1597 |
| OC1(C(=O)Oc2cccc3C(=NNC(=O)c23)Cc4ccc(F)c(c4)C(=O)N5CCN(CC5)C(=O)C6CC6)c7ccccc7c8ccccc18 | Olaparib 1593 |
| CCc1cccc2c1OCc3c(n[nH]c23)C(=O)Oc4cccc5C(=NNC(=O)c45)Cc6ccc(F)c(c6)C(=O)N7CCN(CC7)C(=O)C8CC8 | Olaparib 1580 |
| Fc1ccc(CC2=NNC(=O)c3c(OC(=O)Cc4onc(n4)[C@H]5C[C@H]6CC[C@@H]5O6)cccc23)cc1C(=O)N7CCN(CC7)C(=O)C8CC8 | Olaparib 1596 |
| C[C@]1(C[C@@H](CCO1)OC(=O)c2cccc3C(=NNC(=O)c23)Cc4ccc(F)c(c4)C(=O)N5CCN(CC5)C(=O)C6CC6)c7ccc8ccccc8c7 | Olaparib 1136 |
| C[C@H](CC(=O)Nc1cccc2C(=NNC(=O)c12)Cc3ccc(F)c(c3)C(=O)N4CCN(CC4)C(=O)C5CC5)C(F)(F)F | Olaparib 290 |
| Cc1cc(N2CC(CC(=O)Nc3cccc4C(=NNC(=O)c34)Cc5ccc(F)c(c5)C(=O)N6CCN(CC6)C(=O)C7CC7)C2)n8ncnc8c1 | Olaparib 549 |
| OC(=O)C1=CC=CN(CC(F)(F)Oc2cccc3C(=NNC(=O)c23)Cc4ccc(F)c(c4)C(=O)N5CCN(CC5)C(=O)C6CC6)C1=O | Olaparib 2256 |
| N[C@@H]1CC[C@](C1)(Oc2cccc3C(=NNC(=O)c23)Cc4ccc(F)c(c4)C(=O)N5CCN(CC5)C(=O)C6CC6)C(F)(F)F | Olaparib 2920 |
| C[C@@]12CC[C@@](CC(=O)Oc3cccc4C(=NNC(=O)c34)Cc5ccc(F)c(c5)C(=O)N6CCN(CC6)C(=O)C7CC7)(C[C@H]1O)C2(C)C | Olaparib 1554 |
| Cc1cnc2c(nc(Cl)nc2n1)c3cccc4C(=NNC(=O)c34)Cc5ccc(F)c(c5)C(=O)N6CCN(CC6)C(=O)C7CC7 | Olaparib 1759 |
| Fc1ccc(CC2=NNC(=O)c3c(cccc23)C(=O)NCC#Cc4cncc5ccccc45)cc1C(=O)N6CCN(CC6)C(=O)C7CC7 | Olaparib 243 |
| Fc1ccc(CC2=NNC(=O)c3c2cccc3c4cc(CN5C[C@H]6CC[C@@H]5C6)ccc4F)cc1C(=O)N7CCN(CC7)C(=O)C8CC8 | Olaparib 2084 |
| OC1C[C@H]2CC[C@@H](C1)N2c3ccnc(CNC(=O)c4cccc5C(=NNC(=O)c45)Cc6ccc(F)c(c6)C(=O)N7CCN(CC7)C(=O)C8CC8)c3 | Olaparib 263 |
| Fc1ccc(CC2=NNC(=O)c3c(cccc23)C(=O)NCc4onc(c4)c5ccc6OCCc6c5)cc1C(=O)N7CCN(CC7)C(=O)C8CC8 | Olaparib 268 |
| NC1(CC1)c2ccc3ccccc3c2OC(=O)c4cccc5C(=NNC(=O)c45)Cc6ccc(F)c(c6)C(=O)N7CCN(CC7)C(=O)C8CC8 | Olaparib 1168 |
| O[C@@H]1C[C@@H]2N(C1)C(=O)[C@@H]3C[C@H](CN3C2=O)Oc4cccc5C(=NNC(=O)c45)Cc6ccc(F)c(c6)C(=O)N7CCN(CC7)C(=O)C8CC8 | Olaparib 3190 |
| N[C@H]1CN(Cc2cccc(Oc3cccc4C(=NNC(=O)c34)Cc5ccc(F)c(c5)C(=O)N6CCN(CC6)C(=O)C7CC7)c2)c8ccccc8C1 | Olaparib 2645 |
| Fc1ccc(CC2=NNC(=O)c3c(cccc23)C(=O)NCCC(F)(F)F)cc1C(=O)N4CCN(CC4)C(=O)C5CC5 | Olaparib 82 |
| Cc1cc(N2CC(CC(=O)Oc3cccc4C(=NNC(=O)c34)Cc5ccc(F)c(c5)C(=O)N6CCN(CC6)C(=O)C7CC7)C2)n8ncnc8c1 | Olaparib 1578 |
| CC(=O)OCN1N=CC(=C(C1=O)c2cccc3C(=NNC(=O)c23)Cc4ccc(F)c(c4)C(=O)N5CCN(CC5)C(=O)C6CC6)Cl | Olaparib 1658 |
| COc1c(F)c(F)c(Cc2cccc3C(=NNC(=O)c23)Cc4ccc(F)c(c4)C(=O)N5CCN(CC5)C(=O)C6CC6)c(F)c1F | Olaparib 1854 |
| Fc1ccc(CC2=NNC(=O)c3c2cccc3c4ccnc5ncccc45)cc1C(=O)N6CCN(CC6)C(=O)C7CC7 | Olaparib 2077 |
| Fc1ccc(CC2=NNC(=O)c3c(OC(=O)COc4cccc5ccccc45)cccc23)cc1C(=O)N6CCN(CC6)C(=O)C7CC7 | Olaparib 1563 |
| CC(=O)c1sc2ccccc2c1OC(=O)c3cccc4C(=NNC(=O)c34)Cc5ccc(F)c(c5)C(=O)N6CCN(CC6)C(=O)C7CC7 | Olaparib 1065 |
| Fc1ccc(CC2=NNC(=O)c3c(O[C@H]4CCc5c(OCc6ccccn6)cccc45)cccc23)cc1C(=O)N7CCN(CC7)C(=O)C8CC8 | Olaparib 3167 |
| Fc1ccc(CC2=NNC(=O)c3c(OC(=O)c4cnc5occc5c4)cccc23)cc1C(=O)N6CCN(CC6)C(=O)C7CC7 | Olaparib 1572 |
| Fc1ccc(CC2=NNC(=O)c3c(NC(=O)Cc4csc(n4)[C@H]5C[C@H]6CC[C@@H]5O6)cccc23)cc1C(=O)N7CCN(CC7)C(=O)C8CC8 | Olaparib 552 |
| Fc1ccc(CC2=NNC(=O)c3c(OC(=O)Cc4nc(c5occc5)n6ccccc46)cccc23)cc1C(=O)N7CCN(CC7)C(=O)C8CC8 | Olaparib 1582 |
| Fc1ccc(CC2=NNC(=O)c3c(NC(=O)CN4CCCn5c4nc6ccccc56)cccc23)cc1C(=O)N7CCN(CC7)C(=O)C8CC8 | Olaparib 553 |
| NC1(CC1)c2ccc(OCc3ccc(Oc4cccc5C(=NNC(=O)c45)Cc6ccc(F)c(c6)C(=O)N7CCN(CC7)C(=O)C8CC8)cc3)cc2 | Olaparib 2698 |
| Fc1ccc(CC2=NNC(=O)c3c(NC(=O)CCC(F)(F)F)cccc23)cc1C(=O)N4CCN(CC4)C(=O)C5CC5 | Olaparib 322 |
| Fc1ccc(CC2=NNC(=O)c3c(O[C@@H]4CC[C@H](CC4)c5onc(n5)c6cnccn6)cccc23)cc1C(=O)N7CCN(CC7)C(=O)C8CC8 | Olaparib 3156 |
| CC(=O)N1CCN(C[C@@H](Oc2cccc3C(=NNC(=O)c23)Cc4ccc(F)c(c4)C(=O)N5CCN(CC5)C(=O)C6CC6)C(F)(F)F)CC1 | Olaparib 2855 |
| CC1(C)CCC(=O)C(=C1)c2cccc3C(=NNC(=O)c23)Cc4ccc(F)c(c4)C(=O)N5CCN(CC5)C(=O)C6CC6 | Olaparib 1983 |
| Fc1ccc(CC2=NNC(=O)c3c2cccc3c4cncc5cnccc45)cc1C(=O)N6CCN(CC6)C(=O)C7CC7 | Olaparib 2078 |
| Fc1ccc(CC2=NNC(=O)c3c(cccc23)C(=O)NCc4[nH]c5ccccc5c4Cl)cc1C(=O)N6CCN(CC6)C(=O)C7CC7 | Olaparib 245 |
| Cc1ccc(OCc2ccc(Oc3cccc4C(=NNC(=O)c34)Cc5ccc(F)c(c5)C(=O)N6CCN(CC6)C(=O)C7CC7)s2)c(CN)c1 | Olaparib 2465 |
| Fc1ccc(CC2=NNC(=O)c3c(O[C@H]4CN(C(=O)C4)c5ccc6ccccc6c5)cccc23)cc1C(=O)N7CCN(CC7)C(=O)C8CC8 | Olaparib 3186 |
| CC(C)n1cnc2c(ncnc12)c3cccc4C(=NNC(=O)c34)Cc5ccc(F)c(c5)C(=O)N6CCN(CC6)C(=O)C7CC7 | Olaparib 1769 |
| Fc1ccc(CC2=NNC(=O)c3c(OC(=O)CO\N=C/4\C[N@@]5CC[C@H]4CC5)cccc23)cc1C(=O)N6CCN(CC6)C(=O)C7CC7 | Olaparib 1565 |
| Fc1ccc(CC2=NNC(=O)c3c(OC(=O)c4ccoc4Cn5cnc6ccccc56)cccc23)cc1C(=O)N7CCN(CC7)C(=O)C8CC8 | Olaparib 1583 |
| Fc1ccc(CC2=NNC(=O)c3c(Oc4cncc(c4)c5onc(n5)C6CCCC6)cccc23)cc1C(=O)N7CCN(CC7)C(=O)C8CC8 | Olaparib 3180 |
| Cc1ccc(cc1Oc2cccc3C(=NNC(=O)c23)Cc4ccc(F)c(c4)C(=O)N5CCN(CC5)C(=O)C6CC6)[C@@H](O)c7ccc8COCc8c7 | Olaparib 2639 |
| Fc1ccc(CC2=NNC(=O)c3c(OC(=O)CCC(F)(F)F)cccc23)cc1C(=O)N4CCN(CC4)C(=O)C5CC5 | Olaparib 1246 |
| Cn1ccnc1[C@@H](O)c2cc(Oc3cccc4C(=NNC(=O)c34)Cc5ccc(F)c(c5)C(=O)N6CCN(CC6)C(=O)C7CC7)c8OCOc8c2 | Olaparib 2658 |
| Fc1ccc(CC2=NNC(=O)c3c(Oc4cccc5nncn45)cccc23)cc1C(=O)N6CCN(CC6)C(=O)C7CC7 | Olaparib 2621 |
| Fc1ccc(CC2=NNC(=O)c3c(O[C@@]4(F)CC(Cl)(Cl)[C@]4(F)Cl)cccc23)cc1C(=O)N5CCN(CC5)C(=O)C6CC6 | Olaparib 2272 |
| Fc1ccc(CC2=NNC(=O)c3c2cccc3c4cncc5ncccc45)cc1C(=O)N6CCN(CC6)C(=O)C7CC7 | Olaparib 2079 |
| Fc1ccc(CC2=NNC(=O)c3c2cccc3C(F)(F)c4ccc(Cc5nnc(Cl)s5)cc4)cc1C(=O)N6CCN(CC6)C(=O)C7CC7 | Olaparib 1733 |
| CN[C@@H](C(=O)Oc1cccc2C(=NNC(=O)c12)Cc3ccc(F)c(c3)C(=O)N4CCN(CC4)C(=O)C5CC5)c6csc7ccccc67 | Olaparib 1547 |
| Fc1ccc(CC2=NNC(=O)c3c(Oc4ccc5ccc(OC6CCOCC6)cc5c4)cccc23)cc1C(=O)N7CCN(CC7)C(=O)C8CC8 | Olaparib 3159 |
| Fc1ccc(CC2=NNC(=O)c3c(Oc4ccc(NCc5cnn6ccccc56)cc4F)cccc23)cc1C(=O)N7CCN(CC7)C(=O)C8CC8 | Olaparib 2689 |
| Fc1ccc(CC2=NNC(=O)c3c(OC(=O)Cn4nnc5cc6CCCCc6cc45)cccc23)cc1C(=O)N7CCN(CC7)C(=O)C8CC8 | Olaparib 1590 |
| Fc1ccc(CC2=NNC(=O)c3c(Oc4ccc(cc4)c5n[nH]c(n5)c6cccs6)cccc23)cc1C(=O)N7CCN(CC7)C(=O)C8CC8 | Olaparib 3161 |
| CC1(C)CC(=O)c2c(C1)[nH]c3ccc(Oc4cccc5C(=NNC(=O)c45)Cc6ccc(F)c(c6)C(=O)N7CCN(CC7)C(=O)C8CC8)cc23 | Olaparib 2731 |
| F[C@H](C(=O)Nc1cccc2C(=NNC(=O)c12)Cc3ccc(F)c(c3)C(=O)N4CCN(CC4)C(=O)C5CC5)C(F)(F)F | Olaparib 304 |
| Fc1ccc(CC2=NNC(=O)c3c(NC(=O)CC4CN(C4)c5ncnc6ccsc56)cccc23)cc1C(=O)N7CCN(CC7)C(=O)C8CC8 | Olaparib 547 |
| Fc1ccc(CC2=NNC(=O)c3c(Cc4cccc(c4F)C(F)(F)F)cccc23)cc1C(=O)N5CCN(CC5)C(=O)C6CC6 | Olaparib 1856 |
| NC1(CCC1)c2c(F)c(F)c(Oc3cccc4C(=NNC(=O)c34)Cc5ccc(F)c(c5)C(=O)N6CCN(CC6)C(=O)C7CC7)c(F)c2F | Olaparib 3067 |
| Fc1ccc(CC2=NNC(=O)c3c2cccc3[C@]4(F)CC[C@]4(F)Cl)cc1C(=O)N5CCN(CC5)C(=O)C6CC6 | Olaparib 1727 |
| CC(=O)C1=Cc2cc(Oc3cccc4C(=NNC(=O)c34)Cc5ccc(F)c(c5)C(=O)N6CCN(CC6)C(=O)C7CC7)cc(Cl)c2OC1=O | Olaparib 2486 |
| CN1CCC(CC1)c2c[nH]c3ccc(Oc4cccc5C(=NNC(=O)c45)Cc6ccc(F)c(c6)C(=O)N7CCN(CC7)C(=O)C8CC8)cc23 | Olaparib 3181 |
| Fc1ccc(CC2=NNC(=O)c3c(OC(=O)Cc4c[nH]c5cc(Cl)cc(F)c45)cccc23)cc1C(=O)N6CCN(CC6)C(=O)C7CC7 | Olaparib 1542 |
| Fc1ccc(CC2=NNC(=O)c3c2cccc3C(F)(F)c4cccc(OC[C@H]5CNC(=S)O5)c4)cc1C(=O)N6CCN(CC6)C(=O)C7CC7 | Olaparib 1735 |
| Fc1ccc(NCc2cnn3ccccc23)cc1Oc4cccc5C(=NNC(=O)c45)Cc6ccc(F)c(c6)C(=O)N7CCN(CC7)C(=O)C8CC8 | Olaparib 2688 |
| Fc1ccc(CC2=NNC(=O)c3c(Oc4ccc(cc4)c5nnc6ccc(Cl)nn56)cccc23)cc1C(=O)N7CCN(CC7)C(=O)C8CC8 | Olaparib 2666 |
| C[C@@H]1CCc2c(C1)sc3ncc(C(=O)Oc4cccc5C(=NNC(=O)c45)Cc6ccc(F)c(c6)C(=O)N7CCN(CC7)C(=O)C8CC8)n23 | Olaparib 1587 |
| N[C@H]1C[C@@H]1c2oc(cc2)c3ccc(Oc4cccc5C(=NNC(=O)c45)Cc6ccc(F)c(c6)C(=O)N7CCN(CC7)C(=O)C8CC8)cc3F | Olaparib 2726 |
| FC(F)OC(F)(F)[C@@H](F)Oc1cccc2C(=NNC(=O)c12)Cc3ccc(F)c(c3)C(=O)N4CCN(CC4)C(=O)C5CC5 | Olaparib 2136 |
| Fc1ccc(CC2=NNC(=O)c3c(OC(=O)CC4CN(C4)c5ncnc6ccsc56)cccc23)cc1C(=O)N7CCN(CC7)C(=O)C8CC8 | Olaparib 1575 |
| COC(=O)[C@H](N)CNC(=O)c1cccc2C(=NNC(=O)c12)Cc3ccc(F)c(c3)C(=O)N4CCN(CC4)C(=O)C5CC5 | Olaparib 54 |
| Fc1ccc(CC2=NNC(=O)c3c2cccc3c4occ(c4)C5CCCC5)cc1C(=O)N6CCN(CC6)C(=O)C7CC7 | Olaparib 2075 |
| COC(=O)C(C)(C)CC(=C)Oc1cccc2C(=NNC(=O)c12)Cc3ccc(F)c(c3)C(=O)N4CCN(CC4)C(=O)C5CC5 | Olaparib 2126 |
| C[C@@](N)(CN1CCc2ccccc2CC1)C(=O)Oc3cccc4C(=NNC(=O)c34)Cc5ccc(F)c(c5)C(=O)N6CCN(CC6)C(=O)C7CC7 | Olaparib 1526 |
| CN1CCC(CC1)c2c[nH]c3c(Oc4cccc5C(=NNC(=O)c45)Cc6ccc(F)c(c6)C(=O)N7CCN(CC7)C(=O)C8CC8)cccc23 | Olaparib 2709 |
| Fc1ccc(CC2=NNC(=O)c3c2cccc3c4c[nH]nc4c5ccccn5)cc1C(=O)N6CCN(CC6)C(=O)C7CC7 | Olaparib 2073 |
| CC(C)(NC(=O)c1cccc2C(=NNC(=O)c12)Cc3ccc(F)c(c3)C(=O)N4CCN(CC4)C(=O)C5CC5)c6ccc7ccccc7c6O | Olaparib 240 |
| Fc1ccc(CC2=NNC(=O)c3c(OC(=O)CCc4ncc5ccc6ccccc6n45)cccc23)cc1C(=O)N7CCN(CC7)C(=O)C8CC8 | Olaparib 1584 |
| Oc1c(ccc2ccccc12)C(=O)N3CC(C3)OC(=O)c4cccc5C(=NNC(=O)c45)Cc6ccc(F)c(c6)C(=O)N7CCN(CC7)C(=O)C8CC8 | Olaparib 1133 |
| Fc1ccc(CC2=NNC(=O)c3c(cccc23)C(=O)NC(=S)c4cc(N[C@H]5C[C@H]6CC[C@@H]5C6)ccn4)cc1C(=O)N7CCN(CC7)C(=O)C8CC8 | Olaparib 250 |
| Fc1ccc(CC2=NNC(=O)c3c(O[C@@H]4CCO[C@@H](C4)c5ccc6ncccc6c5)cccc23)cc1C(=O)N7CCN(CC7)C(=O)C8CC8 | Olaparib 3182 |
| CC(C)(Oc1cccc2C(=NNC(=O)c12)Cc3ccc(F)c(c3)C(=O)N4CCN(CC4)C(=O)C5CC5)C(F)(F)F | Olaparib 2775 |
| Fc1ccc(CC2=NNC(=O)c3c(cccc23)C(=O)O[C@@H]4C[C@@H]5N(C4)C(=O)c6ccccc6NC5=O)cc1C(=O)N7CCN(CC7)C(=O)C8CC8 | Olaparib 1149 |
| OC(=O)C(F)(F)C(=O)Oc1cccc2C(=NNC(=O)c12)Cc3ccc(F)c(c3)C(=O)N4CCN(CC4)C(=O)C5CC5 | Olaparib 1249 |
| Fc1cc(F)c(CSC(=N)NC(=O)c2cccc3C(=NNC(=O)c23)Cc4ccc(F)c(c4)C(=O)N5CCN(CC5)C(=O)C6CC6)cc1F | Olaparib 134 |
| O[C@H](CC(=O)Oc1cccc2C(=NNC(=O)c12)Cc3ccc(F)c(c3)C(=O)N4CCN(CC4)C(=O)C5CC5)C(F)(F)F | Olaparib 1202 |
| Oc1c(Oc2cccc3C(=NNC(=O)c23)Cc4ccc(F)c(c4)C(=O)N5CCN(CC5)C(=O)C6CC6)cc(Cl)c7cccnc17 | Olaparib 2483 |
| C[C@@H](CC(=O)Oc1cccc2C(=NNC(=O)c12)Cc3ccc(F)c(c3)C(=O)N4CCN(CC4)C(=O)C5CC5)N6CCN(C)c7ccccc67 | Olaparib 1533 |
| Fc1ccc(CC2=NNC(=O)c3c(O[C@H]4CN(C(=O)C4)c5ccc6CCCc6c5)cccc23)cc1C(=O)N7CCN(CC7)C(=O)C8CC8 | Olaparib 3194 |
| N[C@@H](Cc1cccc(c1)C(F)(F)c2cccc3C(=NNC(=O)c23)Cc4ccc(F)c(c4)C(=O)N5CCN(CC5)C(=O)C6CC6)c7ccncn7 | Olaparib 1739 |
| Oc1ccc2c(c1)[nH]c3cc(Oc4cccc5C(=NNC(=O)c45)Cc6ccc(F)c(c6)C(=O)N7CCN(CC7)C(=O)C8CC8)c(C=O)cc23 | Olaparib 3188 |
| Fc1ccc(CC2=NNC(=O)c3c(cccc23)C(=O)O[C@H]4N(C(=O)c5ccccc45)n6cccc6)cc1C(=O)N7CCN(CC7)C(=O)C8CC8 | Olaparib 1161 |
| Fc1ccc(CC2=NNC(=O)c3c(cccc23)C(=O)NCc4ccc(cc4)c5nc6ccccc6[nH]5)cc1C(=O)N7CCN(CC7)C(=O)C8CC8 | Olaparib 266 |
| Fc1ccc(CC2=NNC(=O)c3c(Oc4ccc5c(c[nH]c5c4)C6=CCNCC6)cccc23)cc1C(=O)N7CCN(CC7)C(=O)C8CC8 | Olaparib 2740 |
| Fc1ccc(CC2=NNC(=O)c3c(O[C@](F)(I)C(F)(F)F)cccc23)cc1C(=O)N4CCN(CC4)C(=O)C5CC5 | Olaparib 2099 |
| Fc1ccc(CC2=NNC(=O)c3c(Oc4ccc(cc4)N5CC(=NC5=O)N6CCOCC6)cccc23)cc1C(=O)N7CCN(CC7)C(=O)C8CC8 | Olaparib 2624 |
| Oc1c(F)c(Oc2cccc3C(=NNC(=O)c23)Cc4ccc(F)c(c4)C(=O)N5CCN(CC5)C(=O)C6CC6)ccc1[C@@H]7CCC(=O)N7 | Olaparib 2581 |
| N[C@H](CF)C(=O)Nc1cccc2C(=NNC(=O)c12)Cc3ccc(F)c(c3)C(=O)N4CCN(CC4)C(=O)C5CC5 | Olaparib 428 |
| CSC[C@H](C)Cn1cnnc1c2cccc3C(=NNC(=O)c23)Cc4ccc(F)c(c4)C(=O)N5CCN(CC5)C(=O)C6CC6 | Olaparib 1679 |
| Oc1cncc2cc(oc12)C(F)(F)c3cccc4C(=NNC(=O)c34)Cc5ccc(F)c(c5)C(=O)N6CCN(CC6)C(=O)C7CC7 | Olaparib 1767 |
| O[C@@H](CN1[C@@H]2CC[C@H]1CC(C2)Oc3cccc4C(=NNC(=O)c34)Cc5ccc(F)c(c5)C(=O)N6CCN(CC6)C(=O)C7CC7)c8ccccc8 | Olaparib 3152 |
| Fc1ccc(CC2=NNC(=O)c3c(cccc23)C(=O)NCc4ccc(s4)c5ccccc5)cc1C(=O)N6CCN(CC6)C(=O)C7CC7 | Olaparib 242 |
| Fc1ccc(CC2=NNC(=O)c3c(Oc4ccc5nc(cn5n4)c6ccccc6)cccc23)cc1C(=O)N7CCN(CC7)C(=O)C8CC8 | Olaparib 2734 |
| OC1=C(Oc2ccc(Oc3cccc4C(=NNC(=O)c34)Cc5ccc(F)c(c5)C(=O)N6CCN(CC6)C(=O)C7CC7)cc2C1=O)c8occc8 | Olaparib 2672 |
| Fc1ccc(CC2=NNC(=O)c3c(Oc4ccc(NCc5cnn6ccccc56)c(Cl)c4)cccc23)cc1C(=O)N7CCN(CC7)C(=O)C8CC8 | Olaparib 2638 |
| CC1=NCCc2c1[nH]c3cc(Oc4cccc5C(=NNC(=O)c45)Cc6ccc(F)c(c6)C(=O)N7CCN(CC7)C(=O)C8CC8)ccc23 | Olaparib 3200 |
| C[C@@H](Oc1cccc2C(=NNC(=O)c12)Cc3ccc(F)c(c3)C(=O)N4CCN(CC4)C(=O)C5CC5)[C@H](O)CO | Olaparib 2829 |
| NCc1cnc(cn1)N2[C@@H]3CC[C@H]2CC(C3)OC(=O)c4cccc5C(=NNC(=O)c45)Cc6ccc(F)c(c6)C(=O)N7CCN(CC7)C(=O)C8CC8 | Olaparib 1147 |
| Oc1c(F)c(Oc2cccc3C(=NNC(=O)c23)Cc4ccc(F)c(c4)C(=O)N5CCN(CC5)C(=O)C6CC6)ccc1[C@@H]7CCC(=O)N7 | Olaparib 2579 |
| COc1ccccc1\C=C\CNC(=O)c2cccc3C(=NNC(=O)c23)Cc4ccc(F)c(c4)C(=O)N5CCN(CC5)C(=O)C6CC6 | Olaparib 173 |
| Fc1ccc(CC2=NNC(=O)c3c(cccc23)C(=O)OC[C@H]4CCCN(Cc5ccccc5)C4)cc1C(=O)N6CCN(CC6)C(=O)C7CC7 | Olaparib 1049 |
| Fc1ccc(CC2=NNC(=O)c3c(OC4C[C@H]5CC[C@@H](C4)N5c6ccc(cc6)C#N)cccc23)cc1C(=O)N7CCN(CC7)C(=O)C8CC8 | Olaparib 3183 |
| O[C@H](C(=O)Oc1cccc2C(=NNC(=O)c12)Cc3ccc(F)c(c3)C(=O)N4CCN(CC4)C(=O)C5CC5)c6ccc7ccccc7c6 | Olaparib 1562 |
| Fc1ccc(CC2=NNC(=O)c3c(Oc4cccc(c4Cl)c5nc6ccncc6[nH]5)cccc23)cc1C(=O)N7CCN(CC7)C(=O)C8CC8 | Olaparib 2668 |
| OC(=O)c1cnn(Cc2ccc(Oc3cccc4C(=NNC(=O)c34)Cc5ccc(F)c(c5)C(=O)N6CCN(CC6)C(=O)C7CC7)cc2)c1C8CC8 | Olaparib 2682 |
| Cn1c(nc2cc(F)ccc12)c3cncc(Oc4cccc5C(=NNC(=O)c45)Cc6ccc(F)c(c6)C(=O)N7CCN(CC7)C(=O)C8CC8)c3 | Olaparib 3163 |
| Fc1ccc(CC2=NNC(=O)c3c(Oc4ccc5c(c[nH]c5c4)C6CCNCC6)cccc23)cc1C(=O)N7CCN(CC7)C(=O)C8CC8 | Olaparib 2738 |
| CC(C)C[C@@](C)(NC(=O)c1cccc2C(=NNC(=O)c12)Cc3ccc(F)c(c3)C(=O)N4CCN(CC4)C(=O)C5CC5)C(F)(F)F | Olaparib 1 |
| Fc1ccc(CC2=NNC(=O)c3c(cccc23)C(=O)OC4C[C@H]5CC[C@@H](C4)N5c6nncs6)cc1C(=O)N7CCN(CC7)C(=O)C8CC8 | Olaparib 1163 |
| Fc1ccc(CC2=NNC(=O)c3c(Oc4nncc(OC5CCCC5)c4Cl)cccc23)cc1C(=O)N6CCN(CC6)C(=O)C7CC7 | Olaparib 2543 |
| N[C@@H](Cc1cccc(Cl)c1)C(=O)Oc2cccc3C(=NNC(=O)c23)Cc4ccc(F)c(c4)C(=O)N5CCN(CC5)C(=O)C6CC6 | Olaparib 1455 |
| Fc1ccc(CC2=NNC(=O)c3c2cccc3C(F)(F)c4ccc([C@@H]5CCC(=O)N5)c(c4)C#N)cc1C(=O)N6CCN(CC6)C(=O)C7CC7 | Olaparib 1745 |
| Fc1ccc(CC2=NNC(=O)c3c(Oc4ccc(cn4)[C@@H]5C[C@H]6CC[C@@H]5N6)cccc23)cc1C(=O)N7CCN(CC7)C(=O)C8CC8 | Olaparib 2745 |
| OC(=O)c1ccc(cc1c2cccc3C(=NNC(=O)c23)Cc4ccc(F)c(c4)C(=O)N5CCN(CC5)C(=O)C6CC6)c7cocc7 | Olaparib 1764 |
| Fc1ccc(CC2=NNC(=O)c3c(cccc23)C(=O)NCc4cc(CN5CCc6ccccc56)ccn4)cc1C(=O)N7CCN(CC7)C(=O)C8CC8 | Olaparib 259 |
| Fc1ccc(CC2=NNC(=O)c3c(Oc4cc(ccn4)c5cc6C(=O)NCCc6[nH]5)cccc23)cc1C(=O)N7CCN(CC7)C(=O)C8CC8 | Olaparib 2712 |
| Cc1ccc2nc([nH]c2c1)c3cncc(Oc4cccc5C(=NNC(=O)c45)Cc6ccc(F)c(c6)C(=O)N7CCN(CC7)C(=O)C8CC8)c3 | Olaparib 3191 |
| CN1CCc2[nH]c3ccc(Oc4cccc5C(=NNC(=O)c45)Cc6ccc(F)c(c6)C(=O)N7CCN(CC7)C(=O)C8CC8)cc3c2C1 | Olaparib 2663 |
| CC[C@@H](C)[C@@H](F)C(=O)Nc1cccc2C(=NNC(=O)c12)Cc3ccc(F)c(c3)C(=O)N4CCN(CC4)C(=O)C5CC5 | Olaparib 333 |
| Fc1ccc(CC2=NNC(=O)c3c(cccc23)C(=O)OC4C[C@H]5CC[C@@H](C4)N5C(=O)C6=COCCO6)cc1C(=O)N7CCN(CC7)C(=O)C8CC8 | Olaparib 1142 |
| C[C@H]1CC[C@H](Nc2nsnc2c3cccc4C(=NNC(=O)c34)Cc5ccc(F)c(c5)C(=O)N6CCN(CC6)C(=O)C7CC7)[C@@H](C)C1 | Olaparib 1749 |
| COC(=O)c1c(F)c(F)c(F)c(F)c1Oc2cccc3C(=NNC(=O)c23)Cc4ccc(F)c(c4)C(=O)N5CCN(CC5)C(=O)C6CC6 | Olaparib 2240 |
| OC(=O)C1(CCC1)c2cc(Oc3cccc4C(=NNC(=O)c34)Cc5ccc(F)c(c5)C(=O)N6CCN(CC6)C(=O)C7CC7)c(F)cc2F | Olaparib 2547 |
| C[C@H]1C[C@@H]1c2oc(cc2)[C@H]3C[C@H](CCO3)Oc4cccc5C(=NNC(=O)c45)Cc6ccc(F)c(c6)C(=O)N7CCN(CC7)C(=O)C8CC8 | Olaparib 3192 |
| Fc1ccc(CC2=NNC(=O)c3c(cccc23)C(=O)OCc4cnc5ccccc5c4)cc1C(=O)N6CCN(CC6)C(=O)C7CC7 | Olaparib 1112 |
| Cc1cc2ccccc2nc1N3CCC[C@@H]3CNC(=O)c4cccc5C(=NNC(=O)c45)Cc6ccc(F)c(c6)C(=O)N7CCN(CC7)C(=O)C8CC8 | Olaparib 256 |
| Fc1ccc2OCc3c(n[nH]c3c2c1)C(=O)Oc4cccc5C(=NNC(=O)c45)Cc6ccc(F)c(c6)C(=O)N7CCN(CC7)C(=O)C8CC8 | Olaparib 1588 |
| Cc1cc2cc(ccc2nc1Oc3cccc4C(=NNC(=O)c34)Cc5ccc(F)c(c5)C(=O)N6CCN(CC6)C(=O)C7CC7)c8nccs8 | Olaparib 2679 |
| Fc1ccc(CC2=NNC(=O)c3c(cccc23)C(=O)O[C@@]4(Cc5ccc6CCCc6c5)CCNC4)cc1C(=O)N7CCN(CC7)C(=O)C8CC8 | Olaparib 1159 |
| C[C@@H](CC(F)(F)F)C(=O)Oc1cccc2C(=NNC(=O)c12)Cc3ccc(F)c(c3)C(=O)N4CCN(CC4)C(=O)C5CC5 | Olaparib 1205 |
| O[C@H]1CN[C@H](C1)C(=O)N2[C@@H]3CC[C@H]2CC(C3)OC(=O)c4cccc5C(=NNC(=O)c45)Cc6ccc(F)c(c6)C(=O)N7CCN(CC7)C(=O)C8CC8 | Olaparib 1138 |
| C[C@]1(CC(=O)Oc2cccc3C(=NNC(=O)c23)Cc4ccc(F)c(c4)C(=O)N5CCN(CC5)C(=O)C6CC6)[C@H]7CC[C@]1(C)\C(=N\O)\C7 | Olaparib 1556 |
| COC(=O)c1c(F)c(F)c(F)c(Oc2cccc3C(=NNC(=O)c23)Cc4ccc(F)c(c4)C(=O)N5CCN(CC5)C(=O)C6CC6)c1F | Olaparib 2241 |
| OC(=O)C1(CCC1)c2cc(F)c(Oc3cccc4C(=NNC(=O)c34)Cc5ccc(F)c(c5)C(=O)N6CCN(CC6)C(=O)C7CC7)cc2F | Olaparib 2548 |
| Fc1ccc(CC2=NNC(=O)c3c(OC4C[C@H]5CC[C@@H](C4)N5CCc6ccsc6)cccc23)cc1C(=O)N7CCN(CC7)C(=O)C8CC8 | Olaparib 3173 |
| Fc1ccc(CC2=NNC(=O)c3c(Oc4nccc(n4)c5ccccc5)cccc23)cc1C(=O)N6CCN(CC6)C(=O)C7CC7 | Olaparib 2609 |
| Fc1ccc(CC2=NNC(=O)c3c(Oc4ccc5c(c[nH]c5c4)C(=O)c6occc6)cccc23)cc1C(=O)N7CCN(CC7)C(=O)C8CC8 | Olaparib 2736 |
| CCc1cc(nc(n1)c2ccc3ncccc3c2)c4cccc5C(=NNC(=O)c45)Cc6ccc(F)c(c6)C(=O)N7CCN(CC7)C(=O)C8CC8 | Olaparib 1784 |
| Fc1ccc(CC2=NNC(=O)c3c(OC(=O)c4noc5c4CSc6ccccc56)cccc23)cc1C(=O)N7CCN(CC7)C(=O)C8CC8 | Olaparib 1589 |
| Fc1ccc(CC2=NNC(=O)c3c(O[C@@H]4CN([C@@H]5CCc6ccccc6C5)C(=O)C4)cccc23)cc1C(=O)N7CCN(CC7)C(=O)C8CC8 | Olaparib 3179 |
| C[C@H](CC(=O)Oc1cccc2C(=NNC(=O)c12)Cc3ccc(F)c(c3)C(=O)N4CCN(CC4)C(=O)C5CC5)C(F)(F)F | Olaparib 1204 |
| CC1(C)O[C@H]2O[C@H](CN3CCCC3)[C@H](OC(=O)c4cccc5C(=NNC(=O)c45)Cc6ccc(F)c(c6)C(=O)N7CCN(CC7)C(=O)C8CC8)[C@H]2O1 | Olaparib 1131 |
| C[C@H]1CC[C@H](CNC(=O)c2cccc3C(=NNC(=O)c23)Cc4ccc(F)c(c4)C(=O)N5CCN(CC5)C(=O)C6CC6)CN1C7CCN(C)CC7 | Olaparib 222 |
| Nc1cccc(c2cccc3C(=NNC(=O)c23)Cc4ccc(F)c(c4)C(=O)N5CCN(CC5)C(=O)C6CC6)c1C(=O)O | Olaparib 1722 |
| OC(=O)C1(CC1)c2ccc(F)c(Oc3cccc4C(=NNC(=O)c34)Cc5ccc(F)c(c5)C(=O)N6CCN(CC6)C(=O)C7CC7)c2 | Olaparib 2480 |
| Fc1ccc(CC2=NNC(=O)c3c(OC4C[C@H]5CC[C@@H](C4)N5C(=O)C6CCCCC6)cccc23)cc1C(=O)N7CCN(CC7)C(=O)C8CC8 | Olaparib 3174 |
| Cc1onc(Cn2ccc3cc(Oc4cccc5C(=NNC(=O)c45)Cc6ccc(F)c(c6)C(=O)N7CCN(CC7)C(=O)C8CC8)ccc23)c1 | Olaparib 2733 |
| Fc1ccc(CC2=NNC(=O)c3c(Oc4nc5ccc(Br)cc5cc4C=O)cccc23)cc1C(=O)N6CCN(CC6)C(=O)C7CC7 | Olaparib 2454 |
| CNc1cc(nc2c(C)c(Oc3cccc4C(=NNC(=O)c34)Cc5ccc(F)c(c5)C(=O)N6CCN(CC6)C(=O)C7CC7)ccc12)C8CC8 | Olaparib 2732 |
| CN1CCN(CC1)C(=O)c2cc3cc(Oc4cccc5C(=NNC(=O)c45)Cc6ccc(F)c(c6)C(=O)N7CCN(CC7)C(=O)C8CC8)ccc3[nH]2 | Olaparib 2633 |
| Fc1ccc(CC2=NNC(=O)c3c(cccc23)C(=O)Oc4ccc5CNCC6(CCCC6)c5c4)cc1C(=O)N7CCN(CC7)C(=O)C8CC8 | Olaparib 1166 |
| CC(F)(F)CC(=O)Oc1cccc2C(=NNC(=O)c12)Cc3ccc(F)c(c3)C(=O)N4CCN(CC4)C(=O)C5CC5 | Olaparib 1315 |
| Cn1c(nc2cc(F)ccc12)c3cncc(OC(=O)c4cccc5C(=NNC(=O)c45)Cc6ccc(F)c(c6)C(=O)N7CCN(CC7)C(=O)C8CC8)c3 | Olaparib 1134 |
| O[C@H](c1ccccc1)c2c(F)c(F)c(F)c(Oc3cccc4C(=NNC(=O)c34)Cc5ccc(F)c(c5)C(=O)N6CCN(CC6)C(=O)C7CC7)c2F | Olaparib 2446 |
| Cc1nc(Cl)cc(Cl)c1C(=O)Oc2cccc3C(=NNC(=O)c23)Cc4ccc(F)c(c4)C(=O)N5CCN(CC5)C(=O)C6CC6 | Olaparib 1446 |
| Nc1nn(cc1Cl)c2ccc(cc2)C(F)(F)c3cccc4C(=NNC(=O)c34)Cc5ccc(F)c(c5)C(=O)N6CCN(CC6)C(=O)C7CC7 | Olaparib 1741 |
| CN1C(=O)N(C)c2ncc(Oc3cccc4C(=NNC(=O)c34)Cc5ccc(F)c(c5)C(=O)N6CCN(CC6)C(=O)C7CC7)nc2C1=O | Olaparib 2551 |
| Fc1ccc(CC2=NNC(=O)c3c(OC(=O)CN4C(=O)c5cccc6cccc4c56)cccc23)cc1C(=O)N7CCN(CC7)C(=O)C8CC8 | Olaparib 1592 |
| CN1CCC[C@@H](N)[C@@H]1c2ccc(Oc3cccc4C(=NNC(=O)c34)Cc5ccc(F)c(c5)C(=O)N6CCN(CC6)C(=O)C7CC7)c(F)c2 | Olaparib 2525 |
| O[C@@H]1CC[C@H](CC1)c2onc(n2)c3cccc(Oc4cccc5C(=NNC(=O)c45)Cc6ccc(F)c(c6)C(=O)N7CCN(CC7)C(=O)C8CC8)c3 | Olaparib 2673 |
| Fc1ccc(CC2=NNC(=O)c3c(Oc4cc5[C@H]6CC[C@H](N6)c5cc4Cl)cccc23)cc1C(=O)N7CCN(CC7)C(=O)C8CC8 | Olaparib 2744 |
| N[C@H]1CCN(C2CC2)[C@@H]1c3ccc(Oc4cccc5C(=NNC(=O)c45)Cc6ccc(F)c(c6)C(=O)N7CCN(CC7)C(=O)C8CC8)cc3 | Olaparib 2725 |
| Fc1ccc(CC2=NNC(=O)c3c(OC(=O)C(C(F)(F)F)C(F)(F)F)cccc23)cc1C(=O)N4CCN(CC4)C(=O)C5CC5 | Olaparib 1178 |
| Fc1ccc(CC2=NNC(=O)c3c(Oc4ccc(cc4)c5oc(Nc6ccccc6)nn5)cccc23)cc1C(=O)N7CCN(CC7)C(=O)C8CC8 | Olaparib 2652 |
| Cc1cc(Oc2cccc3C(=NNC(=O)c23)Cc4ccc(F)c(c4)C(=O)N5CCN(CC5)C(=O)C6CC6)ccc1c7oc(cc7)[C@@H]8C[C@H]8C(=O)O | Olaparib 2685 |
| Fc1ccc(CC2=NNC(=O)c3c2cccc3c4nc(Cl)nc5ccc(Br)cc45)cc1C(=O)N6CCN(CC6)C(=O)C7CC7 | Olaparib 1734 |
| Oc1cncc(C=O)c1c2cccc3C(=NNC(=O)c23)Cc4ccc(F)c(c4)C(=O)N5CCN(CC5)C(=O)C6CC6 | Olaparib 1987 |
| Oc1cc2[nH]c3cc(OC(=O)c4cccc5C(=NNC(=O)c45)Cc6ccc(F)c(c6)C(=O)N7CCN(CC7)C(=O)C8CC8)ccc3c2cc1C=O | Olaparib 1156 |
| Cc1nccn1c2cccc(n2)c3cccc4C(=NNC(=O)c34)Cc5ccc(F)c(c5)C(=O)N6CCN(CC6)C(=O)C7CC7 | Olaparib 2066 |
| Cc1c(nnn1C2CCOCC2)C(=O)Oc3cccc4C(=NNC(=O)c34)Cc5ccc(F)c(c5)C(=O)N6CCN(CC6)C(=O)C7CC7 | Olaparib 1557 |
| NC1(CC1)c2cc(Oc3cccc4C(=NNC(=O)c34)Cc5ccc(F)c(c5)C(=O)N6CCN(CC6)C(=O)C7CC7)c(F)c(F)c2O | Olaparib 2474 |
| Cc1oc(COc2cccc3[C@@H](CCc23)Oc4cccc5C(=NNC(=O)c45)Cc6ccc(F)c(c6)C(=O)N7CCN(CC7)C(=O)C8CC8)nn1 | Olaparib 3157 |
| Fc1ccc(CC2=NNC(=O)c3c2cccc3c4cccc(CN5C[C@H]6CC[C@@H]5C6)c4)cc1C(=O)N7CCN(CC7)C(=O)C8CC8 | Olaparib 2089 |
| Fc1ccc(CC2=NNC(=O)c3c(Oc4ccc5c(ccn5CCNC6CCOCC6)c4)cccc23)cc1C(=O)N7CCN(CC7)C(=O)C8CC8 | Olaparib 2625 |
| Fc1ccc(CC2=NNC(=O)c3c(Oc4ccc(cn4)[C@H]5C[C@@H]6CC[C@H]5N6)cccc23)cc1C(=O)N7CCN(CC7)C(=O)C8CC8 | Olaparib 2746 |
| CC[C@@H](Oc1cccc2C(=NNC(=O)c12)Cc3ccc(F)c(c3)C(=O)N4CCN(CC4)C(=O)C5CC5)C(Cl)(Cl)Cl | Olaparib 2749 |
| Oc1ccc2c(c1)[nH]c3cc(OC(=O)c4cccc5C(=NNC(=O)c45)Cc6ccc(F)c(c6)C(=O)N7CCN(CC7)C(=O)C8CC8)c(C=O)cc23 | Olaparib 1155 |
| C[C@@H]1CCN(C1)C(=O)NC(=O)\C=C\C(=O)Nc2cccc3C(=NNC(=O)c23)Cc4ccc(F)c(c4)C(=O)N5CCN(CC5)C(=O)C6CC6 | Olaparib 464 |
| Nc1c(F)c(F)c(F)c(N)c1Oc2cccc3C(=NNC(=O)c23)Cc4ccc(F)c(c4)C(=O)N5CCN(CC5)C(=O)C6CC6 | Olaparib 2357 |
| CSC[C@@H](OC(=O)c1cccc2C(=NNC(=O)c12)Cc3ccc(F)c(c3)C(=O)N4CCN(CC4)C(=O)C5CC5)c6cncc7ccccc67 | Olaparib 1028 |
| Fc1ccc(CC2=NNC(=O)c3c2cccc3C(F)(F)c4oc(nn4)c5ccccc5)cc1C(=O)N6CCN(CC6)C(=O)C7CC7 | Olaparib 1760 |
| CC[C@@]1(C)C[C@]2(CCO1)C[C@@H]2NC(=O)c3cccc4C(=NNC(=O)c34)Cc5ccc(F)c(c5)C(=O)N6CCN(CC6)C(=O)C7CC7 | Olaparib 247 |
| C[C@H]1CCC[C@@H](CNC(=O)c2cccc3C(=NNC(=O)c23)Cc4ccc(F)c(c4)C(=O)N5CCN(CC5)C(=O)C6CC6)N1[C@H]7CCS(=O)(=O)C7 | Olaparib 203 |
| Fc1ccc(CC2=NNC(=O)c3c(Oc4cc(ccc4Cl)c5cn6ccccc6n5)cccc23)cc1C(=O)N7CCN(CC7)C(=O)C8CC8 | Olaparib 2669 |
| Fc1ccc(CC2=NNC(=O)c3c(Oc4cccc(c4)c5noc(n5)[C@H]6CCCS6)cccc23)cc1C(=O)N7CCN(CC7)C(=O)C8CC8 | Olaparib 3151 |
| Fc1ccc(CC2=NNC(=O)c3c(Oc4ccc5c(c4)C(=O)c6ccccc56)cccc23)cc1C(=O)N7CCN(CC7)C(=O)C8CC8 | Olaparib 2742 |
| Fc1ccc(CC2=NNC(=O)c3c(CCCC(F)(F)F)cccc23)cc1C(=O)N4CCN(CC4)C(=O)C5CC5 | Olaparib 1922 |
| Cn1nccc1CNc2ccnc3cc(Oc4cccc5C(=NNC(=O)c45)Cc6ccc(F)c(c6)C(=O)N7CCN(CC7)C(=O)C8CC8)ccc23 | Olaparib 2647 |
| C[C@@H]1CCN(C1)C(=O)NC(=O)\C=C\C(=O)Oc2cccc3C(=NNC(=O)c23)Cc4ccc(F)c(c4)C(=O)N5CCN(CC5)C(=O)C6CC6 | Olaparib 1422 |
| Nc1c(O)c(Cl)cc(Cl)c1Oc2cccc3C(=NNC(=O)c23)Cc4ccc(F)c(c4)C(=O)N5CCN(CC5)C(=O)C6CC6 | Olaparib 2276 |
| C[C@@H](NCc1scnc1C)c2cccc(Oc3cccc4C(=NNC(=O)c34)Cc5ccc(F)c(c5)C(=O)N6CCN(CC6)C(=O)C7CC7)c2 | Olaparib 2509 |
| Cc1nc2ccccc2nc1Oc3cccc4C(=NNC(=O)c34)Cc5ccc(F)c(c5)C(=O)N6CCN(CC6)C(=O)C7CC7 | Olaparib 3139 |
| Nc1ccc(cc1)c2cccc(Oc3cccc4C(=NNC(=O)c34)Cc5ccc(F)c(c5)C(=O)N6CCN(CC6)C(=O)C7CC7)c2F | Olaparib 2594 |
| COC(=O)c1cc(OC)c2cc(Oc3cccc4C(=NNC(=O)c34)Cc5ccc(F)c(c5)C(=O)N6CCN(CC6)C(=O)C7CC7)c(F)cc2n1 | Olaparib 2500 |
| Cc1c(cccc1c2cccc3C(=NNC(=O)c23)Cc4ccc(F)c(c4)C(=O)N5CCN(CC5)C(=O)C6CC6)c7oc(cc7)[C@@H]8C[C@H]8C(=O)O | Olaparib 1786 |
| Fc1ccc(CC2=NNC(=O)c3c(Oc4ccc5sc(COC6CCNCC6)nc5c4)cccc23)cc1C(=O)N7CCN(CC7)C(=O)C8CC8 | Olaparib 2659 |
| Fc1ccc(CC2=NNC(=O)c3c2cccc3c4ccnc5[nH]c(cc45)C6CC6)cc1C(=O)N7CCN(CC7)C(=O)C8CC8 | Olaparib 2092 |
| Fc1ccc(CC2=NNC(=O)c3c2cccc3C(F)(F)C(F)(F)I)cc1C(=O)N4CCN(CC4)C(=O)C5CC5 | Olaparib 1602 |
| Cc1cc2ncc(CNc3ccc(Oc4cccc5C(=NNC(=O)c45)Cc6ccc(F)c(c6)C(=O)N7CCN(CC7)C(=O)C8CC8)cc3)cn2n1 | Olaparib 2646 |
| OC(=O)C1=NN(C[C@@H](F)Oc2cccc3C(=NNC(=O)c23)Cc4ccc(F)c(c4)C(=O)N5CCN(CC5)C(=O)C6CC6)C(=O)CC1 | Olaparib 2291 |
| Fc1ccc(CC2=NNC(=O)c3c2cccc3C4=CN=CC(=O)N4)cc1C(=O)N5CCN(CC5)C(=O)C6CC6 | Olaparib 2021 |
| C[C@H](N)c1ccc(OCc2ccc(Oc3cccc4C(=NNC(=O)c34)Cc5ccc(F)c(c5)C(=O)N6CCN(CC6)C(=O)C7CC7)s2)cc1 | Olaparib 2464 |
| Fc1ccc(CC2=NNC(=O)c3c2cccc3c4ccnc(c4)c5nccs5)cc1C(=O)N6CCN(CC6)C(=O)C7CC7 | Olaparib 2062 |
| Fc1ccc(CC2=NNC(=O)c3c2cccc3c4ccnc(c4)c5ccnc(Cl)c5)cc1C(=O)N6CCN(CC6)C(=O)C7CC7 | Olaparib 2055 |
| Fc1ccc(CC2=NNC(=O)c3c(Oc4ccc5oc(nc5c4)N6CCOCC6)cccc23)cc1C(=O)N7CCN(CC7)C(=O)C8CC8 | Olaparib 2724 |
| Cc1nn(C)c(NCc2ccc(F)c(Oc3cccc4C(=NNC(=O)c34)Cc5ccc(F)c(c5)C(=O)N6CCN(CC6)C(=O)C7CC7)c2)c1C#N | Olaparib 2431 |
| Fc1ccc(CC2=NNC(=O)c3c(Oc4ccc5c(c4)[nH]c6ccccc56)cccc23)cc1C(=O)N7CCN(CC7)C(=O)C8CC8 | Olaparib 2747 |
| Fc1ccc(CC2=NNC(=O)c3c(Oc4ccc(cc4)N5C(=O)CSC5=S)cccc23)cc1C(=O)N6CCN(CC6)C(=O)C7CC7 | Olaparib 3076 |
| Fc1ccc(CC2=NNC(=O)c3c(O[C@H](CN4CCC(=O)CC4)C(F)(F)F)cccc23)cc1C(=O)N5CCN(CC5)C(=O)C6CC6 | Olaparib 2869 |
| CC1(CCN(CC1)C(=O)NCC(F)(F)Oc2cccc3C(=NNC(=O)c23)Cc4ccc(F)c(c4)C(=O)N5CCN(CC5)C(=O)C6CC6)C(=O)O | Olaparib 2181 |
| Cc1cc(Oc2cccc3C(=NNC(=O)c23)Cc4ccc(F)c(c4)C(=O)N5CCN(CC5)C(=O)C6CC6)ccc1Nc7nc8ccc(N)cc8s7 | Olaparib 2644 |
| [O-][n+]1c2ccccc2[n+]([O-])c3cc(Oc4cccc5C(=NNC(=O)c45)Cc6ccc(F)c(c6)C(=O)N7CCN(CC7)C(=O)C8CC8)ccc13 | Olaparib 3184 |
| Fc1ccc(CC2=NNC(=O)c3c(Oc4ccc5nc(Cl)nc(Cl)c5c4)cccc23)cc1C(=O)N6CCN(CC6)C(=O)C7CC7 | Olaparib 2435 |
| Fc1ccc(CC2=NNC(=O)c3c(Oc4nccc(n4)c5cccs5)cccc23)cc1C(=O)N6CCN(CC6)C(=O)C7CC7 | Olaparib 2600 |
| Oc1cccc2nccc(c3cccc4C(=NNC(=O)c34)Cc5ccc(F)c(c5)C(=O)N6CCN(CC6)C(=O)C7CC7)c12 | Olaparib 2074 |
| Fc1ccc(CC2=NNC(=O)c3c(cccc23)C(=O)OCCC4c5ccccc5c6ccccc46)cc1C(=O)N7CCN(CC7)C(=O)C8CC8 | Olaparib 1164 |
| Fc1ccc(CC2=NNC(=O)c3c(O[C@@H]4C[C@@H]5N(C4)C(=O)c6ccccc6NC5=O)cccc23)cc1C(=O)N7CCN(CC7)C(=O)C8CC8 | Olaparib 3178 |
| O[C@@H](Cc1onc(n1)c2ccc(F)c(Oc3cccc4C(=NNC(=O)c34)Cc5ccc(F)c(c5)C(=O)N6CCN(CC6)C(=O)C7CC7)c2)C(F)F | Olaparib 2439 |
| Cc1nc2ccc(OC(=O)c3cccc4C(=NNC(=O)c34)Cc5ccc(F)c(c5)C(=O)N6CCN(CC6)C(=O)C7CC7)cc2s1 | Olaparib 1101 |
| C[C@@H]1CNC[C@H](C)N1CC(C)(C)Oc2cccc3C(=NNC(=O)c23)Cc4ccc(F)c(c4)C(=O)N5CCN(CC5)C(=O)C6CC6 | Olaparib 2886 |
| Cc1nn(Cc2oc3ccccc3c2)c(N)c1Oc4cccc5C(=NNC(=O)c45)Cc6ccc(F)c(c6)C(=O)N7CCN(CC7)C(=O)C8CC8 | Olaparib 2676 |
| CC[C@]1(CCN(C1)C(=O)NCC(F)(F)Oc2cccc3C(=NNC(=O)c23)Cc4ccc(F)c(c4)C(=O)N5CCN(CC5)C(=O)C6CC6)C(=O)O | Olaparib 2180 |
| Cc1nc2ccccc2n1COc3cccc4C(=NNC(=O)c34)Cc5ccc(F)c(c5)C(=O)N6CCN(CC6)C(=O)C7CC7 | Olaparib 3131 |
| Fc1ccc(CC2=NNC(=O)c3c(OCCn4ccc5ccccc45)cccc23)cc1C(=O)N6CCN(CC6)C(=O)C7CC7 | Olaparib 3136 |
| Fc1ccc(CC2=NNC(=O)c3c2cccc3c4cccc(CN5Cc6ccccc6C5)c4)cc1C(=O)N7CCN(CC7)C(=O)C8CC8 | Olaparib 2083 |
| CC(C)n1cnc2c(Oc3cccc4C(=NNC(=O)c34)Cc5ccc(F)c(c5)C(=O)N6CCN(CC6)C(=O)C7CC7)ncnc12 | Olaparib 2601 |
| CC1(C)Cc2cccc(O[C@@H]3CNC[C@H]3OC(=O)c4cccc5C(=NNC(=O)c45)Cc6ccc(F)c(c6)C(=O)N7CCN(CC7)C(=O)C8CC8)c2O1 | Olaparib 1121 |
| C[C@H](NC(=O)c1cccc2C(=NNC(=O)c12)Cc3ccc(F)c(c3)C(=O)N4CCN(CC4)C(=O)C5CC5)c6ccc(cc6)N7CCSC[C@H]7C | Olaparib 211 |
| CNc1nc2cc(Oc3cccc4C(=NNC(=O)c34)Cc5ccc(F)c(c5)C(=O)N6CCN(CC6)C(=O)C7CC7)ccc2s1 | Olaparib 2598 |
| CC[C@@H](CN1C[C@@H](C)S[C@@H](C)C1)Oc2cccc3C(=NNC(=O)c23)Cc4ccc(F)c(c4)C(=O)N5CCN(CC5)C(=O)C6CC6 | Olaparib 2870 |
| Cc1ccc2sc3nc(CC(=O)Oc4cccc5C(=NNC(=O)c45)Cc6ccc(F)c(c6)C(=O)N7CCN(CC7)C(=O)C8CC8)cn3c2c1 | Olaparib 1577 |
| NCCC1CCN(CC1)S(=O)(=O)[C@@H](F)Oc2cccc3C(=NNC(=O)c23)Cc4ccc(F)c(c4)C(=O)N5CCN(CC5)C(=O)C6CC6 | Olaparib 2215 |
| Fc1ccc(CC2=NNC(=O)c3c2cccc3c4cnc5ccccn45)cc1C(=O)N6CCN(CC6)C(=O)C7CC7 | Olaparib 2082 |
| Cc1csc2ncnc(c3cccc4C(=NNC(=O)c34)Cc5ccc(F)c(c5)C(=O)N6CCN(CC6)C(=O)C7CC7)c12 | Olaparib 1771 |
| Fc1ccc(CC2=NNC(=O)c3c(OCCC4c5ccccc5c6ccccc46)cccc23)cc1C(=O)N7CCN(CC7)C(=O)C8CC8 | Olaparib 3198 |
| CC[C@H]1N(CCNC1=O)C(=O)c2cccc(n2)c3cccc4C(=NNC(=O)c34)Cc5ccc(F)c(c5)C(=O)N6CCN(CC6)C(=O)C7CC7 | Olaparib 1738 |
| NCc1cnc(cn1)N2[C@@H]3CC[C@H]2CC(C3)Oc4cccc5C(=NNC(=O)c45)Cc6ccc(F)c(c6)C(=O)N7CCN(CC7)C(=O)C8CC8 | Olaparib 3177 |
| CSc1ccc(cc1)N2C[C@@H](CC2=O)Oc3cccc4C(=NNC(=O)c34)Cc5ccc(F)c(c5)C(=O)N6CCN(CC6)C(=O)C7CC7 | Olaparib 3077 |
| CN(C(=O)[C@@H]1CCSC1)c2ccc(Oc3cccc4C(=NNC(=O)c34)Cc5ccc(F)c(c5)C(=O)N6CCN(CC6)C(=O)C7CC7)cc2 | Olaparib 3063 |
| CC[C@@]1(C)C[C@](N)(COc2cccc3C(=NNC(=O)c23)Cc4ccc(F)c(c4)C(=O)N5CCN(CC5)C(=O)C6CC6)CCO1 | Olaparib 2908 |
| Nc1ccc(Oc2cccc3C(=NNC(=O)c23)Cc4ccc(F)c(c4)C(=O)N5CCN(CC5)C(=O)C6CC6)cc1N7C(=O)c8ccccc8C7=O | Olaparib 2704 |
| C[C@]1(CCN(C1)C(=O)OCC(F)(F)Oc2cccc3C(=NNC(=O)c23)Cc4ccc(F)c(c4)C(=O)N5CCN(CC5)C(=O)C6CC6)C(=O)O | Olaparib 2192 |
| Fc1ccc(CC2=NNC(=O)c3c(NC(=O)CNC(=O)N4CCc5ccccc5C4)cccc23)cc1C(=O)N6CCN(CC6)C(=O)C7CC7 | Olaparib 526 |
| Fc1ccc(CC2=NNC(=O)c3c2cccc3c4cnc5ccc(Cl)cn45)cc1C(=O)N6CCN(CC6)C(=O)C7CC7 | Olaparib 1907 |
| Cn1c(CNC(=O)c2cccc3C(=NNC(=O)c23)Cc4ccc(F)c(c4)C(=O)N5CCN(CC5)C(=O)C6CC6)nnc1SC7CCOCC7 | Olaparib 220 |
| Fc1ccc(CC2=NNC(=O)c3c2cccc3c4cccc(n4)N5C=CC=CC5=O)cc1C(=O)N6CCN(CC6)C(=O)C7CC7 | Olaparib 2059 |
| Cc1nn(C)c(N2[C@@H]3CC[C@H]2CC(C3)Oc4cccc5C(=NNC(=O)c45)Cc6ccc(F)c(c6)C(=O)N7CCN(CC7)C(=O)C8CC8)c1C#N | Olaparib 3154 |
| CC1(C)CCCN(C1)c2nsnc2c3cccc4C(=NNC(=O)c34)Cc5ccc(F)c(c5)C(=O)N6CCN(CC6)C(=O)C7CC7 | Olaparib 1752 |
| Fc1ccc(CC2=NNC(=O)c3c(NC(=O)Cc4nc5CCCc5s4)cccc23)cc1C(=O)N6CCN(CC6)C(=O)C7CC7 | Olaparib 544 |
| O[C@H]1CCC[C@@H]1[C@](O)(C(F)(F)F)C(F)(F)Oc2cccc3C(=NNC(=O)c23)Cc4ccc(F)c(c4)C(=O)N5CCN(CC5)C(=O)C6CC6 | Olaparib 2200 |
| Oc1cc(O)c2C(=O)C(=COc2c1)c3ccc(Oc4cccc5C(=NNC(=O)c45)Cc6ccc(F)c(c6)C(=O)N7CCN(CC7)C(=O)C8CC8)cc3 | Olaparib 2650 |
| CN(CC(=O)Nc1cccc2C(=NNC(=O)c12)Cc3ccc(F)c(c3)C(=O)N4CCN(CC4)C(=O)C5CC5)C(=O)Nc6ccc(C)c(C)c6 | Olaparib 460 |
| COC1=Cc2cnc(Oc3cccc4C(=NNC(=O)c34)Cc5ccc(F)c(c5)C(=O)N6CCN(CC6)C(=O)C7CC7)cc2NC1=O | Olaparib 2586 |
| Fc1ccc(CC2=NNC(=O)c3c(cccc23)C(=C)CN[C@H]4COc5ccccc45)cc1C(=O)N6CCN(CC6)C(=O)C7CC7 | Olaparib 1763 |
| NC(=O)c1ccc(c(F)c1)n2cc(Oc3cccc4C(=NNC(=O)c34)Cc5ccc(F)c(c5)C(=O)N6CCN(CC6)C(=O)C7CC7)c(N)n2 | Olaparib 2495 |
| Fc1ccc(CC2=NNC(=O)c3c(OC4C[C@H]5CC[C@@H](C4)N5c6nncs6)cccc23)cc1C(=O)N7CCN(CC7)C(=O)C8CC8 | Olaparib 3197 |
| C[C@@H](NC(=O)c1cccc2C(=NNC(=O)c12)Cc3ccc(F)c(c3)C(=O)N4CCN(CC4)C(=O)C5CC5)c6ccc(cc6)N7CCC(C)(C)CC7 | Olaparib 214 |
| CCN(CC1CC1)c2nsnc2c3cccc4C(=NNC(=O)c34)Cc5ccc(F)c(c5)C(=O)N6CCN(CC6)C(=O)C7CC7 | Olaparib 1758 |
| Fc1ccc(CC2=NNC(=O)c3c(OC(=O)Cc4nc5CCCc5s4)cccc23)cc1C(=O)N6CCN(CC6)C(=O)C7CC7 | Olaparib 1570 |
| CCOc1cc(ccn1)c2cccc3C(=NNC(=O)c23)Cc4ccc(F)c(c4)C(=O)N5CCN(CC5)C(=O)C6CC6 | Olaparib 1985 |
| Oc1ccc2C(=O)c3ccc(OC(=O)c4cccc5C(=NNC(=O)c45)Cc6ccc(F)c(c6)C(=O)N7CCN(CC7)C(=O)C8CC8)cc3Oc2c1 | Olaparib 1151 |
| C[C@H](NC(=O)Nc1cc(C)cc(C)c1)C(=O)Nc2cccc3C(=NNC(=O)c23)Cc4ccc(F)c(c4)C(=O)N5CCN(CC5)C(=O)C6CC6 | Olaparib 461 |
| C[C@H]1CC[C@H](CNC(=O)c2cccc3C(=NNC(=O)c23)Cc4ccc(F)c(c4)C(=O)N5CCN(CC5)C(=O)C6CC6)CN1c7ncccc7C#N | Olaparib 216 |
| Nc1ccc(cc1c2cccc3C(=NNC(=O)c23)Cc4ccc(F)c(c4)C(=O)N5CCN(CC5)C(=O)C6CC6)C7(CC7)C#N | Olaparib 1774 |
| Cc1ccc(nn1)n2cc(Oc3cccc4C(=NNC(=O)c34)Cc5ccc(F)c(c5)C(=O)N6CCN(CC6)C(=O)C7CC7)c(N)n2 | Olaparib 2587 |
| C[C@@H](Oc1cccc2C(=NNC(=O)c12)Cc3ccc(F)c(c3)C(=O)N4CCN(CC4)C(=O)C5CC5)C(=O)N6CCC(Cc7ccccc7)CC6 | Olaparib 3055 |
| CN1C=CN=C(N2[C@@H]3CC[C@H]2CC(C3)Oc4cccc5C(=NNC(=O)c45)Cc6ccc(F)c(c6)C(=O)N7CCN(CC7)C(=O)C8CC8)C1=O | Olaparib 3176 |
| C[C@@H](NC(=O)c1cccc2C(=NNC(=O)c12)Cc3ccc(F)c(c3)C(=O)N4CCN(CC4)C(=O)C5CC5)[C@H]6CCCN(C6)[C@@H]7CCCSC7 | Olaparib 218 |
| Fc1ccc(CC2=NNC(=O)c3c(cccc23)C(=O)Oc4ccc(\C=C\c5ccncc5)cc4)cc1C(=O)N6CCN(CC6)C(=O)C7CC7 | Olaparib 1058 |
| Nc1cc(c2cccc3C(=NNC(=O)c23)Cc4ccc(F)c(c4)C(=O)N5CCN(CC5)C(=O)C6CC6)c(cn1)C(F)(F)F | Olaparib 1960 |
| Cc1cc(ccc1Oc2cccc3C(=NNC(=O)c23)Cc4ccc(F)c(c4)C(=O)N5CCN(CC5)C(=O)C6CC6)c7noc(CC(=O)C8CC8)n7 | Olaparib 2681 |
| CN(CC(=O)Oc1cccc2C(=NNC(=O)c12)Cc3ccc(F)c(c3)C(=O)N4CCN(CC4)C(=O)C5CC5)C(=O)Nc6ccc(C)c(C)c6 | Olaparib 1415 |
| CS(=O)(=O)c1ncccc1c2cccc3C(=NNC(=O)c23)Cc4ccc(F)c(c4)C(=O)N5CCN(CC5)C(=O)C6CC6 | Olaparib 1964 |
| C[C@@H](N1C[C@@H](CC1=O)Oc2cccc3C(=NNC(=O)c23)Cc4ccc(F)c(c4)C(=O)N5CCN(CC5)C(=O)C6CC6)c7ccccc7C | Olaparib 3083 |
| Fc1ccc(CC2=NNC(=O)c3c(NC(=O)CO\N=C/4\CCCc5sccc45)cccc23)cc1C(=O)N6CCN(CC6)C(=O)C7CC7 | Olaparib 530 |
| Nc1snnc1CN2C=C(C=CC2=O)C(F)(F)c3cccc4C(=NNC(=O)c34)Cc5ccc(F)c(c5)C(=O)N6CCN(CC6)C(=O)C7CC7 | Olaparib 1736 |
| O[C@H]1CN[C@H](C1)C(=O)N2[C@@H]3CC[C@H]2CC(C3)Oc4cccc5C(=NNC(=O)c45)Cc6ccc(F)c(c6)C(=O)N7CCN(CC7)C(=O)C8CC8 | Olaparib 3168 |
| O[C@@H](C(=O)Nc1cccc2C(=NNC(=O)c12)Cc3ccc(F)c(c3)C(=O)N4CCN(CC4)C(=O)C5CC5)c6ccc(cc6)c7ccccc7 | Olaparib 528 |
| Fc1ccc(CC2=NNC(=O)c3c(Oc4ccc(cc4)C(=O)C(=O)c5ccccc5)cccc23)cc1C(=O)N6CCN(CC6)C(=O)C7CC7 | Olaparib 2524 |
| COc1cncc(c1)c2cccc3C(=NNC(=O)c23)Cc4ccc(F)c(c4)C(=O)N5CCN(CC5)C(=O)C6CC6 | Olaparib 1913 |
| Fc1ccc(CC2=NNC(=O)c3c(NC(=O)CC4CN(C4)c5cnnc6ccccc56)cccc23)cc1C(=O)N7CCN(CC7)C(=O)C8CC8 | Olaparib 551 |
| C[C@H](NC(=O)Nc1cc(C)ccc1F)C(=O)Nc2cccc3C(=NNC(=O)c23)Cc4ccc(F)c(c4)C(=O)N5CCN(CC5)C(=O)C6CC6 | Olaparib 459 |
| CCc1cc(Oc2cccc3C(=NNC(=O)c23)Cc4ccc(F)c(c4)C(=O)N5CCN(CC5)C(=O)C6CC6)nc(n1)c7ccc8ncccc8c7 | Olaparib 2655 |
| NC(=N)Sc1ccc(F)c(Oc2cccc3C(=NNC(=O)c23)Cc4ccc(F)c(c4)C(=O)N5CCN(CC5)C(=O)C6CC6)c1 | Olaparib 2298 |
| Cc1c(Oc2cccc3C(=NNC(=O)c23)Cc4ccc(F)c(c4)C(=O)N5CCN(CC5)C(=O)C6CC6)ccc7C(=O)CC(C)(C)Oc17 | Olaparib 3095 |
| Fc1ccc(CC2=NNC(=O)c3c(OC(=O)CO\N=C/4\CCCc5sccc45)cccc23)cc1C(=O)N6CCN(CC6)C(=O)C7CC7 | Olaparib 1544 |
| Fc1ccc(CC2=NNC(=O)c3c(NC(=O)CC4CN(C4)C(=O)N5CCCNCC5)cccc23)cc1C(=O)N6CCN(CC6)C(=O)C7CC7 | Olaparib 522 |
| Fc1ccc(CC2=NNC(=O)c3c(Oc4cccc(c4)c5oc(nn5)c6ccccc6)cccc23)cc1C(=O)N7CCN(CC7)C(=O)C8CC8 | Olaparib 2702 |
| Fc1ccc(CC2=NNC(=O)c3c(cccc23)C(=O)O[C@@H](Cc4ccccc4)c5ccccn5)cc1C(=O)N6CCN(CC6)C(=O)C7CC7 | Olaparib 1056 |
| Cc1ccc(cc1)C(=O)Nc2ccc(Oc3cccc4C(=NNC(=O)c34)Cc5ccc(F)c(c5)C(=O)N6CCN(CC6)C(=O)C7CC7)cc2 | Olaparib 3072 |
| [2H]c1nc([2H])c(nc1Cl)c2cccc3C(=NNC(=O)c23)Cc4ccc(F)c(c4)C(=O)N5CCN(CC5)C(=O)C6CC6 | Olaparib 1992 |
| Fc1ccc(CC2=NNC(=O)c3c(cccc23)C(=O)Oc4cccc5[C@H](CCc45)NCc6cscn6)cc1C(=O)N7CCN(CC7)C(=O)C8CC8 | Olaparib 1125 |
| CSc1cc(c(O)cc1C)[C@@](C)(N)C(=O)Oc2cccc3C(=NNC(=O)c23)Cc4ccc(F)c(c4)C(=O)N5CCN(CC5)C(=O)C6CC6 | Olaparib 1409 |
| CSCCn1cnnc1c2cccc3C(=NNC(=O)c23)Cc4ccc(F)c(c4)C(=O)N5CCN(CC5)C(=O)C6CC6 | Olaparib 1707 |
| Nc1ccc(cc1c2cccc3C(=NNC(=O)c23)Cc4ccc(F)c(c4)C(=O)N5CCN(CC5)C(=O)C6CC6)[C@@H]7CCC(=O)N7 | Olaparib 1770 |
| C[C@@H]1CCCCCN1c2ncc(CNC(=O)c3cccc4C(=NNC(=O)c34)Cc5ccc(F)c(c5)C(=O)N6CCN(CC6)C(=O)C7CC7)s2 | Olaparib 223 |
| Cc1cc(ccc1n2cc(Cl)c(C)n2)C(=N)NC(=O)c3cccc4C(=NNC(=O)c34)Cc5ccc(F)c(c5)C(=O)N6CCN(CC6)C(=O)C7CC7 | Olaparib 201 |
| Cc1ccc2nc([nH]c2c1)c3ccc(N)c(Oc4cccc5C(=NNC(=O)c45)Cc6ccc(F)c(c6)C(=O)N7CCN(CC7)C(=O)C8CC8)c3 | Olaparib 2722 |
| C[C@@H](C(=O)Oc1cccc2C(=NNC(=O)c12)Cc3ccc(F)c(c3)C(=O)N4CCN(CC4)C(=O)C5CC5)c6ccc7cc(O)ccc7c6 | Olaparib 1552 |
| [O-]\[N+](=C/c1ccc(Oc2cccc3C(=NNC(=O)c23)Cc4ccc(F)c(c4)C(=O)N5CCN(CC5)C(=O)C6CC6)cc1)\c7ccccc7 | Olaparib 2570 |
| CC1=CNC(=O)C=C1c2cccc3C(=NNC(=O)c23)Cc4ccc(F)c(c4)C(=O)N5CCN(CC5)C(=O)C6CC6 | Olaparib 2004 |
| Fc1ccc(CC2=NNC(=O)c3c(Oc4ccc(cn4)C(=O)N[C@H]5CCCc6sccc56)cccc23)cc1C(=O)N7CCN(CC7)C(=O)C8CC8 | Olaparib 2636 |
| CC(C)[C@H](C)NC(=O)Cn1cc(c(N)n1)c2cccc3C(=NNC(=O)c23)Cc4ccc(F)c(c4)C(=O)N5CCN(CC5)C(=O)C6CC6 | Olaparib 1653 |
| Oc1nc(Oc2cccc3C(=NNC(=O)c23)Cc4ccc(F)c(c4)C(=O)N5CCN(CC5)C(=O)C6CC6)sc1Br | Olaparib 2875 |
| Oc1c(ccc(F)c1c2cccc3C(=NNC(=O)c23)Cc4ccc(F)c(c4)C(=O)N5CCN(CC5)C(=O)C6CC6)[C@@H]7CCC(=O)N7 | Olaparib 1762 |
| Fc1ccc(CC2=NNC(=O)c3c(cccc23)C(=O)NCc4cnn(c4)C5CCOCC5)cc1C(=O)N6CCN(CC6)C(=O)C7CC7 | Olaparib 244 |
| CC(C)(NC(=O)c1cccc2C(=NNC(=O)c12)Cc3ccc(F)c(c3)C(=O)N4CCN(CC4)C(=O)C5CC5)c6cn(nn6)c7ccc(Cl)cc7 | Olaparib 210 |
| Cc1cc(ccc1N)c2nc3cc(Oc4cccc5C(=NNC(=O)c45)Cc6ccc(F)c(c6)C(=O)N7CCN(CC7)C(=O)C8CC8)ccc3s2 | Olaparib 2691 |
| Fc1ccc(CC2=NNC(=O)c3c(Oc4nc5cc(Cl)ccc5cc4C=O)cccc23)cc1C(=O)N6CCN(CC6)C(=O)C7CC7 | Olaparib 2552 |
| Fc1ccc(CC2=NNC(=O)c3c(cccc23)C(=O)OCc4cc(ccn4)C#Cc5ccccc5)cc1C(=O)N6CCN(CC6)C(=O)C7CC7 | Olaparib 1043 |
| C[C@@H]1CCCN(C[C@@H](Oc2cccc3C(=NNC(=O)c23)Cc4ccc(F)c(c4)C(=O)N5CCN(CC5)C(=O)C6CC6)C(F)(F)F)CC1 | Olaparib 2861 |
| Fc1ccc(CC2=NNC(=O)c3c(OC(=O)CC4CN(C4)c5cnnc6ccccc56)cccc23)cc1C(=O)N7CCN(CC7)C(=O)C8CC8 | Olaparib 1581 |
| C[C@@H](Cn1cc(c(N)n1)c2cccc3C(=NNC(=O)c23)Cc4ccc(F)c(c4)C(=O)N5CCN(CC5)C(=O)C6CC6)C(=O)N | Olaparib 1682 |
| Fc1ccc(CC2=NNC(=O)c3c(Oc4ccc(OCCn5cnc6ccccc56)cc4)cccc23)cc1C(=O)N7CCN(CC7)C(=O)C8CC8 | Olaparib 2648 |
| Fc1ccc(CC2=NNC(=O)c3c(cccc23)C(=O)NCC=C4SCCS4)cc1C(=O)N5CCN(CC5)C(=O)C6CC6 | Olaparib 188 |
| NC1(CC1)c2cc(F)c(F)c(OC(=O)c3cccc4C(=NNC(=O)c34)Cc5ccc(F)c(c5)C(=O)N6CCN(CC6)C(=O)C7CC7)c2F | Olaparib 1052 |
| N[C@@]1([C@@H]2CC[C@H]1C[C@@H](C2)C(=O)O)C(=O)Oc3cccc4C(=NNC(=O)c34)Cc5ccc(F)c(c5)C(=O)N6CCN(CC6)C(=O)C7CC7 | Olaparib 1553 |
| C[C@@H](NC(=O)c1c[nH]c2ccccc12)C(=O)Oc3cccc4C(=NNC(=O)c34)Cc5ccc(F)c(c5)C(=O)N6CCN(CC6)C(=O)C7CC7 | Olaparib 1538 |
| Oc1cc2[nH]c3cc(Oc4cccc5C(=NNC(=O)c45)Cc6ccc(F)c(c6)C(=O)N7CCN(CC7)C(=O)C8CC8)ccc3c2cc1C=O | Olaparib 3189 |
| Fc1ccc(CC2=NNC(=O)c3c(Oc4ccc5nc(Cl)c(C=O)cc5c4)cccc23)cc1C(=O)N6CCN(CC6)C(=O)C7CC7 | Olaparib 2455 |
| Oc1cccc(c1)c2ccc(OC(=O)c3cccc4C(=NNC(=O)c34)Cc5ccc(F)c(c5)C(=O)N6CCN(CC6)C(=O)C7CC7)cc2 | Olaparib 1079 |
| Cc1nccc(c1C)c2cccc3C(=NNC(=O)c23)Cc4ccc(F)c(c4)C(=O)N5CCN(CC5)C(=O)C6CC6 | Olaparib 2007 |
| Nc1nc(nc(n1)c2cccc(Oc3cccc4C(=NNC(=O)c34)Cc5ccc(F)c(c5)C(=O)N6CCN(CC6)C(=O)C7CC7)c2)C8CC8 | Olaparib 2715 |
| CCOc1nc(Cl)nc(Oc2cccc3C(=NNC(=O)c23)Cc4ccc(F)c(c4)C(=O)N5CCN(CC5)C(=O)C6CC6)n1 | Olaparib 2324 |
| Nc1ccc(cc1Oc2cccc3C(=NNC(=O)c23)Cc4ccc(F)c(c4)C(=O)N5CCN(CC5)C(=O)C6CC6)c7nc8cccc(Cl)c8[nH]7 | Olaparib 2632 |
| Cc1ccc(cc1)C(=O)\C=C\C(=O)Oc2cccc3C(=NNC(=O)c23)Cc4ccc(F)c(c4)C(=O)N5CCN(CC5)C(=O)C6CC6 | Olaparib 1465 |
| OC(=O)C1(CC1)c2ccc(Oc3cccc4C(=NNC(=O)c34)Cc5ccc(F)c(c5)C(=O)N6CCN(CC6)C(=O)C7CC7)c(Br)c2 | Olaparib 2481 |
| CC(C)(C)OC(=O)N1CC[C@H](C1)[C@@H]2C[C@@H](CCN2)Oc3cccc4C(=NNC(=O)c34)Cc5ccc(F)c(c5)C(=O)N6CCN(CC6)C(=O)C7CC7 | Olaparib 2450 |
| COC(=O)c1cc(OC)c2cc(F)c(Oc3cccc4C(=NNC(=O)c34)Cc5ccc(F)c(c5)C(=O)N6CCN(CC6)C(=O)C7CC7)cc2n1 | Olaparib 2501 |
| CC(=O)N(O)c1ccc2c(Cc3cc(Oc4cccc5C(=NNC(=O)c45)Cc6ccc(F)c(c6)C(=O)N7CCN(CC7)C(=O)C8CC8)ccc23)c1 | Olaparib 2699 |
| CC(C)(NC(=O)c1cccc2C(=NNC(=O)c12)Cc3ccc(F)c(c3)C(=O)N4CCN(CC4)C(=O)C5CC5)c6ccc7cc[nH]c7c6 | Olaparib 246 |
| C[C@@H](N)c1cc(Oc2cccc3C(=NNC(=O)c23)Cc4ccc(F)c(c4)C(=O)N5CCN(CC5)C(=O)C6CC6)ccc1OCc7ccccc7F | Olaparib 2476 |
| FC(F)Cn1cccc1c2cccc3C(=NNC(=O)c23)Cc4ccc(F)c(c4)C(=O)N5CCN(CC5)C(=O)C6CC6 | Olaparib 1973 |
| Cn1c(NC2(COC(=O)c3cccc4C(=NNC(=O)c34)Cc5ccc(F)c(c5)C(=O)N6CCN(CC6)C(=O)C7CC7)CCCC2)nc8ccccc18 | Olaparib 1128 |
| CC1=C(C(=O)O)C(=O)N(C[C@@H](F)Oc2cccc3C(=NNC(=O)c23)Cc4ccc(F)c(c4)C(=O)N5CCN(CC5)C(=O)C6CC6)C(=C1)C | Olaparib 2236 |
| Fc1ccc(CC2=NNC(=O)c3c(cccc23)C(=O)OCCN4c5ccccc5Oc6ccccc46)cc1C(=O)N7CCN(CC7)C(=O)C8CC8 | Olaparib 1153 |
| Cc1cc(O)c(cc1Br)C(C)(C)NC(=O)c2cccc3C(=NNC(=O)c23)Cc4ccc(F)c(c4)C(=O)N5CCN(CC5)C(=O)C6CC6 | Olaparib 128 |
| Fc1ccc(CC2=NNC(=O)c3c(Oc4cc(ccc4Br)[C@@H]5CCC(=O)N5)cccc23)cc1C(=O)N6CCN(CC6)C(=O)C7CC7 | Olaparib 2484 |
| COc1cccc2c(OC(=O)c3cccc4C(=NNC(=O)c34)Cc5ccc(F)c(c5)C(=O)N6CCN(CC6)C(=O)C7CC7)c(ccc12)C(=O)C | Olaparib 1031 |
| Oc1nc(OC(=O)c2cccc3C(=NNC(=O)c23)Cc4ccc(F)c(c4)C(=O)N5CCN(CC5)C(=O)C6CC6)c7ccccc7n1 | Olaparib 1104 |
| Oc1ccc2C(=O)c3ccc(Oc4cccc5C(=NNC(=O)c45)Cc6ccc(F)c(c6)C(=O)N7CCN(CC7)C(=O)C8CC8)cc3Oc2c1 | Olaparib 3185 |
| NCc1cc(Oc2cccc3C(=NNC(=O)c23)Cc4ccc(F)c(c4)C(=O)N5CCN(CC5)C(=O)C6CC6)nc7ccccc17 | Olaparib 2607 |
| C[C@H](COc1cccc2ccccc12)OC(=O)c3cccc4C(=NNC(=O)c34)Cc5ccc(F)c(c5)C(=O)N6CCN(CC6)C(=O)C7CC7 | Olaparib 1053 |
| Fc1ccc(CC2=NNC(=O)c3c(cccc23)C(=O)N\C=C\C(=O)C(F)(F)F)cc1C(=O)N4CCN(CC4)C(=O)C5CC5 | Olaparib 13 |
| Cc1cc(ccc1Oc2cccc3C(=NNC(=O)c23)Cc4ccc(F)c(c4)C(=O)N5CCN(CC5)C(=O)C6CC6)c7onc(n7)[C@@H]8CCCNC8 | Olaparib 2677 |
| OC(=O)C1=CC=CN(C[C@@H](F)Oc2cccc3C(=NNC(=O)c23)Cc4ccc(F)c(c4)C(=O)N5CCN(CC5)C(=O)C6CC6)C1=O | Olaparib 2304 |
| CNc1cc(nc(n1)c2cc(Oc3cccc4C(=NNC(=O)c34)Cc5ccc(F)c(c5)C(=O)N6CCN(CC6)C(=O)C7CC7)ccc2C)C8CC8 | Olaparib 2695 |
| Cc1ccc(cc1O)C(C)(C)C(=O)Oc2cccc3C(=NNC(=O)c23)Cc4ccc(F)c(c4)C(=O)N5CCN(CC5)C(=O)C6CC6 | Olaparib 1459 |
| Fc1ccc(CC2=NNC(=O)c3c(Oc4ccc(cc4F)[C@@H]5CCC(=O)N5)cccc23)cc1C(=O)N6CCN(CC6)C(=O)C7CC7 | Olaparib 2485 |
| Fc1ccc(CC2=NNC(=O)c3c(OC(=O)Cc4ccc(nc4Cl)c5ccncc5)cccc23)cc1C(=O)N6CCN(CC6)C(=O)C7CC7 | Olaparib 1525 |
| Cc1cccnc1CNCc2ncc(Oc3cccc4C(=NNC(=O)c34)Cc5ccc(F)c(c5)C(=O)N6CCN(CC6)C(=O)C7CC7)n2C | Olaparib 2505 |
| CNc1cc(nc(n1)c2cccc(Oc3cccc4C(=NNC(=O)c34)Cc5ccc(F)c(c5)C(=O)N6CCN(CC6)C(=O)C7CC7)c2)C8CC8 | Olaparib 2686 |
| Fc1ccc(CC2=NNC(=O)c3c(Oc4ccc5ccnc(CC#N)c5c4)cccc23)cc1C(=O)N6CCN(CC6)C(=O)C7CC7 | Olaparib 3116 |
| C[C@H](C(=O)Nc1cccc2C(=NNC(=O)c12)Cc3ccc(F)c(c3)C(=O)N4CCN(CC4)C(=O)C5CC5)c6ccc7cc(O)ccc7c6 | Olaparib 534 |
| COC(=O)CC(=O)C(F)(F)c1cccc2C(=NNC(=O)c12)Cc3ccc(F)c(c3)C(=O)N4CCN(CC4)C(=O)C5CC5 | Olaparib 1610 |
| Nc1ccc2[nH]cc(C(=O)N3CC[C@H](C3)Oc4cccc5C(=NNC(=O)c45)Cc6ccc(F)c(c6)C(=O)N7CCN(CC7)C(=O)C8CC8)c2c1 | Olaparib 3158 |
| CN(CC(=O)Oc1cccc2C(=NNC(=O)c12)Cc3ccc(F)c(c3)C(=O)N4CCN(CC4)C(=O)C5CC5)C(=O)[C@H]6CCSC6 | Olaparib 1448 |
| Cc1c(Oc2cccc3C(=NNC(=O)c23)Cc4ccc(F)c(c4)C(=O)N5CCN(CC5)C(=O)C6CC6)cccc1c7oc(cc7)[C@@H]8C[C@H]8C(=O)O | Olaparib 2683 |
| COc1c(F)c(F)cc(c1F)C(C)(C)NC(=O)c2cccc3C(=NNC(=O)c23)Cc4ccc(F)c(c4)C(=O)N5CCN(CC5)C(=O)C6CC6 | Olaparib 135 |
| Fc1cc([C@H]2CCC(=O)N2)c(Oc3cccc4C(=NNC(=O)c34)Cc5ccc(F)c(c5)C(=O)N6CCN(CC6)C(=O)C7CC7)cc1Cl | Olaparib 2516 |
| C[C@H](NC(=O)c1cccc2C(=NNC(=O)c12)Cc3ccc(F)c(c3)C(=O)N4CCN(CC4)C(=O)C5CC5)c6ccc(cc6)n7nc(C)c(C)c7C | Olaparib 217 |
| O[C@@H](Cc1onc(n1)c2ccc(Oc3cccc4C(=NNC(=O)c34)Cc5ccc(F)c(c5)C(=O)N6CCN(CC6)C(=O)C7CC7)c(F)c2)C(F)F | Olaparib 2440 |
| Fc1ccc(CC2=NNC(=O)c3c(Oc4ccc(CN5CCNCc6ccccc56)cn4)cccc23)cc1C(=O)N7CCN(CC7)C(=O)C8CC8 | Olaparib 2643 |
| Nc1cnc2cccnc2c1c3cccc4C(=NNC(=O)c34)Cc5ccc(F)c(c5)C(=O)N6CCN(CC6)C(=O)C7CC7 | Olaparib 1773 |
| CC[n+]1ccc2c(OC(=O)c3cccc4C(=NNC(=O)c34)Cc5ccc(F)c(c5)C(=O)N6CCN(CC6)C(=O)C7CC7)cccc2c1 | Olaparib 1096 |
| Fc1ccc(CC2=NNC(=O)c3c(cccc23)C(=O)NC(=N)C(F)(F)F)cc1C(=O)N4CCN(CC4)C(=O)C5CC5 | Olaparib 85 |
| Fc1ccc(CC2=NNC(=O)c3c(Oc4ccc5C(=O)C(=O)N(CC(=O)C6CC6)c5c4)cccc23)cc1C(=O)N7CCN(CC7)C(=O)C8CC8 | Olaparib 2714 |
| O[C@@H]1NC(=O)NC(=O)[C@H]1c2cccc3C(=NNC(=O)c23)Cc4ccc(F)c(c4)C(=O)N5CCN(CC5)C(=O)C6CC6 | Olaparib 1725 |
| CC(C)(C(=O)Oc1cccc2C(=NNC(=O)c12)Cc3ccc(F)c(c3)C(=O)N4CCN(CC4)C(=O)C5CC5)c6cc(Cl)ccc6O | Olaparib 1433 |
| Fc1cc([C@H]2CCC(=O)N2)c(Cl)cc1Oc3cccc4C(=NNC(=O)c34)Cc5ccc(F)c(c5)C(=O)N6CCN(CC6)C(=O)C7CC7 | Olaparib 2518 |
| NCc1ncccc1COc2ccc(F)c(Oc3cccc4C(=NNC(=O)c34)Cc5ccc(F)c(c5)C(=O)N6CCN(CC6)C(=O)C7CC7)c2 | Olaparib 2468 |
| C[C@@H](C(=O)Nc1cccc2C(=NNC(=O)c12)Cc3ccc(F)c(c3)C(=O)N4CCN(CC4)C(=O)C5CC5)n6nnc7ccccc67 | Olaparib 543 |
| Oc1c(ccc2ccccc12)C(=O)N3CC(C3)Oc4cccc5C(=NNC(=O)c45)Cc6ccc(F)c(c6)C(=O)N7CCN(CC7)C(=O)C8CC8 | Olaparib 3162 |
| Oc1cc(Oc2cccc3C(=NNC(=O)c23)Cc4ccc(F)c(c4)C(=O)N5CCN(CC5)C(=O)C6CC6)nc7ccccc17 | Olaparib 3137 |
| OCc1cccc2cccc(COC(=O)c3cccc4C(=NNC(=O)c34)Cc5ccc(F)c(c5)C(=O)N6CCN(CC6)C(=O)C7CC7)c12 | Olaparib 1075 |
| CC[C@@H](C)[C@@H](N)C(=O)Nc1cccc2C(=NNC(=O)c12)Cc3ccc(F)c(c3)C(=O)N4CCN(CC4)C(=O)C5CC5 | Olaparib 348 |
| Fc1ccc(CC2=NNC(=O)c3c(cccc23)C(=O)OC[C@@H]4CCN(C4)C(=O)c5ccc6COCc6c5)cc1C(=O)N7CCN(CC7)C(=O)C8CC8 | Olaparib 1124 |
| Fc1ccc(CC2=NNC(=O)c3c(NC(=O)CNC(=O)c4ccccc4Cl)cccc23)cc1C(=O)N5CCN(CC5)C(=O)C6CC6 | Olaparib 471 |
| C[C@H](CSc1cccc(Br)c1)NC(=O)c2cccc3C(=NNC(=O)c23)Cc4ccc(F)c(c4)C(=O)N5CCN(CC5)C(=O)C6CC6 | Olaparib 127 |
| Fc1ccc(CC2=NNC(=O)c3c(Oc4cc(ccc4Cl)[C@@H]5CCC(=O)N5)cccc23)cc1C(=O)N6CCN(CC6)C(=O)C7CC7 | Olaparib 2577 |
| O[C@@H]([C@H](O)c1cccc2ccccc12)C(=O)Oc3cccc4C(=NNC(=O)c34)Cc5ccc(F)c(c5)C(=O)N6CCN(CC6)C(=O)C7CC7 | Olaparib 1539 |
| C[C@@H](C(=O)Oc1cccc2C(=NNC(=O)c12)Cc3ccc(F)c(c3)C(=O)N4CCN(CC4)C(=O)C5CC5)n6nnc7ccccc67 | Olaparib 1566 |
| OC(=O)[C@H]1C[C@@H]1c2oc(cc2)c3ccc(Oc4cccc5C(=NNC(=O)c45)Cc6ccc(F)c(c6)C(=O)N7CCN(CC7)C(=O)C8CC8)cc3 | Olaparib 2717 |
| CNCCc1c[nH]c2ccc(Oc3cccc4C(=NNC(=O)c34)Cc5ccc(F)c(c5)C(=O)N6CCN(CC6)C(=O)C7CC7)cc12 | Olaparib 3111 |
| O[C@H](C(=O)Nc1cccc2C(=NNC(=O)c12)Cc3ccc(F)c(c3)C(=O)N4CCN(CC4)C(=O)C5CC5)c6ccc7ccccc7c6 | Olaparib 540 |
| CC[C@@H](C)[C@@H](N)C(=O)Nc1cccc2C(=NNC(=O)c12)Cc3ccc(F)c(c3)C(=O)N4CCN(CC4)C(=O)C5CC5 | Olaparib 350 |
| Fc1ccc(CC2=NNC(=O)c3c(Oc4ccc5OC=C(C6OCCO6)C(=O)c5c4)cccc23)cc1C(=O)N7CCN(CC7)C(=O)C8CC8 | Olaparib 2707 |
| OC(=O)[C@H](NC(=O)c1cccc2C(=NNC(=O)c12)Cc3ccc(F)c(c3)C(=O)N4CCN(CC4)C(=O)C5CC5)c6ccc(F)cc6 | Olaparib 166 |
| C[C@@H](COc1ccccc1)NC(=O)c2cccc3C(=NNC(=O)c23)Cc4ccc(F)c(c4)C(=O)N5CCN(CC5)C(=O)C6CC6 | Olaparib 184 |
| CN1CCC[C@@H](N)[C@@H]1c2ccc(Cl)c(Oc3cccc4C(=NNC(=O)c34)Cc5ccc(F)c(c5)C(=O)N6CCN(CC6)C(=O)C7CC7)c2 | Olaparib 2526 |
| Fc1ccc(CC2=NNC(=O)c3c(cccc23)C(=O)OCc4nc(c[nH]4)c5cc(Cl)ccc5Cl)cc1C(=O)N6CCN(CC6)C(=O)C7CC7 | Olaparib 994 |
| CCOC(=O)C1=CNc2cc(Oc3cccc4C(=NNC(=O)c34)Cc5ccc(F)c(c5)C(=O)N6CCN(CC6)C(=O)C7CC7)c(F)cc2C1=O | Olaparib 2499 |
| Cc1cc2cc(ccc2nc1Oc3cccc4C(=NNC(=O)c34)Cc5ccc(F)c(c5)C(=O)N6CCN(CC6)C(=O)C7CC7)N8CCOCC8 | Olaparib 2671 |
| NCCc1c[nH]c2ccc(Oc3cccc4C(=NNC(=O)c34)Cc5ccc(F)c(c5)C(=O)N6CCN(CC6)C(=O)C7CC7)cc12 | Olaparib 3123 |
| Fc1ccc(CC2=NNC(=O)c3c(cccc23)C(=O)OCCOc4ccc5ccccc5c4)cc1C(=O)N6CCN(CC6)C(=O)C7CC7 | Olaparib 1074 |
| C[C@H](CC(=O)N)Oc1cccc2C(=NNC(=O)c12)Cc3ccc(F)c(c3)C(=O)N4CCN(CC4)C(=O)C5CC5 | Olaparib 2850 |
| COc1cccc(CN2[C@@H]3CC[C@H]2CC(C3)OC(=O)c4cccc5C(=NNC(=O)c45)Cc6ccc(F)c(c6)C(=O)N7CCN(CC7)C(=O)C8CC8)n1 | Olaparib 1123 |
| COc1cc(cc(n1)C(=O)O)c2cccc3C(=NNC(=O)c23)Cc4ccc(F)c(c4)C(=O)N5CCN(CC5)C(=O)C6CC6 | Olaparib 1965 |
| O[C@@H](CCNC(=O)c1cccc2C(=NNC(=O)c12)Cc3ccc(F)c(c3)C(=O)N4CCN(CC4)C(=O)C5CC5)c6ccccc6 | Olaparib 186 |
| NC1(CC1)c2ccc(Oc3cccc4C(=NNC(=O)c34)Cc5ccc(F)c(c5)C(=O)N6CCN(CC6)C(=O)C7CC7)cc2CO | Olaparib 2615 |
| CCOC(=O)C1=CNc2cc(F)c(Oc3cccc4C(=NNC(=O)c34)Cc5ccc(F)c(c5)C(=O)N6CCN(CC6)C(=O)C7CC7)cc2C1=O | Olaparib 2498 |
| OC(=O)c1ccc(cc1Oc2cccc3C(=NNC(=O)c23)Cc4ccc(F)c(c4)C(=O)N5CCN(CC5)C(=O)C6CC6)c7cocc7 | Olaparib 2593 |
| Fc1ccc(CC2=NNC(=O)c3c(Oc4ccc(cc4)[C@@H]5CNC[C@H]5c6cc[nH]n6)cccc23)cc1C(=O)N7CCN(CC7)C(=O)C8CC8 | Olaparib 2730 |
| Cn1c(Oc2cccc3C(=NNC(=O)c23)Cc4ccc(F)c(c4)C(=O)N5CCN(CC5)C(=O)C6CC6)nc7ccccc17 | Olaparib 2585 |
| Fc1ccc(CC2=NNC(=O)c3c(cccc23)C(=O)O[C@@H](c4cccc5cccnc45)C(F)(F)F)cc1C(=O)N6CCN(CC6)C(=O)C7CC7 | Olaparib 1012 |
| O[C@H](CC(=O)Nc1cccc2C(=NNC(=O)c12)Cc3ccc(F)c(c3)C(=O)N4CCN(CC4)C(=O)C5CC5)C(F)(F)F | Olaparib 289 |
| Fc1ccc(CC2=NNC(=O)c3c(Oc4ccc(nn4)N5CC[C@H](C5)N6CCCCC6)cccc23)cc1C(=O)N7CCN(CC7)C(=O)C8CC8 | Olaparib 2657 |
| Nc1c(F)c(F)nc(CC(=O)Oc2cccc3C(=NNC(=O)c23)Cc4ccc(F)c(c4)C(=O)N5CCN(CC5)C(=O)C6CC6)c1F | Olaparib 1444 |
| N[C@H](Cc1ccc(Cl)cc1c2cccc3C(=NNC(=O)c23)Cc4ccc(F)c(c4)C(=O)N5CCN(CC5)C(=O)C6CC6)C(=O)O | Olaparib 1663 |
| NC1(CC1)c2cc(Br)c(Oc3cccc4C(=NNC(=O)c34)Cc5ccc(F)c(c5)C(=O)N6CCN(CC6)C(=O)C7CC7)c(F)c2O | Olaparib 2473 |
| Fc1ccc(cc1Oc2cccc3C(=NNC(=O)c23)Cc4ccc(F)c(c4)C(=O)N5CCN(CC5)C(=O)C6CC6)c7c[nH]c(C=O)n7 | Olaparib 2592 |
| Fc1ccc(CC2=NNC(=O)c3c(OC(=O)CN4C(=O)c5ccccc5C4=O)cccc23)cc1C(=O)N6CCN(CC6)C(=O)C7CC7 | Olaparib 1561 |
| Cn1c(nc2ccc(Oc3cccc4C(=NNC(=O)c34)Cc5ccc(F)c(c5)C(=O)N6CCN(CC6)C(=O)C7CC7)cc12)[C@]8(C)CCNC8 | Olaparib 2729 |
| Cc1nc2ccc(Oc3cccc4C(=NNC(=O)c34)Cc5ccc(F)c(c5)C(=O)N6CCN(CC6)C(=O)C7CC7)cc2s1 | Olaparib 3130 |
| Cc1ncc(OC(=O)c2cccc3C(=NNC(=O)c23)Cc4ccc(F)c(c4)C(=O)N5CCN(CC5)C(=O)C6CC6)c7ccccc17 | Olaparib 1113 |
| N[C@H](CF)C(=O)Oc1cccc2C(=NNC(=O)c12)Cc3ccc(F)c(c3)C(=O)N4CCN(CC4)C(=O)C5CC5 | Olaparib 1375 |
| C[C@H](CCN1C(=O)[C@@H]2[C@H]3C[C@H](C=C3)[C@@H]2C1=O)OC(=O)c4cccc5C(=NNC(=O)c45)Cc6ccc(F)c(c6)C(=O)N7CCN(CC7)C(=O)C8CC8 | Olaparib 1146 |
| CCOc1nc(Cl)nc(n1)c2cccc3C(=NNC(=O)c23)Cc4ccc(F)c(c4)C(=O)N5CCN(CC5)C(=O)C6CC6 | Olaparib 1691 |
| Fc1ccc(CC2=NNC(=O)c3c(OCCNC(=O)c4ccc(Cl)cc4)cccc23)cc1C(=O)N5CCN(CC5)C(=O)C6CC6 | Olaparib 2873 |
| Fc1ccc(CC2=NNC(=O)c3c2cccc3c4cnn(c4)C5CCNCC5)cc1C(=O)N6CCN(CC6)C(=O)C7CC7 | Olaparib 2070 |
| C[C@@](O)(CN1CCc2ccccc2C1=O)C(=O)Oc3cccc4C(=NNC(=O)c34)Cc5ccc(F)c(c5)C(=O)N6CCN(CC6)C(=O)C7CC7 | Olaparib 1524 |
| Fc1cc(ccc1Oc2cccc3C(=NNC(=O)c23)Cc4ccc(F)c(c4)C(=O)N5CCN(CC5)C(=O)C6CC6)c7c[nH]c(C=O)n7 | Olaparib 2591 |
| Fc1ccc(CC2=NNC(=O)c3c(OC4C[C@H]5CC[C@@H](C4)N5C(=O)Nc6ccccc6)cccc23)cc1C(=O)N7CCN(CC7)C(=O)C8CC8 | Olaparib 3155 |
| Fc1ccc(CC2=NNC(=O)c3c(Oc4cccc5cn[nH]c45)cccc23)cc1C(=O)N6CCN(CC6)C(=O)C7CC7 | Olaparib 2623 |
| Cc1nc2c(F)cccc2c(OC(=O)c3cccc4C(=NNC(=O)c34)Cc5ccc(F)c(c5)C(=O)N6CCN(CC6)C(=O)C7CC7)c1Cl | Olaparib 1037 |
| O[C@H](C(=O)Nc1cccc2C(=NNC(=O)c12)Cc3ccc(F)c(c3)C(=O)N4CCN(CC4)C(=O)C5CC5)C(F)(F)F | Olaparib 312 |
| OC1C[C@H]2CC[C@@H](C1)N2C(=O)[C@H]3C[C@H](CN3)Oc4cccc5C(=NNC(=O)c45)Cc6ccc(F)c(c6)C(=O)N7CCN(CC7)C(=O)C8CC8 | Olaparib 3169 |
| Fc1ccc(CC2=NNC(=O)c3c(Oc4nc(Cl)n[nH]4)cccc23)cc1C(=O)N5CCN(CC5)C(=O)C6CC6 | Olaparib 2353 |
| Fc1ccc(CC2=NNC(=O)c3c(cccc23)C(=O)NCCSc4ccccc4)cc1C(=O)N5CCN(CC5)C(=O)C6CC6 | Olaparib 183 |
| C[C@H]1CCC[C@@](CC(=O)Nc2cccc3C(=NNC(=O)c23)Cc4ccc(F)c(c4)C(=O)N5CCN(CC5)C(=O)C6CC6)(C1)N7CCCCC7 | Olaparib 523 |
| Fc1ccc(CC2=NNC(=O)c3c(OC(=O)CNC(=O)N4CCc5ccccc5C4)cccc23)cc1C(=O)N6CCN(CC6)C(=O)C7CC7 | Olaparib 1534 |
| Fc1ccc(CC2=NNC(=O)c3c(Oc4nnc(Cc5ccc(cc5)C(F)(F)F)s4)cccc23)cc1C(=O)N6CCN(CC6)C(=O)C7CC7 | Olaparib 2433 |
| Fc1ccc(CC2=NNC(=O)c3c(OC4CN(C4)C(=O)[C@H]5COc6ccccc56)cccc23)cc1C(=O)N7CCN(CC7)C(=O)C8CC8 | Olaparib 3193 |
| Fc1ccc(CC2=NNC(=O)c3c(Oc4cccc5[nH]ncc45)cccc23)cc1C(=O)N6CCN(CC6)C(=O)C7CC7 | Olaparib 2622 |
| Fc1ccc(CC2=NNC(=O)c3c(cccc23)C(=O)OCC#Cc4ccc5ccccc5n4)cc1C(=O)N6CCN(CC6)C(=O)C7CC7 | Olaparib 1081 |
| OCC(CO)COc1cccc2C(=NNC(=O)c12)Cc3ccc(F)c(c3)C(=O)N4CCN(CC4)C(=O)C5CC5 | Olaparib 2819 |
| Fc1ccc(CC2=NNC(=O)c3c2cccc3C(F)(F)C4=CC(=O)NC(=O)N4)cc1C(=O)N5CCN(CC5)C(=O)C6CC6 | Olaparib 1705 |
| Fc1ccc(cc1)c2oc(nn2)c3ccc(Oc4cccc5C(=NNC(=O)c45)Cc6ccc(F)c(c6)C(=O)N7CCN(CC7)C(=O)C8CC8)cc3 | Olaparib 2692 |
| Fc1ccc(CC2=NNC(=O)c3c(cccc23)C(=O)NCCOc4ccccc4)cc1C(=O)N5CCN(CC5)C(=O)C6CC6 | Olaparib 194 |
| OC(=O)Cn1c2ccccc2c3cc(Oc4cccc5C(=NNC(=O)c45)Cc6ccc(F)c(c6)C(=O)N7CCN(CC7)C(=O)C8CC8)ccc13 | Olaparib 2687 |
| C[C@]1(CCC(=O)Oc2cccc3C(=NNC(=O)c23)Cc4ccc(F)c(c4)C(=O)N5CCN(CC5)C(=O)C6CC6)[C@H]7CC[C@]1(C)[C@H](N)C7 | Olaparib 1555 |
| Fc1ccc(CC2=NNC(=O)c3c(cccc23)C(=O)Oc4c(F)cc(cc4[C@H]5CCC(=O)N5)C#N)cc1C(=O)N6CCN(CC6)C(=O)C7CC7 | Olaparib 1027 |
| Cc1cnn(c1)c2nccc(n2)c3cccc4C(=NNC(=O)c34)Cc5ccc(F)c(c5)C(=O)N6CCN(CC6)C(=O)C7CC7 | Olaparib 2064 |
| COc1cccc(CN2[C@@H]3CC[C@H]2CC(C3)Oc4cccc5C(=NNC(=O)c45)Cc6ccc(F)c(c6)C(=O)N7CCN(CC7)C(=O)C8CC8)n1 | Olaparib 3149 |
| Fc1ccc(CC2=NNC(=O)c3c2cccc3c4cccc5nncn45)cc1C(=O)N6CCN(CC6)C(=O)C7CC7 | Olaparib 1776 |
| Fc1ccc(CC2=NNC(=O)c3c(Oc4ccc(nn4)c5ccccc5)cccc23)cc1C(=O)N6CCN(CC6)C(=O)C7CC7 | Olaparib 2610 |
| CCOC(=O)\C=C(/C)\C(F)(F)c1cccc2C(=NNC(=O)c12)Cc3ccc(F)c(c3)C(=O)N4CCN(CC4)C(=O)C5CC5 | Olaparib 1606 |
| Fc1ccc(CC2=NNC(=O)c3c(Oc4ccc(cc4)c5cn6cccnc6n5)cccc23)cc1C(=O)N7CCN(CC7)C(=O)C8CC8 | Olaparib 2641 |
| Fc1ccc(CC2=NNC(=O)c3c2cccc3C4=CC(=O)NC(=O)N4)cc1C(=O)N5CCN(CC5)C(=O)C6CC6 | Olaparib 1656 |
| F[C@@H](N1N=NN(C1=S)c2ccccc2)c3cccc4C(=NNC(=O)c34)Cc5ccc(F)c(c5)C(=O)N6CCN(CC6)C(=O)C7CC7 | Olaparib 1754 |
| N[C@@H](C(=O)Oc1cccc2C(=NNC(=O)c12)Cc3ccc(F)c(c3)C(=O)N4CCN(CC4)C(=O)C5CC5)c6ccc(Br)c(F)c6 | Olaparib 1403 |
| CC[C@H]1C[C@]2(CCO1)C[C@@H]2C(=O)Oc3cccc4C(=NNC(=O)c34)Cc5ccc(F)c(c5)C(=O)N6CCN(CC6)C(=O)C7CC7 | Olaparib 1569 |
| Fc1ccc(CC2=NNC(=O)c3c(Oc4nc(n[nH]4)c5cccc(Cl)c5)cccc23)cc1C(=O)N6CCN(CC6)C(=O)C7CC7 | Olaparib 2575 |
| Fc1ccc(CC2=NNC(=O)c3c(O[C@H]4CCO[C@H](C4)c5ccc6OCCOc6c5)cccc23)cc1C(=O)N7CCN(CC7)C(=O)C8CC8 | Olaparib 3175 |
| Fc1ccc(CC2=NNC(=O)c3c(Oc4cncc5c(Br)coc45)cccc23)cc1C(=O)N6CCN(CC6)C(=O)C7CC7 | Olaparib 3086 |
| Oc1ccc2c(OC[C@]2(O)CC(=O)Nc3cccc4C(=NNC(=O)c34)Cc5ccc(F)c(c5)C(=O)N6CCN(CC6)C(=O)C7CC7)c1 | Olaparib 537 |
| Fc1ccc(CC2=NNC(=O)c3c(Oc4ccc5cc(C=O)c(Cl)nc5c4)cccc23)cc1C(=O)N6CCN(CC6)C(=O)C7CC7 | Olaparib 2553 |
| CCOC(=O)\C=C\C(F)(F)c1cccc2C(=NNC(=O)c12)Cc3ccc(F)c(c3)C(=O)N4CCN(CC4)C(=O)C5CC5 | Olaparib 1614 |
| Fc1ccc(CC2=NNC(=O)c3c(Oc4c(Cl)cccc4c5nc6ccncc6[nH]5)cccc23)cc1C(=O)N7CCN(CC7)C(=O)C8CC8 | Olaparib 2667 |
| CC(C)CC1=NO[C@@H](CNC(=O)c2cccc3C(=NNC(=O)c23)Cc4ccc(F)c(c4)C(=O)N5CCN(CC5)C(=O)C6CC6)C1 | Olaparib 181 |
| CCOC(=O)c1cc(ccn1)c2cccc3C(=NNC(=O)c23)Cc4ccc(F)c(c4)C(=O)N5CCN(CC5)C(=O)C6CC6 | Olaparib 1966 |
| N[C@@H]1[C@@H]2CC[C@@H](C2)[C@@H]1C(=O)Oc3cccc4C(=NNC(=O)c34)Cc5ccc(F)c(c5)C(=O)N6CCN(CC6)C(=O)C7CC7 | Olaparib 1573 |
| CC(C)(NC(=O)c1cccc2C(=NNC(=O)c12)Cc3ccc(F)c(c3)C(=O)N4CCN(CC4)C(=O)C5CC5)c6cn(nn6)C7CCCCC7 | Olaparib 233 |
| Nc1ccc(cc1Cl)c2nc3cccc(c4cccc5C(=NNC(=O)c45)Cc6ccc(F)c(c6)C(=O)N7CCN(CC7)C(=O)C8CC8)c3[nH]2 | Olaparib 1780 |
| Fc1ccc(CC2=NNC(=O)c3c2cccc3c4ccnc(n4)n5cccn5)cc1C(=O)N6CCN(CC6)C(=O)C7CC7 | Olaparib 2072 |
| Fc1ccc(CC2=NNC(=O)c3c(OC4=Cc5ccccc5NC4=O)cccc23)cc1C(=O)N6CCN(CC6)C(=O)C7CC7 | Olaparib 3138 |
| Fc1ccc(CC2=NNC(=O)c3c(cccc23)C(=O)Oc4cccc5cnccc45)cc1C(=O)N6CCN(CC6)C(=O)C7CC7 | Olaparib 1117 |
| Fc1ccc(CC2=NNC(=O)c3c(NC(=O)C(=C)C(F)(F)F)cccc23)cc1C(=O)N4CCN(CC4)C(=O)C5CC5 | Olaparib 323 |
| Fc1ccc(CC2=NNC(=O)c3c(cccc23)C(=O)Oc4ccc5C(=O)C(=COc5c4)c6ccccc6)cc1C(=O)N7CCN(CC7)C(=O)C8CC8 | Olaparib 1144 |
| OC[C@@H]1NC[C@@H](Oc2cccc3C(=NNC(=O)c23)Cc4ccc(F)c(c4)C(=O)N5CCN(CC5)C(=O)C6CC6)[C@H]1O | Olaparib 3010 |
| OC(=O)Cc1ccc(F)c(Oc2cccc3C(=NNC(=O)c23)Cc4ccc(F)c(c4)C(=O)N5CCN(CC5)C(=O)C6CC6)c1 | Olaparib 2378 |
| CC(C)[C@H]1C[C@H](CCO1)N[C@@H]2CC[C@H](CC2)Oc3cccc4C(=NNC(=O)c34)Cc5ccc(F)c(c5)C(=O)N6CCN(CC6)C(=O)C7CC7 | Olaparib 3060 |
| C[C@@H]1CCCCCN1Cc2onc(CNC(=O)c3cccc4C(=NNC(=O)c34)Cc5ccc(F)c(c5)C(=O)N6CCN(CC6)C(=O)C7CC7)c2 | Olaparib 226 |
| Fc1ccc(CC2=NNC(=O)c3c(Oc4ccc(cc4Cl)c5cn6ccccc6n5)cccc23)cc1C(=O)N7CCN(CC7)C(=O)C8CC8 | Olaparib 2670 |
| Cc1ccc(C)c(c1)C2=NO[C@@](C)(C2)C(=O)Oc3cccc4C(=NNC(=O)c34)Cc5ccc(F)c(c5)C(=O)N6CCN(CC6)C(=O)C7CC7 | Olaparib 1536 |
| Fc1ccc(CC2=NNC(=O)c3c(NC(=O)CN4CCC(CC4)c5ccccc5)cccc23)cc1C(=O)N6CCN(CC6)C(=O)C7CC7 | Olaparib 533 |
| Fc1ccc(CC2=NNC(=O)c3c(cccc23)C(=O)OCCn4ccc5ccccc45)cc1C(=O)N6CCN(CC6)C(=O)C7CC7 | Olaparib 1108 |
| Fc1ccc(CC2=NNC(=O)c3c(NC(=O)\C=C\C(F)(F)F)cccc23)cc1C(=O)N4CCN(CC4)C(=O)C5CC5 | Olaparib 324 |
| Fc1ccc(CC2=NNC(=O)c3c(cccc23)C(=O)Oc4ccc5O[C@@H](CC(=O)c5c4)c6ccccc6)cc1C(=O)N7CCN(CC7)C(=O)C8CC8 | Olaparib 1141 |
| C[C@H](N)C(=O)NCC(=O)Nc1cccc2C(=NNC(=O)c12)Cc3ccc(F)c(c3)C(=O)N4CCN(CC4)C(=O)C5CC5 | Olaparib 300 |
| Fc1ccc(CC2=NNC(=O)c3c(Oc4ccc(NC(=O)C(F)(F)F)cc4F)cccc23)cc1C(=O)N5CCN(CC5)C(=O)C6CC6 | Olaparib 2864 |
| CC[C@H]1C[C@]2(CCO1)C[C@H](CCO2)Oc3cccc4C(=NNC(=O)c34)Cc5ccc(F)c(c5)C(=O)N6CCN(CC6)C(=O)C7CC7 | Olaparib 3100 |
| Cc1c(C)c(Oc2cccc3C(=NNC(=O)c23)Cc4ccc(F)c(c4)C(=O)N5CCN(CC5)C(=O)C6CC6)nnc1NCCN7CCCC7 | Olaparib 2493 |
| Fc1ccc(CC2=NNC(=O)c3c(Oc4ccc(cc4F)N5C(=O)CCCCC5=O)cccc23)cc1C(=O)N6CCN(CC6)C(=O)C7CC7 | Olaparib 3065 |
| Fc1ccc2CN(C(=N)c2c1)c3ccc(Oc4cccc5C(=NNC(=O)c45)Cc6ccc(F)c(c6)C(=O)N7CCN(CC7)C(=O)C8CC8)c(Cl)c3 | Olaparib 2630 |
| Fc1ccc(CC2=NNC(=O)c3c(OC(=O)CN4CCC(CC4)c5ccccc5)cccc23)cc1C(=O)N6CCN(CC6)C(=O)C7CC7 | Olaparib 1549 |
| Nc1cc(Oc2cccc3C(=NNC(=O)c23)Cc4ccc(F)c(c4)C(=O)N5CCN(CC5)C(=O)C6CC6)ccc1n7cccc7 | Olaparib 2608 |
| COC(=O)\C(=C(\F)/c1cccc2C(=NNC(=O)c12)Cc3ccc(F)c(c3)C(=O)N4CCN(CC4)C(=O)C5CC5)\F | Olaparib 1635 |
| Fc1ccc(CC2=NNC(=O)c3c(Oc4ccc(cc4)C5=NN(C(=O)C5)c6ccccc6)cccc23)cc1C(=O)N7CCN(CC7)C(=O)C8CC8 | Olaparib 2653 |
| F[C@@H](Oc1cccc2C(=NNC(=O)c12)Cc3ccc(F)c(c3)C(=O)N4CCN(CC4)C(=O)C5CC5)S(=O)(=O)NCCC#C | Olaparib 2117 |
| OC(=O)Cc1cc(Oc2cccc3C(=NNC(=O)c23)Cc4ccc(F)c(c4)C(=O)N5CCN(CC5)C(=O)C6CC6)c(F)cc1F | Olaparib 2335 |
| Cc1ccc(cn1)[C@@H](O)c2cccc(c2)C(F)(F)c3cccc4C(=NNC(=O)c34)Cc5ccc(F)c(c5)C(=O)N6CCN(CC6)C(=O)C7CC7 | Olaparib 1740 |
| Fc1ccc(CC2=NNC(=O)c3c(cccc23)C(=O)NCc4nc5CCOCc5[nH]4)cc1C(=O)N6CCN(CC6)C(=O)C7CC7 | Olaparib 249 |
| Fc1ccc(cc1Cl)N2Cc3ccc(Oc4cccc5C(=NNC(=O)c45)Cc6ccc(F)c(c6)C(=O)N7CCN(CC7)C(=O)C8CC8)cc3C2=N | Olaparib 2631 |
| CC1(C)CCN(Cc2ccnc(CNC(=O)c3cccc4C(=NNC(=O)c34)Cc5ccc(F)c(c5)C(=O)N6CCN(CC6)C(=O)C7CC7)c2)CC1 | Olaparib 213 |
| Fc1ccc(CC2=NNC(=O)c3c(cccc23)C(=O)O[C@@]4(CCCOC4)c5ccccc5C(F)(F)F)cc1C(=O)N6CCN(CC6)C(=O)C7CC7 | Olaparib 992 |
| Cc1nc2ccc(Oc3cccc4C(=NNC(=O)c34)Cc5ccc(F)c(c5)C(=O)N6CCN(CC6)C(=O)C7CC7)cc2nc1C | Olaparib 2606 |
| CC[C@@H](C)[C@@H](F)C(=O)Oc1cccc2C(=NNC(=O)c12)Cc3ccc(F)c(c3)C(=O)N4CCN(CC4)C(=O)C5CC5 | Olaparib 1259 |
| Fc1ccc(CC2=NNC(=O)c3c(Oc4ccc(cc4)N5N=C(CC5=O)c6ccccc6)cccc23)cc1C(=O)N7CCN(CC7)C(=O)C8CC8 | Olaparib 2654 |
| CCOC(=O)[C@H](NC(=O)c1cccc2C(=NNC(=O)c12)Cc3ccc(F)c(c3)C(=O)N4CCN(CC4)C(=O)C5CC5)C#N | Olaparib 36 |
| OC(=O)c1cc(Oc2cccc3C(=NNC(=O)c23)Cc4ccc(F)c(c4)C(=O)N5CCN(CC5)C(=O)C6CC6)c(F)c(F)c1F | Olaparib 2194 |
| NCc1ccc(cc1Oc2cccc3C(=NNC(=O)c23)Cc4ccc(F)c(c4)C(=O)N5CCN(CC5)C(=O)C6CC6)c7c(F)cccc7F | Olaparib 2537 |
| NCCC1CCN(Cc2nnsc2c3cccc4C(=NNC(=O)c34)Cc5ccc(F)c(c5)C(=O)N6CCN(CC6)C(=O)C7CC7)CC1 | Olaparib 1742 |
| Cc1ccc(CN[C@@H]2COc3ccccc23)cc1Oc4cccc5C(=NNC(=O)c45)Cc6ccc(F)c(c6)C(=O)N7CCN(CC7)C(=O)C8CC8 | Olaparib 2696 |
| C[C@H]1CC[C@H](CNC(=O)c2cccc3C(=NNC(=O)c23)Cc4ccc(F)c(c4)C(=O)N5CCN(CC5)C(=O)C6CC6)CN1Cc7ccc(cc7)C#N | Olaparib 204 |
| NC1(CC1)c2ccc(cc2COC(=O)c3cccc4C(=NNC(=O)c34)Cc5ccc(F)c(c5)C(=O)N6CCN(CC6)C(=O)C7CC7)C(F)(F)F | Olaparib 1007 |
| Fc1ccc(CC2=NNC(=O)c3c(cccc23)C(=O)OCCc4cc5ccccc5[nH]4)cc1C(=O)N6CCN(CC6)C(=O)C7CC7 | Olaparib 1107 |
| CC(C)(CC(F)(F)F)C(=O)Oc1cccc2C(=NNC(=O)c12)Cc3ccc(F)c(c3)C(=O)N4CCN(CC4)C(=O)C5CC5 | Olaparib 1188 |
| Oc1ccc2C(=O)C=C(Oc2c1)c3ccc(Oc4cccc5C(=NNC(=O)c45)Cc6ccc(F)c(c6)C(=O)N7CCN(CC7)C(=O)C8CC8)cc3 | Olaparib 2649 |
| CNS(=O)(=O)C(F)(F)Oc1cccc2C(=NNC(=O)c12)Cc3ccc(F)c(c3)C(=O)N4CCN(CC4)C(=O)C5CC5 | Olaparib 2139 |
| Fc1ccc(CC2=NNC(=O)c3c(OC(=O)Cc4ccccn4)cccc23)cc1C(=O)N5CCN(CC5)C(=O)C6CC6 | Olaparib 1511 |
| Fc1ccc(CC2=NNC(=O)c3c2cccc3C(F)(F)c4nc5cc(Cl)ccc5[nH]4)cc1C(=O)N6CCN(CC6)C(=O)C7CC7 | Olaparib 1756 |
| CC1=CC(=O)N(C1=O)C2(CC(=O)Oc3cccc4C(=NNC(=O)c34)Cc5ccc(F)c(c5)C(=O)N6CCN(CC6)C(=O)C7CC7)CCCC2 | Olaparib 1531 |
| C[C@@H]1CC[C@](C1)(Oc2cccc3C(=NNC(=O)c23)Cc4ccc(F)c(c4)C(=O)N5CCN(CC5)C(=O)C6CC6)c7ccc8OCCCOc8c7 | Olaparib 3150 |
| CC[C@H]1C[C@@](CCO1)(OC(=O)c2cccc3C(=NNC(=O)c23)Cc4ccc(F)c(c4)C(=O)N5CCN(CC5)C(=O)C6CC6)c7ccc(C)nc7 | Olaparib 1024 |
| Cc1ccc(CN2C[C@@H](CC2=O)Oc3cccc4C(=NNC(=O)c34)Cc5ccc(F)c(c5)C(=O)N6CCN(CC6)C(=O)C7CC7)cc1 | Olaparib 3099 |
| Cc1cc(Oc2cccc3C(=NNC(=O)c23)Cc4ccc(F)c(c4)C(=O)N5CCN(CC5)C(=O)C6CC6)cc7c(O)ccnc17 | Olaparib 2617 |
| CCC[C@@H](F)C(=O)Nc1cccc2C(=NNC(=O)c12)Cc3ccc(F)c(c3)C(=O)N4CCN(CC4)C(=O)C5CC5 | Olaparib 384 |
| Fc1ccc(CC2=NNC(=O)c3c(cccc23)C(=O)O[C@H]4CCc5c(OCc6ccccn6)cccc45)cc1C(=O)N7CCN(CC7)C(=O)C8CC8 | Olaparib 1137 |
| OS(=O)(=O)CCOc1cccc2C(=NNC(=O)c12)Cc3ccc(F)c(c3)C(=O)N4CCN(CC4)C(=O)C5CC5 | Olaparib 2777 |
| CCC[C@H](OC(=O)c1cccc2C(=NNC(=O)c12)Cc3ccc(F)c(c3)C(=O)N4CCN(CC4)C(=O)C5CC5)c6ccc7OC[C@H](C)COc7c6 | Olaparib 1000 |
| OC(=O)c1ccc(F)c(Oc2cccc3C(=NNC(=O)c23)Cc4ccc(F)c(c4)C(=O)N5CCN(CC5)C(=O)C6CC6)c1 | Olaparib 2264 |
| Fc1ccc2[nH]c(nc2c1)C(F)(F)c3cccc4C(=NNC(=O)c34)Cc5ccc(F)c(c5)C(=O)N6CCN(CC6)C(=O)C7CC7 | Olaparib 1766 |
| Fc1ccc(CC2=NNC(=O)c3c(NC(=O)C[C@H]4CCCN(C4)C(=O)NC5CC5)cccc23)cc1C(=O)N6CCN(CC6)C(=O)C7CC7 | Olaparib 529 |
| O[C@H](c1ccc(Oc2cccc3C(=NNC(=O)c23)Cc4ccc(F)c(c4)C(=O)N5CCN(CC5)C(=O)C6CC6)cc1)c7cccc8ccccc78 | Olaparib 2708 |
| Cc1cc(F)c([C@@H]2CCC(=O)N2)c(F)c1Oc3cccc4C(=NNC(=O)c34)Cc5ccc(F)c(c5)C(=O)N6CCN(CC6)C(=O)C7CC7 | Olaparib 2549 |
| CO[C@H]1CCC[C@@H]1NC(=O)c2ccc(N)c(c2)c3cccc4C(=NNC(=O)c34)Cc5ccc(F)c(c5)C(=O)N6CCN(CC6)C(=O)C7CC7 | Olaparib 1737 |
| Fc1ccc(CC2=NNC(=O)c3c(cccc23)C(=O)OCc4[nH]c5ccccc5c4Cl)cc1C(=O)N6CCN(CC6)C(=O)C7CC7 | Olaparib 1082 |
| CC(F)(F)CCC(=O)Nc1cccc2C(=NNC(=O)c12)Cc3ccc(F)c(c3)C(=O)N4CCN(CC4)C(=O)C5CC5 | Olaparib 329 |
| Fc1ccc(CC2=NNC(=O)c3c(cccc23)C(=O)O[C@H]4CN(C(=O)C4)c5ccc6ccccc6c5)cc1C(=O)N7CCN(CC7)C(=O)C8CC8 | Olaparib 1152 |
| Fc1ccc(CC2=NNC(=O)c3c(OC(F)(F)S(=O)(=O)CC#N)cccc23)cc1C(=O)N4CCN(CC4)C(=O)C5CC5 | Olaparib 2129 |
| OC(=O)c1cnc(F)c(c1)c2cccc3C(=NNC(=O)c23)Cc4ccc(F)c(c4)C(=O)N5CCN(CC5)C(=O)C6CC6 | Olaparib 1968 |
| Fc1ccc(CC2=NNC(=O)c3c2cccc3c4cccc5cn[nH]c45)cc1C(=O)N6CCN(CC6)C(=O)C7CC7 | Olaparib 1777 |
| Fc1ccc(CC2=NNC(=O)c3c(OC(=O)CC4c5ccccc5c6ccccc46)cccc23)cc1C(=O)N7CCN(CC7)C(=O)C8CC8 | Olaparib 1595 |
| CCCc1nccn1c2ccc(C(=O)C)c(Oc3cccc4C(=NNC(=O)c34)Cc5ccc(F)c(c5)C(=O)N6CCN(CC6)C(=O)C7CC7)c2 | Olaparib 2477 |
| CC(=O)N1CCC(CC1)c2cc(nc(C)n2)c3cccc4C(=NNC(=O)c34)Cc5ccc(F)c(c5)C(=O)N6CCN(CC6)C(=O)C7CC7 | Olaparib 1746 |
| Fc1ccc(CC2=NNC(=O)c3c(Oc4cccc(c4)[C@H]5CCC(=O)N5)cccc23)cc1C(=O)N6CCN(CC6)C(=O)C7CC7 | Olaparib 2602 |
| COc1cc(Oc2cccc3C(=NNC(=O)c23)Cc4ccc(F)c(c4)C(=O)N5CCN(CC5)C(=O)C6CC6)nc7ccccc17 | Olaparib 2605 |
| CC[C@@H](F)C(=O)Nc1cccc2C(=NNC(=O)c12)Cc3ccc(F)c(c3)C(=O)N4CCN(CC4)C(=O)C5CC5 | Olaparib 431 |
| Fc1ccc(CC2=NNC(=O)c3c(Oc4ccc(cc4)C(=O)N[C@H]5COc6ccccc56)cccc23)cc1C(=O)N7CCN(CC7)C(=O)C8CC8 | Olaparib 2700 |
| CN(C)C(=O)\C=C\C(=O)Nc1cccc2C(=NNC(=O)c12)Cc3ccc(F)c(c3)C(=O)N4CCN(CC4)C(=O)C5CC5 | Olaparib 314 |
| Fc1ccc(CC2=NNC(=O)c3c2cccc3c4c(C=O)c[nH]c4C=O)cc1C(=O)N5CCN(CC5)C(=O)C6CC6 | Olaparib 1721 |
| Fc1ccc(CC2=NNC(=O)c3c2cccc3C(F)(F)c4ccc(cc4)[C@@H]5CCC(=O)N5)cc1C(=O)N6CCN(CC6)C(=O)C7CC7 | Olaparib 1753 |
| CN1C(=O)N(C)c2nc(Oc3cccc4C(=NNC(=O)c34)Cc5ccc(F)c(c5)C(=O)N6CCN(CC6)C(=O)C7CC7)[nH]c2C1=O | Olaparib 2572 |
| CCn1nccc1c2cc(CNC(=O)c3cccc4C(=NNC(=O)c34)Cc5ccc(F)c(c5)C(=O)N6CCN(CC6)C(=O)C7CC7)ccc2F | Olaparib 227 |
| Cc1cc(c(O)c(Oc2cccc3C(=NNC(=O)c23)Cc4ccc(F)c(c4)C(=O)N5CCN(CC5)C(=O)C6CC6)c1F)C7(CCC7)C(=O)O | Olaparib 2527 |
| Fc1ccc(CC2=NNC(=O)c3c(Oc4cc([C@H]5CCC(=O)N5)c(Cl)cc4Cl)cccc23)cc1C(=O)N6CCN(CC6)C(=O)C7CC7 | Olaparib 2517 |
| Nc1ccnc2ccc(Oc3cccc4C(=NNC(=O)c34)Cc5ccc(F)c(c5)C(=O)N6CCN(CC6)C(=O)C7CC7)cc12 | Olaparib 2616 |
| C[C@@](F)(Cl)C(=O)Nc1cccc2C(=NNC(=O)c12)Cc3ccc(F)c(c3)C(=O)N4CCN(CC4)C(=O)C5CC5 | Olaparib 373 |
| Fc1ccc(CC2=NNC(=O)c3c(cccc23)C(=O)OCc4cc5CN(Cc6ccccc6)CCc5[nH]4)cc1C(=O)N7CCN(CC7)C(=O)C8CC8 | Olaparib 1135 |
| CC[C@@H](C)[C@H](NC(=O)c1cccc2C(=NNC(=O)c12)Cc3ccc(F)c(c3)C(=O)N4CCN(CC4)C(=O)C5CC5)C(=O)O | Olaparib 30 |
| Cc1ccc(CNC(=O)c2cccc3C(=NNC(=O)c23)Cc4ccc(F)c(c4)C(=O)N5CCN(CC5)C(=O)C6CC6)n1C | Olaparib 197 |
| CN(Cc1cccc(c1)c2cccc3C(=NNC(=O)c23)Cc4ccc(F)c(c4)C(=O)N5CCN(CC5)C(=O)C6CC6)[C@H]7CCSC7 | Olaparib 2051 |
| NC1=N[C@@H]2O[C@H](CO)[C@@H](OC(=O)c3cccc4C(=NNC(=O)c34)Cc5ccc(F)c(c5)C(=O)N6CCN(CC6)C(=O)C7CC7)[C@@H]2O1 | Olaparib 1097 |
| NCc1ncccc1COc2ccc(Oc3cccc4C(=NNC(=O)c34)Cc5ccc(F)c(c5)C(=O)N6CCN(CC6)C(=O)C7CC7)c(Cl)c2 | Olaparib 2469 |
| C[C@H]1COc2ccc(cc2OC1)C#CCNC(=O)c3cccc4C(=NNC(=O)c34)Cc5ccc(F)c(c5)C(=O)N6CCN(CC6)C(=O)C7CC7 | Olaparib 228 |
| Fc1ccc(CC2=NNC(=O)c3c2cccc3c4ccnc(c4)C(=O)NC5CC5)cc1C(=O)N6CCN(CC6)C(=O)C7CC7 | Olaparib 2063 |
| Fc1ccc(CC2=NNC(=O)c3c(cccc23)C(=O)Oc4ccc5cc[nH]c5c4)cc1C(=O)N6CCN(CC6)C(=O)C7CC7 | Olaparib 1120 |
| CN(C)C\C=C\c1cccc2C(=NNC(=O)c12)Cc3ccc(F)c(c3)C(=O)N4CCN(CC4)C(=O)C5CC5 | Olaparib 1934 |
| Nc1ccc2N(Cc3ccnc(Oc4cccc5C(=NNC(=O)c45)Cc6ccc(F)c(c6)C(=O)N7CCN(CC7)C(=O)C8CC8)c3)CCCc2c1 | Olaparib 2642 |
| CC(C)(C)OC(=O)CNC(=O)c1cccc2C(=NNC(=O)c12)Cc3ccc(F)c(c3)C(=O)N4CCN(CC4)C(=O)C5CC5 | Olaparib 27 |
| CC(C)(C#N)c1cc(Oc2cccc3C(=NNC(=O)c23)Cc4ccc(F)c(c4)C(=O)N5CCN(CC5)C(=O)C6CC6)c(F)c(F)c1O | Olaparib 2172 |
| Fc1ccc(CC2=NNC(=O)c3c2cccc3c4cncc5ccccc45)cc1C(=O)N6CCN(CC6)C(=O)C7CC7 | Olaparib 1888 |
| Fc1ccc(CC2=NNC(=O)c3c(cccc23)C(=O)NC(=S)c4cccc(c4)c5ncn[nH]5)cc1C(=O)N6CCN(CC6)C(=O)C7CC7 | Olaparib 237 |
| Fc1ccc(CC2=NNC(=O)c3c(Oc4ccc(NCc5ccnc(c5)C#N)c(Cl)c4)cccc23)cc1C(=O)N6CCN(CC6)C(=O)C7CC7 | Olaparib 2434 |
| C[C@@H](CNC(=O)[C@H]1COc2ccccc12)Oc3cccc4C(=NNC(=O)c34)Cc5ccc(F)c(c5)C(=O)N6CCN(CC6)C(=O)C7CC7 | Olaparib 3080 |
| NCc1cc(Oc2cccc3C(=NNC(=O)c23)Cc4ccc(F)c(c4)C(=O)N5CCN(CC5)C(=O)C6CC6)ccc1CN7CCCCCCC7 | Olaparib 2508 |
| Fc1ccc(CC2=NNC(=O)c3c(cccc23)C(=O)Oc4cccc5ccoc45)cc1C(=O)N6CCN(CC6)C(=O)C7CC7 | Olaparib 1119 |
| COC[C@@H](C)c1cccc2C(=NNC(=O)c12)Cc3ccc(F)c(c3)C(=O)N4CCN(CC4)C(=O)C5CC5 | Olaparib 1939 |
| Cc1cc(ccc1Oc2cccc3C(=NNC(=O)c23)Cc4ccc(F)c(c4)C(=O)N5CCN(CC5)C(=O)C6CC6)[C@@H](O)c7ccc8OCOc8c7 | Olaparib 2684 |
| CC(C)C(=O)NCC(=O)Oc1cccc2C(=NNC(=O)c12)Cc3ccc(F)c(c3)C(=O)N4CCN(CC4)C(=O)C5CC5 | Olaparib 1232 |
| CCC[C@@H](C)Cn1cc(c(N)n1)c2cccc3C(=NNC(=O)c23)Cc4ccc(F)c(c4)C(=O)N5CCN(CC5)C(=O)C6CC6 | Olaparib 1685 |
| Fc1ccc(CC2=NNC(=O)c3c2cccc3c4cccc(n4)C5CCCC5)cc1C(=O)N6CCN(CC6)C(=O)C7CC7 | Olaparib 2071 |
| Cc1cc(NC(=O)c2ccc(N)c(Oc3cccc4C(=NNC(=O)c34)Cc5ccc(F)c(c5)C(=O)N6CCN(CC6)C(=O)C7CC7)c2)[nH]n1 | Olaparib 2507 |
| Fc1ccccc1COc2ccnc(Oc3cccc4C(=NNC(=O)c34)Cc5ccc(F)c(c5)C(=O)N6CCN(CC6)C(=O)C7CC7)n2 | Olaparib 2533 |
| C[C@H]1CC[C@H](CNC(=O)c2cccc3C(=NNC(=O)c23)Cc4ccc(F)c(c4)C(=O)N5CCN(CC5)C(=O)C6CC6)CN1Cc7occc7 | Olaparib 234 |
| CCN1CCC[C@H](CN)[C@H]1c2ccc(Oc3cccc4C(=NNC(=O)c34)Cc5ccc(F)c(c5)C(=O)N6CCN(CC6)C(=O)C7CC7)cc2 | Olaparib 2538 |
| Fc1ccc(CC2=NNC(=O)c3c(Oc4ccc5nccnc5c4)cccc23)cc1C(=O)N6CCN(CC6)C(=O)C7CC7 | Olaparib 2589 |
| Fc1ccc(CC2=NNC(=O)c3c2cccc3[C@@](F)(I)C(F)(F)F)cc1C(=O)N4CCN(CC4)C(=O)C5CC5 | Olaparib 1603 |
| CC1=NCCc2c1[nH]c3cc(OC(=O)c4cccc5C(=NNC(=O)c45)Cc6ccc(F)c(c6)C(=O)N7CCN(CC7)C(=O)C8CC8)ccc23 | Olaparib 1167 |
| C[C@@](NC(=O)c1cccc2C(=NNC(=O)c12)Cc3ccc(F)c(c3)C(=O)N4CCN(CC4)C(=O)C5CC5)(C(=O)O)C(F)(F)F | Olaparib 2 |
| OC1=CC(=O)NC(=C1)C(F)(F)c2cccc3C(=NNC(=O)c23)Cc4ccc(F)c(c4)C(=O)N5CCN(CC5)C(=O)C6CC6 | Olaparib 1706 |
| Fc1ccc(CC2=NNC(=O)c3c(cccc23)[C@@H]4C[C@@H]4c5ccccc5)cc1C(=O)N6CCN(CC6)C(=O)C7CC7 | Olaparib 2080 |
| Fc1ccc(CC2=NNC(=O)c3c(Oc4ccc(\C(=C\n5cncn5)\Cl)c(Cl)c4)cccc23)cc1C(=O)N6CCN(CC6)C(=O)C7CC7 | Olaparib 2444 |
| Cc1nonc1CSc2ccccc2OC(=O)c3cccc4C(=NNC(=O)c34)Cc5ccc(F)c(c5)C(=O)N6CCN(CC6)C(=O)C7CC7 | Olaparib 1023 |
| Fc1ccc(CC2=NNC(=O)c3c2cccc3c4nc(Cl)nc(NC5CCCCC5)n4)cc1C(=O)N6CCN(CC6)C(=O)C7CC7 | Olaparib 1748 |
| C[C@H]1CCN(Cc2cccc(Oc3cccc4C(=NNC(=O)c34)Cc5ccc(F)c(c5)C(=O)N6CCN(CC6)C(=O)C7CC7)c2)C[C@@H]1N | Olaparib 2561 |
| Fc1ccc(CC2=NNC(=O)c3c(Oc4ccc5[nH]c(nc5c4)C(F)(F)F)cccc23)cc1C(=O)N6CCN(CC6)C(=O)C7CC7 | Olaparib 2567 |
| C[C@H](N)c1cc(Oc2cccc3C(=NNC(=O)c23)Cc4ccc(F)c(c4)C(=O)N5CCN(CC5)C(=O)C6CC6)ccc1n7ccc(n7)C8CC8 | Olaparib 2718 |
| O[C@H](NC(=O)C=C)C(Cl)(Cl)Oc1cccc2C(=NNC(=O)c12)Cc3ccc(F)c(c3)C(=O)N4CCN(CC4)C(=O)C5CC5 | Olaparib 2103 |
| C\C(=C/C(=O)Oc1cccc2C(=NNC(=O)c12)Cc3ccc(F)c(c3)C(=O)N4CCN(CC4)C(=O)C5CC5)\CN6CCCC(C)(C)C6 | Olaparib 1438 |
| Fc1ccc(CC2=NNC(=O)c3c(CC[C@H]4C[C@H]5C[C@@H]4C=C5)cccc23)cc1C(=O)N6CCN(CC6)C(=O)C7CC7 | Olaparib 1844 |
| Fc1ccc(CC2=NNC(=O)c3c(OC(=O)c4cncc(c4)c5cocc5)cccc23)cc1C(=O)N6CCN(CC6)C(=O)C7CC7 | Olaparib 1567 |
| Fc1ccc(CC2=NNC(=O)c3c(cccc23)C(=O)OCc4cncc(c4)c5nncs5)cc1C(=O)N6CCN(CC6)C(=O)C7CC7 | Olaparib 1063 |
| NC[C@]1(COc2cccc3C(=NNC(=O)c23)Cc4ccc(F)c(c4)C(=O)N5CCN(CC5)C(=O)C6CC6)C[C@H]1c7ccncn7 | Olaparib 3119 |
| CC[C@H]1C[C@H](CCO1)Nc2ccc(Oc3cccc4C(=NNC(=O)c34)Cc5ccc(F)c(c5)C(=O)N6CCN(CC6)C(=O)C7CC7)cc2 | Olaparib 3079 |
| Fc1ccc(CC2=NNC(=O)c3c(cccc23)C(=O)Oc4ccc(cc4)C5=CCNCC5)cc1C(=O)N6CCN(CC6)C(=O)C7CC7 | Olaparib 1092 |
| Fc1ccc(CC2=NNC(=O)c3c(Oc4ccc5c(NCCN6CCCC6)ccnc5c4)cccc23)cc1C(=O)N7CCN(CC7)C(=O)C8CC8 | Olaparib 2637 |
| COC(=O)C[C@@H](O)C(Cl)(Cl)Oc1cccc2C(=NNC(=O)c12)Cc3ccc(F)c(c3)C(=O)N4CCN(CC4)C(=O)C5CC5 | Olaparib 2102 |
| CC[C@H]1C\C(=C/CCNC(=O)c2cccc3C(=NNC(=O)c23)Cc4ccc(F)c(c4)C(=O)N5CCN(CC5)C(=O)C6CC6)\CCO1 | Olaparib 163 |
| CC(C)(C)OC(=O)Nc1sccc1c2cccc3C(=NNC(=O)c23)Cc4ccc(F)c(c4)C(=O)N5CCN(CC5)C(=O)C6CC6 | Olaparib 1953 |
| C[C@@H](CN1C[C@@H](N)Cc2ccccc12)Oc3cccc4C(=NNC(=O)c34)Cc5ccc(F)c(c5)C(=O)N6CCN(CC6)C(=O)C7CC7 | Olaparib 3091 |
| OC(=O)C1=NN(C=CC1=O)c2cc(Oc3cccc4C(=NNC(=O)c34)Cc5ccc(F)c(c5)C(=O)N6CCN(CC6)C(=O)C7CC7)ccc2F | Olaparib 2460 |
| C[C@@H](Oc1cccc2C(=NNC(=O)c12)Cc3ccc(F)c(c3)C(=O)N4CCN(CC4)C(=O)C5CC5)[C@H](C)c6onc(n6)c7ccccc7C | Olaparib 3071 |
| CC1(C)OCc2c(Oc3cccc4C(=NNC(=O)c34)Cc5ccc(F)c(c5)C(=O)N6CCN(CC6)C(=O)C7CC7)nc(Cl)nc12 | Olaparib 2569 |
| Fc1ccc(CC2=NNC(=O)c3c(OC4=Cc5cc(Br)ccc5NC4=O)cccc23)cc1C(=O)N6CCN(CC6)C(=O)C7CC7 | Olaparib 3061 |
| NCc1cc(nc2ccccc12)N3CC[C@H](C3)Oc4cccc5C(=NNC(=O)c45)Cc6ccc(F)c(c6)C(=O)N7CCN(CC7)C(=O)C8CC8 | Olaparib 3160 |
| N[C@@H](COc1cccc2C(=NNC(=O)c12)Cc3ccc(F)c(c3)C(=O)N4CCN(CC4)C(=O)C5CC5)C(=O)N | Olaparib 2841 |
| O[C@H]1OC(=O)C(=C1Cl)c2cccc3C(=NNC(=O)c23)Cc4ccc(F)c(c4)C(=O)N5CCN(CC5)C(=O)C6CC6 | Olaparib 1713 |
| N[C@@H](C(=O)Nc1cccc2C(=NNC(=O)c12)Cc3ccc(F)c(c3)C(=O)N4CCN(CC4)C(=O)C5CC5)c6cccs6 | Olaparib 508 |
| C[C@H]1CCC[C@@](CC(=O)Oc2cccc3C(=NNC(=O)c23)Cc4ccc(F)c(c4)C(=O)N5CCN(CC5)C(=O)C6CC6)(C1)N7CCCCC7 | Olaparib 1528 |
| Cc1cn(C[C@](C)(O)C(=O)Oc2cccc3C(=NNC(=O)c23)Cc4ccc(F)c(c4)C(=O)N5CCN(CC5)C(=O)C6CC6)c7ccccc17 | Olaparib 1537 |
| CC[C@H]1CCCN1S(=O)(=O)c2cc(N)cc(Oc3cccc4C(=NNC(=O)c34)Cc5ccc(F)c(c5)C(=O)N6CCN(CC6)C(=O)C7CC7)c2 | Olaparib 2451 |
| CN1CC[C@@H](N)[C@@H]1c2ccc(Cl)c(Oc3cccc4C(=NNC(=O)c34)Cc5ccc(F)c(c5)C(=O)N6CCN(CC6)C(=O)C7CC7)c2 | Olaparib 2523 |
| CC(C)(NC(=O)c1cccc2C(=NNC(=O)c12)Cc3ccc(F)c(c3)C(=O)N4CCN(CC4)C(=O)C5CC5)c6ccc(N7CCCCC7)c(F)c6 | Olaparib 212 |
| O[C@@H]1OC(=O)C=C1c2cccc3C(=NNC(=O)c23)Cc4ccc(F)c(c4)C(=O)N5CCN(CC5)C(=O)C6CC6 | Olaparib 1730 |
| Fc1ccc(CC2=NNC(=O)c3c(O[C@@H]4CCN(C4)c5cc(C#N)c6ccccc6n5)cccc23)cc1C(=O)N7CCN(CC7)C(=O)C8CC8 | Olaparib 3170 |
| C[C@H]1CC[C@H](CNC(=O)c2cccc3C(=NNC(=O)c23)Cc4ccc(F)c(c4)C(=O)N5CCN(CC5)C(=O)C6CC6)CN1S(=O)(=O)N(C)C | Olaparib 130 |
| N[C@H](C(=O)Nc1cccc2C(=NNC(=O)c12)Cc3ccc(F)c(c3)C(=O)N4CCN(CC4)C(=O)C5CC5)c6sccc6Cl | Olaparib 487 |
| Cc1cccc2c(C[C@@H](N)C(=O)Oc3cccc4C(=NNC(=O)c34)Cc5ccc(F)c(c5)C(=O)N6CCN(CC6)C(=O)C7CC7)c[nH]c12 | Olaparib 1550 |
| CC1(C)N=C(N)N=C(N)N1c2ccc(Oc3cccc4C(=NNC(=O)c34)Cc5ccc(F)c(c5)C(=O)N6CCN(CC6)C(=O)C7CC7)cc2 | Olaparib 2502 |
| NC1(CCC1)c2ccc(Oc3cccc4C(=NNC(=O)c34)Cc5ccc(F)c(c5)C(=O)N6CCN(CC6)C(=O)C7CC7)cc2CO | Olaparib 2603 |
| Cc1cc(C)cc(c1)[C@@H]2C[C@@H](CN2)OC(=O)c3cccc4C(=NNC(=O)c34)Cc5ccc(F)c(c5)C(=O)N6CCN(CC6)C(=O)C7CC7 | Olaparib 1067 |
| Fc1ccc(CC2=NNC(=O)c3c2cccc3C4=C(Cl)OC(=O)O4)cc1C(=O)N5CCN(CC5)C(=O)C6CC6 | Olaparib 1723 |
| Cc1nnc(c2cccc3C(=NNC(=O)c23)Cc4ccc(F)c(c4)C(=O)N5CCN(CC5)C(=O)C6CC6)n1[C@H]7COc8ccccc78 | Olaparib 1789 |
| C[C@@]1(CCCN(C1)S(=O)(=O)C(F)(F)Oc2cccc3C(=NNC(=O)c23)Cc4ccc(F)c(c4)C(=O)N5CCN(CC5)C(=O)C6CC6)C(=O)O | Olaparib 2173 |
| N[C@H](C(=O)Oc1cccc2C(=NNC(=O)c12)Cc3ccc(F)c(c3)C(=O)N4CCN(CC4)C(=O)C5CC5)c6sccc6Cl | Olaparib 1463 |
| C[C@@](N)(Cc1c[nH]c2ccccc12)C(=O)Oc3cccc4C(=NNC(=O)c34)Cc5ccc(F)c(c5)C(=O)N6CCN(CC6)C(=O)C7CC7 | Olaparib 1551 |
| CC[C@H]1N(CCNC1=O)C(=O)c2cccc(Oc3cccc4C(=NNC(=O)c34)Cc5ccc(F)c(c5)C(=O)N6CCN(CC6)C(=O)C7CC7)n2 | Olaparib 2466 |
| Fc1ccc(CC2=NNC(=O)c3c(Oc4cccc(c4)[C@H]5CNC[C@@H]5C#N)cccc23)cc1C(=O)N6CCN(CC6)C(=O)C7CC7 | Olaparib 2611 |
| Fc1ccc(CC2=NNC(=O)c3c(cccc23)C(=O)OCC4CCN(Cc5ccccc5)CC4)cc1C(=O)N6CCN(CC6)C(=O)C7CC7 | Olaparib 1050 |
| C[C@@H](NC(=O)c1cccc2C(=NNC(=O)c12)Cc3ccc(F)c(c3)C(=O)N4CCN(CC4)C(=O)C5CC5)[C@H]6CCCN(CC(C)(C)C)C6 | Olaparib 150 |
| Fc1ccc(CC2=NNC(=O)c3c(Oc4ccc5[nH]c6C(=O)NCCc6c5c4)cccc23)cc1C(=O)N7CCN(CC7)C(=O)C8CC8 | Olaparib 2664 |
| C[C@@H](N(C)C(=O)c1ccnn1C)C(=O)Oc2cccc3C(=NNC(=O)c23)Cc4ccc(F)c(c4)C(=O)N5CCN(CC5)C(=O)C6CC6 | Olaparib 1441 |
| C[C@@H](Cc1nncs1)Oc2cccc3C(=NNC(=O)c23)Cc4ccc(F)c(c4)C(=O)N5CCN(CC5)C(=O)C6CC6 | Olaparib 2989 |
| NCc1oc(COc2ccc(Oc3cccc4C(=NNC(=O)c34)Cc5ccc(F)c(c5)C(=O)N6CCN(CC6)C(=O)C7CC7)c(Cl)c2)cc1 | Olaparib 2492 |
| CC1=CN([C@H]2CC[C@@H](COc3cccc4C(=NNC(=O)c34)Cc5ccc(F)c(c5)C(=O)N6CCN(CC6)C(=O)C7CC7)O2)C(=O)NC1=O | Olaparib 3073 |
| Oc1c(Oc2cccc3C(=NNC(=O)c23)Cc4ccc(F)c(c4)C(=O)N5CCN(CC5)C(=O)C6CC6)ccc(F)c1C7(CC7)C#N | Olaparib 2490 |
| CCCc1ccccc1N2C[C@@H](CC2=O)Oc3cccc4C(=NNC(=O)c34)Cc5ccc(F)c(c5)C(=O)N6CCN(CC6)C(=O)C7CC7 | Olaparib 3084 |
| C[C@H]1CC[C@H](CNC(=O)c2cccc3C(=NNC(=O)c23)Cc4ccc(F)c(c4)C(=O)N5CCN(CC5)C(=O)C6CC6)CN1CCC#C | Olaparib 160 |
| Fc1ccc(CC2=NNC(=O)c3c(Oc4ccc5c(ccn5CCN6CCOCC6)c4)cccc23)cc1C(=O)N7CCN(CC7)C(=O)C8CC8 | Olaparib 2710 |
| CS(=O)(=O)CCn1cc(c(N)n1)c2cccc3C(=NNC(=O)c23)Cc4ccc(F)c(c4)C(=O)N5CCN(CC5)C(=O)C6CC6 | Olaparib 1668 |
| Nc1nnc(s1)C(F)(F)c2cccc3C(=NNC(=O)c23)Cc4ccc(F)c(c4)C(=O)N5CCN(CC5)C(=O)C6CC6 | Olaparib 1711 |
| NCc1oc(COc2ccc(F)c(Oc3cccc4C(=NNC(=O)c34)Cc5ccc(F)c(c5)C(=O)N6CCN(CC6)C(=O)C7CC7)c2)cc1 | Olaparib 2491 |
| NC1=NC(=O)N(C=C1)[C@H]2CC[C@@H](COc3cccc4C(=NNC(=O)c34)Cc5ccc(F)c(c5)C(=O)N6CCN(CC6)C(=O)C7CC7)O2 | Olaparib 3089 |
| CC[C@H]1OCc2ncnc(c3cccc4C(=NNC(=O)c34)Cc5ccc(F)c(c5)C(=O)N6CCN(CC6)C(=O)C7CC7)c12 | Olaparib 1772 |
| COc1ccccc1[C@H]2C[C@@H]2COC(=O)c3cccc4C(=NNC(=O)c34)Cc5ccc(F)c(c5)C(=O)N6CCN(CC6)C(=O)C7CC7 | Olaparib 1087 |
| CC[C@@H]1CCN(CCOc2cccc3C(=NNC(=O)c23)Cc4ccc(F)c(c4)C(=O)N5CCN(CC5)C(=O)C6CC6)C(=O)CC1 | Olaparib 2888 |
| Fc1ccc(CC2=NNC(=O)c3c(cccc23)C(=O)Oc4ccc(NC(=O)[C@H]5C[C@H]6CC[C@@H]5C6)cc4F)cc1C(=O)N7CCN(CC7)C(=O)C8CC8 | Olaparib 1122 |
| Cc1nn(C)c(c2cccc3C(=NNC(=O)c23)Cc4ccc(F)c(c4)C(=O)N5CCN(CC5)C(=O)C6CC6)c1S(=O)(=O)N | Olaparib 1676 |
| Oc1nc(Oc2cccc3C(=NNC(=O)c23)Cc4ccc(F)c(c4)C(=O)N5CCN(CC5)C(=O)C6CC6)c(Br)s1 | Olaparib 2876 |
| Nc1nn(CCc2ccccc2)cc1Oc3cccc4C(=NNC(=O)c34)Cc5ccc(F)c(c5)C(=O)N6CCN(CC6)C(=O)C7CC7 | Olaparib 2562 |
| Fc1ccc(CC2=NNC(=O)c3c(OC(=O)CC4CN(C4)C(=O)N5CCCNCC5)cccc23)cc1C(=O)N6CCN(CC6)C(=O)C7CC7 | Olaparib 1527 |
| N[C@H]1COc2c(Oc3cccc4C(=NNC(=O)c34)Cc5ccc(F)c(c5)C(=O)N6CCN(CC6)C(=O)C7CC7)ccc(Br)c12 | Olaparib 2514 |
| CCc1cccc(c1OC(=O)c2cccc3C(=NNC(=O)c23)Cc4ccc(F)c(c4)C(=O)N5CCN(CC5)C(=O)C6CC6)C7(CC7)C#N | Olaparib 1077 |
| CN(CCNC(=O)c1cccc2C(=NNC(=O)c12)Cc3ccc(F)c(c3)C(=O)N4CCN(CC4)C(=O)C5CC5)C6CCC6 | Olaparib 195 |
| Fc1ccc(CC2=NNC(=O)c3c(Oc4cccc(NC(=O)[C@H]5CC56CCNCC6)c4)cccc23)cc1C(=O)N7CCN(CC7)C(=O)C8CC8 | Olaparib 2711 |
| C[C@@H](Cn1cc(Oc2cccc3C(=NNC(=O)c23)Cc4ccc(F)c(c4)C(=O)N5CCN(CC5)C(=O)C6CC6)c(N)n1)C(=O)N | Olaparib 2306 |
| Fc1ccc(CC2=NNC(=O)c3c2cccc3C4=NSN=C(Cl)C4=O)cc1C(=O)N5CCN(CC5)C(=O)C6CC6 | Olaparib 1702 |
| COc1ccc2[nH]c(CC(=O)Nc3cccc4C(=NNC(=O)c34)Cc5ccc(F)c(c5)C(=O)N6CCN(CC6)C(=O)C7CC7)cc2c1 | Olaparib 538 |
| Cc1nn(Cc2cccc(c2)C(=N)NC(=O)c3cccc4C(=NNC(=O)c34)Cc5ccc(F)c(c5)C(=O)N6CCN(CC6)C(=O)C7CC7)cc1Cl | Olaparib 200 |
| C[C@H]1OCc2ncnc(c3cccc4C(=NNC(=O)c34)Cc5ccc(F)c(c5)C(=O)N6CCN(CC6)C(=O)C7CC7)c12 | Olaparib 1775 |
| NC1(CCC1)c2c(F)c(F)c(OC(=O)c3cccc4C(=NNC(=O)c34)Cc5ccc(F)c(c5)C(=O)N6CCN(CC6)C(=O)C7CC7)c(F)c2F | Olaparib 1001 |
| Fc1ccc(CC2=NNC(=O)c3c(NC(=O)CN4CCC4)cccc23)cc1C(=O)N5CCN(CC5)C(=O)C6CC6 | Olaparib 518 |
| Fc1ccc(CC2=NNC(=O)c3c(cccc23)C(=O)OC4C[C@H]5CC[C@@H](C4)N5C(=O)C6CCCCC6)cc1C(=O)N7CCN(CC7)C(=O)C8CC8 | Olaparib 1145 |
| CN(c1nncs1)C(C)(C)C(=O)Nc2cccc3C(=NNC(=O)c23)Cc4ccc(F)c(c4)C(=O)N5CCN(CC5)C(=O)C6CC6 | Olaparib 481 |
| C[C@H](COc1cccc2C(=NNC(=O)c12)Cc3ccc(F)c(c3)C(=O)N4CCN(CC4)C(=O)C5CC5)NC(=O)[C@H]6CCSC6 | Olaparib 2883 |
| Cc1nccn1c2nccc(n2)c3cccc4C(=NNC(=O)c34)Cc5ccc(F)c(c5)C(=O)N6CCN(CC6)C(=O)C7CC7 | Olaparib 2065 |
| Cc1cc(Oc2cccc3C(=NNC(=O)c23)Cc4ccc(F)c(c4)C(=O)N5CCN(CC5)C(=O)C6CC6)ccc1NC(=O)c7c(C)n[nH]c7C | Olaparib 3057 |
| C[C@H]1CC[C@@H]([C@@H](C)C1)n2cnnc2c3cccc4C(=NNC(=O)c34)Cc5ccc(F)c(c5)C(=O)N6CCN(CC6)C(=O)C7CC7 | Olaparib 1761 |
| Cc1cc(Oc2cccc3C(=NNC(=O)c23)Cc4ccc(F)c(c4)C(=O)N5CCN(CC5)C(=O)C6CC6)ccc1[C@@H]7CCC(=O)N7 | Olaparib 3110 |
| CC[C@@H](CN1CC[C@H](C)[C@@H](N)C1)Oc2cccc3C(=NNC(=O)c23)Cc4ccc(F)c(c4)C(=O)N5CCN(CC5)C(=O)C6CC6 | Olaparib 2885 |
| Fc1ccc(CC2=NNC(=O)c3c(Oc4ccc5oc(nc5c4)c6ccccc6)cccc23)cc1C(=O)N7CCN(CC7)C(=O)C8CC8 | Olaparib 2735 |
| OCc1onc(CCNC(=O)c2cccc3C(=NNC(=O)c23)Cc4ccc(F)c(c4)C(=O)N5CCN(CC5)C(=O)C6CC6)n1 | Olaparib 192 |
| Fc1ccc(CC2=NNC(=O)c3c(OC(=O)CN[C@@H]4CCSC4)cccc23)cc1C(=O)N5CCN(CC5)C(=O)C6CC6 | Olaparib 1492 |
| Fc1ccc(CC2=NNC(=O)c3c(Oc4cc5[nH]nnc5cc4Cl)cccc23)cc1C(=O)N6CCN(CC6)C(=O)C7CC7 | Olaparib 2612 |
| Cc1ccc(cc1)c2nc(N)nc(Oc3cccc4C(=NNC(=O)c34)Cc5ccc(F)c(c5)C(=O)N6CCN(CC6)C(=O)C7CC7)n2 | Olaparib 2566 |
| Fc1ccc(CC2=NNC(=O)c3c2cccc3c4csc(NC5CCCCC5)n4)cc1C(=O)N6CCN(CC6)C(=O)C7CC7 | Olaparib 2057 |
| Fc1ccc(CC2=NNC(=O)c3c(O[C@@H]4CN(C(=O)C4)c5ccc(Cl)cc5Cl)cccc23)cc1C(=O)N6CCN(CC6)C(=O)C7CC7 | Olaparib 3056 |
| CC1(C)CC(CC(C)(C)N1[O])Oc2cccc3C(=NNC(=O)c23)Cc4ccc(F)c(c4)C(=O)N5CCN(CC5)C(=O)C6CC6 | Olaparib 2914 |
| Fc1ccc(CC2=NNC(=O)c3c(Oc4ccc5CC\C(=C/C(=O)Nc6ccccc6)\c5c4)cccc23)cc1C(=O)N7CCN(CC7)C(=O)C8CC8 | Olaparib 2656 |
| CCOC(=O)CN1N=CC(=C(Cl)C1=O)Oc2cccc3C(=NNC(=O)c23)Cc4ccc(F)c(c4)C(=O)N5CCN(CC5)C(=O)C6CC6 | Olaparib 2203 |
| Fc1ccc(CC2=NNC(=O)c3c(cccc23)C(=O)N\C(=C/C#N)\c4ccccc4)cc1C(=O)N5CCN(CC5)C(=O)C6CC6 | Olaparib 189 |
| CC(C)(NC(=O)c1cccc2C(=NNC(=O)c12)Cc3ccc(F)c(c3)C(=O)N4CCN(CC4)C(=O)C5CC5)c6ccc7OC(=O)C=Cc7c6 | Olaparib 238 |
| Fc1ccc(CC2=NNC(=O)c3c(cccc23)C(=O)OCc4cncc(c4)c5cncs5)cc1C(=O)N6CCN(CC6)C(=O)C7CC7 | Olaparib 1064 |
| CC1(C)[C@]2(CC(=O)Nc3cccc4C(=NNC(=O)c34)Cc5ccc(F)c(c5)C(=O)N6CCN(CC6)C(=O)C7CC7)CC[C@@]1(C)C(=O)C2 | Olaparib 536 |
| OC(=O)C1(CC1)c2c(F)cc(Oc3cccc4C(=NNC(=O)c34)Cc5ccc(F)c(c5)C(=O)N6CCN(CC6)C(=O)C7CC7)cc2F | Olaparib 2545 |
| C[C@@H]1CNC[C@H](C)N1C[C@@H](Oc2cccc3C(=NNC(=O)c23)Cc4ccc(F)c(c4)C(=O)N5CCN(CC5)C(=O)C6CC6)C(F)(F)F | Olaparib 2860 |
| OC(=O)C1(Cc2ccccc2C1)c3cccc(Oc4cccc5C(=NNC(=O)c45)Cc6ccc(F)c(c6)C(=O)N7CCN(CC7)C(=O)C8CC8)c3 | Olaparib 2703 |
| C[C@H]1CC[C@H](CNC(=O)c2cccc3C(=NNC(=O)c23)Cc4ccc(F)c(c4)C(=O)N5CCN(CC5)C(=O)C6CC6)CN1S(=O)(=O)C(C)(C)C | Olaparib 125 |
| Fc1ccc(CC2=NNC(=O)c3c(Oc4cc(C=O)c(Cl)cc4C=O)cccc23)cc1C(=O)N5CCN(CC5)C(=O)C6CC6 | Olaparib 2305 |
| Cc1ccc(cc1)N2C(=O)C=C(Oc3cccc4C(=NNC(=O)c34)Cc5ccc(F)c(c5)C(=O)N6CCN(CC6)C(=O)C7CC7)C2=O | Olaparib 2564 |
| OC(=O)c1cnc2cc(Oc3cccc4C(=NNC(=O)c34)Cc5ccc(F)c(c5)C(=O)N6CCN(CC6)C(=O)C7CC7)ccc2c1O | Olaparib 2557 |
| CC1(C)[C@]2(CC(=O)Oc3cccc4C(=NNC(=O)c34)Cc5ccc(F)c(c5)C(=O)N6CCN(CC6)C(=O)C7CC7)CC[C@@]1(C)C(=O)C2 | Olaparib 1558 |
| Fc1ccc(CC2=NNC(=O)c3c(cccc23)C(=O)Oc4ccc5COCc5c4)cc1C(=O)N6CCN(CC6)C(=O)C7CC7 | Olaparib 1118 |
| C[C@H]1CN(C[C@H](Oc2cccc3C(=NNC(=O)c23)Cc4ccc(F)c(c4)C(=O)N5CCN(CC5)C(=O)C6CC6)C(F)(F)F)[C@H](C)CO1 | Olaparib 2859 |
| Fc1ccc(CC2=NNC(=O)c3c(Oc4cc5CCc6ccccc6c5nn4)cccc23)cc1C(=O)N7CCN(CC7)C(=O)C8CC8 | Olaparib 2739 |
| C[C@H]1CC[C@H](CNC(=O)c2cccc3C(=NNC(=O)c23)Cc4ccc(F)c(c4)C(=O)N5CCN(CC5)C(=O)C6CC6)CN1S(=O)(=O)C(F)F | Olaparib 129 |
| CC(C)(C(=O)O)c1cccc(c1O)C(F)(F)c2cccc3C(=NNC(=O)c23)Cc4ccc(F)c(c4)C(=O)N5CCN(CC5)C(=O)C6CC6 | Olaparib 1651 |
| Cc1ccc(C)c(c1)C2=NO[C@@](C)(C2)C(=O)Nc3cccc4C(=NNC(=O)c34)Cc5ccc(F)c(c5)C(=O)N6CCN(CC6)C(=O)C7CC7 | Olaparib 527 |
| Fc1ccc(CC2=NNC(=O)c3c(OC(=O)c4cn(nn4)c5ccc(Cl)cc5)cccc23)cc1C(=O)N6CCN(CC6)C(=O)C7CC7 | Olaparib 1546 |
| OC[C@H]1[C@@H]2C[C@@H](C=C2)[C@H]1COC(=O)c3cccc4C(=NNC(=O)c34)Cc5ccc(F)c(c5)C(=O)N6CCN(CC6)C(=O)C7CC7 | Olaparib 1114 |
| CN(C)C[C@H]1CCCC[C@]1(O)c2cccc(Oc3cccc4C(=NNC(=O)c34)Cc5ccc(F)c(c5)C(=O)N6CCN(CC6)C(=O)C7CC7)c2 | Olaparib 3053 |
| OC[C@@H](CN1CCCCC1)Oc2cccc3C(=NNC(=O)c23)Cc4ccc(F)c(c4)C(=O)N5CCN(CC5)C(=O)C6CC6 | Olaparib 2949 |
| Fc1ccc(CC2=NNC(=O)c3c(cccc23)C(=O)OC[C@H]4CCCN4Cc5ccc6CCCc6c5)cc1C(=O)N7CCN(CC7)C(=O)C8CC8 | Olaparib 1150 |
| C[C@@H](NC(=O)N1CCSC[C@@H]1C)C(=O)Oc2cccc3C(=NNC(=O)c23)Cc4ccc(F)c(c4)C(=O)N5CCN(CC5)C(=O)C6CC6 | Olaparib 1418 |
| CC(C)(COc1cccc2C(=NNC(=O)c12)Cc3ccc(F)c(c3)C(=O)N4CCN(CC4)C(=O)C5CC5)NC(=O)c6ccccc6 | Olaparib 2880 |
| Fc1ccc(CC2=NNC(=O)c3c(cccc23)C(=O)NC[C@@H]4CC(=NO4)c5c(F)cccc5F)cc1C(=O)N6CCN(CC6)C(=O)C7CC7 | Olaparib 231 |
| Oc1nc(OC(=O)c2cccc3C(=NNC(=O)c23)Cc4ccc(F)c(c4)C(=O)N5CCN(CC5)C(=O)C6CC6)nc7ccccc17 | Olaparib 1105 |
| C[C@H](NC(=O)c1cccc2C(=NNC(=O)c12)Cc3ccc(F)c(c3)C(=O)N4CCN(CC4)C(=O)C5CC5)[C@H]6CCCN(C6)C7C[C@H](C)C[C@H](C)C7 | Olaparib 209 |
| CN[C@H](C)C1CCN(Cc2ccc(Oc3cccc4C(=NNC(=O)c34)Cc5ccc(F)c(c5)C(=O)N6CCN(CC6)C(=O)C7CC7)cc2F)CC1 | Olaparib 2461 |
| C[C@H](CN1CCNCC1)Oc2cccc3C(=NNC(=O)c23)Cc4ccc(F)c(c4)C(=O)N5CCN(CC5)C(=O)C6CC6 | Olaparib 2988 |
| Nc1n[n+]([O-])c2ccc(Oc3cccc4C(=NNC(=O)c34)Cc5ccc(F)c(c5)C(=O)N6CCN(CC6)C(=O)C7CC7)cc2[n+]1[O-] | Olaparib 2582 |
| Fc1ccc(CC2=NNC(=O)c3c(OC(=O)\C=C\C(=O)Nc4ccccn4)cccc23)cc1C(=O)N5CCN(CC5)C(=O)C6CC6 | Olaparib 1462 |
| CC(C)(NC(=O)c1cccc2C(=NNC(=O)c12)Cc3ccc(F)c(c3)C(=O)N4CCN(CC4)C(=O)C5CC5)c6cc(F)c(N)cc6Cl | Olaparib 147 |
| Fc1ccc(CC2=NNC(=O)c3c(cccc23)C(=O)OC[C@@H]4CN(Cc5ccccc5)CCO4)cc1C(=O)N6CCN(CC6)C(=O)C7CC7 | Olaparib 1045 |
| Cc1ccc(cc1Oc2cccc3C(=NNC(=O)c23)Cc4ccc(F)c(c4)C(=O)N5CCN(CC5)C(=O)C6CC6)n7cc(nn7)C(C)(C)N | Olaparib 2541 |
| C[C@H](NC(=O)c1cccc2C(=NNC(=O)c12)Cc3ccc(F)c(c3)C(=O)N4CCN(CC4)C(=O)C5CC5)C6CCN(CC6)[C@H](C)C7CCCCC7 | Olaparib 208 |
| C[C@@](N)(C1CCOCC1)c2cccc(Oc3cccc4C(=NNC(=O)c34)Cc5ccc(F)c(c5)C(=O)N6CCN(CC6)C(=O)C7CC7)c2 | Olaparib 2558 |
| C[C@@H](COc1cccc2C(=NNC(=O)c12)Cc3ccc(F)c(c3)C(=O)N4CCN(CC4)C(=O)C5CC5)O[C@H]6CCCCO6 | Olaparib 2948 |
| CN([C@@H]1CCS(=O)(=O)C1)C(=O)c2ccc(Oc3cccc4C(=NNC(=O)c34)Cc5ccc(F)c(c5)C(=O)N6CCN(CC6)C(=O)C7CC7)nc2 | Olaparib 2452 |
| OC(=O)c1ccc(NC(=O)NCC#C)c(c1)c2cccc3C(=NNC(=O)c23)Cc4ccc(F)c(c4)C(=O)N5CCN(CC5)C(=O)C6CC6 | Olaparib 1660 |
| CC(C)(NC(=O)c1cccc2C(=NNC(=O)c12)Cc3ccc(F)c(c3)C(=O)N4CCN(CC4)C(=O)C5CC5)c6cc(F)c(Cl)cc6O | Olaparib 146 |
| Fc1ccc(CC2=NNC(=O)c3c(NC(=O)C[C@H]4CCCN(C4)c5ccncc5)cccc23)cc1C(=O)N6CCN(CC6)C(=O)C7CC7 | Olaparib 532 |
| C[C@@H](CCc1nc2cccnc2n1C)NC(=O)c3cccc4C(=NNC(=O)c34)Cc5ccc(F)c(c5)C(=O)N6CCN(CC6)C(=O)C7CC7 | Olaparib 236 |
| C[C@@H](NC(=O)c1cccc2C(=NNC(=O)c12)Cc3ccc(F)c(c3)C(=O)N4CCN(CC4)C(=O)C5CC5)[C@H]6CCCN(C6)[C@@H]7CCCC[C@H]7C | Olaparib 225 |
| C[C@@H](O)c1ccc(N2CCC(CC2)Oc3cccc4C(=NNC(=O)c34)Cc5ccc(F)c(c5)C(=O)N6CCN(CC6)C(=O)C7CC7)c(F)c1 | Olaparib 3062 |
| Fc1ccc(CC2=NNC(=O)c3c(OCCN4CCOCC4)cccc23)cc1C(=O)N5CCN(CC5)C(=O)C6CC6 | Olaparib 2748 |
| CC1=CN([C@H]2CC[C@@H](COC(=O)c3cccc4C(=NNC(=O)c34)Cc5ccc(F)c(c5)C(=O)N6CCN(CC6)C(=O)C7CC7)O2)C(=O)NC1=O | Olaparib 1015 |
| Fc1ccc(CC2=NNC(=O)c3c(NC(=O)CNC(=O)c4cccc(c4)C#N)cccc23)cc1C(=O)N5CCN(CC5)C(=O)C6CC6 | Olaparib 477 |
| CC(C)(C(=O)O)c1ccc(F)c(Oc2cccc3C(=NNC(=O)c23)Cc4ccc(F)c(c4)C(=O)N5CCN(CC5)C(=O)C6CC6)c1F | Olaparib 2227 |
| Oc1ccc(F)c2CCN(CCc12)C(=O)C(F)(F)Oc3cccc4C(=NNC(=O)c34)Cc5ccc(F)c(c5)C(=O)N6CCN(CC6)C(=O)C7CC7 | Olaparib 2438 |
| Cc1nn(C)c(NCc2ccc(Oc3cccc4C(=NNC(=O)c34)Cc5ccc(F)c(c5)C(=O)N6CCN(CC6)C(=O)C7CC7)c(Cl)c2)c1C#N | Olaparib 2432 |
| C[C@@H](NC(=O)c1cccc2C(=NNC(=O)c12)Cc3ccc(F)c(c3)C(=O)N4CCN(CC4)C(=O)C5CC5)[C@H]6CCCN(C6)[C@@H]7CCC[C@H](C)C7 | Olaparib 224 |
| CN1CC[C@@H](N)[C@@H]1c2ccc(Oc3cccc4C(=NNC(=O)c34)Cc5ccc(F)c(c5)C(=O)N6CCN(CC6)C(=O)C7CC7)c(Cl)c2 | Olaparib 2522 |
| C[C@H](OC(=O)c1cccc2C(=NNC(=O)c12)Cc3ccc(F)c(c3)C(=O)N4CCN(CC4)C(=O)C5CC5)c6cccc(c6)C(F)(F)F | Olaparib 737 |
| NC1=NC(=O)N(C=C1)[C@H]2CC[C@@H](COC(=O)c3cccc4C(=NNC(=O)c34)Cc5ccc(F)c(c5)C(=O)N6CCN(CC6)C(=O)C7CC7)O2 | Olaparib 1040 |
| Fc1ccc(CC2=NNC(=O)c3c(OC(=O)CNC(=O)c4cccc(c4)C#N)cccc23)cc1C(=O)N5CCN(CC5)C(=O)C6CC6 | Olaparib 1447 |
| CC(C)(C(=O)O)c1cc(Oc2cccc3C(=NNC(=O)c23)Cc4ccc(F)c(c4)C(=O)N5CCN(CC5)C(=O)C6CC6)ccc1F | Olaparib 2187 |
| OC(=O)C1(CC1)c2cc(Oc3cccc4C(=NNC(=O)c34)Cc5ccc(F)c(c5)C(=O)N6CCN(CC6)C(=O)C7CC7)c(F)cc2O | Olaparib 2574 |
| Cc1cn(nc1N)c2cc(Oc3cccc4C(=NNC(=O)c34)Cc5ccc(F)c(c5)C(=O)N6CCN(CC6)C(=O)C7CC7)ccc2C#N | Olaparib 2544 |
| C[C@@]12CC[C@@](CC(=O)Nc3cccc4C(=NNC(=O)c34)Cc5ccc(F)c(c5)C(=O)N6CCN(CC6)C(=O)C7CC7)(C[C@H]1O)C2(C)C | Olaparib 535 |
| Cc1cnccc1N2CCC[C@@H](C2)Oc3cccc4C(=NNC(=O)c34)Cc5ccc(F)c(c5)C(=O)N6CCN(CC6)C(=O)C7CC7 | Olaparib 3108 |
| Cc1ccc(cc1)[C@@H](C(=O)Oc2cccc3C(=NNC(=O)c23)Cc4ccc(F)c(c4)C(=O)N5CCN(CC5)C(=O)C6CC6)C(F)(F)F | Olaparib 1429 |
| NC1=N[C@@H]2O[C@H](COC(=O)c3cccc4C(=NNC(=O)c34)Cc5ccc(F)c(c5)C(=O)N6CCN(CC6)C(=O)C7CC7)[C@@H](O)[C@@H]2O1 | Olaparib 1098 |
| CSc1cc(c(O)cc1C)[C@@](C)(N)C(=O)Nc2cccc3C(=NNC(=O)c23)Cc4ccc(F)c(c4)C(=O)N5CCN(CC5)C(=O)C6CC6 | Olaparib 458 |
| CN(CC(=O)Nc1cccc2C(=NNC(=O)c12)Cc3ccc(F)c(c3)C(=O)N4CCN(CC4)C(=O)C5CC5)c6ccccc6F | Olaparib 496 |
| OC(=O)C1(CC1)c2cc(F)c(Oc3cccc4C(=NNC(=O)c34)Cc5ccc(F)c(c5)C(=O)N6CCN(CC6)C(=O)C7CC7)cc2O | Olaparib 2573 |
| OC(=O)C1(CC1)c2cc(F)c(Oc3cccc4C(=NNC(=O)c34)Cc5ccc(F)c(c5)C(=O)N6CCN(CC6)C(=O)C7CC7)cc2O | Olaparib 2573 |
| Nc1nc(N)c2c(Oc3cccc4C(=NNC(=O)c34)Cc5ccc(F)c(c5)C(=O)N6CCN(CC6)C(=O)C7CC7)cccc2n1 | Olaparib 2532 |
| C[C@@H]1CC[C@@H](CCN2C[C@@H](CC2=O)Oc3cccc4C(=NNC(=O)c34)Cc5ccc(F)c(c5)C(=O)N6CCN(CC6)C(=O)C7CC7)CC1 | Olaparib 3074 |
| Fc1ccc(CC2=NNC(=O)c3c(Oc4ccc(CN5CCOCC5)cc4)cccc23)cc1C(=O)N6CCN(CC6)C(=O)C7CC7 | Olaparib 2488 |
| Cc1cc(cc(c1)c2cccc3C(=NNC(=O)c23)Cc4ccc(F)c(c4)C(=O)N5CCN(CC5)C(=O)C6CC6)C(=O)O | Olaparib 1970 |
| CO[C@H]1O[C@H](COC(=O)c2cccc3C(=NNC(=O)c23)Cc4ccc(F)c(c4)C(=O)N5CCN(CC5)C(=O)C6CC6)[C@@H](O)[C@@H]7OC(C)(C)O[C@H]17 | Olaparib 1004 |
| COC(=O)COc1cnnc(Cl)c1c2cccc3C(=NNC(=O)c23)Cc4ccc(F)c(c4)C(=O)N5CCN(CC5)C(=O)C6CC6 | Olaparib 1659 |
| N[C@@H](Cc1cccc(c1)c2cccc3C(=NNC(=O)c23)Cc4ccc(F)c(c4)C(=O)N5CCN(CC5)C(=O)C6CC6)C(=O)O | Olaparib 1701 |
| Oc1c(Oc2cccc3C(=NNC(=O)c23)Cc4ccc(F)c(c4)C(=O)N5CCN(CC5)C(=O)C6CC6)c(F)ccc1[C@@H]7CCC(=O)N7 | Olaparib 2580 |
| CC1(C)CCCN(C1)c2cc(ccn2)C(=N)NC(=O)c3cccc4C(=NNC(=O)c34)Cc5ccc(F)c(c5)C(=O)N6CCN(CC6)C(=O)C7CC7 | Olaparib 215 |
| Fc1ccc(CC2=NNC(=O)c3c(cccc23)[C@H]4CCCN4C(=O)CNC5CCCC5)cc1C(=O)N6CCN(CC6)C(=O)C7CC7 | Olaparib 2052 |
| NC1(CCC1)c2ccc(O)c(Oc3cccc4C(=NNC(=O)c34)Cc5ccc(F)c(c5)C(=O)N6CCN(CC6)C(=O)C7CC7)c2F | Olaparib 2597 |
| Fc1ccc(CC2=NNC(=O)c3c(OC(=O)Cc4c(F)c(F)c(F)c(F)c4F)cccc23)cc1C(=O)N5CCN(CC5)C(=O)C6CC6 | Olaparib 1423 |
| C[C@@H]1OC[C@H]2O[C@H](O)[C@@H](OC(=O)c3cccc4C(=NNC(=O)c34)Cc5ccc(F)c(c5)C(=O)N6CCN(CC6)C(=O)C7CC7)[C@@H](O)[C@@H]2O1 | Olaparib 1047 |
| CCNC(=O)Cn1cc(c(N)n1)c2cccc3C(=NNC(=O)c23)Cc4ccc(F)c(c4)C(=O)N5CCN(CC5)C(=O)C6CC6 | Olaparib 1683 |
| NC(=N)SCc1cccc(c1)C(F)(F)c2cccc3C(=NNC(=O)c23)Cc4ccc(F)c(c4)C(=O)N5CCN(CC5)C(=O)C6CC6 | Olaparib 1662 |
| Oc1c(Oc2cccc3C(=NNC(=O)c23)Cc4ccc(F)c(c4)C(=O)N5CCN(CC5)C(=O)C6CC6)c(F)ccc1[C@@H]7CCC(=O)N7 | Olaparib 2578 |
| CO[C@H]1CCC[C@@H]1NC(=O)c2ccc(N)c(Oc3cccc4C(=NNC(=O)c34)Cc5ccc(F)c(c5)C(=O)N6CCN(CC6)C(=O)C7CC7)c2 | Olaparib 2459 |
| C[C@H]1CCC[C@H](C1)N2C[C@@H](CC2=O)Oc3cccc4C(=NNC(=O)c34)Cc5ccc(F)c(c5)C(=O)N6CCN(CC6)C(=O)C7CC7 | Olaparib 3102 |
| Nc1ccc(cc1Oc2cccc3C(=NNC(=O)c23)Cc4ccc(F)c(c4)C(=O)N5CCN(CC5)C(=O)C6CC6)C7(CC7)C#N | Olaparib 2618 |
| CC(C)(N)c1ccc(F)c(Oc2cccc3C(=NNC(=O)c23)Cc4ccc(F)c(c4)C(=O)N5CCN(CC5)C(=O)C6CC6)c1 | Olaparib 2919 |
| C[C@@H]1OC[C@H]2O[C@H](OC(=O)c3cccc4C(=NNC(=O)c34)Cc5ccc(F)c(c5)C(=O)N6CCN(CC6)C(=O)C7CC7)[C@H](O)[C@@H](O)[C@@H]2O1 | Olaparib 1048 |
| CC[C@H](NC(=O)c1cccc2C(=NNC(=O)c12)Cc3ccc(F)c(c3)C(=O)N4CCN(CC4)C(=O)C5CC5)c6nnc[nH]6 | Olaparib 196 |
| CC(=O)c1cc(O)c(C)cc1Oc2cccc3C(=NNC(=O)c23)Cc4ccc(F)c(c4)C(=O)N5CCN(CC5)C(=O)C6CC6 | Olaparib 2931 |
| NCC[C@H]1CCCN(C1)C(=O)c2oc(cc2)c3cccc4C(=NNC(=O)c34)Cc5ccc(F)c(c5)C(=O)N6CCN(CC6)C(=O)C7CC7 | Olaparib 1743 |
| COc1ccc(Oc2cccc3C(=NNC(=O)c23)Cc4ccc(F)c(c4)C(=O)N5CCN(CC5)C(=O)C6CC6)cc1NC(=O)[C@@]7(C)CCNC7 | Olaparib 2458 |
| Fc1ccc(CC2=NNC(=O)c3c(cccc23)C(=O)O[C@@H]4CCCC[C@H]4N5CCCC5)cc1C(=O)N6CCN(CC6)C(=O)C7CC7 | Olaparib 1100 |
| OC[C@@H]1[C@H]2C[C@H](C=C2)[C@@H]1COC(=O)c3cccc4C(=NNC(=O)c34)Cc5ccc(F)c(c5)C(=O)N6CCN(CC6)C(=O)C7CC7 | Olaparib 1115 |
| CC(C)(N)c1ccc(Oc2cccc3C(=NNC(=O)c23)Cc4ccc(F)c(c4)C(=O)N5CCN(CC5)C(=O)C6CC6)c(F)c1 | Olaparib 2339 |
| Nc1nnc(COc2ccc(Oc3cccc4C(=NNC(=O)c34)Cc5ccc(F)c(c5)C(=O)N6CCN(CC6)C(=O)C7CC7)cc2Cl)s1 | Olaparib 2441 |
| Oc1nc(OC(=O)c2cccc3C(=NNC(=O)c23)Cc4ccc(F)c(c4)C(=O)N5CCN(CC5)C(=O)C6CC6)c(Br)s1 | Olaparib 728 |
| CCC(=O)Nc1ccc(Oc2cccc3C(=NNC(=O)c23)Cc4ccc(F)c(c4)C(=O)N5CCN(CC5)C(=O)C6CC6)c(F)c1 | Olaparib 2893 |
| C[C@@H](N1C[C@@H](CC1=O)Oc2cccc3C(=NNC(=O)c23)Cc4ccc(F)c(c4)C(=O)N5CCN(CC5)C(=O)C6CC6)c7occc7 | Olaparib 3106 |
| C[C@H]1CC[C@H](CNC(=O)c2cccc3C(=NNC(=O)c23)Cc4ccc(F)c(c4)C(=O)N5CCN(CC5)C(=O)C6CC6)CN1c7ccc(Cl)nn7 | Olaparib 205 |
| C[C@H]1CCC[C@H]1N2C[C@@H](CC2=O)Oc3cccc4C(=NNC(=O)c34)Cc5ccc(F)c(c5)C(=O)N6CCN(CC6)C(=O)C7CC7 | Olaparib 3118 |
| C[C@H]1CC(C)(C)C[C@]2(C1)C[C@H](CCO2)OC(=O)c3cccc4C(=NNC(=O)c34)Cc5ccc(F)c(c5)C(=O)N6CCN(CC6)C(=O)C7CC7 | Olaparib 1034 |
| CC(C)(C#N)c1cc(F)c(Oc2cccc3C(=NNC(=O)c23)Cc4ccc(F)c(c4)C(=O)N5CCN(CC5)C(=O)C6CC6)c(F)c1 | Olaparib 2874 |
| C[C@H](OC(=O)c1cccc2C(=NNC(=O)c12)Cc3ccc(F)c(c3)C(=O)N4CCN(CC4)C(=O)C5CC5)c6onc(n6)c7cocc7 | Olaparib 1084 |
| Fc1ccc(CC2=NNC(=O)c3c(NC(=O)\C=C\CN4N=CC=CC4=O)cccc23)cc1C(=O)N5CCN(CC5)C(=O)C6CC6 | Olaparib 498 |
| N[C@@H](C(=O)Nc1cccc2C(=NNC(=O)c12)Cc3ccc(F)c(c3)C(=O)N4CCN(CC4)C(=O)C5CC5)c6ccc(Br)c(F)c6 | Olaparib 455 |
| Fc1ccc(CC2=NNC(=O)c3c(OCCc4ccc5NCCNc5n4)cccc23)cc1C(=O)N6CCN(CC6)C(=O)C7CC7 | Olaparib 3120 |
| C[C@@H]1CCCN1C(=O)c2sccc2C#CCOC(=O)c3cccc4C(=NNC(=O)c34)Cc5ccc(F)c(c5)C(=O)N6CCN(CC6)C(=O)C7CC7 | Olaparib 987 |
| Fc1ccc(CC2=NNC(=O)c3c(O[C@H]4CN(C5CCCCC5)C(=O)C4)cccc23)cc1C(=O)N6CCN(CC6)C(=O)C7CC7 | Olaparib 3117 |
| C[C@H]1CCC[C@@H](CCN2C[C@H](CC2=O)Oc3cccc4C(=NNC(=O)c34)Cc5ccc(F)c(c5)C(=O)N6CCN(CC6)C(=O)C7CC7)C1 | Olaparib 3075 |
| COc1c(C)nc(Cl)cc1c2cccc3C(=NNC(=O)c23)Cc4ccc(F)c(c4)C(=O)N5CCN(CC5)C(=O)C6CC6 | Olaparib 1963 |
| CC1=CN(N=C(C(=O)O)C1=O)c2cccc(Oc3cccc4C(=NNC(=O)c34)Cc5ccc(F)c(c5)C(=O)N6CCN(CC6)C(=O)C7CC7)c2 | Olaparib 2472 |
| CC(=O)CN1N=CC(=C(Cl)C1=O)Oc2cccc3C(=NNC(=O)c23)Cc4ccc(F)c(c4)C(=O)N5CCN(CC5)C(=O)C6CC6 | Olaparib 2259 |
| CCOC(=O)c1cc(Cl)cc(Oc2cccc3C(=NNC(=O)c23)Cc4ccc(F)c(c4)C(=O)N5CCN(CC5)C(=O)C6CC6)c1 | Olaparib 2263 |
| Fc1ccc(CC2=NNC(=O)c3c(NC(=O)CN4CC[N@]5CCCC[C@@H]5C4)cccc23)cc1C(=O)N6CCN(CC6)C(=O)C7CC7 | Olaparib 541 |
| COc1cc(cc(Oc2cccc3C(=NNC(=O)c23)Cc4ccc(F)c(c4)C(=O)N5CCN(CC5)C(=O)C6CC6)c1OC)[C@@H]7CCC(=O)N7 | Olaparib 3064 |
| Fc1ccc(CC2=NNC(=O)c3c(O[C@H]4CCOC5(CCOCC5)C4)cccc23)cc1C(=O)N6CCN(CC6)C(=O)C7CC7 | Olaparib 3128 |
| Fc1ccc(CC2=NNC(=O)c3c(Oc4ccc(\C=C\c5ccncc5)cc4)cccc23)cc1C(=O)N6CCN(CC6)C(=O)C7CC7 | Olaparib 3103 |
| CCOc1ncc(Br)cc1c2cccc3C(=NNC(=O)c23)Cc4ccc(F)c(c4)C(=O)N5CCN(CC5)C(=O)C6CC6 | Olaparib 1952 |
| Fc1ccc(CC2=NNC(=O)c3c(Oc4cccc(c4)S(=O)(=O)N5CCCC[C@H]5C=O)cccc23)cc1C(=O)N6CCN(CC6)C(=O)C7CC7 | Olaparib 2453 |
| NN1C(=NC(=CC1=O)C(F)(F)c2cccc3C(=NNC(=O)c23)Cc4ccc(F)c(c4)C(=O)N5CCN(CC5)C(=O)C6CC6)N | Olaparib 1690 |
| N[C@@H](C(=O)Oc1cccc2C(=NNC(=O)c12)Cc3ccc(F)c(c3)C(=O)N4CCN(CC4)C(=O)C5CC5)c6cc(Br)ccc6F | Olaparib 1402 |
| Fc1ccc(CC2=NNC(=O)c3c(O[C@H]4CN(C[C@H]5CCCCO5)C(=O)C4)cccc23)cc1C(=O)N6CCN(CC6)C(=O)C7CC7 | Olaparib 3101 |
| CC(=O)N1CCC(CC1)c2cncc(Oc3cccc4C(=NNC(=O)c34)Cc5ccc(F)c(c5)C(=O)N6CCN(CC6)C(=O)C7CC7)n2 | Olaparib 2530 |
| Fc1ccc(CC2=NNC(=O)c3c2cccc3c4ccnc(NCc5ccccc5)c4)cc1C(=O)N6CCN(CC6)C(=O)C7CC7 | Olaparib 2056 |
| Oc1ccc(cc1)c2cccc(Oc3cccc4C(=NNC(=O)c34)Cc5ccc(F)c(c5)C(=O)N6CCN(CC6)C(=O)C7CC7)c2 | Olaparib 3113 |
| Oc1cc(cc(c1)c2cccc3C(=NNC(=O)c23)Cc4ccc(F)c(c4)C(=O)N5CCN(CC5)C(=O)C6CC6)C#N | Olaparib 1991 |
| Fc1ccc(CC2=NNC(=O)c3c(OC(=O)CCN4C(=O)NC(=O)c5ccccc45)cccc23)cc1C(=O)N6CCN(CC6)C(=O)C7CC7 | Olaparib 1535 |
| CC[C@H](NC(=O)N1CCCCCCC1)C(=O)Oc2cccc3C(=NNC(=O)c23)Cc4ccc(F)c(c4)C(=O)N5CCN(CC5)C(=O)C6CC6 | Olaparib 1406 |
| FC(F)[C@@](F)(Oc1cccc(C=O)c1)Oc2cccc3C(=NNC(=O)c23)Cc4ccc(F)c(c4)C(=O)N5CCN(CC5)C(=O)C6CC6 | Olaparib 2254 |
| Fc1ccc(CC2=NNC(=O)c3c(O[C@H]4CN(C[C@H]5CCCO5)C(=O)C4)cccc23)cc1C(=O)N6CCN(CC6)C(=O)C7CC7 | Olaparib 3115 |
| OC1CN(CC(=O)Nc2ccc(Oc3cccc4C(=NNC(=O)c34)Cc5ccc(F)c(c5)C(=O)N6CCN(CC6)C(=O)C7CC7)cc2F)C1 | Olaparib 2482 |
| Oc1cccc(c1)c2ccc(Oc3cccc4C(=NNC(=O)c34)Cc5ccc(F)c(c5)C(=O)N6CCN(CC6)C(=O)C7CC7)cc2 | Olaparib 3114 |
| Oc1ncccc1c2cccc3C(=NNC(=O)c23)Cc4ccc(F)c(c4)C(=O)N5CCN(CC5)C(=O)C6CC6 | Olaparib 2024 |
| Fc1ccc(CC2=NNC(=O)c3c(cccc23)C(=O)Oc4ccc(cc4)N5C(=O)CSC5=S)cc1C(=O)N6CCN(CC6)C(=O)C7CC7 | Olaparib 1016 |
| Fc1ccc(CC2=NNC(=O)c3c(OC(=O)\C=C\C(=O)Nc4ccccc4)cccc23)cc1C(=O)N5CCN(CC5)C(=O)C6CC6 | Olaparib 1464 |
| OC(=O)Cc1cc(F)c(Oc2cccc3C(=NNC(=O)c23)Cc4ccc(F)c(c4)C(=O)N5CCN(CC5)C(=O)C6CC6)cc1F | Olaparib 2336 |
| N[C@]1(CC[C@H]2CNC[C@H]12)C(=O)Oc3cccc4C(=NNC(=O)c34)Cc5ccc(F)c(c5)C(=O)N6CCN(CC6)C(=O)C7CC7 | Olaparib 1571 |
| Fc1ccc(CC2=NNC(=O)c3c2cccc3c4cccc(n4)c5ccccn5)cc1C(=O)N6CCN(CC6)C(=O)C7CC7 | Olaparib 1906 |
| Fc1ccc(CC2=NNC(=O)c3c(Oc4cc(ccn4)C(=O)OC[C@H]5CCCCO5)cccc23)cc1C(=O)N6CCN(CC6)C(=O)C7CC7 | Olaparib 2531 |
| Fc1ccc2ccccc2c1OC(=O)c3cccc4C(=NNC(=O)c34)Cc5ccc(F)c(c5)C(=O)N6CCN(CC6)C(=O)C7CC7 | Olaparib 1103 |
| Fc1ccc(CC2=NNC(=O)c3c2cccc3c4cnccn4)cc1C(=O)N5CCN(CC5)C(=O)C6CC6 | Olaparib 1837 |
| Oc1cc(ccc1OC(=O)c2cccc3C(=NNC(=O)c23)Cc4ccc(F)c(c4)C(=O)N5CCN(CC5)C(=O)C6CC6)[C@H](C#N)N7CCOCC7 | Olaparib 1003 |
| OC(=O)\C=C\C(=O)c1ccc(F)c(Oc2cccc3C(=NNC(=O)c23)Cc4ccc(F)c(c4)C(=O)N5CCN(CC5)C(=O)C6CC6)c1 | Olaparib 2279 |
| OC(=O)Cc1ccc(Oc2cccc3C(=NNC(=O)c23)Cc4ccc(F)c(c4)C(=O)N5CCN(CC5)C(=O)C6CC6)c(F)c1 | Olaparib 2379 |
| Cc1cc(nnc1Oc2cccc3C(=NNC(=O)c23)Cc4ccc(F)c(c4)C(=O)N5CCN(CC5)C(=O)C6CC6)c7ccccc7 | Olaparib 2595 |
| Cc1ccc(s1)C2=NO[C@](C)(C2)C(=O)Oc3cccc4C(=NNC(=O)c34)Cc5ccc(F)c(c5)C(=O)N6CCN(CC6)C(=O)C7CC7 | Olaparib 1545 |
| Fc1ccc(CC2=NNC(=O)c3c(Oc4cccc(c4)n5cccc5)cccc23)cc1C(=O)N6CCN(CC6)C(=O)C7CC7 | Olaparib 3140 |
| Fc1ccc(CC2=NNC(=O)c3c(Oc4ccc5cccnc5c4)cccc23)cc1C(=O)N6CCN(CC6)C(=O)C7CC7 | Olaparib 2620 |
| Fc1ccc(CC2=NNC(=O)c3c2cccc3c4occn4)cc1C(=O)N5CCN(CC5)C(=O)C6CC6 | Olaparib 1911 |
| Fc1ccc(CC2=NNC(=O)c3c(cccc23)C(=O)OC[C@H]4CN(Cc5cscn5)C(=O)C4)cc1C(=O)N6CCN(CC6)C(=O)C7CC7 | Olaparib 1036 |
| C[C@H](NC(=O)c1ccccc1)C(=O)Nc2cccc3C(=NNC(=O)c23)Cc4ccc(F)c(c4)C(=O)N5CCN(CC5)C(=O)C6CC6 | Olaparib 486 |
| OC(=O)c1ccc(Oc2cccc3C(=NNC(=O)c23)Cc4ccc(F)c(c4)C(=O)N5CCN(CC5)C(=O)C6CC6)c(Br)c1 | Olaparib 2265 |
| Fc1ccc(CC2=NNC(=O)c3c(Oc4nc(Cl)c5cc(Br)ccc5n4)cccc23)cc1C(=O)N6CCN(CC6)C(=O)C7CC7 | Olaparib 2436 |
| Oc1ccc2c(OC[C@]2(O)CC(=O)Oc3cccc4C(=NNC(=O)c34)Cc5ccc(F)c(c5)C(=O)N6CCN(CC6)C(=O)C7CC7)c1 | Olaparib 1559 |
| Fc1ccc(CC2=NNC(=O)c3c(Oc4ccc5c(Cl)ccnc5c4)cccc23)cc1C(=O)N6CCN(CC6)C(=O)C7CC7 | Olaparib 2599 |
| Fc1ccc(CC2=NNC(=O)c3c(OCCc4cc5ccccc5[nH]4)cccc23)cc1C(=O)N6CCN(CC6)C(=O)C7CC7 | Olaparib 3135 |
| CC1=CC[C@H](CC(=O)Nc2cccc3C(=NNC(=O)c23)Cc4ccc(F)c(c4)C(=O)N5CCN(CC5)C(=O)C6CC6)C1(C)C | Olaparib 500 |
| Fc1ccc(CC2=NNC(=O)c3c(OC4=CN([C@@H]5CCCO5)C(=O)NC4=O)cccc23)cc1C(=O)N6CCN(CC6)C(=O)C7CC7 | Olaparib 2596 |
| C[C@H](NC(=O)c1ccccc1)C(=O)Oc2cccc3C(=NNC(=O)c23)Cc4ccc(F)c(c4)C(=O)N5CCN(CC5)C(=O)C6CC6 | Olaparib 1461 |
| OC(=O)c1cc(Cl)c(Cl)cc1Oc2cccc3C(=NNC(=O)c23)Cc4ccc(F)c(c4)C(=O)N5CCN(CC5)C(=O)C6CC6 | Olaparib 2243 |
| CC(C)[C@@H]1C[C@](Cc2cccc(C)c2)(CCO1)OC(=O)c3cccc4C(=NNC(=O)c34)Cc5ccc(F)c(c5)C(=O)N6CCN(CC6)C(=O)C7CC7 | Olaparib 988 |
| C[C@@H](NC(=O)c1cccc2C(=NNC(=O)c12)Cc3ccc(F)c(c3)C(=O)N4CCN(CC4)C(=O)C5CC5)c6cnc(s6)N7CCCC(C)(C)C7 | Olaparib 206 |
| Fc1ccc(CC2=NNC(=O)c3c(Oc4ccc5cc[nH]c5c4)cccc23)cc1C(=O)N6CCN(CC6)C(=O)C7CC7 | Olaparib 3145 |
| Fc1ccc(CC2=NNC(=O)c3c(NC(=O)C[C@@H]4CC4=C)cccc23)cc1C(=O)N5CCN(CC5)C(=O)C6CC6 | Olaparib 519 |
| CC1(C)O[C@H]2O[C@H](C=O)[C@H](OC(=O)c3cccc4C(=NNC(=O)c34)Cc5ccc(F)c(c5)C(=O)N6CCN(CC6)C(=O)C7CC7)[C@H]2O1 | Olaparib 1076 |
| Fc1ccc(CC2=NNC(=O)c3c(OC(=O)CNC(=O)c4ccccc4Cl)cccc23)cc1C(=O)N5CCN(CC5)C(=O)C6CC6 | Olaparib 1436 |
| OC(=O)c1ccc(Cl)c(Oc2cccc3C(=NNC(=O)c23)Cc4ccc(F)c(c4)C(=O)N5CCN(CC5)C(=O)C6CC6)c1 | Olaparib 2332 |
| C[C@H]1CC[C@H](CNC(=O)c2cccc3C(=NNC(=O)c23)Cc4ccc(F)c(c4)C(=O)N5CCN(CC5)C(=O)C6CC6)CN1Cc7cnc(C)s7 | Olaparib 207 |
| Fc1ccc(CC2=NNC(=O)c3c2cccc3c4cccc(NC5CCCCC5)n4)cc1C(=O)N6CCN(CC6)C(=O)C7CC7 | Olaparib 2058 |
| Fc1ccc(CC2=NNC(=O)c3c2cccc3c4cccc5ccnn45)cc1C(=O)N6CCN(CC6)C(=O)C7CC7 | Olaparib 2081 |
| Fc1ccc(CC2=NNC(=O)c3c(Oc4cccc5ccoc45)cccc23)cc1C(=O)N6CCN(CC6)C(=O)C7CC7 | Olaparib 3144 |
| Fc1ccc(CC2=NNC(=O)c3c(OC(=O)C[C@@H]4CC4=C)cccc23)cc1C(=O)N5CCN(CC5)C(=O)C6CC6 | Olaparib 1520 |
| O[C@H]1[C@H](O)[C@@H](OC(=O)c2cccc3C(=NNC(=O)c23)Cc4ccc(F)c(c4)C(=O)N5CCN(CC5)C(=O)C6CC6)[C@H]7OC[C@@H]1O7 | Olaparib 1106 |
| COc1ccc(cc1CC#N)C(C)(C)NC(=O)c2cccc3C(=NNC(=O)c23)Cc4ccc(F)c(c4)C(=O)N5CCN(CC5)C(=O)C6CC6 | Olaparib 145 |
| OC(=O)c1ccc(Oc2cccc3C(=NNC(=O)c23)Cc4ccc(F)c(c4)C(=O)N5CCN(CC5)C(=O)C6CC6)c(Cl)c1 | Olaparib 2333 |
| NCC[C@H]1CCCN(C1)C(=O)c2oc(Oc3cccc4C(=NNC(=O)c34)Cc5ccc(F)c(c5)C(=O)N6CCN(CC6)C(=O)C7CC7)cc2 | Olaparib 2487 |
| Fc1ccc(CC2=NNC(=O)c3c2cccc3c4csc5cc(Cl)ccc45)cc1C(=O)N6CCN(CC6)C(=O)C7CC7 | Olaparib 2060 |
| C[C@H]1C\C(=C/C(=O)Oc2cccc3C(=NNC(=O)c23)Cc4ccc(F)c(c4)C(=O)N5CCN(CC5)C(=O)C6CC6)\c7ccccc17 | Olaparib 1568 |
| Fc1ccc(CC2=NNC(=O)c3c(Oc4ccc(cc4)C5=CCNCC5)cccc23)cc1C(=O)N6CCN(CC6)C(=O)C7CC7 | Olaparib 3124 |
| C[C@H]1CCN(C\C=C\c2cccc3C(=NNC(=O)c23)Cc4ccc(F)c(c4)C(=O)N5CCN(CC5)C(=O)C6CC6)C[C@@H]1N | Olaparib 1696 |
| NC1=N[C@@H]2O[C@H](COc3cccc4C(=NNC(=O)c34)Cc5ccc(F)c(c5)C(=O)N6CCN(CC6)C(=O)C7CC7)[C@@H](O)[C@@H]2O1 | Olaparib 3125 |
| CC(C)(C)OC(=O)Nc1cnc(Cl)cc1c2cccc3C(=NNC(=O)c23)Cc4ccc(F)c(c4)C(=O)N5CCN(CC5)C(=O)C6CC6 | Olaparib 1951 |
| CC(C)(NC[C@@H](O)c1cccc(Oc2cccc3C(=NNC(=O)c23)Cc4ccc(F)c(c4)C(=O)N5CCN(CC5)C(=O)C6CC6)c1)C#C | Olaparib 2255 |
| C[C@@H](Cn1ccnc1)N2C[C@@H](CC2=O)Oc3cccc4C(=NNC(=O)c34)Cc5ccc(F)c(c5)C(=O)N6CCN(CC6)C(=O)C7CC7 | Olaparib 3090 |
| CS(=O)(=O)CCn1cc(Oc2cccc3C(=NNC(=O)c23)Cc4ccc(F)c(c4)C(=O)N5CCN(CC5)C(=O)C6CC6)c(N)n1 | Olaparib 2250 |
| CCN1CCN(Cc2cccc(c2)c3cccc4C(=NNC(=O)c34)Cc5ccc(F)c(c5)C(=O)N6CCN(CC6)C(=O)C7CC7)C[C@H]1C | Olaparib 2048 |
| CC1(CC1)C(=O)Oc2cccc3C(=NNC(=O)c23)Cc4ccc(F)c(c4)C(=O)N5CCN(CC5)C(=O)C6CC6 | Olaparib 1522 |
| CO[C@H]1O[C@H](CO)[C@@H](Oc2cccc3C(=NNC(=O)c23)Cc4ccc(F)c(c4)C(=O)N5CCN(CC5)C(=O)C6CC6)[C@@H]7OC(C)(C)O[C@H]17 | Olaparib 3070 |
| CC(C)(C(=O)O)c1cc(Oc2cccc3C(=NNC(=O)c23)Cc4ccc(F)c(c4)C(=O)N5CCN(CC5)C(=O)C6CC6)c(F)cc1O | Olaparib 2169 |
| C[C@H](N)COc1c(C)cc(Oc2cccc3C(=NNC(=O)c23)Cc4ccc(F)c(c4)C(=O)N5CCN(CC5)C(=O)C6CC6)cc1C | Olaparib 2877 |
| Oc1ccc(cc1Oc2cccc3C(=NNC(=O)c23)Cc4ccc(F)c(c4)C(=O)N5CCN(CC5)C(=O)C6CC6)[C@H](C#N)N7CCOCC7 | Olaparib 3068 |
| OC(=O)C1=C(Oc2cccc3C(=NNC(=O)c23)Cc4ccc(F)c(c4)C(=O)N5CCN(CC5)C(=O)C6CC6)C(=O)NC(=O)N1 | Olaparib 2373 |
| C[C@@H](C[C@H]1C[C@H](CCO1)Oc2cccc3C(=NNC(=O)c23)Cc4ccc(F)c(c4)C(=O)N5CCN(CC5)C(=O)C6CC6)c7ccccc7 | Olaparib 3081 |
| CC1(C)COC(C)(CCCc2cccc3C(=NNC(=O)c23)Cc4ccc(F)c(c4)C(=O)N5CCN(CC5)C(=O)C6CC6)OC1 | Olaparib 1867 |
| C[C@@H]1OC[C@H]2O[C@H](Oc3cccc4C(=NNC(=O)c34)Cc5ccc(F)c(c5)C(=O)N6CCN(CC6)C(=O)C7CC7)[C@H](O)[C@@H](O)[C@@H]2O1 | Olaparib 3096 |
| COc1ccc(CNC(=O)c2cccc3C(=NNC(=O)c23)Cc4ccc(F)c(c4)C(=O)N5CCN(CC5)C(=O)C6CC6)cc1OC | Olaparib 170 |
| CC(C)(C#N)c1ccc(Oc2cccc3C(=NNC(=O)c23)Cc4ccc(F)c(c4)C(=O)N5CCN(CC5)C(=O)C6CC6)cc1CO | Olaparib 2325 |
| Oc1cc(ccc1Oc2cccc3C(=NNC(=O)c23)Cc4ccc(F)c(c4)C(=O)N5CCN(CC5)C(=O)C6CC6)[C@H](C#N)N7CCOCC7 | Olaparib 3069 |
| NS(=O)(=O)c1ccc(CNC(=O)c2cccc3C(=NNC(=O)c23)Cc4ccc(F)c(c4)C(=O)N5CCN(CC5)C(=O)C6CC6)cc1 | Olaparib 157 |
| C[C@H]1CCN[C@H](C1)[C@@H](Oc2cccc3C(=NNC(=O)c23)Cc4ccc(F)c(c4)C(=O)N5CCN(CC5)C(=O)C6CC6)c7ccccc7 | Olaparib 3097 |
| C[C@H]1CCCN(C[C@H](Oc2cccc3C(=NNC(=O)c23)Cc4ccc(F)c(c4)C(=O)N5CCN(CC5)C(=O)C6CC6)C(F)(F)F)C1 | Olaparib 2868 |
| OC(=O)c1cc(COc2cccc(Oc3cccc4C(=NNC(=O)c34)Cc5ccc(F)c(c5)C(=O)N6CCN(CC6)C(=O)C7CC7)c2)ccn1 | Olaparib 2519 |
| CC(C)(C#N)c1ccc(C(=O)O)c(Oc2cccc3C(=NNC(=O)c23)Cc4ccc(F)c(c4)C(=O)N5CCN(CC5)C(=O)C6CC6)c1 | Olaparib 2251 |
| C[C@H](CO)Nc1ccc(cc1C#N)C(F)(F)c2cccc3C(=NNC(=O)c23)Cc4ccc(F)c(c4)C(=O)N5CCN(CC5)C(=O)C6CC6 | Olaparib 1654 |
| Cn1cc(CN2C[C@@H](CC2=O)Oc3cccc4C(=NNC(=O)c34)Cc5ccc(F)c(c5)C(=O)N6CCN(CC6)C(=O)C7CC7)cn1 | Olaparib 3105 |
| CN(C)C(=O)CN(C)C(=O)c1cncc(Oc2cccc3C(=NNC(=O)c23)Cc4ccc(F)c(c4)C(=O)N5CCN(CC5)C(=O)C6CC6)c1 | Olaparib 2856 |
| C[C@@H]([C@H]1C[C@H](CCO1)Oc2cccc3C(=NNC(=O)c23)Cc4ccc(F)c(c4)C(=O)N5CCN(CC5)C(=O)C6CC6)c7ccccc7 | Olaparib 3094 |
| CCc1ccoc1c2cccc3C(=NNC(=O)c23)Cc4ccc(F)c(c4)C(=O)N5CCN(CC5)C(=O)C6CC6 | Olaparib 2020 |
| C[C@@H](OC(=O)c1cccc2C(=NNC(=O)c12)Cc3ccc(F)c(c3)C(=O)N4CCN(CC4)C(=O)C5CC5)[C@H](C)c6onc(n6)c7ccccc7C | Olaparib 1006 |
| Nc1cc(ccc1Oc2cccc3C(=NNC(=O)c23)Cc4ccc(F)c(c4)C(=O)N5CCN(CC5)C(=O)C6CC6)C(=O)NCCC#C | Olaparib 2253 |
| OC[C@H](COc1cccc2C(=NNC(=O)c12)Cc3ccc(F)c(c3)C(=O)N4CCN(CC4)C(=O)C5CC5)c6ccccn6 | Olaparib 2963 |
| CN1CCCC[C@@H]1CN2C[C@H](COC(=O)c3cccc4C(=NNC(=O)c34)Cc5ccc(F)c(c5)C(=O)N6CCN(CC6)C(=O)C7CC7)CC2=O | Olaparib 1014 |
| Cc1oc(\C=N\NC(=S)NC(=O)c2cccc3C(=NNC(=O)c23)Cc4ccc(F)c(c4)C(=O)N5CCN(CC5)C(=O)C6CC6)cc1 | Olaparib 158 |
| CCc1ccccc1[C@@H]2C[C@@H](CCO2)Oc3cccc4C(=NNC(=O)c34)Cc5ccc(F)c(c5)C(=O)N6CCN(CC6)C(=O)C7CC7 | Olaparib 3092 |
| Cc1cscc1c2cccc3C(=NNC(=O)c23)Cc4ccc(F)c(c4)C(=O)N5CCN(CC5)C(=O)C6CC6 | Olaparib 2014 |
| Cn1c(OC(=O)c2cccc3C(=NNC(=O)c23)Cc4ccc(F)c(c4)C(=O)N5CCN(CC5)C(=O)C6CC6)nnc1c7cccc(c7)C(F)(F)F | Olaparib 993 |
| Nc1cc(C(=O)NCCC#C)c(F)cc1Oc2cccc3C(=NNC(=O)c23)Cc4ccc(F)c(c4)C(=O)N5CCN(CC5)C(=O)C6CC6 | Olaparib 2248 |
| Nc1c(F)c(N)c(F)c(Oc2cccc3C(=NNC(=O)c23)Cc4ccc(F)c(c4)C(=O)N5CCN(CC5)C(=O)C6CC6)c1F | Olaparib 2359 |
| Fc1ccc(CC2=NNC(=O)c3c(OC(=O)C[C@H]4CCCN(C4)C(=O)NC5CC5)cccc23)cc1C(=O)N6CCN(CC6)C(=O)C7CC7 | Olaparib 1543 |
| NC(=O)c1nc(F)cnc1OC(=O)c2cccc3C(=NNC(=O)c23)Cc4ccc(F)c(c4)C(=O)N5CCN(CC5)C(=O)C6CC6 | Olaparib 848 |
| Fc1ccc(CC2=NNC(=O)c3c(O[C@@H]4CC[C@H](CC4)OCc5ccccc5)cccc23)cc1C(=O)N6CCN(CC6)C(=O)C7CC7 | Olaparib 3093 |
| Cc1cc(C)cc(c1)[C@@H]2C[C@@H](CN2)Oc3cccc4C(=NNC(=O)c34)Cc5ccc(F)c(c5)C(=O)N6CCN(CC6)C(=O)C7CC7 | Olaparib 3109 |
| Fc1ccc(CC2=NNC(=O)c3c(cccc23)C(=C)C4CCOCC4)cc1C(=O)N5CCN(CC5)C(=O)C6CC6 | Olaparib 1998 |
| Oc1ccc(Oc2cccc3C(=NNC(=O)c23)Cc4ccc(F)c(c4)C(=O)N5CCN(CC5)C(=O)C6CC6)cc1NC(=O)c7occc7 | Olaparib 2536 |
| COC(=O)COc1cc(ccn1)c2cccc3C(=NNC(=O)c23)Cc4ccc(F)c(c4)C(=O)N5CCN(CC5)C(=O)C6CC6 | Olaparib 1686 |
| Nc1c(O)c(Cl)cc(Oc2cccc3C(=NNC(=O)c23)Cc4ccc(F)c(c4)C(=O)N5CCN(CC5)C(=O)C6CC6)c1Cl | Olaparib 2277 |
| CO[C@H]1CCC[C@H](C1)N2CCO[C@H](CNC(=O)c3cccc4C(=NNC(=O)c34)Cc5ccc(F)c(c5)C(=O)N6CCN(CC6)C(=O)C7CC7)C2 | Olaparib 219 |
| C[C@@H](C(=O)NC(C)(C)C)n1cc(Oc2cccc3C(=NNC(=O)c23)Cc4ccc(F)c(c4)C(=O)N5CCN(CC5)C(=O)C6CC6)c(N)n1 | Olaparib 2212 |
| Cc1ccccc1[C@@H]2C[C@@H](CN2)Oc3cccc4C(=NNC(=O)c34)Cc5ccc(F)c(c5)C(=O)N6CCN(CC6)C(=O)C7CC7 | Olaparib 3122 |
| Fc1ccc(CC2=NNC(=O)c3c(Oc4ccc(cc4)C5(CCC5)C#N)cccc23)cc1C(=O)N6CCN(CC6)C(=O)C7CC7 | Olaparib 3126 |
| OS(=O)(=O)CC(F)(F)c1cccc2C(=NNC(=O)c12)Cc3ccc(F)c(c3)C(=O)N4CCN(CC4)C(=O)C5CC5 | Olaparib 1617 |
| Cc1cc(NCc2nonc2C)ccc1Oc3cccc4C(=NNC(=O)c34)Cc5ccc(F)c(c5)C(=O)N6CCN(CC6)C(=O)C7CC7 | Olaparib 2565 |
| Fc1ccc(CC2=NNC(=O)c3c(OCCNC(=O)c4cccnc4)cccc23)cc1C(=O)N5CCN(CC5)C(=O)C6CC6 | Olaparib 2930 |
| Nc1c(Cl)cc(cc1Oc2cccc3C(=NNC(=O)c23)Cc4ccc(F)c(c4)C(=O)N5CCN(CC5)C(=O)C6CC6)C#N | Olaparib 2340 |
| Oc1ccccc1C(=O)CC(=O)c2ccc(Oc3cccc4C(=NNC(=O)c34)Cc5ccc(F)c(c5)C(=O)N6CCN(CC6)C(=O)C7CC7)cc2 | Olaparib 2443 |
| CC(C)[C@H](C)NC(=O)Cn1cc(Oc2cccc3C(=NNC(=O)c23)Cc4ccc(F)c(c4)C(=O)N5CCN(CC5)C(=O)C6CC6)c(N)n1 | Olaparib 2213 |
| Fc1ccc(CC2=NNC(=O)c3c2cccc3c4ccncc4NC5CC5)cc1C(=O)N6CCN(CC6)C(=O)C7CC7 | Olaparib 2076 |
| Fc1ccc(CC2=NNC(=O)c3c(Cc4ccc5OCOc5c4)cccc23)cc1C(=O)N6CCN(CC6)C(=O)C7CC7 | Olaparib 1891 |
| CC(C)[C@H](O)C(=C)CNC(=O)c1cccc2C(=NNC(=O)c12)Cc3ccc(F)c(c3)C(=O)N4CCN(CC4)C(=O)C5CC5 | Olaparib 34 |
| Cc1nonc1CNCc2cccc(Oc3cccc4C(=NNC(=O)c34)Cc5ccc(F)c(c5)C(=O)N6CCN(CC6)C(=O)C7CC7)c2 | Olaparib 2535 |
| Nc1c(F)c(F)nc(CC(=O)Nc2cccc3C(=NNC(=O)c23)Cc4ccc(F)c(c4)C(=O)N5CCN(CC5)C(=O)C6CC6)c1F | Olaparib 476 |
| Fc1ccc(CC2=NNC(=O)c3c(Oc4cccc(Cl)c4OCC#N)cccc23)cc1C(=O)N5CCN(CC5)C(=O)C6CC6 | Olaparib 2309 |
| Fc1ccc(CC2=NNC(=O)c3c(cccc23)C(=O)OCc4cncc(c4)c5ccccc5C#N)cc1C(=O)N6CCN(CC6)C(=O)C7CC7 | Olaparib 1041 |
| CCNC(=O)Cn1cc(Oc2cccc3C(=NNC(=O)c23)Cc4ccc(F)c(c4)C(=O)N5CCN(CC5)C(=O)C6CC6)c(N)n1 | Olaparib 2307 |
| Fc1ccc(CC2=NNC(=O)c3c(Cc4cc5OCOc5cc4Cl)cccc23)cc1C(=O)N6CCN(CC6)C(=O)C7CC7 | Olaparib 1889 |
| Fc1ccc(CC2=NNC(=O)c3c(Oc4ccc5COCc5c4)cccc23)cc1C(=O)N6CCN(CC6)C(=O)C7CC7 | Olaparib 3143 |
| CCO\C(=C\C(=O)Nc1cccc2C(=NNC(=O)c12)Cc3ccc(F)c(c3)C(=O)N4CCN(CC4)C(=O)C5CC5)\C | Olaparib 363 |
| Cc1cnc2c(Cl)nc(Oc3cccc4C(=NNC(=O)c34)Cc5ccc(F)c(c5)C(=O)N6CCN(CC6)C(=O)C7CC7)nc2n1 | Olaparib 2571 |
| Fc1ccc(cn1)C(=O)NCCOc2cccc3C(=NNC(=O)c23)Cc4ccc(F)c(c4)C(=O)N5CCN(CC5)C(=O)C6CC6 | Olaparib 2890 |
| COc1nccc(C#N)c1c2cccc3C(=NNC(=O)c23)Cc4ccc(F)c(c4)C(=O)N5CCN(CC5)C(=O)C6CC6 | Olaparib 1972 |
| Fc1ccc(CC2=NNC(=O)c3c(cccc23)C(=O)Oc4cccc5ccc(C=O)nc45)cc1C(=O)N6CCN(CC6)C(=O)C7CC7 | Olaparib 1099 |
| Cc1cnn(O)c1[C@H](N)C(=O)Oc2cccc3C(=NNC(=O)c23)Cc4ccc(F)c(c4)C(=O)N5CCN(CC5)C(=O)C6CC6 | Olaparib 1483 |
| C[C@H]1CC(C)(C)C[C@]2(C1)C[C@H](CCO2)Oc3cccc4C(=NNC(=O)c34)Cc5ccc(F)c(c5)C(=O)N6CCN(CC6)C(=O)C7CC7 | Olaparib 3088 |
| Fc1ccc(CC2=NNC(=O)c3c(Cc4cc5OCOc5cc4Br)cccc23)cc1C(=O)N6CCN(CC6)C(=O)C7CC7 | Olaparib 1893 |
| CC(=C)C(=O)NCCOc1cccc2C(=NNC(=O)c12)Cc3ccc(F)c(c3)C(=O)N4CCN(CC4)C(=O)C5CC5 | Olaparib 2774 |
| Fc1ccc(CC2=NNC(=O)c3c(cccc23)C(=O)OCc4cccc5nonc45)cc1C(=O)N6CCN(CC6)C(=O)C7CC7 | Olaparib 1116 |
| N[C@@H](C(=O)Oc1cccc2C(=NNC(=O)c12)Cc3ccc(F)c(c3)C(=O)N4CCN(CC4)C(=O)C5CC5)c6cccnc6 | Olaparib 1507 |
| C[C@@H](OC(=O)c1cccc2C(=NNC(=O)c12)Cc3ccc(F)c(c3)C(=O)N4CCN(CC4)C(=O)C5CC5)c6ccc(c(F)c6)n7nc(C)c(C)c7C | Olaparib 990 |
| OC(=O)C(F)(F)c1occc1c2cccc3C(=NNC(=O)c23)Cc4ccc(F)c(c4)C(=O)N5CCN(CC5)C(=O)C6CC6 | Olaparib 1655 |
| CCn1cccc1C(=O)Nc2ccc(Oc3cccc4C(=NNC(=O)c34)Cc5ccc(F)c(c5)C(=O)N6CCN(CC6)C(=O)C7CC7)cc2C | Olaparib 3058 |
| N[C@@H](Cn1cncn1)C(=O)Oc2cccc3C(=NNC(=O)c23)Cc4ccc(F)c(c4)C(=O)N5CCN(CC5)C(=O)C6CC6 | Olaparib 1501 |
| Fc1ccc(CC2=NNC(=O)c3c(O[C@H]4CCO[C@H](C4)C5CCCC5)cccc23)cc1C(=O)N6CCN(CC6)C(=O)C7CC7 | Olaparib 3129 |
| Fc1ccc(CC2=NNC(=O)c3c(NC(=O)[C@@H]4C[C@@H]5C[C@H]4C=C5)cccc23)cc1C(=O)N6CCN(CC6)C(=O)C7CC7 | Olaparib 546 |
| COC(=O)\C(=C(/F)\Oc1cccc2C(=NNC(=O)c12)Cc3ccc(F)c(c3)C(=O)N4CCN(CC4)C(=O)C5CC5)\F | Olaparib 2156 |
| Fc1ccc(CC2=NNC(=O)c3c(cccc23)C(=O)Oc4ccc(cc4F)N5C(=O)CCCCC5=O)cc1C(=O)N6CCN(CC6)C(=O)C7CC7 | Olaparib 999 |
| Oc1cc(CNC(=O)c2cccc3C(=NNC(=O)c23)Cc4ccc(F)c(c4)C(=O)N5CCN(CC5)C(=O)C6CC6)ccn1 | Olaparib 198 |
| COC(=O)c1sccc1c2cccc3C(=NNC(=O)c23)Cc4ccc(F)c(c4)C(=O)N5CCN(CC5)C(=O)C6CC6 | Olaparib 1718 |
| Fc1ccc(CC2=NNC(=O)c3c(Oc4ccc(CNCc5ccnc(c5)C#N)cc4Cl)cccc23)cc1C(=O)N6CCN(CC6)C(=O)C7CC7 | Olaparib 2442 |
| COc1nc(OC)nc(Oc2cccc3C(=NNC(=O)c23)Cc4ccc(F)c(c4)C(=O)N5CCN(CC5)C(=O)C6CC6)n1 | Olaparib 2369 |
| Nc1ccc(cc1)c2cccc(c2F)c3cccc4C(=NNC(=O)c34)Cc5ccc(F)c(c5)C(=O)N6CCN(CC6)C(=O)C7CC7 | Olaparib 1765 |
| Fc1ccc(CC2=NNC(=O)c3c(OC(=O)[C@@H]4C[C@@H]5C[C@H]4C=C5)cccc23)cc1C(=O)N6CCN(CC6)C(=O)C7CC7 | Olaparib 1574 |
| CC(C)(C)C(=O)NC(C)(C)COc1cccc2C(=NNC(=O)c12)Cc3ccc(F)c(c3)C(=O)N4CCN(CC4)C(=O)C5CC5 | Olaparib 2750 |
| NC(=O)C1=Cc2cc(Oc3cccc4C(=NNC(=O)c34)Cc5ccc(F)c(c5)C(=O)N6CCN(CC6)C(=O)C7CC7)ccc2OC1=N | Olaparib 2560 |
| [O-]C(=O)COc1ccc(Oc2cccc3C(=NNC(=O)c23)Cc4ccc(F)c(c4)C(=O)N5CCN(CC5)C(=O)C6CC6)cc1Cl | Olaparib 2262 |
| Fc1ccc(CC2=NNC(=O)c3c2cccc3c4ccnc(n4)C#N)cc1C(=O)N5CCN(CC5)C(=O)C6CC6 | Olaparib 2010 |
| Cc1cccc(n1)[C@@H](OC(=O)c2cccc3C(=NNC(=O)c23)Cc4ccc(F)c(c4)C(=O)N5CCN(CC5)C(=O)C6CC6)c7occc7 | Olaparib 1072 |
| C[C@H](N(C)C(=O)C1=CNC(=O)C=C1)C(=O)Nc2cccc3C(=NNC(=O)c23)Cc4ccc(F)c(c4)C(=O)N5CCN(CC5)C(=O)C6CC6 | Olaparib 465 |
| Fc1ccc(CC2=NNC(=O)c3c2cccc3c4cccc(n4)c5ccccc5)cc1C(=O)N6CCN(CC6)C(=O)C7CC7 | Olaparib 2067 |
| Fc1ccc(CC2=NNC(=O)c3c(Oc4ccc5ccccc5c4)cccc23)cc1C(=O)N6CCN(CC6)C(=O)C7CC7 | Olaparib 3142 |
| CC(C)(C)[C@@H](CO)COC(=O)c1cccc2C(=NNC(=O)c12)Cc3ccc(F)c(c3)C(=O)N4CCN(CC4)C(=O)C5CC5 | Olaparib 586 |
| OC(=O)C1=Cc2cc(Oc3cccc4C(=NNC(=O)c34)Cc5ccc(F)c(c5)C(=O)N6CCN(CC6)C(=O)C7CC7)ccc2OC1=O | Olaparib 2457 |
| CCCCc1nc(Oc2cccc3C(=NNC(=O)c23)Cc4ccc(F)c(c4)C(=O)N5CCN(CC5)C(=O)C6CC6)c(C=O)[nH]1 | Olaparib 2341 |
| Fc1ccc(CC2=NNC(=O)c3c2cccc3c4nc(Cl)n[nH]4)cc1C(=O)N5CCN(CC5)C(=O)C6CC6 | Olaparib 1704 |
| Fc1ccc(CC2=NNC(=O)c3c(OC(=O)c4cn[nH]c4Cc5ccccc5Cl)cccc23)cc1C(=O)N6CCN(CC6)C(=O)C7CC7 | Olaparib 1532 |
| C[C@H](N(C)C(=O)C1=CNC(=O)C=C1)C(=O)Oc2cccc3C(=NNC(=O)c23)Cc4ccc(F)c(c4)C(=O)N5CCN(CC5)C(=O)C6CC6 | Olaparib 1425 |
| C[C@@H]1CCN(Cc2ccc(F)c(c2)c3cccc4C(=NNC(=O)c34)Cc5ccc(F)c(c5)C(=O)N6CCN(CC6)C(=O)C7CC7)C1 | Olaparib 2053 |
| CC(C)(C)SCCNC(=O)c1cccc2C(=NNC(=O)c12)Cc3ccc(F)c(c3)C(=O)N4CCN(CC4)C(=O)C5CC5 | Olaparib 19 |
| Oc1cc(OC(=O)c2cccc3C(=NNC(=O)c23)Cc4ccc(F)c(c4)C(=O)N5CCN(CC5)C(=O)C6CC6)cc7OC=CC(=O)c17 | Olaparib 1088 |
| COc1cnc(Oc2cccc3C(=NNC(=O)c23)Cc4ccc(F)c(c4)C(=O)N5CCN(CC5)C(=O)C6CC6)cn1 | Olaparib 2419 |
| Fc1ccc(CC2=NNC(=O)c3c2cccc3c4n[nH]c(Br)n4)cc1C(=O)N5CCN(CC5)C(=O)C6CC6 | Olaparib 1703 |
| Fc1ccc(Oc2cccc3C(=NNC(=O)c23)Cc4ccc(F)c(c4)C(=O)N5CCN(CC5)C(=O)C6CC6)cc1OCc7ccnc(c7)C#N | Olaparib 2520 |
| CCOC(=O)CN1N=CC(=C(Oc2cccc3C(=NNC(=O)c23)Cc4ccc(F)c(c4)C(=O)N5CCN(CC5)C(=O)C6CC6)C1=O)Cl | Olaparib 2202 |
| CC1(C)[C@@H]2CC[C@@]1(C)[C@H](C2)Oc3cccc4C(=NNC(=O)c34)Cc5ccc(F)c(c5)C(=O)N6CCN(CC6)C(=O)C7CC7 | Olaparib 3141 |
| CC(C)(CN)CNC(=O)c1cccc2C(=NNC(=O)c12)Cc3ccc(F)c(c3)C(=O)N4CCN(CC4)C(=O)C5CC5 | Olaparib 115 |
| Oc1cc(OC(=O)c2cccc3C(=NNC(=O)c23)Cc4ccc(F)c(c4)C(=O)N5CCN(CC5)C(=O)C6CC6)c7C(=O)C=COc7c1 | Olaparib 1089 |
| CCC[C@@H](C)Cn1cc(Oc2cccc3C(=NNC(=O)c23)Cc4ccc(F)c(c4)C(=O)N5CCN(CC5)C(=O)C6CC6)c(N)n1 | Olaparib 2311 |
| CC1=CC(=C(C#N)C(=O)N1)C(F)(F)c2cccc3C(=NNC(=O)c23)Cc4ccc(F)c(c4)C(=O)N5CCN(CC5)C(=O)C6CC6 | Olaparib 1684 |
| Cc1nc2ccccc2nc1OC(=O)c3cccc4C(=NNC(=O)c34)Cc5ccc(F)c(c5)C(=O)N6CCN(CC6)C(=O)C7CC7 | Olaparib 1111 |
| CC(=O)OCN1N=CC(=C(Cl)C1=O)Oc2cccc3C(=NNC(=O)c23)Cc4ccc(F)c(c4)C(=O)N5CCN(CC5)C(=O)C6CC6 | Olaparib 2223 |
| Fc1ccc(CC2=NNC(=O)c3c(O[C@H]4C[C@H]5CC[C@@H]4C5)cccc23)cc1C(=O)N6CCN(CC6)C(=O)C7CC7 | Olaparib 3146 |
| CC[C@@H](C)[C@@H](N)C(=O)Oc1cccc2C(=NNC(=O)c12)Cc3ccc(F)c(c3)C(=O)N4CCN(CC4)C(=O)C5CC5 | Olaparib 1281 |
| C[C@H](OC(=O)c1cccc2C(=NNC(=O)c12)Cc3ccc(F)c(c3)C(=O)N4CCN(CC4)C(=O)C5CC5)c6ccccc6NC(=O)[C@@H]7CNC[C@H]7C | Olaparib 989 |
| CC(C)(C)n1nnnc1c2cccc3C(=NNC(=O)c23)Cc4ccc(F)c(c4)C(=O)N5CCN(CC5)C(=O)C6CC6 | Olaparib 1717 |
| CC1=CC(=CC(=O)N1)Oc2cccc3C(=NNC(=O)c23)Cc4ccc(F)c(c4)C(=O)N5CCN(CC5)C(=O)C6CC6 | Olaparib 3018 |
| Oc1cc(OC(=O)c2cccc3C(=NNC(=O)c23)Cc4ccc(F)c(c4)C(=O)N5CCN(CC5)C(=O)C6CC6)nc7ccccc17 | Olaparib 1110 |
| CC(=O)OCN1N=CC(=C(Oc2cccc3C(=NNC(=O)c23)Cc4ccc(F)c(c4)C(=O)N5CCN(CC5)C(=O)C6CC6)C1=O)Cl | Olaparib 2222 |
| Fc1ccc(CC2=NNC(=O)c3c(cccc23)[C@H]4C[C@H]4c5cccc(Cl)c5)cc1C(=O)N6CCN(CC6)C(=O)C7CC7 | Olaparib 2069 |
| Fc1ccc(CC2=NNC(=O)c3c(cccc23)[C@H]4C[C@@H]5C[C@H]4C=C5)cc1C(=O)N6CCN(CC6)C(=O)C7CC7 | Olaparib 1843 |
| CC[C@@H](C)[C@@H](N)C(=O)Oc1cccc2C(=NNC(=O)c12)Cc3ccc(F)c(c3)C(=O)N4CCN(CC4)C(=O)C5CC5 | Olaparib 1279 |
| CC1(C)CCCN(C1)C(=O)c2ncccc2OC(=O)c3cccc4C(=NNC(=O)c34)Cc5ccc(F)c(c5)C(=O)N6CCN(CC6)C(=O)C7CC7 | Olaparib 1002 |
| COC1=C(C(=O)N(CC=C)N=C1)c2cccc3C(=NNC(=O)c23)Cc4ccc(F)c(c4)C(=O)N5CCN(CC5)C(=O)C6CC6 | Olaparib 1688 |
| Fc1ccc(CC2=NNC(=O)c3c2cccc3C4=CC(=CNC4=O)C#N)cc1C(=O)N5CCN(CC5)C(=O)C6CC6 | Olaparib 1990 |
| C[C@H](N)c1ccccc1n2nc(C)c(Oc3cccc4C(=NNC(=O)c34)Cc5ccc(F)c(c5)C(=O)N6CCN(CC6)C(=O)C7CC7)c2C | Olaparib 2512 |
| C[C@H]1CCCCCN1C(=O)N[C@H](CO)C(=O)Oc2cccc3C(=NNC(=O)c23)Cc4ccc(F)c(c4)C(=O)N5CCN(CC5)C(=O)C6CC6 | Olaparib 1404 |
| CS(=O)(=O)c1nc(cs1)c2cccc3C(=NNC(=O)c23)Cc4ccc(F)c(c4)C(=O)N5CCN(CC5)C(=O)C6CC6 | Olaparib 1910 |
| CN(C)CCCNC(=O)c1cccc2C(=NNC(=O)c12)Cc3ccc(F)c(c3)C(=O)N4CCN(CC4)C(=O)C5CC5 | Olaparib 114 |
| CCOc1cc(ccc1OC(=O)c2cccc3C(=NNC(=O)c23)Cc4ccc(F)c(c4)C(=O)N5CCN(CC5)C(=O)C6CC6)[C@@H]7CCC(=O)N7 | Olaparib 1026 |
| CC(=O)N1N=CC(=C(C1=O)c2cccc3C(=NNC(=O)c23)Cc4ccc(F)c(c4)C(=O)N5CCN(CC5)C(=O)C6CC6)Cl | Olaparib 1677 |
| CC1(C)CCCN(C\C=C\C(=O)Nc2cccc3C(=NNC(=O)c23)Cc4ccc(F)c(c4)C(=O)N5CCN(CC5)C(=O)C6CC6)C1 | Olaparib 484 |
| C[C@@H](N)c1cc(Oc2cccc3C(=NNC(=O)c23)Cc4ccc(F)c(c4)C(=O)N5CCN(CC5)C(=O)C6CC6)ccc1n7nc(C)cc7C | Olaparib 2542 |
| C[C@H](N(C)C(=O)NC1CCOCC1)C(=O)Nc2cccc3C(=NNC(=O)c23)Cc4ccc(F)c(c4)C(=O)N5CCN(CC5)C(=O)C6CC6 | Olaparib 463 |
| COCCSc1nsc(n1)c2cccc3C(=NNC(=O)c23)Cc4ccc(F)c(c4)C(=O)N5CCN(CC5)C(=O)C6CC6 | Olaparib 1675 |
| CC(C)[C@H](O)CNC(=O)c1cccc2C(=NNC(=O)c12)Cc3ccc(F)c(c3)C(=O)N4CCN(CC4)C(=O)C5CC5 | Olaparib 101 |
| CN(C(=O)[C@@H]1CCSC1)c2ccc(OC(=O)c3cccc4C(=NNC(=O)c34)Cc5ccc(F)c(c5)C(=O)N6CCN(CC6)C(=O)C7CC7)cc2 | Olaparib 998 |
| C[C@H]1CCC[C@H](C1)NC(=O)\C(=C(/C)\C(=O)Oc2cccc3C(=NNC(=O)c23)Cc4ccc(F)c(c4)C(=O)N5CCN(CC5)C(=O)C6CC6)\C | Olaparib 1413 |
| CC1(C)CCCN(C\C=C\C(=O)Oc2cccc3C(=NNC(=O)c23)Cc4ccc(F)c(c4)C(=O)N5CCN(CC5)C(=O)C6CC6)C1 | Olaparib 1457 |
| C[C@@H](N)c1cnn(Cc2cccc(Oc3cccc4C(=NNC(=O)c34)Cc5ccc(F)c(c5)C(=O)N6CCN(CC6)C(=O)C7CC7)c2)c1C | Olaparib 2511 |
| C[C@H]1CCCN([C@@H]1CN)S(=O)(=O)C(F)(F)Oc2cccc3C(=NNC(=O)c23)Cc4ccc(F)c(c4)C(=O)N5CCN(CC5)C(=O)C6CC6 | Olaparib 2188 |
| Fc1ccc(CC2=NNC(=O)c3c(cccc23)C(=O)NC(=S)\C(=C\C=C\c4ccccc4)\C#N)cc1C(=O)N5CCN(CC5)C(=O)C6CC6 | Olaparib 139 |
| C[C@@H](CO)CCNC(=O)c1cccc2C(=NNC(=O)c12)Cc3ccc(F)c(c3)C(=O)N4CCN(CC4)C(=O)C5CC5 | Olaparib 100 |
| C[C@H](CNC(=O)[C@H]1COc2ccccc12)OC(=O)c3cccc4C(=NNC(=O)c34)Cc5ccc(F)c(c5)C(=O)N6CCN(CC6)C(=O)C7CC7 | Olaparib 1025 |
| C[C@H]1CN(CC(=C)C(=O)Oc2cccc3C(=NNC(=O)c23)Cc4ccc(F)c(c4)C(=O)N5CCN(CC5)C(=O)C6CC6)C(=O)C1 | Olaparib 1474 |
| CC(C)[C@H]1C\C(=C/C(=O)Nc2cccc3C(=NNC(=O)c23)Cc4ccc(F)c(c4)C(=O)N5CCN(CC5)C(=O)C6CC6)\CCO1 | Olaparib 494 |
| C[C@H]1CCCN([C@@H]1CN)S(=O)(=O)[C@H](F)Oc2cccc3C(=NNC(=O)c23)Cc4ccc(F)c(c4)C(=O)N5CCN(CC5)C(=O)C6CC6 | Olaparib 2216 |
| Cc1cc2nc(COC(=O)c3cccc4C(=NNC(=O)c34)Cc5ccc(F)c(c5)C(=O)N6CCN(CC6)C(=O)C7CC7)[nH]c2cc1C | Olaparib 1091 |
| NC(=N)Sc1cc(Oc2cccc3C(=NNC(=O)c23)Cc4ccc(F)c(c4)C(=O)N5CCN(CC5)C(=O)C6CC6)ccc1Cl | Olaparib 2257 |
| COc1ccc(cc1)C(=S)NC(=O)c2cccc3C(=NNC(=O)c23)Cc4ccc(F)c(c4)C(=O)N5CCN(CC5)C(=O)C6CC6 | Olaparib 169 |
| COCC(C)(C)C(=O)Oc1cccc2C(=NNC(=O)c12)Cc3ccc(F)c(c3)C(=O)N4CCN(CC4)C(=O)C5CC5 | Olaparib 1271 |
| CSc1ccc(cc1)N2C[C@@H](CC2=O)OC(=O)c3cccc4C(=NNC(=O)c34)Cc5ccc(F)c(c5)C(=O)N6CCN(CC6)C(=O)C7CC7 | Olaparib 1020 |
| CCC1=C(O)C(=O)C=CN1CCOc2cccc3C(=NNC(=O)c23)Cc4ccc(F)c(c4)C(=O)N5CCN(CC5)C(=O)C6CC6 | Olaparib 2892 |
| CN1CCC(=CCCNC(=O)c2cccc3C(=NNC(=O)c23)Cc4ccc(F)c(c4)C(=O)N5CCN(CC5)C(=O)C6CC6)CC1 | Olaparib 182 |
| Fc1ccc(CC2=NNC(=O)c3c(OC(F)(F)CCNC(=O)C(=O)N4CCCNCC4)cccc23)cc1C(=O)N5CCN(CC5)C(=O)C6CC6 | Olaparib 2184 |
| Cn1nccc1[C@@H](O)c2cc(Oc3cccc4C(=NNC(=O)c34)Cc5ccc(F)c(c5)C(=O)N6CCN(CC6)C(=O)C7CC7)ccc2F | Olaparib 2555 |
| NC(=S)Nc1cccc(Oc2cccc3C(=NNC(=O)c23)Cc4ccc(F)c(c4)C(=O)N5CCN(CC5)C(=O)C6CC6)c1 | Olaparib 2921 |
| NC(=S)\C(=C\c1ccc(Oc2cccc3C(=NNC(=O)c23)Cc4ccc(F)c(c4)C(=O)N5CCN(CC5)C(=O)C6CC6)cc1Cl)\C#N | Olaparib 2190 |
| CC(C)(N)C(=O)Nc1cccc2C(=NNC(=O)c12)Cc3ccc(F)c(c3)C(=O)N4CCN(CC4)C(=O)C5CC5 | Olaparib 443 |
| Fc1ccc(CC2=NNC(=O)c3c(cccc23)C(=O)OCCN4C(=O)Sc5ccccc45)cc1C(=O)N6CCN(CC6)C(=O)C7CC7 | Olaparib 1059 |
| COC1=C(C(=O)N(C)N=C1)c2cccc3C(=NNC(=O)c23)Cc4ccc(F)c(c4)C(=O)N5CCN(CC5)C(=O)C6CC6 | Olaparib 1708 |
| Fc1ccc(CC2=NNC(=O)c3c(cccc23)C(=O)NCC\C=C/4\CCSC4)cc1C(=O)N5CCN(CC5)C(=O)C6CC6 | Olaparib 190 |
| CC(C)(NC(=O)c1cccc2C(=NNC(=O)c12)Cc3ccc(F)c(c3)C(=O)N4CCN(CC4)C(=O)C5CC5)c6cccc(CC(=O)O)c6O | Olaparib 143 |
| N[C@H](Cc1c[nH]c2cc(Cl)ccc12)C(=O)Oc3cccc4C(=NNC(=O)c34)Cc5ccc(F)c(c5)C(=O)N6CCN(CC6)C(=O)C7CC7 | Olaparib 1530 |
| CC[C@](C)(NC(=O)c1cccc2C(=NNC(=O)c12)Cc3ccc(F)c(c3)C(=O)N4CCN(CC4)C(=O)C5CC5)c6nccs6 | Olaparib 180 |
| CS(=O)(=O)c1ccc(Oc2cccc3C(=NNC(=O)c23)Cc4ccc(F)c(c4)C(=O)N5CCN(CC5)C(=O)C6CC6)cc1 | Olaparib 2915 |
| CC(C)OCC(=O)Nc1cccc2C(=NNC(=O)c12)Cc3ccc(F)c(c3)C(=O)N4CCN(CC4)C(=O)C5CC5 | Olaparib 394 |
| CN(CC(=O)Nc1ccc(Oc2cccc3C(=NNC(=O)c23)Cc4ccc(F)c(c4)C(=O)N5CCN(CC5)C(=O)C6CC6)cc1F)[C@H]7CCNC7 | Olaparib 2456 |
| OC1=C(Oc2cccc3C(=NNC(=O)c23)Cc4ccc(F)c(c4)C(=O)N5CCN(CC5)C(=O)C6CC6)C(=O)C1=O | Olaparib 3041 |
| CC1(C)OC(=O)C=C(O1)C(F)(F)c2cccc3C(=NNC(=O)c23)Cc4ccc(F)c(c4)C(=O)N5CCN(CC5)C(=O)C6CC6 | Olaparib 1689 |
| C[C@H](NC(=O)Nc1cc(C)cc(C)c1)C(=O)Oc2cccc3C(=NNC(=O)c23)Cc4ccc(F)c(c4)C(=O)N5CCN(CC5)C(=O)C6CC6 | Olaparib 1416 |
| Cn1c(Oc2cccc3C(=NNC(=O)c23)Cc4ccc(F)c(c4)C(=O)N5CCN(CC5)C(=O)C6CC6)nnc1c7cccc(c7)C(F)(F)F | Olaparib 3059 |
| N[C@H](C(=O)Nc1cccc2C(=NNC(=O)c12)Cc3ccc(F)c(c3)C(=O)N4CCN(CC4)C(=O)C5CC5)c6ccsc6 | Olaparib 509 |
| NC(=N)Sc1ccc(Oc2cccc3C(=NNC(=O)c23)Cc4ccc(F)c(c4)C(=O)N5CCN(CC5)C(=O)C6CC6)c(Cl)c1 | Olaparib 2297 |
| CSCCCNC(=O)c1cccc2C(=NNC(=O)c12)Cc3ccc(F)c(c3)C(=O)N4CCN(CC4)C(=O)C5CC5 | Olaparib 89 |
| CC(=O)N1CCC(CC1)c2cc(Oc3cccc4C(=NNC(=O)c34)Cc5ccc(F)c(c5)C(=O)N6CCN(CC6)C(=O)C7CC7)nc(C)n2 | Olaparib 2497 |
| CC(C)(C)CC(=O)N1CCC(C)(CC1)C(=O)Nc2cccc3C(=NNC(=O)c23)Cc4ccc(F)c(c4)C(=O)N5CCN(CC5)C(=O)C6CC6 | Olaparib 457 |
| CC(C)[C@H]1C[C@@H](CC(=O)Nc2cccc3C(=NNC(=O)c23)Cc4ccc(F)c(c4)C(=O)N5CCN(CC5)C(=O)C6CC6)CCO1 | Olaparib 492 |
| C[C@@H](NC(=O)Nc1ccccc1C)C(=O)Oc2cccc3C(=NNC(=O)c23)Cc4ccc(F)c(c4)C(=O)N5CCN(CC5)C(=O)C6CC6 | Olaparib 1426 |
| Nc1nn(cc1Oc2cccc3C(=NNC(=O)c23)Cc4ccc(F)c(c4)C(=O)N5CCN(CC5)C(=O)C6CC6)c7ccc(cc7)C(F)(F)F | Olaparib 2478 |
| COc1ccsc1COC(=O)c2cccc3C(=NNC(=O)c23)Cc4ccc(F)c(c4)C(=O)N5CCN(CC5)C(=O)C6CC6 | Olaparib 903 |
| NC(=S)Nc1ccc(Oc2cccc3C(=NNC(=O)c23)Cc4ccc(F)c(c4)C(=O)N5CCN(CC5)C(=O)C6CC6)cc1 | Olaparib 2922 |
| CCSCCNC(=O)c1cccc2C(=NNC(=O)c12)Cc3ccc(F)c(c3)C(=O)N4CCN(CC4)C(=O)C5CC5 | Olaparib 88 |
| COc1cc(cc(Oc2cccc3C(=NNC(=O)c23)Cc4ccc(F)c(c4)C(=O)N5CCN(CC5)C(=O)C6CC6)c1O)[C@@H]7CCC(=O)N7 | Olaparib 2554 |
| Fc1ccc(CC2=NNC(=O)c3c(NC(=O)CON=C4CCCCCCC4)cccc23)cc1C(=O)N5CCN(CC5)C(=O)C6CC6 | Olaparib 483 |
| CC1(C)OC[C@@H](O1)\C=C/COc2cccc3C(=NNC(=O)c23)Cc4ccc(F)c(c4)C(=O)N5CCN(CC5)C(=O)C6CC6 | Olaparib 2954 |
| OC(=O)CC(=O)Nc1ccc(Oc2cccc3C(=NNC(=O)c23)Cc4ccc(F)c(c4)C(=O)N5CCN(CC5)C(=O)C6CC6)c(Cl)c1 | Olaparib 2209 |
| Fc1ccc(CC2=NNC(=O)c3c(Oc4cccc(c4)c5n[nH]c(Cl)n5)cccc23)cc1C(=O)N6CCN(CC6)C(=O)C7CC7 | Olaparib 2576 |
| CC(C)c1nnc(Oc2cccc3C(=NNC(=O)c23)Cc4ccc(F)c(c4)C(=O)N5CCN(CC5)C(=O)C6CC6)s1 | Olaparib 2990 |
| N[C@@H](C(=O)Oc1cccc2C(=NNC(=O)c12)Cc3ccc(F)c(c3)C(=O)N4CCN(CC4)C(=O)C5CC5)c6ccsc6 | Olaparib 1498 |
| N[C@H](CC(=O)O)C(F)(F)c1cccc2C(=NNC(=O)c12)Cc3ccc(F)c(c3)C(=O)N4CCN(CC4)C(=O)C5CC5 | Olaparib 1621 |
| C[C@@H](N1C[C@@H](CC1=O)OC(=O)c2cccc3C(=NNC(=O)c23)Cc4ccc(F)c(c4)C(=O)N5CCN(CC5)C(=O)C6CC6)c7occc7 | Olaparib 1060 |
| C[C@@H]1CCN(C1)C(=O)CCC(=O)Oc2cccc3C(=NNC(=O)c23)Cc4ccc(F)c(c4)C(=O)N5CCN(CC5)C(=O)C6CC6 | Olaparib 1470 |
| NC1(CCCC1)C(=O)Oc2cccc3C(=NNC(=O)c23)Cc4ccc(F)c(c4)C(=O)N5CCN(CC5)C(=O)C6CC6 | Olaparib 1514 |
| OC(=O)CC(=O)Nc1ccc(Cl)c(Oc2cccc3C(=NNC(=O)c23)Cc4ccc(F)c(c4)C(=O)N5CCN(CC5)C(=O)C6CC6)c1 | Olaparib 2208 |
| Nc1[nH]c2ccc(Oc3cccc4C(=NNC(=O)c34)Cc5ccc(F)c(c5)C(=O)N6CCN(CC6)C(=O)C7CC7)cc2c1C#N | Olaparib 3127 |
| C[C@H](CN1CCCC(C)(C)C1)C(=S)NC(=O)c2cccc3C(=NNC(=O)c23)Cc4ccc(F)c(c4)C(=O)N5CCN(CC5)C(=O)C6CC6 | Olaparib 138 |
| N[C@@H](C(=O)Oc1cccc2C(=NNC(=O)c12)Cc3ccc(F)c(c3)C(=O)N4CCN(CC4)C(=O)C5CC5)c6cccs6 | Olaparib 1497 |
| N[C@@H](CCc1cccc2C(=NNC(=O)c12)Cc3ccc(F)c(c3)C(=O)N4CCN(CC4)C(=O)C5CC5)C(=O)O | Olaparib 1923 |
| C[C@]1(C[C@H](CCO1)OC(=O)c2cccc3C(=NNC(=O)c23)Cc4ccc(F)c(c4)C(=O)N5CCN(CC5)C(=O)C6CC6)c7cnccn7 | Olaparib 1062 |
| C[C@@H]1C[C@](CNC(=O)c2cccc3C(=NNC(=O)c23)Cc4ccc(F)c(c4)C(=O)N5CCN(CC5)C(=O)C6CC6)(C1)C(=O)O | Olaparib 191 |
| Fc1ccc(CC2=NNC(=O)c3c(NC(=O)C[C@@H]4CCCO4)cccc23)cc1C(=O)N5CCN(CC5)C(=O)C6CC6 | Olaparib 516 |
| Cc1nc(C)n(CC(=C)C(=O)Oc2cccc3C(=NNC(=O)c23)Cc4ccc(F)c(c4)C(=O)N5CCN(CC5)C(=O)C6CC6)n1 | Olaparib 1476 |
| CC1=CC=CN(CC(=O)c2ccc(Oc3cccc4C(=NNC(=O)c34)Cc5ccc(F)c(c5)C(=O)N6CCN(CC6)C(=O)C7CC7)cc2)C1=O | Olaparib 2521 |
| Fc1ccc(CC2=NNC(=O)c3c(OC4(Cl)CS(=O)(=O)C4)cccc23)cc1C(=O)N5CCN(CC5)C(=O)C6CC6 | Olaparib 2370 |
| C[C@H]1SC(=O)C(=C1OC(=O)c2cccc3C(=NNC(=O)c23)Cc4ccc(F)c(c4)C(=O)N5CCN(CC5)C(=O)C6CC6)C | Olaparib 904 |
| O[C@H](CC(=O)O)C(F)(F)c1cccc2C(=NNC(=O)c12)Cc3ccc(F)c(c3)C(=O)N4CCN(CC4)C(=O)C5CC5 | Olaparib 1619 |
| NC[C@]1(COC(=O)c2cccc3C(=NNC(=O)c23)Cc4ccc(F)c(c4)C(=O)N5CCN(CC5)C(=O)C6CC6)C[C@H]1c7ccncn7 | Olaparib 1085 |
| Fc1ccc(CC2=NNC(=O)c3c(NC(=O)CON=C4CCCC4)cccc23)cc1C(=O)N5CCN(CC5)C(=O)C6CC6 | Olaparib 510 |
| Fc1ccc(CC2=NNC(=O)c3c(OC(=O)CN4CCC4)cccc23)cc1C(=O)N5CCN(CC5)C(=O)C6CC6 | Olaparib 1519 |
| Fc1ccc(CC2=NNC(=O)c3c(OC(=O)c4cnc(C=O)s4)cccc23)cc1C(=O)N5CCN(CC5)C(=O)C6CC6 | Olaparib 1500 |
| Fc1ccc(CC2=NNC(=O)c3c(cccc23)C(=O)OCC4=CN(C(=O)C=C4)c5ccccc5)cc1C(=O)N6CCN(CC6)C(=O)C7CC7 | Olaparib 1055 |
| C[C@H]1CSCCN1C[C@H](CN)Oc2cccc3C(=NNC(=O)c23)Cc4ccc(F)c(c4)C(=O)N5CCN(CC5)C(=O)C6CC6 | Olaparib 2882 |
| CC[C@@H](CN1C[C@@H](C)S[C@@H](C)C1)OC(=O)c2cccc3C(=NNC(=O)c23)Cc4ccc(F)c(c4)C(=O)N5CCN(CC5)C(=O)C6CC6 | Olaparib 715 |
| OC[C@@H](C(=O)O)C(F)(F)c1cccc2C(=NNC(=O)c12)Cc3ccc(F)c(c3)C(=O)N4CCN(CC4)C(=O)C5CC5 | Olaparib 1620 |
| Fc1ccc(CC2=NNC(=O)c3c(cccc23)C(=O)OCCc4ccc5NCCNc5n4)cc1C(=O)N6CCN(CC6)C(=O)C7CC7 | Olaparib 1086 |
| C[C@H]1CC[C@H](CNC(=O)c2cccc3C(=NNC(=O)c23)Cc4ccc(F)c(c4)C(=O)N5CCN(CC5)C(=O)C6CC6)CN1CC(C)(C)O | Olaparib 149 |
| Fc1ccc(CC2=NNC(=O)c3c(OC[C@H]4CCC(=O)N4)cccc23)cc1C(=O)N5CCN(CC5)C(=O)C6CC6 | Olaparib 3036 |
| CC(C)(NC(=O)c1cccc2C(=NNC(=O)c12)Cc3ccc(F)c(c3)C(=O)N4CCN(CC4)C(=O)C5CC5)c6cn(CC(F)F)nn6 | Olaparib 154 |
| Fc1ccc(CC2=NNC(=O)c3c(OC(=O)\C=C\c4ccccc4[C@H]5CCC(=O)N5)cccc23)cc1C(=O)N6CCN(CC6)C(=O)C7CC7 | Olaparib 1540 |
| CC(C)(COC(=O)c1cccc2C(=NNC(=O)c12)Cc3ccc(F)c(c3)C(=O)N4CCN(CC4)C(=O)C5CC5)\N=C\c6ccccc6 | Olaparib 775 |
| C[C@H]1CSCCN1CC(C)(C)COC(=O)c2cccc3C(=NNC(=O)c23)Cc4ccc(F)c(c4)C(=O)N5CCN(CC5)C(=O)C6CC6 | Olaparib 714 |
| OC(=O)CCSC(F)(F)c1cccc2C(=NNC(=O)c12)Cc3ccc(F)c(c3)C(=O)N4CCN(CC4)C(=O)C5CC5 | Olaparib 1609 |
| C[C@@H](OC(=O)c1cccc2C(=NNC(=O)c12)Cc3ccc(F)c(c3)C(=O)N4CCN(CC4)C(=O)C5CC5)c6c(C)nn(C7CCOCC7)c6C | Olaparib 1017 |
| C[C@H](CC#N)N1CCC[C@@H](C1)[C@@H](C)NC(=O)c2cccc3C(=NNC(=O)c23)Cc4ccc(F)c(c4)C(=O)N5CCN(CC5)C(=O)C6CC6 | Olaparib 152 |
| CN[C@@]1(COc2cccc3C(=NNC(=O)c23)Cc4ccc(F)c(c4)C(=O)N5CCN(CC5)C(=O)C6CC6)CCOC(C)(C)C1 | Olaparib 2910 |
| N[C@@H](Cn1cccn1)C(=O)Oc2cccc3C(=NNC(=O)c23)Cc4ccc(F)c(c4)C(=O)N5CCN(CC5)C(=O)C6CC6 | Olaparib 1503 |
| Fc1ccc(CC2=NNC(=O)c3c(Oc4cncc(c4)N5C=CC=CC5=O)cccc23)cc1C(=O)N6CCN(CC6)C(=O)C7CC7 | Olaparib 3112 |
| Fc1ccc(CC2=NNC(=O)c3c(Oc4c(F)c(C=O)c(F)c(F)c4C=O)cccc23)cc1C(=O)N5CCN(CC5)C(=O)C6CC6 | Olaparib 2292 |
| C[C@@H]1CN(CCCOC(=O)c2cccc3C(=NNC(=O)c23)Cc4ccc(F)c(c4)C(=O)N5CCN(CC5)C(=O)C6CC6)C[C@H](C)S1 | Olaparib 738 |
| C[N+](C)(C)C[C@@H](CO)Oc1cccc2C(=NNC(=O)c12)Cc3ccc(F)c(c3)C(=O)N4CCN(CC4)C(=O)C5CC5 | Olaparib 2759 |
| Cc1c(C)c(nnc1Oc2cccc3C(=NNC(=O)c23)Cc4ccc(F)c(c4)C(=O)N5CCN(CC5)C(=O)C6CC6)N(CCC#N)C7CC7 | Olaparib 2506 |
| C[C@H]1CC[C@H](CNC(=O)c2cccc3C(=NNC(=O)c23)Cc4ccc(F)c(c4)C(=O)N5CCN(CC5)C(=O)C6CC6)CN1CCO | Olaparib 162 |
| Fc1ccc(CC2=NNC(=O)c3c(OC[C@H]4CNCCO4)cccc23)cc1C(=O)N5CCN(CC5)C(=O)C6CC6 | Olaparib 3033 |
| Cc1oc(CCC(=O)Oc2cccc3C(=NNC(=O)c23)Cc4ccc(F)c(c4)C(=O)N5CCN(CC5)C(=O)C6CC6)nn1 | Olaparib 1502 |
| C[C@@H](OC(=O)c1cccc2C(=NNC(=O)c12)Cc3ccc(F)c(c3)C(=O)N4CCN(CC4)C(=O)C5CC5)C(=O)N6CCC(Cc7ccccc7)CC6 | Olaparib 991 |
| Fc1ccc(CC2=NNC(=O)c3c(Oc4ccc(C=O)cc4C=O)cccc23)cc1C(=O)N5CCN(CC5)C(=O)C6CC6 | Olaparib 2978 |
| Fc1ccc(CC2=NNC(=O)c3c(OC4CNC(=S)NC4)cccc23)cc1C(=O)N5CCN(CC5)C(=O)C6CC6 | Olaparib 3011 |
| C[C@](N)(CO)COc1cccc2C(=NNC(=O)c12)Cc3ccc(F)c(c3)C(=O)N4CCN(CC4)C(=O)C5CC5 | Olaparib 2835 |
| CN1CCC[C@H](C1)N2C[C@H](COC(=O)c3cccc4C(=NNC(=O)c34)Cc5ccc(F)c(c5)C(=O)N6CCN(CC6)C(=O)C7CC7)CC2=O | Olaparib 1035 |
| C[C@H]1CC[C@H](CN)CN1CC(=O)Nc2cccc3C(=NNC(=O)c23)Cc4ccc(F)c(c4)C(=O)N5CCN(CC5)C(=O)C6CC6 | Olaparib 491 |
| CCc1ccc(\C=C\CNC(=O)c2cccc3C(=NNC(=O)c23)Cc4ccc(F)c(c4)C(=O)N5CCN(CC5)C(=O)C6CC6)cc1 | Olaparib 174 |
| Fc1ccc(CC2=NNC(=O)c3c(OC(=O)\C=C\CN4N=CC=CC4=O)cccc23)cc1C(=O)N5CCN(CC5)C(=O)C6CC6 | Olaparib 1479 |
| Fc1ccc([C@@H]2CCC(=O)N2)c(OC(=O)c3cccc4C(=NNC(=O)c34)Cc5ccc(F)c(c5)C(=O)N6CCN(CC6)C(=O)C7CC7)c1F | Olaparib 1033 |
| CCC(C)(C)N(C)C(=O)c1cc(Oc2cccc3C(=NNC(=O)c23)Cc4ccc(F)c(c4)C(=O)N5CCN(CC5)C(=O)C6CC6)ccc1F | Olaparib 2189 |
| Cc1ccc(cc1)C(=O)\C=C\C(=O)Nc2cccc3C(=NNC(=O)c23)Cc4ccc(F)c(c4)C(=O)N5CCN(CC5)C(=O)C6CC6 | Olaparib 488 |
| CNC[C@@H](CO)Oc1cccc2C(=NNC(=O)c12)Cc3ccc(F)c(c3)C(=O)N4CCN(CC4)C(=O)C5CC5 | Olaparib 2831 |
| Fc1ccc(CC2=NNC(=O)c3c(cccc23)C(=O)OC[C@H]4CCCN(C4)C(=O)[C@H]5CCSC5)cc1C(=O)N6CCN(CC6)C(=O)C7CC7 | Olaparib 1009 |
| COC[C@H](NC(=O)c1cccc2C(=NNC(=O)c12)Cc3ccc(F)c(c3)C(=O)N4CCN(CC4)C(=O)C5CC5)C6CCOCC6 | Olaparib 176 |
| CC(C)(NC(=O)c1cccc2C(=NNC(=O)c12)Cc3ccc(F)c(c3)C(=O)N4CCN(CC4)C(=O)C5CC5)c6ccc(cc6F)C(F)(F)F | Olaparib 133 |
| CC(=O)CN1N=CC(=C(Oc2cccc3C(=NNC(=O)c23)Cc4ccc(F)c(c4)C(=O)N5CCN(CC5)C(=O)C6CC6)C1=O)Cl | Olaparib 2258 |
| NC(=O)C1=Cc2cc(Oc3cccc4C(=NNC(=O)c34)Cc5ccc(F)c(c5)C(=O)N6CCN(CC6)C(=O)C7CC7)ccc2OC1 | Olaparib 2588 |
| Cc1cc(ccc1N)C(C)(C)NC(=O)c2cccc3C(=NNC(=O)c23)Cc4ccc(F)c(c4)C(=O)N5CCN(CC5)C(=O)C6CC6 | Olaparib 172 |
| CC(=O)\C=C\c1ccc(OC(=O)c2cccc3C(=NNC(=O)c23)Cc4ccc(F)c(c4)C(=O)N5CCN(CC5)C(=O)C6CC6)cc1 | Olaparib 828 |
| COC(=O)C(=C)c1cccc2C(=NNC(=O)c12)Cc3ccc(F)c(c3)C(=O)N4CCN(CC4)C(=O)C5CC5 | Olaparib 1933 |
| Fc1ccc(CC2=NNC(=O)c3c(cccc23)C(=O)OC[C@H]4CN(C5CCOCC5)C(=O)C4)cc1C(=O)N6CCN(CC6)C(=O)C7CC7 | Olaparib 1057 |
| CC1(C)C[C@@](O)(CCO1)C(=O)Oc2cccc3C(=NNC(=O)c23)Cc4ccc(F)c(c4)C(=O)N5CCN(CC5)C(=O)C6CC6 | Olaparib 1481 |
| CN(C)C(=O)c1cccc(c1)c2cccc3C(=NNC(=O)c23)Cc4ccc(F)c(c4)C(=O)N5CCN(CC5)C(=O)C6CC6 | Olaparib 1967 |
| CC(=O)N1N=CC(=C(Oc2cccc3C(=NNC(=O)c23)Cc4ccc(F)c(c4)C(=O)N5CCN(CC5)C(=O)C6CC6)C1=O)Cl | Olaparib 2288 |
| C[C@H](NC(=O)c1cccc2C(=NNC(=O)c12)Cc3ccc(F)c(c3)C(=O)N4CCN(CC4)C(=O)C5CC5)[C@H]6CCCN(Cc7cc(C)ccc7O)C6 | Olaparib 202 |
| Cc1ccc(cc1O)C(C)(C)C(=O)Nc2cccc3C(=NNC(=O)c23)Cc4ccc(F)c(c4)C(=O)N5CCN(CC5)C(=O)C6CC6 | Olaparib 485 |
| CN(C)c1ccc(\C=C\COC(=O)c2cccc3C(=NNC(=O)c23)Cc4ccc(F)c(c4)C(=O)N5CCN(CC5)C(=O)C6CC6)cc1 | Olaparib 776 |
| COC(=O)C(=C(F)F)c1cccc2C(=NNC(=O)c12)Cc3ccc(F)c(c3)C(=O)N4CCN(CC4)C(=O)C5CC5 | Olaparib 1633 |
| Fc1ccc(CC2=NNC(=O)c3c(cccc23)C(=O)O[C@H]4CN(C[C@H]5CCCO5)C(=O)C4)cc1C(=O)N6CCN(CC6)C(=O)C7CC7 | Olaparib 1080 |
| CC(C)CC1=NO[C@](O)(C1)C(F)(F)Oc2cccc3C(=NNC(=O)c23)Cc4ccc(F)c(c4)C(=O)N5CCN(CC5)C(=O)C6CC6 | Olaparib 2282 |
| COCC(=O)c1ccc(cc1)C(F)(F)c2cccc3C(=NNC(=O)c23)Cc4ccc(F)c(c4)C(=O)N5CCN(CC5)C(=O)C6CC6 | Olaparib 1669 |
| OC(=O)C1=CC(=CNC1=O)Oc2cccc3C(=NNC(=O)c23)Cc4ccc(F)c(c4)C(=O)N5CCN(CC5)C(=O)C6CC6 | Olaparib 2375 |
| CC[C@]1(CCCNC1)C(=O)Nc2cccc(F)c2Oc3cccc4C(=NNC(=O)c34)Cc5ccc(F)c(c5)C(=O)N6CCN(CC6)C(=O)C7CC7 | Olaparib 2462 |
| Cc1ccccc1OC(C)(C)C(=O)Oc2cccc3C(=NNC(=O)c23)Cc4ccc(F)c(c4)C(=O)N5CCN(CC5)C(=O)C6CC6 | Olaparib 1458 |
| CC(=O)CCc1ccc(OC(=O)c2cccc3C(=NNC(=O)c23)Cc4ccc(F)c(c4)C(=O)N5CCN(CC5)C(=O)C6CC6)cc1 | Olaparib 822 |
| FC(=C(F)C(=O)Nc1cccc2C(=NNC(=O)c12)Cc3ccc(F)c(c3)C(=O)N4CCN(CC4)C(=O)C5CC5)F | Olaparib 379 |
| O[C@H]1[C@H](O)[C@@H](Oc2cccc3C(=NNC(=O)c23)Cc4ccc(F)c(c4)C(=O)N5CCN(CC5)C(=O)C6CC6)[C@H]7OC[C@@H]1O7 | Olaparib 3134 |
| Fc1ccc(CC2=NNC(=O)c3c(OCCCC(=O)N4CCOCC4)cccc23)cc1C(=O)N5CCN(CC5)C(=O)C6CC6 | Olaparib 2911 |
| Fc1ccc(CC2=NNC(=O)c3c(CCCOC(=O)c4ccccc4)cccc23)cc1C(=O)N5CCN(CC5)C(=O)C6CC6 | Olaparib 1832 |
| CC[C@@H](N1C(=O)CSC=C1C)C(=O)Oc2cccc3C(=NNC(=O)c23)Cc4ccc(F)c(c4)C(=O)N5CCN(CC5)C(=O)C6CC6 | Olaparib 1431 |
| CC[C@@]1(CCCNC1)C(=O)Nc2cc(Oc3cccc4C(=NNC(=O)c34)Cc5ccc(F)c(c5)C(=O)N6CCN(CC6)C(=O)C7CC7)ccc2F | Olaparib 2463 |
| CN[C@H](C)[C@@H](OC(=O)c1cccc2C(=NNC(=O)c12)Cc3ccc(F)c(c3)C(=O)N4CCN(CC4)C(=O)C5CC5)c6ccccc6 | Olaparib 816 |
| COc1ccc(\C=C\COC(=O)c2cccc3C(=NNC(=O)c23)Cc4ccc(F)c(c4)C(=O)N5CCN(CC5)C(=O)C6CC6)cc1 | Olaparib 823 |
| CC(C)C(C)(C)C(=O)Nc1cccc2C(=NNC(=O)c12)Cc3ccc(F)c(c3)C(=O)N4CCN(CC4)C(=O)C5CC5 | Olaparib 355 |
| O[C@@H]1[C@@H](O)[C@H](Oc2cccc3C(=NNC(=O)c23)Cc4ccc(F)c(c4)C(=O)N5CCN(CC5)C(=O)C6CC6)[C@@H]7CO[C@H]1O7 | Olaparib 3133 |
| N[C@]1(CCOC1)C(=O)Nc2cccc3C(=NNC(=O)c23)Cc4ccc(F)c(c4)C(=O)N5CCN(CC5)C(=O)C6CC6 | Olaparib 515 |
| NC(=N)c1cccc(c1)C(F)(F)c2cccc3C(=NNC(=O)c23)Cc4ccc(F)c(c4)C(=O)N5CCN(CC5)C(=O)C6CC6 | Olaparib 1697 |
| Fc1ccc(CC2=NNC(=O)c3c(cccc23)C(=O)NC(=S)NN=C4CCCC4)cc1C(=O)N5CCN(CC5)C(=O)C6CC6 | Olaparib 178 |
| C[C@H](OC(=O)c1cccc2C(=NNC(=O)c12)Cc3ccc(F)c(c3)C(=O)N4CCN(CC4)C(=O)C5CC5)c6ccc(OC7CCOCC7)cc6 | Olaparib 1021 |
| C\C(=C\CN)\c1ccc(O[C@@H](F)Oc2cccc3C(=NNC(=O)c23)Cc4ccc(F)c(c4)C(=O)N5CCN(CC5)C(=O)C6CC6)cc1 | Olaparib 2273 |
| CC(=O)c1ccc(COC(=O)c2cccc3C(=NNC(=O)c23)Cc4ccc(F)c(c4)C(=O)N5CCN(CC5)C(=O)C6CC6)cc1 | Olaparib 882 |
| CCC(C)(C)CC(=O)Nc1cccc2C(=NNC(=O)c12)Cc3ccc(F)c(c3)C(=O)N4CCN(CC4)C(=O)C5CC5 | Olaparib 358 |
| Cc1ccc(cc1)C(=O)Nc2ccc(OC(=O)c3cccc4C(=NNC(=O)c34)Cc5ccc(F)c(c5)C(=O)N6CCN(CC6)C(=O)C7CC7)cc2 | Olaparib 1011 |
| N[C@]1(CCOC1)C(=O)Oc2cccc3C(=NNC(=O)c23)Cc4ccc(F)c(c4)C(=O)N5CCN(CC5)C(=O)C6CC6 | Olaparib 1512 |
| OCC(=O)c1cccc(c1)C(F)(F)c2cccc3C(=NNC(=O)c23)Cc4ccc(F)c(c4)C(=O)N5CCN(CC5)C(=O)C6CC6 | Olaparib 1681 |
| CN1C=C(Oc2cccc3C(=NNC(=O)c23)Cc4ccc(F)c(c4)C(=O)N5CCN(CC5)C(=O)C6CC6)C(=O)N(C)C1=O | Olaparib 2958 |
| CCOc1ccc(cc1)[C@@]2(CCCOC2)OC(=O)c3cccc4C(=NNC(=O)c34)Cc5ccc(F)c(c5)C(=O)N6CCN(CC6)C(=O)C7CC7 | Olaparib 1022 |
| C[C@@](N)(Cc1ccccc1)C(=O)Oc2cccc3C(=NNC(=O)c23)Cc4ccc(F)c(c4)C(=O)N5CCN(CC5)C(=O)C6CC6 | Olaparib 1480 |
| CC(=O)c1ccc(cc1)C#CCOC(=O)c2cccc3C(=NNC(=O)c23)Cc4ccc(F)c(c4)C(=O)N5CCN(CC5)C(=O)C6CC6 | Olaparib 779 |
| CC[C@@](C)(Br)C(=O)Oc1cccc2C(=NNC(=O)c12)Cc3ccc(F)c(c3)C(=O)N4CCN(CC4)C(=O)C5CC5 | Olaparib 1181 |
| Cc1cccc(NC(=O)c2ccc(Oc3cccc4C(=NNC(=O)c34)Cc5ccc(F)c(c5)C(=O)N6CCN(CC6)C(=O)C7CC7)nc2)c1 | Olaparib 2546 |
| Fc1ccc(CC2=NNC(=O)c3c(OC(Cl)(Cl)C(=N)OCc4ccccc4)cccc23)cc1C(=O)N5CCN(CC5)C(=O)C6CC6 | Olaparib 2199 |
| OC(=O)Cc1ccc(F)c(c1)c2cccc3C(=NNC(=O)c23)Cc4ccc(F)c(c4)C(=O)N5CCN(CC5)C(=O)C6CC6 | Olaparib 1709 |
| Fc1ccc(CC2=NNC(=O)c3c(OC4=CC(=O)NC(=O)N4)cccc23)cc1C(=O)N5CCN(CC5)C(=O)C6CC6 | Olaparib 2221 |
| Cc1ccc(cc1Oc2cccc3C(=NNC(=O)c23)Cc4ccc(F)c(c4)C(=O)N5CCN(CC5)C(=O)C6CC6)C(=O)N[C@H]7CCCOC7 | Olaparib 3066 |
| COc1cccc(c1)[C@H](C)NC(=O)c2cccc3C(=NNC(=O)c23)Cc4ccc(F)c(c4)C(=O)N5CCN(CC5)C(=O)C6CC6 | Olaparib 185 |
| Oc1ccc(\C=C\COC(=O)c2cccc3C(=NNC(=O)c23)Cc4ccc(F)c(c4)C(=O)N5CCN(CC5)C(=O)C6CC6)cc1 | Olaparib 881 |
| CCC[C@@H](F)C(=O)Oc1cccc2C(=NNC(=O)c12)Cc3ccc(F)c(c3)C(=O)N4CCN(CC4)C(=O)C5CC5 | Olaparib 1319 |
| OS(=O)(=O)OCCNC(=O)c1cccc2C(=NNC(=O)c12)Cc3ccc(F)c(c3)C(=O)N4CCN(CC4)C(=O)C5CC5 | Olaparib 9 |
| Cc1ccc(NC(=O)c2ccc(Oc3cccc4C(=NNC(=O)c34)Cc5ccc(F)c(c5)C(=O)N6CCN(CC6)C(=O)C7CC7)nc2)c(Cl)c1 | Olaparib 2471 |
| COc1cccc2c(OC(=O)c3cccc4C(=NNC(=O)c34)Cc5ccc(F)c(c5)C(=O)N6CCN(CC6)C(=O)C7CC7)c(Cl)c(C)nc12 | Olaparib 1018 |
| Fc1ccc(CC2=NNC(=O)c3c(OC4=NSN=C(Cl)C4=O)cccc23)cc1C(=O)N5CCN(CC5)C(=O)C6CC6 | Olaparib 2350 |
| Fc1ccc(CC2=NNC(=O)c3c(Oc4ccc(cc4)C(=O)N[C@@H]5CCCCNC5)cccc23)cc1C(=O)N6CCN(CC6)C(=O)C7CC7 | Olaparib 2539 |
| Fc1ccc(CC(=O)Oc2cccc3C(=NNC(=O)c23)Cc4ccc(F)c(c4)C(=O)N5CCN(CC5)C(=O)C6CC6)c(Br)c1 | Olaparib 1417 |
| C[C@H](OC(=O)c1cccc2C(=NNC(=O)c12)Cc3ccc(F)c(c3)C(=O)N4CCN(CC4)C(=O)C5CC5)c6ccccc6CO | Olaparib 857 |
| OC\C=C\c1ccc(OC(=O)c2cccc3C(=NNC(=O)c23)Cc4ccc(F)c(c4)C(=O)N5CCN(CC5)C(=O)C6CC6)cc1 | Olaparib 880 |
| CC(F)(F)CCC(=O)Oc1cccc2C(=NNC(=O)c12)Cc3ccc(F)c(c3)C(=O)N4CCN(CC4)C(=O)C5CC5 | Olaparib 1253 |
| CC(C)(NC(=O)NC(=O)\C=C\C(=O)Nc1cccc2C(=NNC(=O)c12)Cc3ccc(F)c(c3)C(=O)N4CCN(CC4)C(=O)C5CC5)C#C | Olaparib 272 |
| C[C@H](OC(=O)c1cccc2C(=NNC(=O)c12)Cc3ccc(F)c(c3)C(=O)N4CCN(CC4)C(=O)C5CC5)c6ccc(cc6)n7nc(C)c(C)c7C | Olaparib 1008 |
| O[C@@H](Cc1cccc(Oc2cccc3C(=NNC(=O)c23)Cc4ccc(F)c(c4)C(=O)N5CCN(CC5)C(=O)C6CC6)c1)c7ccncn7 | Olaparib 2540 |
| CC(C)N(C)C(=O)N1CCCC[C@H]1CC(=O)Oc2cccc3C(=NNC(=O)c23)Cc4ccc(F)c(c4)C(=O)N5CCN(CC5)C(=O)C6CC6 | Olaparib 1407 |
| COc1ccc(cc1)[C@@]2(CCCOC2)OC(=O)c3cccc4C(=NNC(=O)c34)Cc5ccc(F)c(c5)C(=O)N6CCN(CC6)C(=O)C7CC7 | Olaparib 1044 |
| O\N=C(/c1cccc2C(=NNC(=O)c12)Cc3ccc(F)c(c3)C(=O)N4CCN(CC4)C(=O)C5CC5)\c6c(Cl)cccc6Cl | Olaparib 1667 |
| CC[C@@H](OC(=O)c1cccc2C(=NNC(=O)c12)Cc3ccc(F)c(c3)C(=O)N4CCN(CC4)C(=O)C5CC5)c6ccc(O)cc6 | Olaparib 862 |
| Fc1ccc(CC2=NNC(=O)c3c(cccc23)C(=O)OCc4cc(Cl)c(C=O)c(Cl)c4)cc1C(=O)N5CCN(CC5)C(=O)C6CC6 | Olaparib 713 |
| CC(C)(Br)C(=O)Oc1cccc2C(=NNC(=O)c12)Cc3ccc(F)c(c3)C(=O)N4CCN(CC4)C(=O)C5CC5 | Olaparib 1192 |
| OC(=O)c1ccc([nH]1)c2ccc(Oc3cccc4C(=NNC(=O)c34)Cc5ccc(F)c(c5)C(=O)N6CCN(CC6)C(=O)C7CC7)cc2 | Olaparib 2563 |
| COC(=O)N[C@@H](O)C(=O)Nc1cccc2C(=NNC(=O)c12)Cc3ccc(F)c(c3)C(=O)N4CCN(CC4)C(=O)C5CC5 | Olaparib 292 |
| C[C@H](CN1C[C@@H](C)S[C@@H](C)C1)C(=N)NC(=O)c2cccc3C(=NNC(=O)c23)Cc4ccc(F)c(c4)C(=O)N5CCN(CC5)C(=O)C6CC6 | Olaparib 137 |
| CC(=O)c1cc(Oc2cccc3C(=NNC(=O)c23)Cc4ccc(F)c(c4)C(=O)N5CCN(CC5)C(=O)C6CC6)ccc1OC7CCOCC7 | Olaparib 2494 |
| Oc1ccc(C=O)cc1c2cccc3C(=NNC(=O)c23)Cc4ccc(F)c(c4)C(=O)N5CCN(CC5)C(=O)C6CC6 | Olaparib 1687 |
| Cc1cc(ccc1F)[C@@H](O)C(=O)Nc2cccc3C(=NNC(=O)c23)Cc4ccc(F)c(c4)C(=O)N5CCN(CC5)C(=O)C6CC6 | Olaparib 495 |
| Fc1ccc(CC2=NNC(=O)c3c(cccc23)C(=O)OCc4ccccc4C=O)cc1C(=O)N5CCN(CC5)C(=O)C6CC6 | Olaparib 935 |
| CC(=O)OCCc1cccc2C(=NNC(=O)c12)Cc3ccc(F)c(c3)C(=O)N4CCN(CC4)C(=O)C5CC5 | Olaparib 1805 |
| Fc1ccc(F)c(c1)c2c[nH]c(COC(=O)c3cccc4C(=NNC(=O)c34)Cc5ccc(F)c(c5)C(=O)N6CCN(CC6)C(=O)C7CC7)n2 | Olaparib 1042 |
| CS(=O)(=O)CC(=O)Nc1cccc2C(=NNC(=O)c12)Cc3ccc(F)c(c3)C(=O)N4CCN(CC4)C(=O)C5CC5 | Olaparib 328 |
| CN(C)C(=O)N1CCC[C@H](CC(=O)Nc2cccc3C(=NNC(=O)c23)Cc4ccc(F)c(c4)C(=O)N5CCN(CC5)C(=O)C6CC6)C1 | Olaparib 470 |
| Fc1ccc(CC2=NNC(=O)c3c(OC(=O)C[C@H]4CCCN(C4)c5ccncc5)cccc23)cc1C(=O)N6CCN(CC6)C(=O)C7CC7 | Olaparib 1548 |
| O\N=C(\c1cccc(Cl)c1)/c2cccc3C(=NNC(=O)c23)Cc4ccc(F)c(c4)C(=O)N5CCN(CC5)C(=O)C6CC6 | Olaparib 1695 |
| N[C@@H](C(=O)Nc1cccc2C(=NNC(=O)c12)Cc3ccc(F)c(c3)C(=O)N4CCN(CC4)C(=O)C5CC5)c6ccc(C(F)F)c(F)c6 | Olaparib 467 |
| Fc1ccc(CC2=NNC(=O)c3c(cccc23)C(=O)NC(=S)c4ccc(Br)cc4)cc1C(=O)N5CCN(CC5)C(=O)C6CC6 | Olaparib 136 |
| CC[C@@H](F)C(=O)Oc1cccc2C(=NNC(=O)c12)Cc3ccc(F)c(c3)C(=O)N4CCN(CC4)C(=O)C5CC5 | Olaparib 1378 |
| Fc1ccc(cc1Cl)c2c[nH]c(COC(=O)c3cccc4C(=NNC(=O)c34)Cc5ccc(F)c(c5)C(=O)N6CCN(CC6)C(=O)C7CC7)n2 | Olaparib 1013 |
| CC(=O)[C@@H](C#N)C(=S)NC(=O)c1cccc2C(=NNC(=O)c12)Cc3ccc(F)c(c3)C(=O)N4CCN(CC4)C(=O)C5CC5 | Olaparib 8 |
| C[C@H](N(C)C(=O)[C@H]1CCSC1)C(=O)Oc2cccc3C(=NNC(=O)c23)Cc4ccc(F)c(c4)C(=O)N5CCN(CC5)C(=O)C6CC6 | Olaparib 1430 |
| Fc1ccc(CC2=NNC(=O)c3c(Oc4ccc(cc4)C(=O)OCC5CCOCC5)cccc23)cc1C(=O)N6CCN(CC6)C(=O)C7CC7 | Olaparib 2534 |
| CC(C)(N)c1ccc(Cl)c(Oc2cccc3C(=NNC(=O)c23)Cc4ccc(F)c(c4)C(=O)N5CCN(CC5)C(=O)C6CC6)c1 | Olaparib 2338 |
| CC(=O)c1cc(Oc2cccc3C(=NNC(=O)c23)Cc4ccc(F)c(c4)C(=O)N5CCN(CC5)C(=O)C6CC6)c(C)cc1O | Olaparib 2932 |
| COc1c(C)cc(cc1C)C(C)(C)NC(=O)c2cccc3C(=NNC(=O)c23)Cc4ccc(F)c(c4)C(=O)N5CCN(CC5)C(=O)C6CC6 | Olaparib 153 |
| COC(=O)C(F)(F)c1cccc2C(=NNC(=O)c12)Cc3ccc(F)c(c3)C(=O)N4CCN(CC4)C(=O)C5CC5 | Olaparib 1631 |
| Fc1ccc(CC2=NNC(=O)c3c(cccc23)C(=O)OCc4oc(cc4)c5cccnc5)cc1C(=O)N6CCN(CC6)C(=O)C7CC7 | Olaparib 1095 |
| CCOC(=O)C(=[N+]=[N-])C(F)(F)Oc1cccc2C(=NNC(=O)c12)Cc3ccc(F)c(c3)C(=O)N4CCN(CC4)C(=O)C5CC5 | Olaparib 2122 |
| CN[C@@]1(COc2cccc3C(=NNC(=O)c23)Cc4ccc(F)c(c4)C(=O)N5CCN(CC5)C(=O)C6CC6)CCCS(=O)(=O)C1 | Olaparib 2879 |
| Oc1cc(Oc2cccc3C(=NNC(=O)c23)Cc4ccc(F)c(c4)C(=O)N5CCN(CC5)C(=O)C6CC6)cc(F)c1[C@H]7CCC(=O)N7 | Olaparib 2448 |
| C[C@@](CO)(COc1cccc2C(=NNC(=O)c12)Cc3ccc(F)c(c3)C(=O)N4CCN(CC4)C(=O)C5CC5)c6ccccc6 | Olaparib 2928 |
| C[C@H](COC(=O)c1cccc2C(=NNC(=O)c12)Cc3ccc(F)c(c3)C(=O)N4CCN(CC4)C(=O)C5CC5)Oc6ccccc6 | Olaparib 864 |
| COc1ccc(cc1OC(=O)c2cccc3C(=NNC(=O)c23)Cc4ccc(F)c(c4)C(=O)N5CCN(CC5)C(=O)C6CC6)C(C)(C)C | Olaparib 763 |
| COC(=O)[C@](F)(Cl)c1cccc2C(=NNC(=O)c12)Cc3ccc(F)c(c3)C(=O)N4CCN(CC4)C(=O)C5CC5 | Olaparib 1630 |
| Fc1ccc(CC2=NNC(=O)c3c(cccc23)C(=O)OCc4onc(c4)c5ccccc5)cc1C(=O)N6CCN(CC6)C(=O)C7CC7 | Olaparib 1093 |
| CNC(=O)N(C)CC(=O)Oc1cccc2C(=NNC(=O)c12)Cc3ccc(F)c(c3)C(=O)N4CCN(CC4)C(=O)C5CC5 | Olaparib 1222 |
| CN1CCN(CC1)C(=N)NC(=O)c2cccc3C(=NNC(=O)c23)Cc4ccc(F)c(c4)C(=O)N5CCN(CC5)C(=O)C6CC6 | Olaparib 193 |
| Fc1ccc(CC2=NNC(=O)c3c(Oc4c(F)cc(cc4[C@H]5CCC(=O)N5)C#N)cccc23)cc1C(=O)N6CCN(CC6)C(=O)C7CC7 | Olaparib 3082 |
| CCCCOc1cc(ccn1)c2cccc3C(=NNC(=O)c23)Cc4ccc(F)c(c4)C(=O)N5CCN(CC5)C(=O)C6CC6 | Olaparib 1699 |
| NCCc1ccccc1OC(=O)c2cccc3C(=NNC(=O)c23)Cc4ccc(F)c(c4)C(=O)N5CCN(CC5)C(=O)C6CC6 | Olaparib 927 |
| COc1ccccc1[C@@H](OC(=O)c2cccc3C(=NNC(=O)c23)Cc4ccc(F)c(c4)C(=O)N5CCN(CC5)C(=O)C6CC6)C(C)C | Olaparib 764 |
| COC(=O)C(Cl)(Cl)c1cccc2C(=NNC(=O)c12)Cc3ccc(F)c(c3)C(=O)N4CCN(CC4)C(=O)C5CC5 | Olaparib 1608 |
| Oc1cc(OC(=O)c2cccc3C(=NNC(=O)c23)Cc4ccc(F)c(c4)C(=O)N5CCN(CC5)C(=O)C6CC6)c7ccccc7n1 | Olaparib 1109 |
| COC(=O)[C@H](O)CNC(=O)c1cccc2C(=NNC(=O)c12)Cc3ccc(F)c(c3)C(=O)N4CCN(CC4)C(=O)C5CC5 | Olaparib 48 |
| CN(CC(=O)Nc1cccc2C(=NNC(=O)c12)Cc3ccc(F)c(c3)C(=O)N4CCN(CC4)C(=O)C5CC5)C(=O)[C@H]6CCSC6 | Olaparib 478 |
| Oc1cccc(OC(=O)c2cccc3C(=NNC(=O)c23)Cc4ccc(F)c(c4)C(=O)N5CCN(CC5)C(=O)C6CC6)c1C7(CC7)C#N | Olaparib 1094 |
| CCc1ccc(CCOc2cccc3C(=NNC(=O)c23)Cc4ccc(F)c(c4)C(=O)N5CCN(CC5)C(=O)C6CC6)nc1 | Olaparib 2969 |
| Cc1cc(ccc1F)[C@H](O)C(=O)Oc2cccc3C(=NNC(=O)c23)Cc4ccc(F)c(c4)C(=O)N5CCN(CC5)C(=O)C6CC6 | Olaparib 1473 |
| CC(C)(N)Cc1ccc(OC(=O)c2cccc3C(=NNC(=O)c23)Cc4ccc(F)c(c4)C(=O)N5CCN(CC5)C(=O)C6CC6)cc1 | Olaparib 817 |
| C[C@@](F)(Cl)C(=O)Oc1cccc2C(=NNC(=O)c12)Cc3ccc(F)c(c3)C(=O)N4CCN(CC4)C(=O)C5CC5 | Olaparib 1308 |
| CNCCc1c[nH]c2ccc(OC(=O)c3cccc4C(=NNC(=O)c34)Cc5ccc(F)c(c5)C(=O)N6CCN(CC6)C(=O)C7CC7)cc12 | Olaparib 1071 |
| COC(=O)[C@@H](CO)NC(=O)c1cccc2C(=NNC(=O)c12)Cc3ccc(F)c(c3)C(=O)N4CCN(CC4)C(=O)C5CC5 | Olaparib 43 |
| OCC1CCN(CC1)C(=O)C(=O)Nc2cccc3C(=NNC(=O)c23)Cc4ccc(F)c(c4)C(=O)N5CCN(CC5)C(=O)C6CC6 | Olaparib 489 |
| CCN[C@@H](C)C1CCN(CC1)c2ccc(Oc3cccc4C(=NNC(=O)c34)Cc5ccc(F)c(c5)C(=O)N6CCN(CC6)C(=O)C7CC7)cn2 | Olaparib 2503 |
| CC(C)(C#N)c1ccc(Oc2cccc3C(=NNC(=O)c23)Cc4ccc(F)c(c4)C(=O)N5CCN(CC5)C(=O)C6CC6)c(F)c1 | Olaparib 2899 |
| CN(CC(=O)Oc1cccc2C(=NNC(=O)c12)Cc3ccc(F)c(c3)C(=O)N4CCN(CC4)C(=O)C5CC5)c6ccccc6F | Olaparib 1475 |
| COc1cc(cc(F)c1F)C(C)(C)NC(=O)c2cccc3C(=NNC(=O)c23)Cc4ccc(F)c(c4)C(=O)N5CCN(CC5)C(=O)C6CC6 | Olaparib 148 |
| Fc1ccc(CC2=NNC(=O)c3c(NC(=O)C(F)(F)Cl)cccc23)cc1C(=O)N4CCN(CC4)C(=O)C5CC5 | Olaparib 354 |
| O[C@H](Cn1ccnc1)c2ccc(Oc3cccc4C(=NNC(=O)c34)Cc5ccc(F)c(c5)C(=O)N6CCN(CC6)C(=O)C7CC7)cc2 | Olaparib 2559 |
| CC(C)(Oc1cccc2C(=NNC(=O)c12)Cc3ccc(F)c(c3)C(=O)N4CCN(CC4)C(=O)C5CC5)S(=O)(=O)[O-] | Olaparib 2757 |
| CCNc1nc(CC(F)(F)Oc2cccc3C(=NNC(=O)c23)Cc4ccc(F)c(c4)C(=O)N5CCN(CC5)C(=O)C6CC6)nc7CCCCCc17 | Olaparib 2449 |
| Fc1ccc(CC2=NNC(=O)c3c(NC(=O)CN4CCOCC4=O)cccc23)cc1C(=O)N5CCN(CC5)C(=O)C6CC6 | Olaparib 505 |
| COc1nccc(c1C)c2cccc3C(=NNC(=O)c23)Cc4ccc(F)c(c4)C(=O)N5CCN(CC5)C(=O)C6CC6 | Olaparib 1984 |
| C[C@@](N)(CCc1ccc2OCOc2c1)C(F)(F)Oc3cccc4C(=NNC(=O)c34)Cc5ccc(F)c(c5)C(=O)N6CCN(CC6)C(=O)C7CC7 | Olaparib 2479 |
| Oc1ccccc1CCC(=O)Oc2cccc3C(=NNC(=O)c23)Cc4ccc(F)c(c4)C(=O)N5CCN(CC5)C(=O)C6CC6 | Olaparib 1491 |
| CC(C)(NC(=O)Cc1cccc(Oc2cccc3C(=NNC(=O)c23)Cc4ccc(F)c(c4)C(=O)N5CCN(CC5)C(=O)C6CC6)c1)C#C | Olaparib 2867 |
| NCCCNCCCc1cccc2C(=NNC(=O)c12)Cc3ccc(F)c(c3)C(=O)N4CCN(CC4)C(=O)C5CC5 | Olaparib 1812 |
| COc1ccc2[nH]c(CC(=O)Oc3cccc4C(=NNC(=O)c34)Cc5ccc(F)c(c5)C(=O)N6CCN(CC6)C(=O)C7CC7)cc2c1 | Olaparib 1560 |
| OC(=O)[C@H](CC#N)NC(=O)c1cccc2C(=NNC(=O)c12)Cc3ccc(F)c(c3)C(=O)N4CCN(CC4)C(=O)C5CC5 | Olaparib 80 |
| Fc1ccc(CC2=NNC(=O)c3c(OC(=O)CN4CCOCC4=O)cccc23)cc1C(=O)N5CCN(CC5)C(=O)C6CC6 | Olaparib 1494 |
| CCN(CC1CC1)c2ccc(Oc3cccc4C(=NNC(=O)c34)Cc5ccc(F)c(c5)C(=O)N6CCN(CC6)C(=O)C7CC7)nn2 | Olaparib 2583 |
| Oc1cccc(CCOc2cccc3C(=NNC(=O)c23)Cc4ccc(F)c(c4)C(=O)N5CCN(CC5)C(=O)C6CC6)c1 | Olaparib 3001 |
| Fc1ccc(CC2=NNC(=O)c3c(OCNC(=O)Cc4ccccc4)cccc23)cc1C(=O)N5CCN(CC5)C(=O)C6CC6 | Olaparib 2938 |
| CC(C)(C)NC(=O)c1ccc(Oc2cccc3C(=NNC(=O)c23)Cc4ccc(F)c(c4)C(=O)N5CCN(CC5)C(=O)C6CC6)cc1 | Olaparib 2881 |
| NCCNCCCc1cccc2C(=NNC(=O)c12)Cc3ccc(F)c(c3)C(=O)N4CCN(CC4)C(=O)C5CC5 | Olaparib 1801 |
| CCOC(=O)c1cc2ccc(Oc3cccc4C(=NNC(=O)c34)Cc5ccc(F)c(c5)C(=O)N6CCN(CC6)C(=O)C7CC7)cc2[nH]1 | Olaparib 2556 |
| NC[C@H](O)[C@H](O)CNC(=O)c1cccc2C(=NNC(=O)c12)Cc3ccc(F)c(c3)C(=O)N4CCN(CC4)C(=O)C5CC5 | Olaparib 39 |
| C\C=C\C(=O)N(C)C(C)(C)C(=O)Oc1cccc2C(=NNC(=O)c12)Cc3ccc(F)c(c3)C(=O)N4CCN(CC4)C(=O)C5CC5 | Olaparib 1180 |
| CC(C)(N1CCN(CC(F)(F)Oc2cccc3C(=NNC(=O)c23)Cc4ccc(F)c(c4)C(=O)N5CCN(CC5)C(=O)C6CC6)CC1)C(=S)N | Olaparib 2178 |
| Fc1ccc(CC2=NNC(=O)c3c(O[C@H]4CCCN(Cc5ccnc(c5)C#N)C4)cccc23)cc1C(=O)N6CCN(CC6)C(=O)C7CC7 | Olaparib 3085 |
| Cc1nccc(c1N)c2cccc3C(=NNC(=O)c23)Cc4ccc(F)c(c4)C(=O)N5CCN(CC5)C(=O)C6CC6 | Olaparib 2005 |
| Fc1ccc(CC2=NNC(=O)c3c(OCCC(=O)Nc4ccccc4)cccc23)cc1C(=O)N5CCN(CC5)C(=O)C6CC6 | Olaparib 2937 |
| CN(C)[C@H](COC(=O)c1cccc2C(=NNC(=O)c12)Cc3ccc(F)c(c3)C(=O)N4CCN(CC4)C(=O)C5CC5)c6ccc(F)cc6F | Olaparib 719 |
| F[C@H](Oc1cccc2C(=NNC(=O)c12)Cc3ccc(F)c(c3)C(=O)N4CCN(CC4)C(=O)C5CC5)OC(F)(F)C(F)F | Olaparib 2137 |
| COc1ccc2c(Cl)c(COC(=O)c3cccc4C(=NNC(=O)c34)Cc5ccc(F)c(c5)C(=O)N6CCN(CC6)C(=O)C7CC7)sc2c1 | Olaparib 1010 |
| CN(CC(=O)Nc1cccc2C(=NNC(=O)c12)Cc3ccc(F)c(c3)C(=O)N4CCN(CC4)C(=O)C5CC5)C(=O)C=C(C)C | Olaparib 278 |
| CC(C)[C@@H]1O[C@H](CN)[C@@H](CNC(=O)c2cccc3C(=NNC(=O)c23)Cc4ccc(F)c(c4)C(=O)N5CCN(CC5)C(=O)C6CC6)O1 | Olaparib 161 |
| Nc1ccc(C#N)c(c1)C(F)(F)c2cccc3C(=NNC(=O)c23)Cc4ccc(F)c(c4)C(=O)N5CCN(CC5)C(=O)C6CC6 | Olaparib 1698 |
| C[C@]1(C[C@H](CCO1)Oc2cccc3C(=NNC(=O)c23)Cc4ccc(F)c(c4)C(=O)N5CCN(CC5)C(=O)C6CC6)c7cnccn7 | Olaparib 3107 |
| Cc1cnc(Br)c(CC(=O)Oc2cccc3C(=NNC(=O)c23)Cc4ccc(F)c(c4)C(=O)N5CCN(CC5)C(=O)C6CC6)c1 | Olaparib 1420 |
| CN(C)c1ccc(CCOC(=O)c2cccc3C(=NNC(=O)c23)Cc4ccc(F)c(c4)C(=O)N5CCN(CC5)C(=O)C6CC6)cc1 | Olaparib 815 |
| C[C@@H](CC(=C)C)Oc1cccc2C(=NNC(=O)c12)Cc3ccc(F)c(c3)C(=O)N4CCN(CC4)C(=O)C5CC5 | Olaparib 2853 |
| Cc1nc2ccccc2n1COC(=O)c3cccc4C(=NNC(=O)c34)Cc5ccc(F)c(c5)C(=O)N6CCN(CC6)C(=O)C7CC7 | Olaparib 1102 |
| C[C@H](NC(=O)C=C(C)C)C(=O)Nc1cccc2C(=NNC(=O)c12)Cc3ccc(F)c(c3)C(=O)N4CCN(CC4)C(=O)C5CC5 | Olaparib 279 |
| Fc1ccc(CC2=NNC(=O)c3c(OC(F)(F)CCNC(=O)C4(CCOCC4)C#N)cccc23)cc1C(=O)N5CCN(CC5)C(=O)C6CC6 | Olaparib 2205 |
| Cc1cccc(Oc2cccc3C(=NNC(=O)c23)Cc4ccc(F)c(c4)C(=O)N5CCN(CC5)C(=O)C6CC6)c1O | Olaparib 3022 |
| CC1(C)OCc2c(Cl)nc(Oc3cccc4C(=NNC(=O)c34)Cc5ccc(F)c(c5)C(=O)N6CCN(CC6)C(=O)C7CC7)nc12 | Olaparib 2568 |
| CC(=O)Nc1cccc(Oc2cccc3C(=NNC(=O)c23)Cc4ccc(F)c(c4)C(=O)N5CCN(CC5)C(=O)C6CC6)c1 | Olaparib 2384 |
| CNC[C@H](OC(=O)c1cccc2C(=NNC(=O)c12)Cc3ccc(F)c(c3)C(=O)N4CCN(CC4)C(=O)C5CC5)c6ccc(C)c(F)c6 | Olaparib 755 |
| Fc1ccc(CC2=NNC(=O)c3c(cccc23)C(=C)C(F)(F)F)cc1C(=O)N4CCN(CC4)C(=O)C5CC5 | Olaparib 1927 |
| Oc1nc(Oc2cccc3C(=NNC(=O)c23)Cc4ccc(F)c(c4)C(=O)N5CCN(CC5)C(=O)C6CC6)nc7ccccc17 | Olaparib 3132 |
| CN(CC(=O)Oc1cccc2C(=NNC(=O)c12)Cc3ccc(F)c(c3)C(=O)N4CCN(CC4)C(=O)C5CC5)C(=O)C=C(C)C | Olaparib 1186 |
| OC(=O)\C=C\C(=O)c1ccc(Oc2cccc3C(=NNC(=O)c23)Cc4ccc(F)c(c4)C(=O)N5CCN(CC5)C(=O)C6CC6)c(F)c1 | Olaparib 2280 |
| C[C@@H]1CC[C@](COC(=O)c2cccc3C(=NNC(=O)c23)Cc4ccc(F)c(c4)C(=O)N5CCN(CC5)C(=O)C6CC6)(CC1)NC(=O)C7CC=CC7 | Olaparib 997 |
| Cc1nccc(c1O)c2cccc3C(=NNC(=O)c23)Cc4ccc(F)c(c4)C(=O)N5CCN(CC5)C(=O)C6CC6 | Olaparib 2002 |
| Cc1ccc(cc1Oc2cccc3C(=NNC(=O)c23)Cc4ccc(F)c(c4)C(=O)N5CCN(CC5)C(=O)C6CC6)C(=O)O | Olaparib 2410 |
| CC(C)(NC(=O)c1cccc2C(=NNC(=O)c12)Cc3ccc(F)c(c3)C(=O)N4CCN(CC4)C(=O)C5CC5)c6ccc(O)c(F)c6 | Olaparib 165 |
| CC[C@@H](C)Cc1cccc2C(=NNC(=O)c12)Cc3ccc(F)c(c3)C(=O)N4CCN(CC4)C(=O)C5CC5 | Olaparib 1850 |
| Fc1ccc(CC2=NNC(=O)c3c(cccc23)C(=O)Oc4cncc5c(Br)coc45)cc1C(=O)N6CCN(CC6)C(=O)C7CC7 | Olaparib 1032 |
| C[C@H](NC(=O)C=C(C)C)C(=O)Oc1cccc2C(=NNC(=O)c12)Cc3ccc(F)c(c3)C(=O)N4CCN(CC4)C(=O)C5CC5 | Olaparib 1187 |
| OC(=O)CC(=O)Nc1ccc(Cl)c(c1)c2cccc3C(=NNC(=O)c23)Cc4ccc(F)c(c4)C(=O)N5CCN(CC5)C(=O)C6CC6 | Olaparib 1652 |
| Fc1ccc(CC2=NNC(=O)c3c(NC(=O)CC4(CCCC4)N5CCCCCC5=O)cccc23)cc1C(=O)N6CCN(CC6)C(=O)C7CC7 | Olaparib 524 |
| Cc1cc(c(O)cn1)c2cccc3C(=NNC(=O)c23)Cc4ccc(F)c(c4)C(=O)N5CCN(CC5)C(=O)C6CC6 | Olaparib 2003 |
| O[C@@H](C(=O)Nc1cccc2C(=NNC(=O)c12)Cc3ccc(F)c(c3)C(=O)N4CCN(CC4)C(=O)C5CC5)c6ccc(Cl)cc6 | Olaparib 490 |
| C[C@@H](O)CCc1ccc(OC(=O)c2cccc3C(=NNC(=O)c23)Cc4ccc(F)c(c4)C(=O)N5CCN(CC5)C(=O)C6CC6)cc1 | Olaparib 809 |
| CC(=CC[C@H](NC(=O)c1cccc2C(=NNC(=O)c12)Cc3ccc(F)c(c3)C(=O)N4CCN(CC4)C(=O)C5CC5)C(=O)O)C | Olaparib 6 |
| CC(C)CC[C@H](C)n1c(N)nc2ccc(Oc3cccc4C(=NNC(=O)c34)Cc5ccc(F)c(c5)C(=O)N6CCN(CC6)C(=O)C7CC7)cc12 | Olaparib 2513 |
| COc1cc(ccn1)c2cccc3C(=NNC(=O)c23)Cc4ccc(F)c(c4)C(=O)N5CCN(CC5)C(=O)C6CC6 | Olaparib 1912 |
| Fc1ccc(CC2=NNC(=O)c3c(Oc4cc5nn[nH]c5cc4Cl)cccc23)cc1C(=O)N6CCN(CC6)C(=O)C7CC7 | Olaparib 2613 |
| Fc1ccc(CC2=NNC(=O)c3c(OC(F)(F)S(=O)(=O)C#Cc4ccccc4)cccc23)cc1C(=O)N5CCN(CC5)C(=O)C6CC6 | Olaparib 2230 |
| Fc1ccc(CC2=NNC(=O)c3c(OC(=O)CC4(CCCC4)N5CCCCCC5=O)cccc23)cc1C(=O)N6CCN(CC6)C(=O)C7CC7 | Olaparib 1529 |
| NC(=N)c1cccc(Oc2cccc3C(=NNC(=O)c23)Cc4ccc(F)c(c4)C(=O)N5CCN(CC5)C(=O)C6CC6)c1 | Olaparib 2409 |
| C[C@@](CO)(COC(=O)c1cccc2C(=NNC(=O)c12)Cc3ccc(F)c(c3)C(=O)N4CCN(CC4)C(=O)C5CC5)c6ccccc6 | Olaparib 808 |
| CC1=CC(=O)N(C1=O)c2ccc(Oc3cccc4C(=NNC(=O)c34)Cc5ccc(F)c(c5)C(=O)N6CCN(CC6)C(=O)C7CC7)cc2 | Olaparib 2470 |
| C[C@](NC(=O)c1cccc2C(=NNC(=O)c12)Cc3ccc(F)c(c3)C(=O)N4CCN(CC4)C(=O)C5CC5)(C=C)C(=O)O | Olaparib 78 |
| Oc1ccc(cc1c2cccc3C(=NNC(=O)c23)Cc4ccc(F)c(c4)C(=O)N5CCN(CC5)C(=O)C6CC6)C#N | Olaparib 1724 |
| C[C@@H](N(C)C(=O)c1cncc(Oc2cccc3C(=NNC(=O)c23)Cc4ccc(F)c(c4)C(=O)N5CCN(CC5)C(=O)C6CC6)c1)C(C)(C)C | Olaparib 2857 |
| C[C@@H](NC(=O)c1cccc2C(=NNC(=O)c12)Cc3ccc(F)c(c3)C(=O)N4CCN(CC4)C(=O)C5CC5)C6CCN(CC6)[C@H]7CCO[C@@H](C)C7 | Olaparib 221 |
| COc1cccc(Oc2cccc3C(=NNC(=O)c23)Cc4ccc(F)c(c4)C(=O)N5CCN(CC5)C(=O)C6CC6)c1C=O | Olaparib 2411 |
| CC(C)(N)c1cc(F)cc(F)c1OC(=O)c2cccc3C(=NNC(=O)c23)Cc4ccc(F)c(c4)C(=O)N5CCN(CC5)C(=O)C6CC6 | Olaparib 740 |
| CC(C)[C@@H]1C(=NN(C1=O)c2ccc(Oc3cccc4C(=NNC(=O)c34)Cc5ccc(F)c(c5)C(=O)N6CCN(CC6)C(=O)C7CC7)cc2C)C | Olaparib 2515 |
| CN(CC(=O)Oc1cccc2C(=NNC(=O)c12)Cc3ccc(F)c(c3)C(=O)N4CCN(CC4)C(=O)C5CC5)C(=O)C=C | Olaparib 1238 |
| Cc1cc(ncn1)c2cccc3C(=NNC(=O)c23)Cc4ccc(F)c(c4)C(=O)N5CCN(CC5)C(=O)C6CC6 | Olaparib 2026 |
| Cc1c(N)cccc1C(=O)NCCC(F)(F)Oc2cccc3C(=NNC(=O)c23)Cc4ccc(F)c(c4)C(=O)N5CCN(CC5)C(=O)C6CC6 | Olaparib 2211 |
| Fc1ccc(CC2=NNC(=O)c3c(OC(=O)CN4CC[N@]5CCCC[C@@H]5C4)cccc23)cc1C(=O)N6CCN(CC6)C(=O)C7CC7 | Olaparib 1564 |
| Oc1cc(Cl)ccc1COC(=O)c2cccc3C(=NNC(=O)c23)Cc4ccc(F)c(c4)C(=O)N5CCN(CC5)C(=O)C6CC6 | Olaparib 842 |
| Cc1cc(C)cc(OCCOC(=O)c2cccc3C(=NNC(=O)c23)Cc4ccc(F)c(c4)C(=O)N5CCN(CC5)C(=O)C6CC6)c1 | Olaparib 807 |
| CC1(CNC(=O)c2ccccc2OC(=O)c3cccc4C(=NNC(=O)c34)Cc5ccc(F)c(c5)C(=O)N6CCN(CC6)C(=O)C7CC7)CCCC1 | Olaparib 1005 |
| COC(=O)\C=C\C(=O)Oc1cccc2C(=NNC(=O)c12)Cc3ccc(F)c(c3)C(=O)N4CCN(CC4)C(=O)C5CC5 | Olaparib 1298 |
| Oc1cncc(Cl)c1c2cccc3C(=NNC(=O)c23)Cc4ccc(F)c(c4)C(=O)N5CCN(CC5)C(=O)C6CC6 | Olaparib 1975 |
| CC(C)(C(=O)O)c1cc(F)c(Oc2cccc3C(=NNC(=O)c23)Cc4ccc(F)c(c4)C(=O)N5CCN(CC5)C(=O)C6CC6)cc1O | Olaparib 2269 |
| N[C@]1(CC[C@H]2CNC[C@H]12)C(=O)Nc3cccc4C(=NNC(=O)c34)Cc5ccc(F)c(c5)C(=O)N6CCN(CC6)C(=O)C7CC7 | Olaparib 545 |
| NC(=N)c1ccc(Oc2cccc3C(=NNC(=O)c23)Cc4ccc(F)c(c4)C(=O)N5CCN(CC5)C(=O)C6CC6)cc1 | Olaparib 2317 |
| C[C@@H](CCc1ccc(O)cc1)OC(=O)c2cccc3C(=NNC(=O)c23)Cc4ccc(F)c(c4)C(=O)N5CCN(CC5)C(=O)C6CC6 | Olaparib 810 |
| C[C@@H](N1C[C@@H](CC1=O)OC(=O)c2cccc3C(=NNC(=O)c23)Cc4ccc(F)c(c4)C(=O)N5CCN(CC5)C(=O)C6CC6)c7ccccc7C | Olaparib 1029 |
| C[C@H](N(C)C(=O)C(C)(C)C)C(=O)Nc1cccc2C(=NNC(=O)c12)Cc3ccc(F)c(c3)C(=O)N4CCN(CC4)C(=O)C5CC5 | Olaparib 275 |
| Oc1c(Cl)nccc1c2cccc3C(=NNC(=O)c23)Cc4ccc(F)c(c4)C(=O)N5CCN(CC5)C(=O)C6CC6 | Olaparib 1974 |
| CCC[C@H](OC(=O)c1cccc2C(=NNC(=O)c12)Cc3ccc(F)c(c3)C(=O)N4CCN(CC4)C(=O)C5CC5)c6cc(Cl)cnc6N | Olaparib 720 |
| C[C@H](N)c1ccc(OCc2ccc(Oc3cccc4C(=NNC(=O)c34)Cc5ccc(F)c(c5)C(=O)N6CCN(CC6)C(=O)C7CC7)cc2Cl)cc1 | Olaparib 2430 |
| Oc1ccc(cc1)C(=O)C(F)(F)Oc2cccc3C(=NNC(=O)c23)Cc4ccc(F)c(c4)C(=O)N5CCN(CC5)C(=O)C6CC6 | Olaparib 2334 |
| CC(C)(C#N)c1ccccc1COC(=O)c2cccc3C(=NNC(=O)c23)Cc4ccc(F)c(c4)C(=O)N5CCN(CC5)C(=O)C6CC6 | Olaparib 777 |
| CCCc1ccccc1N2C[C@@H](CC2=O)OC(=O)c3cccc4C(=NNC(=O)c34)Cc5ccc(F)c(c5)C(=O)N6CCN(CC6)C(=O)C7CC7 | Olaparib 1030 |
| C[C@H](NC(=O)c1cccc2C(=NNC(=O)c12)Cc3ccc(F)c(c3)C(=O)N4CCN(CC4)C(=O)C5CC5)C(=O)OC(C)(C)C | Olaparib 5 |
| Fc1ccc(CC2=NNC(=O)c3c(CCCn4ccnc4)cccc23)cc1C(=O)N5CCN(CC5)C(=O)C6CC6 | Olaparib 1841 |
| COc1ccc(cc1)[C@@H](N)C(=O)Nc2cccc3C(=NNC(=O)c23)Cc4ccc(F)c(c4)C(=O)N5CCN(CC5)C(=O)C6CC6 | Olaparib 497 |
| CCC[C@@H](OC(=O)c1cccc2C(=NNC(=O)c12)Cc3ccc(F)c(c3)C(=O)N4CCN(CC4)C(=O)C5CC5)c6ccnc7ccccc67 | Olaparib 1054 |
| Fc1ccc(CC2=NNC(=O)c3c(NC(=O)Cc4ccccn4)cccc23)cc1C(=O)N5CCN(CC5)C(=O)C6CC6 | Olaparib 514 |
| C[C@H](N)Cc1ccc(OC(=O)c2cccc3C(=NNC(=O)c23)Cc4ccc(F)c(c4)C(=O)N5CCN(CC5)C(=O)C6CC6)cc1 | Olaparib 875 |
| Cc1ccc(CN2C[C@@H](CC2=O)OC(=O)c3cccc4C(=NNC(=O)c34)Cc5ccc(F)c(c5)C(=O)N6CCN(CC6)C(=O)C7CC7)cc1 | Olaparib 1051 |
| COC(=O)C(C)(C)CNC(=O)c1cccc2C(=NNC(=O)c12)Cc3ccc(F)c(c3)C(=O)N4CCN(CC4)C(=O)C5CC5 | Olaparib 28 |
| OCc1occc1c2cccc3C(=NNC(=O)c23)Cc4ccc(F)c(c4)C(=O)N5CCN(CC5)C(=O)C6CC6 | Olaparib 2015 |
| COc1ccc(cc1)[C@@H](N)C(=O)Oc2cccc3C(=NNC(=O)c23)Cc4ccc(F)c(c4)C(=O)N5CCN(CC5)C(=O)C6CC6 | Olaparib 1477 |
| O[C@@H](CCc1ccsc1)c2cc(Oc3cccc4C(=NNC(=O)c34)Cc5ccc(F)c(c5)C(=O)N6CCN(CC6)C(=O)C7CC7)ccc2F | Olaparib 2496 |
| NC(=O)c1cccc(Oc2cccc3C(=NNC(=O)c23)Cc4ccc(F)c(c4)C(=O)N5CCN(CC5)C(=O)C6CC6)c1 | Olaparib 2404 |
| CC(C)(NC(=O)c1cccc2C(=NNC(=O)c12)Cc3ccc(F)c(c3)C(=O)N4CCN(CC4)C(=O)C5CC5)c6ccccc6C#N | Olaparib 175 |
| Cc1cc(OC(=O)c2cccc3C(=NNC(=O)c23)Cc4ccc(F)c(c4)C(=O)N5CCN(CC5)C(=O)C6CC6)ccc1[C@@H]7CCC(=O)N7 | Olaparib 1069 |
| CC(C)C(=O)NCC(=O)Nc1cccc2C(=NNC(=O)c12)Cc3ccc(F)c(c3)C(=O)N4CCN(CC4)C(=O)C5CC5 | Olaparib 309 |
| Cc1cc(n[nH]1)c2cccc3C(=NNC(=O)c23)Cc4ccc(F)c(c4)C(=O)N5CCN(CC5)C(=O)C6CC6 | Olaparib 2038 |
| N[C@H](Cc1ccc(Oc2cccc3C(=NNC(=O)c23)Cc4ccc(F)c(c4)C(=O)N5CCN(CC5)C(=O)C6CC6)cc1Cl)C(=O)O | Olaparib 2231 |
| Fc1ccc(CC2=NNC(=O)c3c2cccc3c4cccc(OCc5ccccc5)n4)cc1C(=O)N6CCN(CC6)C(=O)C7CC7 | Olaparib 1886 |
| Fc1ccc(CC2=NNC(=O)c3c(Oc4ccc(OCC=O)cc4)cccc23)cc1C(=O)N5CCN(CC5)C(=O)C6CC6 | Olaparib 2413 |
| CN(C)Cc1cc(Cl)ccc1OC(=O)c2cccc3C(=NNC(=O)c23)Cc4ccc(F)c(c4)C(=O)N5CCN(CC5)C(=O)C6CC6 | Olaparib 746 |
| Fc1ccc(CC2=NNC(=O)c3c(cccc23)C(=O)OCc4cccc(c4)[C@@H]5CCC(=O)N5)cc1C(=O)N6CCN(CC6)C(=O)C7CC7 | Olaparib 1068 |
| CN(C)C(=O)CCC(=O)Oc1cccc2C(=NNC(=O)c12)Cc3ccc(F)c(c3)C(=O)N4CCN(CC4)C(=O)C5CC5 | Olaparib 1230 |
| Fc1ccc(CC2=NNC(=O)c3c2cccc3c4ccsc4C#N)cc1C(=O)N5CCN(CC5)C(=O)C6CC6 | Olaparib 2001 |
| CC(C)(C)c1ccc(cc1)[C@@]2(C)C[C@H](CCO2)Oc3cccc4C(=NNC(=O)c34)Cc5ccc(F)c(c5)C(=O)N6CCN(CC6)C(=O)C7CC7 | Olaparib 3054 |
| CON(C)C(=O)c1ccc(Cl)c(Oc2cccc3C(=NNC(=O)c23)Cc4ccc(F)c(c4)C(=O)N5CCN(CC5)C(=O)C6CC6)c1 | Olaparib 2232 |
| OC(=O)c1cc(F)cc(Oc2cccc3C(=NNC(=O)c23)Cc4ccc(F)c(c4)C(=O)N5CCN(CC5)C(=O)C6CC6)c1 | Olaparib 2399 |
| CC(C)c1cc(OC(=O)c2cccc3C(=NNC(=O)c23)Cc4ccc(F)c(c4)C(=O)N5CCN(CC5)C(=O)C6CC6)ccc1O | Olaparib 868 |
| Fc1ccc(CC2=NNC(=O)c3c(cccc23)C(=O)OCc4ccc5NC(=O)CCCc5c4)cc1C(=O)N6CCN(CC6)C(=O)C7CC7 | Olaparib 1070 |
| C[C@H](CCNC(=O)c1cccc2C(=NNC(=O)c12)Cc3ccc(F)c(c3)C(=O)N4CCN(CC4)C(=O)C5CC5)C(=O)O | Olaparib 60 |
| Fc1ccc(CC2=NNC(=O)c3c2cccc3C4=CC=CNC4=O)cc1C(=O)N5CCN(CC5)C(=O)C6CC6 | Olaparib 2025 |
| CN(Cc1ccccc1c2cccc3C(=NNC(=O)c23)Cc4ccc(F)c(c4)C(=O)N5CCN(CC5)C(=O)C6CC6)c7ccc(F)cc7 | Olaparib 2050 |
| CN(CC(=O)Oc1cccc2C(=NNC(=O)c12)Cc3ccc(F)c(c3)C(=O)N4CCN(CC4)C(=O)C5CC5)c6cccc(Cl)c6C#N | Olaparib 1424 |
| OC(=O)c1cc(Br)c(Oc2cccc3C(=NNC(=O)c23)Cc4ccc(F)c(c4)C(=O)N5CCN(CC5)C(=O)C6CC6)c(F)c1F | Olaparib 2193 |
| CC(C)(C#N)c1cc(F)c(OC(=O)c2cccc3C(=NNC(=O)c23)Cc4ccc(F)c(c4)C(=O)N5CCN(CC5)C(=O)C6CC6)c(F)c1 | Olaparib 726 |
| C[C@@H](OC(=O)c1cccc2C(=NNC(=O)c12)Cc3ccc(F)c(c3)C(=O)N4CCN(CC4)C(=O)C5CC5)\C=C\c6ccc7OCOc7c6 | Olaparib 1066 |
| COC(=O)[C@H](C)CNC(=O)c1cccc2C(=NNC(=O)c12)Cc3ccc(F)c(c3)C(=O)N4CCN(CC4)C(=O)C5CC5 | Olaparib 66 |
| Fc1ccc(CC2=NNC(=O)c3c2cccc3C(F)(F)C(=O)N4CC=CC4)cc1C(=O)N5CCN(CC5)C(=O)C6CC6 | Olaparib 1715 |
| C[C@@H]1CCC[C@@H](C1)N(C)Cc2ccccc2c3cccc4C(=NNC(=O)c34)Cc5ccc(F)c(c5)C(=O)N6CCN(CC6)C(=O)C7CC7 | Olaparib 2049 |
| Nc1ccc(NCC(=O)Oc2cccc3C(=NNC(=O)c23)Cc4ccc(F)c(c4)C(=O)N5CCN(CC5)C(=O)C6CC6)c(Cl)c1 | Olaparib 1453 |
| OC(=O)c1cc(Cl)c(Oc2cccc3C(=NNC(=O)c23)Cc4ccc(F)c(c4)C(=O)N5CCN(CC5)C(=O)C6CC6)cc1Cl | Olaparib 2245 |
| COc1cccc(c1)[C@H](C)OC(=O)c2cccc3C(=NNC(=O)c23)Cc4ccc(F)c(c4)C(=O)N5CCN(CC5)C(=O)C6CC6 | Olaparib 861 |
| Fc1ccc(CC2=NNC(=O)c3c(cccc23)C(=O)OCc4ccc5CCNC(=O)c5c4)cc1C(=O)N6CCN(CC6)C(=O)C7CC7 | Olaparib 1090 |
| C[C@H](CCNC(=O)c1cccc2C(=NNC(=O)c12)Cc3ccc(F)c(c3)C(=O)N4CCN(CC4)C(=O)C5CC5)C(=O)O | Olaparib 70 |
| CC(=O)N1CCC[C@@H]1c2cccc3C(=NNC(=O)c23)Cc4ccc(F)c(c4)C(=O)N5CCN(CC5)C(=O)C6CC6 | Olaparib 1996 |
| Fc1ccc(CC2=NNC(=O)c3c2cccc3c4ccccc4CSC5CCCC5)cc1C(=O)N6CCN(CC6)C(=O)C7CC7 | Olaparib 2054 |
| NC(=O)c1ccc(N)cc1Oc2cccc3C(=NNC(=O)c23)Cc4ccc(F)c(c4)C(=O)N5CCN(CC5)C(=O)C6CC6 | Olaparib 2383 |
| OC(=O)c1cc(Oc2cccc3C(=NNC(=O)c23)Cc4ccc(F)c(c4)C(=O)N5CCN(CC5)C(=O)C6CC6)c(Cl)cc1Cl | Olaparib 2244 |
| C[C@H](COC(=O)c1cccc2C(=NNC(=O)c12)Cc3ccc(F)c(c3)C(=O)N4CCN(CC4)C(=O)C5CC5)Nc6ccccc6 | Olaparib 876 |
| Oc1cc(Oc2cccc3C(=NNC(=O)c23)Cc4ccc(F)c(c4)C(=O)N5CCN(CC5)C(=O)C6CC6)cc7OC=CC(=O)c17 | Olaparib 3121 |
| COC(=O)CC(=O)[C@@H](C)Oc1cccc2C(=NNC(=O)c12)Cc3ccc(F)c(c3)C(=O)N4CCN(CC4)C(=O)C5CC5 | Olaparib 2150 |
| Fc1ccc(CC2=NNC(=O)c3c(NC(=O)CC4CC4)cccc23)cc1C(=O)N5CCN(CC5)C(=O)C6CC6 | Olaparib 521 |
| C[C@@H](CC#N)N(C)S(=O)(=O)c1cnc(Oc2cccc3C(=NNC(=O)c23)Cc4ccc(F)c(c4)C(=O)N5CCN(CC5)C(=O)C6CC6)nc1 | Olaparib 2174 |
| OC(=O)COc1ccc(Oc2cccc3C(=NNC(=O)c23)Cc4ccc(F)c(c4)C(=O)N5CCN(CC5)C(=O)C6CC6)cc1Cl | Olaparib 2260 |
| OC(=O)c1cccc(Oc2cccc3C(=NNC(=O)c23)Cc4ccc(F)c(c4)C(=O)N5CCN(CC5)C(=O)C6CC6)c1 | Olaparib 2403 |
| NCCCc1ccc(OC(=O)c2cccc3C(=NNC(=O)c23)Cc4ccc(F)c(c4)C(=O)N5CCN(CC5)C(=O)C6CC6)cc1 | Olaparib 871 |
| Fc1ccc(CC2=NNC(=O)c3c(Oc4ccc5NC(=O)C(=O)c5c4)cccc23)cc1C(=O)N6CCN(CC6)C(=O)C7CC7 | Olaparib 2614 |
| CC(C)(C(=O)O)C(=O)Oc1cccc2C(=NNC(=O)c12)Cc3ccc(F)c(c3)C(=O)N4CCN(CC4)C(=O)C5CC5 | Olaparib 1273 |
| Fc1ccc(CC2=NNC(=O)c3c(CCCN4CCOCC4)cccc23)cc1C(=O)N5CCN(CC5)C(=O)C6CC6 | Olaparib 1834 |
| COc1ccc(cc1)[C@@H](OC(=O)c2cccc3C(=NNC(=O)c23)Cc4ccc(F)c(c4)C(=O)N5CCN(CC5)C(=O)C6CC6)S(=O)(=O)[O-] | Olaparib 701 |
| Nc1cccnc1OC(=O)c2cccc3C(=NNC(=O)c23)Cc4ccc(F)c(c4)C(=O)N5CCN(CC5)C(=O)C6CC6 | Olaparib 979 |
| O\N=C(/Cl)\c1cccc(Oc2cccc3C(=NNC(=O)c23)Cc4ccc(F)c(c4)C(=O)N5CCN(CC5)C(=O)C6CC6)c1 | Olaparib 2337 |
| COc1ccccc1[C@@H](C)OC(=O)c2cccc3C(=NNC(=O)c23)Cc4ccc(F)c(c4)C(=O)N5CCN(CC5)C(=O)C6CC6 | Olaparib 867 |
| CC[C@]1(CCCNC1)C(=O)Nc2ccc(Oc3cccc4C(=NNC(=O)c34)Cc5ccc(F)c(c5)C(=O)N6CCN(CC6)C(=O)C7CC7)cc2 | Olaparib 2510 |
| C[C@H](CNC(=O)c1cccc2C(=NNC(=O)c12)Cc3ccc(F)c(c3)C(=O)N4CCN(CC4)C(=O)C5CC5)C(=O)O | Olaparib 107 |
| Fc1ccc(CC2=NNC(=O)c3c(CCCC4OCCO4)cccc23)cc1C(=O)N5CCN(CC5)C(=O)C6CC6 | Olaparib 1869 |
| C[C@@H](CC#N)N(C)S(=O)(=O)c1ccnc(Oc2cccc3C(=NNC(=O)c23)Cc4ccc(F)c(c4)C(=O)N5CCN(CC5)C(=O)C6CC6)c1 | Olaparib 2175 |
| CC(C)(C)Cc1cc(CNC(=O)c2cccc3C(=NNC(=O)c23)Cc4ccc(F)c(c4)C(=O)N5CCN(CC5)C(=O)C6CC6)on1 | Olaparib 167 |
| CC(C)(N)c1cc(Oc2cccc3C(=NNC(=O)c23)Cc4ccc(F)c(c4)C(=O)N5CCN(CC5)C(=O)C6CC6)c(N)cc1F | Olaparib 2343 |
| C[C@@H](O)c1ccccc1COC(=O)c2cccc3C(=NNC(=O)c23)Cc4ccc(F)c(c4)C(=O)N5CCN(CC5)C(=O)C6CC6 | Olaparib 858 |
| Cc1cc(ccc1Oc2cccc3C(=NNC(=O)c23)Cc4ccc(F)c(c4)C(=O)N5CCN(CC5)C(=O)C6CC6)C(=O)N[C@H]7CCCC[C@@H]7O | Olaparib 2504 |
| CC(=O)OCCNC(=O)c1cccc2C(=NNC(=O)c12)Cc3ccc(F)c(c3)C(=O)N4CCN(CC4)C(=O)C5CC5 | Olaparib 106 |
| Fc1cc(Br)cc(Oc2cccc3C(=NNC(=O)c23)Cc4ccc(F)c(c4)C(=O)N5CCN(CC5)C(=O)C6CC6)c1 | Olaparib 2329 |
| Cc1nn(CCS(=O)(=O)C)c(Oc2cccc3C(=NNC(=O)c23)Cc4ccc(F)c(c4)C(=O)N5CCN(CC5)C(=O)C6CC6)c1C=O | Olaparib 2204 |
| Cn1cncc1C(=O)Oc2cccc3C(=NNC(=O)c23)Cc4ccc(F)c(c4)C(=O)N5CCN(CC5)C(=O)C6CC6 | Olaparib 1517 |
| C[C@H](N)[C@H](O)c1ccc(Oc2cccc3C(=NNC(=O)c23)Cc4ccc(F)c(c4)C(=O)N5CCN(CC5)C(=O)C6CC6)cc1 | Olaparib 2925 |
| CC(=O)NCCc1ccc(Oc2cccc3C(=NNC(=O)c23)Cc4ccc(F)c(c4)C(=O)N5CCN(CC5)C(=O)C6CC6)cc1 | Olaparib 2898 |
| C[C@H](OC(=O)c1cccc2C(=NNC(=O)c12)Cc3ccc(F)c(c3)C(=O)N4CCN(CC4)C(=O)C5CC5)c6ccc(N7CCC(O)CC7)c(F)c6 | Olaparib 996 |
| CCOC(=O)CNC(=O)c1cccc2C(=NNC(=O)c12)Cc3ccc(F)c(c3)C(=O)N4CCN(CC4)C(=O)C5CC5 | Olaparib 104 |
| Fc1ccc(CC2=NNC(=O)c3c2cccc3c4ncccc4F)cc1C(=O)N5CCN(CC5)C(=O)C6CC6 | Olaparib 1914 |
| C[C@H]1CSCCN1S(=O)(=O)CC(=O)Oc2cccc3C(=NNC(=O)c23)Cc4ccc(F)c(c4)C(=O)N5CCN(CC5)C(=O)C6CC6 | Olaparib 1412 |
| Fc1ccc(CC2=NNC(=O)c3c(NC(=O)Cn4ccnc4)cccc23)cc1C(=O)N5CCN(CC5)C(=O)C6CC6 | Olaparib 517 |
| CC(C)(C#N)c1cc(Oc2cccc3C(=NNC(=O)c23)Cc4ccc(F)c(c4)C(=O)N5CCN(CC5)C(=O)C6CC6)ccc1N | Olaparib 2906 |
| CCc1ccc(CCOC(=O)c2cccc3C(=NNC(=O)c23)Cc4ccc(F)c(c4)C(=O)N5CCN(CC5)C(=O)C6CC6)nc1 | Olaparib 872 |
| C[C@@H](OC(=O)c1cccc2C(=NNC(=O)c12)Cc3ccc(F)c(c3)C(=O)N4CCN(CC4)C(=O)C5CC5)c6ccc(F)cc6OC7CCOCC7 | Olaparib 995 |
| COC(=O)CCNC(=O)c1cccc2C(=NNC(=O)c12)Cc3ccc(F)c(c3)C(=O)N4CCN(CC4)C(=O)C5CC5 | Olaparib 110 |
| Fc1ccc(CC2=NNC(=O)c3c(Cc4ccsc4)cccc23)cc1C(=O)N5CCN(CC5)C(=O)C6CC6 | Olaparib 2013 |
| OC(=O)[C@H]1CSCN1C(=O)NCCC(F)(F)Oc2cccc3C(=NNC(=O)c23)Cc4ccc(F)c(c4)C(=O)N5CCN(CC5)C(=O)C6CC6 | Olaparib 2176 |
| Fc1ccc(CC2=NNC(=O)c3c(OC(=O)Cn4ccnc4)cccc23)cc1C(=O)N5CCN(CC5)C(=O)C6CC6 | Olaparib 1516 |
| CC(C)(N)c1cc(Oc2cccc3C(=NNC(=O)c23)Cc4ccc(F)c(c4)C(=O)N5CCN(CC5)C(=O)C6CC6)cc(c1)C#N | Olaparib 2364 |
| CCCC(=O)Nc1ccc(Oc2cccc3C(=NNC(=O)c23)Cc4ccc(F)c(c4)C(=O)N5CCN(CC5)C(=O)C6CC6)cc1 | Olaparib 2897 |
| C[C@H](CN1C[C@@H](N)Cc2ccccc12)OC(=O)c3cccc4C(=NNC(=O)c34)Cc5ccc(F)c(c5)C(=O)N6CCN(CC6)C(=O)C7CC7 | Olaparib 1046 |
| CN(CC(=O)Oc1cccc2C(=NNC(=O)c12)Cc3ccc(F)c(c3)C(=O)N4CCN(CC4)C(=O)C5CC5)C(=O)C(Cl)(Cl)Cl | Olaparib 1172 |
| Fc1ccc(CC2=NNC(=O)c3c(cccc23)C4=CCOCC4)cc1C(=O)N5CCN(CC5)C(=O)C6CC6 | Olaparib 1908 |
| Cc1cnc(NC(=O)CC(=O)Oc2cccc3C(=NNC(=O)c23)Cc4ccc(F)c(c4)C(=O)N5CCN(CC5)C(=O)C6CC6)s1 | Olaparib 1454 |
| Fc1ccc(CC2=NNC(=O)c3c(OCCn4cncn4)cccc23)cc1C(=O)N5CCN(CC5)C(=O)C6CC6 | Olaparib 3042 |
| CC(C)(C)Sc1cc(Oc2cccc3C(=NNC(=O)c23)Cc4ccc(F)c(c4)C(=O)N5CCN(CC5)C(=O)C6CC6)ccn1 | Olaparib 2310 |
| Cc1ccccc1NCCOC(=O)c2cccc3C(=NNC(=O)c23)Cc4ccc(F)c(c4)C(=O)N5CCN(CC5)C(=O)C6CC6 | Olaparib 874 |
| Cc1cc(c(O)c(F)c1Oc2cccc3C(=NNC(=O)c23)Cc4ccc(F)c(c4)C(=O)N5CCN(CC5)C(=O)C6CC6)C7(CCC7)C(=O)O | Olaparib 2528 |
| CC(C)(Oc1cccc2C(=NNC(=O)c12)Cc3ccc(F)c(c3)C(=O)N4CCN(CC4)C(=O)C5CC5)[C@H](N)C(=O)O | Olaparib 2160 |
| CC(CN)(CN)CNC(=O)c1cccc2C(=NNC(=O)c12)Cc3ccc(F)c(c3)C(=O)N4CCN(CC4)C(=O)C5CC5 | Olaparib 56 |
| Fc1ccc(CC2=NNC(=O)c3c(cccc23)C4CCOCC4)cc1C(=O)N5CCN(CC5)C(=O)C6CC6 | Olaparib 1883 |
| COC(=O)COc1cnnc(Oc2cccc3C(=NNC(=O)c23)Cc4ccc(F)c(c4)C(=O)N5CCN(CC5)C(=O)C6CC6)c1Cl | Olaparib 2225 |
| NC(=O)C1=CC=C(NC1=O)C(F)(F)c2cccc3C(=NNC(=O)c23)Cc4ccc(F)c(c4)C(=O)N5CCN(CC5)C(=O)C6CC6 | Olaparib 1678 |
| C[C@@H](CN)Oc1cc(Oc2cccc3C(=NNC(=O)c23)Cc4ccc(F)c(c4)C(=O)N5CCN(CC5)C(=O)C6CC6)ccc1Cl | Olaparib 2185 |
| COc1cc(COC(=O)c2cccc3C(=NNC(=O)c23)Cc4ccc(F)c(c4)C(=O)N5CCN(CC5)C(=O)C6CC6)ccc1C | Olaparib 863 |
| Nc1ccc(c(COC(=O)c2cccc3C(=NNC(=O)c23)Cc4ccc(F)c(c4)C(=O)N5CCN(CC5)C(=O)C6CC6)c1)C7(CC7)C#N | Olaparib 1073 |
| COC(CNC(=O)c1cccc2C(=NNC(=O)c12)Cc3ccc(F)c(c3)C(=O)N4CCN(CC4)C(=O)C5CC5)OC | Olaparib 93 |
| Fc1ccc(CC2=NNC(=O)c3c(cccc23)[C@H]4CCNC4)cc1C(=O)N5CCN(CC5)C(=O)C6CC6 | Olaparib 2043 |
| CN(C)c1nc(Oc2cccc3C(=NNC(=O)c23)Cc4ccc(F)c(c4)C(=O)N5CCN(CC5)C(=O)C6CC6)nc(n1)N(C)C | Olaparib 2312 |
| C[C@@H]1CCCC[C@H]1NC(=O)\C(=C(/C)\C(=O)Oc2cccc3C(=NNC(=O)c23)Cc4ccc(F)c(c4)C(=O)N5CCN(CC5)C(=O)C6CC6)\C | Olaparib 1414 |
| CNC[C@H](O)c1cccc(Oc2cccc3C(=NNC(=O)c23)Cc4ccc(F)c(c4)C(=O)N5CCN(CC5)C(=O)C6CC6)c1 | Olaparib 2926 |
| Cc1cccc(NCCOC(=O)c2cccc3C(=NNC(=O)c23)Cc4ccc(F)c(c4)C(=O)N5CCN(CC5)C(=O)C6CC6)c1 | Olaparib 873 |
| Nc1ccc(cc1Oc2cccc3C(=NNC(=O)c23)Cc4ccc(F)c(c4)C(=O)N5CCN(CC5)C(=O)C6CC6)[C@@H]7CCC(=O)N7 | Olaparib 2604 |
| C[C@@H](O)C(=O)NCCOc1cccc2C(=NNC(=O)c12)Cc3ccc(F)c(c3)C(=O)N4CCN(CC4)C(=O)C5CC5 | Olaparib 2767 |
| Fc1ccc(CC2=NNC(=O)c3c(cccc23)[C@@H]4CCOC4)cc1C(=O)N5CCN(CC5)C(=O)C6CC6 | Olaparib 2042 |
| CN(c1nncs1)C(C)(C)C(=O)Oc2cccc3C(=NNC(=O)c23)Cc4ccc(F)c(c4)C(=O)N5CCN(CC5)C(=O)C6CC6 | Olaparib 1452 |
| Fc1ccc(CC2=NNC(=O)c3c(NC(=O)\C=C\C(=O)N4CCCCCCC4)cccc23)cc1C(=O)N5CCN(CC5)C(=O)C6CC6 | Olaparib 474 |
| OCCCc1ccc(O)c(Oc2cccc3C(=NNC(=O)c23)Cc4ccc(F)c(c4)C(=O)N5CCN(CC5)C(=O)C6CC6)c1 | Olaparib 2923 |
| C[C@@H](CSc1ccccc1)OC(=O)c2cccc3C(=NNC(=O)c23)Cc4ccc(F)c(c4)C(=O)N5CCN(CC5)C(=O)C6CC6 | Olaparib 796 |
| COc1cc(cc(Cl)c1OC(=O)c2cccc3C(=NNC(=O)c23)Cc4ccc(F)c(c4)C(=O)N5CCN(CC5)C(=O)C6CC6)C7(CC7)C#N | Olaparib 1019 |
| N[C@H]([C@H](O)C(F)F)C(=O)Oc1cccc2C(=NNC(=O)c12)Cc3ccc(F)c(c3)C(=O)N4CCN(CC4)C(=O)C5CC5 | Olaparib 1207 |
| Fc1cccc(F)c1Cc2cccc3C(=NNC(=O)c23)Cc4ccc(F)c(c4)C(=O)N5CCN(CC5)C(=O)C6CC6 | Olaparib 1866 |
| CSCCNc1cnnc(Oc2cccc3C(=NNC(=O)c23)Cc4ccc(F)c(c4)C(=O)N5CCN(CC5)C(=O)C6CC6)n1 | Olaparib 2296 |
| C[C@@H]1CCCCCN1C(=O)\C=C\C(=O)Oc2cccc3C(=NNC(=O)c23)Cc4ccc(F)c(c4)C(=O)N5CCN(CC5)C(=O)C6CC6 | Olaparib 1440 |
| CCCCOc1cc(Oc2cccc3C(=NNC(=O)c23)Cc4ccc(F)c(c4)C(=O)N5CCN(CC5)C(=O)C6CC6)ccn1 | Olaparib 2344 |
| COc1cccc(CCOC(=O)c2cccc3C(=NNC(=O)c23)Cc4ccc(F)c(c4)C(=O)N5CCN(CC5)C(=O)C6CC6)c1 | Olaparib 859 |
| NC\C=C\c1cc(Oc2cccc3C(=NNC(=O)c23)Cc4ccc(F)c(c4)C(=O)N5CCN(CC5)C(=O)C6CC6)c7OCOc7c1 | Olaparib 2584 |
| [15NH2][C@H](CO)C(=O)Oc1cccc2C(=NNC(=O)c12)Cc3ccc(F)c(c3)C(=O)N4CCN(CC4)C(=O)C5CC5 | Olaparib 1379 |
| Fc1cc(F)c(Cc2cccc3C(=NNC(=O)c23)Cc4ccc(F)c(c4)C(=O)N5CCN(CC5)C(=O)C6CC6)cc1F | Olaparib 1863 |
| C[C@H](N1C[C@H](COC(=O)c2cccc3C(=NNC(=O)c23)Cc4ccc(F)c(c4)C(=O)N5CCN(CC5)C(=O)C6CC6)CC1=O)C(=O)N(C)C | Olaparib 706 |
| Fc1ccc(CC2=NNC(=O)c3c(OC(=O)\C=C\C(=O)N4CCCCCCC4)cccc23)cc1C(=O)N5CCN(CC5)C(=O)C6CC6 | Olaparib 1439 |
| NC[C@H](O)c1ccc(Oc2cccc3C(=NNC(=O)c23)Cc4ccc(F)c(c4)C(=O)N5CCN(CC5)C(=O)C6CC6)cc1 | Olaparib 2405 |
| CCOc1ccc(COC(=O)c2cccc3C(=NNC(=O)c23)Cc4ccc(F)c(c4)C(=O)N5CCN(CC5)C(=O)C6CC6)cc1 | Olaparib 860 |
| Fc1ccc(CC2=NNC(=O)c3c(Oc4ccc5OC(=O)Nc5c4)cccc23)cc1C(=O)N6CCN(CC6)C(=O)C7CC7 | Olaparib 2619 |
| CCCC(CCC)c1cccc2C(=NNC(=O)c12)Cc3ccc(F)c(c3)C(=O)N4CCN(CC4)C(=O)C5CC5 | Olaparib 1904 |
| Fc1ccc(CC2=NNC(=O)c3c(\C=C\CC4CCCC4)cccc23)cc1C(=O)N5CCN(CC5)C(=O)C6CC6 | Olaparib 1999 |
| NC(=S)N1N=CC[C@@]1(OC(=O)c2cccc3C(=NNC(=O)c23)Cc4ccc(F)c(c4)C(=O)N5CCN(CC5)C(=O)C6CC6)C(F)(F)F | Olaparib 707 |
| CN1N=CC(=C(Cl)C1=O)Oc2cccc3C(=NNC(=O)c23)Cc4ccc(F)c(c4)C(=O)N5CCN(CC5)C(=O)C6CC6 | Olaparib 2947 |
| NCCOc1ccc(Oc2cccc3C(=NNC(=O)c23)Cc4ccc(F)c(c4)C(=O)N5CCN(CC5)C(=O)C6CC6)cc1Cl | Olaparib 2293 |
| CCc1nc(C)ccc1OC(=O)c2cccc3C(=NNC(=O)c23)Cc4ccc(F)c(c4)C(=O)N5CCN(CC5)C(=O)C6CC6 | Olaparib 928 |
| Fc1ccc(CC2=NNC(=O)c3c(\C=C\C4CCCC4)cccc23)cc1C(=O)N5CCN(CC5)C(=O)C6CC6 | Olaparib 2017 |
| COc1ccc(cc1OC)[C@@H]2C[C@@H](CN2)Oc3cccc4C(=NNC(=O)c34)Cc5ccc(F)c(c5)C(=O)N6CCN(CC6)C(=O)C7CC7 | Olaparib 3078 |
| C[C@@H]1CCN(C1)C(=O)CCC(=O)Nc2cccc3C(=NNC(=O)c23)Cc4ccc(F)c(c4)C(=O)N5CCN(CC5)C(=O)C6CC6 | Olaparib 493 |
| COCCOC1=C(Oc2cccc3C(=NNC(=O)c23)Cc4ccc(F)c(c4)C(=O)N5CCN(CC5)C(=O)C6CC6)C(=O)NN=C1 | Olaparib 2299 |
| OC[C@H](O)c1cccc(Oc2cccc3C(=NNC(=O)c23)Cc4ccc(F)c(c4)C(=O)N5CCN(CC5)C(=O)C6CC6)c1 | Olaparib 2376 |
| O[C@H](CCOC(=O)c1cccc2C(=NNC(=O)c12)Cc3ccc(F)c(c3)C(=O)N4CCN(CC4)C(=O)C5CC5)c6ccccc6 | Olaparib 870 |
| CC1(C)C[C@@]1(C)c2cccc3C(=NNC(=O)c23)Cc4ccc(F)c(c4)C(=O)N5CCN(CC5)C(=O)C6CC6 | Olaparib 2032 |
| CCC[C@H]1CCc2nc(COC(=O)c3cccc4C(=NNC(=O)c34)Cc5ccc(F)c(c5)C(=O)N6CCN(CC6)C(=O)C7CC7)[nH]c2C1 | Olaparib 1061 |
| OC(=O)[C@@H]1CC[C@@H](CNC(=O)c2cccc3C(=NNC(=O)c23)Cc4ccc(F)c(c4)C(=O)N5CCN(CC5)C(=O)C6CC6)CC1 | Olaparib 179 |
| Fc1ccc(CC2=NNC(=O)c3c(OC4(Cl)C(=O)NC(=O)NC4=O)cccc23)cc1C(=O)N5CCN(CC5)C(=O)C6CC6 | Olaparib 2320 |
| COc1cc(Oc2cccc3C(=NNC(=O)c23)Cc4ccc(F)c(c4)C(=O)N5CCN(CC5)C(=O)C6CC6)ccc1N | Olaparib 2401 |
| OCC[C@@H](OC(=O)c1cccc2C(=NNC(=O)c12)Cc3ccc(F)c(c3)C(=O)N4CCN(CC4)C(=O)C5CC5)c6ccccc6 | Olaparib 869 |
| Fc1ccc(CC2=NNC(=O)c3c2cccc3[C@@]4(F)CCC4(F)F)cc1C(=O)N5CCN(CC5)C(=O)C6CC6 | Olaparib 1729 |
| CC[C@H]1CCc2nc(COC(=O)c3cccc4C(=NNC(=O)c34)Cc5ccc(F)c(c5)C(=O)N6CCN(CC6)C(=O)C7CC7)[nH]c2C1 | Olaparib 1083 |
| Fc1ccc(CC2=NNC(=O)c3c(OC(=O)CON=C4CCCC4)cccc23)cc1C(=O)N5CCN(CC5)C(=O)C6CC6 | Olaparib 1499 |
| C[C@H](N(C)C(=O)NC1CCOCC1)C(=O)Oc2cccc3C(=NNC(=O)c23)Cc4ccc(F)c(c4)C(=O)N5CCN(CC5)C(=O)C6CC6 | Olaparib 1419 |
| COc1ccc(N)c(Oc2cccc3C(=NNC(=O)c23)Cc4ccc(F)c(c4)C(=O)N5CCN(CC5)C(=O)C6CC6)c1 | Olaparib 2400 |
| O[C@H](COC(=O)c1cccc2C(=NNC(=O)c12)Cc3ccc(F)c(c3)C(=O)N4CCN(CC4)C(=O)C5CC5)Cc6ccccc6 | Olaparib 866 |
| Fc1ccc(CC2=NNC(=O)c3c2cccc3[C@]4(Cl)CCC4(F)F)cc1C(=O)N5CCN(CC5)C(=O)C6CC6 | Olaparib 1728 |
| C[C@@H]1CC[C@@H]([C@H]1C)N2C[C@H](COC(=O)c3cccc4C(=NNC(=O)c34)Cc5ccc(F)c(c5)C(=O)N6CCN(CC6)C(=O)C7CC7)CC2=O | Olaparib 1039 |
| Fc1ccc(CC2=NNC(=O)c3c(cccc23)[C@@H]4CNC(=O)NC4=O)cc1C(=O)N5CCN(CC5)C(=O)C6CC6 | Olaparib 1994 |
| C[C@@H](NC(=O)N1CCSC[C@@H]1C)C(=O)Nc2cccc3C(=NNC(=O)c23)Cc4ccc(F)c(c4)C(=O)N5CCN(CC5)C(=O)C6CC6 | Olaparib 462 |
| Nc1ccc(cc1Oc2cccc3C(=NNC(=O)c23)Cc4ccc(F)c(c4)C(=O)N5CCN(CC5)C(=O)C6CC6)C#CCO | Olaparib 2394 |
| OC[C@@H](Cc1ccccc1)OC(=O)c2cccc3C(=NNC(=O)c23)Cc4ccc(F)c(c4)C(=O)N5CCN(CC5)C(=O)C6CC6 | Olaparib 865 |
| Fc1ccc(CC2=NNC(=O)c3c(cccc23)C(=O)NC(=S)NCC=C)cc1C(=O)N4CCN(CC4)C(=O)C5CC5 | Olaparib 75 |
| C[C@@H](C1CCCC1)N2C[C@H](COC(=O)c3cccc4C(=NNC(=O)c34)Cc5ccc(F)c(c5)C(=O)N6CCN(CC6)C(=O)C7CC7)CC2=O | Olaparib 1038 |
| O[C@@H]1OC(=O)C=C1Oc2cccc3C(=NNC(=O)c23)Cc4ccc(F)c(c4)C(=O)N5CCN(CC5)C(=O)C6CC6 | Olaparib 2425 |
| NC(=S)N1N=CC[C@@]1(O)C(F)(F)Oc2cccc3C(=NNC(=O)c23)Cc4ccc(F)c(c4)C(=O)N5CCN(CC5)C(=O)C6CC6 | Olaparib 2274 |
| N[C@@H](COc1cccc2C(=NNC(=O)c12)Cc3ccc(F)c(c3)C(=O)N4CCN(CC4)C(=O)C5CC5)c6ccccn6 | Olaparib 2999 |
| CCC(=O)Nc1ccc(Oc2cccc3C(=NNC(=O)c23)Cc4ccc(F)c(c4)C(=O)N5CCN(CC5)C(=O)C6CC6)cc1 | Olaparib 2936 |
| CC[C@H](C)[C@H](N)C(=S)NC(=O)c1cccc2C(=NNC(=O)c12)Cc3ccc(F)c(c3)C(=O)N4CCN(CC4)C(=O)C5CC5 | Olaparib 3 |
| Cc1cc(ccc1Oc2cccc3C(=NNC(=O)c23)Cc4ccc(F)c(c4)C(=O)N5CCN(CC5)C(=O)C6CC6)C(=O)NCc7ccccc7Cl | Olaparib 2437 |
| C[C@@H](C(=O)Nc1cccc2C(=NNC(=O)c12)Cc3ccc(F)c(c3)C(=O)N4CCN(CC4)C(=O)C5CC5)[C@@]6(O)CCOC(C)(C)C6 | Olaparib 480 |
| N[C@@H]([C@@H]1CC(=NO1)Oc2cccc3C(=NNC(=O)c23)Cc4ccc(F)c(c4)C(=O)N5CCN(CC5)C(=O)C6CC6)C(=O)O | Olaparib 2363 |
| NNCc1cccc(Oc2cccc3C(=NNC(=O)c23)Cc4ccc(F)c(c4)C(=O)N5CCN(CC5)C(=O)C6CC6)c1 | Olaparib 2998 |
| CCC(=O)c1cc(Oc2cccc3C(=NNC(=O)c23)Cc4ccc(F)c(c4)C(=O)N5CCN(CC5)C(=O)C6CC6)ccc1O | Olaparib 2933 |
| COCC(=S)NC(=O)c1cccc2C(=NNC(=O)c12)Cc3ccc(F)c(c3)C(=O)N4CCN(CC4)C(=O)C5CC5 | Olaparib 91 |
| Cc1ccc(OCc2cc(Oc3cccc4C(=NNC(=O)c34)Cc5ccc(F)c(c5)C(=O)N6CCN(CC6)C(=O)C7CC7)ccc2F)c(CN)c1 | Olaparib 2475 |
| CC(C)(C#N)N1CCN(CC1)C(=O)CC(F)(F)c2cccc3C(=NNC(=O)c23)Cc4ccc(F)c(c4)C(=O)N5CCN(CC5)C(=O)C6CC6 | Olaparib 1649 |
| NCc1ccc(Oc2cccc3C(=NNC(=O)c23)Cc4ccc(F)c(c4)C(=O)N5CCN(CC5)C(=O)C6CC6)c(O)c1 | Olaparib 2997 |
| C[C@H](C(=O)Oc1cccc2C(=NNC(=O)c12)Cc3ccc(F)c(c3)C(=O)N4CCN(CC4)C(=O)C5CC5)S(=O)(=O)c6ccccc6 | Olaparib 1435 |
| OCP(COC(=O)c1cccc2C(=NNC(=O)c12)Cc3ccc(F)c(c3)C(=O)N4CCN(CC4)C(=O)C5CC5)c6ccccc6 | Olaparib 794 |
| NS(=O)(=O)C(F)(F)c1cccc2C(=NNC(=O)c12)Cc3ccc(F)c(c3)C(=O)N4CCN(CC4)C(=O)C5CC5 | Olaparib 1626 |
| CC1(C)CCCN(CC(=O)c2ccc(Oc3cccc4C(=NNC(=O)c34)Cc5ccc(F)c(c5)C(=O)N6CCN(CC6)C(=O)C7CC7)cc2F)C1 | Olaparib 2467 |
| CC[C@H](N1CCCO[C@@H](C)C1)C(=O)Oc2cccc3C(=NNC(=O)c23)Cc4ccc(F)c(c4)C(=O)N5CCN(CC5)C(=O)C6CC6 | Olaparib 1451 |
| COc1ccc(Oc2cccc3C(=NNC(=O)c23)Cc4ccc(F)c(c4)C(=O)N5CCN(CC5)C(=O)C6CC6)c(N)c1 | Olaparib 2995 |
| C[C@@H](NC(=O)Nc1ccccc1C)C(=O)Nc2cccc3C(=NNC(=O)c23)Cc4ccc(F)c(c4)C(=O)N5CCN(CC5)C(=O)C6CC6 | Olaparib 466 |
| N[C@@H](C(=O)Oc1cccc2C(=NNC(=O)c12)Cc3ccc(F)c(c3)C(=O)N4CCN(CC4)C(=O)C5CC5)c6ccc(C(F)F)c(F)c6 | Olaparib 1428 |
| Fc1ccc(CC2=NNC(=O)c3c(cccc23)C(=O)NC(=S)CC#N)cc1C(=O)N4CCN(CC4)C(=O)C5CC5 | Olaparib 124 |
| C[C@](O)(C#Cc1ccccc1)c2ccc(Oc3cccc4C(=NNC(=O)c34)Cc5ccc(F)c(c5)C(=O)N6CCN(CC6)C(=O)C7CC7)cc2 | Olaparib 2529 |
| C[C@H]1CC[C@H](CN)CN1CC(=O)Oc2cccc3C(=NNC(=O)c23)Cc4ccc(F)c(c4)C(=O)N5CCN(CC5)C(=O)C6CC6 | Olaparib 1468 |
| Nc1c(O)c(Oc2cccc3C(=NNC(=O)c23)Cc4ccc(F)c(c4)C(=O)N5CCN(CC5)C(=O)C6CC6)cc(Cl)c1Cl | Olaparib 2278 |
| COCc1ccnc(c1)C(=N)NC(=O)c2cccc3C(=NNC(=O)c23)Cc4ccc(F)c(c4)C(=O)N5CCN(CC5)C(=O)C6CC6 | Olaparib 171 |
| C[C@H](OC(=O)c1cccc2C(=NNC(=O)c12)Cc3ccc(F)c(c3)C(=O)N4CCN(CC4)C(=O)C5CC5)c6ccc(O)cc6 | Olaparib 925 |
| CSCC[C@H](NC(=O)c1cccc2C(=NNC(=O)c12)Cc3ccc(F)c(c3)C(=O)N4CCN(CC4)C(=O)C5CC5)C#N | Olaparib 32 |
| Fc1ccc(CC2=NNC(=O)c3c(cccc23)C(=O)OCCCc4ccc5ccccc5c4)cc1C(=O)N6CCN(CC6)C(=O)C7CC7 | Olaparib 1078 |
| CC1=NN[C@](O)(C1)C(F)(F)Oc2cccc3C(=NNC(=O)c23)Cc4ccc(F)c(c4)C(=O)N5CCN(CC5)C(=O)C6CC6 | Olaparib 2393 |
| Nc1cc(Oc2cccc3C(=NNC(=O)c23)Cc4ccc(F)c(c4)C(=O)N5CCN(CC5)C(=O)C6CC6)cc(c1)C#N | Olaparib 2415 |
| Fc1ccc(CC2=NNC(=O)c3c(cccc23)C(=O)Oc4ncccc4C(=O)NCCC#C)cc1C(=O)N5CCN(CC5)C(=O)C6CC6 | Olaparib 736 |
| C[C@H](O)c1ccc(OC(=O)c2cccc3C(=NNC(=O)c23)Cc4ccc(F)c(c4)C(=O)N5CCN(CC5)C(=O)C6CC6)cc1 | Olaparib 926 |
| OC(=O)CCSC(F)(F)Oc1cccc2C(=NNC(=O)c12)Cc3ccc(F)c(c3)C(=O)N4CCN(CC4)C(=O)C5CC5 | Olaparib 2128 |
| Fc1ccc(CC2=NNC(=O)c3c(Cc4ccc(cc4)c5ccccc5)cccc23)cc1C(=O)N6CCN(CC6)C(=O)C7CC7 | Olaparib 1890 |
| CC(C)(Nc1ccc(cc1C(=O)O)C(F)(F)c2cccc3C(=NNC(=O)c23)Cc4ccc(F)c(c4)C(=O)N5CCN(CC5)C(=O)C6CC6)C#C | Olaparib 1647 |
| COc1cc(ncn1)c2cccc3C(=NNC(=O)c23)Cc4ccc(F)c(c4)C(=O)N5CCN(CC5)C(=O)C6CC6 | Olaparib 1726 |
| Fc1ccc(CC2=NNC(=O)c3c(cccc23)C(=O)NC(=S)NNc4ccccc4)cc1C(=O)N5CCN(CC5)C(=O)C6CC6 | Olaparib 168 |
| Nc1ccccc1CCOC(=O)c2cccc3C(=NNC(=O)c23)Cc4ccc(F)c(c4)C(=O)N5CCN(CC5)C(=O)C6CC6 | Olaparib 929 |
| CCOC(=O)\C=C\C(=C)Oc1cccc2C(=NNC(=O)c12)Cc3ccc(F)c(c3)C(=O)N4CCN(CC4)C(=O)C5CC5 | Olaparib 2140 |
| Fc1ccc(CC2=NNC(=O)c3c(Cc4ccc5ccccc5c4)cccc23)cc1C(=O)N6CCN(CC6)C(=O)C7CC7 | Olaparib 1887 |
| CC(C)(C(=O)O)c1ccc(Oc2cccc3C(=NNC(=O)c23)Cc4ccc(F)c(c4)C(=O)N5CCN(CC5)C(=O)C6CC6)c(Cl)c1F | Olaparib 2228 |
| COc1nccnc1c2cccc3C(=NNC(=O)c23)Cc4ccc(F)c(c4)C(=O)N5CCN(CC5)C(=O)C6CC6 | Olaparib 1915 |
| COC[C@@H](OC(=O)c1cccc2C(=NNC(=O)c12)Cc3ccc(F)c(c3)C(=O)N4CCN(CC4)C(=O)C5CC5)c6c(C)ccnc6N | Olaparib 759 |
| NC[C@H](OC(=O)c1cccc2C(=NNC(=O)c12)Cc3ccc(F)c(c3)C(=O)N4CCN(CC4)C(=O)C5CC5)c6ccccc6 | Olaparib 930 |
| OC(=O)\C(=C(/Cl)\C=O)\c1cccc2C(=NNC(=O)c12)Cc3ccc(F)c(c3)C(=O)N4CCN(CC4)C(=O)C5CC5 | Olaparib 1611 |
| CC(C)(NC(=O)c1cccc2C(=NNC(=O)c12)Cc3ccc(F)c(c3)C(=O)N4CCN(CC4)C(=O)C5CC5)c6noc(n6)C(C)(C)S(=O)(=O)C | Olaparib 126 |
| C[C@H](Oc1cccc2C(=NNC(=O)c12)Cc3ccc(F)c(c3)C(=O)N4CCN(CC4)C(=O)C5CC5)[C]6[CH][CH][CH][CH]6 | Olaparib 3048 |
| CSCCNC(=O)c1cc(Oc2cccc3C(=NNC(=O)c23)Cc4ccc(F)c(c4)C(=O)N5CCN(CC5)C(=O)C6CC6)ccc1N | Olaparib 2238 |
| Nc1cccnc1Oc2cccc3C(=NNC(=O)c23)Cc4ccc(F)c(c4)C(=O)N5CCN(CC5)C(=O)C6CC6 | Olaparib 3047 |
| Oc1cccc(CCOC(=O)c2cccc3C(=NNC(=O)c23)Cc4ccc(F)c(c4)C(=O)N5CCN(CC5)C(=O)C6CC6)c1 | Olaparib 923 |
| C[C@](N)(CC=C)C(=O)Oc1cccc2C(=NNC(=O)c12)Cc3ccc(F)c(c3)C(=O)N4CCN(CC4)C(=O)C5CC5 | Olaparib 1300 |
| COc1ccc(OC(=O)c2cccc3C(=NNC(=O)c23)Cc4ccc(F)c(c4)C(=O)N5CCN(CC5)C(=O)C6CC6)c(c1)C(=O)NC(=O)N | Olaparib 710 |
| CN(C)C(=O)[C@H](O)[C@H](OC(=O)c1cccc2C(=NNC(=O)c12)Cc3ccc(F)c(c3)C(=O)N4CCN(CC4)C(=O)C5CC5)C(=O)N(C)C | Olaparib 560 |
| COc1cc(COC(=O)c2cccc3C(=NNC(=O)c23)Cc4ccc(F)c(c4)C(=O)N5CCN(CC5)C(=O)C6CC6)cc(OC)c1O | Olaparib 751 |
| [O-][n+]1ccncc1c2cccc3C(=NNC(=O)c23)Cc4ccc(F)c(c4)C(=O)N5CCN(CC5)C(=O)C6CC6 | Olaparib 1732 |
| Cc1ccc(N)cc1OC(=O)c2cccc3C(=NNC(=O)c23)Cc4ccc(F)c(c4)C(=O)N5CCN(CC5)C(=O)C6CC6 | Olaparib 961 |
| CC(C)CC(=O)C(=O)Nc1cccc2C(=NNC(=O)c12)Cc3ccc(F)c(c3)C(=O)N4CCN(CC4)C(=O)C5CC5 | Olaparib 362 |
| Cc1nn(CCS(=O)(=O)C)c(Oc2cccc3C(=NNC(=O)c23)Cc4ccc(F)c(c4)C(=O)N5CCN(CC5)C(=O)C6CC6)c1CO | Olaparib 2198 |
| CC(C)(NC(=O)NC(=O)\C=C\C(=O)Oc1cccc2C(=NNC(=O)c12)Cc3ccc(F)c(c3)C(=O)N4CCN(CC4)C(=O)C5CC5)C#C | Olaparib 1173 |
| COc1cc(CO)cc(OC)c1OC(=O)c2cccc3C(=NNC(=O)c23)Cc4ccc(F)c(c4)C(=O)N5CCN(CC5)C(=O)C6CC6 | Olaparib 750 |
| [O-][n+]1ccnc(c1)c2cccc3C(=NNC(=O)c23)Cc4ccc(F)c(c4)C(=O)N5CCN(CC5)C(=O)C6CC6 | Olaparib 1731 |
| OCCc1cccc(OC(=O)c2cccc3C(=NNC(=O)c23)Cc4ccc(F)c(c4)C(=O)N5CCN(CC5)C(=O)C6CC6)c1 | Olaparib 922 |
| CC(=C[C@@H](O)C(=O)Nc1cccc2C(=NNC(=O)c12)Cc3ccc(F)c(c3)C(=O)N4CCN(CC4)C(=O)C5CC5)C | Olaparib 361 |
| Cc1nn(C)c(Oc2cccc3C(=NNC(=O)c23)Cc4ccc(F)c(c4)C(=O)N5CCN(CC5)C(=O)C6CC6)c1S(=O)(=O)N | Olaparib 2284 |
| OS(=O)(=O)CCCNC(=O)c1cccc2C(=NNC(=O)c12)Cc3ccc(F)c(c3)C(=O)N4CCN(CC4)C(=O)C5CC5 | Olaparib 11 |
| Nc1cc(Oc2cccc3C(=NNC(=O)c23)Cc4ccc(F)c(c4)C(=O)N5CCN(CC5)C(=O)C6CC6)ccc1OCC(=O)O | Olaparib 2314 |
| Fc1ccc(CC2=NNC(=O)c3c(OC(=O)c4sccc4Br)cccc23)cc1C(=O)N5CCN(CC5)C(=O)C6CC6 | Olaparib 1443 |
| C[C@H](OC(=O)c1cccc2C(=NNC(=O)c12)Cc3ccc(F)c(c3)C(=O)N4CCN(CC4)C(=O)C5CC5)c6ccc(cc6)C#N | Olaparib 886 |
| CCOC(=O)[C@H](C(=O)C)c1cccc2C(=NNC(=O)c12)Cc3ccc(F)c(c3)C(=O)N4CCN(CC4)C(=O)C5CC5 | Olaparib 1627 |
| Nn1nnnc1NC(=O)c2cccc3C(=NNC(=O)c23)Cc4ccc(F)c(c4)C(=O)N5CCN(CC5)C(=O)C6CC6 | Olaparib 199 |
| NC(=O)NC(=O)CCC(=O)Oc1cccc2C(=NNC(=O)c12)Cc3ccc(F)c(c3)C(=O)N4CCN(CC4)C(=O)C5CC5 | Olaparib 1198 |
| C[C@H](Sc1ccc(cn1)C#N)C(=O)Oc2cccc3C(=NNC(=O)c23)Cc4ccc(F)c(c4)C(=O)N5CCN(CC5)C(=O)C6CC6 | Olaparib 1442 |
| Fc1ccc(CC2=NNC(=O)c3c(OCCc4cn[nH]c4)cccc23)cc1C(=O)N5CCN(CC5)C(=O)C6CC6 | Olaparib 3045 |
| COCC(=O)c1ccc(Oc2cccc3C(=NNC(=O)c23)Cc4ccc(F)c(c4)C(=O)N5CCN(CC5)C(=O)C6CC6)cc1 | Olaparib 2392 |
| CCC(=O)[C@@H](C(=O)OC)c1cccc2C(=NNC(=O)c12)Cc3ccc(F)c(c3)C(=O)N4CCN(CC4)C(=O)C5CC5 | Olaparib 1629 |
| CC(C)(C(=O)N1CC[C@H](C1)OC(=O)c2cccc3C(=NNC(=O)c23)Cc4ccc(F)c(c4)C(=O)N5CCN(CC5)C(=O)C6CC6)S(=O)(=O)C | Olaparib 690 |
| COC(=O)N[C@@H](O)C(=O)Oc1cccc2C(=NNC(=O)c12)Cc3ccc(F)c(c3)C(=O)N4CCN(CC4)C(=O)C5CC5 | Olaparib 1210 |
| COC(=O)COc1cc(Oc2cccc3C(=NNC(=O)c23)Cc4ccc(F)c(c4)C(=O)N5CCN(CC5)C(=O)C6CC6)ccn1 | Olaparib 2313 |
| Fc1ccc(CC2=NNC(=O)c3c2cccc3C4=CC=NNC4=O)cc1C(=O)N5CCN(CC5)C(=O)C6CC6 | Olaparib 2022 |
| CSCC(=O)c1ccc(Oc2cccc3C(=NNC(=O)c23)Cc4ccc(F)c(c4)C(=O)N5CCN(CC5)C(=O)C6CC6)cc1F | Olaparib 2308 |
| COC(=O)CC(=O)[C@@H](C)c1cccc2C(=NNC(=O)c12)Cc3ccc(F)c(c3)C(=O)N4CCN(CC4)C(=O)C5CC5 | Olaparib 1628 |
| CS(=O)(=O)CC(=O)N1CC[C@@H](COC(=O)c2cccc3C(=NNC(=O)c23)Cc4ccc(F)c(c4)C(=O)N5CCN(CC5)C(=O)C6CC6)C1 | Olaparib 697 |
| CS(=O)(=O)CCCNC(=O)c1cccc2C(=NNC(=O)c12)Cc3ccc(F)c(c3)C(=O)N4CCN(CC4)C(=O)C5CC5 | Olaparib 14 |
| Cc1nc(CCC(=O)Oc2cccc3C(=NNC(=O)c23)Cc4ccc(F)c(c4)C(=O)N5CCN(CC5)C(=O)C6CC6)n(C)n1 | Olaparib 1485 |
| C[C@H]1CC[C@H](CNC(=O)c2cccc3C(=NNC(=O)c23)Cc4ccc(F)c(c4)C(=O)N5CCN(CC5)C(=O)C6CC6)CN1CC=C(C)C | Olaparib 151 |
| COc1ccc(Br)cc1COC(=O)c2cccc3C(=NNC(=O)c23)Cc4ccc(F)c(c4)C(=O)N5CCN(CC5)C(=O)C6CC6 | Olaparib 702 |
| CC(=C[C@@H](O)C(=O)Oc1cccc2C(=NNC(=O)c12)Cc3ccc(F)c(c3)C(=O)N4CCN(CC4)C(=O)C5CC5)C | Olaparib 1294 |
| CC1=C(CCOC(=O)c2cccc3C(=NNC(=O)c23)Cc4ccc(F)c(c4)C(=O)N5CCN(CC5)C(=O)C6CC6)C(=O)N(N1)C(=N)N | Olaparib 749 |
| CCOC(=O)\C=C(/N)\NC(=O)c1cccc2C(=NNC(=O)c12)Cc3ccc(F)c(c3)C(=O)N4CCN(CC4)C(=O)C5CC5 | Olaparib 33 |
| CSCCNc1cncc(Oc2cccc3C(=NNC(=O)c23)Cc4ccc(F)c(c4)C(=O)N5CCN(CC5)C(=O)C6CC6)n1 | Olaparib 2302 |
| C\C(=C/C(=O)Nc1cccc2C(=NNC(=O)c12)Cc3ccc(F)c(c3)C(=O)N4CCN(CC4)C(=O)C5CC5)\CN6CCCC(C)(C)C6 | Olaparib 473 |
| NC(=N)Cc1ccc(Oc2cccc3C(=NNC(=O)c23)Cc4ccc(F)c(c4)C(=O)N5CCN(CC5)C(=O)C6CC6)cc1 | Olaparib 2275 |
| CN(C)\N=C\C(=O)C(F)(F)c1cccc2C(=NNC(=O)c12)Cc3ccc(F)c(c3)C(=O)N4CCN(CC4)C(=O)C5CC5 | Olaparib 1613 |
| Fc1ccc(CC2=NNC(=O)c3c(cccc23)C(=O)OC4C(=O)NC(=O)NC4=O)cc1C(=O)N5CCN(CC5)C(=O)C6CC6 | Olaparib 906 |
| CON(C)C(=O)\C=C\C(=O)Oc1cccc2C(=NNC(=O)c12)Cc3ccc(F)c(c3)C(=O)N4CCN(CC4)C(=O)C5CC5 | Olaparib 1201 |
| CSCCNc1ccnc(Oc2cccc3C(=NNC(=O)c23)Cc4ccc(F)c(c4)C(=O)N5CCN(CC5)C(=O)C6CC6)n1 | Olaparib 2303 |
| NC[C@@H](C(=O)Nc1cccc2C(=NNC(=O)c12)Cc3ccc(F)c(c3)C(=O)N4CCN(CC4)C(=O)C5CC5)C6=CCCCC6 | Olaparib 499 |
| CN\N=C\c1ccc(Oc2cccc3C(=NNC(=O)c23)Cc4ccc(F)c(c4)C(=O)N5CCN(CC5)C(=O)C6CC6)cc1 | Olaparib 2389 |
| N[C@H](CC=C)C(=O)Nc1cccc2C(=NNC(=O)c12)Cc3ccc(F)c(c3)C(=O)N4CCN(CC4)C(=O)C5CC5 | Olaparib 410 |
| Fc1ccc(CC2=NNC(=O)c3c(cccc23)C(=O)OCCN[C@]4(CCCS(=O)(=O)C4)C#N)cc1C(=O)N5CCN(CC5)C(=O)C6CC6 | Olaparib 700 |
| CC\C=N\NC(=S)NC(=O)c1cccc2C(=NNC(=O)c12)Cc3ccc(F)c(c3)C(=O)N4CCN(CC4)C(=O)C5CC5 | Olaparib 26 |
| CC(C)c1nnc(OC(=O)c2cccc3C(=NNC(=O)c23)Cc4ccc(F)c(c4)C(=O)N5CCN(CC5)C(=O)C6CC6)s1 | Olaparib 902 |
| NC[C@@H](C(=O)Oc1cccc2C(=NNC(=O)c12)Cc3ccc(F)c(c3)C(=O)N4CCN(CC4)C(=O)C5CC5)C6=CCCCC6 | Olaparib 1484 |
| Fc1ccc(CC2=NNC(=O)c3c(cccc23)C(=O)OCCOc4ccccc4)cc1C(=O)N5CCN(CC5)C(=O)C6CC6 | Olaparib 924 |
| C[C@](N)(C=C)C(=O)Oc1cccc2C(=NNC(=O)c12)Cc3ccc(F)c(c3)C(=O)N4CCN(CC4)C(=O)C5CC5 | Olaparib 1351 |
| OCCOCN1C=C(Oc2cccc3C(=NNC(=O)c23)Cc4ccc(F)c(c4)C(=O)N5CCN(CC5)C(=O)C6CC6)C(=O)NC1=O | Olaparib 2301 |
| C[C@H](N)C(=O)N[C@@H](C)C(=O)Nc1cccc2C(=NNC(=O)c12)Cc3ccc(F)c(c3)C(=O)N4CCN(CC4)C(=O)C5CC5 | Olaparib 286 |
| Oc1nc(OC(=O)c2cccc3C(=NNC(=O)c23)Cc4ccc(F)c(c4)C(=O)N5CCN(CC5)C(=O)C6CC6)sc1Br | Olaparib 727 |
| CC[C@@H]1OC(=C(Oc2cccc3C(=NNC(=O)c23)Cc4ccc(F)c(c4)C(=O)N5CCN(CC5)C(=O)C6CC6)C1=O)C | Olaparib 2993 |
| NC(=O)Cc1ccc(Oc2cccc3C(=NNC(=O)c23)Cc4ccc(F)c(c4)C(=O)N5CCN(CC5)C(=O)C6CC6)cc1 | Olaparib 2972 |
| N[C@H](CC=C)C(=O)Oc1cccc2C(=NNC(=O)c12)Cc3ccc(F)c(c3)C(=O)N4CCN(CC4)C(=O)C5CC5 | Olaparib 1350 |
| NC(=S)N\N=C\c1ccc(OC(=O)c2cccc3C(=NNC(=O)c23)Cc4ccc(F)c(c4)C(=O)N5CCN(CC5)C(=O)C6CC6)cc1 | Olaparib 729 |
| C[C@H](N)C(=O)N[C@@H](C)C(=O)Oc1cccc2C(=NNC(=O)c12)Cc3ccc(F)c(c3)C(=O)N4CCN(CC4)C(=O)C5CC5 | Olaparib 1196 |
| Fc1ccc(CC2=NNC(=O)c3c(Oc4ncn(CC#N)n4)cccc23)cc1C(=O)N5CCN(CC5)C(=O)C6CC6 | Olaparib 2423 |
| Fc1ccc(CC2=NNC(=O)c3c(OC(F)(F)C(=O)N4CC=CC4)cccc23)cc1C(=O)N5CCN(CC5)C(=O)C6CC6 | Olaparib 2395 |
| Fc1ccc(CC2=NNC(=O)c3c(cccc23)C(=O)OCCOc4c(F)c(F)c(F)c(F)c4F)cc1C(=O)N5CCN(CC5)C(=O)C6CC6 | Olaparib 691 |
| N[C@H](C=C)C(=O)Nc1cccc2C(=NNC(=O)c12)Cc3ccc(F)c(c3)C(=O)N4CCN(CC4)C(=O)C5CC5 | Olaparib 449 |
| NC(=O)CCNC(=O)c1ccc(Oc2cccc3C(=NNC(=O)c23)Cc4ccc(F)c(c4)C(=O)N5CCN(CC5)C(=O)C6CC6)nc1 | Olaparib 2281 |
| CC[C@H](O)[C@H](NC(=O)c1cccc2C(=NNC(=O)c12)Cc3ccc(F)c(c3)C(=O)N4CCN(CC4)C(=O)C5CC5)C(=O)O | Olaparib 23 |
| CCN1C(=O)C=C(C)C(=C1OC(=O)c2cccc3C(=NNC(=O)c23)Cc4ccc(F)c(c4)C(=O)N5CCN(CC5)C(=O)C6CC6)C#N | Olaparib 772 |
| Fc1ccc(CC2=NNC(=O)c3c(O[C@H]4OCC(=O)C=C4)cccc23)cc1C(=O)N5CCN(CC5)C(=O)C6CC6 | Olaparib 3039 |
| CC(=O)c1ccc(Oc2cccc3C(=NNC(=O)c23)Cc4ccc(F)c(c4)C(=O)N5CCN(CC5)C(=O)C6CC6)cc1O | Olaparib 2270 |
| N[C@H](C=C)C(=O)Oc1cccc2C(=NNC(=O)c12)Cc3ccc(F)c(c3)C(=O)N4CCN(CC4)C(=O)C5CC5 | Olaparib 1396 |
| Cc1cnc(NC(=O)CC(=O)Nc2cccc3C(=NNC(=O)c23)Cc4ccc(F)c(c4)C(=O)N5CCN(CC5)C(=O)C6CC6)s1 | Olaparib 482 |
| C[C@H](NC(=O)CN)C(=O)Nc1cccc2C(=NNC(=O)c12)Cc3ccc(F)c(c3)C(=O)N4CCN(CC4)C(=O)C5CC5 | Olaparib 299 |
| Fc1ccc(CC2=NNC(=O)c3c(OC(=O)CCN4C=CC=NC4=O)cccc23)cc1C(=O)N5CCN(CC5)C(=O)C6CC6 | Olaparib 1487 |
| O[C@H]1OC(=O)C(=C1c2cccc3C(=NNC(=O)c23)Cc4ccc(F)c(c4)C(=O)N5CCN(CC5)C(=O)C6CC6)Cl | Olaparib 1712 |
| Nc1ccc(cc1F)C#CCOC(=O)c2cccc3C(=NNC(=O)c23)Cc4ccc(F)c(c4)C(=O)N5CCN(CC5)C(=O)C6CC6 | Olaparib 820 |
| C[C@@H](F)C(=O)C(=O)Nc1cccc2C(=NNC(=O)c12)Cc3ccc(F)c(c3)C(=O)N4CCN(CC4)C(=O)C5CC5 | Olaparib 386 |
| Nc1ncc(COC(=O)c2cccc3C(=NNC(=O)c23)Cc4ccc(F)c(c4)C(=O)N5CCN(CC5)C(=O)C6CC6)c(N)n1 | Olaparib 917 |
| CNC(=O)N(C)CC(=O)Nc1cccc2C(=NNC(=O)c12)Cc3ccc(F)c(c3)C(=O)N4CCN(CC4)C(=O)C5CC5 | Olaparib 302 |
| CC(=O)N1N=CC(=C(Cl)C1=O)Oc2cccc3C(=NNC(=O)c23)Cc4ccc(F)c(c4)C(=O)N5CCN(CC5)C(=O)C6CC6 | Olaparib 2289 |
| CC(C)[C@H]1C[C@@H](CC(=O)Oc2cccc3C(=NNC(=O)c23)Cc4ccc(F)c(c4)C(=O)N5CCN(CC5)C(=O)C6CC6)CCO1 | Olaparib 1469 |
| Cc1cccc(O)c1OC(=O)c2cccc3C(=NNC(=O)c23)Cc4ccc(F)c(c4)C(=O)N5CCN(CC5)C(=O)C6CC6 | Olaparib 959 |
| CC(=O)CC(=O)Oc1cccc2C(=NNC(=O)c12)Cc3ccc(F)c(c3)C(=O)N4CCN(CC4)C(=O)C5CC5 | Olaparib 1393 |
| CNC(=O)c1cc(COC(=O)c2cccc3C(=NNC(=O)c23)Cc4ccc(F)c(c4)C(=O)N5CCN(CC5)C(=O)C6CC6)on1 | Olaparib 851 |
| C[C@@H]([C@H](N)C(=O)Nc1cccc2C(=NNC(=O)c12)Cc3ccc(F)c(c3)C(=O)N4CCN(CC4)C(=O)C5CC5)C(=O)O | Olaparib 297 |
| OC1=C(Oc2cccc3C(=NNC(=O)c23)Cc4ccc(F)c(c4)C(=O)N5CCN(CC5)C(=O)C6CC6)C(=O)C(=O)C1=O | Olaparib 2994 |
| CC(C)CCN1C[C@@H](CC1=O)Oc2cccc3C(=NNC(=O)c23)Cc4ccc(F)c(c4)C(=O)N5CCN(CC5)C(=O)C6CC6 | Olaparib 2916 |
| Cc1cccc(OC(=O)c2cccc3C(=NNC(=O)c23)Cc4ccc(F)c(c4)C(=O)N5CCN(CC5)C(=O)C6CC6)c1O | Olaparib 960 |
| CO\C=C\C(=O)Nc1cccc2C(=NNC(=O)c12)Cc3ccc(F)c(c3)C(=O)N4CCN(CC4)C(=O)C5CC5 | Olaparib 448 |
| COC(=O)COc1cnnc(Cl)c1Oc2cccc3C(=NNC(=O)c23)Cc4ccc(F)c(c4)C(=O)N5CCN(CC5)C(=O)C6CC6 | Olaparib 2224 |
| C[C@H](NC(=O)CN)C(=O)Oc1cccc2C(=NNC(=O)c12)Cc3ccc(F)c(c3)C(=O)N4CCN(CC4)C(=O)C5CC5 | Olaparib 1219 |
| CN(C1CCNCC1)C(=O)\C(=C(/C)\C(=O)Oc2cccc3C(=NNC(=O)c23)Cc4ccc(F)c(c4)C(=O)N5CCN(CC5)C(=O)C6CC6)\C | Olaparib 1410 |
| CC1(C)OC[C@@H](O1)[C@@H](Oc2cccc3C(=NNC(=O)c23)Cc4ccc(F)c(c4)C(=O)N5CCN(CC5)C(=O)C6CC6)C=C | Olaparib 2953 |
| Fc1ccc(CC2=NNC(=O)c3c(OC(F)(F)C(=O)CSc4ccccc4)cccc23)cc1C(=O)N5CCN(CC5)C(=O)C6CC6 | Olaparib 2261 |
| C[C@@H](F)C(=O)C(=O)Oc1cccc2C(=NNC(=O)c12)Cc3ccc(F)c(c3)C(=O)N4CCN(CC4)C(=O)C5CC5 | Olaparib 1321 |
| CC(C)(NC(=O)c1cccc2C(=NNC(=O)c12)Cc3ccc(F)c(c3)C(=O)N4CCN(CC4)C(=O)C5CC5)c6cn(CCN)nn6 | Olaparib 164 |
| C[C@H](N)C(=O)NCC(=O)Oc1cccc2C(=NNC(=O)c12)Cc3ccc(F)c(c3)C(=O)N4CCN(CC4)C(=O)C5CC5 | Olaparib 1220 |
| CCC[C@@H](C)OC1=C(Oc2cccc3C(=NNC(=O)c23)Cc4ccc(F)c(c4)C(=O)N5CCN(CC5)C(=O)C6CC6)C(=O)NN=C1 | Olaparib 2268 |
| CC1(C)O[C@H](COc2cccc3C(=NNC(=O)c23)Cc4ccc(F)c(c4)C(=O)N5CCN(CC5)C(=O)C6CC6)[C@@H](O1)C=C | Olaparib 2952 |
| Nc1ccccc1COC(=O)c2cccc3C(=NNC(=O)c23)Cc4ccc(F)c(c4)C(=O)N5CCN(CC5)C(=O)C6CC6 | Olaparib 962 |
| OC(=O)C(=O)C(F)(F)c1cccc2C(=NNC(=O)c12)Cc3ccc(F)c(c3)C(=O)N4CCN(CC4)C(=O)C5CC5 | Olaparib 1632 |
| COCCSc1nsc(Oc2cccc3C(=NNC(=O)c23)Cc4ccc(F)c(c4)C(=O)N5CCN(CC5)C(=O)C6CC6)n1 | Olaparib 2283 |
| COC(=O)[C@@H](CN)NC(=O)c1cccc2C(=NNC(=O)c12)Cc3ccc(F)c(c3)C(=O)N4CCN(CC4)C(=O)C5CC5 | Olaparib 55 |
| CC(C)CCOC1=C(Oc2cccc3C(=NNC(=O)c23)Cc4ccc(F)c(c4)C(=O)N5CCN(CC5)C(=O)C6CC6)C(=O)NN=C1 | Olaparib 2267 |
| O[C@H](C1CCCCC1)C(=O)Nc2cccc3C(=NNC(=O)c23)Cc4ccc(F)c(c4)C(=O)N5CCN(CC5)C(=O)C6CC6 | Olaparib 506 |
| O[C@@H](C(=O)Oc1cccc2C(=NNC(=O)c12)Cc3ccc(F)c(c3)C(=O)N4CCN(CC4)C(=O)C5CC5)c6ccc(Cl)cc6 | Olaparib 1467 |
| CN(C)C(C)(C)CNC(=O)c1cccc2C(=NNC(=O)c12)Cc3ccc(F)c(c3)C(=O)N4CCN(CC4)C(=O)C5CC5 | Olaparib 73 |
| OC1=C(OC(=O)c2cccc3C(=NNC(=O)c23)Cc4ccc(F)c(c4)C(=O)N5CCN(CC5)C(=O)C6CC6)C(=O)C(=O)C1=O | Olaparib 911 |
| CC(=O)N[C@@H](CO)C(=O)Oc1cccc2C(=NNC(=O)c12)Cc3ccc(F)c(c3)C(=O)N4CCN(CC4)C(=O)C5CC5 | Olaparib 1214 |
| COCCOC1=C(C(=O)NN=C1)c2cccc3C(=NNC(=O)c23)Cc4ccc(F)c(c4)C(=O)N5CCN(CC5)C(=O)C6CC6 | Olaparib 1680 |
| O[C@H](C1CCCCC1)C(=O)Oc2cccc3C(=NNC(=O)c23)Cc4ccc(F)c(c4)C(=O)N5CCN(CC5)C(=O)C6CC6 | Olaparib 1495 |
| CC(=O)Oc1ccc(Oc2cccc3C(=NNC(=O)c23)Cc4ccc(F)c(c4)C(=O)N5CCN(CC5)C(=O)C6CC6)cc1 | Olaparib 2412 |
| CC[C@](C)(N(C)C)C(=O)Nc1cccc2C(=NNC(=O)c12)Cc3ccc(F)c(c3)C(=O)N4CCN(CC4)C(=O)C5CC5 | Olaparib 305 |
| CC1=C(COC(=O)c2cccc3C(=NNC(=O)c23)Cc4ccc(F)c(c4)C(=O)N5CCN(CC5)C(=O)C6CC6)C(=O)NC(=O)N1 | Olaparib 852 |
| O[C@@H](CCNC(=O)c1cccc2C(=NNC(=O)c12)Cc3ccc(F)c(c3)C(=O)N4CCN(CC4)C(=O)C5CC5)C(=O)O | Olaparib 50 |
| COC1=C(Oc2cccc3C(=NNC(=O)c23)Cc4ccc(F)c(c4)C(=O)N5CCN(CC5)C(=O)C6CC6)C(=O)N(C)N=C1 | Olaparib 2372 |
| OC1(CCCCC1)C(=O)COc2cccc3C(=NNC(=O)c23)Cc4ccc(F)c(c4)C(=O)N5CCN(CC5)C(=O)C6CC6 | Olaparib 2955 |
| OCc1ccc(Cl)cc1OC(=O)c2cccc3C(=NNC(=O)c23)Cc4ccc(F)c(c4)C(=O)N5CCN(CC5)C(=O)C6CC6 | Olaparib 841 |
| CC[C@](C)(N(C)C)C(=O)Oc1cccc2C(=NNC(=O)c12)Cc3ccc(F)c(c3)C(=O)N4CCN(CC4)C(=O)C5CC5 | Olaparib 1225 |
| NC1=NC(=O)NC=C1COC(=O)c2cccc3C(=NNC(=O)c23)Cc4ccc(F)c(c4)C(=O)N5CCN(CC5)C(=O)C6CC6 | Olaparib 914 |
| O[C@H](CCNC(=O)c1cccc2C(=NNC(=O)c12)Cc3ccc(F)c(c3)C(=O)N4CCN(CC4)C(=O)C5CC5)C(=O)O | Olaparib 49 |
| CC1=C(C#N)C(=O)NC(=C1Oc2cccc3C(=NNC(=O)c23)Cc4ccc(F)c(c4)C(=O)N5CCN(CC5)C(=O)C6CC6)O | Olaparib 2347 |
| N[C@@H]1CC[C@](C1)(OC(=O)c2cccc3C(=NNC(=O)c23)Cc4ccc(F)c(c4)C(=O)N5CCN(CC5)C(=O)C6CC6)C(F)(F)F | Olaparib 795 |
| CC(=O)Oc1c(Cl)cc(Oc2cccc3C(=NNC(=O)c23)Cc4ccc(F)c(c4)C(=O)N5CCN(CC5)C(=O)C6CC6)cc1Cl | Olaparib 2220 |
| CC[C@H](C)[C@@H](CO)NC(=O)c1cccc2C(=NNC(=O)c12)Cc3ccc(F)c(c3)C(=O)N4CCN(CC4)C(=O)C5CC5 | Olaparib 57 |
| Fc1ccc(CC2=NNC(=O)c3c(cccc23)C(=O)OCC4=CNC(=O)NC4=O)cc1C(=O)N5CCN(CC5)C(=O)C6CC6 | Olaparib 910 |
| O[C@@H](CNC(=O)c1cccc2C(=NNC(=O)c12)Cc3ccc(F)c(c3)C(=O)N4CCN(CC4)C(=O)C5CC5)CC(=O)O | Olaparib 47 |
| CN1C(=O)NC(=CC1=O)Oc2cccc3C(=NNC(=O)c23)Cc4ccc(F)c(c4)C(=O)N5CCN(CC5)C(=O)C6CC6 | Olaparib 2397 |
| CSCCC1(CC1)OC(=O)c2cccc3C(=NNC(=O)c23)Cc4ccc(F)c(c4)C(=O)N5CCN(CC5)C(=O)C6CC6 | Olaparib 944 |
| Oc1ccc(COC(=O)c2cccc3C(=NNC(=O)c23)Cc4ccc(F)c(c4)C(=O)N5CCN(CC5)C(=O)C6CC6)cc1Br | Olaparib 717 |
| CC(C)[C@H](N)CC(=O)Nc1cccc2C(=NNC(=O)c12)Cc3ccc(F)c(c3)C(=O)N4CCN(CC4)C(=O)C5CC5 | Olaparib 347 |
| CC(C)(C(=O)N1CC[C@H](C1)Oc2cccc3C(=NNC(=O)c23)Cc4ccc(F)c(c4)C(=O)N5CCN(CC5)C(=O)C6CC6)S(=O)(=O)C | Olaparib 2858 |
| O[C@H](CNC(=O)c1cccc2C(=NNC(=O)c12)Cc3ccc(F)c(c3)C(=O)N4CCN(CC4)C(=O)C5CC5)CC(=O)O | Olaparib 45 |
| CC1=C(Oc2cccc3C(=NNC(=O)c23)Cc4ccc(F)c(c4)C(=O)N5CCN(CC5)C(=O)C6CC6)C(=O)NC(=O)N1 | Olaparib 2295 |
| Fc1ccc(CC2=NNC(=O)c3c(O[C@H]4CN(CC(F)(F)F)C(=O)C4)cccc23)cc1C(=O)N5CCN(CC5)C(=O)C6CC6 | Olaparib 2894 |
| OC(=O)Cc1cc(Oc2cccc3C(=NNC(=O)c23)Cc4ccc(F)c(c4)C(=O)N5CCN(CC5)C(=O)C6CC6)ccc1F | Olaparib 2377 |
| CC(C)C[13C@@H](N)C(=O)Nc1cccc2C(=NNC(=O)c12)Cc3ccc(F)c(c3)C(=O)N4CCN(CC4)C(=O)C5CC5 | Olaparib 341 |
| CCNC(=O)[C@H](C)N1C[C@@H](CC1=O)OC(=O)c2cccc3C(=NNC(=O)c23)Cc4ccc(F)c(c4)C(=O)N5CCN(CC5)C(=O)C6CC6 | Olaparib 721 |
| O[C@H](CNC(=O)c1cccc2C(=NNC(=O)c12)Cc3ccc(F)c(c3)C(=O)N4CCN(CC4)C(=O)C5CC5)CC(=O)O | Olaparib 44 |
| Fc1ccc(CC2=NNC(=O)c3c(OC4=C(OCC(F)(F)F)C=NNC4=O)cccc23)cc1C(=O)N5CCN(CC5)C(=O)C6CC6 | Olaparib 2237 |
| Fc1ccc(CC2=NNC(=O)c3c(OC(=O)C[C@@H]4CCCO4)cccc23)cc1C(=O)N5CCN(CC5)C(=O)C6CC6 | Olaparib 1513 |
| CC(=O)Oc1ccc(Oc2cccc3C(=NNC(=O)c23)Cc4ccc(F)c(c4)C(=O)N5CCN(CC5)C(=O)C6CC6)cc1Cl | Olaparib 2294 |
| CC[C@H](C)[C@@H](N)C(=O)Nc1cccc2C(=NNC(=O)c12)Cc3ccc(F)c(c3)C(=O)N4CCN(CC4)C(=O)C5CC5 | Olaparib 351 |
| C[C@H](CN[C@H]1CCCS(=O)(=O)C1)OC(=O)c2cccc3C(=NNC(=O)c23)Cc4ccc(F)c(c4)C(=O)N5CCN(CC5)C(=O)C6CC6 | Olaparib 711 |
| NC[C@@H](NC(=O)c1cccc2C(=NNC(=O)c12)Cc3ccc(F)c(c3)C(=O)N4CCN(CC4)C(=O)C5CC5)C(=O)O | Olaparib 97 |
| NC1=NC(=CC(=O)N1)Oc2cccc3C(=NNC(=O)c23)Cc4ccc(F)c(c4)C(=O)N5CCN(CC5)C(=O)C6CC6 | Olaparib 3017 |
| CN1C[C@@H](CC1=O)Oc2cccc3C(=NNC(=O)c23)Cc4ccc(F)c(c4)C(=O)N5CCN(CC5)C(=O)C6CC6 | Olaparib 3035 |
| Nc1ccc(OC(=O)c2cccc3C(=NNC(=O)c23)Cc4ccc(F)c(c4)C(=O)N5CCN(CC5)C(=O)C6CC6)cc1 | Olaparib 982 |
| CC(=O)[C@@H](O)C(C)(C)Oc1cccc2C(=NNC(=O)c12)Cc3ccc(F)c(c3)C(=O)N4CCN(CC4)C(=O)C5CC5 | Olaparib 2769 |
| CS(=O)(=O)CC(=O)N1CCC[C@H](C1)Oc2cccc3C(=NNC(=O)c23)Cc4ccc(F)c(c4)C(=O)N5CCN(CC5)C(=O)C6CC6 | Olaparib 2865 |
| O[C@@H](CNC(=O)c1cccc2C(=NNC(=O)c12)Cc3ccc(F)c(c3)C(=O)N4CCN(CC4)C(=O)C5CC5)C(=O)O | Olaparib 95 |
| NC1=CC(=NNC1=O)Oc2cccc3C(=NNC(=O)c23)Cc4ccc(F)c(c4)C(=O)N5CCN(CC5)C(=O)C6CC6 | Olaparib 2417 |
| C[C@@H](CN1CC[C@H](C)[C@@H](N)C1)Oc2cccc3C(=NNC(=O)c23)Cc4ccc(F)c(c4)C(=O)N5CCN(CC5)C(=O)C6CC6 | Olaparib 2912 |
| Oc1cc(Cl)c(OC(=O)c2cccc3C(=NNC(=O)c23)Cc4ccc(F)c(c4)C(=O)N5CCN(CC5)C(=O)C6CC6)cc1Cl | Olaparib 770 |
| CC(C)NC(=O)[C@@H](C)Oc1cccc2C(=NNC(=O)c12)Cc3ccc(F)c(c3)C(=O)N4CCN(CC4)C(=O)C5CC5 | Olaparib 2772 |
| CN[C@@]1(COC(=O)c2cccc3C(=NNC(=O)c23)Cc4ccc(F)c(c4)C(=O)N5CCN(CC5)C(=O)C6CC6)CCCS(=O)(=O)C1 | Olaparib 732 |
| OC[C@@H](NC(=O)c1cccc2C(=NNC(=O)c12)Cc3ccc(F)c(c3)C(=O)N4CCN(CC4)C(=O)C5CC5)C(=O)O | Olaparib 94 |
| CCC1(C)CCN(CC1)C(=O)N[C@H](C)C(=O)Oc2cccc3C(=NNC(=O)c23)Cc4ccc(F)c(c4)C(=O)N5CCN(CC5)C(=O)C6CC6 | Olaparib 1405 |
| COC[C@H](C)N1CCC[C@H](C1)Oc2cccc3C(=NNC(=O)c23)Cc4ccc(F)c(c4)C(=O)N5CCN(CC5)C(=O)C6CC6 | Olaparib 2909 |
| O\N=C\c1ccc(Oc2cccc3C(=NNC(=O)c23)Cc4ccc(F)c(c4)C(=O)N5CCN(CC5)C(=O)C6CC6)cc1 | Olaparib 3006 |
| COCC(C)(C)C(=O)Nc1cccc2C(=NNC(=O)c12)Cc3ccc(F)c(c3)C(=O)N4CCN(CC4)C(=O)C5CC5 | Olaparib 344 |
| Fc1ccc(CC2=NNC(=O)c3c(cccc23)C(=O)O[C@H]4CS(=O)(=O)C[C@@H]4NCCC#C)cc1C(=O)N5CCN(CC5)C(=O)C6CC6 | Olaparib 716 |
| OS(=O)(=O)[C@@](F)(Oc1cccc2C(=NNC(=O)c12)Cc3ccc(F)c(c3)C(=O)N4CCN(CC4)C(=O)C5CC5)C(F)F | Olaparib 2123 |
| CN(C)C(=O)N1CCC[C@H](CC(=O)Oc2cccc3C(=NNC(=O)c23)Cc4ccc(F)c(c4)C(=O)N5CCN(CC5)C(=O)C6CC6)C1 | Olaparib 1434 |
| CC1(C)OC[C@@H](CCOc2cccc3C(=NNC(=O)c23)Cc4ccc(F)c(c4)C(=O)N5CCN(CC5)C(=O)C6CC6)O1 | Olaparib 2983 |
| Fc1ccc(CC2=NNC(=O)c3c(OC(=O)Cc4cc(Cl)cnc4Cl)cccc23)cc1C(=O)N5CCN(CC5)C(=O)C6CC6 | Olaparib 1445 |
| CC[C@H](C)[C@@H](N)C(=O)Oc1cccc2C(=NNC(=O)c12)Cc3ccc(F)c(c3)C(=O)N4CCN(CC4)C(=O)C5CC5 | Olaparib 1283 |
| CC1=C(CCOc2cccc3C(=NNC(=O)c23)Cc4ccc(F)c(c4)C(=O)N5CCN(CC5)C(=O)C6CC6)C(=O)N(N1)C(=N)N | Olaparib 2889 |
| OC(=O)C(F)(Oc1cccc2C(=NNC(=O)c12)Cc3ccc(F)c(c3)C(=O)N4CCN(CC4)C(=O)C5CC5)C(=O)O | Olaparib 2157 |
| C[C@H](N(C)C(=O)[C@H]1CCSC1)C(=O)Nc2cccc3C(=NNC(=O)c23)Cc4ccc(F)c(c4)C(=O)N5CCN(CC5)C(=O)C6CC6 | Olaparib 468 |
| N[C@@H]1CNCCC[C@@H]1Oc2cccc3C(=NNC(=O)c23)Cc4ccc(F)c(c4)C(=O)N5CCN(CC5)C(=O)C6CC6 | Olaparib 3013 |
| O\N=C(/Cl)\c1ccc(Oc2cccc3C(=NNC(=O)c23)Cc4ccc(F)c(c4)C(=O)N5CCN(CC5)C(=O)C6CC6)cc1 | Olaparib 2229 |
| CCC[C@](C)(N)C(=O)Oc1cccc2C(=NNC(=O)c12)Cc3ccc(F)c(c3)C(=O)N4CCN(CC4)C(=O)C5CC5 | Olaparib 1280 |
| NC(=O)CN1C[C@@H](CC1=O)OC(=O)c2cccc3C(=NNC(=O)c23)Cc4ccc(F)c(c4)C(=O)N5CCN(CC5)C(=O)C6CC6 | Olaparib 847 |
| [O-]S(=O)(=O)[C@](F)(Oc1cccc2C(=NNC(=O)c12)Cc3ccc(F)c(c3)C(=O)N4CCN(CC4)C(=O)C5CC5)C(F)(F)F | Olaparib 2110 |
| OCC1CCN(CC1)C(=O)C(=O)Oc2cccc3C(=NNC(=O)c23)Cc4ccc(F)c(c4)C(=O)N5CCN(CC5)C(=O)C6CC6 | Olaparib 1466 |
| Fc1ccc(CC2=NNC(=O)c3c(OCCN4CCOC4)cccc23)cc1C(=O)N5CCN(CC5)C(=O)C6CC6 | Olaparib 3032 |
| Oc1ccc(C=O)cc1Oc2cccc3C(=NNC(=O)c23)Cc4ccc(F)c(c4)C(=O)N5CCN(CC5)C(=O)C6CC6 | Olaparib 2315 |
| CC(C)(C)[C@H](O)C(=O)Oc1cccc2C(=NNC(=O)c12)Cc3ccc(F)c(c3)C(=O)N4CCN(CC4)C(=O)C5CC5 | Olaparib 1269 |
| Fc1ccc(CC2=NNC(=O)c3c(OC(=O)CCN4CCCS4(=O)=O)cccc23)cc1C(=O)N5CCN(CC5)C(=O)C6CC6 | Olaparib 1460 |
| NC[C@@H](O)[C@@H](O)CNC(=O)c1cccc2C(=NNC(=O)c12)Cc3ccc(F)c(c3)C(=O)N4CCN(CC4)C(=O)C5CC5 | Olaparib 40 |
| [15NH2][C@H](C[15NH]O)C(=O)Oc1cccc2C(=NNC(=O)c12)Cc3ccc(F)c(c3)C(=O)N4CCN(CC4)C(=O)C5CC5 | Olaparib 1316 |
| NC(=O)CN1C[C@@H](CC1=O)Oc2cccc3C(=NNC(=O)c23)Cc4ccc(F)c(c4)C(=O)N5CCN(CC5)C(=O)C6CC6 | Olaparib 2956 |
| CC\C(=C\CN)\c1cccc(Oc2cccc3C(=NNC(=O)c23)Cc4ccc(F)c(c4)C(=O)N5CCN(CC5)C(=O)C6CC6)c1 | Olaparib 2323 |
| Oc1ccc(Cl)cc1OC(=O)c2cccc3C(=NNC(=O)c23)Cc4ccc(F)c(c4)C(=O)N5CCN(CC5)C(=O)C6CC6 | Olaparib 898 |
| CC(C)C[13C@@H](N)C(=O)Oc1cccc2C(=NNC(=O)c12)Cc3ccc(F)c(c3)C(=O)N4CCN(CC4)C(=O)C5CC5 | Olaparib 1268 |
| Fc1ccc(CC2=NNC(=O)c3c(cccc23)C(=O)OCCN4C(=O)CNC4=O)cc1C(=O)N5CCN(CC5)C(=O)C6CC6 | Olaparib 905 |
| [15NH2][C@H](C[15NH]O)C(=O)Nc1cccc2C(=NNC(=O)c12)Cc3ccc(F)c(c3)C(=O)N4CCN(CC4)C(=O)C5CC5 | Olaparib 381 |
| CC(=CCNC(=N)NC(=O)c1cccc2C(=NNC(=O)c12)Cc3ccc(F)c(c3)C(=O)N4CCN(CC4)C(=O)C5CC5)C | Olaparib 37 |
| Fc1ccc(CC2=NNC(=O)c3c(OCCN4C(=O)CNC4=O)cccc23)cc1C(=O)N5CCN(CC5)C(=O)C6CC6 | Olaparib 2992 |
| CC(C)(COc1cccc2C(=NNC(=O)c12)Cc3ccc(F)c(c3)C(=O)N4CCN(CC4)C(=O)C5CC5)\N=C\c6ccccc6 | Olaparib 2905 |
| C[C@@H](N)c1ccc(cc1Oc2cccc3C(=NNC(=O)c23)Cc4ccc(F)c(c4)C(=O)N5CCN(CC5)C(=O)C6CC6)N(C)CC(C)(C)C | Olaparib 2195 |
| CC[C@H](N)CCNC(=O)c1cccc2C(=NNC(=O)c12)Cc3ccc(F)c(c3)C(=O)N4CCN(CC4)C(=O)C5CC5 | Olaparib 113 |
| NC1=NC(=O)N(CCC#N)C=C1Oc2cccc3C(=NNC(=O)c23)Cc4ccc(F)c(c4)C(=O)N5CCN(CC5)C(=O)C6CC6 | Olaparib 2354 |
| CN(CC(=O)Nc1cccc2C(=NNC(=O)c12)Cc3ccc(F)c(c3)C(=O)N4CCN(CC4)C(=O)C5CC5)C(=O)C=C | Olaparib 315 |
| CC(C)(C#N)N1CCN(CC1)C(=O)C(F)(F)Oc2cccc3C(=NNC(=O)c23)Cc4ccc(F)c(c4)C(=O)N5CCN(CC5)C(=O)C6CC6 | Olaparib 2206 |
| CO[C@@H](CC=C)c1ccc(Oc2cccc3C(=NNC(=O)c23)Cc4ccc(F)c(c4)C(=O)N5CCN(CC5)C(=O)C6CC6)cc1 | Olaparib 2321 |
| CCC(C)(C)N(C)Cc1ccc(N)c(Oc2cccc3C(=NNC(=O)c23)Cc4ccc(F)c(c4)C(=O)N5CCN(CC5)C(=O)C6CC6)c1 | Olaparib 2246 |
| CC(C)(CN)C(=O)Nc1cccc2C(=NNC(=O)c12)Cc3ccc(F)c(c3)C(=O)N4CCN(CC4)C(=O)C5CC5 | Olaparib 400 |
| O[C@H]1[C@H](O)[C@H](O[C@@H]1COC(=O)c2cccc3C(=NNC(=O)c23)Cc4ccc(F)c(c4)C(=O)N5CCN(CC5)C(=O)C6CC6)C=O | Olaparib 831 |
| C\C(=C/C(=O)Nc1cccc2C(=NNC(=O)c12)Cc3ccc(F)c(c3)C(=O)N4CCN(CC4)C(=O)C5CC5)\CC(=O)O | Olaparib 311 |
| O[C@@H]1CCN[C@H](COC(=O)c2cccc3C(=NNC(=O)c23)Cc4ccc(F)c(c4)C(=O)N5CCN(CC5)C(=O)C6CC6)[C@H]1O | Olaparib 888 |
| C[C@H](OC(=O)c1cccc2C(=NNC(=O)c12)Cc3ccc(F)c(c3)C(=O)N4CCN(CC4)C(=O)C5CC5)c6cccc(C)c6 | Olaparib 933 |
| COc1c(Cl)cc(cc1Oc2cccc3C(=NNC(=O)c23)Cc4ccc(F)c(c4)C(=O)N5CCN(CC5)C(=O)C6CC6)C(C)(C)N | Olaparib 2266 |
| C[C@@H](N)[C@H](C)C(=O)Nc1cccc2C(=NNC(=O)c12)Cc3ccc(F)c(c3)C(=O)N4CCN(CC4)C(=O)C5CC5 | Olaparib 398 |
| OC[C@H]1O[C@H](C=O)[C@@H](OC(=O)c2cccc3C(=NNC(=O)c23)Cc4ccc(F)c(c4)C(=O)N5CCN(CC5)C(=O)C6CC6)[C@@H]1O | Olaparib 832 |
| CCNC(=O)\C=C\C(=O)Oc1cccc2C(=NNC(=O)c12)Cc3ccc(F)c(c3)C(=O)N4CCN(CC4)C(=O)C5CC5 | Olaparib 1236 |
| COC(=O)c1ccc(c(F)c1)C(C)(C)NC(=O)c2cccc3C(=NNC(=O)c23)Cc4ccc(F)c(c4)C(=O)N5CCN(CC5)C(=O)C6CC6 | Olaparib 142 |
| Cc1cc(C)c(C=O)c(Oc2cccc3C(=NNC(=O)c23)Cc4ccc(F)c(c4)C(=O)N5CCN(CC5)C(=O)C6CC6)c1 | Olaparib 2976 |
| CC(C)(C#N)c1ccc(Oc2cccc3C(=NNC(=O)c23)Cc4ccc(F)c(c4)C(=O)N5CCN(CC5)C(=O)C6CC6)c(N)c1 | Olaparib 2365 |
| CC[C@H](COC)NC(=O)c1cccc2C(=NNC(=O)c12)Cc3ccc(F)c(c3)C(=O)N4CCN(CC4)C(=O)C5CC5 | Olaparib 99 |
| OC[C@H]1O[C@H](C=O)[C@@H](O)[C@H]1OC(=O)c2cccc3C(=NNC(=O)c23)Cc4ccc(F)c(c4)C(=O)N5CCN(CC5)C(=O)C6CC6 | Olaparib 830 |
| CNC(=O)\C=C\C(=O)Oc1cccc2C(=NNC(=O)c12)Cc3ccc(F)c(c3)C(=O)N4CCN(CC4)C(=O)C5CC5 | Olaparib 1301 |
| COc1ccc(cc1)[C@H](OC(=O)c2cccc3C(=NNC(=O)c23)Cc4ccc(F)c(c4)C(=O)N5CCN(CC5)C(=O)C6CC6)C(=O)C | Olaparib 767 |
| CC(=O)Nc1cccc(c1)c2cccc3C(=NNC(=O)c23)Cc4ccc(F)c(c4)C(=O)N5CCN(CC5)C(=O)C6CC6 | Olaparib 1710 |
| CC(C)[C@H](OC(=O)c1cccc2C(=NNC(=O)c12)Cc3ccc(F)c(c3)C(=O)N4CCN(CC4)C(=O)C5CC5)c6occc6 | Olaparib 915 |
| CCC[C@@H](N)C(=O)Nc1cccc2C(=NNC(=O)c12)Cc3ccc(F)c(c3)C(=O)N4CCN(CC4)C(=O)C5CC5 | Olaparib 396 |
| CO[C@@H]1O[C@@H](C)[C@@H](O)[C@@H](O)[C@H]1OC(=O)c2cccc3C(=NNC(=O)c23)Cc4ccc(F)c(c4)C(=O)N5CCN(CC5)C(=O)C6CC6 | Olaparib 773 |
| OC(=O)\C(=C(/C(=O)O)\c1cccc2C(=NNC(=O)c12)Cc3ccc(F)c(c3)C(=O)N4CCN(CC4)C(=O)C5CC5)\Br | Olaparib 1601 |
| CC(C)(C)[C@@H](Cc1cnccc1N)OC(=O)c2cccc3C(=NNC(=O)c23)Cc4ccc(F)c(c4)C(=O)N5CCN(CC5)C(=O)C6CC6 | Olaparib 731 |
| COc1ccc(\C=C\Oc2cccc3C(=NNC(=O)c23)Cc4ccc(F)c(c4)C(=O)N5CCN(CC5)C(=O)C6CC6)cc1 | Olaparib 2390 |
| CNC[C@@H](O)c1ccc(Oc2cccc3C(=NNC(=O)c23)Cc4ccc(F)c(c4)C(=O)N5CCN(CC5)C(=O)C6CC6)cc1 | Olaparib 2385 |
| C[C@@H](N)[C@H](C)C(=O)Oc1cccc2C(=NNC(=O)c12)Cc3ccc(F)c(c3)C(=O)N4CCN(CC4)C(=O)C5CC5 | Olaparib 1335 |
| CO[C@@H]1O[C@@H](C)[C@H](OC(=O)c2cccc3C(=NNC(=O)c23)Cc4ccc(F)c(c4)C(=O)N5CCN(CC5)C(=O)C6CC6)[C@@H](O)[C@@H]1O | Olaparib 774 |
| C[C@H](N(C)C(=O)C(C)(C)C)C(=O)Oc1cccc2C(=NNC(=O)c12)Cc3ccc(F)c(c3)C(=O)N4CCN(CC4)C(=O)C5CC5 | Olaparib 1179 |
| C[C@@H](CCN)N(C)C(=O)c1ccc(Oc2cccc3C(=NNC(=O)c23)Cc4ccc(F)c(c4)C(=O)N5CCN(CC5)C(=O)C6CC6)cc1F | Olaparib 2217 |
| Fc1ccc(CC2=NNC(=O)c3c(OC(=O)CCc4ccc(Cl)cc4)cccc23)cc1C(=O)N5CCN(CC5)C(=O)C6CC6 | Olaparib 1471 |
| OCCCc1ccc(Oc2cccc3C(=NNC(=O)c23)Cc4ccc(F)c(c4)C(=O)N5CCN(CC5)C(=O)C6CC6)c(O)c1 | Olaparib 2924 |
| C[C@H](CN)CC(=O)Nc1cccc2C(=NNC(=O)c12)Cc3ccc(F)c(c3)C(=O)N4CCN(CC4)C(=O)C5CC5 | Olaparib 399 |
| CO[C@H]1OC[C@@H](OC(=O)c2cccc3C(=NNC(=O)c23)Cc4ccc(F)c(c4)C(=O)N5CCN(CC5)C(=O)C6CC6)[C@H](O)[C@H]1O | Olaparib 827 |
| CC(C)C(=O)NC(C)(C)C(=O)Nc1cccc2C(=NNC(=O)c12)Cc3ccc(F)c(c3)C(=O)N4CCN(CC4)C(=O)C5CC5 | Olaparib 277 |
| COc1cc(cc(Cl)c1OC(=O)c2cccc3C(=NNC(=O)c23)Cc4ccc(F)c(c4)C(=O)N5CCN(CC5)C(=O)C6CC6)C(C)(C)N | Olaparib 703 |
| Fc1ccc(CC2=NNC(=O)c3c(OC(=O)CCc4ccccc4Br)cccc23)cc1C(=O)N5CCN(CC5)C(=O)C6CC6 | Olaparib 1421 |
| COc1cc(Oc2cccc3C(=NNC(=O)c23)Cc4ccc(F)c(c4)C(=O)N5CCN(CC5)C(=O)C6CC6)c(C)cc1N | Olaparib 2380 |
| CC(C)(CN)C(=O)Oc1cccc2C(=NNC(=O)c12)Cc3ccc(F)c(c3)C(=O)N4CCN(CC4)C(=O)C5CC5 | Olaparib 1337 |
| CO[C@@H]1OC[C@H](OC(=O)c2cccc3C(=NNC(=O)c23)Cc4ccc(F)c(c4)C(=O)N5CCN(CC5)C(=O)C6CC6)[C@@H](O)[C@H]1O | Olaparib 826 |
| CC(C)(C(=O)O)C(C)(C)C(=O)Oc1cccc2C(=NNC(=O)c12)Cc3ccc(F)c(c3)C(=O)N4CCN(CC4)C(=O)C5CC5 | Olaparib 1182 |
| CC(C)(C(=O)O)c1cc(Br)c(Oc2cccc3C(=NNC(=O)c23)Cc4ccc(F)c(c4)C(=O)N5CCN(CC5)C(=O)C6CC6)cc1O | Olaparib 2168 |
| COc1ccc(cc1)\C(=C\C#N)\c2cccc3C(=NNC(=O)c23)Cc4ccc(F)c(c4)C(=O)N5CCN(CC5)C(=O)C6CC6 | Olaparib 1692 |
| Nc1cc(Oc2cccc3C(=NNC(=O)c23)Cc4ccc(F)c(c4)C(=O)N5CCN(CC5)C(=O)C6CC6)ccc1OCC#C | Olaparib 2355 |
| CC[C@@H](F)[C@@H](N)C(=O)Nc1cccc2C(=NNC(=O)c12)Cc3ccc(F)c(c3)C(=O)N4CCN(CC4)C(=O)C5CC5 | Olaparib 331 |
| CO[C@@H]1OC[C@@H](O)[C@H](OC(=O)c2cccc3C(=NNC(=O)c23)Cc4ccc(F)c(c4)C(=O)N5CCN(CC5)C(=O)C6CC6)[C@H]1O | Olaparib 825 |
| CC(C)C(=O)NC(C)(C)C(=O)Oc1cccc2C(=NNC(=O)c12)Cc3ccc(F)c(c3)C(=O)N4CCN(CC4)C(=O)C5CC5 | Olaparib 1184 |
| CC(C)(NC(=O)c1cccc2C(=NNC(=O)c12)Cc3ccc(F)c(c3)C(=O)N4CCN(CC4)C(=O)C5CC5)c6cc(C#N)c(F)c(F)c6O | Olaparib 141 |
| O\N=C(\c1ccc(Br)cc1)/c2cccc3C(=NNC(=O)c23)Cc4ccc(F)c(c4)C(=O)N5CCN(CC5)C(=O)C6CC6 | Olaparib 1661 |
| NCc1ccc(O)c(Oc2cccc3C(=NNC(=O)c23)Cc4ccc(F)c(c4)C(=O)N5CCN(CC5)C(=O)C6CC6)c1 | Olaparib 2996 |
| CCC[C@@H](O)C(=O)Nc1cccc2C(=NNC(=O)c12)Cc3ccc(F)c(c3)C(=O)N4CCN(CC4)C(=O)C5CC5 | Olaparib 395 |
| CC(C)(NC(=O)c1cccc2C(=NNC(=O)c12)Cc3ccc(F)c(c3)C(=O)N4CCN(CC4)C(=O)C5CC5)c6cccc(c6)S(=O)(=O)C | Olaparib 140 |
| CC(C)N(C)C(=O)CC(=O)Oc1cccc2C(=NNC(=O)c12)Cc3ccc(F)c(c3)C(=O)N4CCN(CC4)C(=O)C5CC5 | Olaparib 1200 |
| CC(C)(C#N)c1ccc(Oc2cccc3C(=NNC(=O)c23)Cc4ccc(F)c(c4)C(=O)N5CCN(CC5)C(=O)C6CC6)cc1C(=O)O | Olaparib 2287 |
| Fc1cccc(F)c1OC(=O)c2cccc3C(=NNC(=O)c23)Cc4ccc(F)c(c4)C(=O)N5CCN(CC5)C(=O)C6CC6 | Olaparib 949 |
| C[C@H](OC(=O)c1cccc2C(=NNC(=O)c12)Cc3ccc(F)c(c3)C(=O)N4CCN(CC4)C(=O)C5CC5)c6ccsc6 | Olaparib 951 |
| CC[C@@H](F)[C@@H](N)C(=O)Oc1cccc2C(=NNC(=O)c12)Cc3ccc(F)c(c3)C(=O)N4CCN(CC4)C(=O)C5CC5 | Olaparib 1255 |
| CC(C)(C(=O)c1ccc(Oc2cccc3C(=NNC(=O)c23)Cc4ccc(F)c(c4)C(=O)N5CCN(CC5)C(=O)C6CC6)cc1)S(=O)(=O)C | Olaparib 2214 |
| CC(=O)NCC(=O)NCCCc1cccc2C(=NNC(=O)c12)Cc3ccc(F)c(c3)C(=O)N4CCN(CC4)C(=O)C5CC5 | Olaparib 1798 |
| C[C@@H](Nc1ccncc1C)C(=O)Oc2cccc3C(=NNC(=O)c23)Cc4ccc(F)c(c4)C(=O)N5CCN(CC5)C(=O)C6CC6 | Olaparib 1478 |
| COc1ccc(c(c1)c2cccc3C(=NNC(=O)c23)Cc4ccc(F)c(c4)C(=O)N5CCN(CC5)C(=O)C6CC6)C(C)(C)N | Olaparib 1700 |
| Nc1cccc(Oc2cccc3C(=NNC(=O)c23)Cc4ccc(F)c(c4)C(=O)N5CCN(CC5)C(=O)C6CC6)c1O | Olaparib 3020 |
| NC[C@@H](Oc1cccc2C(=NNC(=O)c12)Cc3ccc(F)c(c3)C(=O)N4CCN(CC4)C(=O)C5CC5)[C@H](O)C=C | Olaparib 2809 |
| C[C@@H](CS(=O)(=O)c1ccc(C)cc1)OC(=O)c2cccc3C(=NNC(=O)c23)Cc4ccc(F)c(c4)C(=O)N5CCN(CC5)C(=O)C6CC6 | Olaparib 705 |
| CC[C@](C)(NC(=O)c1cccc2C(=NNC(=O)c12)Cc3ccc(F)c(c3)C(=O)N4CCN(CC4)C(=O)C5CC5)C(=O)O | Olaparib 64 |
| OCc1cc(CO)cc(COC(=O)c2cccc3C(=NNC(=O)c23)Cc4ccc(F)c(c4)C(=O)N5CCN(CC5)C(=O)C6CC6)c1 | Olaparib 797 |
| CC[N+](C)(C)c1cccc(Oc2cccc3C(=NNC(=O)c23)Cc4ccc(F)c(c4)C(=O)N5CCN(CC5)C(=O)C6CC6)c1 | Olaparib 2927 |
| Nc1cccc(O)c1Oc2cccc3C(=NNC(=O)c23)Cc4ccc(F)c(c4)C(=O)N5CCN(CC5)C(=O)C6CC6 | Olaparib 3019 |
| CCC[C@@H](N)C(=O)Oc1cccc2C(=NNC(=O)c12)Cc3ccc(F)c(c3)C(=O)N4CCN(CC4)C(=O)C5CC5 | Olaparib 1333 |
| Fc1ccc(CC2=NNC(=O)c3c(cccc23)C(=O)OCCS(=O)(=O)c4ccccc4)cc1C(=O)N5CCN(CC5)C(=O)C6CC6 | Olaparib 745 |
| CC(C)OC(=O)CNC(=O)c1cccc2C(=NNC(=O)c12)Cc3ccc(F)c(c3)C(=O)N4CCN(CC4)C(=O)C5CC5 | Olaparib 62 |
| CCOc1cc(COC(=O)c2cccc3C(=NNC(=O)c23)Cc4ccc(F)c(c4)C(=O)N5CCN(CC5)C(=O)C6CC6)ccc1O | Olaparib 802 |
| CC(C)(C)c1cc(Oc2cccc3C(=NNC(=O)c23)Cc4ccc(F)c(c4)C(=O)N5CCN(CC5)C(=O)C6CC6)ccc1O | Olaparib 2391 |
| Nc1cc(O)ccc1Oc2cccc3C(=NNC(=O)c23)Cc4ccc(F)c(c4)C(=O)N5CCN(CC5)C(=O)C6CC6 | Olaparib 2429 |
| CC[C@@H](N)C(=O)Nc1cccc2C(=NNC(=O)c12)Cc3ccc(F)c(c3)C(=O)N4CCN(CC4)C(=O)C5CC5 | Olaparib 440 |
| CS(=O)(=O)c1ccc(OC(=O)c2cccc3C(=NNC(=O)c23)Cc4ccc(F)c(c4)C(=O)N5CCN(CC5)C(=O)C6CC6)cc1 | Olaparib 789 |
| CC[C@@H](NC(=O)c1cccc2C(=NNC(=O)c12)Cc3ccc(F)c(c3)C(=O)N4CCN(CC4)C(=O)C5CC5)C(=O)OC | Olaparib 61 |
| CN(C)C(=O)Nc1ccc(Oc2cccc3C(=NNC(=O)c23)Cc4ccc(F)c(c4)C(=O)N5CCN(CC5)C(=O)C6CC6)c(Cl)c1 | Olaparib 2235 |
| CC(C)c1cc(Oc2cccc3C(=NNC(=O)c23)Cc4ccc(F)c(c4)C(=O)N5CCN(CC5)C(=O)C6CC6)ccc1O | Olaparib 2967 |
| ONc1ccc(Oc2cccc3C(=NNC(=O)c23)Cc4ccc(F)c(c4)C(=O)N5CCN(CC5)C(=O)C6CC6)cc1 | Olaparib 2422 |
| C[C@H](OC(=O)C)C(Cl)(Cl)Oc1cccc2C(=NNC(=O)c12)Cc3ccc(F)c(c3)C(=O)N4CCN(CC4)C(=O)C5CC5 | Olaparib 2107 |
| Fc1ccc(CC2=NNC(=O)c3c(cccc23)C(=O)NC(=S)\C(=C\c4cccnc4)\C#N)cc1C(=O)N5CCN(CC5)C(=O)C6CC6 | Olaparib 155 |
| COC(=O)C[C@H](C)NC(=O)c1cccc2C(=NNC(=O)c12)Cc3ccc(F)c(c3)C(=O)N4CCN(CC4)C(=O)C5CC5 | Olaparib 59 |
| N[C@@H](Cc1cccc(Oc2cccc3C(=NNC(=O)c23)Cc4ccc(F)c(c4)C(=O)N5CCN(CC5)C(=O)C6CC6)c1)C(=O)O | Olaparib 2349 |
| C[C@H](COc1cccc2C(=NNC(=O)c12)Cc3ccc(F)c(c3)C(=O)N4CCN(CC4)C(=O)C5CC5)Nc6ccccc6 | Olaparib 2971 |
| Fc1ccc(CC2=NNC(=O)c3c(OCCC#Cc4cnccn4)cccc23)cc1C(=O)N5CCN(CC5)C(=O)C6CC6 | Olaparib 2979 |
| O[C@@H](COc1cccc2C(=NNC(=O)c12)Cc3ccc(F)c(c3)C(=O)N4CCN(CC4)C(=O)C5CC5)[C@H](O)C=C | Olaparib 2801 |
| CC[C@@H](NC(=O)c1ccc(Oc2cccc3C(=NNC(=O)c23)Cc4ccc(F)c(c4)C(=O)N5CCN(CC5)C(=O)C6CC6)c(C)c1)C(=S)N | Olaparib 2196 |
| CC[C@@](OC(=O)N)(\C=C\Oc1cccc2C(=NNC(=O)c12)Cc3ccc(F)c(c3)C(=O)N4CCN(CC4)C(=O)C5CC5)C#C | Olaparib 2114 |
| CON(C)C(=O)c1ccc(Oc2cccc3C(=NNC(=O)c23)Cc4ccc(F)c(c4)C(=O)N5CCN(CC5)C(=O)C6CC6)c(Cl)c1 | Olaparib 2233 |
| C[C@@H](N)c1cccc(Oc2cccc3C(=NNC(=O)c23)Cc4ccc(F)c(c4)C(=O)N5CCN(CC5)C(=O)C6CC6)c1 | Olaparib 3004 |
| Oc1ccc(cc1Oc2cccc3C(=NNC(=O)c23)Cc4ccc(F)c(c4)C(=O)N5CCN(CC5)C(=O)C6CC6)C#N | Olaparib 2414 |
| CC(C)OCC(=O)Oc1cccc2C(=NNC(=O)c12)Cc3ccc(F)c(c3)C(=O)N4CCN(CC4)C(=O)C5CC5 | Olaparib 1329 |
| Fc1ccc(CC2=NNC(=O)c3c(cccc23)C(=O)OCCNC(=O)c4cccnc4)cc1C(=O)N5CCN(CC5)C(=O)C6CC6 | Olaparib 811 |
| CCC[C@H](NC(=O)c1cccc2C(=NNC(=O)c12)Cc3ccc(F)c(c3)C(=O)N4CCN(CC4)C(=O)C5CC5)C(=O)O | Olaparib 65 |
| Cc1cncc(c1)[C@@H](N)C(=O)Nc2cccc3C(=NNC(=O)c23)Cc4ccc(F)c(c4)C(=O)N5CCN(CC5)C(=O)C6CC6 | Olaparib 502 |
| COc1cccc(CCOc2cccc3C(=NNC(=O)c23)Cc4ccc(F)c(c4)C(=O)N5CCN(CC5)C(=O)C6CC6)c1 | Olaparib 2964 |
| Nc1cnccc1Oc2cccc3C(=NNC(=O)c23)Cc4ccc(F)c(c4)C(=O)N5CCN(CC5)C(=O)C6CC6 | Olaparib 3046 |
| CNC(=O)CCCOc1cccc2C(=NNC(=O)c12)Cc3ccc(F)c(c3)C(=O)N4CCN(CC4)C(=O)C5CC5 | Olaparib 2808 |
| Fc1ccc(cn1)C(=O)NCCOC(=O)c2cccc3C(=NNC(=O)c23)Cc4ccc(F)c(c4)C(=O)N5CCN(CC5)C(=O)C6CC6 | Olaparib 753 |
| CCOC(=O)[C@H](C)NC(=O)c1cccc2C(=NNC(=O)c12)Cc3ccc(F)c(c3)C(=O)N4CCN(CC4)C(=O)C5CC5 | Olaparib 68 |
| Cc1cc(ccn1)[C@@H](N)C(=O)Nc2cccc3C(=NNC(=O)c23)Cc4ccc(F)c(c4)C(=O)N5CCN(CC5)C(=O)C6CC6 | Olaparib 503 |
| C[C@H](O)c1ccc(Oc2cccc3C(=NNC(=O)c23)Cc4ccc(F)c(c4)C(=O)N5CCN(CC5)C(=O)C6CC6)cc1 | Olaparib 3003 |
| Fc1ccc(CC2=NNC(=O)c3c(Oc4ccc(cc4)[N+]#N)cccc23)cc1C(=O)N5CCN(CC5)C(=O)C6CC6 | Olaparib 2348 |
| CC[C@@H](N)C(=O)Oc1cccc2C(=NNC(=O)c12)Cc3ccc(F)c(c3)C(=O)N4CCN(CC4)C(=O)C5CC5 | Olaparib 1387 |
| CS(=O)(=O)Nc1ccc(Oc2cccc3C(=NNC(=O)c23)Cc4ccc(F)c(c4)C(=O)N5CCN(CC5)C(=O)C6CC6)cc1 | Olaparib 2884 |
| CCOC(=O)\C=C(\N)/C(F)(F)Oc1cccc2C(=NNC(=O)c12)Cc3ccc(F)c(c3)C(=O)N4CCN(CC4)C(=O)C5CC5 | Olaparib 2119 |
| Cc1cncc(c1)[C@@H](N)C(=O)Oc2cccc3C(=NNC(=O)c23)Cc4ccc(F)c(c4)C(=O)N5CCN(CC5)C(=O)C6CC6 | Olaparib 1489 |
| OCP(COc1cccc2C(=NNC(=O)c12)Cc3ccc(F)c(c3)C(=O)N4CCN(CC4)C(=O)C5CC5)c6ccccc6 | Olaparib 2918 |
| CCCCN1C=CC(=O)C(=C1C)Oc2cccc3C(=NNC(=O)c23)Cc4ccc(F)c(c4)C(=O)N5CCN(CC5)C(=O)C6CC6 | Olaparib 2895 |
| CN(C)CC(=O)Oc1cccc2C(=NNC(=O)c12)Cc3ccc(F)c(c3)C(=O)N4CCN(CC4)C(=O)C5CC5 | Olaparib 1389 |
| NC(=S)N\N=C\c1ccc(Oc2cccc3C(=NNC(=O)c23)Cc4ccc(F)c(c4)C(=O)N5CCN(CC5)C(=O)C6CC6)cc1 | Olaparib 2878 |
| C[C@@H](N(C)C(=O)C#C)C(=O)Oc1cccc2C(=NNC(=O)c12)Cc3ccc(F)c(c3)C(=O)N4CCN(CC4)C(=O)C5CC5 | Olaparib 1206 |
| Cc1cc(ccn1)[C@@H](N)C(=O)Oc2cccc3C(=NNC(=O)c23)Cc4ccc(F)c(c4)C(=O)N5CCN(CC5)C(=O)C6CC6 | Olaparib 1490 |
| COc1cncc(C)c1c2cccc3C(=NNC(=O)c23)Cc4ccc(F)c(c4)C(=O)N5CCN(CC5)C(=O)C6CC6 | Olaparib 1986 |
| CC1=CC=C[C@H](OC(=O)c2cccc3C(=NNC(=O)c23)Cc4ccc(F)c(c4)C(=O)N5CCN(CC5)C(=O)C6CC6)[C@@H]1O | Olaparib 954 |
| CC[C@@H](O)C(=O)Nc1cccc2C(=NNC(=O)c12)Cc3ccc(F)c(c3)C(=O)N4CCN(CC4)C(=O)C5CC5 | Olaparib 436 |
| C[C@@H](CC#N)N(C)C(=O)c1ccc(Oc2cccc3C(=NNC(=O)c23)Cc4ccc(F)c(c4)C(=O)N5CCN(CC5)C(=O)C6CC6)c(N)c1 | Olaparib 2201 |
| CC(C)(C(=O)O)C(=O)Nc1cccc2C(=NNC(=O)c12)Cc3ccc(F)c(c3)C(=O)N4CCN(CC4)C(=O)C5CC5 | Olaparib 345 |
| Oc1ccc(CC(=O)Oc2cccc3C(=NNC(=O)c23)Cc4ccc(F)c(c4)C(=O)N5CCN(CC5)C(=O)C6CC6)cc1O | Olaparib 1488 |
| OCCc1cccc(Oc2cccc3C(=NNC(=O)c23)Cc4ccc(F)c(c4)C(=O)N5CCN(CC5)C(=O)C6CC6)c1 | Olaparib 3000 |
| CC1=CC(=C(C#N)C(=O)N1)c2cccc3C(=NNC(=O)c23)Cc4ccc(F)c(c4)C(=O)N5CCN(CC5)C(=O)C6CC6 | Olaparib 1714 |
| CC[C@@H](O)C(=O)Oc1cccc2C(=NNC(=O)c12)Cc3ccc(F)c(c3)C(=O)N4CCN(CC4)C(=O)C5CC5 | Olaparib 1383 |
| COc1cc(COC(=O)c2cccc3C(=NNC(=O)c23)Cc4ccc(F)c(c4)C(=O)N5CCN(CC5)C(=O)C6CC6)cc(O)c1OC | Olaparib 752 |
| C[C@H](CNC(=O)c1cccc2C(=NNC(=O)c12)Cc3ccc(F)c(c3)C(=O)N4CCN(CC4)C(=O)C5CC5)C(=O)O | Olaparib 108 |
| N[C@@H](C(=O)Nc1cccc2C(=NNC(=O)c12)Cc3ccc(F)c(c3)C(=O)N4CCN(CC4)C(=O)C5CC5)c6cccnc6 | Olaparib 512 |
| NCc1cccc(Oc2cccc3C(=NNC(=O)c23)Cc4ccc(F)c(c4)C(=O)N5CCN(CC5)C(=O)C6CC6)c1 | Olaparib 3026 |
| CC(C)[C@H]1C\C(=C/C(=O)Oc2cccc3C(=NNC(=O)c23)Cc4ccc(F)c(c4)C(=O)N5CCN(CC5)C(=O)C6CC6)\CCO1 | Olaparib 1472 |
| CCOCC(=O)Nc1cccc2C(=NNC(=O)c12)Cc3ccc(F)c(c3)C(=O)N4CCN(CC4)C(=O)C5CC5 | Olaparib 433 |
| NC[C@](OC(=O)c1cccc2C(=NNC(=O)c12)Cc3ccc(F)c(c3)C(=O)N4CCN(CC4)C(=O)C5CC5)(c6cnccn6)C(F)(F)F | Olaparib 712 |
| COC(=O)[C@H](C)NC(=O)c1cccc2C(=NNC(=O)c12)Cc3ccc(F)c(c3)C(=O)N4CCN(CC4)C(=O)C5CC5 | Olaparib 105 |
| Fc1ccc(CC2=NNC(=O)c3c(cccc23)C(=O)O[C@H](CC(F)(F)F)c4cnccn4)cc1C(=O)N5CCN(CC5)C(=O)C6CC6 | Olaparib 735 |
| CNc1cccc(Oc2cccc3C(=NNC(=O)c23)Cc4ccc(F)c(c4)C(=O)N5CCN(CC5)C(=O)C6CC6)c1 | Olaparib 3028 |
| Fc1ccc(CC2=NNC(=O)c3c(cccc23)C(=O)OCCC(=C)[C@@H]4CCCN4)cc1C(=O)N5CCN(CC5)C(=O)C6CC6 | Olaparib 912 |
| Fc1ccc(CC2=NNC(=O)c3c(OCCNC(=O)C(F)(F)F)cccc23)cc1C(=O)N4CCN(CC4)C(=O)C5CC5 | Olaparib 2753 |
| Cc1cc(OC(=O)c2cccc3C(=NNC(=O)c23)Cc4ccc(F)c(c4)C(=O)N5CCN(CC5)C(=O)C6CC6)nc(O)c1C#N | Olaparib 884 |
| CC[C@@H](NC(=O)c1cccc2C(=NNC(=O)c12)Cc3ccc(F)c(c3)C(=O)N4CCN(CC4)C(=O)C5CC5)C(=O)O | Olaparib 103 |
| Nc1cccc(O)c1OC(=O)c2cccc3C(=NNC(=O)c23)Cc4ccc(F)c(c4)C(=O)N5CCN(CC5)C(=O)C6CC6 | Olaparib 956 |
| Cc1ccc(Oc2cccc3C(=NNC(=O)c23)Cc4ccc(F)c(c4)C(=O)N5CCN(CC5)C(=O)C6CC6)c(O)c1 | Olaparib 3024 |
| Fc1ccc(CC2=NNC(=O)c3c(NC(=O)C[C@@H]4CCC(=O)C4)cccc23)cc1C(=O)N5CCN(CC5)C(=O)C6CC6 | Olaparib 513 |
| CCOCC(=O)Oc1cccc2C(=NNC(=O)c12)Cc3ccc(F)c(c3)C(=O)N4CCN(CC4)C(=O)C5CC5 | Olaparib 1380 |
| Cc1cc(O)nc(OC(=O)c2cccc3C(=NNC(=O)c23)Cc4ccc(F)c(c4)C(=O)N5CCN(CC5)C(=O)C6CC6)c1C#N | Olaparib 883 |
| CN(CC(=O)Nc1cccc2C(=NNC(=O)c12)Cc3ccc(F)c(c3)C(=O)N4CCN(CC4)C(=O)C5CC5)C(=O)C(Cl)(Cl)Cl | Olaparib 271 |
| COC(=O)c1ccnc(Oc2cccc3C(=NNC(=O)c23)Cc4ccc(F)c(c4)C(=O)N5CCN(CC5)C(=O)C6CC6)c1 | Olaparib 2406 |
| OCc1ccc(Oc2cccc3C(=NNC(=O)c23)Cc4ccc(F)c(c4)C(=O)N5CCN(CC5)C(=O)C6CC6)c(Br)c1 | Olaparib 2871 |
| Fc1ccc(CC2=NNC(=O)c3c(OC(=O)C[C@@H]4CCC(=O)C4)cccc23)cc1C(=O)N5CCN(CC5)C(=O)C6CC6 | Olaparib 1510 |
| CCOC(=O)C(Br)(Br)Oc1cccc2C(=NNC(=O)c12)Cc3ccc(F)c(c3)C(=O)N4CCN(CC4)C(=O)C5CC5 | Olaparib 2095 |
| NC(=O)c1nc(F)cnc1Oc2cccc3C(=NNC(=O)c23)Cc4ccc(F)c(c4)C(=O)N5CCN(CC5)C(=O)C6CC6 | Olaparib 2957 |
| OP(=O)CCCNC(=O)c1cccc2C(=NNC(=O)c12)Cc3ccc(F)c(c3)C(=O)N4CCN(CC4)C(=O)C5CC5 | Olaparib 38 |
| Fc1ccc(CC2=NNC(=O)c3c(Oc4c(C=O)c[nH]c4C=O)cccc23)cc1C(=O)N5CCN(CC5)C(=O)C6CC6 | Olaparib 2402 |
| COc1c(Br)nccc1c2cccc3C(=NNC(=O)c23)Cc4ccc(F)c(c4)C(=O)N5CCN(CC5)C(=O)C6CC6 | Olaparib 1956 |
| C[C@H]1OC(=C(Oc2cccc3C(=NNC(=O)c23)Cc4ccc(F)c(c4)C(=O)N5CCN(CC5)C(=O)C6CC6)C1=O)C | Olaparib 3016 |
| NCCNCCNCCCc1cccc2C(=NNC(=O)c12)Cc3ccc(F)c(c3)C(=O)N4CCN(CC4)C(=O)C5CC5 | Olaparib 1800 |
| CC(C)Cn1cc(nn1)C(C)(C)NC(=O)c2cccc3C(=NNC(=O)c23)Cc4ccc(F)c(c4)C(=O)N5CCN(CC5)C(=O)C6CC6 | Olaparib 159 |
| OC(=O)CCCNC(=O)c1cccc2C(=NNC(=O)c12)Cc3ccc(F)c(c3)C(=O)N4CCN(CC4)C(=O)C5CC5 | Olaparib 109 |
| Fc1ccc(CC2=NNC(=O)c3c(OC(=O)c4cncnc4)cccc23)cc1C(=O)N5CCN(CC5)C(=O)C6CC6 | Olaparib 1518 |
| COc1cccc(n1)c2cccc3C(=NNC(=O)c23)Cc4ccc(F)c(c4)C(=O)N5CCN(CC5)C(=O)C6CC6 | Olaparib 1870 |
| C[C@H]1SC(=O)C(=C1Oc2cccc3C(=NNC(=O)c23)Cc4ccc(F)c(c4)C(=O)N5CCN(CC5)C(=O)C6CC6)C | Olaparib 2991 |
| C[C@@H](Oc1cccc2C(=NNC(=O)c12)Cc3ccc(F)c(c3)C(=O)N4CCN(CC4)C(=O)C5CC5)[C@H](N)CO | Olaparib 2833 |
| CC(C)(C)Cc1noc(COC(=O)c2cccc3C(=NNC(=O)c23)Cc4ccc(F)c(c4)C(=O)N5CCN(CC5)C(=O)C6CC6)n1 | Olaparib 792 |
| OC(=O)[C@H](CC#C)NC(=O)c1cccc2C(=NNC(=O)c12)Cc3ccc(F)c(c3)C(=O)N4CCN(CC4)C(=O)C5CC5 | Olaparib 81 |
| Cc1cc(C)n(CC(=O)Oc2cccc3C(=NNC(=O)c23)Cc4ccc(F)c(c4)C(=O)N5CCN(CC5)C(=O)C6CC6)n1 | Olaparib 1504 |
| COc1ncccc1c2cccc3C(=NNC(=O)c23)Cc4ccc(F)c(c4)C(=O)N5CCN(CC5)C(=O)C6CC6 | Olaparib 1860 |
| CC1=CC(=O)N[C@@H]1Oc2cccc3C(=NNC(=O)c23)Cc4ccc(F)c(c4)C(=O)N5CCN(CC5)C(=O)C6CC6 | Olaparib 3043 |
| COC[C@@H](O)COc1cccc2C(=NNC(=O)c12)Cc3ccc(F)c(c3)C(=O)N4CCN(CC4)C(=O)C5CC5 | Olaparib 2824 |
| CN(C)Cc1nc(COC(=O)c2cccc3C(=NNC(=O)c23)Cc4ccc(F)c(c4)C(=O)N5CCN(CC5)C(=O)C6CC6)cs1 | Olaparib 787 |
| CCOC(=O)C(=O)C(F)(F)Oc1cccc2C(=NNC(=O)c12)Cc3ccc(F)c(c3)C(=O)N4CCN(CC4)C(=O)C5CC5 | Olaparib 2131 |
| Cn1ccnc1C(=O)CC(F)(F)Oc2cccc3C(=NNC(=O)c23)Cc4ccc(F)c(c4)C(=O)N5CCN(CC5)C(=O)C6CC6 | Olaparib 2331 |
| Nc1cnc(Cl)c(c1)c2cccc3C(=NNC(=O)c23)Cc4ccc(F)c(c4)C(=O)N5CCN(CC5)C(=O)C6CC6 | Olaparib 1976 |
| CC1=C(Oc2cccc3C(=NNC(=O)c23)Cc4ccc(F)c(c4)C(=O)N5CCN(CC5)C(=O)C6CC6)C(=O)CO1 | Olaparib 3040 |
| COC[C@@H](CO)Oc1cccc2C(=NNC(=O)c12)Cc3ccc(F)c(c3)C(=O)N4CCN(CC4)C(=O)C5CC5 | Olaparib 2823 |
| N[C@H](COC(=O)c1cccc2C(=NNC(=O)c12)Cc3ccc(F)c(c3)C(=O)N4CCN(CC4)C(=O)C5CC5)Cc6c[nH]cn6 | Olaparib 913 |
| CCOC(=O)\C(=N/O)\c1cccc2C(=NNC(=O)c12)Cc3ccc(F)c(c3)C(=O)N4CCN(CC4)C(=O)C5CC5 | Olaparib 1623 |
| COc1cc(Oc2cccc3C(=NNC(=O)c23)Cc4ccc(F)c(c4)C(=O)N5CCN(CC5)C(=O)C6CC6)ncn1 | Olaparib 2418 |
| Oc1ccc(Cl)cc1Oc2cccc3C(=NNC(=O)c23)Cc4ccc(F)c(c4)C(=O)N5CCN(CC5)C(=O)C6CC6 | Olaparib 2986 |
| CC(C)(COC(=O)c1cccc2C(=NNC(=O)c12)Cc3ccc(F)c(c3)C(=O)N4CCN(CC4)C(=O)C5CC5)CN6CCCC(C)(C)C6 | Olaparib 723 |
| CC(=C)\C=C\C(=O)Oc1cccc2C(=NNC(=O)c12)Cc3ccc(F)c(c3)C(=O)N4CCN(CC4)C(=O)C5CC5 | Olaparib 1373 |
| C[C@@H](Cc1nncs1)OC(=O)c2cccc3C(=NNC(=O)c23)Cc4ccc(F)c(c4)C(=O)N5CCN(CC5)C(=O)C6CC6 | Olaparib 901 |
| CC(=O)\C(=N\O)\Oc1cccc2C(=NNC(=O)c12)Cc3ccc(F)c(c3)C(=O)N4CCN(CC4)C(=O)C5CC5 | Olaparib 2163 |
| Nc1ccnc(Oc2cccc3C(=NNC(=O)c23)Cc4ccc(F)c(c4)C(=O)N5CCN(CC5)C(=O)C6CC6)n1 | Olaparib 2428 |
| Oc1cc(Cl)c(Oc2cccc3C(=NNC(=O)c23)Cc4ccc(F)c(c4)C(=O)N5CCN(CC5)C(=O)C6CC6)cc1Cl | Olaparib 2901 |
| C[C@H](COC1C[C@H](C)C[C@H](C)C1)OC(=O)c2cccc3C(=NNC(=O)c23)Cc4ccc(F)c(c4)C(=O)N5CCN(CC5)C(=O)C6CC6 | Olaparib 743 |
| CC[C@H](C)\C=C(/C)\C(=O)Nc1cccc2C(=NNC(=O)c12)Cc3ccc(F)c(c3)C(=O)N4CCN(CC4)C(=O)C5CC5 | Olaparib 319 |
| NCc1onc(OC(=O)c2cccc3C(=NNC(=O)c23)Cc4ccc(F)c(c4)C(=O)N5CCN(CC5)C(=O)C6CC6)c1 | Olaparib 971 |
| COC(OC)[C@H](C)NC(=O)c1cccc2C(=NNC(=O)c12)Cc3ccc(F)c(c3)C(=O)N4CCN(CC4)C(=O)C5CC5 | Olaparib 42 |
| [O-][n+]1ccncc1Oc2cccc3C(=NNC(=O)c23)Cc4ccc(F)c(c4)C(=O)N5CCN(CC5)C(=O)C6CC6 | Olaparib 2427 |
| Fc1ccc(CC2=NNC(=O)c3c2cccc3c4coc(C=O)c4)cc1C(=O)N5CCN(CC5)C(=O)C6CC6 | Olaparib 2023 |
| C[C@H]1CCN(C[C@@H](OC(=O)c2cccc3C(=NNC(=O)c23)Cc4ccc(F)c(c4)C(=O)N5CCN(CC5)C(=O)C6CC6)C(F)(F)F)[C@@H](C)C1 | Olaparib 695 |
| CC(C)(CC=C)C(=O)Nc1cccc2C(=NNC(=O)c12)Cc3ccc(F)c(c3)C(=O)N4CCN(CC4)C(=O)C5CC5 | Olaparib 372 |
| Fc1ccc(CC2=NNC(=O)c3c(cccc23)C(=O)OCCn4cncn4)cc1C(=O)N5CCN(CC5)C(=O)C6CC6 | Olaparib 975 |
| CP(=O)(O)C[C@@H](CN)Oc1cccc2C(=NNC(=O)c12)Cc3ccc(F)c(c3)C(=O)N4CCN(CC4)C(=O)C5CC5 | Olaparib 2754 |
| [O-][n+]1ccnc(Oc2cccc3C(=NNC(=O)c23)Cc4ccc(F)c(c4)C(=O)N5CCN(CC5)C(=O)C6CC6)c1 | Olaparib 2426 |
| Fc1ccc(Oc2cccc3C(=NNC(=O)c23)Cc4ccc(F)c(c4)C(=O)N5CCN(CC5)C(=O)C6CC6)nc1 | Olaparib 2368 |
| C[C@@H]1CCCN(C[C@@H](OC(=O)c2cccc3C(=NNC(=O)c23)Cc4ccc(F)c(c4)C(=O)N5CCN(CC5)C(=O)C6CC6)C(F)(F)F)CC1 | Olaparib 694 |
| CC(C)(CC=C)C(=O)Oc1cccc2C(=NNC(=O)c12)Cc3ccc(F)c(c3)C(=O)N4CCN(CC4)C(=O)C5CC5 | Olaparib 1306 |
| Cc1nnsc1C(=O)Oc2cccc3C(=NNC(=O)c23)Cc4ccc(F)c(c4)C(=O)N5CCN(CC5)C(=O)C6CC6 | Olaparib 1509 |
| C[C@H](OC(=O)N)[C@](F)(Oc1cccc2C(=NNC(=O)c12)Cc3ccc(F)c(c3)C(=O)N4CCN(CC4)C(=O)C5CC5)C(F)F | Olaparib 2112 |
| Fc1ccc(CC2=NNC(=O)c3c(Oc4cnc(nc4)C#N)cccc23)cc1C(=O)N5CCN(CC5)C(=O)C6CC6 | Olaparib 2424 |
| Fc1ccc(CC2=NNC(=O)c3c(Oc4ccc(Br)nc4)cccc23)cc1C(=O)N5CCN(CC5)C(=O)C6CC6 | Olaparib 2367 |
| C[C@H]1CCCN(C[C@H](OC(=O)c2cccc3C(=NNC(=O)c23)Cc4ccc(F)c(c4)C(=O)N5CCN(CC5)C(=O)C6CC6)C(F)(F)F)C1 | Olaparib 708 |
| C\C=C\[C@@H](C)CC(=O)Oc1cccc2C(=NNC(=O)c12)Cc3ccc(F)c(c3)C(=O)N4CCN(CC4)C(=O)C5CC5 | Olaparib 1302 |
| OC1=C(OC(=O)c2cccc3C(=NNC(=O)c23)Cc4ccc(F)c(c4)C(=O)N5CCN(CC5)C(=O)C6CC6)C(=O)C(=CC=C1)O | Olaparib 854 |
| C[C@@H](Oc1cccc2C(=NNC(=O)c12)Cc3ccc(F)c(c3)C(=O)N4CCN(CC4)C(=O)C5CC5)[C@H](O)[C@@H](O)C=O | Olaparib 2762 |
| NCc1onc(Oc2cccc3C(=NNC(=O)c23)Cc4ccc(F)c(c4)C(=O)N5CCN(CC5)C(=O)C6CC6)c1 | Olaparib 3038 |
| Fc1ccc(CC2=NNC(=O)c3c2cccc3c4cncc(c4)C#N)cc1C(=O)N5CCN(CC5)C(=O)C6CC6 | Olaparib 2012 |
| CCCC[C@@]1(CN[C@H]1C)OC(=O)c2cccc3C(=NNC(=O)c23)Cc4ccc(F)c(c4)C(=O)N5CCN(CC5)C(=O)C6CC6 | Olaparib 907 |
| CC(=CCC(=O)Nc1cccc2C(=NNC(=O)c12)Cc3ccc(F)c(c3)C(=O)N4CCN(CC4)C(=O)C5CC5)C | Olaparib 417 |
| CCC1(CC)C(=O)NC=C(COC(=O)c2cccc3C(=NNC(=O)c23)Cc4ccc(F)c(c4)C(=O)N5CCN(CC5)C(=O)C6CC6)C1=O | Olaparib 725 |
| C[C@@H](Oc1cccc2C(=NNC(=O)c12)Cc3ccc(F)c(c3)C(=O)N4CCN(CC4)C(=O)C5CC5)C(=O)NCCO | Olaparib 2766 |
| Fc1ccc(CC2=NNC(=O)c3c(Oc4n[nH]c(Br)n4)cccc23)cc1C(=O)N5CCN(CC5)C(=O)C6CC6 | Olaparib 2352 |
| Fc1ccc(CC2=NNC(=O)c3c2cccc3c4cccnn4)cc1C(=O)N5CCN(CC5)C(=O)C6CC6 | Olaparib 1838 |
| CNC1(CC(=O)Nc2cccc3C(=NNC(=O)c23)Cc4ccc(F)c(c4)C(=O)N5CCN(CC5)C(=O)C6CC6)CCCC1 | Olaparib 507 |
| CC(=CCC(=O)Oc1cccc2C(=NNC(=O)c12)Cc3ccc(F)c(c3)C(=O)N4CCN(CC4)C(=O)C5CC5)C | Olaparib 1360 |
| CCC1=C(O)C(=O)C=CN1CCOC(=O)c2cccc3C(=NNC(=O)c23)Cc4ccc(F)c(c4)C(=O)N5CCN(CC5)C(=O)C6CC6 | Olaparib 756 |
| COC[C@@H](N)C(=O)Nc1cccc2C(=NNC(=O)c12)Cc3ccc(F)c(c3)C(=O)N4CCN(CC4)C(=O)C5CC5 | Olaparib 387 |
| OC1=C(Oc2cccc3C(=NNC(=O)c23)Cc4ccc(F)c(c4)C(=O)N5CCN(CC5)C(=O)C6CC6)C(=O)C(=CC=C1)O | Olaparib 2960 |
| Fc1ccc(CC2=NNC(=O)c3c2cccc3c4cc(Cl)ncn4)cc1C(=O)N5CCN(CC5)C(=O)C6CC6 | Olaparib 1993 |
| CNC1(CC(=O)Oc2cccc3C(=NNC(=O)c23)Cc4ccc(F)c(c4)C(=O)N5CCN(CC5)C(=O)C6CC6)CCCC1 | Olaparib 1496 |
| C\C=C(/Br)\C(=O)Oc1cccc2C(=NNC(=O)c12)Cc3ccc(F)c(c3)C(=O)N4CCN(CC4)C(=O)C5CC5 | Olaparib 1193 |
| CC(=O)C1=C(C[C@@H](O)CC1=O)OC(=O)c2cccc3C(=NNC(=O)c23)Cc4ccc(F)c(c4)C(=O)N5CCN(CC5)C(=O)C6CC6 | Olaparib 793 |
| N[C@H]([C@H](O)C(F)F)C(=O)Nc1cccc2C(=NNC(=O)c12)Cc3ccc(F)c(c3)C(=O)N4CCN(CC4)C(=O)C5CC5 | Olaparib 291 |
| OC1=CC=CC(=C(O)C1=O)Oc2cccc3C(=NNC(=O)c23)Cc4ccc(F)c(c4)C(=O)N5CCN(CC5)C(=O)C6CC6 | Olaparib 2962 |
| Cc1c(cnn1C)c2cccc3C(=NNC(=O)c23)Cc4ccc(F)c(c4)C(=O)N5CCN(CC5)C(=O)C6CC6 | Olaparib 2019 |
| F[C@@H]1CN(CCOC(=O)c2cccc3C(=NNC(=O)c23)Cc4ccc(F)c(c4)C(=O)N5CCN(CC5)C(=O)C6CC6)C[C@H]1F | Olaparib 879 |
| COC(=O)\C(=C(/F)\c1cccc2C(=NNC(=O)c12)Cc3ccc(F)c(c3)C(=O)N4CCN(CC4)C(=O)C5CC5)\F | Olaparib 1634 |
| CN1C(=O)C=C(C)C(=C1OC(=O)c2cccc3C(=NNC(=O)c23)Cc4ccc(F)c(c4)C(=O)N5CCN(CC5)C(=O)C6CC6)C#N | Olaparib 824 |
| OCP(=O)(CO)COc1cccc2C(=NNC(=O)c12)Cc3ccc(F)c(c3)C(=O)N4CCN(CC4)C(=O)C5CC5 | Olaparib 2755 |
| C[C@@H]1CCCCCN1C(=O)\C=C\C(=O)Nc2cccc3C(=NNC(=O)c23)Cc4ccc(F)c(c4)C(=O)N5CCN(CC5)C(=O)C6CC6 | Olaparib 475 |
| CC1=CC[C@H](CC(=O)Oc2cccc3C(=NNC(=O)c23)Cc4ccc(F)c(c4)C(=O)N5CCN(CC5)C(=O)C6CC6)C1(C)C | Olaparib 1486 |
| Fc1ccc(CC2=NNC(=O)c3c(cccc23)C(=O)OC[C@@H]4CCCO4)cc1C(=O)N5CCN(CC5)C(=O)C6CC6 | Olaparib 986 |
| Fc1ccc(CC2=NNC(=O)c3c(OC(=O)C(=C)C(F)(F)F)cccc23)cc1C(=O)N4CCN(CC4)C(=O)C5CC5 | Olaparib 1247 |
| CN1N=CC(=C(Cl)C1=O)OC(=O)c2cccc3C(=NNC(=O)c23)Cc4ccc(F)c(c4)C(=O)N5CCN(CC5)C(=O)C6CC6 | Olaparib 833 |
| N[C@H](CC(=O)O)C(F)(F)Oc1cccc2C(=NNC(=O)c12)Cc3ccc(F)c(c3)C(=O)N4CCN(CC4)C(=O)C5CC5 | Olaparib 2145 |
| CC(C)(C)COC1=C(C(=O)NN=C1)c2cccc3C(=NNC(=O)c23)Cc4ccc(F)c(c4)C(=O)N5CCN(CC5)C(=O)C6CC6 | Olaparib 1672 |
| CC1(CCCC1)C(=O)Oc2cccc3C(=NNC(=O)c23)Cc4ccc(F)c(c4)C(=O)N5CCN(CC5)C(=O)C6CC6 | Olaparib 1515 |
| Fc1ccc(CC2=NNC(=O)c3c(O[C@@H]4CNC(=O)C4)cccc23)cc1C(=O)N5CCN(CC5)C(=O)C6CC6 | Olaparib 3052 |
| Fc1ccc(CC2=NNC(=O)c3c(NC(=O)C(=C)Cl)cccc23)cc1C(=O)N4CCN(CC4)C(=O)C5CC5 | Olaparib 429 |
| Fc1ccc(CC2=NNC(=O)c3c(cccc23)C(=O)OCN4N=CC(=C(Cl)C4=O)Cl)cc1C(=O)N5CCN(CC5)C(=O)C6CC6 | Olaparib 730 |
| O[C@H](CC(=O)O)C(F)(F)Oc1cccc2C(=NNC(=O)c12)Cc3ccc(F)c(c3)C(=O)N4CCN(CC4)C(=O)C5CC5 | Olaparib 2141 |
| CC(C)CCOC1=C(C(=O)NN=C1)c2cccc3C(=NNC(=O)c23)Cc4ccc(F)c(c4)C(=O)N5CCN(CC5)C(=O)C6CC6 | Olaparib 1671 |
| Fc1ccc(CC2=NNC(=O)c3c(\C=C\CN4CCOCC4)cccc23)cc1C(=O)N5CCN(CC5)C(=O)C6CC6 | Olaparib 1716 |
| O[C@@H]1C[C@H](O)CC(C1)Oc2cccc3C(=NNC(=O)c23)Cc4ccc(F)c(c4)C(=O)N5CCN(CC5)C(=O)C6CC6 | Olaparib 3012 |
| CC[C@H](C)C[C@H](C)NC(=O)c1cccc2C(=NNC(=O)c12)Cc3ccc(F)c(c3)C(=O)N4CCN(CC4)C(=O)C5CC5 | Olaparib 76 |
| OC1=C(OC(=O)c2cccc3C(=NNC(=O)c23)Cc4ccc(F)c(c4)C(=O)N5CCN(CC5)C(=O)C6CC6)C(=O)C1=O | Olaparib 974 |
| OC[C@@H](Oc1cccc2C(=NNC(=O)c12)Cc3ccc(F)c(c3)C(=O)N4CCN(CC4)C(=O)C5CC5)[C@H](O)C=O | Olaparib 2791 |
| CCC[C@@H](C)OC1=C(C(=O)NN=C1)c2cccc3C(=NNC(=O)c23)Cc4ccc(F)c(c4)C(=O)N5CCN(CC5)C(=O)C6CC6 | Olaparib 1673 |
| CC1(CC1)C(=O)Nc2cccc3C(=NNC(=O)c23)Cc4ccc(F)c(c4)C(=O)N5CCN(CC5)C(=O)C6CC6 | Olaparib 520 |
| N[C@H]1COC[C@@H]1Oc2cccc3C(=NNC(=O)c23)Cc4ccc(F)c(c4)C(=O)N5CCN(CC5)C(=O)C6CC6 | Olaparib 3051 |
| C[C@H](NC(=O)c1cccc2C(=NNC(=O)c12)Cc3ccc(F)c(c3)C(=O)N4CCN(CC4)C(=O)C5CC5)C(C)(C)C | Olaparib 118 |
| CC(C)N1C(=O)NC(=C(Oc2cccc3C(=NNC(=O)c23)Cc4ccc(F)c(c4)C(=O)N5CCN(CC5)C(=O)C6CC6)C1=O)C | Olaparib 2210 |
| OCC(=O)NCCOc1cccc2C(=NNC(=O)c12)Cc3ccc(F)c(c3)C(=O)N4CCN(CC4)C(=O)C5CC5 | Olaparib 2794 |
| CC[C@@H]1OC(=C(OC(=O)c2cccc3C(=NNC(=O)c23)Cc4ccc(F)c(c4)C(=O)N5CCN(CC5)C(=O)C6CC6)C1=O)C | Olaparib 909 |
| Fc1ccc(CC2=NNC(=O)c3c(OC(=O)CC4CC4)cccc23)cc1C(=O)N5CCN(CC5)C(=O)C6CC6 | Olaparib 1523 |
| O[C@@H]1CNC[C@H]1Oc2cccc3C(=NNC(=O)c23)Cc4ccc(F)c(c4)C(=O)N5CCN(CC5)C(=O)C6CC6 | Olaparib 3050 |
| CC(C)(OCCCN)\C=C\c1cccc2C(=NNC(=O)c12)Cc3ccc(F)c(c3)C(=O)N4CCN(CC4)C(=O)C5CC5 | Olaparib 1802 |
| COC(=O)N1CCC(=C1)C(=O)C(F)(F)Oc2cccc3C(=NNC(=O)c23)Cc4ccc(F)c(c4)C(=O)N5CCN(CC5)C(=O)C6CC6 | Olaparib 2252 |
| OCP(=O)(O)COc1cccc2C(=NNC(=O)c12)Cc3ccc(F)c(c3)C(=O)N4CCN(CC4)C(=O)C5CC5 | Olaparib 2778 |
| CC1=C(Oc2cccc3C(=NNC(=O)c23)Cc4ccc(F)c(c4)C(=O)N5CCN(CC5)C(=O)C6CC6)C(=C(C#N)C(=O)N1)C | Olaparib 2351 |
| Fc1ccc(CC2=NNC(=O)c3c(OC(=O)[C@@H]4C[C@@H]4C(F)(F)F)cccc23)cc1C(=O)N5CCN(CC5)C(=O)C6CC6 | Olaparib 1505 |
| CC(=O)\C=C\c1ccc(Oc2cccc3C(=NNC(=O)c23)Cc4ccc(F)c(c4)C(=O)N5CCN(CC5)C(=O)C6CC6)cc1 | Olaparib 2945 |
| CC(C)C(C)(C)C(=O)Oc1cccc2C(=NNC(=O)c12)Cc3ccc(F)c(c3)C(=O)N4CCN(CC4)C(=O)C5CC5 | Olaparib 1288 |
| CN1N=CC(=C(Oc2cccc3C(=NNC(=O)c23)Cc4ccc(F)c(c4)C(=O)N5CCN(CC5)C(=O)C6CC6)C1=O)N | Olaparib 2398 |
| CN1C(=O)C=C(C)C(=C1Oc2cccc3C(=NNC(=O)c23)Cc4ccc(F)c(c4)C(=O)N5CCN(CC5)C(=O)C6CC6)C#N | Olaparib 2941 |
| CC(C)(C)C[C@H]1C[C@H](CCO1)Oc2cccc3C(=NNC(=O)c23)Cc4ccc(F)c(c4)C(=O)N5CCN(CC5)C(=O)C6CC6 | Olaparib 2913 |
| CC(C)(C)c1ccc(COC(=O)c2cccc3C(=NNC(=O)c23)Cc4ccc(F)c(c4)C(=O)N5CCN(CC5)C(=O)C6CC6)cc1 | Olaparib 821 |
| CCCC(C)(C)C(=O)Nc1cccc2C(=NNC(=O)c12)Cc3ccc(F)c(c3)C(=O)N4CCN(CC4)C(=O)C5CC5 | Olaparib 357 |
| Fc1ccc(CC2=NNC(=O)c3c(OC4=CNC(=S)NC4=O)cccc23)cc1C(=O)N5CCN(CC5)C(=O)C6CC6 | Olaparib 2197 |
| CN1N=CC(=C(C1=O)c2cccc3C(=NNC(=O)c23)Cc4ccc(F)c(c4)C(=O)N5CCN(CC5)C(=O)C6CC6)N | Olaparib 1719 |
| CCCC[C@H]1C[C@H](CCO1)Oc2cccc3C(=NNC(=O)c23)Cc4ccc(F)c(c4)C(=O)N5CCN(CC5)C(=O)C6CC6 | Olaparib 2951 |
| CC(C)(NC(=O)c1cccc2C(=NNC(=O)c12)Cc3ccc(F)c(c3)C(=O)N4CCN(CC4)C(=O)C5CC5)c6ccc(Cl)cc6F | Olaparib 156 |
| CC(C)[C@H](C)CC(=O)Nc1cccc2C(=NNC(=O)c12)Cc3ccc(F)c(c3)C(=O)N4CCN(CC4)C(=O)C5CC5 | Olaparib 356 |
| Fc1ccc(CC2=NNC(=O)c3c(OC4=CNC(=O)NC4=S)cccc23)cc1C(=O)N5CCN(CC5)C(=O)C6CC6 | Olaparib 2416 |
| FCCOC1=C(C(=O)NN=C1)c2cccc3C(=NNC(=O)c23)Cc4ccc(F)c(c4)C(=O)N5CCN(CC5)C(=O)C6CC6 | Olaparib 1693 |
| CC1(CCCc2cccc3C(=NNC(=O)c23)Cc4ccc(F)c(c4)C(=O)N5CCN(CC5)C(=O)C6CC6)OCCO1 | Olaparib 1878 |
| CC(=O)c1cc(C)c(Oc2cccc3C(=NNC(=O)c23)Cc4ccc(F)c(c4)C(=O)N5CCN(CC5)C(=O)C6CC6)c(C)c1 | Olaparib 2940 |
| CC(C)CCCNC(=O)c1cccc2C(=NNC(=O)c12)Cc3ccc(F)c(c3)C(=O)N4CCN(CC4)C(=O)C5CC5 | Olaparib 120 |
| CCC1(C)CCN(CC1)C(=O)N[C@H](C)C(=O)Nc2cccc3C(=NNC(=O)c23)Cc4ccc(F)c(c4)C(=O)N5CCN(CC5)C(=O)C6CC6 | Olaparib 456 |
| Fc1ccc(CC2=NNC(=O)c3c2cccc3C4=C(OCC(F)(F)F)C=NNC4=O)cc1C(=O)N5CCN(CC5)C(=O)C6CC6 | Olaparib 1665 |
| F[C@@H]1CN(CCOc2cccc3C(=NNC(=O)c23)Cc4ccc(F)c(c4)C(=O)N5CCN(CC5)C(=O)C6CC6)C[C@H]1F | Olaparib 2973 |
| CC(C)c1ccccc1OC(=O)c2cccc3C(=NNC(=O)c23)Cc4ccc(F)c(c4)C(=O)N5CCN(CC5)C(=O)C6CC6 | Olaparib 934 |
| CCC(C)(C)CC(=O)Oc1cccc2C(=NNC(=O)c12)Cc3ccc(F)c(c3)C(=O)N4CCN(CC4)C(=O)C5CC5 | Olaparib 1291 |
| CN(CCOC(=O)c1cccc2C(=NNC(=O)c12)Cc3ccc(F)c(c3)C(=O)N4CCN(CC4)C(=O)C5CC5)[C@@H]6CCCNC(=O)C6 | Olaparib 744 |
| Fc1ccc(CC2=NNC(=O)c3c(OC(F)(F)OC4=CNC(=O)C=C4)cccc23)cc1C(=O)N5CCN(CC5)C(=O)C6CC6 | Olaparib 2362 |
| Fc1ccc(CC2=NNC(=O)c3c(CCCCOc4ccccc4)cccc23)cc1C(=O)N5CCN(CC5)C(=O)C6CC6 | Olaparib 1892 |
| CC(=O)CCc1ccc(Oc2cccc3C(=NNC(=O)c23)Cc4ccc(F)c(c4)C(=O)N5CCN(CC5)C(=O)C6CC6)cc1 | Olaparib 2939 |
| CCCC(C)(C)C(=O)Oc1cccc2C(=NNC(=O)c12)Cc3ccc(F)c(c3)C(=O)N4CCN(CC4)C(=O)C5CC5 | Olaparib 1290 |
| CC(=O)N1CCN(C[C@@H](OC(=O)c2cccc3C(=NNC(=O)c23)Cc4ccc(F)c(c4)C(=O)N5CCN(CC5)C(=O)C6CC6)C(F)(F)F)CC1 | Olaparib 689 |
| CN1C(=O)C(=C(Oc2cccc3C(=NNC(=O)c23)Cc4ccc(F)c(c4)C(=O)N5CCN(CC5)C(=O)C6CC6)C1=O)Br | Olaparib 2179 |
| COc1ccc(Cc2cccc3C(=NNC(=O)c23)Cc4ccc(F)c(c4)C(=O)N5CCN(CC5)C(=O)C6CC6)cc1F | Olaparib 1865 |
| CCc1ccccc1COC(=O)c2cccc3C(=NNC(=O)c23)Cc4ccc(F)c(c4)C(=O)N5CCN(CC5)C(=O)C6CC6 | Olaparib 932 |
| CCCC[C@H](C)NC(=O)c1cccc2C(=NNC(=O)c12)Cc3ccc(F)c(c3)C(=O)N4CCN(CC4)C(=O)C5CC5 | Olaparib 119 |
| CCNC(=O)[C@H](C)N1C[C@@H](CC1=O)Oc2cccc3C(=NNC(=O)c23)Cc4ccc(F)c(c4)C(=O)N5CCN(CC5)C(=O)C6CC6 | Olaparib 2872 |
| CC1=C(Oc2cccc3C(=NNC(=O)c23)Cc4ccc(F)c(c4)C(=O)N5CCN(CC5)C(=O)C6CC6)N=C(Cl)C(=O)O1 | Olaparib 2360 |
| Fc1ccc(CCOc2cccc3C(=NNC(=O)c23)Cc4ccc(F)c(c4)C(=O)N5CCN(CC5)C(=O)C6CC6)c(Br)c1 | Olaparib 2866 |
| C[C@H](OC(=O)c1cccc2C(=NNC(=O)c12)Cc3ccc(F)c(c3)C(=O)N4CCN(CC4)C(=O)C5CC5)c6ccc(F)cc6 | Olaparib 916 |
| CC[C@@H](C)CC(=O)Nc1cccc2C(=NNC(=O)c12)Cc3ccc(F)c(c3)C(=O)N4CCN(CC4)C(=O)C5CC5 | Olaparib 405 |
| N[C@H]1CCCN(CCCOC(=O)c2cccc3C(=NNC(=O)c23)Cc4ccc(F)c(c4)C(=O)N5CCN(CC5)C(=O)C6CC6)C1=O | Olaparib 788 |
| Fc1ccc(CC2=NNC(=O)c3c(OC4=CNC(=O)N=C4)cccc23)cc1C(=O)N5CCN(CC5)C(=O)C6CC6 | Olaparib 2371 |
| CCc1cccc(n1)c2cccc3C(=NNC(=O)c23)Cc4ccc(F)c(c4)C(=O)N5CCN(CC5)C(=O)C6CC6 | Olaparib 2009 |
| C[C@@H](OC(=O)c1cccc2C(=NNC(=O)c12)Cc3ccc(F)c(c3)C(=O)N4CCN(CC4)C(=O)C5CC5)c6ccccc6Cl | Olaparib 849 |
| CCC[C@H](C)C(=O)Oc1cccc2C(=NNC(=O)c12)Cc3ccc(F)c(c3)C(=O)N4CCN(CC4)C(=O)C5CC5 | Olaparib 1343 |
| CO[C@H]1O[C@](OC)(C=C1)[C@H](C)OC(=O)c2cccc3C(=NNC(=O)c23)Cc4ccc(F)c(c4)C(=O)N5CCN(CC5)C(=O)C6CC6 | Olaparib 780 |
| CC1(C)OC[C@@H](O1)[C@H](OC(=O)c2cccc3C(=NNC(=O)c23)Cc4ccc(F)c(c4)C(=O)N5CCN(CC5)C(=O)C6CC6)C=C | Olaparib 844 |
| Cc1ccnc(c1)c2cccc3C(=NNC(=O)c23)Cc4ccc(F)c(c4)C(=O)N5CCN(CC5)C(=O)C6CC6 | Olaparib 2027 |
| C[C@H](OC(=O)c1cccc2C(=NNC(=O)c12)Cc3ccc(F)c(c3)C(=O)N4CCN(CC4)C(=O)C5CC5)C#Cc6ccccc6 | Olaparib 894 |
| CC[C@@H](C)CC(=O)Oc1cccc2C(=NNC(=O)c12)Cc3ccc(F)c(c3)C(=O)N4CCN(CC4)C(=O)C5CC5 | Olaparib 1344 |
| C[C@@H](COC(=O)c1cccc2C(=NNC(=O)c12)Cc3ccc(F)c(c3)C(=O)N4CCN(CC4)C(=O)C5CC5)NC(=O)[C@H]6CCSC6 | Olaparib 739 |
| Fc1ccc(CC2=NNC(=O)c3c(OC(=O)CON=C4CCCCCCC4)cccc23)cc1C(=O)N5CCN(CC5)C(=O)C6CC6 | Olaparib 1456 |
| Fc1cc(F)cc(Oc2cccc3C(=NNC(=O)c23)Cc4ccc(F)c(c4)C(=O)N5CCN(CC5)C(=O)C6CC6)c1 | Olaparib 2330 |
| Fc1ccc(CCOC(=O)c2cccc3C(=NNC(=O)c23)Cc4ccc(F)c(c4)C(=O)N5CCN(CC5)C(=O)C6CC6)c(Br)c1 | Olaparib 699 |
| CC(C)(C)C(=O)Oc1cccc2C(=NNC(=O)c12)Cc3ccc(F)c(c3)C(=O)N4CCN(CC4)C(=O)C5CC5 | Olaparib 1391 |
| Fc1ccc(CC2=NNC(=O)c3c(cccc23)C(=O)OCCCC(=O)N4CCOCC4)cc1C(=O)N5CCN(CC5)C(=O)C6CC6 | Olaparib 785 |
| Fc1ccc(CC2=NNC(=O)c3c(cccc23)C(=O)OC[C@H]4CCC(=O)N4)cc1C(=O)N5CCN(CC5)C(=O)C6CC6 | Olaparib 970 |
| Fc1cccc(F)c1Oc2cccc3C(=NNC(=O)c23)Cc4ccc(F)c(c4)C(=O)N5CCN(CC5)C(=O)C6CC6 | Olaparib 3015 |
| C\C=C(/C#N)\c1ccc(Oc2cccc3C(=NNC(=O)c23)Cc4ccc(F)c(c4)C(=O)N5CCN(CC5)C(=O)C6CC6)cc1 | Olaparib 2396 |
| CCC[C@H](C(=O)O)c1cccc2C(=NNC(=O)c12)Cc3ccc(F)c(c3)C(=O)N4CCN(CC4)C(=O)C5CC5 | Olaparib 1641 |
| C[C@H](CN1CCNC1=O)C(=O)Oc2cccc3C(=NNC(=O)c23)Cc4ccc(F)c(c4)C(=O)N5CCN(CC5)C(=O)C6CC6 | Olaparib 1482 |
| O[C@H]1OC(=O)C(=C1Oc2cccc3C(=NNC(=O)c23)Cc4ccc(F)c(c4)C(=O)N5CCN(CC5)C(=O)C6CC6)Cl | Olaparib 2387 |
| Fc1ccc(CC2=NNC(=O)c3c2cccc3c4cccc(Br)n4)cc1C(=O)N5CCN(CC5)C(=O)C6CC6 | Olaparib 1962 |
| Fc1ccc(CC2=NNC(=O)c3c(Oc4ccc(CCC=O)cc4)cccc23)cc1C(=O)N5CCN(CC5)C(=O)C6CC6 | Olaparib 2975 |
| CC(F)(F)CC(=O)Nc1cccc2C(=NNC(=O)c12)Cc3ccc(F)c(c3)C(=O)N4CCN(CC4)C(=O)C5CC5 | Olaparib 380 |
| O[C@@H]1C=CO[C@H](COC(=O)c2cccc3C(=NNC(=O)c23)Cc4ccc(F)c(c4)C(=O)N5CCN(CC5)C(=O)C6CC6)[C@H]1O | Olaparib 896 |
| Fc1ccc(CC2=NNC(=O)c3c(OC4=C(Cl)OC(=O)O4)cccc23)cc1C(=O)N5CCN(CC5)C(=O)C6CC6 | Olaparib 2408 |
| Fc1ccc(CC2=NNC(=O)c3c2cccc3c4cccnc4)cc1C(=O)N5CCN(CC5)C(=O)C6CC6 | Olaparib 1839 |
| OC\C=C\c1ccc(Oc2cccc3C(=NNC(=O)c23)Cc4ccc(F)c(c4)C(=O)N5CCN(CC5)C(=O)C6CC6)cc1 | Olaparib 2974 |
| CC(Cl)(Cl)C(=O)Nc1cccc2C(=NNC(=O)c12)Cc3ccc(F)c(c3)C(=O)N4CCN(CC4)C(=O)C5CC5 | Olaparib 317 |
| Fc1ccc(CC2=NNC(=O)c3c(cccc23)C(=O)OCCN4CCNC4=O)cc1C(=O)N5CCN(CC5)C(=O)C6CC6 | Olaparib 948 |
| C[C@@H]1CNC[C@H](C)N1C[C@@H](OC(=O)c2cccc3C(=NNC(=O)c23)Cc4ccc(F)c(c4)C(=O)N5CCN(CC5)C(=O)C6CC6)C(F)(F)F | Olaparib 693 |
| Fc1ccc(CC2=NNC(=O)c3c2cccc3c4ccncc4)cc1C(=O)N5CCN(CC5)C(=O)C6CC6 | Olaparib 1840 |
| Fc1ccc(CC2=NNC(=O)c3c(cccc23)C(=O)OCc4ccccc4Cl)cc1C(=O)N5CCN(CC5)C(=O)C6CC6 | Olaparib 908 |
| NOC\C=C\c1cccc2C(=NNC(=O)c12)Cc3ccc(F)c(c3)C(=O)N4CCN(CC4)C(=O)C5CC5 | Olaparib 1642 |
| CC1(C)C[C@](CCO1)(NCCOC(=O)c2cccc3C(=NNC(=O)c23)Cc4ccc(F)c(c4)C(=O)N5CCN(CC5)C(=O)C6CC6)C#N | Olaparib 724 |
| C[C@@H](C(=O)Oc1cccc2C(=NNC(=O)c12)Cc3ccc(F)c(c3)C(=O)N4CCN(CC4)C(=O)C5CC5)[C@@]6(O)CCOC(C)(C)C6 | Olaparib 1450 |
| Fc1ccc(CC2=NNC(=O)c3c2cccc3c4cccs4)cc1C(=O)N5CCN(CC5)C(=O)C6CC6 | Olaparib 1836 |
| NC(=O)c1ccccc1c2cccc3C(=NNC(=O)c23)Cc4ccc(F)c(c4)C(=O)N5CCN(CC5)C(=O)C6CC6 | Olaparib 1988 |
| NCCCCCCNCCCc1cccc2C(=NNC(=O)c12)Cc3ccc(F)c(c3)C(=O)N4CCN(CC4)C(=O)C5CC5 | Olaparib 1797 |
| CC1(C)O[C@H](CO)[C@@H](COC(=O)c2cccc3C(=NNC(=O)c23)Cc4ccc(F)c(c4)C(=O)N5CCN(CC5)C(=O)C6CC6)O1 | Olaparib 829 |
| CC(C)C[C@@]1(C)OC[C@@H](COC(=O)c2cccc3C(=NNC(=O)c23)Cc4ccc(F)c(c4)C(=O)N5CCN(CC5)C(=O)C6CC6)O1 | Olaparib 778 |
| Fc1ccc(CC2=NNC(=O)c3c2cccc3C4=C(C=O)C=CCC4)cc1C(=O)N5CCN(CC5)C(=O)C6CC6 | Olaparib 2006 |
| CCC(C)(C)c1ccc(Oc2cccc3C(=NNC(=O)c23)Cc4ccc(F)c(c4)C(=O)N5CCN(CC5)C(=O)C6CC6)c(N)c1 | Olaparib 2896 |
| C[C@@H](Oc1cccc2C(=NNC(=O)c12)Cc3ccc(F)c(c3)C(=O)N4CCN(CC4)C(=O)C5CC5)C(C)(C)CO | Olaparib 2796 |
| C[C@@H]1NC[C@@H](O)[C@@H](OC(=O)c2cccc3C(=NNC(=O)c23)Cc4ccc(F)c(c4)C(=O)N5CCN(CC5)C(=O)C6CC6)[C@@H]1O | Olaparib 889 |
| CC[C@H]1C[C@](N)(CNC(=O)c2cccc3C(=NNC(=O)c23)Cc4ccc(F)c(c4)C(=O)N5CCN(CC5)C(=O)C6CC6)CCO1 | Olaparib 177 |
| Fc1ccc(CC2=NNC(=O)c3c2cccc3C4=CCCC4=O)cc1C(=O)N5CCN(CC5)C(=O)C6CC6 | Olaparib 2040 |
| CC(C)(N)Cc1ccc(Oc2cccc3C(=NNC(=O)c23)Cc4ccc(F)c(c4)C(=O)N5CCN(CC5)C(=O)C6CC6)cc1 | Olaparib 2935 |
| CC[C@H](C)[C@H](N)COc1cccc2C(=NNC(=O)c12)Cc3ccc(F)c(c3)C(=O)N4CCN(CC4)C(=O)C5CC5 | Olaparib 2802 |
| C[C@H]1NC[C@H](O)[C@@H](OC(=O)c2cccc3C(=NNC(=O)c23)Cc4ccc(F)c(c4)C(=O)N5CCN(CC5)C(=O)C6CC6)[C@H]1O | Olaparib 887 |
| CCOC(=O)[C@]1(C)CNCC[C@]1(F)Oc2cccc3C(=NNC(=O)c23)Cc4ccc(F)c(c4)C(=O)N5CCN(CC5)C(=O)C6CC6 | Olaparib 2285 |
| Fc1ccc(CC2=NNC(=O)c3c(cccc23)C4=CCNCC4)cc1C(=O)N5CCN(CC5)C(=O)C6CC6 | Olaparib 2035 |
| CN(C)c1ccc(CCOc2cccc3C(=NNC(=O)c23)Cc4ccc(F)c(c4)C(=O)N5CCN(CC5)C(=O)C6CC6)cc1 | Olaparib 2934 |
| CN(CCO)CCCc1cccc2C(=NNC(=O)c12)Cc3ccc(F)c(c3)C(=O)N4CCN(CC4)C(=O)C5CC5 | Olaparib 1811 |
| C[C@H]1N[C@H](CO)[C@@H](OC(=O)c2cccc3C(=NNC(=O)c23)Cc4ccc(F)c(c4)C(=O)N5CCN(CC5)C(=O)C6CC6)[C@@H]1O | Olaparib 890 |
| CC1(COC(=O)c2cccc3C(=NNC(=O)c23)Cc4ccc(F)c(c4)C(=O)N5CCN(CC5)C(=O)C6CC6)COC(C)(C)OC1 | Olaparib 837 |
| Fc1ccc(CC2=NNC(=O)c3c(cccc23)C4=CCCNC4)cc1C(=O)N5CCN(CC5)C(=O)C6CC6 | Olaparib 2036 |
| Cc1ccc(cc1Oc2cccc3C(=NNC(=O)c23)Cc4ccc(F)c(c4)C(=O)N5CCN(CC5)C(=O)C6CC6)C(C)(C)C#N | Olaparib 2907 |
| NC\C=C\Cc1cccc2C(=NNC(=O)c12)Cc3ccc(F)c(c3)C(=O)N4CCN(CC4)C(=O)C5CC5 | Olaparib 1945 |
| C[C@H]1N[C@H](COC(=O)c2cccc3C(=NNC(=O)c23)Cc4ccc(F)c(c4)C(=O)N5CCN(CC5)C(=O)C6CC6)[C@H](O)[C@@H]1O | Olaparib 891 |
| C[C@H]1CNCC[C@@H]1OCCOC(=O)c2cccc3C(=NNC(=O)c23)Cc4ccc(F)c(c4)C(=O)N5CCN(CC5)C(=O)C6CC6 | Olaparib 838 |
| Fc1ccc(CC2=NNC(=O)c3c2cccc3C4=CCCCO4)cc1C(=O)N5CCN(CC5)C(=O)C6CC6 | Olaparib 2034 |
| C[C@@H](O)CCc1ccc(Oc2cccc3C(=NNC(=O)c23)Cc4ccc(F)c(c4)C(=O)N5CCN(CC5)C(=O)C6CC6)cc1 | Olaparib 2929 |
| CC(C)CC(C)(C)Oc1cccc2C(=NNC(=O)c12)Cc3ccc(F)c(c3)C(=O)N4CCN(CC4)C(=O)C5CC5 | Olaparib 2810 |
| O[C@H]1CN[C@H](COC(=O)c2cccc3C(=NNC(=O)c23)Cc4ccc(F)c(c4)C(=O)N5CCN(CC5)C(=O)C6CC6)[C@@H](O)C1 | Olaparib 892 |
| COC(=O)[C@]1(C)CCNC[C@]1(F)Oc2cccc3C(=NNC(=O)c23)Cc4ccc(F)c(c4)C(=O)N5CCN(CC5)C(=O)C6CC6 | Olaparib 2326 |
| Fc1ccc(CC2=NNC(=O)c3c(CCCN4CCCC4)cccc23)cc1C(=O)N5CCN(CC5)C(=O)C6CC6 | Olaparib 1995 |
| C[C@H](N)Cc1ccc(Oc2cccc3C(=NNC(=O)c23)Cc4ccc(F)c(c4)C(=O)N5CCN(CC5)C(=O)C6CC6)cc1 | Olaparib 2970 |
| CC[C@H](C)[C@H](C)COc1cccc2C(=NNC(=O)c12)Cc3ccc(F)c(c3)C(=O)N4CCN(CC4)C(=O)C5CC5 | Olaparib 2811 |
| OC[C@H]1NC[C@H](C[C@@H]1O)OC(=O)c2cccc3C(=NNC(=O)c23)Cc4ccc(F)c(c4)C(=O)N5CCN(CC5)C(=O)C6CC6 | Olaparib 893 |
| CN[C@]1(CCCOC1)C(=O)Nc2cccc3C(=NNC(=O)c23)Cc4ccc(F)c(c4)C(=O)N5CCN(CC5)C(=O)C6CC6 | Olaparib 504 |
| C[C@@H]1CCCC[C@H]1Oc2cccc3C(=NNC(=O)c23)Cc4ccc(F)c(c4)C(=O)N5CCN(CC5)C(=O)C6CC6 | Olaparib 3037 |
| CCCCOc1ccc(Oc2cccc3C(=NNC(=O)c23)Cc4ccc(F)c(c4)C(=O)N5CCN(CC5)C(=O)C6CC6)cc1 | Olaparib 2346 |
| CCC(CC)[C@@H](C)Oc1cccc2C(=NNC(=O)c12)Cc3ccc(F)c(c3)C(=O)N4CCN(CC4)C(=O)C5CC5 | Olaparib 2812 |
| OC[C@H]1NC[C@H](O)[C@@H]1OC(=O)c2cccc3C(=NNC(=O)c23)Cc4ccc(F)c(c4)C(=O)N5CCN(CC5)C(=O)C6CC6 | Olaparib 939 |
| CN[C@]1(CCCOC1)C(=O)Oc2cccc3C(=NNC(=O)c23)Cc4ccc(F)c(c4)C(=O)N5CCN(CC5)C(=O)C6CC6 | Olaparib 1493 |
| Fc1ccc(CC2=NNC(=O)c3c(cccc23)[C@H]4CCCOC4)cc1C(=O)N5CCN(CC5)C(=O)C6CC6 | Olaparib 2028 |
| NCCCc1ccc(Oc2cccc3C(=NNC(=O)c23)Cc4ccc(F)c(c4)C(=O)N5CCN(CC5)C(=O)C6CC6)cc1 | Olaparib 2968 |
| COCCCc1cccc2C(=NNC(=O)c12)Cc3ccc(F)c(c3)C(=O)N4CCN(CC4)C(=O)C5CC5 | Olaparib 1828 |
| OC[C@@H]1NC[C@@H](OC(=O)c2cccc3C(=NNC(=O)c23)Cc4ccc(F)c(c4)C(=O)N5CCN(CC5)C(=O)C6CC6)[C@H]1O | Olaparib 943 |
| Fc1ccc(CC2=NNC(=O)c3c(cccc23)C(=O)OC[C@H]4CNCCO4)cc1C(=O)N5CCN(CC5)C(=O)C6CC6 | Olaparib 967 |
| Fc1ccc(Cl)c(Cc2cccc3C(=NNC(=O)c23)Cc4ccc(F)c(c4)C(=O)N5CCN(CC5)C(=O)C6CC6)c1 | Olaparib 1864 |
| COc1ccc(Cc2cccc3C(=NNC(=O)c23)Cc4ccc(F)c(c4)C(=O)N5CCN(CC5)C(=O)C6CC6)cc1OC | Olaparib 1872 |
| CC(C)CC\C=C\c1cccc2C(=NNC(=O)c12)Cc3ccc(F)c(c3)C(=O)N4CCN(CC4)C(=O)C5CC5 | Olaparib 1926 |
| O[C@H]1CN[C@H](COC(=O)c2cccc3C(=NNC(=O)c23)Cc4ccc(F)c(c4)C(=O)N5CCN(CC5)C(=O)C6CC6)[C@H]1O | Olaparib 940 |
| Fc1ccc(CC2=NNC(=O)c3c(OCCN4CCNC4=O)cccc23)cc1C(=O)N5CCN(CC5)C(=O)C6CC6 | Olaparib 3014 |
| Fc1ccc(CC2=NNC(=O)c3c(Cc4ccccc4Cl)cccc23)cc1C(=O)N5CCN(CC5)C(=O)C6CC6 | Olaparib 1879 |
| COc1cc(Cc2cccc3C(=NNC(=O)c23)Cc4ccc(F)c(c4)C(=O)N5CCN(CC5)C(=O)C6CC6)cc(OC)c1 | Olaparib 1861 |
| CC(=C(C)c1cccc2C(=NNC(=O)c12)Cc3ccc(F)c(c3)C(=O)N4CCN(CC4)C(=O)C5CC5)C | Olaparib 1948 |
| OC[C@H]1NC[C@@H](OC(=O)c2cccc3C(=NNC(=O)c23)Cc4ccc(F)c(c4)C(=O)N5CCN(CC5)C(=O)C6CC6)[C@@H]1O | Olaparib 941 |
| NC[C@H](N1CCOCC1)C(F)(F)Oc2cccc3C(=NNC(=O)c23)Cc4ccc(F)c(c4)C(=O)N5CCN(CC5)C(=O)C6CC6 | Olaparib 2319 |
| Fc1ccc(CC2=NNC(=O)c3c(cccc23)C4=CCCCC4)cc1C(=O)N5CCN(CC5)C(=O)C6CC6 | Olaparib 2037 |
| CC[C@H](O)c1ccc(Oc2cccc3C(=NNC(=O)c23)Cc4ccc(F)c(c4)C(=O)N5CCN(CC5)C(=O)C6CC6)cc1 | Olaparib 2965 |
| CCC(CC)Cc1cccc2C(=NNC(=O)c12)Cc3ccc(F)c(c3)C(=O)N4CCN(CC4)C(=O)C5CC5 | Olaparib 1852 |
| O[C@@H]1CN[C@@H](COC(=O)c2cccc3C(=NNC(=O)c23)Cc4ccc(F)c(c4)C(=O)N5CCN(CC5)C(=O)C6CC6)[C@@H]1O | Olaparib 942 |
| CCCC[C@H](CC)c1cccc2C(=NNC(=O)c12)Cc3ccc(F)c(c3)C(=O)N4CCN(CC4)C(=O)C5CC5 | Olaparib 1900 |
| Fc1ccc(CC2=NNC(=O)c3c(\C=C\C4CC4)cccc23)cc1C(=O)N5CCN(CC5)C(=O)C6CC6 | Olaparib 2046 |
| CC(C)(C#N)c1ccc(cc1COC(=O)c2cccc3C(=NNC(=O)c23)Cc4ccc(F)c(c4)C(=O)N5CCN(CC5)C(=O)C6CC6)C(F)(F)F | Olaparib 687 |
| OC[C@@H](Cc1ccccc1)Oc2cccc3C(=NNC(=O)c23)Cc4ccc(F)c(c4)C(=O)N5CCN(CC5)C(=O)C6CC6 | Olaparib 2966 |
| CCC[C@H](CC)c1cccc2C(=NNC(=O)c12)Cc3ccc(F)c(c3)C(=O)N4CCN(CC4)C(=O)C5CC5 | Olaparib 1849 |
| O[C@@H]1C[C@H](O)[C@@H](COC(=O)c2cccc3C(=NNC(=O)c23)Cc4ccc(F)c(c4)C(=O)N5CCN(CC5)C(=O)C6CC6)O1 | Olaparib 937 |
| Fc1ccc(CC2=NNC(=O)c3c(Oc4ccc(cc4Cl)C(=O)NCCC#C)cccc23)cc1C(=O)N5CCN(CC5)C(=O)C6CC6 | Olaparib 2863 |
| Fc1ccc(CC2=NNC(=O)c3c(cccc23)C4=CCCC4)cc1C(=O)N5CCN(CC5)C(=O)C6CC6 | Olaparib 1884 |
| COc1cc(C)cc(Oc2cccc3C(=NNC(=O)c23)Cc4ccc(F)c(c4)C(=O)N5CCN(CC5)C(=O)C6CC6)c1 | Olaparib 3002 |
| CC(C)CCc1cccc2C(=NNC(=O)c12)Cc3ccc(F)c(c3)C(=O)N4CCN(CC4)C(=O)C5CC5 | Olaparib 1853 |
| OC[C@H]1O[C@@H](C[C@@H]1O)OC(=O)c2cccc3C(=NNC(=O)c23)Cc4ccc(F)c(c4)C(=O)N5CCN(CC5)C(=O)C6CC6 | Olaparib 938 |
| CC(C)(NC[C@@H](O)c1cc(Oc2cccc3C(=NNC(=O)c23)Cc4ccc(F)c(c4)C(=O)N5CCN(CC5)C(=O)C6CC6)ccc1Cl)C#C | Olaparib 2177 |
| Fc1ccc(CC2=NNC(=O)c3c(CC4CCCC4)cccc23)cc1C(=O)N5CCN(CC5)C(=O)C6CC6 | Olaparib 2033 |
| CSc1ccc(Oc2cccc3C(=NNC(=O)c23)Cc4ccc(F)c(c4)C(=O)N5CCN(CC5)C(=O)C6CC6)cc1C | Olaparib 2959 |
| Fc1ccc(CC2=NNC(=O)c3c2cccc3C(F)(F)F)cc1C(=O)N4CCN(CC4)C(=O)C5CC5 | Olaparib 1918 |
| OC[C@H]1O[C@H](O)C[C@@H]1OC(=O)c2cccc3C(=NNC(=O)c23)Cc4ccc(F)c(c4)C(=O)N5CCN(CC5)C(=O)C6CC6 | Olaparib 936 |
| CC(C)(C#N)c1cc(Br)c(F)c(Oc2cccc3C(=NNC(=O)c23)Cc4ccc(F)c(c4)C(=O)N5CCN(CC5)C(=O)C6CC6)c1O | Olaparib 2170 |
| Fc1ccc(CC2=NNC(=O)c3c(cccc23)C4CCCCC4)cc1C(=O)N5CCN(CC5)C(=O)C6CC6 | Olaparib 1835 |
| Nc1ccccc1CCOc2cccc3C(=NNC(=O)c23)Cc4ccc(F)c(c4)C(=O)N5CCN(CC5)C(=O)C6CC6 | Olaparib 3005 |
| CC(C)(\C(=C\C#N)\c1cccc2C(=NNC(=O)c12)Cc3ccc(F)c(c3)C(=O)N4CCN(CC4)C(=O)C5CC5)S(=O)(=O)C | Olaparib 1605 |
| O[C@H]1OC[C@@H](OC(=O)c2cccc3C(=NNC(=O)c23)Cc4ccc(F)c(c4)C(=O)N5CCN(CC5)C(=O)C6CC6)[C@H]1O | Olaparib 964 |
| CC(C)(C#N)c1cc(Br)c(Oc2cccc3C(=NNC(=O)c23)Cc4ccc(F)c(c4)C(=O)N5CCN(CC5)C(=O)C6CC6)c(F)c1O | Olaparib 2171 |
| Cc1ccc(N)cc1Oc2cccc3C(=NNC(=O)c23)Cc4ccc(F)c(c4)C(=O)N5CCN(CC5)C(=O)C6CC6 | Olaparib 3025 |
| CC[C@H](C)NC(=S)NC(=O)c1cccc2C(=NNC(=O)c12)Cc3ccc(F)c(c3)C(=O)N4CCN(CC4)C(=O)C5CC5 | Olaparib 24 |
| O[C@H]1CO[C@H](OC(=O)c2cccc3C(=NNC(=O)c23)Cc4ccc(F)c(c4)C(=O)N5CCN(CC5)C(=O)C6CC6)[C@@H]1O | Olaparib 965 |
| Nc1ccc(SC(F)(F)Oc2cccc3C(=NNC(=O)c23)Cc4ccc(F)c(c4)C(=O)N5CCN(CC5)C(=O)C6CC6)cc1 | Olaparib 2328 |
| Cc1ccc(Oc2cccc3C(=NNC(=O)c23)Cc4ccc(F)c(c4)C(=O)N5CCN(CC5)C(=O)C6CC6)c(N)c1 | Olaparib 3027 |
| CN(C)CC(=S)NC(=O)c1cccc2C(=NNC(=O)c12)Cc3ccc(F)c(c3)C(=O)N4CCN(CC4)C(=O)C5CC5 | Olaparib 52 |
| O[C@@H]1OCCO[C@H]1OC(=O)c2cccc3C(=NNC(=O)c23)Cc4ccc(F)c(c4)C(=O)N5CCN(CC5)C(=O)C6CC6 | Olaparib 963 |
| Fc1ccc(cc1)C(=O)\C=C\C(Cl)(Cl)Oc2cccc3C(=NNC(=O)c23)Cc4ccc(F)c(c4)C(=O)N5CCN(CC5)C(=O)C6CC6 | Olaparib 2182 |
| Cc1cccc(O)c1Oc2cccc3C(=NNC(=O)c23)Cc4ccc(F)c(c4)C(=O)N5CCN(CC5)C(=O)C6CC6 | Olaparib 3021 |
| CC(C)NC(=S)NC(=O)c1cccc2C(=NNC(=O)c12)Cc3ccc(F)c(c3)C(=O)N4CCN(CC4)C(=O)C5CC5 | Olaparib 51 |
| CO[C@@H]1O[C@@H](C)[C@@H](O)[C@@H](O)[C@H]1Oc2cccc3C(=NNC(=O)c23)Cc4ccc(F)c(c4)C(=O)N5CCN(CC5)C(=O)C6CC6 | Olaparib 2903 |
| CC(C)[C@@H]1CC[C@@H](C)C[C@H]1OC(=O)c2cccc3C(=NNC(=O)c23)Cc4ccc(F)c(c4)C(=O)N5CCN(CC5)C(=O)C6CC6 | Olaparib 850 |
| Cc1ccc(O)c(Oc2cccc3C(=NNC(=O)c23)Cc4ccc(F)c(c4)C(=O)N5CCN(CC5)C(=O)C6CC6)c1 | Olaparib 3023 |
| CCCNC(=S)NC(=O)c1cccc2C(=NNC(=O)c12)Cc3ccc(F)c(c3)C(=O)N4CCN(CC4)C(=O)C5CC5 | Olaparib 53 |
| CO[C@@H]1O[C@@H](C)[C@@H](O)[C@H](Oc2cccc3C(=NNC(=O)c23)Cc4ccc(F)c(c4)C(=O)N5CCN(CC5)C(=O)C6CC6)[C@@H]1O | Olaparib 2904 |
| Fc1ccc(Br)c(Cc2cccc3C(=NNC(=O)c23)Cc4ccc(F)c(c4)C(=O)N5CCN(CC5)C(=O)C6CC6)c1 | Olaparib 1954 |
| Fc1ccc(CC2=NNC(=O)c3c2cccc3c4ccnc(OCC(F)(F)F)c4)cc1C(=O)N5CCN(CC5)C(=O)C6CC6 | Olaparib 1958 |
| CC(=O)SCCNC(=O)c1cccc2C(=NNC(=O)c12)Cc3ccc(F)c(c3)C(=O)N4CCN(CC4)C(=O)C5CC5 | Olaparib 41 |
| CO[C@H]1OC[C@H](O)[C@H](O)[C@H]1Oc2cccc3C(=NNC(=O)c23)Cc4ccc(F)c(c4)C(=O)N5CCN(CC5)C(=O)C6CC6 | Olaparib 2943 |
| Fc1ccc(Cc2cccc3C(=NNC(=O)c23)Cc4ccc(F)c(c4)C(=O)N5CCN(CC5)C(=O)C6CC6)c(Br)c1 | Olaparib 1955 |
| OCc1ccc(Cl)cc1Oc2cccc3C(=NNC(=O)c23)Cc4ccc(F)c(c4)C(=O)N5CCN(CC5)C(=O)C6CC6 | Olaparib 2950 |
| CSCC(=O)C(=O)Nc1cccc2C(=NNC(=O)c12)Cc3ccc(F)c(c3)C(=O)N4CCN(CC4)C(=O)C5CC5 | Olaparib 332 |
| CO[C@@H]1OC[C@H](Oc2cccc3C(=NNC(=O)c23)Cc4ccc(F)c(c4)C(=O)N5CCN(CC5)C(=O)C6CC6)[C@@H](O)[C@H]1O | Olaparib 2942 |
| Fc1ccc(CC2=NNC(=O)c3c(Cc4ccc(Cl)cc4Cl)cccc23)cc1C(=O)N5CCN(CC5)C(=O)C6CC6 | Olaparib 1868 |
| Nc1ccc(Oc2cccc3C(=NNC(=O)c23)Cc4ccc(F)c(c4)C(=O)N5CCN(CC5)C(=O)C6CC6)cc1 | Olaparib 3049 |
| CSCC(=O)C(=O)Oc1cccc2C(=NNC(=O)c12)Cc3ccc(F)c(c3)C(=O)N4CCN(CC4)C(=O)C5CC5 | Olaparib 1257 |
| CO[C@H]1OC[C@@H](Oc2cccc3C(=NNC(=O)c23)Cc4ccc(F)c(c4)C(=O)N5CCN(CC5)C(=O)C6CC6)[C@H](O)[C@H]1O | Olaparib 2944 |
| CN(C)C(=O)[C@@H](O)[C@H](OC(=O)c1cccc2C(=NNC(=O)c12)Cc3ccc(F)c(c3)C(=O)N4CCN(CC4)C(=O)C5CC5)C(=O)N(C)C | Olaparib 561 |
| Oc1cc(Cl)ccc1Oc2cccc3C(=NNC(=O)c23)Cc4ccc(F)c(c4)C(=O)N5CCN(CC5)C(=O)C6CC6 | Olaparib 2987 |
| C[C@@H](SCC#N)C(=O)Nc1cccc2C(=NNC(=O)c12)Cc3ccc(F)c(c3)C(=O)N4CCN(CC4)C(=O)C5CC5 | Olaparib 307 |
| CC(C)(C)NC(=O)c1ccc(OC(=O)c2cccc3C(=NNC(=O)c23)Cc4ccc(F)c(c4)C(=O)N5CCN(CC5)C(=O)C6CC6)cc1 | Olaparib 734 |
| OC(=O)[C@](F)(Oc1cccc2C(=NNC(=O)c12)Cc3ccc(F)c(c3)C(=O)N4CCN(CC4)C(=O)C5CC5)S(=O)(=O)O | Olaparib 2127 |
| Fc1ccc(CC2=NNC(=O)c3c(NC(=O)C(=C)[C@H]4CCCC=C4)cccc23)cc1C(=O)N5CCN(CC5)C(=O)C6CC6 | Olaparib 511 |
| CNC(=S)NCOc1cccc2C(=NNC(=O)c12)Cc3ccc(F)c(c3)C(=O)N4CCN(CC4)C(=O)C5CC5 | Olaparib 2782 |
| CC(C)(COC(=O)c1cccc2C(=NNC(=O)c12)Cc3ccc(F)c(c3)C(=O)N4CCN(CC4)C(=O)C5CC5)NC(=O)c6ccccc6 | Olaparib 733 |
| NC(=S(=O)=O)NC(=O)c1cccc2C(=NNC(=O)c12)Cc3ccc(F)c(c3)C(=O)N4CCN(CC4)C(=O)C5CC5 | Olaparib 87 |
| Fc1ccc(CC2=NNC(=O)c3c(OC(=O)C(=C)[C@H]4CCCC=C4)cccc23)cc1C(=O)N5CCN(CC5)C(=O)C6CC6 | Olaparib 1506 |
| C[C@@H](SCC#N)C(=O)Oc1cccc2C(=NNC(=O)c12)Cc3ccc(F)c(c3)C(=O)N4CCN(CC4)C(=O)C5CC5 | Olaparib 1227 |
| CCCC(=O)Nc1ccc(OC(=O)c2cccc3C(=NNC(=O)c23)Cc4ccc(F)c(c4)C(=O)N5CCN(CC5)C(=O)C6CC6)cc1 | Olaparib 768 |
| CN(CC(=O)O)C(=N)NC(=O)c1cccc2C(=NNC(=O)c12)Cc3ccc(F)c(c3)C(=O)N4CCN(CC4)C(=O)C5CC5 | Olaparib 31 |
| Fc1ccc(CC2=NNC(=O)c3c(OC(=O)C4=CCCC4)cccc23)cc1C(=O)N5CCN(CC5)C(=O)C6CC6 | Olaparib 1521 |
| C\C=C\C(=O)C(=O)Nc1cccc2C(=NNC(=O)c12)Cc3ccc(F)c(c3)C(=O)N4CCN(CC4)C(=O)C5CC5 | Olaparib 421 |
| CC(=O)NCCc1ccc(OC(=O)c2cccc3C(=NNC(=O)c23)Cc4ccc(F)c(c4)C(=O)N5CCN(CC5)C(=O)C6CC6)cc1 | Olaparib 769 |
| NC(=O)NC(=O)CCC(=O)Nc1cccc2C(=NNC(=O)c12)Cc3ccc(F)c(c3)C(=O)N4CCN(CC4)C(=O)C5CC5 | Olaparib 287 |
| N[C@@H]1CCCC[C@H]1Oc2cccc3C(=NNC(=O)c23)Cc4ccc(F)c(c4)C(=O)N5CCN(CC5)C(=O)C6CC6 | Olaparib 3034 |
| C\C=C\C(=O)C(=O)Oc1cccc2C(=NNC(=O)c12)Cc3ccc(F)c(c3)C(=O)N4CCN(CC4)C(=O)C5CC5 | Olaparib 1367 |
| COc1cc(CC(=O)C)ccc1OC(=O)c2cccc3C(=NNC(=O)c23)Cc4ccc(F)c(c4)C(=O)N5CCN(CC5)C(=O)C6CC6 | Olaparib 766 |
| OC(=O)CNC(=N)NC(=O)c1cccc2C(=NNC(=O)c12)Cc3ccc(F)c(c3)C(=O)N4CCN(CC4)C(=O)C5CC5 | Olaparib 72 |
| CC(C)Oc1ccc(Cc2cccc3C(=NNC(=O)c23)Cc4ccc(F)c(c4)C(=O)N5CCN(CC5)C(=O)C6CC6)cc1 | Olaparib 1873 |
| OC(=O)\C(=C(\C=O)/c1cccc2C(=NNC(=O)c12)Cc3ccc(F)c(c3)C(=O)N4CCN(CC4)C(=O)C5CC5)\Cl | Olaparib 1612 |
| CC(=O)c1cc(OC(=O)c2cccc3C(=NNC(=O)c23)Cc4ccc(F)c(c4)C(=O)N5CCN(CC5)C(=O)C6CC6)c(C)cc1O | Olaparib 812 |
| OS(=O)(=O)CCC(=O)Oc1cccc2C(=NNC(=O)c12)Cc3ccc(F)c(c3)C(=O)N4CCN(CC4)C(=O)C5CC5 | Olaparib 1208 |
| COc1ccc(Cc2cccc3C(=NNC(=O)c23)Cc4ccc(F)c(c4)C(=O)N5CCN(CC5)C(=O)C6CC6)c(C)c1 | Olaparib 1875 |
| Fc1ccc(CC2=NNC(=O)c3c(NC(=O)\C(=C(/Cl)\C=O)\Cl)cccc23)cc1C(=O)N4CCN(CC4)C(=O)C5CC5 | Olaparib 281 |
| CC(=O)c1c(O)ccc(C)c1OC(=O)c2cccc3C(=NNC(=O)c23)Cc4ccc(F)c(c4)C(=O)N5CCN(CC5)C(=O)C6CC6 | Olaparib 814 |
| CC(=O)[C@H](O)[C@H](OC(=O)c1cccc2C(=NNC(=O)c12)Cc3ccc(F)c(c3)C(=O)N4CCN(CC4)C(=O)C5CC5)C(=O)C | Olaparib 569 |
| COc1ccc(Cc2cccc3C(=NNC(=O)c23)Cc4ccc(F)c(c4)C(=O)N5CCN(CC5)C(=O)C6CC6)cc1C | Olaparib 1876 |
| Fc1ccc(CC2=NNC(=O)c3c(OC(=O)\C(=C(/Cl)\C=O)\Cl)cccc23)cc1C(=O)N4CCN(CC4)C(=O)C5CC5 | Olaparib 1189 |
| COc1cc(\C=C\COC(=O)c2cccc3C(=NNC(=O)c23)Cc4ccc(F)c(c4)C(=O)N5CCN(CC5)C(=O)C6CC6)ccc1O | Olaparib 765 |
| CC\C(=N\NC(=S)NC(=O)c1cccc2C(=NNC(=O)c12)Cc3ccc(F)c(c3)C(=O)N4CCN(CC4)C(=O)C5CC5)\C | Olaparib 4 |
| CCSc1ccc(Cc2cccc3C(=NNC(=O)c23)Cc4ccc(F)c(c4)C(=O)N5CCN(CC5)C(=O)C6CC6)cc1 | Olaparib 1871 |
| CCCC(=O)[C@H](C)NC(=O)c1cccc2C(=NNC(=O)c12)Cc3ccc(F)c(c3)C(=O)N4CCN(CC4)C(=O)C5CC5 | Olaparib 77 |
| CCC(=O)Nc1ccc(OC(=O)c2cccc3C(=NNC(=O)c23)Cc4ccc(F)c(c4)C(=O)N5CCN(CC5)C(=O)C6CC6)c(F)c1 | Olaparib 757 |
| CC(=NNC(=S)NC(=O)c1cccc2C(=NNC(=O)c12)Cc3ccc(F)c(c3)C(=O)N4CCN(CC4)C(=O)C5CC5)C | Olaparib 25 |
| CCOc1ccc(Cc2cccc3C(=NNC(=O)c23)Cc4ccc(F)c(c4)C(=O)N5CCN(CC5)C(=O)C6CC6)cc1 | Olaparib 1877 |
| C[C@](N)(CC=C)C(=O)Nc1cccc2C(=NNC(=O)c12)Cc3ccc(F)c(c3)C(=O)N4CCN(CC4)C(=O)C5CC5 | Olaparib 366 |
| CCC(=O)c1cc(OC(=O)c2cccc3C(=NNC(=O)c23)Cc4ccc(F)c(c4)C(=O)N5CCN(CC5)C(=O)C6CC6)ccc1O | Olaparib 813 |
| COC(=O)CC(=S)NC(=O)c1cccc2C(=NNC(=O)c12)Cc3ccc(F)c(c3)C(=O)N4CCN(CC4)C(=O)C5CC5 | Olaparib 21 |
| [2H]C([2H])([2H])C([2H])(c1cc(ccn1)c2cccc3C(=NNC(=O)c23)Cc4ccc(F)c(c4)C(=O)N5CCN(CC5)C(=O)C6CC6)C([2H])([2H])[2H] | Olaparib 1977 |
| CN(C)C\C=C\C(=O)Nc1cccc2C(=NNC(=O)c12)Cc3ccc(F)c(c3)C(=O)N4CCN(CC4)C(=O)C5CC5 | Olaparib 365 |
| COC(=O)CC(=O)c1ccc(Oc2cccc3C(=NNC(=O)c23)Cc4ccc(F)c(c4)C(=O)N5CCN(CC5)C(=O)C6CC6)cc1 | Olaparib 2191 |
| CCOC(=O)C(=S)NC(=O)c1cccc2C(=NNC(=O)c12)Cc3ccc(F)c(c3)C(=O)N4CCN(CC4)C(=O)C5CC5 | Olaparib 20 |
| CSc1ccc(Cc2cccc3C(=NNC(=O)c23)Cc4ccc(F)c(c4)C(=O)N5CCN(CC5)C(=O)C6CC6)cc1 | Olaparib 1874 |
| CC(C)C(=O)CC(=O)Nc1cccc2C(=NNC(=O)c12)Cc3ccc(F)c(c3)C(=O)N4CCN(CC4)C(=O)C5CC5 | Olaparib 360 |
| COc1ccc(cc1)C(=N)NC(=O)c2cccc3C(=NNC(=O)c23)Cc4ccc(F)c(c4)C(=O)N5CCN(CC5)C(=O)C6CC6 | Olaparib 187 |
| CS(=O)(=O)CC(=O)Oc1cccc2C(=NNC(=O)c12)Cc3ccc(F)c(c3)C(=O)N4CCN(CC4)C(=O)C5CC5 | Olaparib 1252 |
| COc1ccc(Cc2cccc3C(=NNC(=O)c23)Cc4ccc(F)c(c4)C(=O)N5CCN(CC5)C(=O)C6CC6)cc1 | Olaparib 1880 |
| C[C@](O)(CC=C)C(=O)Nc1cccc2C(=NNC(=O)c12)Cc3ccc(F)c(c3)C(=O)N4CCN(CC4)C(=O)C5CC5 | Olaparib 359 |
| Fc1ccc(CC2=NNC(=O)c3c(cccc23)C(=O)OCCC(=O)Nc4ccccc4)cc1C(=O)N5CCN(CC5)C(=O)C6CC6 | Olaparib 818 |
| OC(=O)\C(=C(/Oc1cccc2C(=NNC(=O)c12)Cc3ccc(F)c(c3)C(=O)N4CCN(CC4)C(=O)C5CC5)\C(=O)O)\Br | Olaparib 2093 |
| COc1cccc(Cc2cccc3C(=NNC(=O)c23)Cc4ccc(F)c(c4)C(=O)N5CCN(CC5)C(=O)C6CC6)c1 | Olaparib 1881 |
| C[C@](N)(C=C)C(=O)Nc1cccc2C(=NNC(=O)c12)Cc3ccc(F)c(c3)C(=O)N4CCN(CC4)C(=O)C5CC5 | Olaparib 411 |
| Fc1ccc(CC2=NNC(=O)c3c(cccc23)C(=O)OCNC(=O)Cc4ccccc4)cc1C(=O)N5CCN(CC5)C(=O)C6CC6 | Olaparib 819 |
| C[C@@H](N(C)C(=O)NC(C)(C)C#C)C(=O)Oc1cccc2C(=NNC(=O)c12)Cc3ccc(F)c(c3)C(=O)N4CCN(CC4)C(=O)C5CC5 | Olaparib 1174 |
| COc1ccc(Cc2cccc3C(=NNC(=O)c23)Cc4ccc(F)c(c4)C(=O)N5CCN(CC5)C(=O)C6CC6)c(Cl)c1 | Olaparib 1859 |
| CC(C)CC(=O)C(=O)Oc1cccc2C(=NNC(=O)c12)Cc3ccc(F)c(c3)C(=O)N4CCN(CC4)C(=O)C5CC5 | Olaparib 1295 |
| Fc1ccc(CC2=NNC(=O)c3c(cccc23)C(=O)OCCNC(=O)c4ccc(Cl)cc4)cc1C(=O)N5CCN(CC5)C(=O)C6CC6 | Olaparib 722 |
| CP(=O)(O)C[C@@H](CN)OC(=O)c1cccc2C(=NNC(=O)c12)Cc3ccc(F)c(c3)C(=O)N4CCN(CC4)C(=O)C5CC5 | Olaparib 568 |
| FC(F)Oc1ccc(Cc2cccc3C(=NNC(=O)c23)Cc4ccc(F)c(c4)C(=O)N5CCN(CC5)C(=O)C6CC6)cc1 | Olaparib 1858 |
| CC(C)C(=O)CC(=O)Oc1cccc2C(=NNC(=O)c12)Cc3ccc(F)c(c3)C(=O)N4CCN(CC4)C(=O)C5CC5 | Olaparib 1293 |
| CC(=O)c1cccc(COC(=O)c2cccc3C(=NNC(=O)c23)Cc4ccc(F)c(c4)C(=O)N5CCN(CC5)C(=O)C6CC6)n1 | Olaparib 877 |
| CNC(=O)N(C)CCC(=O)Oc1cccc2C(=NNC(=O)c12)Cc3ccc(F)c(c3)C(=O)N4CCN(CC4)C(=O)C5CC5 | Olaparib 1197 |
| FC(F)Oc1cccc(Cc2cccc3C(=NNC(=O)c23)Cc4ccc(F)c(c4)C(=O)N5CCN(CC5)C(=O)C6CC6)c1 | Olaparib 1857 |
| C[C@](O)(CC=C)C(=O)Oc1cccc2C(=NNC(=O)c12)Cc3ccc(F)c(c3)C(=O)N4CCN(CC4)C(=O)C5CC5 | Olaparib 1292 |
| NC(=O)\C(=C\c1ccc(Oc2cccc3C(=NNC(=O)c23)Cc4ccc(F)c(c4)C(=O)N5CCN(CC5)C(=O)C6CC6)cc1)\C#N | Olaparib 2290 |
| C[C@H]([C@H](N)C(=O)O)C(=O)Nc1cccc2C(=NNC(=O)c12)Cc3ccc(F)c(c3)C(=O)N4CCN(CC4)C(=O)C5CC5 | Olaparib 296 |
| Nc1ccc(Cc2cccc3C(=NNC(=O)c23)Cc4ccc(F)c(c4)C(=O)N5CCN(CC5)C(=O)C6CC6)cc1 | Olaparib 2008 |
| C[C@H](C\C=C\C(=O)Nc1cccc2C(=NNC(=O)c12)Cc3ccc(F)c(c3)C(=O)N4CCN(CC4)C(=O)C5CC5)C#N | Olaparib 326 |
| Fc1ccc(CC2=NNC(=O)c3c(cccc23)C(=O)OCNC(=O)c4ccccc4)cc1C(=O)N5CCN(CC5)C(=O)C6CC6 | Olaparib 878 |
| CC(=O)[C@@H](O)[C@@H](CO)OC(=O)c1cccc2C(=NNC(=O)c12)Cc3ccc(F)c(c3)C(=O)N4CCN(CC4)C(=O)C5CC5 | Olaparib 581 |
| Fc1ccc(CC2=NNC(=O)c3c(Cc4ccc(cc4)C#N)cccc23)cc1C(=O)N5CCN(CC5)C(=O)C6CC6 | Olaparib 1898 |
| CCO\C(=C\C(=O)Oc1cccc2C(=NNC(=O)c12)Cc3ccc(F)c(c3)C(=O)N4CCN(CC4)C(=O)C5CC5)\C | Olaparib 1297 |
| Fc1ccc(CC2=NNC(=O)c3c(cccc23)C(=O)Oc4ccc(NC(=O)C(F)(F)F)cc4F)cc1C(=O)N5CCN(CC5)C(=O)C6CC6 | Olaparib 696 |
| C[C@@H]([C@H](N)C(=O)Oc1cccc2C(=NNC(=O)c12)Cc3ccc(F)c(c3)C(=O)N4CCN(CC4)C(=O)C5CC5)C(=O)O | Olaparib 1216 |
| Fc1ccc(CC2=NNC(=O)c3c(Cc4cccc(c4)C#N)cccc23)cc1C(=O)N5CCN(CC5)C(=O)C6CC6 | Olaparib 1894 |
| CO\C=C(/C)\C(=O)Nc1cccc2C(=NNC(=O)c12)Cc3ccc(F)c(c3)C(=O)N4CCN(CC4)C(=O)C5CC5 | Olaparib 409 |
| O\N=C\c1ccc(OC(=O)c2cccc3C(=NNC(=O)c23)Cc4ccc(F)c(c4)C(=O)N5CCN(CC5)C(=O)C6CC6)cc1 | Olaparib 931 |
| C[C@](O)(CC(=O)O)C(=O)Nc1cccc2C(=NNC(=O)c12)Cc3ccc(F)c(c3)C(=O)N4CCN(CC4)C(=O)C5CC5 | Olaparib 293 |
| Fc1ccc(CC2=NNC(=O)c3c2cccc3c4ccccn4)cc1C(=O)N5CCN(CC5)C(=O)C6CC6 | Olaparib 2041 |
| CCOC(=O)\C=C\C(F)(F)Oc1cccc2C(=NNC(=O)c12)Cc3ccc(F)c(c3)C(=O)N4CCN(CC4)C(=O)C5CC5 | Olaparib 2134 |
| Fc1ccc(CC2=NNC(=O)c3c(cccc23)C(=O)OCc4ncc(C=O)cc4Cl)cc1C(=O)N5CCN(CC5)C(=O)C6CC6 | Olaparib 790 |
| C[C@H]([C@H](N)C(=O)O)C(=O)Oc1cccc2C(=NNC(=O)c12)Cc3ccc(F)c(c3)C(=O)N4CCN(CC4)C(=O)C5CC5 | Olaparib 1215 |
| CCc1ccc(Cc2cccc3C(=NNC(=O)c23)Cc4ccc(F)c(c4)C(=O)N5CCN(CC5)C(=O)C6CC6)s1 | Olaparib 1980 |
| OC(=O)[C@@](F)(CC=C)Oc1cccc2C(=NNC(=O)c12)Cc3ccc(F)c(c3)C(=O)N4CCN(CC4)C(=O)C5CC5 | Olaparib 2159 |
| Fc1ccc(CC2=NNC(=O)c3c(OC(=O)c4cnccc4C=O)cccc23)cc1C(=O)N5CCN(CC5)C(=O)C6CC6 | Olaparib 1508 |
| OP(=O)(O)CCCNC(=O)c1cccc2C(=NNC(=O)c12)Cc3ccc(F)c(c3)C(=O)N4CCN(CC4)C(=O)C5CC5 | Olaparib 12 |
| Fc1ccc(CC2=NNC(=O)c3c(Cc4occc4)cccc23)cc1C(=O)N5CCN(CC5)C(=O)C6CC6 | Olaparib 2039 |
| COC\C=C\C(=O)Nc1cccc2C(=NNC(=O)c12)Cc3ccc(F)c(c3)C(=O)N4CCN(CC4)C(=O)C5CC5 | Olaparib 408 |
| CC(C)[C@@](C)(OC(=O)c1cccc2C(=NNC(=O)c12)Cc3ccc(F)c(c3)C(=O)N4CCN(CC4)C(=O)C5CC5)c6cnccc6N | Olaparib 762 |
| C[C@](O)(CC(=O)Oc1cccc2C(=NNC(=O)c12)Cc3ccc(F)c(c3)C(=O)N4CCN(CC4)C(=O)C5CC5)C(=O)O | Olaparib 1213 |
| CC1=C[C@H](CCC1)Oc2cccc3C(=NNC(=O)c23)Cc4ccc(F)c(c4)C(=O)N5CCN(CC5)C(=O)C6CC6 | Olaparib 3044 |
| Fc1ccc(CC2=NNC(=O)c3c(OCCNC(=O)C=C)cccc23)cc1C(=O)N4CCN(CC4)C(=O)C5CC5 | Olaparib 2815 |
| CC(C)(C)OC(=O)Nc1ccc(Oc2cccc3C(=NNC(=O)c23)Cc4ccc(F)c(c4)C(=O)N5CCN(CC5)C(=O)C6CC6)cc1 | Olaparib 2239 |
| C[C@](O)(CC(=O)Oc1cccc2C(=NNC(=O)c12)Cc3ccc(F)c(c3)C(=O)N4CCN(CC4)C(=O)C5CC5)C(=O)O | Olaparib 1212 |
| Fc1ccc(CC2=NNC(=O)c3c2cccc3C4=CCCO4)cc1C(=O)N5CCN(CC5)C(=O)C6CC6 | Olaparib 2045 |
| CSC\C=C\C(=O)Nc1cccc2C(=NNC(=O)c12)Cc3ccc(F)c(c3)C(=O)N4CCN(CC4)C(=O)C5CC5 | Olaparib 340 |
| COc1ccc(OC)c(c1)[C@H](C)OC(=O)c2cccc3C(=NNC(=O)c23)Cc4ccc(F)c(c4)C(=O)N5CCN(CC5)C(=O)C6CC6 | Olaparib 761 |
| C[C@](O)(CC(=O)O)C(=O)Oc1cccc2C(=NNC(=O)c12)Cc3ccc(F)c(c3)C(=O)N4CCN(CC4)C(=O)C5CC5 | Olaparib 1211 |
| Fc1ccc(CC2=NNC(=O)c3c(cccc23)C4CCNCC4)cc1C(=O)N5CCN(CC5)C(=O)C6CC6 | Olaparib 2030 |
| CO\C=C(/C)\C(=O)Oc1cccc2C(=NNC(=O)c12)Cc3ccc(F)c(c3)C(=O)N4CCN(CC4)C(=O)C5CC5 | Olaparib 1348 |
| COc1c(CO)cc(C)cc1COC(=O)c2cccc3C(=NNC(=O)c23)Cc4ccc(F)c(c4)C(=O)N5CCN(CC5)C(=O)C6CC6 | Olaparib 760 |
| CS(=O)(=O)NCCOc1cccc2C(=NNC(=O)c12)Cc3ccc(F)c(c3)C(=O)N4CCN(CC4)C(=O)C5CC5 | Olaparib 2756 |
| Fc1ccc(CC2=NNC(=O)c3c(cccc23)[C@@H]4CCCNC4)cc1C(=O)N5CCN(CC5)C(=O)C6CC6 | Olaparib 2031 |
| CC(=O)CC(=O)Nc1cccc2C(=NNC(=O)c12)Cc3ccc(F)c(c3)C(=O)N4CCN(CC4)C(=O)C5CC5 | Olaparib 446 |
| C[C@H](CN(C)c1cccc(Br)n1)OC(=O)c2cccc3C(=NNC(=O)c23)Cc4ccc(F)c(c4)C(=O)N5CCN(CC5)C(=O)C6CC6 | Olaparib 686 |
| OCCNC(=N)NC(=O)c1cccc2C(=NNC(=O)c12)Cc3ccc(F)c(c3)C(=O)N4CCN(CC4)C(=O)C5CC5 | Olaparib 102 |
| Fc1ccc(CC2=NNC(=O)c3c(C[C@@H]4CCCO4)cccc23)cc1C(=O)N5CCN(CC5)C(=O)C6CC6 | Olaparib 2029 |
| CSC\C=C\C(=O)Oc1cccc2C(=NNC(=O)c12)Cc3ccc(F)c(c3)C(=O)N4CCN(CC4)C(=O)C5CC5 | Olaparib 1267 |
| C[C@@H](N)[C@@H](OC(=O)c1cccc2C(=NNC(=O)c12)Cc3ccc(F)c(c3)C(=O)N4CCN(CC4)C(=O)C5CC5)c6ccc(O)cc6 | Olaparib 804 |
| N[C@H](CC(=O)N)C(=O)Oc1cccc2C(=NNC(=O)c12)Cc3ccc(F)c(c3)C(=O)N4CCN(CC4)C(=O)C5CC5 | Olaparib 1272 |
| Fc1ccc(CC2=NNC(=O)c3c(\C=C/c4ccccc4)cccc23)cc1C(=O)N5CCN(CC5)C(=O)C6CC6 | Olaparib 2011 |
| Fc1ccc(CC2=NNC(=O)c3c(OCNC(=O)C=C)cccc23)cc1C(=O)N4CCN(CC4)C(=O)C5CC5 | Olaparib 2852 |
| C[C@H](N)[C@H](O)c1ccc(OC(=O)c2cccc3C(=NNC(=O)c23)Cc4ccc(F)c(c4)C(=O)N5CCN(CC5)C(=O)C6CC6)cc1 | Olaparib 805 |
| OC[C@@H](OC(=O)c1cccc2C(=NNC(=O)c12)Cc3ccc(F)c(c3)C(=O)N4CCN(CC4)C(=O)C5CC5)[C@@H](O)C=O | Olaparib 615 |
| Fc1ccc(CC2=NNC(=O)c3c(Cc4ccc(Cl)cc4)cccc23)cc1C(=O)N5CCN(CC5)C(=O)C6CC6 | Olaparib 1979 |
| CO\C=C\C(=O)Oc1cccc2C(=NNC(=O)c12)Cc3ccc(F)c(c3)C(=O)N4CCN(CC4)C(=O)C5CC5 | Olaparib 1395 |
| CNC[C@H](OC(=O)c1cccc2C(=NNC(=O)c12)Cc3ccc(F)c(c3)C(=O)N4CCN(CC4)C(=O)C5CC5)c6cccc(O)c6 | Olaparib 806 |
| N[C@H](CC(=O)O)C(=O)Nc1cccc2C(=NNC(=O)c12)Cc3ccc(F)c(c3)C(=O)N4CCN(CC4)C(=O)C5CC5 | Olaparib 337 |
| Fc1ccc(CC2=NNC(=O)c3c(Cc4ccccc4)cccc23)cc1C(=O)N5CCN(CC5)C(=O)C6CC6 | Olaparib 1897 |
| CC(=O)\C(=N\O)\c1cccc2C(=NNC(=O)c12)Cc3ccc(F)c(c3)C(=O)N4CCN(CC4)C(=O)C5CC5 | Olaparib 1640 |
| CC(C)(Nc1ccc(cc1F)C#N)C(=O)Oc2cccc3C(=NNC(=O)c23)Cc4ccc(F)c(c4)C(=O)N5CCN(CC5)C(=O)C6CC6 | Olaparib 1427 |
| N[C@H](CC(=O)Nc1cccc2C(=NNC(=O)c12)Cc3ccc(F)c(c3)C(=O)N4CCN(CC4)C(=O)C5CC5)C(=O)O | Olaparib 338 |
| Fc1ccc(CC2=NNC(=O)c3c(Cc4ccc(I)cc4)cccc23)cc1C(=O)N5CCN(CC5)C(=O)C6CC6 | Olaparib 1896 |
| COC(=O)C(=C(F)F)Oc1cccc2C(=NNC(=O)c12)Cc3ccc(F)c(c3)C(=O)N4CCN(CC4)C(=O)C5CC5 | Olaparib 2155 |
| COc1ccc(CCN)cc1OC(=O)c2cccc3C(=NNC(=O)c23)Cc4ccc(F)c(c4)C(=O)N5CCN(CC5)C(=O)C6CC6 | Olaparib 803 |
| O[C@H](CC(=O)Nc1cccc2C(=NNC(=O)c12)Cc3ccc(F)c(c3)C(=O)N4CCN(CC4)C(=O)C5CC5)C(=O)O | Olaparib 335 |
| Fc1ccc(Cc2cccc3C(=NNC(=O)c23)Cc4ccc(F)c(c4)C(=O)N5CCN(CC5)C(=O)C6CC6)cc1 | Olaparib 2000 |
| OC(=O)C(=O)Cc1cccc2C(=NNC(=O)c12)Cc3ccc(F)c(c3)C(=O)N4CCN(CC4)C(=O)C5CC5 | Olaparib 1645 |
| CCNC(=O)COc1ccc(Oc2cccc3C(=NNC(=O)c23)Cc4ccc(F)c(c4)C(=O)N5CCN(CC5)C(=O)C6CC6)cc1Cl | Olaparib 2207 |
| N[C@H](CC(=O)O)C(=O)Oc1cccc2C(=NNC(=O)c12)Cc3ccc(F)c(c3)C(=O)N4CCN(CC4)C(=O)C5CC5 | Olaparib 1264 |
| Fc1ccc(Cc2cccc3C(=NNC(=O)c23)Cc4ccc(F)c(c4)C(=O)N5CCN(CC5)C(=O)C6CC6)cc1Cl | Olaparib 1895 |
| CN(C)CC(C)(C)COC(=O)c1cccc2C(=NNC(=O)c12)Cc3ccc(F)c(c3)C(=O)N4CCN(CC4)C(=O)C5CC5 | Olaparib 591 |
| CN(C)C(=O)Nc1ccc(Oc2cccc3C(=NNC(=O)c23)Cc4ccc(F)c(c4)C(=O)N5CCN(CC5)C(=O)C6CC6)cc1 | Olaparib 2318 |
| N[C@H](CC(=O)Oc1cccc2C(=NNC(=O)c12)Cc3ccc(F)c(c3)C(=O)N4CCN(CC4)C(=O)C5CC5)C(=O)O | Olaparib 1266 |
| Fc1ccc(CC2=NNC(=O)c3c(CCCC4C=CC=C4)cccc23)cc1C(=O)N5CCN(CC5)C(=O)C6CC6 | Olaparib 1833 |
| CC(C)N(C(C)C)C(=O)COc1cccc2C(=NNC(=O)c12)Cc3ccc(F)c(c3)C(=O)N4CCN(CC4)C(=O)C5CC5 | Olaparib 2752 |
| COc1cc(Br)c(COC(=O)c2cccc3C(=NNC(=O)c23)Cc4ccc(F)c(c4)C(=O)N5CCN(CC5)C(=O)C6CC6)cc1OC | Olaparib 685 |
| OP(=O)(O)CCC(=O)Oc1cccc2C(=NNC(=O)c12)Cc3ccc(F)c(c3)C(=O)N4CCN(CC4)C(=O)C5CC5 | Olaparib 1209 |
| Fc1ccc(CC2=NNC(=O)c3c(cccc23)C4=CCCCCC4)cc1C(=O)N5CCN(CC5)C(=O)C6CC6 | Olaparib 2018 |
| CC(C)(O)C(C)(C)C(=O)Nc1cccc2C(=NNC(=O)c12)Cc3ccc(F)c(c3)C(=O)N4CCN(CC4)C(=O)C5CC5 | Olaparib 298 |
| OCCCc1ccc(O)c(OC(=O)c2cccc3C(=NNC(=O)c23)Cc4ccc(F)c(c4)C(=O)N5CCN(CC5)C(=O)C6CC6)c1 | Olaparib 798 |
| NC(=O)NCCOC(=O)c1cccc2C(=NNC(=O)c12)Cc3ccc(F)c(c3)C(=O)N4CCN(CC4)C(=O)C5CC5 | Olaparib 670 |
| Fc1ccc(CC2=NNC(=O)c3c(CCC4CC4)cccc23)cc1C(=O)N5CCN(CC5)C(=O)C6CC6 | Olaparib 2044 |
| CC(C)C[C@](C)(N)C(=O)Nc1cccc2C(=NNC(=O)c12)Cc3ccc(F)c(c3)C(=O)N4CCN(CC4)C(=O)C5CC5 | Olaparib 306 |
| OCCCc1ccc(OC(=O)c2cccc3C(=NNC(=O)c23)Cc4ccc(F)c(c4)C(=O)N5CCN(CC5)C(=O)C6CC6)c(O)c1 | Olaparib 799 |
| NC(=O)N[C@H](OC(=O)c1cccc2C(=NNC(=O)c12)Cc3ccc(F)c(c3)C(=O)N4CCN(CC4)C(=O)C5CC5)C(Cl)(Cl)Cl | Olaparib 559 |
| CCS(=O)(=O)CCOc1cccc2C(=NNC(=O)c12)Cc3ccc(F)c(c3)C(=O)N4CCN(CC4)C(=O)C5CC5 | Olaparib 2758 |
| C[C@@H](N)CC(C)(C)NC(=O)c1cccc2C(=NNC(=O)c12)Cc3ccc(F)c(c3)C(=O)N4CCN(CC4)C(=O)C5CC5 | Olaparib 74 |
| Oc1ccc(CCCOC(=O)c2cccc3C(=NNC(=O)c23)Cc4ccc(F)c(c4)C(=O)N5CCN(CC5)C(=O)C6CC6)cc1O | Olaparib 800 |
| CCOC(=O)\C(=N\O)\Oc1cccc2C(=NNC(=O)c12)Cc3ccc(F)c(c3)C(=O)N4CCN(CC4)C(=O)C5CC5 | Olaparib 2148 |
| CCNC(=S)NC(=O)c1cccc2C(=NNC(=O)c12)Cc3ccc(F)c(c3)C(=O)N4CCN(CC4)C(=O)C5CC5 | Olaparib 96 |
| CC(C)C[C@](C)(N)C(=O)Oc1cccc2C(=NNC(=O)c12)Cc3ccc(F)c(c3)C(=O)N4CCN(CC4)C(=O)C5CC5 | Olaparib 1226 |
| CCOc1cc(CO)ccc1OC(=O)c2cccc3C(=NNC(=O)c23)Cc4ccc(F)c(c4)C(=O)N5CCN(CC5)C(=O)C6CC6 | Olaparib 801 |
| NC(=O)C(Br)(Oc1cccc2C(=NNC(=O)c12)Cc3ccc(F)c(c3)C(=O)N4CCN(CC4)C(=O)C5CC5)C(=O)N | Olaparib 2097 |
| CC(=O)SCC(=O)Oc1cccc2C(=NNC(=O)c12)Cc3ccc(F)c(c3)C(=O)N4CCN(CC4)C(=O)C5CC5 | Olaparib 1258 |
| CC(C)(O)C(C)(C)C(=O)Oc1cccc2C(=NNC(=O)c12)Cc3ccc(F)c(c3)C(=O)N4CCN(CC4)C(=O)C5CC5 | Olaparib 1218 |
| CN(C)C(=O)Nc1ccc(Cl)c(Oc2cccc3C(=NNC(=O)c23)Cc4ccc(F)c(c4)C(=O)N5CCN(CC5)C(=O)C6CC6)c1 | Olaparib 2234 |
| N[C@@H](CC(=O)Oc1cccc2C(=NNC(=O)c12)Cc3ccc(F)c(c3)C(=O)N4CCN(CC4)C(=O)C5CC5)C(=O)[O-] | Olaparib 1275 |
| CS[C@@H](CO)[C@H](C)NC(=O)c1cccc2C(=NNC(=O)c12)Cc3ccc(F)c(c3)C(=O)N4CCN(CC4)C(=O)C5CC5 | Olaparib 16 |
| CCC[C@H](O)[C@H](C)NC(=O)c1cccc2C(=NNC(=O)c12)Cc3ccc(F)c(c3)C(=O)N4CCN(CC4)C(=O)C5CC5 | Olaparib 58 |
| Cc1cnccc1NCCOC(=O)c2cccc3C(=NNC(=O)c23)Cc4ccc(F)c(c4)C(=O)N5CCN(CC5)C(=O)C6CC6 | Olaparib 856 |
| O[C@H](CC(=O)O)C(=O)Oc1cccc2C(=NNC(=O)c12)Cc3ccc(F)c(c3)C(=O)N4CCN(CC4)C(=O)C5CC5 | Olaparib 1261 |
| CS[C@@H](COC(=O)c1cccc2C(=NNC(=O)c12)Cc3ccc(F)c(c3)C(=O)N4CCN(CC4)C(=O)C5CC5)[C@@H](C)N | Olaparib 573 |
| C[C@@H](O)C(C)(C)COC(=O)c1cccc2C(=NNC(=O)c12)Cc3ccc(F)c(c3)C(=O)N4CCN(CC4)C(=O)C5CC5 | Olaparib 625 |
| OC[C@H](COC(=O)c1cccc2C(=NNC(=O)c12)Cc3ccc(F)c(c3)C(=O)N4CCN(CC4)C(=O)C5CC5)c6ccccn6 | Olaparib 855 |
| Fc1ccc(CC2=NNC(=O)c3c(NC(=O)CONC(=O)C#C)cccc23)cc1C(=O)N4CCN(CC4)C(=O)C5CC5 | Olaparib 316 |
| Fc1ccc(CC2=NNC(=O)c3c(NC(=O)\C=C\C=C\C=O)cccc23)cc1C(=O)N4CCN(CC4)C(=O)C5CC5 | Olaparib 378 |
| CC(C)(C)C[C@H](O)C(=O)Oc1cccc2C(=NNC(=O)c12)Cc3ccc(F)c(c3)C(=O)N4CCN(CC4)C(=O)C5CC5 | Olaparib 1217 |
| COc1ccc(COC(=O)c2cccc3C(=NNC(=O)c23)Cc4ccc(F)c(c4)C(=O)N5CCN(CC5)C(=O)C6CC6)cc1O | Olaparib 853 |
| NC(=O)NCC(=O)Oc1cccc2C(=NNC(=O)c12)Cc3ccc(F)c(c3)C(=O)N4CCN(CC4)C(=O)C5CC5 | Olaparib 1331 |
| Fc1ccc(CC2=NNC(=O)c3c(OC(=O)\C=C\C=C\C=O)cccc23)cc1C(=O)N4CCN(CC4)C(=O)C5CC5 | Olaparib 1313 |
| C[C@H](OC(=O)c1cccc2C(=NNC(=O)c12)Cc3ccc(F)c(c3)C(=O)N4CCN(CC4)C(=O)C5CC5)C(C)(C)CO | Olaparib 624 |
| NCc1ccc(OC(=O)c2cccc3C(=NNC(=O)c23)Cc4ccc(F)c(c4)C(=O)N5CCN(CC5)C(=O)C6CC6)c(O)c1 | Olaparib 919 |
| NC(=S)NCC(=O)Oc1cccc2C(=NNC(=O)c12)Cc3ccc(F)c(c3)C(=O)N4CCN(CC4)C(=O)C5CC5 | Olaparib 1256 |
| CC(=O)\C=C\C(=O)Oc1cccc2C(=NNC(=O)c12)Cc3ccc(F)c(c3)C(=O)N4CCN(CC4)C(=O)C5CC5 | Olaparib 1366 |
| C[C@@H](CN(C)C)NC(=O)c1cccc2C(=NNC(=O)c12)Cc3ccc(F)c(c3)C(=O)N4CCN(CC4)C(=O)C5CC5 | Olaparib 111 |
| CC(=O)N(O)c1ccc(Oc2cccc3C(=NNC(=O)c23)Cc4ccc(F)c(c4)C(=O)N5CCN(CC5)C(=O)C6CC6)cc1 | Olaparib 2345 |
| COC(=O)\C(=N\O)\Oc1cccc2C(=NNC(=O)c12)Cc3ccc(F)c(c3)C(=O)N4CCN(CC4)C(=O)C5CC5 | Olaparib 2158 |
| C[C@H](\N=C/C(C)(C)C)C(=O)Oc1cccc2C(=NNC(=O)c12)Cc3ccc(F)c(c3)C(=O)N4CCN(CC4)C(=O)C5CC5 | Olaparib 1203 |
| CCC[C@](C)(N)C(=O)Nc1cccc2C(=NNC(=O)c12)Cc3ccc(F)c(c3)C(=O)N4CCN(CC4)C(=O)C5CC5 | Olaparib 349 |
| N[C@@H](COC(=O)c1cccc2C(=NNC(=O)c12)Cc3ccc(F)c(c3)C(=O)N4CCN(CC4)C(=O)C5CC5)c6ccccn6 | Olaparib 921 |
| Fc1ccc(CC2=NNC(=O)c3c(OC(=O)CONC(=O)C#C)cccc23)cc1C(=O)N4CCN(CC4)C(=O)C5CC5 | Olaparib 1239 |
| CCCCC(=N)NC(=O)c1cccc2C(=NNC(=O)c12)Cc3ccc(F)c(c3)C(=O)N4CCN(CC4)C(=O)C5CC5 | Olaparib 123 |
| CC[C@@](C)(CO)COC(=O)c1cccc2C(=NNC(=O)c12)Cc3ccc(F)c(c3)C(=O)N4CCN(CC4)C(=O)C5CC5 | Olaparib 623 |
| Oc1ccc(CC(=O)Nc2cccc3C(=NNC(=O)c23)Cc4ccc(F)c(c4)C(=O)N5CCN(CC5)C(=O)C6CC6)cc1O | Olaparib 501 |
| O\N=C(/Cl)\C(=N\O)\Oc1cccc2C(=NNC(=O)c12)Cc3ccc(F)c(c3)C(=O)N4CCN(CC4)C(=O)C5CC5 | Olaparib 2147 |
| CCOC(=O)\C=C(/C)\C(F)(F)Oc1cccc2C(=NNC(=O)c12)Cc3ccc(F)c(c3)C(=O)N4CCN(CC4)C(=O)C5CC5 | Olaparib 2121 |
| CCC[C@@](C)(O)C(=O)Nc1cccc2C(=NNC(=O)c12)Cc3ccc(F)c(c3)C(=O)N4CCN(CC4)C(=O)C5CC5 | Olaparib 343 |
| N[C@H](C(=O)O)c1ccc(Oc2cccc3C(=NNC(=O)c23)Cc4ccc(F)c(c4)C(=O)N5CCN(CC5)C(=O)C6CC6)cc1 | Olaparib 2386 |
| N[C@H](C[C@H](N)C(F)(F)Oc1cccc2C(=NNC(=O)c12)Cc3ccc(F)c(c3)C(=O)N4CCN(CC4)C(=O)C5CC5)C(=O)O | Olaparib 2115 |
| NC(=O)N[C@@H](O)C(Cl)(Cl)Oc1cccc2C(=NNC(=O)c12)Cc3ccc(F)c(c3)C(=O)N4CCN(CC4)C(=O)C5CC5 | Olaparib 2105 |
| CCOC(=O)\C=C(\C)/C(F)(F)Oc1cccc2C(=NNC(=O)c12)Cc3ccc(F)c(c3)C(=O)N4CCN(CC4)C(=O)C5CC5 | Olaparib 2120 |
| CC[C@H](CCN)NC(=O)c1cccc2C(=NNC(=O)c12)Cc3ccc(F)c(c3)C(=O)N4CCN(CC4)C(=O)C5CC5 | Olaparib 112 |
| Cc1nc(OC(=O)c2cccc3C(=NNC(=O)c23)Cc4ccc(F)c(c4)C(=O)N5CCN(CC5)C(=O)C6CC6)c(cc1C(F)(F)F)C#N | Olaparib 718 |
| OCNC(=O)NCOc1cccc2C(=NNC(=O)c12)Cc3ccc(F)c(c3)C(=O)N4CCN(CC4)C(=O)C5CC5 | Olaparib 2785 |
| CN(C)C\C=C\C(=O)Oc1cccc2C(=NNC(=O)c12)Cc3ccc(F)c(c3)C(=O)N4CCN(CC4)C(=O)C5CC5 | Olaparib 1299 |
| CC(C)[C@H](N)CC(=O)Oc1cccc2C(=NNC(=O)c12)Cc3ccc(F)c(c3)C(=O)N4CCN(CC4)C(=O)C5CC5 | Olaparib 1278 |
| Fc1ccc(CC2=NNC(=O)c3c(cccc23)C(=O)OCc4cccc(OCC#N)c4Br)cc1C(=O)N5CCN(CC5)C(=O)C6CC6 | Olaparib 688 |
| CC(C)OC(=O)\C=C(/C)\NC(=O)c1cccc2C(=NNC(=O)c12)Cc3ccc(F)c(c3)C(=O)N4CCN(CC4)C(=O)C5CC5 | Olaparib 7 |
| C[C@H](CC(=O)C)C(=O)Oc1cccc2C(=NNC(=O)c12)Cc3ccc(F)c(c3)C(=O)N4CCN(CC4)C(=O)C5CC5 | Olaparib 1296 |
| CC(C)(C)NCC(=O)Oc1cccc2C(=NNC(=O)c12)Cc3ccc(F)c(c3)C(=O)N4CCN(CC4)C(=O)C5CC5 | Olaparib 1277 |
| COc1ncccc1COC(=O)c2cccc3C(=NNC(=O)c23)Cc4ccc(F)c(c4)C(=O)N5CCN(CC5)C(=O)C6CC6 | Olaparib 920 |
| CC(C)(C)NC(=O)\C=C\C(=O)Oc1cccc2C(=NNC(=O)c12)Cc3ccc(F)c(c3)C(=O)N4CCN(CC4)C(=O)C5CC5 | Olaparib 1185 |
| CC(C)C(=S)NC(=O)c1cccc2C(=NNC(=O)c12)Cc3ccc(F)c(c3)C(=O)N4CCN(CC4)C(=O)C5CC5 | Olaparib 98 |
| C[C@H](N)[C@H](C)C(=O)Nc1cccc2C(=NNC(=O)c12)Cc3ccc(F)c(c3)C(=O)N4CCN(CC4)C(=O)C5CC5 | Olaparib 397 |
| Nc1cccc(Oc2cccc3C(=NNC(=O)c23)Cc4ccc(F)c(c4)C(=O)N5CCN(CC5)C(=O)C6CC6)c1C(=O)O | Olaparib 2407 |
| C\C=C\[C@H](CC(=O)N)OC(=O)c1cccc2C(=NNC(=O)c12)Cc3ccc(F)c(c3)C(=O)N4CCN(CC4)C(=O)C5CC5 | Olaparib 599 |
| C[C@H](C\C=C\C(=O)Oc1cccc2C(=NNC(=O)c12)Cc3ccc(F)c(c3)C(=O)N4CCN(CC4)C(=O)C5CC5)C#N | Olaparib 1250 |
| CC[C@H](CCO)OC(=O)c1cccc2C(=NNC(=O)c12)Cc3ccc(F)c(c3)C(=O)N4CCN(CC4)C(=O)C5CC5 | Olaparib 667 |
| OC(=O)c1ccc(Oc2cccc3C(=NNC(=O)c23)Cc4ccc(F)c(c4)C(=O)N5CCN(CC5)C(=O)C6CC6)cc1OC(F)(F)F | Olaparib 2249 |
| CCOC(=O)\C=C(/C)\NC(=O)c1cccc2C(=NNC(=O)c12)Cc3ccc(F)c(c3)C(=O)N4CCN(CC4)C(=O)C5CC5 | Olaparib 35 |
| Nc1cc(Oc2cccc3C(=NNC(=O)c23)Cc4ccc(F)c(c4)C(=O)N5CCN(CC5)C(=O)C6CC6)ccc1C(=O)O | Olaparib 2382 |
| C\C(=C/C(=O)Nc1cccc2C(=NNC(=O)c12)Cc3ccc(F)c(c3)C(=O)N4CCN(CC4)C(=O)C5CC5)\CO | Olaparib 407 |
| C[C@H](N)[C@H](C)C(=O)Oc1cccc2C(=NNC(=O)c12)Cc3ccc(F)c(c3)C(=O)N4CCN(CC4)C(=O)C5CC5 | Olaparib 1334 |
| Nc1cccc(COC(=O)c2cccc3C(=NNC(=O)c23)Cc4ccc(F)c(c4)C(=O)N5CCN(CC5)C(=O)C6CC6)n1 | Olaparib 958 |
| COC(=O)\C=C(/C)\NC(=O)c1cccc2C(=NNC(=O)c12)Cc3ccc(F)c(c3)C(=O)N4CCN(CC4)C(=O)C5CC5 | Olaparib 79 |
| C\C(=C/C(=O)Oc1cccc2C(=NNC(=O)c12)Cc3ccc(F)c(c3)C(=O)N4CCN(CC4)C(=O)C5CC5)\CO | Olaparib 1346 |
| C\C(=N/O)\C(C)(C)Oc1cccc2C(=NNC(=O)c12)Cc3ccc(F)c(c3)C(=O)N4CCN(CC4)C(=O)C5CC5 | Olaparib 2807 |
| Nc1cccc(OC(=O)c2cccc3C(=NNC(=O)c23)Cc4ccc(F)c(c4)C(=O)N5CCN(CC5)C(=O)C6CC6)c1O | Olaparib 957 |
| CN(C)C(=O)\C=C\C(=O)Oc1cccc2C(=NNC(=O)c12)Cc3ccc(F)c(c3)C(=O)N4CCN(CC4)C(=O)C5CC5 | Olaparib 1237 |
| COC\C=C\C(=O)Oc1cccc2C(=NNC(=O)c12)Cc3ccc(F)c(c3)C(=O)N4CCN(CC4)C(=O)C5CC5 | Olaparib 1347 |
| C[C@@H](Oc1cccc2C(=NNC(=O)c12)Cc3ccc(F)c(c3)C(=O)N4CCN(CC4)C(=O)C5CC5)C(=O)N(C)C | Olaparib 2804 |
| Fc1ccc(CC2=NNC(=O)c3c(cccc23)C(=O)OCCC#Cc4cnccn4)cc1C(=O)N5CCN(CC5)C(=O)C6CC6 | Olaparib 885 |
| CCNC(=O)\C=C\C(=O)Nc1cccc2C(=NNC(=O)c12)Cc3ccc(F)c(c3)C(=O)N4CCN(CC4)C(=O)C5CC5 | Olaparib 313 |
| OC\C=C\C(=O)Nc1cccc2C(=NNC(=O)c12)Cc3ccc(F)c(c3)C(=O)N4CCN(CC4)C(=O)C5CC5 | Olaparib 447 |
| CN(C)CC#CCNC(=O)c1cccc2C(=NNC(=O)c12)Cc3ccc(F)c(c3)C(=O)N4CCN(CC4)C(=O)C5CC5 | Olaparib 84 |
| O\N=C\c1cc(Oc2cccc3C(=NNC(=O)c23)Cc4ccc(F)c(c4)C(=O)N5CCN(CC5)C(=O)C6CC6)ccc1O | Olaparib 2381 |
| C\C(=C/C(=O)O)\CC(=O)Nc1cccc2C(=NNC(=O)c12)Cc3ccc(F)c(c3)C(=O)N4CCN(CC4)C(=O)C5CC5 | Olaparib 310 |
| Fc1ccc(CC2=NNC(=O)c3c(NC(=O)CCC=O)cccc23)cc1C(=O)N4CCN(CC4)C(=O)C5CC5 | Olaparib 445 |
| C[C@H](CN)CC(=O)Oc1cccc2C(=NNC(=O)c12)Cc3ccc(F)c(c3)C(=O)N4CCN(CC4)C(=O)C5CC5 | Olaparib 1336 |
| Nc1cnccc1OC(=O)c2cccc3C(=NNC(=O)c23)Cc4ccc(F)c(c4)C(=O)N5CCN(CC5)C(=O)C6CC6 | Olaparib 978 |
| CNC(=O)\C=C\C(=O)Nc1cccc2C(=NNC(=O)c12)Cc3ccc(F)c(c3)C(=O)N4CCN(CC4)C(=O)C5CC5 | Olaparib 367 |
| Fc1ccc(CC2=NNC(=O)c3c(OC(=O)CCC=O)cccc23)cc1C(=O)N4CCN(CC4)C(=O)C5CC5 | Olaparib 1392 |
| C[C@@H](CNC(=O)C)Oc1cccc2C(=NNC(=O)c12)Cc3ccc(F)c(c3)C(=O)N4CCN(CC4)C(=O)C5CC5 | Olaparib 2805 |
| Fc1ccc(CC2=NNC(=O)c3c(cccc23)C(=O)OCc4cnccn4)cc1C(=O)N5CCN(CC5)C(=O)C6CC6 | Olaparib 980 |
| COC(=O)\C=C\C(=O)Nc1cccc2C(=NNC(=O)c12)Cc3ccc(F)c(c3)C(=O)N4CCN(CC4)C(=O)C5CC5 | Olaparib 364 |
| OC\C=C\C(=O)Oc1cccc2C(=NNC(=O)c12)Cc3ccc(F)c(c3)C(=O)N4CCN(CC4)C(=O)C5CC5 | Olaparib 1394 |
| CC[C@@H](O)CC(=O)Nc1cccc2C(=NNC(=O)c12)Cc3ccc(F)c(c3)C(=O)N4CCN(CC4)C(=O)C5CC5 | Olaparib 393 |
| CCCCOc1ccc(Oc2cccc3C(=NNC(=O)c23)Cc4ccc(F)c(c4)C(=O)N5CCN(CC5)C(=O)C6CC6)nn1 | Olaparib 2342 |
| OC(=O)\C=C\CNC(=O)c1cccc2C(=NNC(=O)c12)Cc3ccc(F)c(c3)C(=O)N4CCN(CC4)C(=O)C5CC5 | Olaparib 122 |
| CCN[C@H](C)C(C)(C)OC(=O)c1cccc2C(=NNC(=O)c12)Cc3ccc(F)c(c3)C(=O)N4CCN(CC4)C(=O)C5CC5 | Olaparib 592 |
| CN(C)CC(=O)Nc1cccc2C(=NNC(=O)c12)Cc3ccc(F)c(c3)C(=O)N4CCN(CC4)C(=O)C5CC5 | Olaparib 442 |
| Fc1ccc(CC2=NNC(=O)c3c(Oc4c[nH]c(c4)C(=O)NCCC#C)cccc23)cc1C(=O)N5CCN(CC5)C(=O)C6CC6 | Olaparib 2322 |
| OC(=O)\C(=C(/Oc1cccc2C(=NNC(=O)c12)Cc3ccc(F)c(c3)C(=O)N4CCN(CC4)C(=O)C5CC5)\C=O)\Cl | Olaparib 2132 |
| C[C@@H](CN)C[C@H](C)OC(=O)c1cccc2C(=NNC(=O)c12)Cc3ccc(F)c(c3)C(=O)N4CCN(CC4)C(=O)C5CC5 | Olaparib 632 |
| C[C@H](N)CC(=O)Nc1cccc2C(=NNC(=O)c12)Cc3ccc(F)c(c3)C(=O)N4CCN(CC4)C(=O)C5CC5 | Olaparib 439 |
| COC[C@H](OC(=O)c1cccc2C(=NNC(=O)c12)Cc3ccc(F)c(c3)C(=O)N4CCN(CC4)C(=O)C5CC5)c6occc6Br | Olaparib 698 |
| CC[C@@H](C)N(C)C(=O)C(=O)Oc1cccc2C(=NNC(=O)c12)Cc3ccc(F)c(c3)C(=O)N4CCN(CC4)C(=O)C5CC5 | Olaparib 1199 |
| CC[C@H](C)[C@H](N)COC(=O)c1cccc2C(=NNC(=O)c12)Cc3ccc(F)c(c3)C(=O)N4CCN(CC4)C(=O)C5CC5 | Olaparib 631 |
| CC(C)(Oc1cccc2C(=NNC(=O)c12)Cc3ccc(F)c(c3)C(=O)N4CCN(CC4)C(=O)C5CC5)C(=O)N | Olaparib 2846 |
| NCc1oc(COC(=O)c2cccc3C(=NNC(=O)c23)Cc4ccc(F)c(c4)C(=O)N5CCN(CC5)C(=O)C6CC6)cc1 | Olaparib 953 |
| CC(C)[C@@H](COC(=O)c1cccc2C(=NNC(=O)c12)Cc3ccc(F)c(c3)C(=O)N4CCN(CC4)C(=O)C5CC5)NC=O | Olaparib 593 |
| CC(C)(C)SCCOC(=O)c1cccc2C(=NNC(=O)c12)Cc3ccc(F)c(c3)C(=O)N4CCN(CC4)C(=O)C5CC5 | Olaparib 574 |
| CC[C@@H](O)CC(=O)Oc1cccc2C(=NNC(=O)c12)Cc3ccc(F)c(c3)C(=O)N4CCN(CC4)C(=O)C5CC5 | Olaparib 1328 |
| Cc1cc(Oc2cccc3C(=NNC(=O)c23)Cc4ccc(F)c(c4)C(=O)N5CCN(CC5)C(=O)C6CC6)nc(O)c1C#N | Olaparib 2977 |
| CP(=O)(O)CCCNC(=O)c1cccc2C(=NNC(=O)c12)Cc3ccc(F)c(c3)C(=O)N4CCN(CC4)C(=O)C5CC5 | Olaparib 15 |
| CC(C)(C)[C@H](O)C(=O)Nc1cccc2C(=NNC(=O)c12)Cc3ccc(F)c(c3)C(=O)N4CCN(CC4)C(=O)C5CC5 | Olaparib 342 |
| CCC[C@@H](O)C(=O)Oc1cccc2C(=NNC(=O)c12)Cc3ccc(F)c(c3)C(=O)N4CCN(CC4)C(=O)C5CC5 | Olaparib 1330 |
| Fc1ccc(CC2=NNC(=O)c3c(cccc23)C(=O)OCCc4cn[nH]c4)cc1C(=O)N5CCN(CC5)C(=O)C6CC6 | Olaparib 977 |
| CC(C)(CNC(=O)c1cccc2C(=NNC(=O)c12)Cc3ccc(F)c(c3)C(=O)N4CCN(CC4)C(=O)C5CC5)C(=O)O | Olaparib 69 |
| CC(=O)NC(C)(C)COc1cccc2C(=NNC(=O)c12)Cc3ccc(F)c(c3)C(=O)N4CCN(CC4)C(=O)C5CC5 | Olaparib 2773 |
| OC[C@@H](Oc1cccc2C(=NNC(=O)c12)Cc3ccc(F)c(c3)C(=O)N4CCN(CC4)C(=O)C5CC5)[C@H](O)C=C | Olaparib 2800 |
| CN1C=CC=C(COC(=O)c2cccc3C(=NNC(=O)c23)Cc4ccc(F)c(c4)C(=O)N5CCN(CC5)C(=O)C6CC6)C1=O | Olaparib 918 |
| C[C@H](OC(=O)c1cccc2C(=NNC(=O)c12)Cc3ccc(F)c(c3)C(=O)N4CCN(CC4)C(=O)C5CC5)C(=O)N(C)C | Olaparib 633 |
| CC(C)(CN)COC(=O)c1cccc2C(=NNC(=O)c12)Cc3ccc(F)c(c3)C(=O)N4CCN(CC4)C(=O)C5CC5 | Olaparib 674 |
| CN[C@@H](C)C(=O)Nc1cccc2C(=NNC(=O)c12)Cc3ccc(F)c(c3)C(=O)N4CCN(CC4)C(=O)C5CC5 | Olaparib 441 |
| CC1=CC(=CC(=O)N1)OC(=O)c2cccc3C(=NNC(=O)c23)Cc4ccc(F)c(c4)C(=O)N5CCN(CC5)C(=O)C6CC6 | Olaparib 955 |
| COC(=O)C(C)(C)NC(=O)c1cccc2C(=NNC(=O)c12)Cc3ccc(F)c(c3)C(=O)N4CCN(CC4)C(=O)C5CC5 | Olaparib 71 |
| CC(C)(N)CCC(=O)Oc1cccc2C(=NNC(=O)c12)Cc3ccc(F)c(c3)C(=O)N4CCN(CC4)C(=O)C5CC5 | Olaparib 1282 |
| CC(C)(C#N)C(=O)Nc1cccc2C(=NNC(=O)c12)Cc3ccc(F)c(c3)C(=O)N4CCN(CC4)C(=O)C5CC5 | Olaparib 423 |
| OC1=C(O)C(=O)C(=CC=C1)Oc2cccc3C(=NNC(=O)c23)Cc4ccc(F)c(c4)C(=O)N5CCN(CC5)C(=O)C6CC6 | Olaparib 2961 |
| CN(CCC(=O)Oc1cccc2C(=NNC(=O)c12)Cc3ccc(F)c(c3)C(=O)N4CCN(CC4)C(=O)C5CC5)C(=O)C | Olaparib 1228 |
| CC[C@](C)(NC)C(=O)Oc1cccc2C(=NNC(=O)c12)Cc3ccc(F)c(c3)C(=O)N4CCN(CC4)C(=O)C5CC5 | Olaparib 1276 |
| CN[C@@H](C)C(=O)Oc1cccc2C(=NNC(=O)c12)Cc3ccc(F)c(c3)C(=O)N4CCN(CC4)C(=O)C5CC5 | Olaparib 1388 |
| CC1(C)CC(=O)C=C(C1)NCCOC(=O)c2cccc3C(=NNC(=O)c23)Cc4ccc(F)c(c4)C(=O)N5CCN(CC5)C(=O)C6CC6 | Olaparib 754 |
| C[C@@H](CCC(=O)O)NC(=O)c1cccc2C(=NNC(=O)c12)Cc3ccc(F)c(c3)C(=O)N4CCN(CC4)C(=O)C5CC5 | Olaparib 63 |
| N[C@@H](CCCc1cccc2C(=NNC(=O)c12)Cc3ccc(F)c(c3)C(=O)N4CCN(CC4)C(=O)C5CC5)CC(=O)N | Olaparib 1793 |
| CC(C)(C#N)C(=O)Oc1cccc2C(=NNC(=O)c12)Cc3ccc(F)c(c3)C(=O)N4CCN(CC4)C(=O)C5CC5 | Olaparib 1369 |
| CCC1=C(Oc2cccc3C(=NNC(=O)c23)Cc4ccc(F)c(c4)C(=O)N5CCN(CC5)C(=O)C6CC6)C(=O)C=CN1CCO | Olaparib 2891 |
| CC(C)NC(=N)NC(=O)c1cccc2C(=NNC(=O)c12)Cc3ccc(F)c(c3)C(=O)N4CCN(CC4)C(=O)C5CC5 | Olaparib 121 |
| CC[C@](C)(O)COC(=O)c1cccc2C(=NNC(=O)c12)Cc3ccc(F)c(c3)C(=O)N4CCN(CC4)C(=O)C5CC5 | Olaparib 669 |
| CCSCC(=O)Nc1cccc2C(=NNC(=O)c12)Cc3ccc(F)c(c3)C(=O)N4CCN(CC4)C(=O)C5CC5 | Olaparib 383 |
| CC(=O)C1=C(O)C[C@H](CC1=O)Oc2cccc3C(=NNC(=O)c23)Cc4ccc(F)c(c4)C(=O)N5CCN(CC5)C(=O)C6CC6 | Olaparib 2917 |
| C[C@H](CNC(=O)c1cccc2C(=NNC(=O)c12)Cc3ccc(F)c(c3)C(=O)N4CCN(CC4)C(=O)C5CC5)CC(=O)O | Olaparib 67 |
| CCC[C@@](C)(O)C(=O)Oc1cccc2C(=NNC(=O)c12)Cc3ccc(F)c(c3)C(=O)N4CCN(CC4)C(=O)C5CC5 | Olaparib 1270 |
| CC(=O)NCCOc1cccc2C(=NNC(=O)c12)Cc3ccc(F)c(c3)C(=O)N4CCN(CC4)C(=O)C5CC5 | Olaparib 2849 |
| CCC1=C(NC(=O)C=C1O)Oc2cccc3C(=NNC(=O)c23)Cc4ccc(F)c(c4)C(=O)N5CCN(CC5)C(=O)C6CC6 | Olaparib 2374 |
| NC[C@H](O)[C@H](OC(=O)c1cccc2C(=NNC(=O)c12)Cc3ccc(F)c(c3)C(=O)N4CCN(CC4)C(=O)C5CC5)C=C | Olaparib 637 |
| CC[C@H](O)CCOC(=O)c1cccc2C(=NNC(=O)c12)Cc3ccc(F)c(c3)C(=O)N4CCN(CC4)C(=O)C5CC5 | Olaparib 668 |
| NC[C@H](C(=O)O)c1cccc2C(=NNC(=O)c12)Cc3ccc(F)c(c3)C(=O)N4CCN(CC4)C(=O)C5CC5 | Olaparib 1644 |
| C[C@H]1OC(=C(OC(=O)c2cccc3C(=NNC(=O)c23)Cc4ccc(F)c(c4)C(=O)N5CCN(CC5)C(=O)C6CC6)C1=O)C | Olaparib 952 |
| CCOC(=O)\C=C(/N)\C(F)(F)Oc1cccc2C(=NNC(=O)c12)Cc3ccc(F)c(c3)C(=O)N4CCN(CC4)C(=O)C5CC5 | Olaparib 2118 |
| CCSC[C@H](C)OC(=O)c1cccc2C(=NNC(=O)c12)Cc3ccc(F)c(c3)C(=O)N4CCN(CC4)C(=O)C5CC5 | Olaparib 607 |
| Fc1ccc(CC2=NNC(=O)c3c(OCCCNC=O)cccc23)cc1C(=O)N4CCN(CC4)C(=O)C5CC5 | Olaparib 2847 |
| CC1=CC(=O)N[C@@H]1OC(=O)c2cccc3C(=NNC(=O)c23)Cc4ccc(F)c(c4)C(=O)N5CCN(CC5)C(=O)C6CC6 | Olaparib 976 |
| OC[C@H](O)[C@H](OC(=O)c1cccc2C(=NNC(=O)c12)Cc3ccc(F)c(c3)C(=O)N4CCN(CC4)C(=O)C5CC5)C=C | Olaparib 629 |
| CC(C)SCCOC(=O)c1cccc2C(=NNC(=O)c12)Cc3ccc(F)c(c3)C(=O)N4CCN(CC4)C(=O)C5CC5 | Olaparib 606 |
| CCOC(=O)C(F)(F)Oc1cccc2C(=NNC(=O)c12)Cc3ccc(F)c(c3)C(=O)N4CCN(CC4)C(=O)C5CC5 | Olaparib 2108 |
| Fc1ccc(CC2=NNC(=O)c3c(CCOC4=C(Cl)C(=O)NN=C4)cccc23)cc1C(=O)N5CCN(CC5)C(=O)C6CC6 | Olaparib 1694 |
| CCOCC(=N)NC(=O)c1cccc2C(=NNC(=O)c12)Cc3ccc(F)c(c3)C(=O)N4CCN(CC4)C(=O)C5CC5 | Olaparib 116 |
| CC(=O)NCCCOc1cccc2C(=NNC(=O)c12)Cc3ccc(F)c(c3)C(=O)N4CCN(CC4)C(=O)C5CC5 | Olaparib 2806 |
| CCOC(=O)[C@@](F)(Br)Oc1cccc2C(=NNC(=O)c12)Cc3ccc(F)c(c3)C(=O)N4CCN(CC4)C(=O)C5CC5 | Olaparib 2096 |
| Fc1ccc(CC2=NNC(=O)c3c(CCOC4=CC(=O)NN=C4)cccc23)cc1C(=O)N5CCN(CC5)C(=O)C6CC6 | Olaparib 1720 |
| CN(CC(=O)Oc1cccc2C(=NNC(=O)c12)Cc3ccc(F)c(c3)C(=O)N4CCN(CC4)C(=O)C5CC5)C(=O)C | Olaparib 1284 |
| CC(C)(N)C(=O)Oc1cccc2C(=NNC(=O)c12)Cc3ccc(F)c(c3)C(=O)N4CCN(CC4)C(=O)C5CC5 | Olaparib 1390 |
| N[C@H](Cc1cccc2C(=NNC(=O)c12)Cc3ccc(F)c(c3)C(=O)N4CCN(CC4)C(=O)C5CC5)C(=O)O | Olaparib 1643 |
| CC1=C(OC(=O)c2cccc3C(=NNC(=O)c23)Cc4ccc(F)c(c4)C(=O)N5CCN(CC5)C(=O)C6CC6)C(=O)CO1 | Olaparib 973 |
| CN(C)\N=C\C(=O)C(F)(F)Oc1cccc2C(=NNC(=O)c12)Cc3ccc(F)c(c3)C(=O)N4CCN(CC4)C(=O)C5CC5 | Olaparib 2133 |
| C[C@H](N)CC(=O)Oc1cccc2C(=NNC(=O)c12)Cc3ccc(F)c(c3)C(=O)N4CCN(CC4)C(=O)C5CC5 | Olaparib 1386 |
| CCSCC(=O)Oc1cccc2C(=NNC(=O)c12)Cc3ccc(F)c(c3)C(=O)N4CCN(CC4)C(=O)C5CC5 | Olaparib 1318 |
| Fc1ccc(CC2=NNC(=O)c3c(cccc23)C(=O)O[C@H]4OCC(=O)C=C4)cc1C(=O)N5CCN(CC5)C(=O)C6CC6 | Olaparib 972 |
| CC(=O)NCCC(=O)Nc1cccc2C(=NNC(=O)c12)Cc3ccc(F)c(c3)C(=O)N4CCN(CC4)C(=O)C5CC5 | Olaparib 352 |
| C[C@H](O)CC(=O)Nc1cccc2C(=NNC(=O)c12)Cc3ccc(F)c(c3)C(=O)N4CCN(CC4)C(=O)C5CC5 | Olaparib 437 |
| COC(=O)NCc1cccc2C(=NNC(=O)c12)Cc3ccc(F)c(c3)C(=O)N4CCN(CC4)C(=O)C5CC5 | Olaparib 1823 |
| CC1=C(Cl)N=C(Oc2cccc3C(=NNC(=O)c23)Cc4ccc(F)c(c4)C(=O)N5CCN(CC5)C(=O)C6CC6)C(=O)O1 | Olaparib 2361 |
| CCCNC(=O)C(=O)Nc1cccc2C(=NNC(=O)c12)Cc3ccc(F)c(c3)C(=O)N4CCN(CC4)C(=O)C5CC5 | Olaparib 353 |
| C[C@H](CO)C(=O)Nc1cccc2C(=NNC(=O)c12)Cc3ccc(F)c(c3)C(=O)N4CCN(CC4)C(=O)C5CC5 | Olaparib 434 |
| NOC\C=C\Oc1cccc2C(=NNC(=O)c12)Cc3ccc(F)c(c3)C(=O)N4CCN(CC4)C(=O)C5CC5 | Olaparib 2164 |
| CC(C)(C)CC(=O)N1CCC(C)(CC1)C(=O)Oc2cccc3C(=NNC(=O)c23)Cc4ccc(F)c(c4)C(=O)N5CCN(CC5)C(=O)C6CC6 | Olaparib 1408 |
| OC(=O)CC[C@H](C(=O)O)c1cccc2C(=NNC(=O)c12)Cc3ccc(F)c(c3)C(=O)N4CCN(CC4)C(=O)C5CC5 | Olaparib 1625 |
| C[C@@H](O)CC(=O)Nc1cccc2C(=NNC(=O)c12)Cc3ccc(F)c(c3)C(=O)N4CCN(CC4)C(=O)C5CC5 | Olaparib 435 |
| COC(=O)C(F)(F)Oc1cccc2C(=NNC(=O)c12)Cc3ccc(F)c(c3)C(=O)N4CCN(CC4)C(=O)C5CC5 | Olaparib 2152 |
| CCC(C)(C)N1C[C@H](COC(=O)c2cccc3C(=NNC(=O)c23)Cc4ccc(F)c(c4)C(=O)N5CCN(CC5)C(=O)C6CC6)CC1=O | Olaparib 748 |
| C[C@H](OC(=O)C)C(=O)Oc1cccc2C(=NNC(=O)c12)Cc3ccc(F)c(c3)C(=O)N4CCN(CC4)C(=O)C5CC5 | Olaparib 1274 |
| CCSCCOC(=O)c1cccc2C(=NNC(=O)c12)Cc3ccc(F)c(c3)C(=O)N4CCN(CC4)C(=O)C5CC5 | Olaparib 648 |
| COC(=O)[C@](F)(Cl)Oc1cccc2C(=NNC(=O)c12)Cc3ccc(F)c(c3)C(=O)N4CCN(CC4)C(=O)C5CC5 | Olaparib 2151 |
| CC[C@@H]1CCN(CCOC(=O)c2cccc3C(=NNC(=O)c23)Cc4ccc(F)c(c4)C(=O)N5CCN(CC5)C(=O)C6CC6)C(=O)CC1 | Olaparib 747 |
| CC(=O)NCCOC(=O)c1cccc2C(=NNC(=O)c12)Cc3ccc(F)c(c3)C(=O)N4CCN(CC4)C(=O)C5CC5 | Olaparib 679 |
| CSCCCOC(=O)c1cccc2C(=NNC(=O)c12)Cc3ccc(F)c(c3)C(=O)N4CCN(CC4)C(=O)C5CC5 | Olaparib 647 |
| C[N+](C)(C)C[C@H](O)COc1cccc2C(=NNC(=O)c12)Cc3ccc(F)c(c3)C(=O)N4CCN(CC4)C(=O)C5CC5 | Olaparib 2760 |
| CC[C@H](C)N1C[C@H](COC(=O)c2cccc3C(=NNC(=O)c23)Cc4ccc(F)c(c4)C(=O)N5CCN(CC5)C(=O)C6CC6)CC1=O | Olaparib 791 |
| CCCNC(=O)C(=O)Oc1cccc2C(=NNC(=O)c12)Cc3ccc(F)c(c3)C(=O)N4CCN(CC4)C(=O)C5CC5 | Olaparib 1286 |
| C[C@H](O)CC(=O)Oc1cccc2C(=NNC(=O)c12)Cc3ccc(F)c(c3)C(=O)N4CCN(CC4)C(=O)C5CC5 | Olaparib 1384 |
| COC(OC)[C@@H](C)Oc1cccc2C(=NNC(=O)c12)Cc3ccc(F)c(c3)C(=O)N4CCN(CC4)C(=O)C5CC5 | Olaparib 2783 |
| CC1(C)O[C@H](COC(=O)c2cccc3C(=NNC(=O)c23)Cc4ccc(F)c(c4)C(=O)N5CCN(CC5)C(=O)C6CC6)[C@@H](O1)C=C | Olaparib 843 |
| COC(=O)CC(=O)C(F)(F)Oc1cccc2C(=NNC(=O)c12)Cc3ccc(F)c(c3)C(=O)N4CCN(CC4)C(=O)C5CC5 | Olaparib 2130 |
| C[C@@H](O)CC(=O)Oc1cccc2C(=NNC(=O)c12)Cc3ccc(F)c(c3)C(=O)N4CCN(CC4)C(=O)C5CC5 | Olaparib 1382 |
| CC(CO)(CO)COc1cccc2C(=NNC(=O)c12)Cc3ccc(F)c(c3)C(=O)N4CCN(CC4)C(=O)C5CC5 | Olaparib 2784 |
| Fc1ccc(CC2=NNC(=O)c3c(cccc23)C(=O)O[C@H](CN4CCC(=O)CC4)C(F)(F)F)cc1C(=O)N5CCN(CC5)C(=O)C6CC6 | Olaparib 709 |
| OCC(=O)CCOC(=O)c1cccc2C(=NNC(=O)c12)Cc3ccc(F)c(c3)C(=O)N4CCN(CC4)C(=O)C5CC5 | Olaparib 673 |
| C[C@H](CO)C(=O)Oc1cccc2C(=NNC(=O)c12)Cc3ccc(F)c(c3)C(=O)N4CCN(CC4)C(=O)C5CC5 | Olaparib 1381 |
| C[C@@H](O)[C@H](N)COc1cccc2C(=NNC(=O)c12)Cc3ccc(F)c(c3)C(=O)N4CCN(CC4)C(=O)C5CC5 | Olaparib 2834 |
| CC1(C)OC[C@@H](O1)\C=C/COC(=O)c2cccc3C(=NNC(=O)c23)Cc4ccc(F)c(c4)C(=O)N5CCN(CC5)C(=O)C6CC6 | Olaparib 845 |
| CCOC(=O)\C(=N\O)\c1cccc2C(=NNC(=O)c12)Cc3ccc(F)c(c3)C(=O)N4CCN(CC4)C(=O)C5CC5 | Olaparib 1624 |
| NC(=O)CCCOc1cccc2C(=NNC(=O)c12)Cc3ccc(F)c(c3)C(=O)N4CCN(CC4)C(=O)C5CC5 | Olaparib 2848 |
| CNC[C@@H](O)COc1cccc2C(=NNC(=O)c12)Cc3ccc(F)c(c3)C(=O)N4CCN(CC4)C(=O)C5CC5 | Olaparib 2832 |
| OC1(CCCCC1)C(=O)COC(=O)c2cccc3C(=NNC(=O)c23)Cc4ccc(F)c(c4)C(=O)N5CCN(CC5)C(=O)C6CC6 | Olaparib 846 |
| Fc1ccc(CC2=NNC(=O)c3c(OC(F)(F)C(=O)NC(=O)C(F)(F)F)cccc23)cc1C(=O)N4CCN(CC4)C(=O)C5CC5 | Olaparib 2104 |
| OCCC(=O)COc1cccc2C(=NNC(=O)c12)Cc3ccc(F)c(c3)C(=O)N4CCN(CC4)C(=O)C5CC5 | Olaparib 2842 |
| C[C@@H](O)[C@H](O)COc1cccc2C(=NNC(=O)c12)Cc3ccc(F)c(c3)C(=O)N4CCN(CC4)C(=O)C5CC5 | Olaparib 2830 |
| CC1=N[C@](C)(COC(=O)c2cccc3C(=NNC(=O)c23)Cc4ccc(F)c(c4)C(=O)N5CCN(CC5)C(=O)C6CC6)CO1 | Olaparib 950 |
| COC(=O)C(=O)Nc1cccc2C(=NNC(=O)c12)Cc3ccc(F)c(c3)C(=O)N4CCN(CC4)C(=O)C5CC5 | Olaparib 438 |
| OCC(=O)CCOc1cccc2C(=NNC(=O)c12)Cc3ccc(F)c(c3)C(=O)N4CCN(CC4)C(=O)C5CC5 | Olaparib 2843 |
| OCC[C@@H](O)COc1cccc2C(=NNC(=O)c12)Cc3ccc(F)c(c3)C(=O)N4CCN(CC4)C(=O)C5CC5 | Olaparib 2825 |
| Fc1ccc(CC2=NNC(=O)c3c(cccc23)C(=O)O[C@H]4CN(CC(F)(F)F)C(=O)C4)cc1C(=O)N5CCN(CC5)C(=O)C6CC6 | Olaparib 758 |
| OC(=O)C(F)(C(=O)O)c1cccc2C(=NNC(=O)c12)Cc3ccc(F)c(c3)C(=O)N4CCN(CC4)C(=O)C5CC5 | Olaparib 1636 |
| Fc1ccc(CC2=NNC(=O)c3c(NC(=O)CCCC#N)cccc23)cc1C(=O)N4CCN(CC4)C(=O)C5CC5 | Olaparib 424 |
| CO[C@@H](CO)COc1cccc2C(=NNC(=O)c12)Cc3ccc(F)c(c3)C(=O)N4CCN(CC4)C(=O)C5CC5 | Olaparib 2827 |
| Fc1ccc(CC2=NNC(=O)c3c(cccc23)C(=O)OC[C@@H]4CCC(=O)N4)cc1C(=O)N5CCN(CC5)C(=O)C6CC6 | Olaparib 969 |
| COC(=O)\C(=N\O)\c1cccc2C(=NNC(=O)c12)Cc3ccc(F)c(c3)C(=O)N4CCN(CC4)C(=O)C5CC5 | Olaparib 1637 |
| Fc1ccc(CC2=NNC(=O)c3c(OC(=O)CCCC#N)cccc23)cc1C(=O)N4CCN(CC4)C(=O)C5CC5 | Olaparib 1370 |
| C\C=C\C=C\C=C\C(=O)Oc1cccc2C(=NNC(=O)c12)Cc3ccc(F)c(c3)C(=O)N4CCN(CC4)C(=O)C5CC5 | Olaparib 1251 |
| O[C@H]1OC(=O)C(=C1Cl)Oc2cccc3C(=NNC(=O)c23)Cc4ccc(F)c(c4)C(=O)N5CCN(CC5)C(=O)C6CC6 | Olaparib 2388 |
| OC(=O)C(=O)COc1cccc2C(=NNC(=O)c12)Cc3ccc(F)c(c3)C(=O)N4CCN(CC4)C(=O)C5CC5 | Olaparib 2167 |
| O[C@H](OC(=O)c1cccc2C(=NNC(=O)c12)Cc3ccc(F)c(c3)C(=O)N4CCN(CC4)C(=O)C5CC5)C(Cl)(Cl)Cl | Olaparib 564 |
| CC(=C\C=C\C(=O)Nc1cccc2C(=NNC(=O)c12)Cc3ccc(F)c(c3)C(=O)N4CCN(CC4)C(=O)C5CC5)C | Olaparib 375 |
| C[C@@H]1CNC[C@H](C)N1CC(C)(C)OC(=O)c2cccc3C(=NNC(=O)c23)Cc4ccc(F)c(c4)C(=O)N5CCN(CC5)C(=O)C6CC6 | Olaparib 742 |
| COC(=O)C(=O)Oc1cccc2C(=NNC(=O)c12)Cc3ccc(F)c(c3)C(=O)N4CCN(CC4)C(=O)C5CC5 | Olaparib 1385 |
| COC(=O)C(Cl)(Cl)Oc1cccc2C(=NNC(=O)c12)Cc3ccc(F)c(c3)C(=O)N4CCN(CC4)C(=O)C5CC5 | Olaparib 2125 |
| CC(=C\C=C\C(=O)Oc1cccc2C(=NNC(=O)c12)Cc3ccc(F)c(c3)C(=O)N4CCN(CC4)C(=O)C5CC5)C | Olaparib 1310 |
| CC[C@@H](CN1CC[C@H](C)[C@@H](N)C1)OC(=O)c2cccc3C(=NNC(=O)c23)Cc4ccc(F)c(c4)C(=O)N5CCN(CC5)C(=O)C6CC6 | Olaparib 741 |
| CC(CO)(CO)C(=O)Nc1cccc2C(=NNC(=O)c12)Cc3ccc(F)c(c3)C(=O)N4CCN(CC4)C(=O)C5CC5 | Olaparib 334 |
| C[C@@H](Oc1cccc2C(=NNC(=O)c12)Cc3ccc(F)c(c3)C(=O)N4CCN(CC4)C(=O)C5CC5)[C@H](N)CN | Olaparib 2836 |
| CC(=C)C(=O)OC\C=C\c1cccc2C(=NNC(=O)c12)Cc3ccc(F)c(c3)C(=O)N4CCN(CC4)C(=O)C5CC5 | Olaparib 1809 |
| C[C@H](CN1CC[C@H](C)[C@@H](N)C1)OC(=O)c2cccc3C(=NNC(=O)c23)Cc4ccc(F)c(c4)C(=O)N5CCN(CC5)C(=O)C6CC6 | Olaparib 786 |
| CC(CO)(CO)NC(=O)c1cccc2C(=NNC(=O)c12)Cc3ccc(F)c(c3)C(=O)N4CCN(CC4)C(=O)C5CC5 | Olaparib 92 |
| C[C@@H](O)[C@@H](CO)Oc1cccc2C(=NNC(=O)c12)Cc3ccc(F)c(c3)C(=O)N4CCN(CC4)C(=O)C5CC5 | Olaparib 2828 |
| C\C=C\C(=C\C(=O)Oc1cccc2C(=NNC(=O)c12)Cc3ccc(F)c(c3)C(=O)N4CCN(CC4)C(=O)C5CC5)\C | Olaparib 1311 |
| CC(C)[C@H]1C[C@](O)(CC(=O)Nc2cccc3C(=NNC(=O)c23)Cc4ccc(F)c(c4)C(=O)N5CCN(CC5)C(=O)C6CC6)CCO1 | Olaparib 479 |
| C[C@@H](O)[C@@H](CO)OC(=O)c1cccc2C(=NNC(=O)c12)Cc3ccc(F)c(c3)C(=O)N4CCN(CC4)C(=O)C5CC5 | Olaparib 658 |
| OC[C@H](O)CCOc1cccc2C(=NNC(=O)c12)Cc3ccc(F)c(c3)C(=O)N4CCN(CC4)C(=O)C5CC5 | Olaparib 2826 |
| CCOC(=O)\C=C\C(=C)c1cccc2C(=NNC(=O)c12)Cc3ccc(F)c(c3)C(=O)N4CCN(CC4)C(=O)C5CC5 | Olaparib 1618 |
| CN[C@@]1(COC(=O)c2cccc3C(=NNC(=O)c23)Cc4ccc(F)c(c4)C(=O)N5CCN(CC5)C(=O)C6CC6)CCOC(C)(C)C1 | Olaparib 784 |
| CC(CO)(CO)C(=O)Oc1cccc2C(=NNC(=O)c12)Cc3ccc(F)c(c3)C(=O)N4CCN(CC4)C(=O)C5CC5 | Olaparib 1260 |
| OC[C@@H](O)CCOc1cccc2C(=NNC(=O)c12)Cc3ccc(F)c(c3)C(=O)N4CCN(CC4)C(=O)C5CC5 | Olaparib 2822 |
| CCC(=C=CC(=O)Oc1cccc2C(=NNC(=O)c12)Cc3ccc(F)c(c3)C(=O)N4CCN(CC4)C(=O)C5CC5)C | Olaparib 1309 |
| C[C@H](CN[C@H]1CCO[C@@H](C)C1)OC(=O)c2cccc3C(=NNC(=O)c23)Cc4ccc(F)c(c4)C(=O)N5CCN(CC5)C(=O)C6CC6 | Olaparib 782 |
| C[C@H](O)[C@@H](Oc1cccc2C(=NNC(=O)c12)Cc3ccc(F)c(c3)C(=O)N4CCN(CC4)C(=O)C5CC5)[C@@H](O)C=O | Olaparib 2761 |
| OCC[C@H](O)COc1cccc2C(=NNC(=O)c12)Cc3ccc(F)c(c3)C(=O)N4CCN(CC4)C(=O)C5CC5 | Olaparib 2821 |
| CC\C=C\C=C\C(=O)Oc1cccc2C(=NNC(=O)c12)Cc3ccc(F)c(c3)C(=O)N4CCN(CC4)C(=O)C5CC5 | Olaparib 1312 |
| CCN[C@H](C)[C@]1(CCCOC1)OC(=O)c2cccc3C(=NNC(=O)c23)Cc4ccc(F)c(c4)C(=O)N5CCN(CC5)C(=O)C6CC6 | Olaparib 783 |
| NCC[C@@H](N)C(=O)Nc1cccc2C(=NNC(=O)c12)Cc3ccc(F)c(c3)C(=O)N4CCN(CC4)C(=O)C5CC5 | Olaparib 392 |
| CC(=O)[C@@H](O)[C@@H](CO)Oc1cccc2C(=NNC(=O)c12)Cc3ccc(F)c(c3)C(=O)N4CCN(CC4)C(=O)C5CC5 | Olaparib 2763 |
| OCC[C@@H](CO)Oc1cccc2C(=NNC(=O)c12)Cc3ccc(F)c(c3)C(=O)N4CCN(CC4)C(=O)C5CC5 | Olaparib 2820 |
| C\C=C\C=C\C(=O)Nc1cccc2C(=NNC(=O)c12)Cc3ccc(F)c(c3)C(=O)N4CCN(CC4)C(=O)C5CC5 | Olaparib 425 |
| C[C@H]1CN(C[C@H](OC(=O)c2cccc3C(=NNC(=O)c23)Cc4ccc(F)c(c4)C(=O)N5CCN(CC5)C(=O)C6CC6)C(F)(F)F)[C@H](C)CO1 | Olaparib 692 |
| NC[C@H](N)CC(=O)Nc1cccc2C(=NNC(=O)c12)Cc3ccc(F)c(c3)C(=O)N4CCN(CC4)C(=O)C5CC5 | Olaparib 391 |
| C[C@@H](O)[C@H](N)C(=O)Nc1cccc2C(=NNC(=O)c12)Cc3ccc(F)c(c3)C(=O)N4CCN(CC4)C(=O)C5CC5 | Olaparib 389 |
| OCP(CO)COc1cccc2C(=NNC(=O)c12)Cc3ccc(F)c(c3)C(=O)N4CCN(CC4)C(=O)C5CC5 | Olaparib 2779 |
| C\C=C\C=C\C(=O)Oc1cccc2C(=NNC(=O)c12)Cc3ccc(F)c(c3)C(=O)N4CCN(CC4)C(=O)C5CC5 | Olaparib 1371 |
| COC[C@H](C)N1CCC[C@H](C1)OC(=O)c2cccc3C(=NNC(=O)c23)Cc4ccc(F)c(c4)C(=O)N5CCN(CC5)C(=O)C6CC6 | Olaparib 781 |
| CC(=O)N[C@@H](CO)COc1cccc2C(=NNC(=O)c12)Cc3ccc(F)c(c3)C(=O)N4CCN(CC4)C(=O)C5CC5 | Olaparib 2765 |
| C\C=C\C=C\C=C\C(=O)Nc1cccc2C(=NNC(=O)c12)Cc3ccc(F)c(c3)C(=O)N4CCN(CC4)C(=O)C5CC5 | Olaparib 327 |
| CC(C)C[C@H](OC(=O)c1cccc2C(=NNC(=O)c12)Cc3ccc(F)c(c3)C(=O)N4CCN(CC4)C(=O)C5CC5)C=C | Olaparib 644 |
| CC(C)[C@H]1C[C@](O)(CC(=O)Oc2cccc3C(=NNC(=O)c23)Cc4ccc(F)c(c4)C(=O)N5CCN(CC5)C(=O)C6CC6)CCO1 | Olaparib 1449 |
| NCC[C@@H](N)C(=O)Oc1cccc2C(=NNC(=O)c12)Cc3ccc(F)c(c3)C(=O)N4CCN(CC4)C(=O)C5CC5 | Olaparib 1327 |
| CCC(=C=CC(=O)Nc1cccc2C(=NNC(=O)c12)Cc3ccc(F)c(c3)C(=O)N4CCN(CC4)C(=O)C5CC5)C | Olaparib 374 |
| CC(C)C\C(=C\C(=O)Oc1cccc2C(=NNC(=O)c12)Cc3ccc(F)c(c3)C(=O)N4CCN(CC4)C(=O)C5CC5)\C | Olaparib 1243 |
| C[C@@H](N)CCC(=O)N1CC[C@H](C1)Oc2cccc3C(=NNC(=O)c23)Cc4ccc(F)c(c4)C(=O)N5CCN(CC5)C(=O)C6CC6 | Olaparib 2887 |
| COC[C@@H](CO)OC(=O)c1cccc2C(=NNC(=O)c12)Cc3ccc(F)c(c3)C(=O)N4CCN(CC4)C(=O)C5CC5 | Olaparib 653 |
| C\C=C\C(=C\C(=O)Nc1cccc2C(=NNC(=O)c12)Cc3ccc(F)c(c3)C(=O)N4CCN(CC4)C(=O)C5CC5)\C | Olaparib 376 |
| CC[C@H](C)\C=C(/C)\C(=O)Oc1cccc2C(=NNC(=O)c12)Cc3ccc(F)c(c3)C(=O)N4CCN(CC4)C(=O)C5CC5 | Olaparib 1242 |
| NCC[C@H]1CCCN(C1)C(=O)C(F)(F)Oc2cccc3C(=NNC(=O)c23)Cc4ccc(F)c(c4)C(=O)N5CCN(CC5)C(=O)C6CC6 | Olaparib 2247 |
| C[C@@H](CNC(=O)N)Oc1cccc2C(=NNC(=O)c12)Cc3ccc(F)c(c3)C(=O)N4CCN(CC4)C(=O)C5CC5 | Olaparib 2798 |
| CC\C=C\C=C\C(=O)Nc1cccc2C(=NNC(=O)c12)Cc3ccc(F)c(c3)C(=O)N4CCN(CC4)C(=O)C5CC5 | Olaparib 377 |
| C\C=C\[C@@H](C)CC(=O)Nc1cccc2C(=NNC(=O)c12)Cc3ccc(F)c(c3)C(=O)N4CCN(CC4)C(=O)C5CC5 | Olaparib 368 |
| CCOC(=O)[C@]1(C)CCNC[C@]1(F)Oc2cccc3C(=NNC(=O)c23)Cc4ccc(F)c(c4)C(=O)N5CCN(CC5)C(=O)C6CC6 | Olaparib 2286 |
| N[C@H]([C@H](O)CF)C(=O)Nc1cccc2C(=NNC(=O)c12)Cc3ccc(F)c(c3)C(=O)N4CCN(CC4)C(=O)C5CC5 | Olaparib 330 |
| CC(C)(C)CC(=C)COC(=O)c1cccc2C(=NNC(=O)c12)Cc3ccc(F)c(c3)C(=O)N4CCN(CC4)C(=O)C5CC5 | Olaparib 600 |
| CC(C)(C)\C=C\C(=O)Oc1cccc2C(=NNC(=O)c12)Cc3ccc(F)c(c3)C(=O)N4CCN(CC4)C(=O)C5CC5 | Olaparib 1307 |
| COC1(CCCC[C@H]1OC(=O)c2cccc3C(=NNC(=O)c23)Cc4ccc(F)c(c4)C(=O)N5CCN(CC5)C(=O)C6CC6)OC | Olaparib 836 |
| CN[C@H](CO)C(=O)Nc1cccc2C(=NNC(=O)c12)Cc3ccc(F)c(c3)C(=O)N4CCN(CC4)C(=O)C5CC5 | Olaparib 388 |
| CC(=CCCC(=O)Nc1cccc2C(=NNC(=O)c12)Cc3ccc(F)c(c3)C(=O)N4CCN(CC4)C(=O)C5CC5)C | Olaparib 369 |
| OC[C@@H](CN1CCCCC1)OC(=O)c2cccc3C(=NNC(=O)c23)Cc4ccc(F)c(c4)C(=O)N5CCN(CC5)C(=O)C6CC6 | Olaparib 839 |
| NCC[C@@H](O)C(=O)Nc1cccc2C(=NNC(=O)c12)Cc3ccc(F)c(c3)C(=O)N4CCN(CC4)C(=O)C5CC5 | Olaparib 390 |
| CC(C)C\C=C\C(=O)Nc1cccc2C(=NNC(=O)c12)Cc3ccc(F)c(c3)C(=O)N4CCN(CC4)C(=O)C5CC5 | Olaparib 370 |
| O[C@@H](COC(=O)c1cccc2C(=NNC(=O)c12)Cc3ccc(F)c(c3)C(=O)N4CCN(CC4)C(=O)C5CC5)CN6CCCCC6 | Olaparib 840 |
| C[C@@H](O)[C@H](N)C(=O)Oc1cccc2C(=NNC(=O)c12)Cc3ccc(F)c(c3)C(=O)N4CCN(CC4)C(=O)C5CC5 | Olaparib 1324 |
| CC(C)\C=C\CC(=O)Nc1cccc2C(=NNC(=O)c12)Cc3ccc(F)c(c3)C(=O)N4CCN(CC4)C(=O)C5CC5 | Olaparib 371 |
| C[C@@H](COC(=O)c1cccc2C(=NNC(=O)c12)Cc3ccc(F)c(c3)C(=O)N4CCN(CC4)C(=O)C5CC5)O[C@H]6CCCCO6 | Olaparib 834 |
| NCC[C@@H](O)C(=O)Oc1cccc2C(=NNC(=O)c12)Cc3ccc(F)c(c3)C(=O)N4CCN(CC4)C(=O)C5CC5 | Olaparib 1325 |
| C[C@@H](CC(=C)C)OC(=O)c1cccc2C(=NNC(=O)c12)Cc3ccc(F)c(c3)C(=O)N4CCN(CC4)C(=O)C5CC5 | Olaparib 683 |
| CC(=C)C(=O)OCCCc1cccc2C(=NNC(=O)c12)Cc3ccc(F)c(c3)C(=O)N4CCN(CC4)C(=O)C5CC5 | Olaparib 1794 |
| COC[C@H](OC(=O)c1cccc2C(=NNC(=O)c12)Cc3ccc(F)c(c3)C(=O)N4CCN(CC4)C(=O)C5CC5)C6CCOCC6 | Olaparib 835 |
| N[C@H]([C@H](O)CF)C(=O)Oc1cccc2C(=NNC(=O)c12)Cc3ccc(F)c(c3)C(=O)N4CCN(CC4)C(=O)C5CC5 | Olaparib 1254 |
| CC(=C(C)C(=O)Nc1cccc2C(=NNC(=O)c12)Cc3ccc(F)c(c3)C(=O)N4CCN(CC4)C(=O)C5CC5)C | Olaparib 416 |
| CC(=CCCC(=O)Oc1cccc2C(=NNC(=O)c12)Cc3ccc(F)c(c3)C(=O)N4CCN(CC4)C(=O)C5CC5)C | Olaparib 1303 |
| C[C@H](CN1CCNCC1)OC(=O)c2cccc3C(=NNC(=O)c23)Cc4ccc(F)c(c4)C(=O)N5CCN(CC5)C(=O)C6CC6 | Olaparib 899 |
| CC(=O)N[C@@H](O)C(Cl)(Cl)Oc1cccc2C(=NNC(=O)c12)Cc3ccc(F)c(c3)C(=O)N4CCN(CC4)C(=O)C5CC5 | Olaparib 2106 |
| CC(C)C\C=C\C(=O)Oc1cccc2C(=NNC(=O)c12)Cc3ccc(F)c(c3)C(=O)N4CCN(CC4)C(=O)C5CC5 | Olaparib 1304 |
| CC1(C)OC[C@@H](CCOC(=O)c2cccc3C(=NNC(=O)c23)Cc4ccc(F)c(c4)C(=O)N5CCN(CC5)C(=O)C6CC6)O1 | Olaparib 895 |
| NC(=O)NCCCOc1cccc2C(=NNC(=O)c12)Cc3ccc(F)c(c3)C(=O)N4CCN(CC4)C(=O)C5CC5 | Olaparib 2799 |
| CC(C)\C=C\CC(=O)Oc1cccc2C(=NNC(=O)c12)Cc3ccc(F)c(c3)C(=O)N4CCN(CC4)C(=O)C5CC5 | Olaparib 1305 |
| Fc1ccc(CC2=NNC(=O)c3c(cccc23)C(=O)OCCCN4CCNCC4)cc1C(=O)N5CCN(CC5)C(=O)C6CC6 | Olaparib 900 |
| CN[C@H](CO)C(=O)Oc1cccc2C(=NNC(=O)c12)Cc3ccc(F)c(c3)C(=O)N4CCN(CC4)C(=O)C5CC5 | Olaparib 1323 |
| CC(=C(C)C(=O)Oc1cccc2C(=NNC(=O)c12)Cc3ccc(F)c(c3)C(=O)N4CCN(CC4)C(=O)C5CC5)C | Olaparib 1358 |
| Fc1ccc(CC2=NNC(=O)c3c(cccc23)C(=O)OCCCN4CCOCC4)cc1C(=O)N5CCN(CC5)C(=O)C6CC6 | Olaparib 897 |
| COC[C@@H](N)C(=O)Oc1cccc2C(=NNC(=O)c12)Cc3ccc(F)c(c3)C(=O)N4CCN(CC4)C(=O)C5CC5 | Olaparib 1322 |
| C[C@H](CC(=O)Nc1cccc2C(=NNC(=O)c12)Cc3ccc(F)c(c3)C(=O)N4CCN(CC4)C(=O)C5CC5)C=C | Olaparib 420 |
| N[C@@H]1CNCCC[C@@H]1OC(=O)c2cccc3C(=NNC(=O)c23)Cc4ccc(F)c(c4)C(=O)N5CCN(CC5)C(=O)C6CC6 | Olaparib 947 |
| OCC(=O)[C@@H](O)COc1cccc2C(=NNC(=O)c12)Cc3ccc(F)c(c3)C(=O)N4CCN(CC4)C(=O)C5CC5 | Olaparib 2787 |
| C\C=C\[C@@H](C)C(=O)Nc1cccc2C(=NNC(=O)c12)Cc3ccc(F)c(c3)C(=O)N4CCN(CC4)C(=O)C5CC5 | Olaparib 418 |
| CC1(C)OC[C@@H](COC(=O)c2cccc3C(=NNC(=O)c23)Cc4ccc(F)c(c4)C(=O)N5CCN(CC5)C(=O)C6CC6)O1 | Olaparib 945 |
| OC[C@@H](Oc1cccc2C(=NNC(=O)c12)Cc3ccc(F)c(c3)C(=O)N4CCN(CC4)C(=O)C5CC5)C(=O)CO | Olaparib 2786 |
| C[C@H](CC=C)C(=O)Nc1cccc2C(=NNC(=O)c12)Cc3ccc(F)c(c3)C(=O)N4CCN(CC4)C(=O)C5CC5 | Olaparib 412 |
| NC[C@@]1(CCCOC1)OC(=O)c2cccc3C(=NNC(=O)c23)Cc4ccc(F)c(c4)C(=O)N5CCN(CC5)C(=O)C6CC6 | Olaparib 946 |
| [15NH2][C@H](CO)C(=O)Nc1cccc2C(=NNC(=O)c12)Cc3ccc(F)c(c3)C(=O)N4CCN(CC4)C(=O)C5CC5 | Olaparib 432 |
| CC\C(=C\C(=O)Nc1cccc2C(=NNC(=O)c12)Cc3ccc(F)c(c3)C(=O)N4CCN(CC4)C(=O)C5CC5)\C | Olaparib 413 |
| Fc1ccc(CC2=NNC(=O)c3c(cccc23)C(=O)OCCN4CCOCC4)cc1C(=O)N5CCN(CC5)C(=O)C6CC6 | Olaparib 557 |
| O[C@H](COc1cccc2C(=NNC(=O)c12)Cc3ccc(F)c(c3)C(=O)N4CCN(CC4)C(=O)C5CC5)[C@@H](O)C=O | Olaparib 2790 |
| C[C@H](CC(=O)Nc1cccc2C(=NNC(=O)c12)Cc3ccc(F)c(c3)C(=O)N4CCN(CC4)C(=O)C5CC5)C=C | Olaparib 414 |
| O[C@H]1CNC[C@@H]1COC(=O)c2cccc3C(=NNC(=O)c23)Cc4ccc(F)c(c4)C(=O)N5CCN(CC5)C(=O)C6CC6 | Olaparib 968 |
| O[C@@H](COc1cccc2C(=NNC(=O)c12)Cc3ccc(F)c(c3)C(=O)N4CCN(CC4)C(=O)C5CC5)[C@H](O)C=O | Olaparib 2792 |
| C\C=C/CCCOC(=O)c1cccc2C(=NNC(=O)c12)Cc3ccc(F)c(c3)C(=O)N4CCN(CC4)C(=O)C5CC5 | Olaparib 684 |
| C[C@H](CC(=O)Oc1cccc2C(=NNC(=O)c12)Cc3ccc(F)c(c3)C(=O)N4CCN(CC4)C(=O)C5CC5)C=C | Olaparib 1365 |
| OC[C@H]1OC=C[C@H](Oc2cccc3C(=NNC(=O)c23)Cc4ccc(F)c(c4)C(=O)N5CCN(CC5)C(=O)C6CC6)[C@@H]1O | Olaparib 2985 |
| NC(=N)NCCOc1cccc2C(=NNC(=O)c12)Cc3ccc(F)c(c3)C(=O)N4CCN(CC4)C(=O)C5CC5 | Olaparib 2845 |
| CC(=C)CCC(=O)Oc1cccc2C(=NNC(=O)c12)Cc3ccc(F)c(c3)C(=O)N4CCN(CC4)C(=O)C5CC5 | Olaparib 1364 |
| Fc1ccc(CC2=NNC(=O)c3c(cccc23)C(=O)OCCN4CCOC4)cc1C(=O)N5CCN(CC5)C(=O)C6CC6 | Olaparib 966 |
| NC(=O)NCCOc1cccc2C(=NNC(=O)c12)Cc3ccc(F)c(c3)C(=O)N4CCN(CC4)C(=O)C5CC5 | Olaparib 2840 |
| C\C=C\[C@@H](C)C(=O)Oc1cccc2C(=NNC(=O)c12)Cc3ccc(F)c(c3)C(=O)N4CCN(CC4)C(=O)C5CC5 | Olaparib 1362 |
| CC(C)\C=C\C(=O)Oc1cccc2C(=NNC(=O)c12)Cc3ccc(F)c(c3)C(=O)N4CCN(CC4)C(=O)C5CC5 | Olaparib 1359 |
| OC[C@H]1OC=C[C@@H](O)[C@H]1Oc2cccc3C(=NNC(=O)c23)Cc4ccc(F)c(c4)C(=O)N5CCN(CC5)C(=O)C6CC6 | Olaparib 2984 |
| C[C@H](CC(=O)Oc1cccc2C(=NNC(=O)c12)Cc3ccc(F)c(c3)C(=O)N4CCN(CC4)C(=O)C5CC5)C=C | Olaparib 1356 |
| N[C@H]1COC[C@@H]1OC(=O)c2cccc3C(=NNC(=O)c23)Cc4ccc(F)c(c4)C(=O)N5CCN(CC5)C(=O)C6CC6 | Olaparib 985 |
| CC\C(=C\C(=O)Oc1cccc2C(=NNC(=O)c12)Cc3ccc(F)c(c3)C(=O)N4CCN(CC4)C(=O)C5CC5)\C | Olaparib 1355 |
| O[C@@H]1CNC[C@H]1OC(=O)c2cccc3C(=NNC(=O)c23)Cc4ccc(F)c(c4)C(=O)N5CCN(CC5)C(=O)C6CC6 | Olaparib 984 |
| C[C@H](CC=C)C(=O)Oc1cccc2C(=NNC(=O)c12)Cc3ccc(F)c(c3)C(=O)N4CCN(CC4)C(=O)C5CC5 | Olaparib 1354 |
| C\C=C\CCC(=O)Nc1cccc2C(=NNC(=O)c12)Cc3ccc(F)c(c3)C(=O)N4CCN(CC4)C(=O)C5CC5 | Olaparib 415 |
| Fc1ccc(CC2=NNC(=O)c3c(cccc23)C(=O)OC[C@H]4COCO4)cc1C(=O)N5CCN(CC5)C(=O)C6CC6 | Olaparib 983 |
| CC\C=C\CC(=O)Nc1cccc2C(=NNC(=O)c12)Cc3ccc(F)c(c3)C(=O)N4CCN(CC4)C(=O)C5CC5 | Olaparib 419 |
| O[C@@]1(CC=NO1)C(Cl)(Cl)Oc2cccc3C(=NNC(=O)c23)Cc4ccc(F)c(c4)C(=O)N5CCN(CC5)C(=O)C6CC6 | Olaparib 2300 |
| C\C=C\[C@H](CC#C)OC(=O)c1cccc2C(=NNC(=O)c12)Cc3ccc(F)c(c3)C(=O)N4CCN(CC4)C(=O)C5CC5 | Olaparib 646 |
| C[C@H](C=C)C(=O)Nc1cccc2C(=NNC(=O)c12)Cc3ccc(F)c(c3)C(=O)N4CCN(CC4)C(=O)C5CC5 | Olaparib 453 |
| CC1(C)O[C@H](CO)[C@@H](COc2cccc3C(=NNC(=O)c23)Cc4ccc(F)c(c4)C(=O)N5CCN(CC5)C(=O)C6CC6)O1 | Olaparib 2946 |
| Fc1ccc(CC2=NNC(=O)c3c(cccc23)C(=O)OCCCC(=C)Br)cc1C(=O)N4CCN(CC4)C(=O)C5CC5 | Olaparib 565 |
| CCC(=C)C(=O)Nc1cccc2C(=NNC(=O)c12)Cc3ccc(F)c(c3)C(=O)N4CCN(CC4)C(=O)C5CC5 | Olaparib 451 |
| C[C@H]1NC[C@H](Oc2cccc3C(=NNC(=O)c23)Cc4ccc(F)c(c4)C(=O)N5CCN(CC5)C(=O)C6CC6)[C@@H](O)[C@H]1O | Olaparib 2980 |
| C\C=C(/C)\C(=O)Nc1cccc2C(=NNC(=O)c12)Cc3ccc(F)c(c3)C(=O)N4CCN(CC4)C(=O)C5CC5 | Olaparib 452 |
| CC\C=C\CC(=O)Oc1cccc2C(=NNC(=O)c12)Cc3ccc(F)c(c3)C(=O)N4CCN(CC4)C(=O)C5CC5 | Olaparib 1363 |
| C[C@@H]1NC[C@H](Oc2cccc3C(=NNC(=O)c23)Cc4ccc(F)c(c4)C(=O)N5CCN(CC5)C(=O)C6CC6)[C@H](O)[C@@H]1O | Olaparib 2982 |
| CC(=CC(=O)Nc1cccc2C(=NNC(=O)c12)Cc3ccc(F)c(c3)C(=O)N4CCN(CC4)C(=O)C5CC5)C | Olaparib 450 |
| CC\C=C/CC(=O)Oc1cccc2C(=NNC(=O)c12)Cc3ccc(F)c(c3)C(=O)N4CCN(CC4)C(=O)C5CC5 | Olaparib 1361 |
| OC[C@H]1NCC[C@H](Oc2cccc3C(=NNC(=O)c23)Cc4ccc(F)c(c4)C(=O)N5CCN(CC5)C(=O)C6CC6)[C@@H]1O | Olaparib 2981 |
| C\C=C\CCC(=O)Oc1cccc2C(=NNC(=O)c12)Cc3ccc(F)c(c3)C(=O)N4CCN(CC4)C(=O)C5CC5 | Olaparib 1357 |
| C\C=C(/C)\C(=O)Oc1cccc2C(=NNC(=O)c12)Cc3ccc(F)c(c3)C(=O)N4CCN(CC4)C(=O)C5CC5 | Olaparib 1399 |
| OC[C@H]1NC[C@@H](Oc2cccc3C(=NNC(=O)c23)Cc4ccc(F)c(c4)C(=O)N5CCN(CC5)C(=O)C6CC6)[C@@H]1O | Olaparib 3009 |
| C[C@H](C=C)C(=O)Oc1cccc2C(=NNC(=O)c12)Cc3ccc(F)c(c3)C(=O)N4CCN(CC4)C(=O)C5CC5 | Olaparib 1400 |
| OC[C@H]1O[C@H](O)C[C@@H]1Oc2cccc3C(=NNC(=O)c23)Cc4ccc(F)c(c4)C(=O)N5CCN(CC5)C(=O)C6CC6 | Olaparib 3007 |
| CCC(=C)C(=O)Oc1cccc2C(=NNC(=O)c12)Cc3ccc(F)c(c3)C(=O)N4CCN(CC4)C(=O)C5CC5 | Olaparib 1398 |
| OC[C@H]1O[C@@H](C[C@@H]1O)Oc2cccc3C(=NNC(=O)c23)Cc4ccc(F)c(c4)C(=O)N5CCN(CC5)C(=O)C6CC6 | Olaparib 3008 |
| CC(=CC(=O)Oc1cccc2C(=NNC(=O)c12)Cc3ccc(F)c(c3)C(=O)N4CCN(CC4)C(=O)C5CC5)C | Olaparib 1397 |
| O[C@H]1CO[C@H](Oc2cccc3C(=NNC(=O)c23)Cc4ccc(F)c(c4)C(=O)N5CCN(CC5)C(=O)C6CC6)[C@@H]1O | Olaparib 3031 |
| CC\C=C\C(=O)Nc1cccc2C(=NNC(=O)c12)Cc3ccc(F)c(c3)C(=O)N4CCN(CC4)C(=O)C5CC5 | Olaparib 454 |
| O[C@H]1OC[C@@H](Oc2cccc3C(=NNC(=O)c23)Cc4ccc(F)c(c4)C(=O)N5CCN(CC5)C(=O)C6CC6)[C@H]1O | Olaparib 3030 |
| CC\C=C\C(=O)Oc1cccc2C(=NNC(=O)c12)Cc3ccc(F)c(c3)C(=O)N4CCN(CC4)C(=O)C5CC5 | Olaparib 1401 |
| O[C@H]1CO[C@H](O)[C@H]1Oc2cccc3C(=NNC(=O)c23)Cc4ccc(F)c(c4)C(=O)N5CCN(CC5)C(=O)C6CC6 | Olaparib 3029 |
| C\C(=C/C(=O)Nc1cccc2C(=NNC(=O)c12)Cc3ccc(F)c(c3)C(=O)N4CCN(CC4)C(=O)C5CC5)\Cl | Olaparib 382 |
| Fc1ccc(CC2=NNC(=O)c3c(Oc4ccc(C(=O)NCCC#C)c(Cl)c4)cccc23)cc1C(=O)N5CCN(CC5)C(=O)C6CC6 | Olaparib 2218 |
| C\C(=C/Br)\C(=O)Nc1cccc2C(=NNC(=O)c12)Cc3ccc(F)c(c3)C(=O)N4CCN(CC4)C(=O)C5CC5 | Olaparib 284 |
| Nc1ccccc1SC(F)(F)Oc2cccc3C(=NNC(=O)c23)Cc4ccc(F)c(c4)C(=O)N5CCN(CC5)C(=O)C6CC6 | Olaparib 2327 |
| C\C(=C/Br)\C(=O)Oc1cccc2C(=NNC(=O)c12)Cc3ccc(F)c(c3)C(=O)N4CCN(CC4)C(=O)C5CC5 | Olaparib 1194 |
| C\C(=C/C(=O)Oc1cccc2C(=NNC(=O)c12)Cc3ccc(F)c(c3)C(=O)N4CCN(CC4)C(=O)C5CC5)\Cl | Olaparib 1317 |
| Oc1ccc(OC(F)(F)Oc2cccc3C(=NNC(=O)c23)Cc4ccc(F)c(c4)C(=O)N5CCN(CC5)C(=O)C6CC6)cc1 | Olaparib 2366 |
| Fc1ccc(CC2=NNC(=O)c3c(COC(=O)C=C)cccc23)cc1C(=O)N4CCN(CC4)C(=O)C5CC5 | Olaparib 1827 |
| Fc1ccc(CC2=NNC(=O)c3c(OC(=O)C(=C)Cl)cccc23)cc1C(=O)N4CCN(CC4)C(=O)C5CC5 | Olaparib 1376 |
| Fc1ccc(CC2=NNC(=O)c3c(Oc4ccc(cc4)C(=O)\C=C\C(Cl)(Cl)Cl)cccc23)cc1C(=O)N5CCN(CC5)C(=O)C6CC6 | Olaparib 2183 |
| Fc1ccc(CC2=NNC(=O)c3c(NC(=O)\C=C\I)cccc23)cc1C(=O)N4CCN(CC4)C(=O)C5CC5 | Olaparib 273 |
| CC(C)(C)c1ccc(CCOC(=O)c2cccc3C(=NNC(=O)c23)Cc4ccc(F)c(c4)C(=O)N5CCN(CC5)C(=O)C6CC6)cc1 | Olaparib 771 |
| Fc1ccc(CC2=NNC(=O)c3c(NC(=O)\C=C\Cl)cccc23)cc1C(=O)N4CCN(CC4)C(=O)C5CC5 | Olaparib 430 |
| CCCCC(=O)c1ccc(Oc2cccc3C(=NNC(=O)c23)Cc4ccc(F)c(c4)C(=O)N5CCN(CC5)C(=O)C6CC6)cc1 | Olaparib 2356 |
| Fc1ccc(CC2=NNC(=O)c3c(OC(=O)\C=C\I)cccc23)cc1C(=O)N4CCN(CC4)C(=O)C5CC5 | Olaparib 1177 |
| CCOC(=O)CCCCCCc1cccc2C(=NNC(=O)c12)Cc3ccc(F)c(c3)C(=O)N4CCN(CC4)C(=O)C5CC5 | Olaparib 1901 |
| CCc1cc(Br)ccc1COC(=O)c2cccc3C(=NNC(=O)c23)Cc4ccc(F)c(c4)C(=O)N5CCN(CC5)C(=O)C6CC6 | Olaparib 704 |
| Fc1ccc(CC2=NNC(=O)c3c(OC(=O)\C=C\Cl)cccc23)cc1C(=O)N4CCN(CC4)C(=O)C5CC5 | Olaparib 1377 |
| CCC(CC)[C@H](C)OC(=O)c1cccc2C(=NNC(=O)c12)Cc3ccc(F)c(c3)C(=O)N4CCN(CC4)C(=O)C5CC5 | Olaparib 641 |
| C[C@H](OC(=O)c1cccc2C(=NNC(=O)c12)Cc3ccc(F)c(c3)C(=O)N4CCN(CC4)C(=O)C5CC5)[C]6[CH][CH][CH][CH]6 | Olaparib 981 |
| CCC[C@H](OC(=O)c1cccc2C(=NNC(=O)c12)Cc3ccc(F)c(c3)C(=O)N4CCN(CC4)C(=O)C5CC5)C(C)(C)C | Olaparib 597 |
| CC(C)(C)[C@H](CC#C)OC(=O)c1cccc2C(=NNC(=O)c12)Cc3ccc(F)c(c3)C(=O)N4CCN(CC4)C(=O)C5CC5 | Olaparib 602 |
| CC(C)(C)c1ccc(CCOc2cccc3C(=NNC(=O)c23)Cc4ccc(F)c(c4)C(=O)N5CCN(CC5)C(=O)C6CC6)cc1 | Olaparib 2902 |
| CC[C@H](CC(C)C)OC(=O)c1cccc2C(=NNC(=O)c12)Cc3ccc(F)c(c3)C(=O)N4CCN(CC4)C(=O)C5CC5 | Olaparib 642 |
| CC(C)[C@H](C)CC(=O)Oc1cccc2C(=NNC(=O)c12)Cc3ccc(F)c(c3)C(=O)N4CCN(CC4)C(=O)C5CC5 | Olaparib 1289 |
| COc1ccc(Cc2cccc3C(=NNC(=O)c23)Cc4ccc(F)c(c4)C(=O)N5CCN(CC5)C(=O)C6CC6)cc1C(F)(F)F | Olaparib 1855 |
| CC[C@H](C)[C@H](C)COC(=O)c1cccc2C(=NNC(=O)c12)Cc3ccc(F)c(c3)C(=O)N4CCN(CC4)C(=O)C5CC5 | Olaparib 640 |
| CC[C@@H](OC(=O)c1cccc2C(=NNC(=O)c12)Cc3ccc(F)c(c3)C(=O)N4CCN(CC4)C(=O)C5CC5)C(C)C | Olaparib 681 |
| CC(C)(C)c1ccc(Cc2cccc3C(=NNC(=O)c23)Cc4ccc(F)c(c4)C(=O)N5CCN(CC5)C(=O)C6CC6)cc1 | Olaparib 1862 |
| CC(C)(C)CCCOC(=O)c1cccc2C(=NNC(=O)c12)Cc3ccc(F)c(c3)C(=O)N4CCN(CC4)C(=O)C5CC5 | Olaparib 639 |
| CC(C)CCC(=O)Nc1cccc2C(=NNC(=O)c12)Cc3ccc(F)c(c3)C(=O)N4CCN(CC4)C(=O)C5CC5 | Olaparib 406 |
| CCc1ccc(Cc2cccc3C(=NNC(=O)c23)Cc4ccc(F)c(c4)C(=O)N5CCN(CC5)C(=O)C6CC6)cc1 | Olaparib 1882 |
| CN(Cc1cccc2C(=NNC(=O)c12)Cc3ccc(F)c(c3)C(=O)N4CCN(CC4)C(=O)C5CC5)C(=O)C(C)(C)C | Olaparib 1920 |
| CCC(CC)C(=O)Oc1cccc2C(=NNC(=O)c12)Cc3ccc(F)c(c3)C(=O)N4CCN(CC4)C(=O)C5CC5 | Olaparib 1342 |
| Fc1ccc(CC2=NNC(=O)c3c(CCCc4ccccc4)cccc23)cc1C(=O)N5CCN(CC5)C(=O)C6CC6 | Olaparib 1989 |
| CCCC(C)(C)NC(=O)c1cccc2C(=NNC(=O)c12)Cc3ccc(F)c(c3)C(=O)N4CCN(CC4)C(=O)C5CC5 | Olaparib 117 |
| CC(C)(C)C(=O)Nc1cccc2C(=NNC(=O)c12)Cc3ccc(F)c(c3)C(=O)N4CCN(CC4)C(=O)C5CC5 | Olaparib 444 |
| Fc1ccc(CC2=NNC(=O)c3c(Cc4ccc(Cl)c(Cl)c4)cccc23)cc1C(=O)N5CCN(CC5)C(=O)C6CC6 | Olaparib 1961 |
| CCC[C@H](C)C(=O)Nc1cccc2C(=NNC(=O)c12)Cc3ccc(F)c(c3)C(=O)N4CCN(CC4)C(=O)C5CC5 | Olaparib 404 |
| CC(C)CCC(=O)Oc1cccc2C(=NNC(=O)c12)Cc3ccc(F)c(c3)C(=O)N4CCN(CC4)C(=O)C5CC5 | Olaparib 1345 |
| Fc1ccc(CC2=NNC(=O)c3c(\C=C\CC4CCCCC4)cccc23)cc1C(=O)N5CCN(CC5)C(=O)C6CC6 | Olaparib 1981 |
| CC[C@@](C)(Br)C(=O)Nc1cccc2C(=NNC(=O)c12)Cc3ccc(F)c(c3)C(=O)N4CCN(CC4)C(=O)C5CC5 | Olaparib 276 |
| Fc1ccc(CC2=NNC(=O)c3c(CCCC4CCCC4)cccc23)cc1C(=O)N5CCN(CC5)C(=O)C6CC6 | Olaparib 1997 |
| CCOC(=O)CCCc1cccc2C(=NNC(=O)c12)Cc3ccc(F)c(c3)C(=O)N4CCN(CC4)C(=O)C5CC5 | Olaparib 1899 |
| CC(=O)OCCCCc1cccc2C(=NNC(=O)c12)Cc3ccc(F)c(c3)C(=O)N4CCN(CC4)C(=O)C5CC5 | Olaparib 1903 |
| CC(C)(NC(=O)C(=O)Oc1cccc2C(=NNC(=O)c12)Cc3ccc(F)c(c3)C(=O)N4CCN(CC4)C(=O)C5CC5)C(=O)N | Olaparib 1183 |
| CC(C)(OC(=O)c1cccc2C(=NNC(=O)c12)Cc3ccc(F)c(c3)C(=O)N4CCN(CC4)C(=O)C5CC5)C(F)(F)F | Olaparib 601 |
| CC(C)(Br)C(=O)Nc1cccc2C(=NNC(=O)c12)Cc3ccc(F)c(c3)C(=O)N4CCN(CC4)C(=O)C5CC5 | Olaparib 283 |
| CN(C)S(=O)(=O)CC(=O)Oc1cccc2C(=NNC(=O)c12)Cc3ccc(F)c(c3)C(=O)N4CCN(CC4)C(=O)C5CC5 | Olaparib 1191 |
| CCOC(=O)CCc1cccc2C(=NNC(=O)c12)Cc3ccc(F)c(c3)C(=O)N4CCN(CC4)C(=O)C5CC5 | Olaparib 1902 |
| CC[C@@H](OC(=O)c1cccc2C(=NNC(=O)c12)Cc3ccc(F)c(c3)C(=O)N4CCN(CC4)C(=O)C5CC5)C(Cl)(Cl)Cl | Olaparib 562 |
| CS(=O)(=O)NCCOC(=O)c1cccc2C(=NNC(=O)c12)Cc3ccc(F)c(c3)C(=O)N4CCN(CC4)C(=O)C5CC5 | Olaparib 571 |
| Fc1ccc(CC2=NNC(=O)c3c(NC(=O)CCCC#C)cccc23)cc1C(=O)N4CCN(CC4)C(=O)C5CC5 | Olaparib 426 |
| OC(=O)CNC(=S)NC(=O)c1cccc2C(=NNC(=O)c12)Cc3ccc(F)c(c3)C(=O)N4CCN(CC4)C(=O)C5CC5 | Olaparib 18 |
| CC#CCCC(=O)Nc1cccc2C(=NNC(=O)c12)Cc3ccc(F)c(c3)C(=O)N4CCN(CC4)C(=O)C5CC5 | Olaparib 427 |
| OS(=O)(=O)CCOC(=O)c1cccc2C(=NNC(=O)c12)Cc3ccc(F)c(c3)C(=O)N4CCN(CC4)C(=O)C5CC5 | Olaparib 603 |
| COC(=O)CCc1cccc2C(=NNC(=O)c12)Cc3ccc(F)c(c3)C(=O)N4CCN(CC4)C(=O)C5CC5 | Olaparib 1825 |
| CC#CCCC(=O)Oc1cccc2C(=NNC(=O)c12)Cc3ccc(F)c(c3)C(=O)N4CCN(CC4)C(=O)C5CC5 | Olaparib 1374 |
| OCC(CO)(NC(=O)c1cccc2C(=NNC(=O)c12)Cc3ccc(F)c(c3)C(=O)N4CCN(CC4)C(=O)C5CC5)C(=O)O | Olaparib 17 |
| Fc1ccc(CC2=NNC(=O)c3c(OC(=O)CCCC#C)cccc23)cc1C(=O)N4CCN(CC4)C(=O)C5CC5 | Olaparib 1372 |
| OCNC(=O)NCOC(=O)c1cccc2C(=NNC(=O)c12)Cc3ccc(F)c(c3)C(=O)N4CCN(CC4)C(=O)C5CC5 | Olaparib 611 |
| CC(Cl)(Cl)C(=O)Oc1cccc2C(=NNC(=O)c12)Cc3ccc(F)c(c3)C(=O)N4CCN(CC4)C(=O)C5CC5 | Olaparib 1240 |
| CCS(=O)(=O)CCOC(=O)c1cccc2C(=NNC(=O)c12)Cc3ccc(F)c(c3)C(=O)N4CCN(CC4)C(=O)C5CC5 | Olaparib 572 |
| CCN[C@H](C)C(C)(C)Oc1cccc2C(=NNC(=O)c12)Cc3ccc(F)c(c3)C(=O)N4CCN(CC4)C(=O)C5CC5 | Olaparib 2771 |
| CON(C)C(=O)\C=C\C(=O)Nc1cccc2C(=NNC(=O)c12)Cc3ccc(F)c(c3)C(=O)N4CCN(CC4)C(=O)C5CC5 | Olaparib 288 |
| CN(C)CC(C)(C)COc1cccc2C(=NNC(=O)c12)Cc3ccc(F)c(c3)C(=O)N4CCN(CC4)C(=O)C5CC5 | Olaparib 2770 |
| CC(C)(NC(=O)NC(C)(C)C(=O)Oc1cccc2C(=NNC(=O)c12)Cc3ccc(F)c(c3)C(=O)N4CCN(CC4)C(=O)C5CC5)C#C | Olaparib 1175 |
| NCCCCC[C@@H](N)Cc1cccc2C(=NNC(=O)c12)Cc3ccc(F)c(c3)C(=O)N4CCN(CC4)C(=O)C5CC5 | Olaparib 1792 |
| NCCCCCCNCc1cccc2C(=NNC(=O)c12)Cc3ccc(F)c(c3)C(=O)N4CCN(CC4)C(=O)C5CC5 | Olaparib 1804 |
| C[C@@H](NC(=O)NC(C)(C)C#C)C(=O)Oc1cccc2C(=NNC(=O)c12)Cc3ccc(F)c(c3)C(=O)N4CCN(CC4)C(=O)C5CC5 | Olaparib 1176 |
| C[C@@H](CN)C[C@H](C)Oc1cccc2C(=NNC(=O)c12)Cc3ccc(F)c(c3)C(=O)N4CCN(CC4)C(=O)C5CC5 | Olaparib 2803 |
| C[C@@H](O)C(C)(C)COc1cccc2C(=NNC(=O)c12)Cc3ccc(F)c(c3)C(=O)N4CCN(CC4)C(=O)C5CC5 | Olaparib 2797 |
| CC(C)(COC(=O)c1cccc2C(=NNC(=O)c12)Cc3ccc(F)c(c3)C(=O)N4CCN(CC4)C(=O)C5CC5)NC(=O)N | Olaparib 587 |
| CC[C@](C)(CO)COc1cccc2C(=NNC(=O)c12)Cc3ccc(F)c(c3)C(=O)N4CCN(CC4)C(=O)C5CC5 | Olaparib 2795 |
| C[C@@H](O)[C@](C)(NC(=O)c1cccc2C(=NNC(=O)c12)Cc3ccc(F)c(c3)C(=O)N4CCN(CC4)C(=O)C5CC5)C(=O)O | Olaparib 22 |
| CC(C)(CN)COc1cccc2C(=NNC(=O)c12)Cc3ccc(F)c(c3)C(=O)N4CCN(CC4)C(=O)C5CC5 | Olaparib 2844 |
| CC[C@@H](COC(=O)c1cccc2C(=NNC(=O)c12)Cc3ccc(F)c(c3)C(=O)N4CCN(CC4)C(=O)C5CC5)NC(=O)N | Olaparib 588 |
| CC[C@@](C)(O)COc1cccc2C(=NNC(=O)c12)Cc3ccc(F)c(c3)C(=O)N4CCN(CC4)C(=O)C5CC5 | Olaparib 2839 |
| C[C@H](OC(=O)c1cccc2C(=NNC(=O)c12)Cc3ccc(F)c(c3)C(=O)N4CCN(CC4)C(=O)C5CC5)C(=O)NCCO | Olaparib 584 |
| CC(C)SCCOc1cccc2C(=NNC(=O)c12)Cc3ccc(F)c(c3)C(=O)N4CCN(CC4)C(=O)C5CC5 | Olaparib 2780 |
| C[C@H](O)[C@@H](OC(=O)c1cccc2C(=NNC(=O)c12)Cc3ccc(F)c(c3)C(=O)N4CCN(CC4)C(=O)C5CC5)[C@@H](O)C=O | Olaparib 577 |
| CCSC[C@@H](C)Oc1cccc2C(=NNC(=O)c12)Cc3ccc(F)c(c3)C(=O)N4CCN(CC4)C(=O)C5CC5 | Olaparib 2781 |
| C[C@H](O)[C@H](O)[C@H](OC(=O)c1cccc2C(=NNC(=O)c12)Cc3ccc(F)c(c3)C(=O)N4CCN(CC4)C(=O)C5CC5)C=O | Olaparib 578 |
| CC[C@H](CCO)Oc1cccc2C(=NNC(=O)c12)Cc3ccc(F)c(c3)C(=O)N4CCN(CC4)C(=O)C5CC5 | Olaparib 2837 |
| C[C@H](OC(=O)c1cccc2C(=NNC(=O)c12)Cc3ccc(F)c(c3)C(=O)N4CCN(CC4)C(=O)C5CC5)[C@H](O)[C@@H](O)C=O | Olaparib 579 |
| CC[C@H](O)CCOc1cccc2C(=NNC(=O)c12)Cc3ccc(F)c(c3)C(=O)N4CCN(CC4)C(=O)C5CC5 | Olaparib 2838 |
| CC(=O)[C@@H](OC(=O)c1cccc2C(=NNC(=O)c12)Cc3ccc(F)c(c3)C(=O)N4CCN(CC4)C(=O)C5CC5)[C@H](O)CO | Olaparib 580 |
| NCCNCCc1cccc2C(=NNC(=O)c12)Cc3ccc(F)c(c3)C(=O)N4CCN(CC4)C(=O)C5CC5 | Olaparib 1824 |
| CC(=O)N[C@@H](CO)COC(=O)c1cccc2C(=NNC(=O)c12)Cc3ccc(F)c(c3)C(=O)N4CCN(CC4)C(=O)C5CC5 | Olaparib 583 |
| CSCCCOc1cccc2C(=NNC(=O)c12)Cc3ccc(F)c(c3)C(=O)N4CCN(CC4)C(=O)C5CC5 | Olaparib 2817 |
| CC(=O)[C@@H](O)[C@H](O)COC(=O)c1cccc2C(=NNC(=O)c12)Cc3ccc(F)c(c3)C(=O)N4CCN(CC4)C(=O)C5CC5 | Olaparib 582 |
| CCSCCOc1cccc2C(=NNC(=O)c12)Cc3ccc(F)c(c3)C(=O)N4CCN(CC4)C(=O)C5CC5 | Olaparib 2818 |
| C[C@@H](O)C(=O)NCCOC(=O)c1cccc2C(=NNC(=O)c12)Cc3ccc(F)c(c3)C(=O)N4CCN(CC4)C(=O)C5CC5 | Olaparib 585 |
| Fc1ccc(CC2=NNC(=O)c3c(CCSCCC#N)cccc23)cc1C(=O)N4CCN(CC4)C(=O)C5CC5 | Olaparib 1842 |
| CC(=O)CCCc1cccc2C(=NNC(=O)c12)Cc3ccc(F)c(c3)C(=O)N4CCN(CC4)C(=O)C5CC5 | Olaparib 1932 |
| C[C@](O)(CC(=O)Nc1cccc2C(=NNC(=O)c12)Cc3ccc(F)c(c3)C(=O)N4CCN(CC4)C(=O)C5CC5)C(=O)O | Olaparib 294 |
| O[C@H](Oc1cccc2C(=NNC(=O)c12)Cc3ccc(F)c(c3)C(=O)N4CCN(CC4)C(=O)C5CC5)C(Cl)(Cl)Cl | Olaparib 2751 |
| Fc1ccc(CC2=NNC(=O)c3c(CCCSC=C)cccc23)cc1C(=O)N4CCN(CC4)C(=O)C5CC5 | Olaparib 1818 |
| C[C@](O)(CC(=O)Nc1cccc2C(=NNC(=O)c12)Cc3ccc(F)c(c3)C(=O)N4CCN(CC4)C(=O)C5CC5)C(=O)O | Olaparib 295 |
| C\C=C/CCCOc1cccc2C(=NNC(=O)c12)Cc3ccc(F)c(c3)C(=O)N4CCN(CC4)C(=O)C5CC5 | Olaparib 2854 |
| CC(=O)CCc1cccc2C(=NNC(=O)c12)Cc3ccc(F)c(c3)C(=O)N4CCN(CC4)C(=O)C5CC5 | Olaparib 1944 |
| C[C@@](CO)(NC(=O)c1cccc2C(=NNC(=O)c12)Cc3ccc(F)c(c3)C(=O)N4CCN(CC4)C(=O)C5CC5)C(=O)O | Olaparib 46 |
| Fc1ccc(CC2=NNC(=O)c3c(CCCC=O)cccc23)cc1C(=O)N4CCN(CC4)C(=O)C5CC5 | Olaparib 1816 |
| CNC(=O)NCCC(=O)Nc1cccc2C(=NNC(=O)c12)Cc3ccc(F)c(c3)C(=O)N4CCN(CC4)C(=O)C5CC5 | Olaparib 301 |
| COC\C=C\c1cccc2C(=NNC(=O)c12)Cc3ccc(F)c(c3)C(=O)N4CCN(CC4)C(=O)C5CC5 | Olaparib 1943 |
| C[C@@H](CNC(=O)N)OC(=O)c1cccc2C(=NNC(=O)c12)Cc3ccc(F)c(c3)C(=O)N4CCN(CC4)C(=O)C5CC5 | Olaparib 626 |
| CC(C)[C@](C)(O)CCc1cccc2C(=NNC(=O)c12)Cc3ccc(F)c(c3)C(=O)N4CCN(CC4)C(=O)C5CC5 | Olaparib 1921 |
| CC[C@@H](C)C(C)(C)Oc1cccc2C(=NNC(=O)c12)Cc3ccc(F)c(c3)C(=O)N4CCN(CC4)C(=O)C5CC5 | Olaparib 2813 |
| CNC(=O)NCCC(=O)Oc1cccc2C(=NNC(=O)c12)Cc3ccc(F)c(c3)C(=O)N4CCN(CC4)C(=O)C5CC5 | Olaparib 1221 |
| CC[C@H](CC(C)C)Oc1cccc2C(=NNC(=O)c12)Cc3ccc(F)c(c3)C(=O)N4CCN(CC4)C(=O)C5CC5 | Olaparib 2814 |
| CNC(=O)N[C@H](OC(=O)c1cccc2C(=NNC(=O)c12)Cc3ccc(F)c(c3)C(=O)N4CCN(CC4)C(=O)C5CC5)C(Cl)(Cl)Cl | Olaparib 558 |
| CC[C@](C)(O)CCc1cccc2C(=NNC(=O)c12)Cc3ccc(F)c(c3)C(=O)N4CCN(CC4)C(=O)C5CC5 | Olaparib 1924 |
| CC[C@@H](Oc1cccc2C(=NNC(=O)c12)Cc3ccc(F)c(c3)C(=O)N4CCN(CC4)C(=O)C5CC5)C(C)C | Olaparib 2851 |
| OCP(=O)(CO)COC(=O)c1cccc2C(=NNC(=O)c12)Cc3ccc(F)c(c3)C(=O)N4CCN(CC4)C(=O)C5CC5 | Olaparib 570 |
| CC(C)(O)CCc1cccc2C(=NNC(=O)c12)Cc3ccc(F)c(c3)C(=O)N4CCN(CC4)C(=O)C5CC5 | Olaparib 1930 |
| NCCCCc1cccc2C(=NNC(=O)c12)Cc3ccc(F)c(c3)C(=O)N4CCN(CC4)C(=O)C5CC5 | Olaparib 1813 |
| NC(=O)NCCCOC(=O)c1cccc2C(=NNC(=O)c12)Cc3ccc(F)c(c3)C(=O)N4CCN(CC4)C(=O)C5CC5 | Olaparib 627 |
| CC(C)OCCc1cccc2C(=NNC(=O)c12)Cc3ccc(F)c(c3)C(=O)N4CCN(CC4)C(=O)C5CC5 | Olaparib 1929 |
| CCC[C@H](C)\C=C\c1cccc2C(=NNC(=O)c12)Cc3ccc(F)c(c3)C(=O)N4CCN(CC4)C(=O)C5CC5 | Olaparib 1925 |
| O[C@H](COC(=O)c1cccc2C(=NNC(=O)c12)Cc3ccc(F)c(c3)C(=O)N4CCN(CC4)C(=O)C5CC5)[C@@H](O)C=O | Olaparib 617 |
| C[C@@H](O)CCc1cccc2C(=NNC(=O)c12)Cc3ccc(F)c(c3)C(=O)N4CCN(CC4)C(=O)C5CC5 | Olaparib 1942 |
| CC(C)C\C=C\c1cccc2C(=NNC(=O)c12)Cc3ccc(F)c(c3)C(=O)N4CCN(CC4)C(=O)C5CC5 | Olaparib 1935 |
| OC[C@@H](OC(=O)c1cccc2C(=NNC(=O)c12)Cc3ccc(F)c(c3)C(=O)N4CCN(CC4)C(=O)C5CC5)[C@H](O)C=O | Olaparib 618 |
| C[C@H](CCc1cccc2C(=NNC(=O)c12)Cc3ccc(F)c(c3)C(=O)N4CCN(CC4)C(=O)C5CC5)C#N | Olaparib 1810 |
| CC[C@H](C)\C=C\c1cccc2C(=NNC(=O)c12)Cc3ccc(F)c(c3)C(=O)N4CCN(CC4)C(=O)C5CC5 | Olaparib 1936 |
| OC[C@H](O)[C@H](OC(=O)c1cccc2C(=NNC(=O)c12)Cc3ccc(F)c(c3)C(=O)N4CCN(CC4)C(=O)C5CC5)C=O | Olaparib 619 |
| OCCCCc1cccc2C(=NNC(=O)c12)Cc3ccc(F)c(c3)C(=O)N4CCN(CC4)C(=O)C5CC5 | Olaparib 1941 |
| CCCC\C=C\c1cccc2C(=NNC(=O)c12)Cc3ccc(F)c(c3)C(=O)N4CCN(CC4)C(=O)C5CC5 | Olaparib 1937 |
| O[C@@H](COC(=O)c1cccc2C(=NNC(=O)c12)Cc3ccc(F)c(c3)C(=O)N4CCN(CC4)C(=O)C5CC5)[C@H](O)C=O | Olaparib 620 |
| CCOCCc1cccc2C(=NNC(=O)c12)Cc3ccc(F)c(c3)C(=O)N4CCN(CC4)C(=O)C5CC5 | Olaparib 1940 |
| Fc1ccc(CC2=NNC(=O)c3c(CCCCC=C)cccc23)cc1C(=O)N4CCN(CC4)C(=O)C5CC5 | Olaparib 1808 |
| OC[C@@H](O)[C@H](OC(=O)c1cccc2C(=NNC(=O)c12)Cc3ccc(F)c(c3)C(=O)N4CCN(CC4)C(=O)C5CC5)C=O | Olaparib 616 |
| Fc1ccc(CC2=NNC(=O)c3c(CCCC#N)cccc23)cc1C(=O)N4CCN(CC4)C(=O)C5CC5 | Olaparib 1829 |
| CCC\C=C\c1cccc2C(=NNC(=O)c12)Cc3ccc(F)c(c3)C(=O)N4CCN(CC4)C(=O)C5CC5 | Olaparib 1946 |
| NC(=N)NCCOC(=O)c1cccc2C(=NNC(=O)c12)Cc3ccc(F)c(c3)C(=O)N4CCN(CC4)C(=O)C5CC5 | Olaparib 675 |
| CC\C(=C\CCc1cccc2C(=NNC(=O)c12)Cc3ccc(F)c(c3)C(=O)N4CCN(CC4)C(=O)C5CC5)\C | Olaparib 1847 |
| CCC(C)(C)Cc1cccc2C(=NNC(=O)c12)Cc3ccc(F)c(c3)C(=O)N4CCN(CC4)C(=O)C5CC5 | Olaparib 1806 |
| N[C@@H](COC(=O)c1cccc2C(=NNC(=O)c12)Cc3ccc(F)c(c3)C(=O)N4CCN(CC4)C(=O)C5CC5)C(=O)N | Olaparib 671 |
| CCCC[C@@H](CC)Cc1cccc2C(=NNC(=O)c12)Cc3ccc(F)c(c3)C(=O)N4CCN(CC4)C(=O)C5CC5 | Olaparib 1905 |
| CCCC\C=C/c1cccc2C(=NNC(=O)c12)Cc3ccc(F)c(c3)C(=O)N4CCN(CC4)C(=O)C5CC5 | Olaparib 1938 |
| OCC(=O)NCCOC(=O)c1cccc2C(=NNC(=O)c12)Cc3ccc(F)c(c3)C(=O)N4CCN(CC4)C(=O)C5CC5 | Olaparib 622 |
| Fc1ccc(CC2=NNC(=O)c3c(CCCC=C)cccc23)cc1C(=O)N4CCN(CC4)C(=O)C5CC5 | Olaparib 1949 |
| OCCNC(=O)COC(=O)c1cccc2C(=NNC(=O)c12)Cc3ccc(F)c(c3)C(=O)N4CCN(CC4)C(=O)C5CC5 | Olaparib 621 |
| CCC\C=C/c1cccc2C(=NNC(=O)c12)Cc3ccc(F)c(c3)C(=O)N4CCN(CC4)C(=O)C5CC5 | Olaparib 1947 |
| OC[C@H](O)C(=O)COC(=O)c1cccc2C(=NNC(=O)c12)Cc3ccc(F)c(c3)C(=O)N4CCN(CC4)C(=O)C5CC5 | Olaparib 614 |
| CC(C)(C)CCc1cccc2C(=NNC(=O)c12)Cc3ccc(F)c(c3)C(=O)N4CCN(CC4)C(=O)C5CC5 | Olaparib 1931 |
| OCC(=O)[C@@H](O)COC(=O)c1cccc2C(=NNC(=O)c12)Cc3ccc(F)c(c3)C(=O)N4CCN(CC4)C(=O)C5CC5 | Olaparib 613 |
| CC(C)CCCc1cccc2C(=NNC(=O)c12)Cc3ccc(F)c(c3)C(=O)N4CCN(CC4)C(=O)C5CC5 | Olaparib 1848 |
| OC[C@@H](OC(=O)c1cccc2C(=NNC(=O)c12)Cc3ccc(F)c(c3)C(=O)N4CCN(CC4)C(=O)C5CC5)C(=O)CO | Olaparib 612 |
| C[Si](C)(C)C#CCc1cccc2C(=NNC(=O)c12)Cc3ccc(F)c(c3)C(=O)N4CCN(CC4)C(=O)C5CC5 | Olaparib 1846 |
| N[C@H](CC(=O)Nc1cccc2C(=NNC(=O)c12)Cc3ccc(F)c(c3)C(=O)N4CCN(CC4)C(=O)C5CC5)C(=O)O | Olaparib 339 |
| CCCCCCc1cccc2C(=NNC(=O)c12)Cc3ccc(F)c(c3)C(=O)N4CCN(CC4)C(=O)C5CC5 | Olaparib 1795 |
| CNC(=S)NCOC(=O)c1cccc2C(=NNC(=O)c12)Cc3ccc(F)c(c3)C(=O)N4CCN(CC4)C(=O)C5CC5 | Olaparib 608 |
| CCCCCc1cccc2C(=NNC(=O)c12)Cc3ccc(F)c(c3)C(=O)N4CCN(CC4)C(=O)C5CC5 | Olaparib 1814 |
| CN(N)C(=S)NC(=O)c1cccc2C(=NNC(=O)c12)Cc3ccc(F)c(c3)C(=O)N4CCN(CC4)C(=O)C5CC5 | Olaparib 90 |
| N[C@@H](CC(=O)Nc1cccc2C(=NNC(=O)c12)Cc3ccc(F)c(c3)C(=O)N4CCN(CC4)C(=O)C5CC5)C(=O)[O-] | Olaparib 346 |
| N[C@H](CC(=O)Oc1cccc2C(=NNC(=O)c12)Cc3ccc(F)c(c3)C(=O)N4CCN(CC4)C(=O)C5CC5)C(=O)O | Olaparib 1265 |
| OCP(=O)(O)COC(=O)c1cccc2C(=NNC(=O)c12)Cc3ccc(F)c(c3)C(=O)N4CCN(CC4)C(=O)C5CC5 | Olaparib 604 |
| O[C@H](CC(=O)Oc1cccc2C(=NNC(=O)c12)Cc3ccc(F)c(c3)C(=O)N4CCN(CC4)C(=O)C5CC5)C(=O)O | Olaparib 1262 |
| OP(=O)(O)COC(=O)c1cccc2C(=NNC(=O)c12)Cc3ccc(F)c(c3)C(=O)N4CCN(CC4)C(=O)C5CC5 | Olaparib 645 |
| N[C@@H](CSC(F)(F)Oc1cccc2C(=NNC(=O)c12)Cc3ccc(F)c(c3)C(=O)N4CCN(CC4)C(=O)C5CC5)C(=O)O | Olaparib 2111 |
| OC(=O)\C=C\C=C\C(=O)Nc1cccc2C(=NNC(=O)c12)Cc3ccc(F)c(c3)C(=O)N4CCN(CC4)C(=O)C5CC5 | Olaparib 320 |
| OC(=O)\C=C/C=C/C(=O)Nc1cccc2C(=NNC(=O)c12)Cc3ccc(F)c(c3)C(=O)N4CCN(CC4)C(=O)C5CC5 | Olaparib 321 |
| OC(=O)\C=C\C=C\C(=O)Oc1cccc2C(=NNC(=O)c12)Cc3ccc(F)c(c3)C(=O)N4CCN(CC4)C(=O)C5CC5 | Olaparib 1244 |
| OC(=O)\C=C/C=C/C(=O)Oc1cccc2C(=NNC(=O)c12)Cc3ccc(F)c(c3)C(=O)N4CCN(CC4)C(=O)C5CC5 | Olaparib 1245 |
| CC(=C)C(=O)NCCOC(=O)c1cccc2C(=NNC(=O)c12)Cc3ccc(F)c(c3)C(=O)N4CCN(CC4)C(=O)C5CC5 | Olaparib 598 |
| C\C(=C/C(=O)O)\CC(=O)Oc1cccc2C(=NNC(=O)c12)Cc3ccc(F)c(c3)C(=O)N4CCN(CC4)C(=O)C5CC5 | Olaparib 1233 |
| Fc1ccc(CC2=NNC(=O)c3c(cccc23)C(=O)OCCNC(=O)C=C)cc1C(=O)N4CCN(CC4)C(=O)C5CC5 | Olaparib 643 |
| C\C(=C/C(=O)Oc1cccc2C(=NNC(=O)c12)Cc3ccc(F)c(c3)C(=O)N4CCN(CC4)C(=O)C5CC5)\CC(=O)O | Olaparib 1234 |
| Fc1ccc(CC2=NNC(=O)c3c(cccc23)C(=O)OCNC(=O)C=C)cc1C(=O)N4CCN(CC4)C(=O)C5CC5 | Olaparib 682 |
| NC(=O)\C=C\C(=O)Oc1cccc2C(=NNC(=O)c12)Cc3ccc(F)c(c3)C(=O)N4CCN(CC4)C(=O)C5CC5 | Olaparib 1352 |
| OC(=O)\C=C\C(=O)Oc1cccc2C(=NNC(=O)c12)Cc3ccc(F)c(c3)C(=O)N4CCN(CC4)C(=O)C5CC5 | Olaparib 1349 |
| [O-]C(=O)\C=C\C(=O)Oc1cccc2C(=NNC(=O)c12)Cc3ccc(F)c(c3)C(=O)N4CCN(CC4)C(=O)C5CC5 | Olaparib 1353 |
| CC(C)(C)C(=O)NC(C)(C)COC(=O)c1cccc2C(=NNC(=O)c12)Cc3ccc(F)c(c3)C(=O)N4CCN(CC4)C(=O)C5CC5 | Olaparib 563 |
| CC(C)N(C(C)C)C(=O)COC(=O)c1cccc2C(=NNC(=O)c12)Cc3ccc(F)c(c3)C(=O)N4CCN(CC4)C(=O)C5CC5 | Olaparib 566 |
| CC(=O)[C@H](O)C(C)(C)OC(=O)c1cccc2C(=NNC(=O)c12)Cc3ccc(F)c(c3)C(=O)N4CCN(CC4)C(=O)C5CC5 | Olaparib 590 |
| CC(=O)NC(C)(C)COC(=O)c1cccc2C(=NNC(=O)c12)Cc3ccc(F)c(c3)C(=O)N4CCN(CC4)C(=O)C5CC5 | Olaparib 595 |
| CC(C)NC(=O)[C@H](C)OC(=O)c1cccc2C(=NNC(=O)c12)Cc3ccc(F)c(c3)C(=O)N4CCN(CC4)C(=O)C5CC5 | Olaparib 594 |
| CC(C)(CCC(=O)O)NC(=O)c1cccc2C(=NNC(=O)c12)Cc3ccc(F)c(c3)C(=O)N4CCN(CC4)C(=O)C5CC5 | Olaparib 29 |
| CC(=O)[C@H](OC(=O)c1cccc2C(=NNC(=O)c12)Cc3ccc(F)c(c3)C(=O)N4CCN(CC4)C(=O)C5CC5)C(C)(C)O | Olaparib 589 |
| CC[C@@H](COC(=O)c1cccc2C(=NNC(=O)c12)Cc3ccc(F)c(c3)C(=O)N4CCN(CC4)C(=O)C5CC5)NC(=O)C | Olaparib 596 |
| CC(=O)NC(C)(C)C(=O)Oc1cccc2C(=NNC(=O)c12)Cc3ccc(F)c(c3)C(=O)N4CCN(CC4)C(=O)C5CC5 | Olaparib 1231 |
| CN(C)C(=O)CCC(=O)Nc1cccc2C(=NNC(=O)c12)Cc3ccc(F)c(c3)C(=O)N4CCN(CC4)C(=O)C5CC5 | Olaparib 308 |
| CC(C)(C)NC(=O)C(=O)Oc1cccc2C(=NNC(=O)c12)Cc3ccc(F)c(c3)C(=O)N4CCN(CC4)C(=O)C5CC5 | Olaparib 1229 |
| NC[C@@H](OC(=O)c1cccc2C(=NNC(=O)c12)Cc3ccc(F)c(c3)C(=O)N4CCN(CC4)C(=O)C5CC5)[C@H](O)C=C | Olaparib 638 |
| C[C@@H](CNC(=O)C)OC(=O)c1cccc2C(=NNC(=O)c12)Cc3ccc(F)c(c3)C(=O)N4CCN(CC4)C(=O)C5CC5 | Olaparib 634 |
| CNC(=O)CCCOC(=O)c1cccc2C(=NNC(=O)c12)Cc3ccc(F)c(c3)C(=O)N4CCN(CC4)C(=O)C5CC5 | Olaparib 636 |
| CC(=O)NCCCOC(=O)c1cccc2C(=NNC(=O)c12)Cc3ccc(F)c(c3)C(=O)N4CCN(CC4)C(=O)C5CC5 | Olaparib 635 |
| OC[C@@H](OC(=O)c1cccc2C(=NNC(=O)c12)Cc3ccc(F)c(c3)C(=O)N4CCN(CC4)C(=O)C5CC5)[C@H](O)C=C | Olaparib 628 |
| O[C@@H](COC(=O)c1cccc2C(=NNC(=O)c12)Cc3ccc(F)c(c3)C(=O)N4CCN(CC4)C(=O)C5CC5)[C@H](O)C=C | Olaparib 630 |
| CC(C)(OC(=O)c1cccc2C(=NNC(=O)c12)Cc3ccc(F)c(c3)C(=O)N4CCN(CC4)C(=O)C5CC5)C(=O)N | Olaparib 676 |
| C[C@@H](CC(=O)N)OC(=O)c1cccc2C(=NNC(=O)c12)Cc3ccc(F)c(c3)C(=O)N4CCN(CC4)C(=O)C5CC5 | Olaparib 680 |
| NC(=O)CCCOC(=O)c1cccc2C(=NNC(=O)c12)Cc3ccc(F)c(c3)C(=O)N4CCN(CC4)C(=O)C5CC5 | Olaparib 678 |
| CC(=O)NCCC(=O)Oc1cccc2C(=NNC(=O)c12)Cc3ccc(F)c(c3)C(=O)N4CCN(CC4)C(=O)C5CC5 | Olaparib 1285 |
| Fc1ccc(CC2=NNC(=O)c3c(cccc23)C(=O)OCCCNC=O)cc1C(=O)N4CCN(CC4)C(=O)C5CC5 | Olaparib 677 |
| C[C@@H](NC=O)C(=O)Nc1cccc2C(=NNC(=O)c12)Cc3ccc(F)c(c3)C(=O)N4CCN(CC4)C(=O)C5CC5 | Olaparib 402 |
| Fc1ccc(CC2=NNC(=O)c3c(cccc23)C(=O)OCCNC(=O)C(F)(F)F)cc1C(=O)N4CCN(CC4)C(=O)C5CC5 | Olaparib 567 |
| OCCC(=O)COC(=O)c1cccc2C(=NNC(=O)c12)Cc3ccc(F)c(c3)C(=O)N4CCN(CC4)C(=O)C5CC5 | Olaparib 672 |
| NC(=O)CCC(=O)Oc1cccc2C(=NNC(=O)c12)Cc3ccc(F)c(c3)C(=O)N4CCN(CC4)C(=O)C5CC5 | Olaparib 1339 |
| C[C@@H](NC=O)C(=O)Oc1cccc2C(=NNC(=O)c12)Cc3ccc(F)c(c3)C(=O)N4CCN(CC4)C(=O)C5CC5 | Olaparib 1340 |
| Fc1ccc(CC2=NNC(=O)c3c(NC(=O)CCNC=O)cccc23)cc1C(=O)N4CCN(CC4)C(=O)C5CC5 | Olaparib 401 |
| [O-]C(=O)CCC(=O)Nc1cccc2C(=NNC(=O)c12)Cc3ccc(F)c(c3)C(=O)N4CCN(CC4)C(=O)C5CC5 | Olaparib 403 |
| OC(=O)CCC(=O)Oc1cccc2C(=NNC(=O)c12)Cc3ccc(F)c(c3)C(=O)N4CCN(CC4)C(=O)C5CC5 | Olaparib 1332 |
| Fc1ccc(CC2=NNC(=O)c3c(OC(=O)CCNC=O)cccc23)cc1C(=O)N4CCN(CC4)C(=O)C5CC5 | Olaparib 1338 |
| [O-]C(=O)CCC(=O)Oc1cccc2C(=NNC(=O)c12)Cc3ccc(F)c(c3)C(=O)N4CCN(CC4)C(=O)C5CC5 | Olaparib 1341 |
| O\N=C(\C(=N\O)\Cl)/c1cccc2C(=NNC(=O)c12)Cc3ccc(F)c(c3)C(=O)N4CCN(CC4)C(=O)C5CC5 | Olaparib 1622 |
| C[N+](C)(C)C[C@@H](CO)OC(=O)c1cccc2C(=NNC(=O)c12)Cc3ccc(F)c(c3)C(=O)N4CCN(CC4)C(=O)C5CC5 | Olaparib 575 |
| C[N+](C)(C)C[C@H](O)COC(=O)c1cccc2C(=NNC(=O)c12)Cc3ccc(F)c(c3)C(=O)N4CCN(CC4)C(=O)C5CC5 | Olaparib 576 |
| CC(C)(O)[C@H](N)C(=O)Nc1cccc2C(=NNC(=O)c12)Cc3ccc(F)c(c3)C(=O)N4CCN(CC4)C(=O)C5CC5 | Olaparib 336 |
| C[C@H](C[C@H](N)C(=O)O)C(F)(F)Oc1cccc2C(=NNC(=O)c12)Cc3ccc(F)c(c3)C(=O)N4CCN(CC4)C(=O)C5CC5 | Olaparib 2116 |
| CC(C)(COc1cccc2C(=NNC(=O)c12)Cc3ccc(F)c(c3)C(=O)N4CCN(CC4)C(=O)C5CC5)NC(=O)N | Olaparib 2768 |
| CC(CO)(CO)COC(=O)c1cccc2C(=NNC(=O)c12)Cc3ccc(F)c(c3)C(=O)N4CCN(CC4)C(=O)C5CC5 | Olaparib 610 |
| CC(C)(O)[C@H](N)C(=O)Oc1cccc2C(=NNC(=O)c12)Cc3ccc(F)c(c3)C(=O)N4CCN(CC4)C(=O)C5CC5 | Olaparib 1263 |
| COC(OC)[C@H](C)OC(=O)c1cccc2C(=NNC(=O)c12)Cc3ccc(F)c(c3)C(=O)N4CCN(CC4)C(=O)C5CC5 | Olaparib 609 |
| C[C@@H](OC(=O)c1cccc2C(=NNC(=O)c12)Cc3ccc(F)c(c3)C(=O)N4CCN(CC4)C(=O)C5CC5)[C@H](N)CN | Olaparib 666 |
| C[C@@](N)(CO)COC(=O)c1cccc2C(=NNC(=O)c12)Cc3ccc(F)c(c3)C(=O)N4CCN(CC4)C(=O)C5CC5 | Olaparib 665 |
| C[C@@H](O)[C@H](N)COC(=O)c1cccc2C(=NNC(=O)c12)Cc3ccc(F)c(c3)C(=O)N4CCN(CC4)C(=O)C5CC5 | Olaparib 664 |
| C[C@H](OC(=O)c1cccc2C(=NNC(=O)c12)Cc3ccc(F)c(c3)C(=O)N4CCN(CC4)C(=O)C5CC5)[C@H](N)CO | Olaparib 663 |
| C[C@H](OC(=O)c1cccc2C(=NNC(=O)c12)Cc3ccc(F)c(c3)C(=O)N4CCN(CC4)C(=O)C5CC5)[C@@H](O)CO | Olaparib 659 |
| C[C@@H](O)[C@H](O)COC(=O)c1cccc2C(=NNC(=O)c12)Cc3ccc(F)c(c3)C(=O)N4CCN(CC4)C(=O)C5CC5 | Olaparib 660 |
| CNC[C@@H](CO)OC(=O)c1cccc2C(=NNC(=O)c12)Cc3ccc(F)c(c3)C(=O)N4CCN(CC4)C(=O)C5CC5 | Olaparib 661 |
| CNC[C@H](O)COC(=O)c1cccc2C(=NNC(=O)c12)Cc3ccc(F)c(c3)C(=O)N4CCN(CC4)C(=O)C5CC5 | Olaparib 662 |
| CC(=O)[C@@H](O)[C@H](O)COc1cccc2C(=NNC(=O)c12)Cc3ccc(F)c(c3)C(=O)N4CCN(CC4)C(=O)C5CC5 | Olaparib 2764 |
| OC[C@@H](O)CCOC(=O)c1cccc2C(=NNC(=O)c12)Cc3ccc(F)c(c3)C(=O)N4CCN(CC4)C(=O)C5CC5 | Olaparib 652 |
| NC[C@H](N)CC(=O)Oc1cccc2C(=NNC(=O)c12)Cc3ccc(F)c(c3)C(=O)N4CCN(CC4)C(=O)C5CC5 | Olaparib 1326 |
| OCC[C@@H](CO)OC(=O)c1cccc2C(=NNC(=O)c12)Cc3ccc(F)c(c3)C(=O)N4CCN(CC4)C(=O)C5CC5 | Olaparib 650 |
| OCC(CO)COC(=O)c1cccc2C(=NNC(=O)c12)Cc3ccc(F)c(c3)C(=O)N4CCN(CC4)C(=O)C5CC5 | Olaparib 649 |
| OCC[C@H](O)COC(=O)c1cccc2C(=NNC(=O)c12)Cc3ccc(F)c(c3)C(=O)N4CCN(CC4)C(=O)C5CC5 | Olaparib 651 |
| OC[C@H](O)CCOC(=O)c1cccc2C(=NNC(=O)c12)Cc3ccc(F)c(c3)C(=O)N4CCN(CC4)C(=O)C5CC5 | Olaparib 656 |
| CO[C@@H](CO)COC(=O)c1cccc2C(=NNC(=O)c12)Cc3ccc(F)c(c3)C(=O)N4CCN(CC4)C(=O)C5CC5 | Olaparib 657 |
| OCC[C@H](O)COC(=O)c1cccc2C(=NNC(=O)c12)Cc3ccc(F)c(c3)C(=O)N4CCN(CC4)C(=O)C5CC5 | Olaparib 655 |
| COC[C@H](O)COC(=O)c1cccc2C(=NNC(=O)c12)Cc3ccc(F)c(c3)C(=O)N4CCN(CC4)C(=O)C5CC5 | Olaparib 654 |
| N[C@@H](CC(F)(F)Oc1cccc2C(=NNC(=O)c12)Cc3ccc(F)c(c3)C(=O)N4CCN(CC4)C(=O)C5CC5)C(=O)O | Olaparib 2144 |
| OCP(CO)COC(=O)c1cccc2C(=NNC(=O)c12)Cc3ccc(F)c(c3)C(=O)N4CCN(CC4)C(=O)C5CC5 | Olaparib 605 |
| OC[C@H](O)CC(=O)Nc1cccc2C(=NNC(=O)c12)Cc3ccc(F)c(c3)C(=O)N4CCN(CC4)C(=O)C5CC5 | Olaparib 385 |
| OC[C@@H](Oc1cccc2C(=NNC(=O)c12)Cc3ccc(F)c(c3)C(=O)N4CCN(CC4)C(=O)C5CC5)[C@@H](O)C=O | Olaparib 2789 |
| OC[C@H](O)C(=O)COc1cccc2C(=NNC(=O)c12)Cc3ccc(F)c(c3)C(=O)N4CCN(CC4)C(=O)C5CC5 | Olaparib 2788 |
| OC[C@H](O)CC(=O)Oc1cccc2C(=NNC(=O)c12)Cc3ccc(F)c(c3)C(=O)N4CCN(CC4)C(=O)C5CC5 | Olaparib 1320 |
| OCCNC(=O)COc1cccc2C(=NNC(=O)c12)Cc3ccc(F)c(c3)C(=O)N4CCN(CC4)C(=O)C5CC5 | Olaparib 2793 |
| NC[C@@H](Oc1cccc2C(=NNC(=O)c12)Cc3ccc(F)c(c3)C(=O)N4CCN(CC4)C(=O)C5CC5)C(=O)O | Olaparib 2166 |
| CC(C)(C)[C@H](CC#C)Oc1cccc2C(=NNC(=O)c12)Cc3ccc(F)c(c3)C(=O)N4CCN(CC4)C(=O)C5CC5 | Olaparib 2776 |
| C[C@@H](Cc1cccc2C(=NNC(=O)c12)Cc3ccc(F)c(c3)C(=O)N4CCN(CC4)C(=O)C5CC5)CC(C)(C)C | Olaparib 1815 |
| CCCCCCCc1cccc2C(=NNC(=O)c12)Cc3ccc(F)c(c3)C(=O)N4CCN(CC4)C(=O)C5CC5 | Olaparib 1919 |
| Fc1ccc(CC2=NNC(=O)c3ccccc23)cc1C(=O)N4CCN(CC4)c5cnc6nonc6c5 | Olaparib 268 |
| FC(F)Cn1cccc1N2CCN(CC2)C(=O)c3cc(CC4=NNC(=O)c5ccccc45)ccc3F | Olaparib 209 |
| CCOc1nc(Cl)nc(n1)N2CCN(CC2)C(=O)c3cc(CC4=NNC(=O)c5ccccc45)ccc3F | Olaparib 88 |
| Fc1ccc(CC2=NNC(=O)c3ccccc23)cc1C(=O)N4CCN(CC4)c5cc6nn[nH]c6cc5Cl | Olaparib 121 |
| CSc1cccc(c1)N2CCN(CC2)C(=O)c3cc(CC4=NNC(=O)c5ccccc45)ccc3F | Olaparib 163 |
| Fc1ccc(CC2=NNC(=O)c3ccccc23)cc1C(=O)N4CCN(CC4)c5cccc6nncn56 | Olaparib 122 |
| COC(=O)C(F)(F)ON1CCN(CC1)C(=O)c2cc(CC3=NNC(=O)c4ccccc34)ccc2F | Olaparib 280 |
| Fc1ccc(CC2=NNC(=O)c3ccccc23)cc1C(=O)N4CCN(CC4)[C@]5(F)CC(Cl)(Cl)[C@]5(F)Cl | Olaparib 82 |
| Fc1ccc(CC2=NNC(=O)c3ccccc23)cc1C(=O)N4CCN(CC4)c5cc6[nH]nnc6cc5Cl | Olaparib 120 |
| CN1N=CC(=C(N2CCN(CC2)C(=O)c3cc(CC4=NNC(=O)c5ccccc45)ccc3F)C1=O)N | Olaparib 101 |
| Fc1ccc(CC2=NNC(=O)c3ccccc23)cc1C(=O)N4CCN(CC4)O[C@]5(F)C(F)(F)CC5(Cl)Cl | Olaparib 286 |
| Fc1ccc(CC2=NNC(=O)c3ccccc23)cc1C(=O)N4CCN(CC4)c5cccc6cn[nH]c56 | Olaparib 124 |
| Fc1ccc(CC2=NNC(=O)c3ccccc23)cc1C(=O)N4CCN(CC4)c5ccc6nonc6c5 | Olaparib 269 |
| Fc1ccc(CC2=NNC(=O)c3ccccc23)cc1C(=O)N4CCN(CC4)c5cncc6ncccc56 | Olaparib 267 |
| COC(=O)\C(=C(/F)\N1CCN(CC1)C(=O)c2cc(CC3=NNC(=O)c4ccccc34)ccc2F)\F | Olaparib 69 |
| Fc1ccc(CC2=NNC(=O)c3ccccc23)cc1C(=O)N4CCN(CC4)O[C@]5(Cl)CC(F)(F)[C@@]5(F)Cl | Olaparib 287 |
| OC(=O)c1ccc(Cl)c(c1)N2CCN(CC2)C(=O)c3cc(CC4=NNC(=O)c5ccccc45)ccc3F | Olaparib 91 |
| Fc1ccc(CC2=NNC(=O)c3ccccc23)cc1C(=O)N4CCN(CC4)c5cccc6[nH]ncc56 | Olaparib 123 |
| FC1=CC(=CNC1=O)N2CCN(CC2)C(=O)c3cc(CC4=NNC(=O)c5ccccc45)ccc3F | Olaparib 224 |
| CC1=CNC(=O)C=C1N2CCN(CC2)C(=O)c3cc(CC4=NNC(=O)c5ccccc45)ccc3F | Olaparib 231 |
| Fc1ccc(CC2=NNC(=O)c3ccccc23)cc1C(=O)N4CCN(CC4)c5cccc6ccnn56 | Olaparib 272 |
| COC(=O)CCN1CCN(CC1)C(=O)c2cc(CC3=NNC(=O)c4ccccc34)ccc2F | Olaparib 138 |
| Fc1cc(Br)cc(c1)N2CCN(CC2)C(=O)c3cc(CC4=NNC(=O)c5ccccc45)ccc3F | Olaparib 89 |
| Fc1ccc(CC2=NNC(=O)c3ccccc23)cc1C(=O)N4CCN(CC4)OC(=O)[C@@H]5C[C@@H]5C(F)(F)F | Olaparib 44 |
| Fc1ccc(CC2=NNC(=O)c3ccccc23)cc1C(=O)N4CCN(CC4)c5ccc6oncc6c5 | Olaparib 270 |
| Cc1c(Cl)cncc1N2CCN(CC2)C(=O)c3cc(CC4=NNC(=O)c5ccccc45)ccc3F | Olaparib 214 |
| Fc1ccc(CC2=NNC(=O)c3ccccc23)cc1C(=O)N4CCN(CC4)c5ccc6OCOc6c5 | Olaparib 154 |
| Fc1ccc(CC2=NNC(=O)c3ccccc23)cc1C(=O)N4CCN(CC4)OC(F)(F)C(F)(F)I | Olaparib 273 |
| Fc1ccc(CC2=NNC(=O)c3ccccc23)cc1C(=O)N4CCN(CC4)[C@]5(Cl)CCC5(F)F | Olaparib 112 |
| Oc1nc(ON2CCN(CC2)C(=O)c3cc(CC4=NNC(=O)c5ccccc45)ccc3F)c(Br)s1 | Olaparib 299 |
| Cc1cscc1N2CCN(CC2)C(=O)c3cc(CC4=NNC(=O)c5ccccc45)ccc3F | Olaparib 235 |
| COc1cc(ncn1)N2CCN(CC2)C(=O)c3cc(CC4=NNC(=O)c5ccccc45)ccc3F | Olaparib 109 |
| Fc1ccc(CC2=NNC(=O)c3ccccc23)cc1C(=O)N4CCN(CC4)[C@@]5(Cl)CC(F)(F)[C@@]5(F)Cl | Olaparib 83 |
| Fc1ccc(CC2=NNC(=O)c3ccccc23)cc1C(=O)N4CCN(CC4)c5cccc6ncccc56 | Olaparib 119 |
| Fc1ccc(CC2=NNC(=O)c3ccccc23)cc1C(=O)N4CCN(CC4)c5cccc(c5)C6CC6 | Olaparib 271 |
| Fc1ccc(CC2=NNC(=O)c3ccccc23)cc1C(=O)N4CCN(CC4)OC(F)(F)C(F)(F)F | Olaparib 275 |
| COC(=O)C(=O)NN1CCN(CC1)C(=O)c2cc(CC3=NNC(=O)c4ccccc34)ccc2F | Olaparib 23 |
| CN1C(=O)C(=O)S[C@@]1(Cl)N2CCN(CC2)C(=O)c3cc(CC4=NNC(=O)c5ccccc45)ccc3F | Olaparib 86 |
| Cc1ccc(F)c(c1)N2CCN(CC2)C(=O)c3cc(CC4=NNC(=O)c5ccccc45)ccc3F | Olaparib 227 |
| CCOc1cc(ccn1)N2CCN(CC2)C(=O)c3cc(CC4=NNC(=O)c5ccccc45)ccc3F | Olaparib 216 |
| COC(=O)\C(=N\O)\ON1CCN(CC1)C(=O)c2cc(CC3=NNC(=O)c4ccccc34)ccc2F | Olaparib 281 |
| Fc1ccc(CC2=NNC(=O)c3ccccc23)cc1C(=O)N4CCN(CC4)[C@H]5C[C@@H]6C[C@H]5C=C6 | Olaparib 155 |
| Fc1ccc(CC2=NNC(=O)c3ccccc23)cc1C(=O)N4CCN(CC4)C(F)(F)C(F)(F)I | Olaparib 48 |
| C[C@H](C=C)C(=O)NN1CCN(CC1)C(=O)c2cc(CC3=NNC(=O)c4ccccc34)ccc2F | Olaparib 32 |
| O[C@@H]1NC(=O)NC(=O)[C@H]1N2CCN(CC2)C(=O)c3cc(CC4=NNC(=O)c5ccccc45)ccc3F | Olaparib 106 |
| CNS(=O)(=O)C(F)(F)N1CCN(CC1)C(=O)c2cc(CC3=NNC(=O)c4ccccc34)ccc2F | Olaparib 60 |
| Cc1cc(ncn1)N2CCN(CC2)C(=O)c3cc(CC4=NNC(=O)c5ccccc45)ccc3F | Olaparib 248 |
| NC1=CC(=NNC1=O)N2CCN(CC2)C(=O)c3cc(CC4=NNC(=O)c5ccccc45)ccc3F | Olaparib 108 |
| OC(=O)c1cc(F)cc(c1)N2CCN(CC2)C(=O)c3cc(CC4=NNC(=O)c5ccccc45)ccc3F | Olaparib 102 |
| Fc1ccc(CC2=NNC(=O)c3ccccc23)cc1C(=O)N4CCN(CC4)OC(=O)c5ncc[nH]5 | Olaparib 45 |
| C[C@@](N)(C(=O)O)C(F)(F)N1CCN(CC1)C(=O)c2cc(CC3=NNC(=O)c4ccccc34)ccc2F | Olaparib 62 |
| CC(C)(C#N)C(=O)NN1CCN(CC1)C(=O)c2cc(CC3=NNC(=O)c4ccccc34)ccc2F | Olaparib 18 |
| CC1=C(N2CCN(CC2)C(=O)c3cc(CC4=NNC(=O)c5ccccc45)ccc3F)C(=O)NC(=O)N1 | Olaparib 84 |
| F[C@H](CC(F)(F)F)N1CCN(CC1)C(=O)c2cc(CC3=NNC(=O)c4ccccc34)ccc2F | Olaparib 75 |
| Fc1ccc(CC2=NNC(=O)c3ccccc23)cc1C(=O)N4CCN(CC4)C5(Cl)C(=O)NC(=O)NC5=O | Olaparib 87 |
| Fc1ccc(CC2=NNC(=O)c3ccccc23)cc1C(=O)N4CCN(CC4)O[C@](F)(I)C(F)(F)F | Olaparib 274 |
| OC(=O)C1=CC(=CNC1=O)N2CCN(CC2)C(=O)c3cc(CC4=NNC(=O)c5ccccc45)ccc3F | Olaparib 98 |
| [O-]S(=O)(=O)[C@@](F)(N1CCN(CC1)C(=O)c2cc(CC3=NNC(=O)c4ccccc34)ccc2F)C(F)(F)F | Olaparib 52 |
| Nc1cnc(Cl)c(c1)N2CCN(CC2)C(=O)c3cc(CC4=NNC(=O)c5ccccc45)ccc3F | Olaparib 213 |
| C\C=C(/C)\C(=O)NN1CCN(CC1)C(=O)c2cc(CC3=NNC(=O)c4ccccc34)ccc2F | Olaparib 31 |
| Fc1ccc(CC2=NNC(=O)c3ccccc23)cc1C(=O)N4CCN(CC4)C5=CC(=O)NC(=O)N5 | Olaparib 81 |
| COc1cc(OC)cc(c1)N2CCN(CC2)C(=O)c3cc(CC4=NNC(=O)c5ccccc45)ccc3F | Olaparib 161 |
| Fc1ccc(CC2=NNC(=O)c3ccccc23)cc1C(=O)N4CCN(CC4)C5=CNC(=O)NC5=S | Olaparib 107 |
| Fc1ccc(CC2=NNC(=O)c3ccccc23)cc1C(=O)N4CCN(CC4)C5=CNC(=O)NC5=O | Olaparib 226 |
| FC(=C(F)C(=O)NN1CCN(CC1)C(=O)c2cc(CC3=NNC(=O)c4ccccc34)ccc2F)F | Olaparib 13 |
| Fc1ccc(CC2=NNC(=O)c3ccccc23)cc1C(=O)N4CCN(CC4)C5=NSN=C(Cl)C5=O | Olaparib 92 |
| Oc1c(Cl)nccc1N2CCN(CC2)C(=O)c3cc(CC4=NNC(=O)c5ccccc45)ccc3F | Olaparib 211 |
| OC(=O)c1cncc(c1)N2CCN(CC2)C(=O)c3cc(CC4=NNC(=O)c5ccccc45)ccc3F | Olaparib 217 |
| Fc1ccc(CC2=NNC(=O)c3ccccc23)cc1C(=O)N4CCN(CC4)[C@@H]5CNC(=O)NC5=O | Olaparib 223 |
| CC(C)(C)C(=O)NN1CCN(CC1)C(=O)c2cc(CC3=NNC(=O)c4ccccc34)ccc2F | Olaparib 27 |
| Fc1ccc(CC2=NNC(=O)c3ccccc23)cc1C(=O)N4CCN(CC4)OC5(Cl)CS(=O)(=O)C5 | Olaparib 288 |
| Oc1cncc(Cl)c1N2CCN(CC2)C(=O)c3cc(CC4=NNC(=O)c5ccccc45)ccc3F | Olaparib 212 |
| [O-][n+]1ccnc(c1)N2CCN(CC2)C(=O)c3cc(CC4=NNC(=O)c5ccccc45)ccc3F | Olaparib 115 |
| Fc1ccc(CC2=NNC(=O)c3ccccc23)cc1C(=O)N4CCN(CC4)OCc5cnccn5 | Olaparib 302 |
| COc1nccnc1N2CCN(CC2)C(=O)c3cc(CC4=NNC(=O)c5ccccc45)ccc3F | Olaparib 184 |
| COc1ncc(cc1O)N2CCN(CC2)C(=O)c3cc(CC4=NNC(=O)c5ccccc45)ccc3F | Olaparib 215 |
| COC(=O)C(F)(F)N1CCN(CC1)C(=O)c2cc(CC3=NNC(=O)c4ccccc34)ccc2F | Olaparib 66 |
| O[C@@H]1C=CO[C@H](CON2CCN(CC2)C(=O)c3cc(CC4=NNC(=O)c5ccccc45)ccc3F)[C@H]1O | Olaparib 300 |
| FC(F)C(F)(F)C(=O)NN1CCN(CC1)C(=O)c2cc(CC3=NNC(=O)c4ccccc34)ccc2F | Olaparib 6 |
| Cc1c(cnn1C)N2CCN(CC2)C(=O)c3cc(CC4=NNC(=O)c5ccccc45)ccc3F | Olaparib 240 |
| [O-][n+]1ccncc1N2CCN(CC2)C(=O)c3cc(CC4=NNC(=O)c5ccccc45)ccc3F | Olaparib 116 |
| COc1cnc(cn1)N2CCN(CC2)C(=O)c3cc(CC4=NNC(=O)c5ccccc45)ccc3F | Olaparib 110 |
| Nc1ccnc(n1)N2CCN(CC2)C(=O)c3cc(CC4=NNC(=O)c5ccccc45)ccc3F | Olaparib 117 |
| Fc1ccc(CC2=NNC(=O)c3ccccc23)cc1C(=O)N4CCN(CC4)c5ccnc(n5)C#N | Olaparib 233 |
| Fc1ccc(CC2=NNC(=O)c3ccccc23)cc1C(=O)N4CCN(CCC(F)(F)F)CC4 | Olaparib 127 |
| Fc1ccc(CC2=NNC(=O)c3ccccc23)cc1C(=O)N4CCN(CC4)NC(=O)[C@@H]5C[C@@H]5C(F)(F)F | Olaparib 33 |
| Fc1cncc(c1)N2CCN(CC2)C(=O)c3cc(CC4=NNC(=O)c5ccccc45)ccc3F | Olaparib 237 |
| Fc1ccc(CC2=NNC(=O)c3ccccc23)cc1C(=O)N4CCN(CC4)c5cnc(C=O)s5 | Olaparib 178 |
| Fc1ccc(CC2=NNC(=O)c3ccccc23)cc1C(=O)N4CCN(CC4)c5nc(Cl)n[nH]5 | Olaparib 94 |
| Fc1ccc(CC2=NNC(=O)c3ccccc23)cc1C(=O)N4CCN(CC4)C(F)(F)C(F)(F)F | Olaparib 50 |
| Fc1ccc(CC2=NNC(=O)c3ccccc23)cc1C(=O)N4CCN(CC4)c5n[nH]c(Br)n5 | Olaparib 93 |
| Fc1ccc(cc1F)N2CCN(CC2)C(=O)c3cc(CC4=NNC(=O)c5ccccc45)ccc3F | Olaparib 165 |
| Fc1ccc(CC2=NNC(=O)c3ccccc23)cc1C(=O)N4CCN(CC4)C5=CCS(=O)(=O)CC5 | Olaparib 208 |
| Fc1ccc(F)c(c1)N2CCN(CC2)C(=O)c3cc(CC4=NNC(=O)c5ccccc45)ccc3F | Olaparib 174 |
| Fc1ccc(CC2=NNC(=O)c3ccccc23)cc1C(=O)N4CCN(CC4)C5=CN=CC(=O)N5 | Olaparib 242 |
| Fc1ccc(CC2=NNC(=O)c3ccccc23)cc1C(=O)N4CCN(CC4)C5=CC(=CNC5=O)C#N | Olaparib 218 |
| Fc1ccc(CC2=NNC(=O)c3ccccc23)cc1C(=O)N4CCN(CC4)C5=CC=NNC5=O | Olaparib 243 |
| Fc1ccc(CC2=NNC(=O)c3ccccc23)cc1C(=O)N4CCN(CC4)[C@]5(F)CC[C@]5(F)Cl | Olaparib 111 |
| Fc1ccc(CC2=NNC(=O)c3ccccc23)cc1C(=O)N4CCN(CC4)C5(Cl)CS(=O)(=O)C5 | Olaparib 96 |
| Fc1ccc(CC2=NNC(=O)c3ccccc23)cc1C(=O)N4CCN(CC4)C5=C(Cl)OC(=O)O5 | Olaparib 104 |
| O[C@H]1OC(=O)C(=C1N2CCN(CC2)C(=O)c3cc(CC4=NNC(=O)c5ccccc45)ccc3F)Cl | Olaparib 99 |
| Fc1ccc(CC2=NNC(=O)c3ccccc23)cc1C(=O)N4CCN(CC4)C5=CNC(=O)N=C5 | Olaparib 97 |
| N[C@H](C(=O)O)C(F)(F)N1CCN(CC1)C(=O)c2cc(CC3=NNC(=O)c4ccccc34)ccc2F | Olaparib 67 |
| OS(=O)(=O)[C@@](F)(ON1CCN(CC1)C(=O)c2cc(CC3=NNC(=O)c4ccccc34)ccc2F)C(F)F | Olaparib 276 |
| N[C@]1(CCCC[C@H](C1)N2CCN(CC2)C(=O)c3cc(CC4=NNC(=O)c5ccccc45)ccc3F)C(=O)O | Olaparib 207 |
| NC(=O)c1cccc(c1)N2CCN(CC2)C(=O)c3cc(CC4=NNC(=O)c5ccccc45)ccc3F | Olaparib 103 |
| O[C@@H]1OC(=O)C=C1N2CCN(CC2)C(=O)c3cc(CC4=NNC(=O)c5ccccc45)ccc3F | Olaparib 114 |
| COC(=O)NCN1CCN(CC1)C(=O)c2cc(CC3=NNC(=O)c4ccccc34)ccc2F | Olaparib 137 |
| OS(=O)(=O)[C@](F)(C(F)F)N1CCN(CC1)C(=O)c2cc(CC3=NNC(=O)c4ccccc34)ccc2F | Olaparib 53 |
| COc1cncc(c1)N2CCN(CC2)C(=O)c3cc(CC4=NNC(=O)c5ccccc45)ccc3F | Olaparib 181 |
| Oc1ccc(C=O)cc1N2CCN(CC2)C(=O)c3cc(CC4=NNC(=O)c5ccccc45)ccc3F | Olaparib 85 |
| O[C@H]1OC(=O)C(=C1Cl)N2CCN(CC2)C(=O)c3cc(CC4=NNC(=O)c5ccccc45)ccc3F | Olaparib 100 |
| COC(=O)C(C)(C)N1CCN(CC1)C(=O)c2cc(CC3=NNC(=O)c4ccccc34)ccc2F | Olaparib 72 |
| OS(=O)(=O)C(F)(F)[C@@H](F)N1CCN(CC1)C(=O)c2cc(CC3=NNC(=O)c4ccccc34)ccc2F | Olaparib 54 |
| COc1cc(ccn1)N2CCN(CC2)C(=O)c3cc(CC4=NNC(=O)c5ccccc45)ccc3F | Olaparib 180 |
| Cc1cc(N2CCN(CC2)C(=O)c3cc(CC4=NNC(=O)c5ccccc45)ccc3F)c(O)cn1 | Olaparib 230 |
| O[C@H]1CNC[C@@H]1CON2CCN(CC2)C(=O)c3cc(CC4=NNC(=O)c5ccccc45)ccc3F | Olaparib 301 |
| CC(C)(CN)CON1CCN(CC1)C(=O)c2cc(CC3=NNC(=O)c4ccccc34)ccc2F | Olaparib 297 |
| [O-]S(=O)(=O)C(F)(F)C(F)(F)N1CCN(CC1)C(=O)c2cc(CC3=NNC(=O)c4ccccc34)ccc2F | Olaparib 51 |
| Fc1ccc(CC2=NNC(=O)c3ccccc23)cc1C(=O)N4CCN(CC4)c5coc(C=O)c5 | Olaparib 244 |
| Cc1nccc(N2CCN(CC2)C(=O)c3cc(CC4=NNC(=O)c5ccccc45)ccc3F)c1O | Olaparib 229 |
| N[C@H]1COC[C@@H]1ON2CCN(CC2)C(=O)c3cc(CC4=NNC(=O)c5ccccc45)ccc3F | Olaparib 304 |
| CC(=C(C)N1CCN(CC1)C(=O)c2cc(CC3=NNC(=O)c4ccccc34)ccc2F)C | Olaparib 202 |
| C\C=C(/Br)\C(=O)NN1CCN(CC1)C(=O)c2cc(CC3=NNC(=O)c4ccccc34)ccc2F | Olaparib 4 |
| Fc1ccc(CC2=NNC(=O)c3ccccc23)cc1C(=O)N4CCN(CC4)c5cncc(c5)C#N | Olaparib 234 |
| COc1cccc(n1)N2CCN(CC2)C(=O)c3cc(CC4=NNC(=O)c5ccccc45)ccc3F | Olaparib 166 |
| COc1ccc(cn1)N2CCN(CC2)C(=O)c3cc(CC4=NNC(=O)c5ccccc45)ccc3F | Olaparib 185 |
| Nc1cc(O)ccc1N2CCN(CC2)C(=O)c3cc(CC4=NNC(=O)c5ccccc45)ccc3F | Olaparib 118 |
| Fc1ccc(CC2=NNC(=O)c3ccccc23)cc1C(=O)N4CCN(CC4)\C=C\C(F)(F)F | Olaparib 190 |
| FC(=C(Cl)C(=O)NN1CCN(CC1)C(=O)c2cc(CC3=NNC(=O)c4ccccc34)ccc2F)F | Olaparib 8 |
| Fc1ccc(CC2=NNC(=O)c3ccccc23)cc1C(=O)N4CCN(CC4)c5cc(Cl)ncn5 | Olaparib 221 |
| Oc1cc(cc(c1)N2CCN(CC2)C(=O)c3cc(CC4=NNC(=O)c5ccccc45)ccc3F)C#N | Olaparib 219 |
| COc1ncccc1N2CCN(CC2)C(=O)c3cc(CC4=NNC(=O)c5ccccc45)ccc3F | Olaparib 159 |
| Nc1nc(F)ccc1N2CCN(CC2)C(=O)c3cc(CC4=NNC(=O)c5ccccc45)ccc3F | Olaparib 225 |
| FC(F)CC(F)(F)N1CCN(CC1)C(=O)c2cc(CC3=NNC(=O)c4ccccc34)ccc2F | Olaparib 74 |
| [2H]c1nc([2H])c(nc1Cl)N2CCN(CC2)C(=O)c3cc(CC4=NNC(=O)c5ccccc45)ccc3F | Olaparib 220 |
| Oc1ccc(cc1N2CCN(CC2)C(=O)c3cc(CC4=NNC(=O)c5ccccc45)ccc3F)C#N | Olaparib 105 |
| Fc1ccc(CC2=NNC(=O)c3ccccc23)cc1C(=O)N4CCN(CC4)[C@@](F)(I)C(F)(F)F | Olaparib 49 |
| CC1(CC1)C(=O)NN2CCN(CC2)C(=O)c3cc(CC4=NNC(=O)c5ccccc45)ccc3F | Olaparib 34 |
| COC(=O)[C@@]1(C)C[C@H]1N2CCN(CC2)C(=O)c3cc(CC4=NNC(=O)c5ccccc45)ccc3F | Olaparib 222 |
| Oc1ncccc1N2CCN(CC2)C(=O)c3cc(CC4=NNC(=O)c5ccccc45)ccc3F | Olaparib 245 |
| Fc1ccc(CC2=NNC(=O)c3ccccc23)cc1C(=O)N4CCN(CC4)NC(=O)C5CCC5 | Olaparib 35 |
| Fc1ccc(CC2=NNC(=O)c3ccccc23)cc1C(=O)N4CCN(CC4)c5ncccn5 | Olaparib 148 |
| Cc1nccc(N2CCN(CC2)C(=O)c3cc(CC4=NNC(=O)c5ccccc45)ccc3F)c1C | Olaparib 232 |
| Fc1ccc(CC2=NNC(=O)c3ccccc23)cc1C(=O)N4CCN(CC4)c5cnccn5 | Olaparib 149 |
| Fc1ccc(CC2=NNC(=O)c3ccccc23)cc1C(=O)N4CCN(CC4)c5cccnn5 | Olaparib 150 |
| CSc1ccccc1N2CCN(CC2)C(=O)c3cc(CC4=NNC(=O)c5ccccc45)ccc3F | Olaparib 164 |
| Cc1cc(n[nH]1)N2CCN(CC2)C(=O)c3cc(CC4=NNC(=O)c5ccccc45)ccc3F | Olaparib 258 |
| COc1cccc(c1)N2CCN(CC2)C(=O)c3cc(CC4=NNC(=O)c5ccccc45)ccc3F | Olaparib 182 |
| OCc1occc1N2CCN(CC2)C(=O)c3cc(CC4=NNC(=O)c5ccccc45)ccc3F | Olaparib 236 |
| Cc1ccnc(c1)N2CCN(CC2)C(=O)c3cc(CC4=NNC(=O)c5ccccc45)ccc3F | Olaparib 249 |
| Fc1ccc(CC2=NNC(=O)c3ccccc23)cc1C(=O)N4CCN(CC4)C5=CC=CNC5=O | Olaparib 247 |
| Fc1ccc(CC2=NNC(=O)c3ccccc23)cc1C(=O)N4CCN(CC4)c5ccsc5C#N | Olaparib 228 |
| CCc1ccccc1N2CCN(CC2)C(=O)c3cc(CC4=NNC(=O)c5ccccc45)ccc3F | Olaparib 175 |
| Fc1ccc(CC2=NNC(=O)c3ccccc23)cc1C(=O)N4CCN(CC4)c5cccc(Br)n5 | Olaparib 206 |
| Fc1ccc(CC2=NNC(=O)c3ccccc23)cc1C(=O)N4CCN(CC4)c5ncccc5F | Olaparib 183 |
| Fc1ccc(CC2=NNC(=O)c3ccccc23)cc1C(=O)N4CCN(CC4)c5occn5 | Olaparib 179 |
| Fc1ccc(CC2=NNC(=O)c3ccccc23)cc1C(=O)N4CCN(CC4)C5=CCC(=O)N=C5 | Olaparib 246 |
| Cc1ccc(Cl)c(c1)N2CCN(CC2)C(=O)c3cc(CC4=NNC(=O)c5ccccc45)ccc3F | Olaparib 162 |
| Fc1ccc(CC2=NNC(=O)c3ccccc23)cc1C(=O)N4CCN(CC4)OC(=O)C5CCC5 | Olaparib 46 |
| Fc1ccc(nc1)N2CCN(CC2)C(=O)c3cc(CC4=NNC(=O)c5ccccc45)ccc3F | Olaparib 95 |
| Fc1ccc(CC2=NNC(=O)c3ccccc23)cc1C(=O)N4CCN(CC4)c5cccnc5 | Olaparib 151 |
| Fc1cccc(c1)N2CCN(CC2)C(=O)c3cc(CC4=NNC(=O)c5ccccc45)ccc3F | Olaparib 168 |
| Fc1ccc(CC2=NNC(=O)c3ccccc23)cc1C(=O)N4CCN(CC4)c5ccccn5 | Olaparib 260 |
| CC1(C)C[C@@]1(C)N2CCN(CC2)C(=O)c3cc(CC4=NNC(=O)c5ccccc45)ccc3F | Olaparib 253 |
| CN1CCC(CC1)N2CCN(CC2)C(=O)c3cc(CC4=NNC(=O)c5ccccc45)ccc3F | Olaparib 170 |
| CCc1ccoc1N2CCN(CC2)C(=O)c3cc(CC4=NNC(=O)c5ccccc45)ccc3F | Olaparib 241 |
| Fc1ccc(CC2=NNC(=O)c3ccccc23)cc1C(=O)N4CCN(CC4)c5ccncc5 | Olaparib 152 |
| N[C@H](C=C)C(=O)NN1CCN(CC1)C(=O)c2cc(CC3=NNC(=O)c4ccccc34)ccc2F | Olaparib 29 |
| OC(=O)[C@@](F)(N1CCN(CC1)C(=O)c2cc(CC3=NNC(=O)c4ccccc34)ccc2F)S(=O)(=O)O | Olaparib 56 |
| Fc1ccc(CC2=NNC(=O)c3ccccc23)cc1C(=O)N4CCN(CC4)C(F)(F)S(=O)(=O)CC#N | Olaparib 57 |
| Fc1ccc(CC2=NNC(=O)c3ccccc23)cc1C(=O)N4CCN(CC4)O[C@@]5(F)CCC5(F)F | Olaparib 291 |
| Fc1ccc(CC2=NNC(=O)c3ccccc23)cc1C(=O)N4CCN(CC4)C5=CCCNC5 | Olaparib 256 |
| Fc1ccc(CC2=NNC(=O)c3ccccc23)cc1C(=O)N4CCN(CC4)c5cccs5 | Olaparib 147 |
| CC(C)([C@H](N)C(=O)O)N1CCN(CC1)C(=O)c2cc(CC3=NNC(=O)c4ccccc34)ccc2F | Olaparib 73 |
| [15NH2][C@H](C[15NH]O)C(=O)NN1CCN(CC1)C(=O)c2cc(CC3=NNC(=O)c4ccccc34)ccc2F | Olaparib 14 |
| Fc1ccc(CC2=NNC(=O)c3ccccc23)cc1C(=O)N4CCN(CC4)O[C@@]5(F)CC[C@]5(F)Cl | Olaparib 289 |
| Fc1ccc(CC2=NNC(=O)c3ccccc23)cc1C(=O)N4CCN(CC4)[C@@H]5CCCNC5 | Olaparib 252 |
| Fc1ccc(CC2=NNC(=O)c3ccccc23)cc1C(=O)N4CCN(CC4)C5=CCCCO5 | Olaparib 254 |
| Fc1ccc(CC2=NNC(=O)c3ccccc23)cc1C(=O)N4CCN(CC4)c5c(F)cccc5Cl | Olaparib 210 |
| Fc1ccc(CC2=NNC(=O)c3ccccc23)cc1C(=O)N4CCN(CC4)C5=CCCC5=O | Olaparib 259 |
| CC[C@@H](N)C(=O)NN1CCN(CC1)C(=O)c2cc(CC3=NNC(=O)c4ccccc34)ccc2F | Olaparib 24 |
| Fc1ccc(CC2=NNC(=O)c3ccccc23)cc1C(=O)N4CCN(CC4)O[C@@]5(Cl)CCC5(F)F | Olaparib 290 |
| Fc1ccc(CC2=NNC(=O)c3ccccc23)cc1C(=O)N4CCN(CC4)[C@@]5(F)CCC5(F)F | Olaparib 113 |
| C[C@H](ON1CCN(CC1)C(=O)c2cc(CC3=NNC(=O)c4ccccc34)ccc2F)[C]5[CH][CH][CH][CH]5 | Olaparib 303 |
| Fc1ccc(CC2=NNC(=O)c3ccccc23)cc1C(=O)N4CCN(CC4)C5=CCOCC5 | Olaparib 176 |
| Fc1ccc(CC2=NNC(=O)c3ccccc23)cc1C(=O)N4CCN(CC4)C5=CCNCC5 | Olaparib 255 |
| N[C@H](CF)C(=O)NN1CCN(CC1)C(=O)c2cc(CC3=NNC(=O)c4ccccc34)ccc2F | Olaparib 19 |
| NS(=O)(=O)C(F)(F)N1CCN(CC1)C(=O)c2cc(CC3=NNC(=O)c4ccccc34)ccc2F | Olaparib 64 |
| Fc1ccc(CC2=NNC(=O)c3ccccc23)cc1C(=O)N4CCN(CC4)[C@H]5CCCOC5 | Olaparib 250 |
| Fc1ccc(CC2=NNC(=O)c3ccccc23)cc1C(=O)N4CCN(CC4)C5=CCCO5 | Olaparib 264 |
| Fc1ccc(CC2=NNC(=O)c3ccccc23)cc1C(=O)N4CCN(CC4)C5CCNCC5 | Olaparib 251 |
| CCC(=C)C(=O)NN1CCN(CC1)C(=O)c2cc(CC3=NNC(=O)c4ccccc34)ccc2F | Olaparib 30 |
| [15NH2][C@H](CO)C(=O)NN1CCN(CC1)C(=O)c2cc(CC3=NNC(=O)c4ccccc34)ccc2F | Olaparib 22 |
| Fc1ccc(CC2=NNC(=O)c3ccccc23)cc1C(=O)N4CCN(CC4)[C@@H]5CCOC5 | Olaparib 261 |
| Fc1ccc(CC2=NNC(=O)c3ccccc23)cc1C(=O)N4CCN(CC4)C5CCOCC5 | Olaparib 171 |
| Fc1ccc(CC2=NNC(=O)c3ccccc23)cc1C(=O)N4CCN(CC4)[C@H]5CCNC5 | Olaparib 262 |
| Fc1ccc(CC2=NNC(=O)c3ccccc23)cc1C(=O)N4CCN(CC4)NC(=O)C(F)(F)F | Olaparib 17 |
| Cc1cccc(c1)N2CCN(CC2)C(=O)c3cc(CC4=NNC(=O)c5ccccc45)ccc3F | Olaparib 169 |
| C[C@](N)(C=C)C(=O)NN1CCN(CC1)C(=O)c2cc(CC3=NNC(=O)c4ccccc34)ccc2F | Olaparib 15 |
| Fc1ccc(CC2=NNC(=O)c3ccccc23)cc1C(=O)N4CCN(CC4)c5ccccc5 | Olaparib 145 |
| Fc1ccc(CC2=NNC(=O)c3ccccc23)cc1C(=O)N4CCN(CC4)OC(=O)C(F)(F)F | Olaparib 42 |
| Fc1ccc(CC2=NNC(=O)c3ccccc23)cc1C(=O)N4CCN(CC4)c5c(Cl)cccc5Cl | Olaparib 160 |
| OC(=O)C(=O)C(F)(F)N1CCN(CC1)C(=O)c2cc(CC3=NNC(=O)c4ccccc34)ccc2F | Olaparib 68 |
| Fc1cc(F)cc(c1)N2CCN(CC2)C(=O)c3cc(CC4=NNC(=O)c5ccccc45)ccc3F | Olaparib 90 |
| Fc1ccc(CC2=NNC(=O)c3ccccc23)cc1C(=O)N4CCN(CC4)C5=CCCCCC5 | Olaparib 239 |
| Fc1ccc(CC2=NNC(=O)c3ccccc23)cc1C(=O)N4CCN(CC4)C(=C)C=C | Olaparib 156 |
| CC(C)(ON1CCN(CC1)C(=O)c2cc(CC3=NNC(=O)c4ccccc34)ccc2F)C(=O)N | Olaparib 298 |
| Fc1ccc(CC2=NNC(=O)c3ccccc23)cc1C(=O)N4CCN(CC4)\C=C\C5CCCC5 | Olaparib 238 |
| Fc1ccc(CC2=NNC(=O)c3ccccc23)cc1C(=O)N4CCN(CC4)C5=CCCCC5 | Olaparib 257 |
| CC[C@H](C)\C=C\N1CCN(CC1)C(=O)c2cc(CC3=NNC(=O)c4ccccc34)ccc2F | Olaparib 195 |
| N[C@H](CN1CCN(CC1)C(=O)c2cc(CC3=NNC(=O)c4ccccc34)ccc2F)C(=O)O | Olaparib 78 |
| Fc1ccc(CC2=NNC(=O)c3ccccc23)cc1C(=O)N4CCN(CC4)C5CCCCCC5 | Olaparib 167 |
| Fc1ccc(CC2=NNC(=O)c3ccccc23)cc1C(=O)N4CCN(CC4)C5=CCCC5 | Olaparib 172 |
| Fc1ccc(CC2=NNC(=O)c3ccccc23)cc1C(=O)N4CCN(CC4)C(=C)C(F)(F)F | Olaparib 189 |
| CO[C@@H](CO)CON1CCN(CC1)C(=O)c2cc(CC3=NNC(=O)c4ccccc34)ccc2F | Olaparib 295 |
| Fc1ccc(CC2=NNC(=O)c3ccccc23)cc1C(=O)N4CCN(CCC5CC5)CC4 | Olaparib 263 |
| Fc1ccc(CC2=NNC(=O)c3ccccc23)cc1C(=O)N4CCN(CC4)\C=C\C5CC5 | Olaparib 265 |
| CC(C)C(C)(C)N1CCN(CC1)C(=O)c2cc(CC3=NNC(=O)c4ccccc34)ccc2F | Olaparib 192 |
| O\N=C(/Cl)\C(=N\O)\ON1CCN(CC1)C(=O)c2cc(CC3=NNC(=O)c4ccccc34)ccc2F | Olaparib 278 |
| CC[C@@H](ON1CCN(CC1)C(=O)c2cc(CC3=NNC(=O)c4ccccc34)ccc2F)C(Cl)(Cl)Cl | Olaparib 292 |
| OS(=O)(=O)CC(F)(F)N1CCN(CC1)C(=O)c2cc(CC3=NNC(=O)c4ccccc34)ccc2F | Olaparib 59 |
| COC(=O)\C(=N\O)\N1CCN(CC1)C(=O)c2cc(CC3=NNC(=O)c4ccccc34)ccc2F | Olaparib 71 |
| CCC\C=C\N1CCN(CC1)C(=O)c2cc(CC3=NNC(=O)c4ccccc34)ccc2F | Olaparib 201 |
| Fc1ccc(CC2=NNC(=O)c3ccccc23)cc1C(=O)N4CCN(CC4)C5CCCCC5 | Olaparib 146 |
| CC(=CN1CCN(CC1)C(=O)c2cc(CC3=NNC(=O)c4ccccc34)ccc2F)C | Olaparib 157 |
| Fc1ccc(CC2=NNC(=O)c3ccccc23)cc1C(=O)N4CCN(CC4)C(=O)NC(=S)CC#N | Olaparib 1 |
| Fc1ccc(CC2=NNC(=O)c3ccccc23)cc1C(=O)N4CCN(CC4)C5CCCC5 | Olaparib 153 |
| CC(C)(C)N1CCN(CC1)C(=O)c2cc(CC3=NNC(=O)c4ccccc34)ccc2F | Olaparib 205 |
| N[C@H](CON1CCN(CC1)C(=O)c2cc(CC3=NNC(=O)c4ccccc34)ccc2F)C(=O)O | Olaparib 283 |
| Fc1ccc(CC2=NNC(=O)c3ccccc23)cc1C(=O)N4CCN(CC4)C5CCC5 | Olaparib 173 |
| Fc1ccc(CC2=NNC(=O)c3ccccc23)cc1C(=O)N4CCN(CC4)C(F)(F)F | Olaparib 188 |
| NC[C@@H](ON1CCN(CC1)C(=O)c2cc(CC3=NNC(=O)c4ccccc34)ccc2F)C(=O)O | Olaparib 284 |
| Fc1ccc(CC2=NNC(=O)c3ccccc23)cc1C(=O)N4CCN(CC4)C5CC5 | Olaparib 186 |
| Fc1ccc(CC2=NNC(=O)c3ccccc23)cc1C(=O)N4CCN(CC4)[C]5[CH][CH][CH][CH]5 | Olaparib 266 |
| OC\C=C\C(=O)NN1CCN(CC1)C(=O)c2cc(CC3=NNC(=O)c4ccccc34)ccc2F | Olaparib 28 |
| [O-]C(=O)\C=C\C(=O)NN1CCN(CC1)C(=O)c2cc(CC3=NNC(=O)c4ccccc34)ccc2F | Olaparib 16 |
| CN[C@@H](C)C(=O)NN1CCN(CC1)C(=O)c2cc(CC3=NNC(=O)c4ccccc34)ccc2F | Olaparib 25 |
| N[C@H](CC(=O)N)C(=O)NN1CCN(CC1)C(=O)c2cc(CC3=NNC(=O)c4ccccc34)ccc2F | Olaparib 10 |
| N[C@H](CC(=O)O)C(=O)NN1CCN(CC1)C(=O)c2cc(CC3=NNC(=O)c4ccccc34)ccc2F | Olaparib 9 |
| OC(=O)C(F)(N1CCN(CC1)C(=O)c2cc(CC3=NNC(=O)c4ccccc34)ccc2F)C(=O)O | Olaparib 70 |
| COC(=O)C(=C)N1CCN(CC1)C(=O)c2cc(CC3=NNC(=O)c4ccccc34)ccc2F | Olaparib 194 |
| Fc1ccc(CC2=NNC(=O)c3ccccc23)cc1C(=O)N4CCN(CC4)OC(F)(F)S(=O)(=O)CC#N | Olaparib 277 |
| OC[C@@H](C(=O)O)C(F)(F)N1CCN(CC1)C(=O)c2cc(CC3=NNC(=O)c4ccccc34)ccc2F | Olaparib 61 |
| CC(=O)\C(=N\O)\ON1CCN(CC1)C(=O)c2cc(CC3=NNC(=O)c4ccccc34)ccc2F | Olaparib 282 |
| OC(=O)C(=O)CON1CCN(CC1)C(=O)c2cc(CC3=NNC(=O)c4ccccc34)ccc2F | Olaparib 285 |
| C[C@@](F)(Cl)C(=O)NN1CCN(CC1)C(=O)c2cc(CC3=NNC(=O)c4ccccc34)ccc2F | Olaparib 12 |
| NS(=O)(=O)C(F)(F)ON1CCN(CC1)C(=O)c2cc(CC3=NNC(=O)c4ccccc34)ccc2F | Olaparib 279 |
| C\C(=C/Br)\C(=O)NN1CCN(CC1)C(=O)c2cc(CC3=NNC(=O)c4ccccc34)ccc2F | Olaparib 5 |
| OP(=O)(O)CON1CCN(CC1)C(=O)c2cc(CC3=NNC(=O)c4ccccc34)ccc2F | Olaparib 294 |
| O\N=C(/Cl)\C(=N\O)\N1CCN(CC1)C(=O)c2cc(CC3=NNC(=O)c4ccccc34)ccc2F | Olaparib 63 |
| NOC\C=C\N1CCN(CC1)C(=O)c2cc(CC3=NNC(=O)c4ccccc34)ccc2F | Olaparib 77 |
| NC(=O)C(Br)(N1CCN(CC1)C(=O)c2cc(CC3=NNC(=O)c4ccccc34)ccc2F)C(=O)N | Olaparib 47 |
| OC(=O)C(F)(F)C(=O)ON1CCN(CC1)C(=O)c2cc(CC3=NNC(=O)c4ccccc34)ccc2F | Olaparib 39 |
| OC(=O)C(=O)CN1CCN(CC1)C(=O)c2cc(CC3=NNC(=O)c4ccccc34)ccc2F | Olaparib 80 |
| OC(=O)\C(=C(/Cl)\C=O)\N1CCN(CC1)C(=O)c2cc(CC3=NNC(=O)c4ccccc34)ccc2F | Olaparib 58 |
| CC(=O)\C(=N\O)\N1CCN(CC1)C(=O)c2cc(CC3=NNC(=O)c4ccccc34)ccc2F | Olaparib 76 |
| C[C@@](F)(Cl)C(=O)ON1CCN(CC1)C(=O)c2cc(CC3=NNC(=O)c4ccccc34)ccc2F | Olaparib 41 |
| CC(C)(N)C(=O)NN1CCN(CC1)C(=O)c2cc(CC3=NNC(=O)c4ccccc34)ccc2F | Olaparib 26 |
| NC[C@@H](N1CCN(CC1)C(=O)c2cc(CC3=NNC(=O)c4ccccc34)ccc2F)C(=O)O | Olaparib 79 |
| Fc1ccc(CC2=NNC(=O)c3ccccc23)cc1C(=O)N4CCN(CC4)NC(=O)C(F)(F)Cl | Olaparib 11 |
| COC(=O)C(Cl)(Cl)N1CCN(CC1)C(=O)c2cc(CC3=NNC(=O)c4ccccc34)ccc2F | Olaparib 55 |
| Fc1ccc(CC2=NNC(=O)c3ccccc23)cc1C(=O)N4CCN(COC(=O)C=C)CC4 | Olaparib 140 |
| Fc1ccc(CC2=NNC(=O)c3ccccc23)cc1C(=O)N4CCN(CC4)OC(=O)C(F)(F)Cl | Olaparib 40 |
| C\C(=C/Br)\C(=O)ON1CCN(CC1)C(=O)c2cc(CC3=NNC(=O)c4ccccc34)ccc2F | Olaparib 37 |
| COC(=O)[C@](F)(Cl)N1CCN(CC1)C(=O)c2cc(CC3=NNC(=O)c4ccccc34)ccc2F | Olaparib 65 |
| Fc1ccc(CC2=NNC(=O)c3ccccc23)cc1C(=O)N4CCN(CC4)NC(=O)C(=C)Cl | Olaparib 20 |
| CC[C@@](C)(O)CON1CCN(CC1)C(=O)c2cc(CC3=NNC(=O)c4ccccc34)ccc2F | Olaparib 296 |
| CC(C)(Br)C(=O)NN1CCN(CC1)C(=O)c2cc(CC3=NNC(=O)c4ccccc34)ccc2F | Olaparib 3 |
| Fc1ccc(CC2=NNC(=O)c3ccccc23)cc1C(=O)N4CCN(CC4)NC(=O)\C=C\Cl | Olaparib 21 |
| Fc1ccc(CC2=NNC(=O)c3ccccc23)cc1C(=O)N4CCN(CC4)OC(=O)C(=C)Cl | Olaparib 43 |
| Fc1ccc(CC2=NNC(=O)c3ccccc23)cc1C(=O)N4CCN(CCCC=O)CC4 | Olaparib 131 |
| Fc1ccc(CC2=NNC(=O)c3ccccc23)cc1C(=O)N4CCN(CC4)NC(=O)\C=C\I | Olaparib 2 |
| CC(C)(Br)C(=O)ON1CCN(CC1)C(=O)c2cc(CC3=NNC(=O)c4ccccc34)ccc2F | Olaparib 36 |
| Fc1ccc(CC2=NNC(=O)c3ccccc23)cc1C(=O)N4CCN(CC4)C(=O)C=C | Olaparib 177 |
| CC(=O)CCN1CCN(CC1)C(=O)c2cc(CC3=NNC(=O)c4ccccc34)ccc2F | Olaparib 200 |
| COC\C=C\N1CCN(CC1)C(=O)c2cc(CC3=NNC(=O)c4ccccc34)ccc2F | Olaparib 199 |
| CC(Cl)(Cl)C(=O)NN1CCN(CC1)C(=O)c2cc(CC3=NNC(=O)c4ccccc34)ccc2F | Olaparib 7 |
| CC(Cl)(Cl)C(=O)ON1CCN(CC1)C(=O)c2cc(CC3=NNC(=O)c4ccccc34)ccc2F | Olaparib 38 |
| NCC(=C)N1CCN(CC1)C(=O)c2cc(CC3=NNC(=O)c4ccccc34)ccc2F | Olaparib 187 |
| NCCCCN1CCN(CC1)C(=O)c2cc(CC3=NNC(=O)c4ccccc34)ccc2F | Olaparib 130 |
| O[C@H](ON1CCN(CC1)C(=O)c2cc(CC3=NNC(=O)c4ccccc34)ccc2F)C(Cl)(Cl)Cl | Olaparib 293 |
| CC(C)(O)CCN1CCN(CC1)C(=O)c2cc(CC3=NNC(=O)c4ccccc34)ccc2F | Olaparib 191 |
| Fc1ccc(CC2=NNC(=O)c3ccccc23)cc1C(=O)N4CCN(CCCC=C)CC4 | Olaparib 203 |
| COC[C@@H](C)N1CCN(CC1)C(=O)c2cc(CC3=NNC(=O)c4ccccc34)ccc2F | Olaparib 196 |
| C[C@@H](O)CCN1CCN(CC1)C(=O)c2cc(CC3=NNC(=O)c4ccccc34)ccc2F | Olaparib 198 |
| COCCCN1CCN(CC1)C(=O)c2cc(CC3=NNC(=O)c4ccccc34)ccc2F | Olaparib 141 |
| CC(C)CN1CCN(CC1)C(=O)c2cc(CC3=NNC(=O)c4ccccc34)ccc2F | Olaparib 134 |
| C[C@H](CCN1CCN(CC1)C(=O)c2cc(CC3=NNC(=O)c4ccccc34)ccc2F)C#N | Olaparib 129 |
| OCCCCN1CCN(CC1)C(=O)c2cc(CC3=NNC(=O)c4ccccc34)ccc2F | Olaparib 197 |
| NCCCN1CCN(CC1)C(=O)c2cc(CC3=NNC(=O)c4ccccc34)ccc2F | Olaparib 133 |
| NCCN1CCN(CC1)C(=O)c2cc(CC3=NNC(=O)c4ccccc34)ccc2F | Olaparib 139 |
| COCCN1CCN(CC1)C(=O)c2cc(CC3=NNC(=O)c4ccccc34)ccc2F | Olaparib 204 |
| Fc1ccc(CC2=NNC(=O)c3ccccc23)cc1C(=O)N4CCN(CCCC#N)CC4 | Olaparib 142 |
| Fc1ccc(CC2=NNC(=O)c3ccccc23)cc1C(=O)N4CCN(CCC=C)CC4 | Olaparib 136 |
| Fc1ccc(CC2=NNC(=O)c3ccccc23)cc1C(=O)N4CCN(CC=C)CC4 | Olaparib 144 |
| CC(C)(C)CCN1CCN(CC1)C(=O)c2cc(CC3=NNC(=O)c4ccccc34)ccc2F | Olaparib 193 |
| Fc1ccc(CC2=NNC(=O)c3ccccc23)cc1C(=O)N4CCN(CC4)C=C | Olaparib 125 |
| CC(C)CCN1CCN(CC1)C(=O)c2cc(CC3=NNC(=O)c4ccccc34)ccc2F | Olaparib 158 |
| CC[C@@H](C)N1CCN(CC1)C(=O)c2cc(CC3=NNC(=O)c4ccccc34)ccc2F | Olaparib 135 |
| CCCCN1CCN(CC1)C(=O)c2cc(CC3=NNC(=O)c4ccccc34)ccc2F | Olaparib 143 |
| CCCN1CCN(CC1)C(=O)c2cc(CC3=NNC(=O)c4ccccc34)ccc2F | Olaparib 128 |
| CCN1CCN(CC1)C(=O)c2cc(CC3=NNC(=O)c4ccccc34)ccc2F | Olaparib 132 |
| CN1CCN(CC1)C(=O)c2cc(CC3=NNC(=O)c4ccccc34)ccc2F | Olaparib 126 |
| CNCc1ccc(cc1)c2c3CCNC(=O)c4cc(F)cc(c34)n2C(=O)OCc5cccc6nonc56 | Rucaparib 172 |
| CNCc1ccc(cc1)c2c3CCNC(=O)c4cc(F)cc(c34)n2CN(C)C(=O)C(C)(C)C | Rucaparib 267 |
| CNCc1ccc(cc1)c2c3CCNC(=O)c4cc(F)cc(c34)n2Oc5ccc6CN(Cc7onc(C)n7)C(=N)c6c5 | Rucaparib 365 |
| CNCc1ccc(cc1)c2c3CCNC(=O)c4cc(F)cc(c34)n2Oc5ccc6C(=O)C=CN(Cc7nn[nH]n7)c6c5 | Rucaparib 366 |
| CNCc1ccc(cc1)c2c3CCNC(=O)c4cc(F)cc(c34)n2C(=O)OCc5cc(ccc5C6(N)CC6)C(F)(F)F | Rucaparib 160 |
| CNCc1ccc(cc1)c2c3CCNC(=O)c4cc(F)cc(c34)n2O\C(=C(/F)\C(=O)OC)\F | Rucaparib 294 |
| CNCc1ccc(cc1)c2c3CCNC(=O)c4cc(F)cc(c34)n2C(=O)OCc5cncc(c5)c6nncs6 | Rucaparib 167 |
| CNCc1ccc(cc1)c2c3CCNC(=O)c4cc(F)cc(c34)n2OC5=NNC(=O)C(=C5)N | Rucaparib 337 |
| CNCc1ccc(cc1)c2c3CCNC(=O)c4cc(F)cc(c34)n2Oc5cccc6cn[nH]c56 | Rucaparib 357 |
| CNCc1ccc(cc1)c2c3CCNC(=O)c4cc(F)cc(c34)n2Oc5ccc(cc5)C6=COc7cc(O)cc(O)c7C6=O | Rucaparib 361 |
| CNCc1ccc(cc1)c2c3CCNC(=O)c4cc(F)cc(c34)n2Oc5ccc6NC(=O)C(=O)c6c5 | Rucaparib 354 |
| CNCc1ccc(cc1)c2c3CCNC(=O)c4cc(F)cc(c34)n2OC(=O)CCC(F)(F)F | Rucaparib 179 |
| CNCc1ccc(cc1)c2c3CCNC(=O)c4cc(F)cc(c34)n2C(=O)OCc5nn([C@@H]6CCOC6)c7nccc(N)c57 | Rucaparib 173 |
| CNCc1ccc(cc1)c2c3CCNC(=O)c4cc(F)cc(c34)n2C(=O)Oc5c(F)c(F)cc(c5F)C6(N)CC6 | Rucaparib 164 |
| CNCc1ccc(cc1)c2c3CCNC(=O)c4cc(F)cc(c34)n2Oc5cc(F)cc(F)c5 | Rucaparib 315 |
| CNCc1ccc(cc1)c2c3CCNC(=O)c4cc(F)cc(c34)n2OC(=O)\C=C\C(F)(F)F | Rucaparib 180 |
| CNCc1ccc(cc1)c2c3CCNC(=O)c4cc(F)cc(c34)n2OC5=CNC(=S)NC5=O | Rucaparib 299 |
| CNCc1ccc(cc1)c2c3CCNC(=O)c4cc(F)cc(c34)n2C(=O)O[C@H](C)CNC(=O)[C@H]5COc6ccccc56 | Rucaparib 162 |
| CNCc1ccc(cc1)c2c3CCNC(=O)c4cc(F)cc(c34)n2O[C@@H]5CC[C@H](CC5)c6onc(n6)c7cnccn7 | Rucaparib 435 |
| CNCc1ccc(cc1)c2c3CCNC(=O)c4cc(F)cc(c34)n2CC[C@@H]5C[C@@H]6C[C@H]5C=C6 | Rucaparib 247 |
| CNCc1ccc(cc1)c2c3CCNC(=O)c4cc(F)cc(c34)n2Oc5ccc6sc(COC7CCNCC7)nc6c5 | Rucaparib 362 |
| CNCc1ccc(cc1)c2c3CCNC(=O)c4cc(F)cc(c34)n2Oc5cc(OC)c6ccccc6n5 | Rucaparib 352 |
| CNCc1ccc(cc1)c2c3CCNC(=O)c4cc(F)cc(c34)n2Oc5ccc6OC(=O)Nc6c5 | Rucaparib 355 |
| CNCc1ccc(cc1)c2c3CCNC(=O)c4cc(F)cc(c34)n2OC(=O)C(F)(F)C(F)F | Rucaparib 177 |
| CNCc1ccc(cc1)c2c3CCNC(=O)c4cc(F)cc(c34)n2O[C@@H]5C[C@@H]6N(C5)C(=O)[C@@H]7C[C@@H](O)CN7C6=O | Rucaparib 436 |
| CNCc1ccc(cc1)c2c3CCNC(=O)c4cc(F)cc(c34)n2C(=O)OCc5sc6cc(OC)ccc6c5Cl | Rucaparib 161 |
| CNCc1ccc(cc1)c2c3CCNC(=O)c4cc(F)cc(c34)n2Cc5ccc(F)c(Cl)c5 | Rucaparib 262 |
| CNCc1ccc(cc1)c2c3CCNC(=O)c4cc(F)cc(c34)n2OC5=C(Br)C(=O)N(C)C5=O | Rucaparib 298 |
| CNCc1ccc(cc1)c2c3CCNC(=O)c4cc(F)cc(c34)n2O[C@H](C)C(=O)N(C)C | Rucaparib 378 |
| CNCc1ccc(cc1)c2c3CCNC(=O)c4cc(F)cc(c34)n2OC5=NSN=C(Cl)C5=O | Rucaparib 319 |
| CNCc1ccc(cc1)c2c3CCNC(=O)c4cc(F)cc(c34)n2NC(=O)[C@@H](F)C(F)(F)F | Rucaparib 2 |
| CNCc1ccc(cc1)c2c3CCNC(=O)c4cc(F)cc(c34)n2Oc5ccc6nc(cn6n5)c7ccccc7 | Rucaparib 370 |
| CNCc1ccc(cc1)c2c3CCNC(=O)c4cc(F)cc(c34)n2Oc5ccc6nc(C)sc6c5 | Rucaparib 429 |
| CNCc1ccc(cc1)c2c3CCNC(=O)c4cc(F)cc(c34)n2OC5=C(O)C(=O)C(=O)C5=O | Rucaparib 407 |
| CNCc1ccc(cc1)c2c3CCNC(=O)c4cc(F)cc(c34)n2C(=O)OCC5=CNC(=O)N=C5N | Rucaparib 133 |
| CNCc1ccc(cc1)c2c3CCNC(=O)c4cc(F)cc(c34)n2OC5=C(NC(=O)NC5=O)C(=O)O | Rucaparib 329 |
| CNCc1ccc(cc1)c2c3CCNC(=O)c4cc(F)cc(c34)n2Oc5ccc(cc5)C(=O)C(=O)c6ccccc6 | Rucaparib 346 |
| CNCc1ccc(cc1)c2c3CCNC(=O)c4cc(F)cc(c34)n2Oc5nccc(n5)c6ccccc6 | Rucaparib 353 |
| CNCc1ccc(cc1)c2c3CCNC(=O)c4cc(F)cc(c34)n2CCCC(F)(F)F | Rucaparib 268 |
| CNCc1ccc(cc1)c2c3CCNC(=O)c4cc(F)cc(c34)n2Oc5ccc6oc(nc6c5)N7CCOCC7 | Rucaparib 368 |
| CNCc1ccc(cc1)c2c3CCNC(=O)c4cc(F)cc(c34)n2C(=O)OCc5cc(OC)c(OC)cc5Br | Rucaparib 72 |
| CNCc1ccc(cc1)c2c3CCNC(=O)c4cc(F)cc(c34)n2Oc5c(N)c(F)c(N)c(F)c5F | Rucaparib 323 |
| CCOCC(=O)Nn1c(c2CCNC(=O)c3cc(F)cc1c23)c4ccc(CNC)cc4 | Rucaparib 10 |
| CNCc1ccc(cc1)c2c3CCNC(=O)c4cc(F)cc(c34)n2C(=O)OCCN5c6ccccc6Oc7ccccc57 | Rucaparib 174 |
| CNCc1ccc(cc1)c2c3CCNC(=O)c4cc(F)cc(c34)n2OC(F)(F)CC(F)F | Rucaparib 295 |
| CNCc1ccc(cc1)c2c3CCNC(=O)c4cc(F)cc(c34)n2OC5=CC(=O)NC(=N5)N | Rucaparib 413 |
| CNCc1ccc(cc1)c2c3CCNC(=O)c4cc(F)cc(c34)n2OCCn5cncn5 | Rucaparib 419 |
| CNCc1ccc(cc1)c2c3CCNC(=O)c4cc(F)cc(c34)n2C(=O)O[C@H](C)c5ccc(F)cc5OC6CCOCC6 | Rucaparib 158 |
| CNCc1ccc(cc1)c2c3CCNC(=O)c4cc(F)cc(c34)n2C(=O)OCC5=CNC(=O)NC5=O | Rucaparib 132 |
| CNCc1ccc(cc1)c2c3CCNC(=O)c4cc(F)cc(c34)n2Oc5cccc(c5)[C@H]6CCC(=O)N6 | Rucaparib 351 |
| CNCc1ccc(cc1)c2c3CCNC(=O)c4cc(F)cc(c34)n2Oc5ccc(C(=O)O)c(OC(F)(F)F)c5 | Rucaparib 306 |
| CNCc1ccc(cc1)c2c3CCNC(=O)c4cc(F)cc(c34)n2Oc5ccc6c(NCCN7CCCC7)ccnc6c5 | Rucaparib 359 |
| CNCc1ccc(cc1)c2c3CCNC(=O)c4cc(F)cc(c34)n2Oc5ccc(cn5)C(=O)NCCC(=O)N | Rucaparib 310 |
| CNCc1ccc(cc1)c2c3CCNC(=O)c4cc(F)cc(c34)n2Oc5cc(O)c6ccccc6n5 | Rucaparib 430 |
| CCOCC(=O)On1c(c2CCNC(=O)c3cc(F)cc1c23)c4ccc(CNC)cc4 | Rucaparib 205 |
| CNCc1ccc(cc1)c2c3CCNC(=O)c4cc(F)cc(c34)n2Oc5ccc(cc5)c6oc(nn6)c7ccc(F)cc7 | Rucaparib 364 |
| CNCc1ccc(cc1)c2c3CCNC(=O)c4cc(F)cc(c34)n2Oc5ccc6nc(cn6c5)c7ccc(C)cc7 | Rucaparib 367 |
| CNCc1ccc(cc1)c2c3CCNC(=O)c4cc(F)cc(c34)n2Oc5c(F)cccc5F | Rucaparib 412 |
| CNCc1ccc(cc1)c2c3CCNC(=O)c4cc(F)cc(c34)n2OC5=CC(=O)NC(=O)N5 | Rucaparib 302 |
| CNCc1ccc(cc1)c2c3CCNC(=O)c4cc(F)cc(c34)n2OC5=NO[C@@H](C5)[C@H](N)C(=O)O | Rucaparib 324 |
| CNCc1ccc(cc1)c2c3CCNC(=O)c4cc(F)cc(c34)n2Oc5nc(OC)nc(OC)n5 | Rucaparib 327 |
| CNCc1ccc(cc1)c2c3CCNC(=O)c4cc(F)cc(c34)n2OC5=Cc6ccccc6NC5=O | Rucaparib 431 |
| CNCc1ccc(cc1)c2c3CCNC(=O)c4cc(F)cc(c34)n2CNC(=O)OC | Rucaparib 238 |
| CNCc1ccc(cc1)c2c3CCNC(=O)c4cc(F)cc(c34)n2Oc5ccc(OCCn6cnc7ccccc67)cc5 | Rucaparib 360 |
| CNCc1ccc(cc1)c2c3CCNC(=O)c4cc(F)cc(c34)n2Cc5c(F)cccc5F | Rucaparib 255 |
| CNCc1ccc(cc1)c2c3CCNC(=O)c4cc(F)cc(c34)n2Oc5c[n+]([O-])ccn5 | Rucaparib 340 |
| CNCc1ccc(cc1)c2c3CCNC(=O)c4cc(F)cc(c34)n2OCNC(=S)NC | Rucaparib 375 |
| CNCc1ccc(cc1)c2c3CCNC(=O)c4cc(F)cc(c34)n2C(=O)OCCn5cncn5 | Rucaparib 154 |
| CNCc1ccc(cc1)c2c3CCNC(=O)c4cc(F)cc(c34)n2O[C@H]5CCO[C@H](C5)c6oc(cc6)[C@H]7C[C@@H]7C | Rucaparib 437 |
| CNCc1ccc(cc1)c2c3CCNC(=O)c4cc(F)cc(c34)n2C(=O)OC[C@H](N)c5ccccn5 | Rucaparib 139 |
| CNCc1ccc(cc1)c2c3CCNC(=O)c4cc(F)cc(c34)n2C(=O)O[C@H](C(=O)C)c5ccc(OC)cc5 | Rucaparib 93 |
| CNCc1ccc(cc1)c2c3CCNC(=O)c4cc(F)cc(c34)n2Oc5ccc(C(=O)N(C)[C@@H](C)CCN)c(F)c5 | Rucaparib 300 |
| CNCc1ccc(cc1)c2c3CCNC(=O)c4cc(F)cc(c34)n2Oc5cccc(c5)n6cccc6 | Rucaparib 432 |
| CNCc1ccc(cc1)c2c3CCNC(=O)c4cc(F)cc(c34)n2NC(=O)C(=C(F)F)F | Rucaparib 4 |
| CNCc1ccc(cc1)c2c3CCNC(=O)c4cc(F)cc(c34)n2Oc5ccc6c(ccn6Cc7cc(C)on7)c5 | Rucaparib 369 |
| CNCc1ccc(cc1)c2c3CCNC(=O)c4cc(F)cc(c34)n2Cc5ccc(Cl)cc5 | Rucaparib 282 |
| CNCc1ccc(cc1)c2c3CCNC(=O)c4cc(F)cc(c34)n2Oc5cncc[n+]5[O-] | Rucaparib 341 |
| CNCc1ccc(cc1)c2c3CCNC(=O)c4cc(F)cc(c34)n2OC(=O)C[C@@H](C)C=C | Rucaparib 199 |
| CNCc1ccc(cc1)c2c3CCNC(=O)c4cc(F)cc(c34)n2OC5=C(C)NC(=O)NC5=O | Rucaparib 312 |
| CNCc1ccc(cc1)c2c3CCNC(=O)c4cc(F)cc(c34)n2Oc5ccc6c(c5)C(=O)c7ccccc67 | Rucaparib 372 |
| CNCc1ccc(cc1)c2c3CCNC(=O)c4cc(F)cc(c34)n2C(=O)OCc5ncc(C=O)cc5Cl | Rucaparib 99 |
| CNCc1ccc(cc1)c2c3CCNC(=O)c4cc(F)cc(c34)n2C(=O)Oc5ccc(OC)cc5N | Rucaparib 135 |
| CNCc1ccc(cc1)c2c3CCNC(=O)c4cc(F)cc(c34)n2Oc5ccc6cccnc6c5 | Rucaparib 356 |
| CNCc1ccc(cc1)c2c3CCNC(=O)c4cc(F)cc(c34)n2Oc5ccc6oc(nc6c5)c7ccccc7 | Rucaparib 371 |
| CC\C(=C\CCn1c(c2CCNC(=O)c3cc(F)cc1c23)c4ccc(CNC)cc4)\C | Rucaparib 248 |
| CC\C(=C\C(=O)On1c(c2CCNC(=O)c3cc(F)cc1c23)c4ccc(CNC)cc4)\C | Rucaparib 198 |
| CNCc1ccc(cc1)c2c3CCNC(=O)c4cc(F)cc(c34)n2Oc5nc(Cl)n[nH]5 | Rucaparib 321 |
| CNCc1ccc(cc1)c2c3CCNC(=O)c4cc(F)cc(c34)n2OC5=CC(=O)N(C)C(=O)N5 | Rucaparib 334 |
| CNCc1ccc(cc1)c2c3CCNC(=O)c4cc(F)cc(c34)n2C(=O)OCc5cncc(c5)c6cncs6 | Rucaparib 168 |
| CNCc1ccc(cc1)c2c3CCNC(=O)c4cc(F)cc(c34)n2Oc5ccc(cc5)N(O)C(=O)C | Rucaparib 317 |
| CNCc1ccc(cc1)c2c3CCNC(=O)c4cc(F)cc(c34)n2Oc5ccc(c(c5)C(=O)O)C(C)(C)C#N | Rucaparib 311 |
| CNCc1ccc(cc1)c2c3CCNC(=O)c4cc(F)cc(c34)n2Oc5ccc6cc[nH]c6c5 | Rucaparib 434 |
| CNCc1ccc(cc1)c2c3CCNC(=O)c4cc(F)cc(c34)n2Oc5ccc6CN(C(=N)c6c5)c7ccc(F)c(F)c7 | Rucaparib 363 |
| CNCc1ccc(cc1)c2c3CCNC(=O)c4cc(F)cc(c34)n2CCCCC=C | Rucaparib 230 |
| CNCc1ccc(cc1)c2c3CCNC(=O)c4cc(F)cc(c34)n2C(=O)Oc5nc(C)c(cc5C#N)C(F)(F)F | Rucaparib 82 |
| CNCc1ccc(cc1)c2c3CCNC(=O)c4cc(F)cc(c34)n2CCNCCN | Rucaparib 239 |
| CNCc1ccc(cc1)c2c3CCNC(=O)c4cc(F)cc(c34)n2OC5=C(O)C(=O)C5=O | Rucaparib 418 |
| CNCc1ccc(cc1)c2c3CCNC(=O)c4cc(F)cc(c34)n2O[C@H]5CN(CC(=O)N)C(=O)C5 | Rucaparib 401 |
| CNCc1ccc(cc1)c2c3CCNC(=O)c4cc(F)cc(c34)n2C(=O)OCc5cc(N)ccc5C6(CC6)C#N | Rucaparib 170 |
| CNCc1ccc(cc1)c2c3CCNC(=O)c4cc(F)cc(c34)n2Oc5nccc(N)n5 | Rucaparib 342 |
| CNCc1ccc(cc1)c2c3CCNC(=O)c4cc(F)cc(c34)n2Oc5ccc6COCc6c5 | Rucaparib 433 |
| CNCc1ccc(cc1)c2c3CCNC(=O)c4cc(F)cc(c34)n2Oc5ccc6CN(C(=N)c6c5)c7ccc(F)c(Cl)c7 | Rucaparib 358 |
| CNCc1ccc(cc1)c2c3CCNC(=O)c4cc(F)cc(c34)n2[C@H](F)C(F)(F)OC(F)F | Rucaparib 219 |
| CNCc1ccc(cc1)c2c3CCNC(=O)c4cc(F)cc(c34)n2C(=O)OC[C@@H]5CCC(=O)N5 | Rucaparib 151 |
| CNCc1ccc(cc1)c2c3CCNC(=O)c4cc(F)cc(c34)n2OC5=C(O)C=CC=C(O)C5=O | Rucaparib 402 |
| CNCc1ccc(cc1)c2c3CCNC(=O)c4cc(F)cc(c34)n2C(=O)OCCN5CCNC5=O | Rucaparib 144 |
| CNCc1ccc(cc1)c2c3CCNC(=O)c4cc(F)cc(c34)n2O[C@H]5CCCN(Cc6ccnc(c6)C#N)C5 | Rucaparib 425 |
| CNCc1ccc(cc1)c2c3CCNC(=O)c4cc(F)cc(c34)n2O[C@H]5CCO[C@H](C5)C6CCCC6 | Rucaparib 428 |
| CNCc1ccc(cc1)c2c3CCNC(=O)c4cc(F)cc(c34)n2Oc5ccc(cn5)[C@H]6C[C@@H]7CC[C@H]6N7 | Rucaparib 373 |
| CNCc1ccc(cc1)c2c3CCNC(=O)c4cc(F)cc(c34)n2C(=O)OC5=CC(=O)NC(=N5)N | Rucaparib 147 |
| CNCc1ccc(cc1)c2c3CCNC(=O)c4cc(F)cc(c34)n2C(=O)Oc5ccc(cc5O)[C@H](C#N)N6CCOCC6 | Rucaparib 159 |
| CNCc1ccc(cc1)c2c3CCNC(=O)c4cc(F)cc(c34)n2CCCC(C)C | Rucaparib 249 |
| CNCc1ccc(cc1)c2c3CCNC(=O)c4cc(F)cc(c34)n2C(=O)O[C@H](C)COc5cccc6ccccc56 | Rucaparib 165 |
| CNCc1ccc(cc1)c2c3CCNC(=O)c4cc(F)cc(c34)n2OC5=C(Cl)[C@@H](O)OC5=O | Rucaparib 332 |
| CNCc1ccc(cc1)c2c3CCNC(=O)c4cc(F)cc(c34)n2OC5=CC=CC(=C(O)C5=O)O | Rucaparib 403 |
| CNCc1ccc(cc1)c2c3CCNC(=O)c4cc(F)cc(c34)n2C(=O)OC[C@H]5NC[C@H](O)[C@@H]5O | Rucaparib 143 |
| CNCc1ccc(cc1)c2c3CCNC(=O)c4cc(F)cc(c34)n2C(=O)OCCN[C@]5(CCCS(=O)(=O)C5)C#N | Rucaparib 76 |
| CNCc1ccc(cc1)c2c3CCNC(=O)c4cc(F)cc(c34)n2C(=O)Oc5cc(CN)on5 | Rucaparib 152 |
| CNCc1ccc(cc1)c2c3CCNC(=O)c4cc(F)cc(c34)n2Oc5ccc(cc5)C(=O)COC | Rucaparib 333 |
| CNCc1ccc(cc1)c2c3CCNC(=O)c4cc(F)cc(c34)n2OC5=CN([C@@H]6CCCO6)C(=O)NC5=O | Rucaparib 350 |
| CNCc1ccc(cc1)c2c3CCNC(=O)c4cc(F)cc(c34)n2C(=O)O[C@@H](CN5C[C@H](C)OC[C@H]5C)C(F)(F)F | Rucaparib 74 |
| CNCc1ccc(cc1)c2c3CCNC(=O)c4cc(F)cc(c34)n2C(=O)OCc5cnc(N)nc5N | Rucaparib 134 |
| CNCc1ccc(cc1)c2c3CCNC(=O)c4cc(F)cc(c34)n2OC5=C(Cl)C(=O)O[C@@H]5O | Rucaparib 331 |
| CNCc1ccc(cc1)c2c3CCNC(=O)c4cc(F)cc(c34)n2C(=O)Oc5ccc(CN)cc5O | Rucaparib 137 |
| CCOc1nc(Cl)nc(On2c(c3CCNC(=O)c4cc(F)cc2c34)c5ccc(CNC)cc5)n1 | Rucaparib 314 |
| CNCc1ccc(cc1)c2c3CCNC(=O)c4cc(F)cc(c34)n2OCCc5cn[nH]c5 | Rucaparib 420 |
| CNCc1ccc(cc1)c2c3CCNC(=O)c4cc(F)cc(c34)n2C(=O)OCCN5C(=O)Sc6ccccc56 | Rucaparib 166 |
| CNCc1ccc(cc1)c2c3CCNC(=O)c4cc(F)cc(c34)n2C(=O)OC[C@@H](N(C)C)c5ccc(F)cc5F | Rucaparib 83 |
| CNCc1ccc(cc1)c2c3CCNC(=O)c4cc(F)cc(c34)n2Oc5ccc(NC(=O)CN(C)[C@H]6CCNC6)c(F)c5 | Rucaparib 344 |
| CNCc1ccc(cc1)c2c3CCNC(=O)c4cc(F)cc(c34)n2Oc5ccc(C(=O)CSC)c(F)c5 | Rucaparib 313 |
| CNCc1ccc(cc1)c2c3CCNC(=O)c4cc(F)cc(c34)n2C(=O)Oc5ncccc5N | Rucaparib 156 |
| CNCc1ccc(cc1)c2c3CCNC(=O)c4cc(F)cc(c34)n2Oc5ccc(C(=O)C)c(O)c5 | Rucaparib 308 |
| CNCc1ccc(cc1)c2c3CCNC(=O)c4cc(F)cc(c34)n2C(=O)Oc5cc(C=O)cc(OC)c5OC | Rucaparib 92 |
| CNCc1ccc(cc1)c2c3CCNC(=O)c4cc(F)cc(c34)n2C(=O)OCc5cccnc5OC | Rucaparib 138 |
| CNCc1ccc(cc1)c2c3CCNC(=O)c4cc(F)cc(c34)n2Oc5c(C)cccc5O | Rucaparib 416 |
| CNCc1ccc(cc1)c2c3CCNC(=O)c4cc(F)cc(c34)n2O[C@H]5CN([C@@H](C)Cn6ccnc6)C(=O)C5 | Rucaparib 426 |
| CNCc1ccc(cc1)c2c3CCNC(=O)c4cc(F)cc(c34)n2C(=O)OCc5cc(Br)ccc5OC | Rucaparib 77 |
| CNCc1ccc(cc1)c2c3CCNC(=O)c4cc(F)cc(c34)n2C(=O)O[C@H](COC)c5c(C)ccnc5N | Rucaparib 90 |
| CNCc1ccc(cc1)c2c3CCNC(=O)c4cc(F)cc(c34)n2Oc5c(N)cccc5O | Rucaparib 415 |
| CNCc1ccc(cc1)c2c3CCNC(=O)c4cc(F)cc(c34)n2C(=O)OCc5cnccn5 | Rucaparib 157 |
| CNCc1ccc(cc1)c2c3CCNC(=O)c4cc(F)cc(c34)n2C(=O)OCc5cccc(N)n5 | Rucaparib 148 |
| CNCc1ccc(cc1)c2c3CCNC(=O)c4cc(F)cc(c34)n2Oc5cc(Cl)ccc5O | Rucaparib 406 |
| CNCc1ccc(cc1)c2c3CCNC(=O)c4cc(F)cc(c34)n2O[C@@H]5CCN[C@@H](C5)[C@@H]6CCN(C6)C(=O)OC(C)(C)C | Rucaparib 343 |
| CNCc1ccc(cc1)c2c3CCNC(=O)c4cc(F)cc(c34)n2C(=O)OCc5ccc(O)c(Br)c5 | Rucaparib 80 |
| CNCc1ccc(cc1)c2c3CCNC(=O)c4cc(F)cc(c34)n2C(=O)OCc5cc(OC)c(O)c(OC)c5 | Rucaparib 89 |
| CNCc1ccc(cc1)c2c3CCNC(=O)c4cc(F)cc(c34)n2C(=O)OCCOc5c(F)c(F)c(F)c(F)c5F | Rucaparib 73 |
| CCCCOc1ccc(On2c(c3CCNC(=O)c4cc(F)cc2c34)c5ccc(CNC)cc5)nn1 | Rucaparib 316 |
| CNCc1ccc(cc1)c2c3CCNC(=O)c4cc(F)cc(c34)n2Oc5ccc(cc5)[N+]#N | Rucaparib 318 |
| CNCc1ccc(cc1)c2c3CCNC(=O)c4cc(F)cc(c34)n2Oc5ccc(NC(=S)N)cc5 | Rucaparib 396 |
| CNCc1ccc(cc1)c2c3CCNC(=O)c4cc(F)cc(c34)n2Cc5ccc(N)cc5 | Rucaparib 285 |
| CNCc1ccc(cc1)c2c3CCNC(=O)c4cc(F)cc(c34)n2Oc5ccc(COc6ccc(cc6)[C@H](C)N)s5 | Rucaparib 345 |
| CNCc1ccc(cc1)c2c3CCNC(=O)c4cc(F)cc(c34)n2O[C@H]5CN(CC(F)(F)F)C(=O)C5 | Rucaparib 391 |
| CNCc1ccc(cc1)c2c3CCNC(=O)c4cc(F)cc(c34)n2C(=O)Oc5nc(O)cc(C)c5C#N | Rucaparib 125 |
| CNCc1ccc(cc1)c2c3CCNC(=O)c4cc(F)cc(c34)n2Oc5cc(C)c(C#N)c(O)n5 | Rucaparib 405 |
| CNCc1ccc(cc1)c2c3CCNC(=O)c4cc(F)cc(c34)n2Oc5ccc(cc5)[C@H](O)Cn6ccnc6 | Rucaparib 348 |
| CNCc1ccc(cc1)c2c3CCNC(=O)c4cc(F)cc(c34)n2NC(=O)CN5CCC5 | Rucaparib 16 |
| CNCc1ccc(cc1)c2c3CCNC(=O)c4cc(F)cc(c34)n2C(=O)OCCC#Cc5cnccn5 | Rucaparib 126 |
| CNCc1ccc(cc1)c2c3CCNC(=O)c4cc(F)cc(c34)n2Cc5ccccc5 | Rucaparib 263 |
| CNCc1ccc(cc1)c2c3CCNC(=O)c4cc(F)cc(c34)n2C(=O)OCc5ccc6NC(=O)CCCc6c5 | Rucaparib 169 |
| CNCc1ccc(cc1)c2c3CCNC(=O)c4cc(F)cc(c34)n2C(=O)Oc5nc(O)sc5Br | Rucaparib 85 |
| CNCc1ccc(cc1)c2c3CCNC(=O)c4cc(F)cc(c34)n2Oc5ccc(\C=[N+](/[O-])\c6ccccc6)cc5 | Rucaparib 349 |
| CNCc1ccc(cc1)c2c3CCNC(=O)c4cc(F)cc(c34)n2O[C@@H]5CCCN(C5)[C@@H](C)COC | Rucaparib 394 |
| CNCc1ccc(cc1)c2c3CCNC(=O)c4cc(F)cc(c34)n2C(=O)OC[C@H]5OC(C)(C)O[C@@H]5CO | Rucaparib 105 |
| CNCc1ccc(cc1)c2c3CCNC(=O)c4cc(F)cc(c34)n2C(=O)OC[C@]5(C)COC(=N5)C | Rucaparib 145 |
| CNCc1ccc(cc1)c2c3CCNC(=O)c4cc(F)cc(c34)n2C(=O)OCc5oc(CN)cc5 | Rucaparib 146 |
| CNCc1ccc(cc1)c2c3CCNC(=O)c4cc(F)cc(c34)n2Oc5ccc6cc(C=O)c(Cl)nc6c5 | Rucaparib 347 |
| CNCc1ccc(cc1)c2c3CCNC(=O)c4cc(F)cc(c34)n2C(=O)OCc5sccc5OC | Rucaparib 131 |
| CNCc1ccc(cc1)c2c3CCNC(=O)c4cc(F)cc(c34)n2OC(=O)C(=O)OC | Rucaparib 208 |
| CNCc1ccc(cc1)c2c3CCNC(=O)c4cc(F)cc(c34)n2Oc5cccc(c5)[C@@]6(O)CCCC[C@@H]6CN(C)C | Rucaparib 422 |
| CNCc1ccc(cc1)c2c3CCNC(=O)c4cc(F)cc(c34)n2C(=O)OCC#Cc5ccc6ccccc6n5 | Rucaparib 171 |
| CNCc1ccc(cc1)c2c3CCNC(=O)c4cc(F)cc(c34)n2C(=O)OCCNC(=O)c5ccc(Cl)cc5 | Rucaparib 84 |
| CCCCC(=O)c1ccc(On2c(c3CCNC(=O)c4cc(F)cc2c34)c5ccc(CNC)cc5)cc1 | Rucaparib 322 |
| CNCc1ccc(cc1)c2c3CCNC(=O)c4cc(F)cc(c34)n2C(=O)OCc5ccc(OC)c(O)c5 | Rucaparib 111 |
| CNCc1ccc(cc1)c2c3CCNC(=O)c4cc(F)cc(c34)n2C(=O)OCCN5CCOC5 | Rucaparib 150 |
| CNCc1ccc(cc1)c2c3CCNC(=O)c4cc(F)cc(c34)n2C(=O)OCC5CCN(Cc6ccccc6)CC5 | Rucaparib 163 |
| CNCc1ccc(cc1)c2c3CCNC(=O)c4cc(F)cc(c34)n2O[C@H]5CCO[C@@H](C[C@H](C)c6ccccc6)C5 | Rucaparib 424 |
| CNCc1ccc(cc1)c2c3CCNC(=O)c4cc(F)cc(c34)n2C(=O)OCCc5cn[nH]c5 | Rucaparib 155 |
| CNCc1ccc(cc1)c2c3CCNC(=O)c4cc(F)cc(c34)n2OC(=O)C(=O)\C=C\C | Rucaparib 200 |
| CC[C@H]1C[C@H](CCO1)Nc2ccc(On3c(c4CCNC(=O)c5cc(F)cc3c45)c6ccc(CNC)cc6)cc2 | Rucaparib 423 |
| CNCc1ccc(cc1)c2c3CCNC(=O)c4cc(F)cc(c34)n2C(=O)OCc5cc(C)cc(CO)c5OC | Rucaparib 91 |
| CNCc1ccc(cc1)c2c3CCNC(=O)c4cc(F)cc(c34)n2Oc5ccc(F)cn5 | Rucaparib 326 |
| CNCc1ccc(cc1)c2c3CCNC(=O)c4cc(F)cc(c34)n2Oc5ccc(OCC(=O)[O-])c(Cl)c5 | Rucaparib 307 |
| CNCc1ccc(cc1)c2c3CCNC(=O)c4cc(F)cc(c34)n2C(=O)OCc5ccc(cc5)C(=O)C | Rucaparib 124 |
| CNCc1ccc(cc1)c2c3CCNC(=O)c4cc(F)cc(c34)n2O[C@@H]5CC[C@H](CC5)OCc6ccccc6 | Rucaparib 427 |
| CNCc1ccc(cc1)c2c3CCNC(=O)c4cc(F)cc(c34)n2Oc5cnc(nc5)C#N | Rucaparib 339 |
| CNCc1ccc(cc1)c2c3CCNC(=O)c4cc(F)cc(c34)n2c5nncn5CCSC | Rucaparib 225 |
| CNCc1ccc(cc1)c2c3CCNC(=O)c4cc(F)cc(c34)n2OC(=O)C(=O)[C@@H](C)F | Rucaparib 190 |
| CNCc1ccc(cc1)c2c3CCNC(=O)c4cc(F)cc(c34)n2O[C@@H]5CCCN(C5)C(=O)CS(=O)(=O)C | Rucaparib 387 |
| CNCc1ccc(cc1)c2c3CCNC(=O)c4cc(F)cc(c34)n2C(=O)OC5=CC(=O)NC(=C5)C(F)(F)F | Rucaparib 94 |
| CNCc1ccc(cc1)c2c3CCNC(=O)c4cc(F)cc(c34)n2NC(=O)CC5CC5 | Rucaparib 17 |
| CNCc1ccc(cc1)c2c3CCNC(=O)c4cc(F)cc(c34)n2Oc5ccc(cc5)\C(=N\O)\Cl | Rucaparib 303 |
| CNCc1ccc(cc1)c2c3CCNC(=O)c4cc(F)cc(c34)n2Oc5cc(CN)on5 | Rucaparib 417 |
| CNCc1ccc(cc1)c2c3CCNC(=O)c4cc(F)cc(c34)n2O[C@H](C)CC(=O)N | Rucaparib 385 |
| CNCc1ccc(cc1)c2c3CCNC(=O)c4cc(F)cc(c34)n2C(=O)OCCS(=O)(=O)c5ccccc5 | Rucaparib 87 |
| CNCc1ccc(cc1)c2c3CCNC(=O)c4cc(F)cc(c34)n2C(=O)OCC(C)(C)CN5CCSC[C@@H]5C | Rucaparib 79 |
| CNCc1ccc(cc1)c2c3CCNC(=O)c4cc(F)cc(c34)n2Cc5ccc(Cl)c(Cl)c5 | Rucaparib 281 |
| CNCc1ccc(cc1)c2c3CCNC(=O)c4cc(F)cc(c34)n2OC(F)(F)C(=O)C(=O)O | Rucaparib 293 |
| CNCc1ccc(cc1)c2c3CCNC(=O)c4cc(F)cc(c34)n2C(=O)Oc5ccc(cc5)S(=O)(=O)C | Rucaparib 98 |
| CNCc1ccc(cc1)c2c3CCNC(=O)c4cc(F)cc(c34)n2OC(=O)CN5CCC5 | Rucaparib 217 |
| CNCc1ccc(cc1)c2c3CCNC(=O)c4cc(F)cc(c34)n2C(=O)OCC5=CC=CN(C)C5=O | Rucaparib 136 |
| CNCc1ccc(cc1)c2c3CCNC(=O)c4cc(F)cc(c34)n2Oc5cc(Cl)c(OC(=O)C)c(Cl)c5 | Rucaparib 301 |
| CNCc1ccc(cc1)c2c3CCNC(=O)c4cc(F)cc(c34)n2Oc5n[nH]c(Br)n5 | Rucaparib 320 |
| CNCc1ccc(cc1)c2c3CCNC(=O)c4cc(F)cc(c34)n2OCCCNC=O | Rucaparib 384 |
| CNCc1ccc(cc1)c2c3CCNC(=O)c4cc(F)cc(c34)n2Oc5ccc(NS(=O)(=O)C)cc5 | Rucaparib 390 |
| CC[C@@H](OC(=O)n1c(c2CCNC(=O)c3cc(F)cc1c23)c4ccc(CNC)cc4)c5ccc(O)cc5 | Rucaparib 116 |
| CNCc1ccc(cc1)c2c3CCNC(=O)c4cc(F)cc(c34)n2\C=C\CC5CCCC5 | Rucaparib 283 |
| CNCc1ccc(cc1)c2c3CCNC(=O)c4cc(F)cc(c34)n2OC(=O)[C@H](N)COC | Rucaparib 191 |
| CNCc1ccc(cc1)c2c3CCNC(=O)c4cc(F)cc(c34)n2OC(=O)CC5CC5 | Rucaparib 218 |
| CNCc1ccc(cc1)c2c3CCNC(=O)c4cc(F)cc(c34)n2C(=O)OC5=C(C)OCC5=O | Rucaparib 153 |
| CNCc1ccc(cc1)c2c3CCNC(=O)c4cc(F)cc(c34)n2C(=O)OCCN5C[C@@H](F)[C@H](F)C5 | Rucaparib 121 |
| CNCc1ccc(cc1)c2c3CCNC(=O)c4cc(F)cc(c34)n2OC5=CNC(=O)N=C5 | Rucaparib 328 |
| CNCc1ccc(cc1)c2c3CCNC(=O)c4cc(F)cc(c34)n2OC(=O)\C=C(/C)\Cl | Rucaparib 189 |
| CNCc1ccc(cc1)c2c3CCNC(=O)c4cc(F)cc(c34)n2Oc5ccc(\C=N\NC(=S)N)cc5 | Rucaparib 389 |
| CNCc1ccc(cc1)c2c3CCNC(=O)c4cc(F)cc(c34)n2C(=O)Oc5ccc(SC)c(C)c5 | Rucaparib 110 |
| CNCc1ccc(cc1)c2c3CCNC(=O)c4cc(F)cc(c34)n2OCC(=O)[C@@H](O)CO | Rucaparib 376 |
| CCOc1ccc(Cn2c(c3CCNC(=O)c4cc(F)cc2c34)c5ccc(CNC)cc5)cc1 | Rucaparib 258 |
| CNCc1ccc(cc1)c2c3CCNC(=O)c4cc(F)cc(c34)n2Cc5ccc(OC)cc5 | Rucaparib 260 |
| CNCc1ccc(cc1)c2c3CCNC(=O)c4cc(F)cc(c34)n2C(=O)Oc5nnc(s5)C(C)C | Rucaparib 130 |
| CNCc1ccc(cc1)c2c3CCNC(=O)c4cc(F)cc(c34)n2C(=O)OCCCN5CCOCC5 | Rucaparib 127 |
| CNCc1ccc(cc1)c2c3CCNC(=O)c4cc(F)cc(c34)n2C(=O)O[C@H](C)CN5CCNCC5 | Rucaparib 128 |
| CNCc1ccc(cc1)c2c3CCNC(=O)c4cc(F)cc(c34)n2OC(=O)C(=C(F)F)F | Rucaparib 186 |
| CNCc1ccc(cc1)c2c3CCNC(=O)c4cc(F)cc(c34)n2C(=O)OCCN(C)[C@@H]5CCCNC(=O)C5 | Rucaparib 86 |
| CNCc1ccc(cc1)c2c3CCNC(=O)c4cc(F)cc(c34)n2C(=O)Oc5ccc(CO)cc5Br | Rucaparib 81 |
| CCC(=O)[C@@H](C(=O)OC)n1c(c2CCNC(=O)c3cc(F)cc1c23)c4ccc(CNC)cc4 | Rucaparib 220 |
| CNCc1ccc(cc1)c2c3CCNC(=O)c4cc(F)cc(c34)n2Cc5ccc(OC)c(F)c5 | Rucaparib 254 |
| CNCc1ccc(cc1)c2c3CCNC(=O)c4cc(F)cc(c34)n2C(=O)OCCN5CCOCC5 | Rucaparib 18 |
| CNCc1ccc(cc1)c2c3CCNC(=O)c4cc(F)cc(c34)n2C(=O)OCCCN5CCNCC5 | Rucaparib 129 |
| CNCc1ccc(cc1)c2c3CCNC(=O)c4cc(F)cc(c34)n2CCC(=O)OC | Rucaparib 240 |
| CNCc1ccc(cc1)c2c3CCNC(=O)c4cc(F)cc(c34)n2C(=O)OCCCN5CCC[C@H](N)C5=O | Rucaparib 97 |
| CNCc1ccc(cc1)c2c3CCNC(=O)c4cc(F)cc(c34)n2C(=O)OCc5ccc(Cl)cc5O | Rucaparib 109 |
| CNCc1ccc(cc1)c2c3CCNC(=O)c4cc(F)cc(c34)n2OC(=O)\C=C(/C)\CO | Rucaparib 196 |
| CNCc1ccc(cc1)c2c3CCNC(=O)c4cc(F)cc(c34)n2Cc5ccc(OC(F)F)cc5 | Rucaparib 253 |
| CNCc1ccc(cc1)c2c3CCNC(=O)c4cc(F)cc(c34)n2Cc5ccc(cc5)C#N | Rucaparib 264 |
| CNCc1ccc(cc1)c2c3CCNC(=O)c4cc(F)cc(c34)n2C(=O)OCc5ccccc5C=O | Rucaparib 142 |
| CCC(=O)Nc1ccc(On2c(c3CCNC(=O)c4cc(F)cc2c34)c5ccc(CNC)cc5)cc1 | Rucaparib 398 |
| CNCc1ccc(cc1)c2c3CCNC(=O)c4cc(F)cc(c34)n2OC(=O)C(F)(F)C(F)(F)F | Rucaparib 176 |
| CNCc1ccc(cc1)c2c3CCNC(=O)c4cc(F)cc(c34)n2C(=O)OCCCC(=O)N5CCOCC5 | Rucaparib 95 |
| CCc1cc(Br)ccc1COC(=O)n2c(c3CCNC(=O)c4cc(F)cc2c34)c5ccc(CNC)cc5 | Rucaparib 78 |
| CNCc1ccc(cc1)c2c3CCNC(=O)c4cc(F)cc(c34)n2OC(=O)[C@H](N)C=C | Rucaparib 215 |
| CNCc1ccc(cc1)c2c3CCNC(=O)c4cc(F)cc(c34)n2[C@@H]5CCOC5 | Rucaparib 290 |
| CNCc1ccc(cc1)c2c3CCNC(=O)c4cc(F)cc(c34)n2C(=O)OCc5ccccc5N | Rucaparib 149 |
| CNCc1ccc(cc1)c2c3CCNC(=O)c4cc(F)cc(c34)n2C(=O)OCCc5ccccc5N | Rucaparib 141 |
| CNCc1ccc(cc1)c2c3CCNC(=O)c4cc(F)cc(c34)n2NC(=O)C(F)(F)F | Rucaparib 8 |
| CNCc1ccc(cc1)c2c3CCNC(=O)c4cc(F)cc(c34)n2C(=O)OCCC(=O)Nc5ccccc5 | Rucaparib 102 |
| CNCc1ccc(cc1)c2c3CCNC(=O)c4cc(F)cc(c34)n2C(=O)OCCc5ccc(F)cc5Br | Rucaparib 75 |
| CNCc1ccc(cc1)c2c3CCNC(=O)c4cc(F)cc(c34)n2C(=O)OCNC(=O)c5ccccc5 | Rucaparib 120 |
| CNCc1ccc(cc1)c2c3CCNC(=O)c4cc(F)cc(c34)n2NC(=O)C(=O)[C@@H](C)F | Rucaparib 7 |
| CNCc1ccc(cc1)c2c3CCNC(=O)c4cc(F)cc(c34)n2Cc5ccc(F)cc5 | Rucaparib 284 |
| CNCc1ccc(cc1)c2c3CCNC(=O)c4cc(F)cc(c34)n2OC5=CC(=O)NC(=C5)C | Rucaparib 414 |
| CNCc1ccc(cc1)c2c3CCNC(=O)c4cc(F)cc(c34)n2Oc5ccc(CC(=N)N)cc5 | Rucaparib 309 |
| CNCc1ccc(cc1)c2c3CCNC(=O)c4cc(F)cc(c34)n2OC(=O)C(F)(F)Cl | Rucaparib 185 |
| CNCc1ccc(cc1)c2c3CCNC(=O)c4cc(F)cc(c34)n2C(=O)OCNC(=O)Cc5ccccc5 | Rucaparib 103 |
| CNCc1ccc(cc1)c2c3CCNC(=O)c4cc(F)cc(c34)n2Oc5ccc(NC(=O)OC(C)(C)C)cc5 | Rucaparib 305 |
| CNCc1ccc(cc1)c2c3CCNC(=O)c4cc(F)cc(c34)n2NC(=O)C[C@H](C)O | Rucaparib 11 |
| CNCc1ccc(cc1)c2c3CCNC(=O)c4cc(F)cc(c34)n2NC(=O)CS(=O)(=O)C | Rucaparib 3 |
| CNCc1ccc(cc1)c2c3CCNC(=O)c4cc(F)cc(c34)n2Oc5ccc(Br)nc5 | Rucaparib 325 |
| CNCc1ccc(cc1)c2c3CCNC(=O)c4cc(F)cc(c34)n2Oc5cccc(c5)C(=N)N | Rucaparib 336 |
| CC[C@H](O)CCOn1c(c2CCNC(=O)c3cc(F)cc1c23)c4ccc(CNC)cc4 | Rucaparib 382 |
| CNCc1ccc(cc1)c2c3CCNC(=O)c4cc(F)cc(c34)n2C(=O)OCCCc5ccc(O)c(O)c5 | Rucaparib 101 |
| CNCc1ccc(cc1)c2c3CCNC(=O)c4cc(F)cc(c34)n2O\C(=C(\Br)/C(=O)O)\C(=O)O | Rucaparib 291 |
| CNCc1ccc(cc1)c2c3CCNC(=O)c4cc(F)cc(c34)n2C(=O)OCCNc5ccncc5C | Rucaparib 113 |
| CNCc1ccc(cc1)c2c3CCNC(=O)c4cc(F)cc(c34)n2OC(=O)C[C@H](C)N | Rucaparib 209 |
| CC[C@@H](COC(=O)n1c(c2CCNC(=O)c3cc(F)cc1c23)c4ccc(CNC)cc4)NC(=O)N | Rucaparib 28 |
| CNCc1ccc(cc1)c2c3CCNC(=O)c4cc(F)cc(c34)n2O[C@H]5CCO[C@@H](CC(C)(C)C)C5 | Rucaparib 395 |
| CNCc1ccc(cc1)c2c3CCNC(=O)c4cc(F)cc(c34)n2Oc5ccc(F)c(CC(=O)O)c5 | Rucaparib 330 |
| CNCc1ccc(cc1)c2c3CCNC(=O)c4cc(F)cc(c34)n2CCOC(C)C | Rucaparib 269 |
| CNCc1ccc(cc1)c2c3CCNC(=O)c4cc(F)cc(c34)n2Oc5ccc(C[C@@H](N)C(=O)O)c(Cl)c5 | Rucaparib 304 |
| CNCc1ccc(cc1)c2c3CCNC(=O)c4cc(F)cc(c34)n2C(=O)O[C@H](CN)CP(=O)(C)O | Rucaparib 23 |
| CNCc1ccc(cc1)c2c3CCNC(=O)c4cc(F)cc(c34)n2OC(=O)CN(C)C | Rucaparib 210 |
| CNCc1ccc(cc1)c2c3CCNC(=O)c4cc(F)cc(c34)n2Oc5nc(O)c(Br)s5 | Rucaparib 388 |
| CNCc1ccc(cc1)c2c3CCNC(=O)c4cc(F)cc(c34)n2C(=O)O[C@H]([C@H](C)O)[C@@H](O)C=O | Rucaparib 27 |
| CCCC[C@H]1C[C@H](CCO1)On2c(c3CCNC(=O)c4cc(F)cc2c34)c5ccc(CNC)cc5 | Rucaparib 400 |
| CNCc1ccc(cc1)c2c3CCNC(=O)c4cc(F)cc(c34)n2Oc5cccc(c5)C(=O)O | Rucaparib 335 |
| CCOCCn1c(c2CCNC(=O)c3cc(F)cc1c23)c4ccc(CNC)cc4 | Rucaparib 275 |
| CNCc1ccc(cc1)c2c3CCNC(=O)c4cc(F)cc(c34)n2C(=O)OC[C@@H](CO)c5ccccn5 | Rucaparib 112 |
| CC[C@@H]1CCN(CCOC(=O)n2c(c3CCNC(=O)c4cc(F)cc2c34)c5ccc(CNC)cc5)C(=O)CC1 | Rucaparib 88 |
| CNCc1ccc(cc1)c2c3CCNC(=O)c4cc(F)cc(c34)n2C[C@@H](N)C(=O)O | Rucaparib 223 |
| CNCc1ccc(cc1)c2c3CCNC(=O)c4cc(F)cc(c34)n2C(=O)OC[C@H](O)C(=O)CO | Rucaparib 38 |
| CNCc1ccc(cc1)c2c3CCNC(=O)c4cc(F)cc(c34)n2Cc5ccsc5 | Rucaparib 287 |
| CNCc1ccc(cc1)c2c3CCNC(=O)c4cc(F)cc(c34)n2Oc5cccc(CNN)c5 | Rucaparib 408 |
| CNCc1ccc(cc1)c2c3CCNC(=O)c4cc(F)cc(c34)n2CCCOC | Rucaparib 243 |
| CNCc1ccc(cc1)c2c3CCNC(=O)c4cc(F)cc(c34)n2C(=O)O[C@H](C)CN5CC[C@H](C)[C@@H](N)C5 | Rucaparib 96 |
| CNCc1ccc(cc1)c2c3CCNC(=O)c4cc(F)cc(c34)n2C(=O)OCC5(C)COC(C)(C)OC5 | Rucaparib 106 |
| CNCc1ccc(cc1)c2c3CCNC(=O)c4cc(F)cc(c34)n2OCC[C@@H](O)CO | Rucaparib 381 |
| CNCc1ccc(cc1)c2c3CCNC(=O)c4cc(F)cc(c34)n2C(=O)OC[C@H](O)[C@H](O)C=O | Rucaparib 43 |
| CNCc1ccc(cc1)c2c3CCNC(=O)c4cc(F)cc(c34)n2Cc5occc5 | Rucaparib 289 |
| CNCc1ccc(cc1)c2c3CCNC(=O)c4cc(F)cc(c34)n2Oc5ccc(NO)cc5 | Rucaparib 338 |
| CNCc1ccc(cc1)c2c3CCNC(=O)c4cc(F)cc(c34)n2CCC=C | Rucaparib 237 |
| CNCc1ccc(cc1)c2c3CCNC(=O)c4cc(F)cc(c34)n2C(=O)OCCO[C@H]5CCNC[C@@H]5C | Rucaparib 107 |
| CNCc1ccc(cc1)c2c3CCNC(=O)c4cc(F)cc(c34)n2C(=O)OC[C@H](N)C(=O)N | Rucaparib 65 |
| CNCc1ccc(cc1)c2c3CCNC(=O)c4cc(F)cc(c34)n2OCC[C@H](O)CO | Rucaparib 379 |
| CNCc1ccc(cc1)c2c3CCNC(=O)c4cc(F)cc(c34)n2OC(=O)C[C@H](N)C(=O)[O-] | Rucaparib 184 |
| CNCc1ccc(cc1)c2c3CCNC(=O)c4cc(F)cc(c34)n2CC5CCOCC5 | Rucaparib 286 |
| CNCc1ccc(cc1)c2c3CCNC(=O)c4cc(F)cc(c34)n2OC5C[C@H](O)C[C@H](O)C5 | Rucaparib 411 |
| CCCCCn1c(c2CCNC(=O)c3cc(F)cc1c23)c4ccc(CNC)cc4 | Rucaparib 232 |
| CNCc1ccc(cc1)c2c3CCNC(=O)c4cc(F)cc(c34)n2C(=O)OC[C@H](O)CN5CCCCC5 | Rucaparib 108 |
| CNCc1ccc(cc1)c2c3CCNC(=O)c4cc(F)cc(c34)n2C(=O)O[C@@H](NC(=O)N)C(Cl)(Cl)Cl | Rucaparib 19 |
| CNCc1ccc(cc1)c2c3CCNC(=O)c4cc(F)cc(c34)n2OC(=O)C=C(C)C | Rucaparib 216 |
| CNCc1ccc(cc1)c2c3CCNC(=O)c4cc(F)cc(c34)n2O\C(=C(\Cl)/C=O)\C(=O)O | Rucaparib 292 |
| CNCc1ccc(cc1)c2c3CCNC(=O)c4cc(F)cc(c34)n2C[C@@H]5CCCO5 | Rucaparib 288 |
| CNCc1ccc(cc1)c2c3CCNC(=O)c4cc(F)cc(c34)n2Oc5ccc(CCC(=O)C)cc5 | Rucaparib 399 |
| CNCc1ccc(cc1)c2c3CCNC(=O)c4cc(F)cc(c34)n2CC(C)C | Rucaparib 236 |
| CNCc1ccc(cc1)c2c3CCNC(=O)c4cc(F)cc(c34)n2C(=O)OC\C=C\c5ccc(OC)cc5 | Rucaparib 104 |
| CNCc1ccc(cc1)c2c3CCNC(=O)c4cc(F)cc(c34)n2OC(=O)[C@H]([15NH2])C[15NH]O | Rucaparib 188 |
| CNCc1ccc(cc1)c2c3CCNC(=O)c4cc(F)cc(c34)n2NC(=O)\C=C(/C)\Cl | Rucaparib 6 |
| CNCc1ccc(cc1)c2c3CCNC(=O)c4cc(F)cc(c34)n2C(=O)Oc5ccc(CCC=O)cc5 | Rucaparib 123 |
| CNCc1ccc(cc1)c2c3CCNC(=O)c4cc(F)cc(c34)n2C(=O)OC[C@H](O)[C@H](O)C=C | Rucaparib 45 |
| CNCc1ccc(cc1)c2c3CCNC(=O)c4cc(F)cc(c34)n2C5CCC5 | Rucaparib 261 |
| CNCc1ccc(cc1)c2c3CCNC(=O)c4cc(F)cc(c34)n2Oc5ccc(\C=C\CO)cc5 | Rucaparib 404 |
| CCCCn1c(c2CCNC(=O)c3cc(F)cc1c23)c4ccc(CNC)cc4 | Rucaparib 245 |
| CNCc1ccc(cc1)c2c3CCNC(=O)c4cc(F)cc(c34)n2C(=O)OC\C=C\c5ccc(O)cc5 | Rucaparib 122 |
| CNCc1ccc(cc1)c2c3CCNC(=O)c4cc(F)cc(c34)n2C(=O)OCCNc5cccc(C)c5 | Rucaparib 118 |
| CNCc1ccc(cc1)c2c3CCNC(=O)c4cc(F)cc(c34)n2NC(=O)\C=C\Cl | Rucaparib 9 |
| CNCc1ccc(cc1)c2c3CCNC(=O)c4cc(F)cc(c34)n2OCC(=O)C(=O)O | Rucaparib 297 |
| CNCc1ccc(cc1)c2c3CCNC(=O)c4cc(F)cc(c34)n2C(=O)OCCOc5ccccc5 | Rucaparib 140 |
| CNCc1ccc(cc1)c2c3CCNC(=O)c4cc(F)cc(c34)n2C(=O)OCNC(=O)NCO | Rucaparib 36 |
| CNCc1ccc(cc1)c2c3CCNC(=O)c4cc(F)cc(c34)n2Oc5ccc(CC[C@@H](C)O)cc5 | Rucaparib 397 |
| CNCc1ccc(cc1)c2c3CCNC(=O)c4cc(F)cc(c34)n2NC(=O)CC(C)(F)F | Rucaparib 5 |
| CNCc1ccc(cc1)c2c3CCNC(=O)c4cc(F)cc(c34)n2Oc5ccc(CCNC(=O)C)cc5 | Rucaparib 393 |
| CNCc1ccc(cc1)c2c3CCNC(=O)c4cc(F)cc(c34)n2C(=O)OC[C@@H](C)Nc5ccccc5 | Rucaparib 119 |
| CNC[C@@H](CO)OC(=O)n1c(c2CCNC(=O)c3cc(F)cc1c23)c4ccc(CNC)cc4 | Rucaparib 58 |
| CNCc1ccc(cc1)c2c3CCNC(=O)c4cc(F)cc(c34)n2Cc5ccc(OC)c(C)c5 | Rucaparib 257 |
| CCS(=O)(=O)CCOC(=O)n1c(c2CCNC(=O)c3cc(F)cc1c23)c4ccc(CNC)cc4 | Rucaparib 26 |
| CNCc1ccc(cc1)c2c3CCNC(=O)c4cc(F)cc(c34)n2Oc5cccc(c5)[C@@H](C)N | Rucaparib 410 |
| CNCc1ccc(cc1)c2c3CCNC(=O)c4cc(F)cc(c34)n2OC(=O)CCCC#C | Rucaparib 203 |
| CCCC(=O)Nc1ccc(On2c(c3CCNC(=O)c4cc(F)cc2c34)c5ccc(CNC)cc5)cc1 | Rucaparib 392 |
| CCOc1ccc(COC(=O)n2c(c3CCNC(=O)c4cc(F)cc2c34)c5ccc(CNC)cc5)cc1 | Rucaparib 115 |
| CNCc1ccc(cc1)c2c3CCNC(=O)c4cc(F)cc(c34)n2NC(=O)\C=C\OC | Rucaparib 15 |
| CNCc1ccc(cc1)c2c3CCNC(=O)c4cc(F)cc(c34)n2CC5CCCCC5 | Rucaparib 256 |
| CNCc1ccc(cc1)c2c3CCNC(=O)c4cc(F)cc(c34)n2OC(=O)CS(=O)(=O)C | Rucaparib 181 |
| CNCc1ccc(cc1)c2c3CCNC(=O)c4cc(F)cc(c34)n2Oc5cccc(CCO)c5 | Rucaparib 409 |
| CNCc1ccc(cc1)c2c3CCNC(=O)c4cc(F)cc(c34)n2OC(=O)[C@@H](F)C(F)(F)F | Rucaparib 178 |
| CNCc1ccc(cc1)c2c3CCNC(=O)c4cc(F)cc(c34)n2C(=O)OCc5ccccc5[C@@H](C)O | Rucaparib 114 |
| CNCc1ccc(cc1)c2c3CCNC(=O)c4cc(F)cc(c34)n2NC(=O)CCC=O | Rucaparib 14 |
| CNCc1ccc(cc1)c2c3CCNC(=O)c4cc(F)cc(c34)n2C(=O)OCP(=O)(CO)CO | Rucaparib 25 |
| CNCc1ccc(cc1)c2c3CCNC(=O)c4cc(F)cc(c34)n2Oc5ccc(N)cc5 | Rucaparib 421 |
| CNCc1ccc(cc1)c2c3CCNC(=O)c4cc(F)cc(c34)n2CCCC(=O)C | Rucaparib 270 |
| CNCc1ccc(cc1)c2c3CCNC(=O)c4cc(F)cc(c34)n2C(=O)OCC[C@@H](O)c5ccccc5 | Rucaparib 117 |
| CNCc1ccc(cc1)c2c3CCNC(=O)c4cc(F)cc(c34)n2OC(=O)\C=C\CO | Rucaparib 213 |
| CNCc1ccc(cc1)c2c3CCNC(=O)c4cc(F)cc(c34)n2C(=O)O[C@H](CO)[C@H](O)C=O | Rucaparib 42 |
| CNCc1ccc(cc1)c2c3CCNC(=O)c4cc(F)cc(c34)n2CC5OCCO5 | Rucaparib 259 |
| CNCc1ccc(cc1)c2c3CCNC(=O)c4cc(F)cc(c34)n2C\C=C\CN | Rucaparib 278 |
| CNCc1ccc(cc1)c2c3CCNC(=O)c4cc(F)cc(c34)n2C(=O)OCP(CO)c5ccccc5 | Rucaparib 100 |
| CNCc1ccc(cc1)c2c3CCNC(=O)c4cc(F)cc(c34)n2NC(=O)CN(C)C | Rucaparib 13 |
| CNCc1ccc(cc1)c2c3CCNC(=O)c4cc(F)cc(c34)n2C(=O)O[C@H](CO)C(=O)CO | Rucaparib 37 |
| CNCc1ccc(cc1)c2c3CCNC(=O)c4cc(F)cc(c34)n2C(=O)O[C@@H]([C@@H](O)C(=O)C)C(=O)C | Rucaparib 24 |
| CNCc1ccc(cc1)c2c3CCNC(=O)c4cc(F)cc(c34)n2CCCC=O | Rucaparib 233 |
| CNCc1ccc(cc1)c2c3CCNC(=O)c4cc(F)cc(c34)n2C(=O)OCCNC(=N)N | Rucaparib 67 |
| CNCc1ccc(cc1)c2c3CCNC(=O)c4cc(F)cc(c34)n2NC(=O)C[C@H](C)N | Rucaparib 12 |
| CNCc1ccc(cc1)c2c3CCNC(=O)c4cc(F)cc(c34)n2C(=O)OCNC(=S)NC | Rucaparib 35 |
| CNCc1ccc(cc1)c2c3CCNC(=O)c4cc(F)cc(c34)n2C(=O)O[C@H](CO)[C@@H](O)C=O | Rucaparib 39 |
| CNCc1ccc(cc1)c2c3CCNC(=O)c4cc(F)cc(c34)n2CCCCN | Rucaparib 231 |
| CNCc1ccc(cc1)c2c3CCNC(=O)c4cc(F)cc(c34)n2OC(=O)C[C@@H](C)O | Rucaparib 206 |
| CNCc1ccc(cc1)c2c3CCNC(=O)c4cc(F)cc(c34)n2C(=O)OCCNC(=O)N | Rucaparib 64 |
| CNCc1ccc(cc1)c2c3CCNC(=O)c4cc(F)cc(c34)n2C(=O)O[C@H](C=O)[C@H](O)CO | Rucaparib 40 |
| CNCc1ccc(cc1)c2c3CCNC(=O)c4cc(F)cc(c34)n2CCCN | Rucaparib 235 |
| CNCc1ccc(cc1)c2c3CCNC(=O)c4cc(F)cc(c34)n2[C@H](CN)C(=O)O | Rucaparib 224 |
| CNCc1ccc(cc1)c2c3CCNC(=O)c4cc(F)cc(c34)n2C(=O)OCP(=O)(O)CO | Rucaparib 33 |
| CNCc1ccc(cc1)c2c3CCNC(=O)c4cc(F)cc(c34)n2C(=O)OC[C@@H](O)[C@@H](O)C=O | Rucaparib 41 |
| CNCc1ccc(cc1)c2c3CCNC(=O)c4cc(F)cc(c34)n2\C=C\CC(C)C | Rucaparib 272 |
| CNCc1ccc(cc1)c2c3CCNC(=O)c4cc(F)cc(c34)n2O\C=C\CON | Rucaparib 296 |
| CNCc1ccc(cc1)c2c3CCNC(=O)c4cc(F)cc(c34)n2OC(=O)C[C@@H](O)C(=O)O | Rucaparib 183 |
| CNCc1ccc(cc1)c2c3CCNC(=O)c4cc(F)cc(c34)n2OCCS(=O)(=O)O | Rucaparib 374 |
| CNCc1ccc(cc1)c2c3CCNC(=O)c4cc(F)cc(c34)n2CCCC=C | Rucaparib 279 |
| CNCc1ccc(cc1)c2c3CCNC(=O)c4cc(F)cc(c34)n2OC[C@H](O)COC | Rucaparib 380 |
| CNCc1ccc(cc1)c2c3CCNC(=O)c4cc(F)cc(c34)n2OC(=O)CNC(=O)N | Rucaparib 193 |
| CNCc1ccc(cc1)c2c3CCNC(=O)c4cc(F)cc(c34)n2C=C(C)C | Rucaparib 252 |
| CNCc1ccc(cc1)c2c3CCNC(=O)c4cc(F)cc(c34)n2COC(=O)C=C | Rucaparib 242 |
| CNCc1ccc(cc1)c2c3CCNC(=O)c4cc(F)cc(c34)n2C(=O)O[C@@H](CC(=O)N)\C=C\C | Rucaparib 31 |
| CCCn1c(c2CCNC(=O)c3cc(F)cc1c23)c4ccc(CNC)cc4 | Rucaparib 229 |
| CCCC[C@H](CC)n1c(c2CCNC(=O)c3cc(F)cc1c23)c4ccc(CNC)cc4 | Rucaparib 265 |
| CCC[C@H](C(=O)O)n1c(c2CCNC(=O)c3cc(F)cc1c23)c4ccc(CNC)cc4 | Rucaparib 221 |
| CNCc1ccc(cc1)c2c3CCNC(=O)c4cc(F)cc(c34)n2C(=O)OCC(=O)N(C(C)C)C(C)C | Rucaparib 22 |
| CNCc1ccc(cc1)c2c3CCNC(=O)c4cc(F)cc(c34)n2C(=O)OCC(C)(C)NC(=O)C | Rucaparib 29 |
| CNCc1ccc(cc1)c2c3CCNC(=O)c4cc(F)cc(c34)n2C(=O)OCNC(=O)C=C | Rucaparib 71 |
| CCC[C@H](CC)n1c(c2CCNC(=O)c3cc(F)cc1c23)c4ccc(CNC)cc4 | Rucaparib 250 |
| CNCc1ccc(cc1)c2c3CCNC(=O)c4cc(F)cc(c34)n2OC(=O)CC(C)(F)F | Rucaparib 187 |
| CNCc1ccc(cc1)c2c3CCNC(=O)c4cc(F)cc(c34)n2C(=O)O[C@H](CO)[C@H](O)C=C | Rucaparib 44 |
| CNCc1ccc(cc1)c2c3CCNC(=O)c4cc(F)cc(c34)n2OC(=O)\C=C\C(=O)[O-] | Rucaparib 197 |
| CNCc1ccc(cc1)c2c3CCNC(=O)c4cc(F)cc(c34)n2CCOC(=O)C | Rucaparib 228 |
| CNCc1ccc(cc1)c2c3CCNC(=O)c4cc(F)cc(c34)n2C(=O)OCCNC(=O)C | Rucaparib 69 |
| CNCc1ccc(cc1)c2c3CCNC(=O)c4cc(F)cc(c34)n2C(=O)OCCCC(=O)N | Rucaparib 68 |
| CNCc1ccc(cc1)c2c3CCNC(=O)c4cc(F)cc(c34)n2\C=C\CON | Rucaparib 222 |
| CNCc1ccc(cc1)c2c3CCNC(=O)c4cc(F)cc(c34)n2C(=O)OCC(=O)CCO | Rucaparib 66 |
| CNCc1ccc(cc1)c2c3CCNC(=O)c4cc(F)cc(c34)n2OC(=O)CCC(=O)N | Rucaparib 194 |
| CNCc1ccc(cc1)c2c3CCNC(=O)c4cc(F)cc(c34)n2OC(=O)C(F)(F)F | Rucaparib 201 |
| CNCc1ccc(cc1)c2c3CCNC(=O)c4cc(F)cc(c34)n2OC(=O)CSC(=O)C | Rucaparib 182 |
| CNCc1ccc(cc1)c2c3CCNC(=O)c4cc(F)cc(c34)n2\C=C\CN(C)C | Rucaparib 271 |
| CNC[C@H](O)COC(=O)n1c(c2CCNC(=O)c3cc(F)cc1c23)c4ccc(CNC)cc4 | Rucaparib 59 |
| CNCc1ccc(cc1)c2c3CCNC(=O)c4cc(F)cc(c34)n2OC(=O)CCC(=O)[O-] | Rucaparib 195 |
| CNCc1ccc(cc1)c2c3CCNC(=O)c4cc(F)cc(c34)n2\C=C\COC | Rucaparib 277 |
| CNCc1ccc(cc1)c2c3CCNC(=O)c4cc(F)cc(c34)n2C(=O)OC[C@@H](O)COC | Rucaparib 55 |
| CNCc1ccc(cc1)c2c3CCNC(=O)c4cc(F)cc(c34)n2C(=O)OC[C@@H](N)[C@@H](C)O | Rucaparib 60 |
| CNCc1ccc(cc1)c2c3CCNC(=O)c4cc(F)cc(c34)n2[C@H](C)COC | Rucaparib 274 |
| CNCc1ccc(cc1)c2c3CCNC(=O)c4cc(F)cc(c34)n2C(=O)OC[C@@H](O)CCO | Rucaparib 53 |
| CNCc1ccc(cc1)c2c3CCNC(=O)c4cc(F)cc(c34)n2C(=O)OCC[C@H](O)CO | Rucaparib 54 |
| CNCc1ccc(cc1)c2c3CCNC(=O)c4cc(F)cc(c34)n2CCCCO | Rucaparib 276 |
| CNCc1ccc(cc1)c2c3CCNC(=O)c4cc(F)cc(c34)n2C(=O)OCC(CO)CO | Rucaparib 52 |
| CNCc1ccc(cc1)c2c3CCNC(=O)c4cc(F)cc(c34)n2C(=O)OCC[C@@H](O)CO | Rucaparib 56 |
| CNCc1ccc(cc1)c2c3CCNC(=O)c4cc(F)cc(c34)n2CCN | Rucaparib 241 |
| CNCc1ccc(cc1)c2c3CCNC(=O)c4cc(F)cc(c34)n2C(=O)OC[C@H](CO)OC | Rucaparib 57 |
| CNCc1ccc(cc1)c2c3CCNC(=O)c4cc(F)cc(c34)n2OC(=O)[C@H](O)CCN | Rucaparib 192 |
| CNCc1ccc(cc1)c2c3CCNC(=O)c4cc(F)cc(c34)n2CCCC#N | Rucaparib 244 |
| CNCc1ccc(cc1)c2c3CCNC(=O)c4cc(F)cc(c34)n2OC[C@H](O)[C@H](O)C=O | Rucaparib 377 |
| CNCc1ccc(cc1)c2c3CCNC(=O)c4cc(F)cc(c34)n2CC=C | Rucaparib 246 |
| CNCc1ccc(cc1)c2c3CCNC(=O)c4cc(F)cc(c34)n2OC(=O)CC(=O)C | Rucaparib 212 |
| CCn1c(c2CCNC(=O)c3cc(F)cc1c23)c4ccc(CNC)cc4 | Rucaparib 234 |
| CNCc1ccc(cc1)c2c3CCNC(=O)c4cc(F)cc(c34)n2OCNC(=O)C=C | Rucaparib 386 |
| CNCc1ccc(cc1)c2c3CCNC(=O)c4cc(F)cc(c34)n2OC(=O)\C=C\OC | Rucaparib 214 |
| CNCc1ccc(cc1)c2c3CCNC(=O)c4cc(F)cc(c34)n2OC(=O)CCC=O | Rucaparib 211 |
| CNCc1ccc(cc1)c2c3CCNC(=O)c4cc(F)cc(c34)n2OC(=O)C[C@H](C)O | Rucaparib 207 |
| CC[C@](C)(O)COC(=O)n1c(c2CCNC(=O)c3cc(F)cc1c23)c4ccc(CNC)cc4 | Rucaparib 63 |
| CNCc1ccc(cc1)c2c3CCNC(=O)c4cc(F)cc(c34)n2OCCC(=O)CO | Rucaparib 383 |
| CNCc1ccc(cc1)c2c3CCNC(=O)c4cc(F)cc(c34)n2C(=O)OCCSC(C)C | Rucaparib 34 |
| CNCc1ccc(cc1)c2c3CCNC(=O)c4cc(F)cc(c34)n2CCOC | Rucaparib 280 |
| CNCc1ccc(cc1)c2c3CCNC(=O)c4cc(F)cc(c34)n2C(=O)OCC(=C)CC(C)(C)C | Rucaparib 32 |
| CC[C@H](O)CCOC(=O)n1c(c2CCNC(=O)c3cc(F)cc1c23)c4ccc(CNC)cc4 | Rucaparib 62 |
| CNCc1ccc(cc1)c2c3CCNC(=O)c4cc(F)cc(c34)n2C=C | Rucaparib 226 |
| CNCc1ccc(cc1)c2c3CCNC(=O)c4cc(F)cc(c34)n2NC(=O)\C=C\I | Rucaparib 1 |
| CC[C@H](CCO)OC(=O)n1c(c2CCNC(=O)c3cc(F)cc1c23)c4ccc(CNC)cc4 | Rucaparib 61 |
| CCC(CC)n1c(c2CCNC(=O)c3cc(F)cc1c23)c4ccc(CNC)cc4 | Rucaparib 251 |
| CNCc1ccc(cc1)c2c3CCNC(=O)c4cc(F)cc(c34)n2OC(=O)\C=C\I | Rucaparib 175 |
| CCSCCOC(=O)n1c(c2CCNC(=O)c3cc(F)cc1c23)c4ccc(CNC)cc4 | Rucaparib 51 |
| CNCc1ccc(cc1)c2c3CCNC(=O)c4cc(F)cc(c34)n2C | Rucaparib 227 |
| CCC[C@H](OC(=O)n1c(c2CCNC(=O)c3cc(F)cc1c23)c4ccc(CNC)cc4)C(C)(C)C | Rucaparib 30 |
| CNCc1ccc(cc1)c2c3CCNC(=O)c4cc(F)cc(c34)n2C(=O)OCCCSC | Rucaparib 50 |
| CC[C@H](C)[C@H](C)COC(=O)n1c(c2CCNC(=O)c3cc(F)cc1c23)c4ccc(CNC)cc4 | Rucaparib 47 |
| CNCc1ccc(cc1)c2c3CCNC(=O)c4cc(F)cc(c34)n2OC(=O)CCCC#N | Rucaparib 202 |
| CC[C@@H](OC(=O)n1c(c2CCNC(=O)c3cc(F)cc1c23)c4ccc(CNC)cc4)C(C)C | Rucaparib 70 |
| CNCc1ccc(cc1)c2c3CCNC(=O)c4cc(F)cc(c34)n2OC(=O)\C=C\Cl | Rucaparib 204 |
| CNCc1ccc(cc1)c2c3CCNC(=O)c4cc(F)cc(c34)n2C(=O)O[C@@H](CC#C)\C=C\C | Rucaparib 49 |
| CCCC(CCC)n1c(c2CCNC(=O)c3cc(F)cc1c23)c4ccc(CNC)cc4 | Rucaparib 266 |
| CC[C@@H](OC(=O)n1c(c2CCNC(=O)c3cc(F)cc1c23)c4ccc(CNC)cc4)C(Cl)(Cl)Cl | Rucaparib 20 |
| CNCc1ccc(cc1)c2c3CCNC(=O)c4cc(F)cc(c34)n2C(=O)OCCCC(C)(C)C | Rucaparib 46 |
| CNCc1ccc(cc1)c2c3CCNC(=O)c4cc(F)cc(c34)n2C(=O)OCCCC(=C)Br | Rucaparib 21 |
| CC[C@H](CC(C)C)OC(=O)n1c(c2CCNC(=O)c3cc(F)cc1c23)c4ccc(CNC)cc4 | Rucaparib 48 |
| CCCC\C=C/n1c(c2CCNC(=O)c3cc(F)cc1c23)c4ccc(CNC)cc4 | Rucaparib 273 |
